# Genome sequence of *Malania oleifera*, a tree with great value for nervonic acid production

**DOI:** 10.1093/gigascience/giy164

**Published:** 2019-01-24

**Authors:** Chao-Qun Xu, Hui Liu, Shan-Shan Zhou, Dong-Xu Zhang, Wei Zhao, Sihai Wang, Fu Chen, Yan-Qiang Sun, Shuai Nie, Kai-Hua Jia, Si-Qian Jiao, Ren-Gang Zhang, Quan-Zheng Yun, Wenbin Guan, Xuewen Wang, Qiong Gao, Jeffrey L Bennetzen, Fatemeh Maghuly, Ilga Porth, Yves Van de Peer, Xiao-Ru Wang, Yongpeng Ma, Jian-Feng Mao

**Affiliations:** 1 Beijing Advanced Innovation Center for Tree Breeding by Molecular Design, National Engineering Laboratory for Tree Breeding, School of Nature Conservation, College of Biological Sciences and Technology, Beijing Forestry University, Beijing, 100083, China; 2 College of Life Science, Datong University, Datong, Shanxi, 037009, China; 3 Yunnan Key Laboratory of Forest Plant Cultivation and Utilization, State Forestry Administration Key Laboratory of Yunnan Rare and Endangered Species Conservation and Propagation, Yunnan Academy of Forestry, Kunming, Yunnan, 650201, China; 4 The Camellia Institute, Yunnan Academy of Forestry, Guangnan, Yunnan, 663300, China; 5 Beijing Ori-Gene Science and Technology Co. Ltd, Beijing, 102206, China; 6 Department of Genetics, University of Georgia, Athens, GA 30602, USA; 7 Plant Biotechnology Unit (PBU), Dept. Biotechnology, BOKU-VIBT, University of Natural Resources and Life Sciences, Muthgasse 18, Vienna 1190, Austria; 8 Département des sciences du bois et de la forêt, 1030, Avenue de la Médecine, Université Laval, Québec (Québec) G1V 0A6, Canada; 9 Institute for System and Integrated Biology, Pavillon Charles-Eugène-Marchand, 1030, Avenue de la Médecine, Université Laval, Québec (Québec) G1V 0A6, Canada; 10 Centre d'Étude de la Forêt, 1030, Avenue de la Médecine, Université Laval, Québec (Québec) G1V 0A6, Canada; 11 Department of Plant Biotechnology and Bioinformatics, Ghent University, Ghent 9052, Belgium; 12 VIB Center for Plant Systems Biology, Ghent 9052, Belgium; 13 Centre for Microbial Ecology and Genomics, Department of Biochemistry, Genetics and Microbiology Genetics, University of Pretoria, Private bag X20, Pretoria 0028, South Africa; 14 Department of Ecology and Environmental Science, UPSC, Umeå University, Umeå SE-901 87, Sweden; 15 Yunnan Key Laboratory for Integrative Conservation of Plant Species with Extremely Small Population, Kunming Institute of Botany, Chinese Academy of Sciences, Kunming, 650201, China

**Keywords:** *de novo* genome assembly, vulnerable plant, *Malania*, nervonic acid, transcriptomes

## Abstract

**Background:**

*Malania oleifera*, a member of the Olacaceae family, is an IUCN red listed tree, endemic and restricted to the Karst region of southwest China. This tree's seed is valued for its high content of precious fatty acids (especially nervonic acid). However, studies on its genetic makeup and fatty acid biogenesis are severely hampered by a lack of molecular and genetic tools.

**Findings:**

We generated 51 Gb and 135 Gb of raw DNA sequences, using Pacific Biosciences (PacBio) single-molecule real-time and 10× Genomics sequencing, respectively. A final genome assembly, with a scaffold N50 size of 4.65 Mb and a total length of 1.51 Gb, was obtained by primary assembly based on PacBio long reads plus scaffolding with 10× Genomics reads. Identified repeats constituted ∼82% of the genome, and 24,064 protein-coding genes were predicted with high support. The genome has low heterozygosity and shows no evidence for recent whole genome duplication. Metabolic pathway genes relating to the accumulation of long-chain fatty acid were identified and studied in detail.

**Conclusions:**

Here, we provide the first genome assembly and gene annotation for *M. oleifera*. The availability of these resources will be of great importance for conservation biology and for the functional genomics of nervonic acid biosynthesis.

## Data Description

### Background information

**Figure 1:**
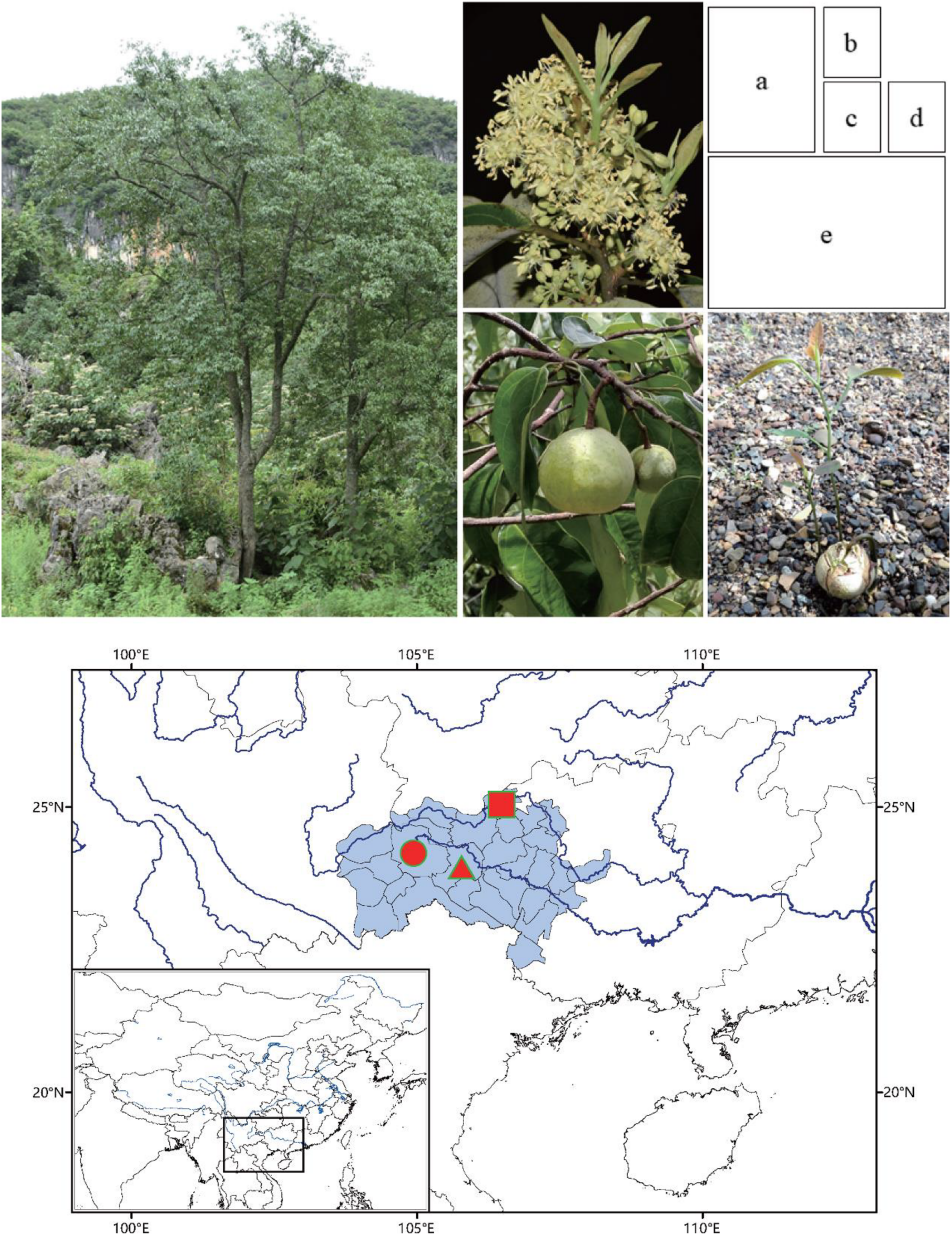
Images of *M. oleifera*, recorded distribution range, and sampling sites. Mature tree **(a)**, flower **(b)**, fruit **(c)**, and naturally germinated seedling **(d). (e)** Blue-shaded region denotes the reported distribution range of *M. oleifera*, while the red circle denotes the position (N23.90°, E104.09°, Guangnan County, Yunnan) where one tree was sampled for whole genome sequencing, and the red triangle and square denote the positions (N23.9°, E106.00°, Funing County, Yunnan, and N24.78°, E106.57°, Leye County, Guangxi) where trees were sampled for RNA sequencing.

*Malania oleifera* Chun & SK Lee (NCBI:txid397392), a 10- to 20-m tall tree (Fig. 1a–d), is from the monotypic genus *Malania* of the Olacaceae family [1]. This tree is endemic to a restricted area within the Karst topography of southwest Guangxi and southeast Yunnan provinces, China. The recorded distribution range is bounded by N23°23′–N24°28′ in latitude and E105°30′–E107°30′ in longitude (Fig. 1e). This tree is called "garlic-fruit tree" or ‘suantouguo’ (蒜头果) by local communities due to its garlic-shaped fruits. As an endemic tree and because of its natural populations being much reduced because of ongoing logging and habitat clearance, this tree species has been listed in the IUCN Red List as Vulnerable B1+2c (extent of occurrence estimated to be <20,000 km^2^ and a continuing decline, observed, projected, or inferred, in numbers of mature individuals) [2], and has been assigned as a plant species with an extremely small population size for urgent conservation action [3]. Different mechanisms that could explain why *M. oleifera* became a vulnerable species have been proposed, such as niche specialization [4], limited germination and regeneration [5, 6], or pollination/mating system [7], as well as the biology of its pathogens [8]. However, until now, apart from a recent chloroplast genome sequence [9], only a few molecular genetic resources are available for *M. oleifera* to investigate its population structure and genetic makeup.

Besides conservation urgency, *M. oleifera* is also notable for its substantial phytochemical and phytopharmaceutical value. Its seed has very high (64.5%) oil content [10, 11] and the highest-known proportion (55.70%–67%) of nervonic acid (C_24_H_46_O_2_, PubChem CID: 5281120). Nervonic acid is an important component in myelin biosynthesis in the central and peripheral nervous system. Myelin is generally localized to the sphingomyelin of animal cell membranes [12], where it has been proposed to enhance human brain function. Treatment of myelin disorders may attenuate or prevent various psychotic disorders [13, 14]. *Malania oleifera* produces essential oils with benzyl alcohol (58.42%) and benzaldehyde (29.66%) as the main constituents as well as benzoic acid (1.49%) [10]. *Malania oleifera* seeds also produce the glycoprotein malania, which has high cytotoxic activity towards tumor cells and is one of the most potent toxins of plant origin [15]. Yet, little is known about the molecular mechanisms underlying the metabolic biosynthesis processes of these promising compounds in *M. oleifera*.

Here, we present a high-quality genome assembly for *M. oleifera*, combining Pacific Biosciences (PacBio) single-molecule long reads and 10× Genomics linked reads. The assembled genome, its structural and functional annotation, and in-depth characterization will provide valuable tools for the genomic dissection of the species’ genetic diversity and its population demography for future conservation purposes, as well as for in-depth molecular knowledge regarding biosynthesis and regulation of metabolism to promote the efficient and sustainable exploitation of this precious biological resource.

### Plant material

One mature and healthy tree with abundant fruit (Fig. 1 a–d) was chosen as a tissue source for whole-genome sequencing. The selected tree measured ∼18 m in height, ∼35 cm in diameter (at breast height), and is believed to be ∼50 years old. This tree is located within a natural stand close to Diji Village, Jiumoxiang, Guangnan County, Yunnan Province, China (N23.90° latitude, E104.90° longitude, 1,402 m elevation) (Fig. 1e). The stand from which the samples were taken experienced little anthropogenic intervention and consists of trees of the same species but of different ages. Fresh leaves were sampled in September 2017.

For RNA sequencing, leaves, fruits, and seeds were sampled from healthy, high-yielding, mature trees from Funing County, Yunnan Province, and Leye County, Guangxi Province, China, in different seasons during the years 2013–2016 (Fig. 1e and Supplementary Table S1). Samples were immediately flash frozen in liquid nitrogen upon collection and transported on dry ice to Beijing Forestry University for sequence analysis.

All samples were collected with permission from and under the supervision of local forestry bureaus. See Supplementary Table S1 and Fig. 1 for more details.

### PacBio SMRT sequencing

High-quality and high-molecular-weight genomic DNA was extracted from leaves of the selected tree, following the ∼20 kb SMRTbell Libraries protocol [16]. DNA was purified using the Mobio PowerClean Pro DNA Clean-Up Kit, and its quality was assessed by standard agarose gel electrophoresis and Thermo Fisher Scientific Qubit Fluorometry. Genomic DNA was sheared to a size range of 15–50  Kb using either AMPure beads (Beckman Coulte) or g-TUBE (Covaris) and enzymatically repaired and converted into SMRTbell template libraries according to PacBio instructions. Following this procedure, hairpin adapters were ligated after exonuclease-based digestion (of the remaining damaged DNA fragments and those fragments without adapters at both ends). The resulting SMRTbell templates were subsequently size-selected by Blue Pippin electrophoresis (Sage Sciences). Templates ranging from 15 to 50  Kb were sequenced on a PacBio Sequel instrument using S/P2-C2 sequencing chemistry (10 SMRT cells). A total of 5,778,035 PacBio long reads were generated, yielding 51.15 Gb (roughly 30× coverage of the assembled genome) of single-molecule sequencing data with an average read length of 8,852 bp (Supplementary Fig. S1 and Table S1).

### 10× Genomics library preparation and Illumina sequencing

Purified high-molecular-weight genomic DNA of high quality was incubated with Proteinase K and RNaseA for 30  minutes at 25°C. DNA was further purified, indexed, and partitioned into bar coded libraries that were prepared using the GemCode kit (10× Genomics, Pleasanton, CA). Following the GemCode procedure, 1.0 ng of DNA was used for gel beads in emulsion (GEM) reactions in which DNA fragments were partitioned into molecular reactors to extend the DNA and to introduce specific 14-bp partition bar codes. Subsequently, GEM reactions were polymerase chain reaction (PCR)-amplified. The PCR cycling protocol was as follows: 95°C for 5  minutes; cycled 18×: 4°C for 30  seconds, 45°C for 1  second, 70°C for 20  seconds, and 98°C for 30  seconds; held at 4°C. The PCR products were purified as described in the GemCode protocol. Purified DNA was sheared, end-repaired, adenylation tailed, and universal adapter ligated, and samples were indexed according to the manufacturer's recommendations.

The whole genome GemCode library was sequenced using 2 × 150 paired-end (PE) sequencing on Illumina HiSeq X Ten. A total of 899.778 million reads (∼134.97 Gb, roughly 89× coverage of the assembled genome) were obtained, of which 89.1% had base quality values over 20 and 80% over 30 (Supplementary Table S1). There were 19,319,151 (99.98% of total read pairs) indexes assigned to more than one read pair, while 27,368 (9.55%), 830 (2.12%), and 450 (1.80%) had more than 1,000, 3,000, or 5,000 read pairs, respectively (Supplementary Table S2). Sequence data were analyzed using the GemCode Long Ranger Software Suite [17, 18].

### RNA sequencing

Frozen tissues were ground with a mortar and pestle, and RNA was isolated using the NEBNext Poly (A) mRNA Magnetic Isolation Module. RNA quality was determined on an Agilent 2100 BioAnalyzer. Seven sequencing libraries were prepared using the NEBNext Ultra RNA Library Prep Kit for Illumina. Next, 100/150 bp PE sequencing was performed on an Illumina HiSeq 2000/2500 machine. See Supplementary Table S1 for details.

### Estimation of genome size, heterozygosity, and repeat content

Canu v1.6 (Canu, RRID:SCR_015880) [19] was employed to filter and correct the PacBio reads. Next, *k*-mers were counted using Jellyfish (Jellyfish, RRID:SCR_005491) [20]. Finally, gce v1.0.0 [21] was used to estimate genome size, repeat content, and the level of heterozygosity. A total of 29,971,959,192 *k*-mers (size 17) were identified, and the peak *k*-mer depth obtained was 21 (Supplementary Fig. S2). The genome size was estimated to be ∼1.50 Gb (Supplementary Table S3). The final cleaned data corresponded to about 21-fold coverage. Repeat and error frequencies were estimated to be 54.61% and 0.34%, respectively. Heterozygosity was very low (∼0.06%). See Supplementary File 1 for commands and settings.

### *De novo* genome assembly and quality control

First, primary assemblies (eight from PacBio long reads, one from 10× Genomics linked reads) were prepared by different pipelines. Next, scaffolding and polishing were performed on the optimal primary assemblies in order to obtain a final genome assembly. Primary assembly v0.1 was generated from PacBio long reads after correction by Canu v1.6 [19], assembly v0.2 by MECAT v1.1 [22], assembly v0.3 by miniasm v0.2-r168 [23] after alignment by minimap v0.2-r124 [23], assembly v0.4 by Falcon v0.7 (Falcon, RRID:SCR_016089) [24, 25] after correction with Canu v1.6, assembly v0.5 by SMARTdenovo v1.0.0 [26] after correction with Canu v1.6, assembly v0.6 by Wtdbg v1.2.8 [27] after correction with Canu v1.6, assembly v0.7 by SMARTdenovo v1.0.0 after correction, and assembly v0.8 by Wtdbg v1.2.8. Assembly v0.9 was prepared by Supernova assembler 2.0 [28, 29] from 10× Genomics linked reads data. Based on quality-control parameters, assembly v0.7 was chosen as optimal for further scaffolding and polishing. It generated a reasonably sized assembly (1.51 Gb), providing the highest N50 (i.e., the shortest sequence length at 50% of the total genome assembly length) (1.12 Mb) and the lowest number of contigs (3,038) and L50 (i.e., the smallest number of contig sequences whose lengths sum produces the N50 value) (330). Furthermore, genome assembly version v0.7 exhibited the longest contig length (6.72 Mb), as well as 71.80% gene completeness as determined by Benchmarking Universal Single-Copy Orthologs (BUSCO) (BUSCO, RRID:SCR_015008) [30] assessment (Supplementary Table S4). This assembly (v0.7) was further polished with raw PacBio long reads using arrow v2.2.1 [31] to produce (in two rounds) assembly v1.0. Subsequently, 10× Genomics linked reads were processed with Long Ranger [17, 18], and were then aligned to v1.0 using BWA mem v0.7.15 (default values, *-t*12) (BWA, RRID:SCR_010910) [32] and subsequently scaffolded by ARCS v1.0.1 [33] to produce assembly v1.1. The final assembly was generated after one further iteration of polishing with arrow v2.21 and three iterations with Pilon v1.22 (Pilon, RRID:SCR_014731) [34]. Before arrow-based polishing, PacBio raw reads were aligned using BLASR v5.1 (BLASR, RRID:SCR_000764) [35, 36], and PacBio raw reads were mapped with Bowtie2 v2.2.6 (Bowtie2, RRID:SCR_016368) [37] before each iteration with Pilon. In the final assembly, a genome size of 1.51 Gb was obtained, consisting of 2,987 contigs, 1,277 scaffolds (with contig N50 of 1.22 Mb, scaffold N50 of 4.65 Mb, longest contig of 6.7 Mb, and longest scaffold of 25.1 Mb), and with a gene completeness of 90.60% (Table 1 and Supplementary Table S4).

**Table 1:** Statistics of the final genome assembly for *M. oleifera*.

	Contig		Scaffold	
	Size (bp)	Number	Size (bp)	Number
Total size	1,509,344,141	–	1,519,782,615	–
Total number	–	2,987	–	1,277
N10	2,959,726	39	11,755,999	10
N50	1,218,690	376	4,647,296	94
N90	272,293	1,337	1,153,659	339
Max.	6,703,356	–	25,060,663	–
Min.	334	–	8,256	–
Mean	505,304	–	1,190,119	–
Median	200,407	–	85,436	–
Gap	–	–	10,438,474 (0.69%)	1,710
GC content	36.07%	–	35.82%	–

–, Data not available.

The consistency of the predicted genome size based on *k*-mer characterization and the assembled genome indicated a good quality for our assembly. Furthermore, when all clean Illumina reads were mapped to the final assembly (v1.2f), a high sequence coverage of 98.5% was obtained. In addition, an even higher sequence coverage of 99.32% was observed for mapping PacBio long reads to the final assembly using BLASR. These two coverage values suggested high sequence completeness and fidelity of the genome assembly. Mapping rates (91%–98%) were also very high for transcriptomic datasets mapped to the final assembly, of which most (79%–96%) were uniquely mapped (Supplementary Table S1), with the exception of one RNA sequencing library (Short Read Archive accession: SRR7221534) that yielded low mapping rates (10.31%), a result that we cannot explain by anything aside from microbial or other contamination (see Supplementary File 1 for commands and settings).

### Transposable element and other repeat annotation

**Figure 2:**
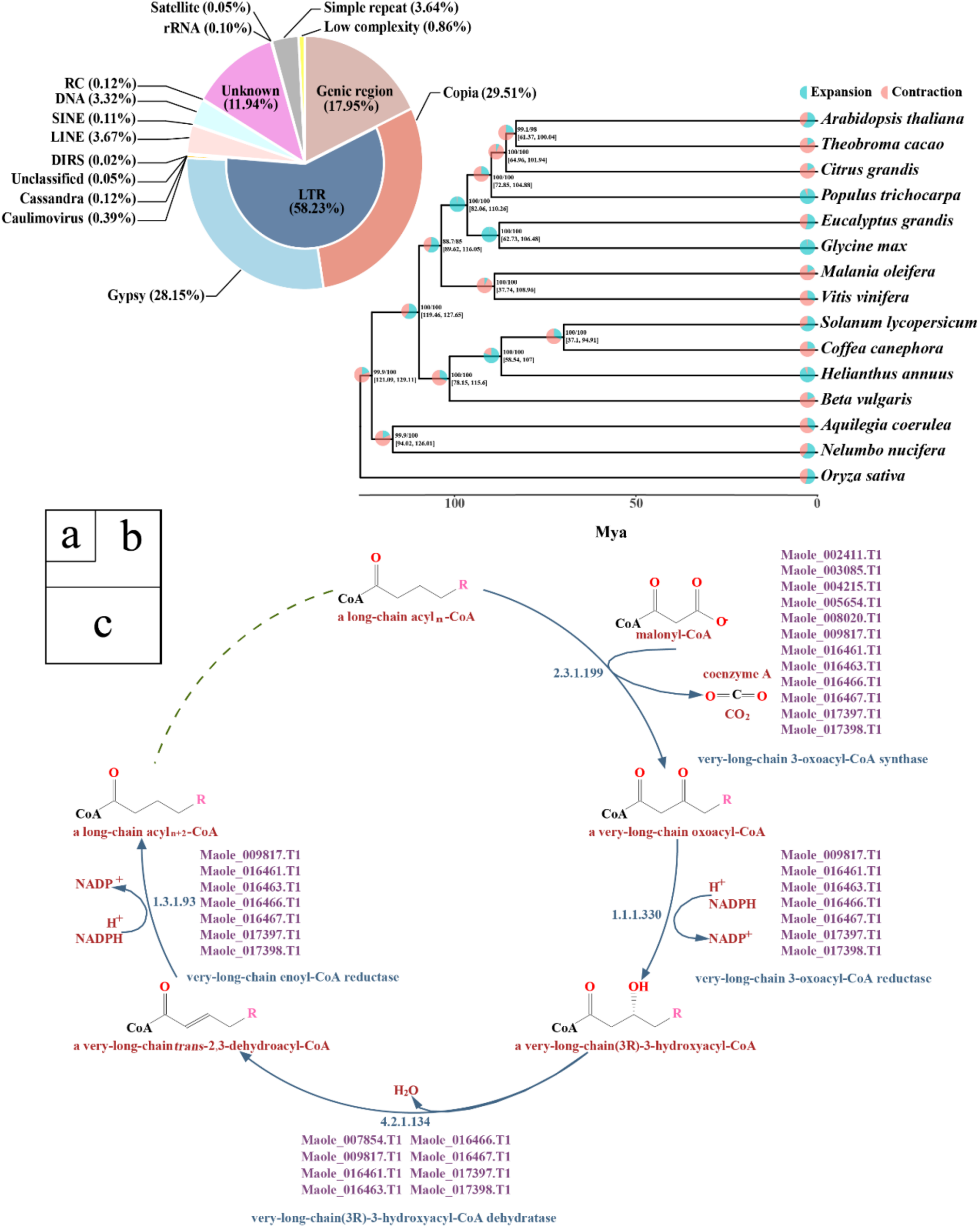
Repeat composition, phylogenomic inferences, and biosynthesis pathway for very long-chain fatty acids synthesis in *M. oleifera*. **(a)** Genome proportions of genic and various repeat sequences. **(b)** Phylogenetic tree, divergence time, and profiles of gene families that underwent expansion or contraction. Bootstrapping supports (SH-aLRT/UFBoot) are presented along with the 95% confidence intervals for each dating point in brackets. **(c)** Annotated genes involved in the biosynthesis pathway of very long-chain fatty acids (a fatty acid with minimum 22 carbon moieties) in *M. oleifera*.

*De novo* repeat identification was pursued with RepeatModeler v1.0.10 (RepeatModeler, RRID:SCR_015027) [38], which employs two complementary computational methods (RECON v1.08 and RepeatScout v1.0.5 (RepeatScout, RRID:SCR_014653) [39]) for identifying repeat element boundaries and family relationships from sequence data. Subsequently, the outputs from RepeatModeler and the RepBase library [40] were combined and used for further characterization of transposable elements (TEs), many of which are not repetitive, and other repeats by homology-based methods, including identification with RepeatMasker (v4.0.7, rmblast-2.2.28) (RepeatMasker, RRID:SCR_012954) [41]. In sum, a high percentage of the genome (82.05%) was predicted to be TEs and/or repeats in the assembled genome, predominantly (65.45%) known TEs, with 11.94% uncharacterized TEs, and a smaller number (3.64%) of simple repeats. Long terminal repeat-retrotransposons (LTR-RTs) represented the highest proportion (58.23%) of the genome, while long interspersed nuclear element (3.67%), short interspersed nuclear element (0.11%), DNA (3.32%), and rolling-circle transposon (0.12%) TEs made up a minor fraction (7.22%) of the genome. *Copia* (29.51% of the genome sequence) and *Gypsy* (28.15%) LTR-RTs were about equally abundant. Repeat annotations are provided in Fig. 2a and Supplementary Table S5.

### Transcriptome assembly and candidate gene annotation

In total, 313.36 million raw reads from RNA analyses were generated from leaf, seed, and fruit tissues and used for gene annotation (Supplementary Table S1). Illumina raw reads were processed by Trimmomatic v0.33 (Trimmomatic, RRID:SCR_011848) [42] and Cutadapt v1.13 (Cutadapt, RRID:SCR_011841) [43] and aligned to the genome assembly using HiSat2 v2.1.0 (HiSat2, RRID:SCR_015530) [44]. Base quality was assessed with FastQC (FastQC, RRID:SCR_014583) [45] before and after data cleaning. Statistics for the RNA sequencing data are shown in Supplementary Table S1. Reference genome-guided and *de novo* transcriptome assemblies, respectively, were constructed with StringTie v1.3.3b (StringTie, RRID:SCR_016323) [46] and Trinity v2.0.6 (Trinity, RRID:SCR_013048) [47]. Then, transcriptome assemblies were combined and further refined using CD-HIT v4.6 (CD-HIT, RRID:SCR_007105) [48]. Finally, 57,299 unique transcripts were predicted. The summary of transcriptome assemblies is reported in Supplementary Table S6.

For *ab initio* gene prediction, AUGUSTUS v3.2.3 (AUGUSTUS, RRID:SCR_008417) [49, 50] was employed, using model training based on coding sequences from *Arabidopsis thaliana* and 1,440 single-copy orthologs from the BUSCO embryophyta_odb9 database. For evidence-based gene prediction, the individual transcripts from RNA sequencing as well as the transcriptome assembly were aligned to the repeat-masked reference genome assembly with BlastN (BLASTN, RRID:SCR_001598) and TblastX (TBLASTX, RRID:SCR_011823) from BLAST v2.2.28+ (NCBI BLAST, RRID:SCR_004870) [51] (E-value cutoff of 10–5), respectively. Protein sequences from *A. thaliana* [52], *Vitis vinifera* [53], *Solanum lycopersicum* [54], and *Olea europaea* [55] were aligned to the TE-masked and repeat-masked reference genome assembly with BlastX (BLASTX, RRID:SCR_001653) (E-value cutoff of 10–5). After optimization with Exonerate v2.4.0 (Exonerate, RRID:SCR_016088) [56, 57], gene model predictions were finalized using the MAKER package v2.31.9 (MAKER, RRID:SCR_005309) [58] within AUGUSTUS. Annotation edit distance (AED) scores were calculated for each of the predicted genes as part of the MAKER pipeline to assess the quality of gene prediction. Putative functions for each identified gene were predicted by homology searches with BLAT (BLAT, RRID:SCR_011919) [59] against the UniProt database (UniProt, RRID:SCR_002380) [60]. Protein annotation against Pfam (Pfam, RRID:SCR_004726) [61, 62] and InterProScan (InterProScan, RRID:SCR_005829) [63] were also conducted using the scripts provided in the MAKER package. The completeness of gene annotation was checked using the BUSCO dataset (i.e., the 1,440 single-copy orthologs from the embryophyta_odb9 database) with 10^−5^ as BLAST E-value cutoff (see Supplementary File 1 for commands and settings).

A total of 24,094 genes were predicted, with average lengths of gene regions, transcript length, coding sequence, and exons, respectively, of 11,809 bp, 1,460 bp, 1,281 bp and 244 bp (Supplementary Table S7). The distribution of AED tagged by MAKER is shown in Supplementary Fig. S3, in which about 83.39% of the annotated genes (20,092 genes) had an AED <0.5 (Supplementary Table S7), indicating well-supported gene annotation. The result from BUSCO assessment of genome assembly and annotation qualities are shown in Supplementary Table S8. Identification of 92.29% of the universal single-copy genes (1,329 genes out of the total 1,440 genes) supported the high quality of the genome assembly. Among the 1,329 BUSCO conserved single-copy genes detected in the assembled genome, 1,217 (84.51% of the completed genes) were found to be single copy, while 41 genes (2.85%) were complete and duplicated (Supplementary Table S8).

The predicted genes were annotated using seven functional databases: (1) the NCBI non-redundant protein database (NR) [64], (2) the Swiss-Prot protein database [60, 65], (3) the Translated EMBL-Bank (part of the International Nucleotide Sequence Database Collaboration, TrEMBL) [60, 66], (4) the protein families database (Pfam) [67], (5) the Cluster of Orthologous Groups for eukaryotic complete genomes (KOG) database [68], (6) the KEGG (the Kyoto Encyclopedia of Genes and Genomes, Orthology) database (KEGG, RRID:SCR_012773) [69, 70], and (7) the Gene Ontology (GO) database (GO, RRID:SCR_002811) [71, 72]. By this combined strategy, 91.60% of all predicted genes could be annotated with the following protein-related database outcomes: NR (91.40%), Swiss-Prot (57.20%), TrEMBL (90.60%), Pfam (76.80%), KOG (87.60%), KEGG (32.90%), and GO (78.70%) (Supplementary Table S9).

### Differential proliferation, age dynamics, and gene proximity of different LTR-RT families

LTR-RTs (58.23% of the annotated genome) represent the most abundant group of TEs in the genome of *M. oleifera*. We further examined their classification, age distribution, birth, and death. LTRharvest [73] and LTRdigest [74] were used for *de novo* prediction of LTR-RTs. In this workflow, it was required that a candidate LTR-RT was separated by 1 to 15 Kb from other candidates and flanked by a pair of putative LTRs, which could range from 100 to 3,000 bp, but with a similarity >80%. The LTR-RT candidates that possessed complete *Gag-Pol* protein sequences were retained as intact LTR-RTs (*I*), while solo-LTRs (*S*) and truncated LTRs (*T*) were identified based on sequence similarity to the intact LTR-RTs. LTR homologies were identified by BLASTN analysis [51] with an E-value cutoff of 1e-10, 90% overlap in length, and 90% identity. Further, 3 Kb of sequence data both upstream and downstream of each detected LTR homology were extracted and compared with *Gag-Pol* protein sequences within the GyDB 2.0 database [75, 76] using TBLASTN (TBLASTN, RRID:SCR_011822). If at least 50% of any *Gag-Pol* sequence was covered by the flanking sequences with an identity >30% and an E-value cutoff of 1e-8, the corresponding LTR was excluded from the solo-LTR list. The LTR homologies that lacked any *Gag-Pol* homology in both the upstream and downstream sequences were considered to be solo-LTRs. In addition, LTRs with *Gag-Pol* sequences on one side of flanking sequences were retained as truncated LTR-RTs. The timing of LTR-RT insertion was estimated based on the divergence between the 5′-LTR and 3′-LTR of the same transposon [77]. In this procedure, each LTR pair was aligned using MUSCLE v3.8.31 (MUSCLE, RRID:SCR_011812) [78] with default settings. Kimura's two-parameter method [79] was employed with a mutation rate of 1.3e-8 substitutions year^−1^ per site to calculate approximate insertion time [80]. Superfamily classifications within the *Gypsy* and *Copia* classes are provided in Supplementary Table S10. Although the actual mode of LTR-RT activation and amplification is manifested at the family level [81], as defined by >80% sequence homology in the LTR-RTs, we focused on overall genome properties that could be more carefully assayed and compared at the LTR-RT superfamily level (>60% homology), with categories such as *Tat* and *Reina* of *Gypsy* or *Tork* and *Oryco* of *Copia*. The proliferation history of different superfamilies of *Gypsy* and *Copia* LTR-RTs are provided in Supplementary Figs. S4 and S5. The distances of intact LTR-RTs to adjacent genes were calculated, and the relationships of proximity to gene and insertion time of LTR-RTs was also examined. Gene proximity for different superfamilies of *Gypsy* and *Copia* LTR-RTs are provided in Supplementary Figs. S6 and S7 and Table S11. The relationship between gene proximity and insertion time for major LTR-RTs superfamilies are depicted in Supplementary Figs. S8 and S9.

To obtain further LTR-RT relationship insights, 5′-LTR sequences of all LTR-RTs were compared against each other with BLASTN. Two LTRs were assigned to the same cluster if they mutually covered at least 70% of their lengths with an identity of at least 60% between them. This clustering was performed using Silix v1.2.9 [82]. Solo-LTRs (*S*) and truncated LTR-RTs (*T*) were also mapped to the same cluster containing 5′ LTRs from the most similar intact LTR-RTs (*I*). Furthermore, ratios of solo-LTR-RTs and truncated LTR-RTs, respectively, to intact LTR-RTs (*S*:*I*; *T*:*I*) as well as their sums were assessed to study the removal rates of LTR-RTs over the past several million years. We further assessed the proportions of clusters with *S*:*I* values greater than 3 to evaluate LTR-RT deletions. The above-mentioned estimates remained consistent with or without shorter scaffolds, indicating that the draft genome assembly does not affect the results presented. To make an interspecific comparison, we also collected data on LTR-RT accumulation and removal rates for related plant species from a previous study [83], in which the same pipeline as ours was used for LTR-RTs analysis. Results of the interspecific comparison are provided in Supplementary Fig. S10 and Table S12.

A few categories of LTR-RTs were highly abundant within the *M. oleifera* genome. Twenty-six annotated clades and one unclassified clade of *Gypsy* LTR-RTs, as well as 17 annotated clades of *Copia*, were identified by querying the GyDB 2.0 database with full-length LTR-RTs of *M. oleifera*. Significant differences in their individual counts, average length, and genomic representation were found for superfamilies with both *Gypsy* and *Copia* classes of LTR-RTs (Supplementary Table S10). *Del* is the most prevalent clade of *Gypsy* in the *M. oleifera* genome, representing 6.99% of the assembled genome. *Sire* and *Tork* are the two most abundant clades of *Copia*, representing 3.77% and 1.16% of the assembled genome, respectively. More considerable variation in average sequence length was observed for clades of *Gypsy* (4,848–11,592 bp) compared to those of *Copia*, (4,823–9,473 bp). In sum, for most clades of both *Gypsy* and *Copia* LTR-TRs, few recent amplification were identified, while a single peak of ancient amplification 2–10 million years ago (Mya) was observed. Exceptionally, *Galadriel* and *Tat* superfamilies of *Gypsy* showed an active recent amplification less than 1 Mya (Supplementary Figs. S4 and S5). We observed some LTR-RTs overlapping genes for most of the subgroups of *Gypsy* and *Copia*, especially for the prevalent clades. About 1,500 from the *Del* clade of *Gypsy* were found to overlap with genes; >200 from Galadriel overlapped, and also hundreds from *Sire*, *Tork*, *Oryco*, and *Retrofit* of *Copia* overlapped (Supplementary Fig. S6 , Fig. S7, and Table S11). Except for the ones overlapping with genes, LTR-RTs were mostly distributed in regions characterized by 3–5 Kb distance to genes. In addition, we found that gene-overlapping LTR-RTs had been generated over an extended period of time, as revealed by the insertion dates for the most representative sub-groups of *Gypsy* (Supplementary Fig. S8) and *Copia* (Supplementary Fig. S9).

When comparing *M. oleifera* to other related plant species with respect to LTR-RT accumulation and removal rates, we found that the *M. oleifera* genome is characterized by the largest numbers of intact, solo, and truncated LTR-RTs. Moreover, the *M. oleifera* genome has experienced relatively low removal rates (*S*:*I* = 2.28, (*S*+*T*)/*I* = 2.61) as evidenced by the lowest proportion of LTR clusters with *S*:*I* > 3 (Supplementary Fig. S10 and Table S12). Target site duplications (TSDs), usually 5 bp of identical sequence for LTR-RTs, are the direct repeats that occur at the insertion sites of most TEs. TSDs were detected for all (24,660) intact LTR-RTs. However, they were found for only 510 (<0.1% of 56,170) solo-LTRs, indicating that these elements called "solo-LTRs" in our analysis are mostly truncated LTR-RT rather than the products of unequal homologous recombination. As expected, very few (251 out of 8,196, or about 0.3%) of the truncated LTR-RTs had TSDs. Regardless of whether an LTR-RT has been converted into a solo-LTR or a truncated LTR-RT, this still represents decay of a formerly intact LTR-RT into a non-functional (i.e., immobile) status that will eventually be fully removed by the deletions associated with illegitimate recombination [80]. Given the abundance of LTR-RTs and their proximity to genes, it will be interesting to further explore their potential influence on genome evolution and gene expression.

### Orthologous genes, whole genome duplication, and phylogenetic inference

OrthoMCL v2.0.9 (Ortholog Groups of Protein Sequences, RRID:SCR_007839) [84] was used to identify orthologous and paralogous gene clusters in the assembled genomes of *M. oleifera* and 14 related plant species (Supplementary Table S13), namely, *Arabidopsis thaliana* [85], *Theobroma cacao* [86], *Citrus grandis* [87], *Populus trichocarpa* [88], *Eucalyptus grandis* [89, 90], *Glycine max* [91], *Vitis vinifera* [92, 93], *Solanum lycopersicum* [54], *Coffea canephora* [94], *Helianthus annuus* [95], *Beta vugaris* [96], *Nelumbo nucifera* [97], *Aquilegia coerulea* [98], and *Oryza sativa* [99]. Recommended settings were used for all-against-all BLASTP comparisons (Blast+ v2.3.056) [51] and OrthoMCL analyses. OrthoMCL analyses identified 30,367 gene families (414,518 genes involved in these analyses) based on effective database sizes of all vs all BLASTP with an E-value of 10^–5^ and a Markov chain clustering default inflation parameter.

The amino acid sequences of 282 orthologous protein-coding single-copy genes (Supplementary File 2), identified by OrthoMCL among the 15 analyzed genomes, were acquired and aligned with MUSCLE v3.8.31 [78], employing default settings (see Supplementary File 1 for commands and settings). The concatenated amino acid sequences (Supplementary File 3) were trimmed using trimAI v1.2 (trimal -gt 0.8 -st 0.001 -cons 60) [100] and were further used for sequence evolution model selection with ModelFinder [101]. JTT+F+R5 was selected as the best model based on all employed criteria (Akaike information criterion [AIC], corrected AIC, and Bayesian information criterion). To construct the maximum likelihood phylogenetic tree (see Supplementary File 1 for commands and settings), IQ-TREE v1.6.7 [102] was run with the selected optimal sequence evolution model (-m JTT+F+R5) and with ultrafast bootstrapping (-bb 1000) [103, 104], and employing the Shimodaira-Hasegawa-like approximate likelihood-ratio test (SH-aLRT, -alrt 1000) [105].

Phylogenetic dating (see Supplementary File 1 for commands and settings) was done with the MCMCTree program of PAML v4.9h [106] with the following parameters: ‘burnin 100000, sampfreq 200, nsample 10000’. Rice (*O. sativa*) was defined as outgroup. The dating was calibrated against the recently summated timing of divergence [107]: the divergence of rice from other plant genomes at 113–128.63 Mya (refers to MRCA [most recent common ancestor], Monocotyledoneae: Acorales—[Dioscoreales + [Liliales + [Asparagales + Aracales + Poales]]], 113–128.63 Mya), divergence of *N. nucifera* and *A. coerulea* from other dicots at 119.6–128.6 Mya (refers to MRCA, Eudicotyledoneae: Ranunculales—[Vitales + Rosids + [Caryophyllales + Asterids]], 119.6–128.63 Mya), and divergence of *C. canephora*, *S. lycopersicum*, and *H. annuus* to the lineage formed by *A. thaliana*, *V. vinifera*, and other related plants at 85.8–128.63 Mya (refers to MRCA, Vitales—[Rosids + [Caryophyllales + Asterids]], 85.8–128.63 Mya; MRCA, Rosids (minus Vitales)—[Caryophyllales + Asterids], 85. 128.63 Mya; MRCA, Caryophyllales—Asterids, 85.8–128.63 Mya). The Molecular Clock test as implemented in MEGA X [108] rejected the null hypothesis that all tips of the tree are equidistant from the root of the tree.

All branches of the reconstructed phylogenetic tree gained high support from both Shimodaira-Hasegawa-like approximate likelihood-ratio and the ultrafast bootstrapping tests with SH-aLRT >88% and UFBoot >85%, respectively (Fig. 2b). The phylogenetic analysis identified the closest relationship of *M. oleifera* (Santalales) to grape (*V. vinifera*, Vitales), with the divergence time between *M. oleifera* and grape estimated at ∼88.9798 Mya with 95% confidence intervals of 37.7394–108.955 Mya. *Nelumbo nucifera* (Proteales) and *Aquilegia coerulea* (Ranunculales) were forming a sister clade to all other Eudicots. The phylogenetic relationship among Ranunculales, Proteales, Santalales, and Vitales is unresolved in the most recent phylogeny of the angiosperms (APG IV) [109] ([110], accessed at 22 October 2018) [111].

Amino acid sequences of intra-specific in-paralogs constructed by OrthoMCL analyses were aligned with MUSCLE v3.8.31 [112] employing default settings. *Ks* (the number of synonymous substitutions per synonymous site) was calculated with KaKs_Calculator v2.0 [113] under a YN model, after the conversion of protein sequence alignments into the corresponding codon alignments with PAL2NAL v14 [114]. The *Ks* distribution suggests that the *M. oleifera* genome has not undergone any recent or lineage-specific whole-genome duplication (Supplementary Fig. S11). This finding is also supported by the low number of intra-specific collinear blocks called with MCScanX (Supplementary Fig. S12) [115].

Of the identified OrthoMCL gene families, 6,509 gene families (194,824 genes) were shared among all of the genomes analyzed. A total of 520 gene families (2,097 genes) were found to be specific to the assembled *M. oleifera* genome when compared with the other 14 genomes (Supplementary Table S14). Using CAFE v4.0 [95, 116], 309 gene families were detected that have expanded, while 1,528 gene families were found to have contracted in the *M. oleifera* lineage (Fig. 2b). Hypergeometric tests were performed to determine if specific functional categories of KEGG or GO were significantly overrepresented in the families that were significantly expanded or contracted within the *M. oleifera* genome. The expanded gene families were enriched for >100 significant (*q* < 0.05) GO-terms of three different functional categories (biological process, cellular component, and molecular function) (Supplementary Table S15) and seven KEGG pathways (Supplementary Table S16). Three enriched categories were related to hormone signal transduction and to biosynthesis of tyrosine, isoquinoline alkaloid, cutin and wax, terpenoid, pantothenate and CoA, and glycine. The contracted gene families were enriched for >400 GO-terms (Supplementary Table S17) and 11 KEGG pathways (Supplementary Table S18) related to various aspects of secondary metabolism, at *q* <0.05. Results from functional enrichment analysis of rapidly evolving genes are summarized in Supplementary Table S19 (for GO enrichment) and Supplementary Table S20 (for KEGG enrichment).

### Metabolic gene clusters and candidate genes for fatty acid biosynthesis pathways

It is evident that genes for numerous plant secondary metabolic pathways are sometimes densely clustered in a specific genomic region, generating biosynthetic gene clusters (BGCs) [117–119]. With the newly released and robust computational toolkit, plantiSMASH [120], 23 such BGCs related to various secondary metabolic pathways were detected (Supplementary Table S21 and Supplementary File 4), such as saccharide- (10 gene clusters), terpene- (4), alkaloid- (2), polyketide- (1), and lignan-polyketide (1)-related. An additional five putative BGCs were identified that could not be assigned to specific secondary metabolic pathways. The identified BGCs spanned 258 to 1,282 Kb and contained three to eight core protein domains related to secondary metabolism.

Given the importance of fatty acid production in *M. oleifera*, we further annotated genes within the fatty acid biosynthesis pathway by querying the Plant Metabolic Network (PMN v12.5, RRID:SCR_003778) [121, 122], after enzymatic annotations for coding genes through the E2P2 package v3.1 [123]. The initial (*de novo*) fatty acid biosynthesis process mainly occurs in plastids [124] of leaf mesophyll cells, seeds, and oil-accumulating fruits in plants. In this process, acetyl and malonyl groups are condensed and further elongated to give rise to the production of 16:0-ACPs (palmitic acids, acyl carrier protein) and 18:0-ACPs (stearic acid and oleic acids). After this initial process, very long-chain fatty acids (VLCFAs, with 22 or more carbons) can be synthesized at the endoplasmic reticulum by sequential addition of C2 moieties from malonyl-CoA to form C18 acyl groups [125].

We detected 14 genes that are predicted to function in the four reactions of the elongation cycle, including the condensation of long-chain acyl-CoA and malonyl-CoA to form 3-oxoacyl-CoA, the reduction to 3-hydroxyacyl-CoA, the dehydration to (2E)-alkan-2-enoyl-CoA, and the final reduction to an elongated fatty acyl-CoA [125]. We detected 19 candidate genes potentially functioning in the reactions of the initial process (Supplementary Fig. S13) and 14 genes in the subsequent VLCFA biosynthesis pathway (Fig. 2c). Interestingly, we found the genes of the VLCFA pathway forming two gene clusters of local duplicates, one composed of four genes (Maole_016461, Maole_016463, Maole_016466, and Maole_016467) and the other of two genes (Maole_017397 and Maole_017398). These six genes occurring in localized clusters are all predicted to be involved in the four key reactions of the chain elongation cycle, suggesting an important effect of local gene duplication on efficient VLCFA production. By comparison, only a few cases (one including Maole_003221.T1 and Maole_003222.T1, the other including Maole_008716.T1 and Maole_008717.T1) of localized gene duplication were found for the initial fatty acid biosynthesis pathway.

## Conclusions

In sum, we provide a high-quality *de novo* genome assembly and in-depth characterization for *M. oleifera*, combining PacBio single-molecule long reads and 10× Genomics linked reads. The excellent quality of the genome assembly is supported by both the 92.29% BUSCO analysis-based single-copy gene coverage and the 99.32% (PacBio long reads), 98.5% (10× Genomics linked reads), and 91%–98% (Illumina RNA sequencing reads) mapping rates of the genome and transcriptome reads. Of note, the significantly low heterozygosity of the sequenced genome was a key factor for the high continuity in genome assembly of *M. oleifera* obtained in this study. This low level of heterozygosity also suggests a high level of inbreeding in the wild population of trees that was the source of genomic DNA used for genome analysis. The novel genomic resources generated in the present study provide a vital foundation for further studies on the genetics of metabolite biogenesis, the genetic basis of the vulnerable status, the significance of local gene duplications in genomes without a recent whole genome duplication, and for biotechnology aiming at an efficient exploration of valuable plant compounds. The pattern of birth-death dynamics and gene proximity of LTR-RTs, revealed here, provide a basis for future LTR-RTs studies in plants. It will be particularly interesting to investigate whether the observed slow rate of LTR-RT amplification and removal are related to the long-lived perennial lifestyle of this largely undomesticated tree species. As the only whole genome and the second genome released for the Olacaceae family and in the Santalales order, the present data resource is also of critical value for phylogenomic and comparative genomic studies.

## Supplementary Material

GIGA-D-18-00301_Original_Submission.pdf

GIGA-D-18-00301_Revision_1.pdf

Response_to_Reviewer_Comments_Original_Submission.pdf

Reviewer_1_Report_Original_Submission -- Catherine Jane Nock, Ph.D 9/3/2018 Reviewed

Reviewer_2_Report_Original_Submission -- Stephen Tsui 9/12/2018 Reviewed

Reviewer_2_Report_Revision_1 -- Stephen Tsui 12/3/2018 Reviewed

Supplemental Files

## Data Availability

The genome assembly, annotations, and other supporting data are available via the *Giga*Science database *Giga*DB [126]. The raw sequence data have been deposited in the Short Read Archive under NCBI BioProject ID PRJNA472200. All commands and parameter settings for genome assembly, quality assessment of genome assembly, transcriptome assembly from RNA sequencing, repeat and gene annotation, ortholog identification, phylogenetic reconstruction, and dating have been uploaded to protocols.io [127].

## Additional files

**Fig. S1:** Length distribution of PacBio subreads.

**Fig. S2:** *K*-mer frequency distribution estimated from PacBio sequences after filtering and correction at *k*-mer size of 17. A *k*-mer refers to an artificial sequence division of K nucleotides. From *k*-mer frequencies, genomic characteristics (genome size, repeat structure and heterozygous rate) could be estimated. Peaks at depth of 21 are annotated with dashed lines.

**Fig. S3:** Distribution of AED (annotation edit distance) scores from gene prediction. AED = 0 indicates perfect agreement between the gene prediction and the transcript and protein evidence; AED = 1 indicates no evidence support for annotation.

**Fig. S4:** Proliferation history of different superfamilies of the *Gypsy* class of LTR-RTs (long terminal repeat-retrotransposons) in the *M. oleifera* genome.

**Fig. S5:** Proliferation history of different superfamilies of the *Copia* class of LTR-RTs in the *M. oleifera* genome.

**Fig. S6:** Gene proximity for different superfamilies of the *Gypsy* class of LTR-RTs in the *M. oleifera* genome. The natural logarithm of the base distance between an LTR-RT and an adjacent gene (plus one) was used as the X axis.

**Fig. S7:** Gene proximity for different superfamilies of the *Copia* class of LTR-RTs in the *M. oleifera* genome. The natural logarithm of the base distance between an LTR-RT and an adjacent gene (plus one) was used as the X axis.

**Fig. S8:** Gene proximity and insertion time for major superfamilies of the *Gypsy* class of LTR-RTs in the *M. oleifera* genome. The natural logarithm of the base distance between an LTR-RT and an adjacent gene (plus one) was used as the Y axis, time in mya as X axis.

**Fig. S9:** Gene proximity and insertion time for major superfamilies of the *Copia* class of LTR-RTs in the *M. oleifera* genome. The natural logarithm of the base distance between an LTR-RT and an adjacent gene (plus one) was used as the Y axis, time in mya as X axis.

**Fig. S10:** Birth and death of LTR-RTs (long terminal repeat-retrotransposons) in the *M. oleifera* genome compared to six other members of Rosids and two members from Asterids. (a) total numbers of intact LTR-RTs in the genome; (b) comparison of *S* + *T* values among these nine plant species; (c) total numbers of intact LTR-RTs and traces of LTR-RT death; (d) ratios of solo-LTR to intact LTR-RT (*S*:*I*); (e) proportions of LTR-RTs found in the clusters with high removal rates (filtered *S*:*I* ≥ 3). *S*, number of solo-LTRs; *T*, number of truncated LTR-RTs; *I*, number of intact LTR-RTs.

**Fig. S11:** *Ks* distribution of paralogs in synteny blocks within the *M. oleifera* genome.

**Fig. S12:** Gene: synteny-block pattern in the *M. oleifera* genome.

**Fig. S13:** Genes annotation for the initial (*de novo*) fatty acid biosynthesis process in the *M. oleifera* genome.

**Table S1:** Summary of PacBio and Illumina sequencing data (10× Genomics and RNA sequencing) generated in the present study. IDs of the study, sample, library and accessions in NCBI SRA and employed sequencing platform, material origins of the sequenced DNA or RNA, statistics of raw and cleaned data, and mapping rates are shown.

**Table S2:** Data summary from 10× Genomics analysis based on GemCode index multiplicity. Read subsets are based on the number of associated reads for each index. For raw reads, all indices (including those with N's) are included in the count. For all other read sets, only the indices without N's were used for binning.

**Table S3:** Estimation of genome characteristics based on 17-mer statistics.

**Table S4:** Statistics of the different versions of *M. oleifera* genome assembly in ascending order. N50: shortest sequence length at 50% of the genome; L50: smallest number of contigs whose length sum produces N50. NA: data not available; * statistics for contigs/scaffolds. Gene completeness was generated by assessment with 1440 single copy orthologs from the BUSCO embryophyta_odb9 database.

**Table S5:** Summary of the annotated TEs in the genome assembly for *M. oleifera*. LTR: Long Terminal Repeat retrotransposons; LINE: Long Interspersed Nuclear Element, a category of non-LTR (long terminal repeat) retroelements; SINE: Short Interspersed Nuclear Element, a category of non-autonomous and non-coding retroelements (TEs); RC: Rolling-circle transposons.

**Table S6:** Summary of transcriptome assemblies using three different analysis pipelines.

**Table S7:** Summary of annotated genes. AED: Annotation Edit Distance; gene region (including 5′, 3’ UTRs, exons and introns).

**Table S8:** Summary of BUSCO evaluation for gene prediction.

**Table S9:** Summary of functional annotation of predicted genes.

**Table S10:** Superfamilies within the *Gypsy* and *Copia* LTR-RTs classes of TEs.

**Table S11:** Gene proximity of superfamilies of *Gypsy* and *Copia* classes of LTR-RTs.

**Table S12:** Comparison of the number of original and filtered intact LTR-RT, solo-LTR and Truncated LTR TEs among 9 plant species.

**Table S13:** Genomic data used for phylogenomic and gene family analyses. Origins, download links, assembly versions, genome properties and references of 14 genomes are shown.

**Table S14:** Summary of gene family analyses. Unique groups and genes, single-copy and duplicated groups and genes are summarized for the 15 analyzed plant genomes.

**Table S15:** GO enrichment of expanded gene families. (A) ‘Category’ is the Gene Ontology (GO) term ID; (B) ‘P_value’ is the overrepresentation *P*-value indicating the observed frequency of a given term among analyzed genes is equal to the expected frequency based on the null distribution; i.e., lower *P*-values indicate stronger evidence for overrepresentation; (C) ‘Q_value’ is the Benjamini and Hochberg adjusted *P*-value, (D) ‘numEPInCat’ is the number of expanded gene families in the corresponding GO category; (E) ‘numInCat’ is the number of detected gene families in the corresponding GO category; (F) ‘Term’ is the GO term; (G) ‘Ontology’ indicates which ontology the term comes from. Significant at *q* < 0.05.

**Table S16:** KEGG enrichment of expanded gene families. (A) ‘KO category’ is the KEGG Orthology (KO) category ID; (B) ‘P_value’ is the over represented *P*-value indicating the observed frequency of a given term among analyzed genes is equal to the expected frequency based on the null distribution; i.e., lower *P*-values indicate stronger evidence for overrepresentation; (C) ‘Q_value’ is the Benjamini and Hochberg adjusted *P*-value, (D) ‘numEPInCat’ is the number of expanded gene families in the corresponding KO category; (E) ‘numInCat’ is the number of detected gene families in the corresponding KO category; (F) ‘Pathway’ is the KEGG pathway; (G) ‘Class’ indicates which KEGG class the pathway comes from. Significant at *q* < 0.05.

**Table S17:** GO enrichment of contracted gene families. (A) ‘Category’ is the Gene Ontology (GO) term ID; (B) ‘P_value’ is the over represented *P*-value indicating the observed frequency of a given term among analyzed genes is equal to the expected frequency based on the null distribution; i.e., lower *P*-values indicate stronger evidence for overrepresentation; (C) ‘Q_value’ is the Benjamini and Hochberg adjusted *P*-value, (D) ‘numEPInCat’ is the number of expanded gene families in the corresponding GO category; (E) ‘numInCat’ is the number of detected gene families in the corresponding GO category; (F) ‘Term’ is the GO term; (G) ‘Ontology’ indicates which ontology the term comes from. Significant at *q* < 0.05.

**Table S18:** KEGG enrichment of contracted gene families. (A) ‘KO category’ is the KEGG Orthology (KO) category ID; (B) ‘P_value’ is the over represented *P*-value indicating the observed frequency of a given term among analyzed genes is equal to the expected frequency based on the null distribution; i.e., lower *P*-values indicate stronger evidence for overrepresentation; (C) ‘Q_value’ is the Benjamini and Hochberg adjusted *P*-value, (D) ‘numEPInCat’ is the number of expanded gene families in the corresponding KO category; (E) ‘numInCat’ is the number of detected gene families in the corresponding KO category; (F) ‘Pathway’ is the KEGG pathway; (G) ‘Class’ indicates which KEGG class the pathway comes from. Significant at *q* < 0.05.

**Table S19:** GO enrichment of fast evolving gene families. (A) ‘Category’ is the Gene Ontology (GO) term ID; (B) ‘P_value’ is the over represented *P*-value indicating the observed frequency of a given term among analyzed genes is equal to the expected frequency based on the null distribution; i.e., lower *P*-values indicate stronger evidence for overrepresentation; (C) ‘Q_value’ is the Benjamini and Hochberg adjusted *P*-value, (D) ‘numEPInCat’ is the number of expanded gene families in the corresponding GO category; (E) ‘numInCat’ is the number of detected gene families in the corresponding GO category; (F) ‘Term’ is the GO term; (G) ‘Ontology’ indicates which ontology the term comes from. Significant at *q* < 0.05.

**Table S20:** KEGG enrichment of fast evolving gene families. (A) ‘KO category’ is the KEGG Orthology (KO) category ID; (B) ‘P_value’ is the over represented *P*-value indicating the observed frequency of a given term among analyzed genes is equal to the expected frequency based on the null distribution; i.e., lower *P*-values indicate stronger evidence for overrepresentation; (C) ‘Q_value’ is the Benjamini and Hochberg adjusted *P*-value, (D) ‘numEPInCat’ is the number of expanded gene families in the corresponding KO category; (E) ‘numInCat’ is the number of detected gene families in the corresponding KO category; (F) ‘Pathway’ is the KEGG pathway; (G) ‘Class’ indicates which KEGG class the pathway comes from. Significant at *q* < 0.05.

**Table S21:** Summary of 23 metabolic gene clusters in the *M. oleifera* genome. Genomic coordinates, gene composition, core protein domains related to metabolism and pathway assignments are shown.

**Supplementary File 1:** The commands and parameter settings for all steps in genome assembly, quality assessment of the genome assembly, transcriptome assembly from RNA-seq data, repeat and gene annotation, ortholog identification and phylogenetic reconstruction and dating.

**Supplementary File 2:** Gene names/codes for the 282 orthologous protein-encoding single-copy genes used in the phylogenetic analyses.

**Supplementary File 3:** Concatenated alignment of amino acid sequences used in the phylogenetic analyses.

**Supplementary File 4:** Visualization of each metabolic gene cluster detected within the *M. oleifera* genome.

## Abbreviations

ACP: acyl carrier protein; AED: annotation edit distance; AIC: Akaike information criterion; BGC: biosynthetic gene cluster; BUSCO: Benchmarking Universal Single-Copy Orthologs; GEM: gel beads in emulsion; GO: Gene Ontology; KO: KEGG Orthology; KEGG: Kyoto Encyclopedia of Genes and Genomes; KOG: Cluster of Orthologous Groups for eukaryotic complete genomes; LTR-RT: long terminal repeat-retrotransposon; MRCA: most recent common ancestor; Mya: million years ago; NCBI: National Center for Biotechnology information; NR: NCBI non-redundant protein database; PacBio: Pacific Biosciences; PCR: polymerase chain reaction; PE: paired end; Pfam: protein families; TE: transposable element; TrEMBL: Translated EMBL-Bank; TSD: target site duplication; VLCFA: very long-chain fatty acid.
