# Supplementary material for: Genome sequence of Malania oleifera, a tree with great value for nervonic acid production
Source: Gigascience. 2019 Jan 24;8(2):giy164. doi: 10.1093/gigascience/giy164 (PMC6377399; doi:10.1093/gigascience/giy164)
Supplement: GIGA-D-18-00301_Original_Submission.pdf [file giy164_giga-d-18-00301_original_submission.pdf]

GigaScience

Genome sequence of Malania oleifera, an endangered tree with great value for nervonic acid production

--Manuscript Draft--

**Manuscript Number:**	GIGA-D-18-00301
**Full Title:**	Genome sequence of Malania oleifera, an endangered tree with great value for nervonic acid production
**Article Type:**	Data Note
**Funding Information:**	Fundamental Research Funds for the Central Universities (YX2013-41) — Mr. Jian-Feng Mao<br>the construction of the workstation for Academician Bennetzen (2015AC018) — Mr. Fu Chen<br>Yunnan Provincial Science and Technology Department (CN) (2015BB018) — Mr. Sihai Wang<br>the State Key Laboratory of Phytochemistry and Plant Resources in West China (P2015-KF11) — Mr. Sihai Wang
**Abstract:**	Background: Malania oleifera Chun et S. Lee, a member of the Olacaceae family, is an IUCN Red Listed tree, endemic and restricted to the Karst region of southwest China. This tree's seed is valued for its high content of precious fatty acids (especially nervonic acid). However, studies on its genetic make-up, and fatty acid biogenesis are severely hampered by a lack of molecular and genetic tools.<br>Findings: We generated 51 Gigabases (Gb) and 135 Gb of raw DNA sequences, using PacBio Single-Molecule Real-Time (SMRT) and 10x Genomics sequencing, respectively. A final genome assembly, with a scaffold N50 size of 4.65 Megabases (Mb) and a total length of 1.51 Gb, was obtained by primary assembly based on PacBio long reads plus scaffolding with 10x Genomics reads. Identified repeats constituted ~82% of the genome, and 24,064 protein-coding genes were predicted with high support. The genome has low heterozygosity and shows no evidence for recent whole genome duplication. Metabolic pathway genes relating to the accumulation of long chain fatty acid were identified and studied in detail.<br>Conclusions: Here, we provide the first genome assembly and gene annotation for M. oleifera. The availability of these resources will be of great importance for conservation biology, and for the functional genomics of nervonic acid biosynthesis.
**Corresponding Author:**	Jian-Feng Mao, Ph.D.<br>Beijing Forestry University<br>Beijing, CHINA
**Corresponding Author Secondary Information:**	
**Corresponding Author's Institution:**	Beijing Forestry University
**Corresponding Author's Secondary Institution:**	
**First Author:**	Chao-Qun Xu
**First Author Secondary Information:**	
**Order of Authors:**	Chao-Qun Xu
	Hui Liu
	Shan-Shan Zhou
	Dongxu Zhang
	Wei Zhao, Ph.D.

Powered by Editorial Manager® and ProduXion Manager® from Aries Systems Corporation

	Sihai Wang
	Fu Chen
	Yan-Qiang Sun
	Shuai Nie
	Kai-Hua Jia
	Si-Qian Jiao
	Ren-Gang Zhang
	Quan-Zheng Yun
	Wenbin Guan, Ph.D.
	Xuewen Wang, Ph.D.
	Jeffrey L. Bennetzen, Ph.D.
	Fatemeh Maghuly, Ph.D.
	Ilga Porth, Ph.D.
	Yves Van de Peer, Ph.D.
	Xiao-Ru Wang, Ph.D.
	Yongpeng Ma, Ph.D.
	Jian-Feng Mao, Ph.D.
**Order of Authors Secondary Information:**	
**Additional Information:**	
**Question**	**Response**
Are you submitting this manuscript to a special series or article collection?	No
**Experimental design and statistics**<br><br>Full details of the experimental design and statistical methods used should be given in the Methods section, as detailed in our Minimum Standards Reporting Checklist. Information essential to interpreting the data presented should be made available in the figure legends.<br><br>Have you included all the information requested in your manuscript?	Yes
**Resources**<br><br>A description of all resources used, including antibodies, cell lines, animals and software tools, with enough information to allow them to be uniquely identified, should be included in the	Yes

Methods section. Authors are strongly encouraged to cite Research Resource Identifiers (RRIDs) for antibodies, model organisms and tools, where possible.<br><br>Have you included the information requested as detailed in our Minimum Standards Reporting Checklist?	
**Availability of data and materials**<br><br>All datasets and code on which the conclusions of the paper rely must be either included in your submission or deposited in publicly available repositories (where available and ethically appropriate), referencing such data using a unique identifier in the references and in the "Availability of Data and Materials" section of your manuscript.<br><br>Have you have met the above requirement as detailed in our Minimum Standards Reporting Checklist?	Yes

Manuscript

Click here to download Manuscript Malania_genome_mao8_2_xu5_notrack.docx

Genome sequence of *Malania oleifera*, an endangered tree with great value for nervonic acid production

Chao-Qun Xu[1‡], Hui Liu[1‡], Shan-Shan Zhou[1‡], Dong-Xu Zhang[2], Wei Zhao[1], Sihai Wang[3], Fu Chen[4], Yan-Qiang Sun[1], Shuai Nie[1], Kai-Hua Jia[1], Si-Qian Jiao[1], Ren-Gang Zhang[5], Quan-Zheng Yun[5], Wenbin Guan[1], Xuewen Wang[4,6], Jeffrey L. Bennetzen[4,6], Fatemeh Maghuly[7], Ilga Porth[8,9,10], Yves Van de Peer[11,12,13], Xiao-Ru Wang[1,14], Yongpeng Ma[15*], Jian-Feng Mao[1*]

[1] Beijing Advanced Innovation Center for Tree Breeding by Molecular Design, National Engineering Laboratory for Tree Breeding, School of Nature Conservation, College of Biological Sciences and Technology, Beijing Forestry University, Beijing, 100083, China.

[2] College of Life Science, Datong University, Datong, 037009, Shanxi, China.

[3] Yunnan Key Laboratory of Forest Plant Cultivation and Utilization, State Forestry Administration Key Laboratory of Yunnan Rare and Endangered Species Conservation and Propagation, Yunnan Academy of Forestry, Kunming, 650201, Yunnan, China.

[4] The Camellia Institute, Yunnan Academy of Forestry, Guangnan, 663300, Yunnan, China.

[5] Beijing Ori-Gene Science and Technology Co. Ltd, Beijing, 102206, China.

[6] Department of Genetics, University of Georgia, Athens, GA 30602, USA

[7] Plant Biotechnology Unit (PBU), Dept. Biotechnology, BOKU-VIBT, University of Natural Resources and Life Sciences, Muthgasse 18, 1190 Vienna, Austria.

[8] Département des sciences du bois et de la forêt, 1030, Avenue de la Médecine, Université Laval, Québec (Québec) G1V 0A6, Canada.

[9] Institute for System and Integrated Biology, Pavillon Charles-Eugène-Marchand, 1030, Avenue de la Médecine, Université Laval, Québec (Québec) G1V 0A6, Canada.

[10] Centre d'Étude de la Forêt, 1030, Avenue de la Médecine, Université Laval, Québec (Québec) G1V 0A6, Canada.

[11] Department of Plant Biotechnology and Bioinformatics, Ghent University, 9052 Ghent, Belgium

[12] VIB Center for Plant Systems Biology, 9052 Ghent, Belgium

[13] Department of Biochemistry, Genetics and Microbiology Genetics, University of Pretoria, Private bag X20, Pretoria 0028, South Africa

[14] Department of Ecology and Environmental Science, UPSC, Umeå University, SE-901 87 Umeå, Sweden.

[15] Yunnan Key Laboratory for Integrative Conservation of Plant Species with Extremely Small Population, Kunming Institute of Botany, Chinese Academy of Sciences, Kunming, 650201, China.

‡These authors contributed equally to this paper.

*Correspondence to: mayongpeng@mail.kib.ac.cn (YPM); jianfeng.mao@bjfu.edu.cn (JFM)

Abstract

Background: *Malania oleifera* Chun et S. Lee, a member of the Olacaceae family, is an IUCN Red Listed tree, endemic and restricted to the Karst region of southwest China. This tree's seed is valued for its high content of precious fatty acids (especially nervonic acid). However, studies on its genetic make-up, and fatty acid biogenesis are severely hampered by a lack of molecular and genetic tools.

Findings: We generated 51 Gigabases (Gb) and 135 Gb of raw DNA sequences, using PacBio Single-Molecule Real-Time (SMRT) and 10x Genomics sequencing, respectively. A final genome assembly, with a scaffold N50 size of 4.65 Megabases (Mb) and a total length of 1.51 Gb, was obtained by primary assembly based on PacBio long reads plus scaffolding with 10x Genomics reads. Identified repeats constituted ~82% of the genome, and 24,064 protein-coding genes were predicted with high support. The genome has low heterozygosity and shows no evidence for recent whole genome duplication. Metabolic pathway genes relating to the accumulation of long chain fatty acid were identified and studied in detail.

Conclusions: Here, we provide the first genome assembly and gene annotation for *M. oleifera*. The availability of these resources will be of great importance for conservation biology, and for the functional genomics of nervonic acid biosynthesis.

Keywords: *de novo* genome assembly, endangered plant, *Malania*, nervonic acid, transcriptome

DATA DESCRIPTION

Background information

Malania oleifera Chun et S. Lee, a 10-20 m high tree (**Fig. 1a-d**), is from the monotypic genus *Malania* of the Olacaceae family [1]. This tree is endemic to a restricted area within the Karst topography of southwest Guangxi and southeast Yunnan provinces, China. The recorded distribution range is bounded by N23°23′- N24°28′ in latitude and E105°30′- E107°30′ in longitude (**Fig. 1e**). This tree is called “garlic-fruit tree” or “suantouguo” (蒜头果) by local communities, due to its garlic shaped fruits. As an endemic tree and because of its natural populations being much reduced because of ongoing logging and habitat clearance, this tree species has been listed in the IUCN Red List as “Vulnerable B1+2c” (extent of occurrence estimated to be < 20,000 km^2 and a continuing decline, observed, projected, or inferred, in numbers of mature individuals) [2], and has been assigned as a plant species with an extremely small population size (PSESP) for urgent conservation action [3]. Different mechanisms that could explain why *M. oleifera* became an endangered species have been proposed, such as niche specialization [4], limited germination and regeneration [5, 6], or pollination/mating system [7], as well as the biology of its pathogens [8]. However, until now, apart from a recent chloroplast genome sequence [9], only a few molecular genetic resources are available for *M. oleifera* to investigate its population structure and genetic makeup.

Besides conservation urgency, *M. oleifera* is also notable for its substantial phytochemical and phytopharmaceutical value: its seed has very high (64.5%) oil

content [10, 11], and the highest-known proportion (55.70-67%) of nervonic acid ($C_{24}H_{46}O_2$, PubChem CID: 5281120). Nervonic acid, an important component of the central and peripheral nerve system, is typically localized in sphingomyelin of animal cell membranes [12], where it enhances brain function and, in healthy individuals, prevents obesity-related health risks [13] that can otherwise occur due to demyelination in patients with a genetic defect [14]. *M. oleifera* produces essential oils with benzyl alcohol (58.42%) and benzaldehyde (29.66%) as the main constituents as well as benzoic acid (1.49%) [10]. *M. oleifera* seeds also produce the glycoprotein *malania* which has high cytotoxic activity towards tumor cells and is one of the most potent toxins of plant origin [15]. Yet, little is known about the molecular mechanisms underlying the metabolic biosynthesis processes of these promising compounds in *M. oleifera*.

Here, we present a high-quality genome assembly for *M. oleifera*, combining PacBio single molecule long-reads and 10x Genomics linked reads. The assembled genome, its structural and functional annotation and in-depth characterization will provide valuable tools for the genomic dissection of the species' endangering mechanism for future conservation purposes, as well as for in-depth molecular knowledge regarding biosynthesis and regulation of metabolism to promote the efficient and sustainable exploitation of this precious biological resource.

Plant material

One mature and healthy tree with abundant fruit (**Fig. 1 a, b, c, d**) was chosen as a tissue source for whole genome sequencing. The selected tree measured ~18 m in height, ~35 cm in diameter (at breast height) and is believed to be ~50 years old. This tree is located within a natural stand close to Diji Village, Jiumoxiang, Guangnan County, Yunnan Province, China (N23.90^{o} latitude, E104.90^{o} longitude, 1,402 m elevation) (**Fig. 1e**). The stand, from which the samples were taken, experienced little anthropogenic intervention and consists of trees of the same species but of different ages. Fresh leaves were sampled in September of 2017.

For RNA sequencing, leaves, fruits and seeds were sampled from healthy, high-yielding, mature trees from Funing County, Yunnan province and Leye County, Guangxi province, China, in different seasons during the years 2013-2016 (**Fig. 1e** and **Table S1**). Samples were immediately flash frozen in liquid nitrogen upon collection and transported on dry ice to Beijing Forestry University (BFU) for sequence analysis.

All samples were collected with permission from and under the supervision of local forestry bureaus. See **Table S1** and **Fig. 1** for more details.

PacBio SMRT sequencing

High-quality and high-molecular-weight genomic DNA was extracted from leaves of the selected tree, following the "~20 kb SMRTbell™ Libraries" protocol [16]. DNA was purified using the Mobio PowerClean® Pro DNA Clean-Up Kit, and its quality was assessed by standard agarose gel electrophoresis and Thermo Fisher Scientific

Qubit Fluorometry. Genomic DNA was sheared to a size range of 15-50 kb using either AMPure beads (Beckman Coulte) or g-TUBE (Covaris), and enzymatically repaired and converted into SMRTbell template libraries according to Pacific Biosciences instructions. Following this procedure, hairpin adapters were ligated after exonuclease-based digestion (of the remaining damaged DNA fragments and those fragments without adapters at both ends). The resulting SMRTbell templates were subsequently size-selected by Blue Pippin electrophoresis (Sage Sciences). Templates ranging from 15 to 50 kb were sequenced on a PacBio Sequel instrument using S/P2-C2 sequencing chemistry (10 SMRT cells). A total of 5,778,035 PacBio long reads were generated, yielding a total of 51,149,552,938 bases (roughly 30x coverage of the assembled genome) of single-molecule sequencing data with an average read length of 8,852 bases (**Fig. S1** and **Table S1**).

10x Genomics library preparation and Illumina sequencing

Purified high-molecular-weight genomic DNA of high quality was incubated with Proteinase K and RNaseA for 30 min at 25 °C. DNA was further purified, indexed and partitioned into barcoded libraries that were prepared using the GemCode kit (10x Genomics, Pleasanton, CA). Following the GemCode procedure, 1.0 ng of DNA was used for GEM (Gel Beads in Emulsion) reactions in which DNA fragments were partitioned into molecular reactors to extend the DNA and to introduce specific 14-bp partition barcodes. Subsequently, GEM reactions were PCR-amplified. The PCR cycling protocol was: 95 °C for 5 min; cycled 18x: 4 °C for 30 s, 45 °C for 1 s, 70 °C

for 20 s, and 98 °C for 30 s; held at 4 °C. The PCR products were purified as described in the GemCode protocol. Purified DNA was sheared, end-repaired, adenylation tailed, universal adapter ligated and samples indexed according to the manufacturer's recommendations.

The whole genome GemCode library was sequenced using 2x150 paired-end (PE) sequencing on Illumina HiSeq X Ten. A total of 899.778 million reads (~134.97 Gb, roughly 89x coverage of the assembled genome) were obtained, of which 89.1% had base quality values over 20 and 80% over 30 (**Table S1**). There were 19,319,151 (99.98% of total read pairs) indexes assigned to more than one read pair, while 27,368 (9.55%), 830 (2.12%) and 450 (1.80%) had more than 1000, 3000, or 5000 read pairs, respectively (**Table S2**). Sequence data were analyzed using the GemCode Long Ranger Software Suite [17, 18].

RNA sequencing

Frozen tissues were ground with a mortar and pestle, and RNA was isolated using the NEBNext Poly (A) mRNA Magnetic Isolation Module. RNA quality was determined on an Agilent 2100 BioAnalyzer. Seven sequencing libraries were prepared using the NEBNext Ultra RNA Library Prep Kit for Illumina. 150/100 bp PE sequencing was performed on an Illumina HiSeq 2000/2500 machine. See **Table S1** for details.

Estimation of genome size, heterozygosity, and repeat content

Canu v1.6 [19] was employed to filter and correct the PacBio reads. Next, k-mers were

counted using Jellyfish [20]. Finally, gce v1.0.0 [21] was used to estimate genome size, repeat content and the level of heterozygosity. A total of 29,971,959,192 k-mers were identified, and the peak k-mer depth obtained was 21 (**Fig. S2**). Genome size was estimated to be ~1.50 Gb (**Table S3**). The final cleaned data corresponded to about 21-fold coverage. Repeat and error frequencies were estimated to be 54.61% and 0.34%, respectively. Finally, a very low level of heterozygosity was observed, ~0.06%.

De novo genome assembly and quality control

First, primary assemblies (eight from PacBio long reads, one from 10x Genomics linked reads) were prepared by different pipelines. Next, scaffolding and polishing were performed on the optimal primary assemblies in order to obtain a final genome assembly. Primary assembly v0.1 was generated from PacBio long reads after correction by Canu v1.6 [19], assembly v0.2 by MECAT v1.1 [22], assembly v0.3 by miniasm v0.2-r168 [23] after alignment by minimap v0.2-r124 [23], assembly v0.4 by Falcon v0.7 [24, 25] after correction with Canu v1.6, assembly v0.5 by SMARTdenovo v1.0.0 [26] after correction with Canu v1.6, assembly v0.6 by Wtdbg v1.2.8 [27] after correction with Canu v1.6, assembly v0.7 by SMARTdenovo v1.0.0 after correction, and assembly v0.8 by Wtdbg v1.2.8. Assembly v0.9 was prepared by Supernova™ assembler 2.0 [28, 29] from 10x Genomics linked reads data. Based on quality control parameters, assembly v0.7 was chosen as optimal for further scaffolding and polishing. It generated a reasonably-sized assembly (1.51 Gb), providing the highest N50 (i.e. the shortest sequence length at 50% of the total genome assembly length) (1.12 Mb), and

the lowest number of contigs (3,038) and L50 (i.e. the smallest number of contig sequences whose lengths sum produces the N50 value) (330). Furthermore, v0.7 exhibited the longest contig length (6.72 Mb), as well as 71.80% gene completeness as determined by BUSCO assessment (**Table S4**). This assembly (v0.7) was further polished with raw PacBio long reads using arrow v2.2.1 [30] to produce (in two rounds) assembly v1.0. Subsequently, 10x Genomics linked reads were processed with Long Ranger [17, 18], and were then aligned to v1.0 using BWA mem v0.7.15 (default values, -*t*12) [31] and subsequently scaffolded by ARCS v1.0.1 [32] to produce assembly v1.1. The final assembly was generated after one further iteration of polishing with arrow v2.21 and three iterations with Pilon v1.22 [33]. Before arrow-based polishing, PacBio raw reads were aligned using BLASR v5.1 [34, 35], and PacBio raw reads were mapped with bowtie2 v2.2.6 [36] before each iteration with Pilon. In the final assembly, a genome size of 1.51 Gb was obtained, consisting of 2,987 contigs, 1,277 scaffolds (with contig N50 of 1.22 Mb, scaffold N50 of 4.65 Mb, longest contig of 6.7 Mb and longest scaffold of 25.1 Mb), and has a gene completeness of 90.60% (**Table 1** and **Table S4**). The consistency of the predicted genome size based on k-mer characterization and the assembled genome indicated a good quality for our assembly. Furthermore, when all clean Illumina reads were mapped to the final assembly (v1.2f), a high sequence coverage of 98.5% was obtained. In addition, an even higher sequence coverage of 99.32% was observed for mapping PacBio long reads to the final assembly using BLASR. These two coverage values suggested high sequence completeness and fidelity of the genome assembly. Mapping rates (91-98%) were also very high for

transcriptomic datasets mapped to the final assembly, of which most (79-96%) were uniquely mapped (**Table S1**), with the exception of one RNA sequencing library (SRA accession: SRR7221534) that yielded low mapping rates (10.31%), a result that we cannot explain by anything aside from microbial or other contamination.

Transposable element and other repeat annotation

De novo repeat identification was pursued with RepeatModeler v1.0.10 [37], which employs two complementary computational methods (RECON v1.08 and RepeatScout v1.0.5) for identifying repeat element boundaries and family relationships from sequence data. Subsequently, the outputs from RepeatModeler and the RepBase library [38] were combined and used for further characterization of transposable elements (TEs), many of which are not repetitive, and other repeats by homology-based methods, including identification with RepeatMasker (v4.0.7, rmblast-2.2.28) [39]. In sum, a high percentage of the genome (82.05%) was predicted to be TEs and/or repeats in the assembled genome, predominantly (65.45%) known TEs, with 11.94% uncharacterized TEs, and a smaller number (3.64%) of simple repeats. Long terminal repeat-retrotransposons (LTR-RTs) represented the highest proportion (58.23%) of the genome, while LINE (3.67%), SINE (0.11%), DNA (3.32%) and RC (0.12%) TEs made up a minor fraction (7.22%) of the genome. *Copia* (29.51% of the genome sequence) and *Gypsy* (28.15%) LTR-RTs were about equally abundant. Repeat annotations are provided in **Fig.2a** and **Table S5**.

Transcriptome assembly and candidate gene annotation

In total, 313.36 million raw reads from RNA analyses were generated from leaf, seed, and fruit tissues and used for gene annotation (**Table S1**). Illumina raw reads were processed by Trimmomatic v0.33 [40] and Cutadapt v1.13 [41] and aligned to the genome assembly using HiSat2 v2.1.0 [42]. Base quality was assessed with FastQC [43] before and after data cleaning. Statistics for the RNA sequencing data are shown in **Table S1**. Reference genome-guided and *de novo* transcriptome assemblies, respectively, were constructed with StringTie v1.3.3b [44] and Trinity v2.0.6 [45]. Then, transcriptome assemblies were combined and further refined using CD-HIT v4.6 [46]. Finally, 57,299 unique transcripts were predicted. The summary of transcriptome assemblies is reported in **Table S6**.

For *ab initio* gene prediction, AUGUSTUS v3.2.3 [47, 48] was employed, using model training based on coding sequences from *Arabidopsis thaliana* and 1,440 single copy orthologs from the BUSCO embryophyta_odb9 database. For evidence-based gene prediction, the individual transcripts from RNA sequencing as well as the transcriptome assembly were aligned to the repeat-masked reference genome assembly with BlastN and TblastX from BLAST v2.2.28+ [49] (E-value cutoff of 10^{-5}), respectively. Protein sequences from *A. thaliana* [50], *Vitis vinifera* [51], *Solanum lycopersicum* [52] and *Olea europaea* [53] were aligned to the TE-masked and repeat-masked reference genome assembly with BlastX (E-value cutoff of 10^{-5}). After optimization with Exonerate v2.4.0 [54, 55], gene model predictions were finalized using the MAKER package v2.31.9 [56] within AUGUSTUS. AED (Annotation Edit

Distance) scores were calculated for each of the predicted genes as part of the MAKER pipeline to assess the quality of gene prediction. Putative functions for each identified gene were predicted by homology searches with BLAT [57] against the UniProt database [58]. Protein annotation against Pfam [59, 60] and InterProScan [61] were also conducted using the scripts provided in the MAKER package. The completeness of gene annotation was checked using the BUSCO dataset (i.e. the 1,440 single-copy orthologs from the embryophyta_odb9 database) with 10^{-5} as BLAST E-value cutoff.

A total of 24,094 genes were predicted, with average lengths of gene regions, genes (including 5', 3' UTRs, exons and introns), CDS and exons, respectively, of 11,809 bp, 1,460 bp, 1,281 bp and 244 bp (**Table S7**). The distribution of AED tagged by MAKER is shown in **Fig. S3**, in which about 83.39% of the annotated genes (20,092 genes) had an AED < 0.5 (**Table S7**), indicating well-supported gene annotation. The result from BUSCO assessment of genome assembly and annotation qualities are shown in **Table S8**. Identification of 92.29% of the universal single-copy genes (1,329 genes out of the total 1,440 genes) supported the high quality of the genome assembly. Among the 1,329 BUSCO conserved single-copy genes detected in the assembled genome, 1,217 (84.51% of the completed genes) were found to be single-copy, while 41 genes (2.85%) were complete and duplicated (**Table S8**).

The predicted genes were annotated using seven functional databases: (1) the NCBI non-redundant protein database (NR) [62], (2) the Swiss-Prot protein database [58, 63], (3) the Translated EMBL-Bank (part of the International Nucleotide Sequence Database Collaboration, TrEMBL) [58, 64], (4) the protein families database (Pfam) [65], (5) the

Cluster of Orthologous Groups for eukaryotic complete genomes (KOG) database [66], (6) the KO (the Kyoto Encyclopedia of Genes and Genomes, Orthology) database [67, 68], and (7) the Gene Ontology (GO) database [69, 70]. By this combined strategy, 91.60% of all predicted genes could be annotated with the following protein related database outcomes: NR (57.20%), Swiss-Prot (90.60%), TrEMBL (91.40%), Pfam (76.80%), KOG (87.60%), KO (32.90%), and GO (78.70%) (**Table S9**).

Differential proliferation, age dynamics and gene proximity of different LTR-RT families

LTR-RTs (58.23% of the annotated genome) represent the most abundant group of TEs in the genome of *M. oleifera*. We further examined their classification, age distribution, birth and death. LTRharvest [71] and LTRdigest [72] were used for *de novo* prediction of LTR-RTs. In this workflow, it was required that a candidate LTR-RT was separated by 1 to 15 kb from other candidates and flanked by a pair of putative LTRs, which could range from 100 to 3,000 bp, but with a similarity >80%. The LTR-RT candidates that possessed complete Gag-Pol protein sequences were retained as intact LTR-RTs (*I*), while solo-LTRs (*S*) and truncated LTRs (*T*), were identified based on sequence similarity to the intact LTR-RTs. LTR homologies were identified by BLASTN analysis [49] with an E-value cutoff of 1e-10, 90% overlap in length and 90% identity. Further, 3 kb of sequence data both upstream and downstream of each detected LTR homology were extracted and compared with Gag-Pol protein sequences within the GyDB 2.0 database [73, 74] using TBLASTN. If at least 50% of any Gag-Pol sequence was

covered by the flanking sequences with an identity > 30% and an E-value cutoff of 1e-8, the corresponding LTR was excluded from the solo-LTR list. The LTR homologies that lacked any Gag-Pol homology in both the upstream and downstream sequences were considered to be solo-LTRs. In addition, LTRs with Gag-Pol sequences on one side of flanking sequences were retained as truncated LTR-RTs. The timing of LTR-RT insertion was estimated based on the divergence between the 5′ -LTR and 3′ -LTR of the same transposon [75]. In this procedure, each LTR pair was aligned using MUSCLE v3.8.31 [76] with default settings. Kimura's two-parameter method [77] was employed with a mutation rate of 1.3e-8 substitutions yr^{-1} per site to calculate approximate insertion time [78]. Superfamily classifications within the *Gypsy* and *Copia* classes are provided in **Table S10**. Although the actual mode of LTR-RT activation and amplification is manifested at the family level [79], as defined by >80% sequence homology in the LTR-RTs, we focused on overall genome properties that could be more carefully assayed and compared at the LTR-RT superfamily level (>60% homology), with categories such as Tat and Reina of *Gypsy* or Tork and Oryco of *Copia*. The proliferation history of different superfamilies of *Gypsy* and *Copia* LTR-RTs are provided in **Figs. S4** and **S5**. The distances of intact LTR-RTs to adjacent genes were calculated, and the relationships of proximity to gene and insertion time of LTR-RTs was also examined. Gene proximity for different superfamilies of *Gypsy* and *Copia* LTR-RTs are provided in **Figs. S6** and **S7, and Table S11**. The relationship between gene proximity and insertion time for major LTR-RTs superfamilies are depicted in **Figs. S8** and **S9**.

To obtain further LTR-RT relationship insights, 5′-LTR sequences of all LTR-RTs were compared against each other with BLASTN. Two LTRs were assigned to the same cluster if they mutually covered at least 70% of their lengths with an identity of at least 60% between them. This clustering was performed using Silix v1.2.9 [80]. Solo-LTRs (*S*) and truncated LTR-RTs (*T*) were also mapped to the same cluster containing 5' LTRs from the most similar intact LTR-RTs (*I*). Furthermore, ratios of solo-LTR-RTs and truncated LTR-RTs, respectively, to intact LTR-RTs (*S*:*I*; *T*:*I*) as well as their sums were assessed to study the removal rates of LTR - RTs over the past several million years. We further assessed the proportions of clusters with *S*:*I* values greater than three to evaluate LTR-RT deletions. The abovementioned estimates remained consistent with or without shorter scaffolds, indicating that the draft genome assembly does not affect the results presented. To make an interspecific comparison, we also collected data on LTR-RT accumulation and removal rates for related plant species from a previous study [81], in which the same pipeline as ours was used for LTR-RTs analysis. Results of the interspecific comparison are provided in **Fig S10** and **Table S12**.

A few categories of LTR-RTs were highly abundant within the *M. oleifera* genome. Twenty-six annotated clades and one unclassified clade of *Gypsy* LTR-RTs, as well as 17 annotated clades of *Copia* were identified by querying the GyDB 2.0 database with full-length LTR-RTs of *M. oleifera*. Significant differences in their individual counts, average length, and genomic representation were found for superfamilies with both *Gypsy* and *Copia* classes of LTR-RTs (**Table S10**). Del is the most prevalent clade of *Gypsy* in the *M. oleifera* genome, representing 6.99% of the assembled genome. Sire

and Tork are the two most abundant clades of *Copia*, representing 3.77% and 1.16% of the assembled genome, respectively. More considerable variation in average sequence length was observed for clades of *Gypsy* (4,848 - 11,592 bp) compared to those of *Copia* (4,823 - 9,473 bp). In sum, for most clades of both *Gypsy* and *Copia* LTR-TRs, few recent amplification were identified while a single peak of ancient amplification 2-10 million years ago (mya) were observed. Exceptionally, Galadriel and Tat superfamilies of *Gypsy* showed an active recent amplification less than one mya (**Fig. S4** and **S5**). We observed some LTR-RTs overlapping genes for most of the subgroups of *Gypsy* and *Copia*, especially for the prevalent clades: about 1,500 from the Del clade of *Gypsy* were found to overlap with genes; > 200 from Galadriel overlapped, and also hundreds from Sire, Tork, Oryco and Retrofit of *Copia* overlapped (**Fig. S6, Fig. S7** and **Table S11**). Except for the ones overlapping with genes, LTR-RTs were mostly distributed in regions characterized by 3-5 kb distance to genes. In addition, we found that gene-overlapping LTR-RTs had been generated over an extended period of time, as revealed by the insertion dates for the most representative sub-groups of *Gypsy* (**Fig. S8**) and *Copia* (**Fig. S9**).

When comparing *M. oleifera* to other related plant species with respect to LTR-RTs accumulation and removal rates, we found that the *M. oleifera* genome is characterized by the largest numbers of intact, solo- and truncated LTR-RTs. Moreover, the *M. oleifera* genome has experienced relatively low removal rates ($S{:}I$ = 2.28, $(S+T)/I$ = 2.61) as evidenced by the lowest proportion of LTR clusters with $S{:}I > 3$ (**Fig. S10** and **Table S12**). Target site duplications (TSDs), usually 5 bp of identical sequence for LTR-

RTs, are the direct repeats that occur at the insertion sites of most TEs. TSDs were detected for all (24,660) intact LTR-RTs. However, they were found for only 510 (<0.1% of 56,170) solo-LTRs, indicating that these elements called “solo-LTRs” in our analysis are mostly truncated LTR-RT rather than the products of unequal homologous recombination. As expected, very few (251 out of 8,196, or about 0.3%) of the truncated LTR-RTs had TSDs. Regardless of whether an LTR-RT has been converted into a solo-LTR or a truncated LTR-RT, this still represents decay of a formerly intact LTR-RT into a non-functional (i.e., immobile) status that will eventually be fully removed by the deletions associated with illegitimate recombination [78]. Given the abundance of LTR-RTs and their proximity to genes, it will be interesting to further explore their potential influence on genome evolution and gene expression.

Orthologous genes, whole genome duplication and phylogenetic inference

OrthoMCL v2.0.9 [82] was used to identify orthologous and paralogous gene clusters in the assembled genomes of *M. oleifera* and 14 related plant species (**Table S13**), namely *Arabidopsis thaliana* [83], *Theobroma cacao* [84], *Citrus grandis* [85], *Populus trichocarpa* [86], *Eucalyptus grandis* [87, 88], *Glycine max* [89], *Vitis vinifera* [90, 91], *Solanum lycopersicum* [52], *Coffea canephora* [92], *Helianthus annuus* [93], *Beta vugaris* [94], *Nelumbo nucifera* [95], *Aquilegia coerulea* [96] and *Oryza sativa* [97]. Recommended settings were used for all-against-all BLASTP comparisons (Blast+ v2.3.056) [49] and OrthoMCL analyses. OrthoMCL analyses identified 30,367 gene families (414,518 genes involved in these analyses) based on effective database sizes

of all versus all BLASTP with an E-value of 10^{-5} and a Markov Chain Clustering default inflation parameter.

A total of 282 orthologous protein-encoding genes among the 15 analyzed genomes were acquired and aligned with MUSCLE v3.8.31 [98] employing default settings. The concatenated amino acid sequences were used to generate a maximum likelihood phylogenetic tree with PhyML v3.0 [99] based on a GTR+G+I model. The divergence time was estimated with r8s v1.81 [100] and calibrated against the timing of divergence between *A. thaliana* and *P. trichocarpa* lineages (~90 mya) [101] as well as against the divergence time between *A. thaliana* and *V. vinifera* lineages (~124 mya) [102]. The phylogenetic analysis identified the closest relationship of *M. oleifera* to grape (*V. vinifera*), with their lineage divergence time estimated at ~112 mya (**Fig. 2b**) [103]. Amino acid sequences of intra-specific in-paralogs were aligned with MUSCLE v3.8.31 [98] employing default settings. *Ks* (the number of synonymous substitutions per synonymous site) was calculated with KaKs_Calculator v2.0 [104] under a YN model, after the conversion of protein sequence alignments into the corresponding codon alignments with PAL2NAL v14 [105]. The *Ks* distribution indicated that the *M. oleifera* genome is not derived from any recent or lineage-specific whole-genome duplication (**Fig. S11**). This conclusion is also supported by the low number of intra-specific collinear blocks called with MCScanX (**Fig. S12**) [103].

Of the identified OrthoMCL gene families, 6,509 gene families (194,824 genes) were shared among all of the genomes analyzed. A total of 520 gene families (2,097 genes) were found to be specific to the assembled *M. oleifera* genome when compared

with the other 14 genomes (**Table S14**). Using CAFE v4.0 [93, 106], 309 gene families were detected that have expanded, while 1,528 gene families were found to have contracted in the *M. oleifera* lineage (**Fig. 2b**). Hypergeometric tests were performed to determine if specific functional categories of KEGG or GO were significantly overrepresented in the families that were significantly expanded or contracted within the *M. oleifera* genome. The expanded gene families were enriched for > 100 significant ($q < 0.05$) GO-terms of three different functional categories (Biological Process (BP), Cellular Component (CC), and Molecular Function (MF)) (**Table S15**) and seven KEGG pathways (**Table S16**). Three enriched categories were related to hormone signal transduction and to biosynthesis of tyrosine, isoquinoline alkaloid, cutin and wax, terpenoid, pantothenate and CoA, and glycine. The contracted gene families were enriched for > 400 GO-terms (**Table S17**) and 11 KEGG pathways (**Table S18**) related to various aspects of secondary metabolism, at $q < 0.05$. Results from functional enrichment analysis of rapidly evolving genes are summarized in **Table S19** (for GO enrichment) and **Table S20** (for KEGG enrichment).

Metabolic gene clusters and candidate genes for fatty acid biosynthesis pathways

It is evident that genes for numerous plant secondary metabolic pathways are sometimes densely clustered in a specific genomic region, generating biosynthetic gene clusters (BGCs) [107-109]. With the newly released and robust computational toolkit, plantiSMASH [110], 23 such BGCs related to various secondary metabolic pathways were detected (**Table S21** and **Supplementary File 1**), such as saccharide- (10 gene

clusters), terpene- (4), alkaloid- (2), polyketide- (1), and lignan-polyketide (1)-related. An additional five putative BGCs were identified that could not be assigned to specific secondary metabolic pathways. The identified BGCs spanned 258 to 1,282 kb and contained 3-8 core protein domains related to secondary metabolism.

Given the importance of fatty acid production in *M. oleifera*, we further annotated genes within the fatty acid biosynthesis pathway by querying the Plant Metabolic Network (PMN v12.5 [111, 112], after enzymatic annotations for coding genes through the E2P2 package v3.1 [113]. The initial (*de novo*) fatty acid biosynthesis process mainly occurs in plastids [114] of leaf mesophyll cells, seeds, and oil-accumulating fruits in plants. In this process, acetyl and malonyl groups are condensed and further elongated to give rise to the production of 16:0-ACPs (palmitic acid) and 18:0-ACPs (stearic acid and oleic acids). After this initial process, very long chain fatty acids (VLCFAs, with 22 or more carbons) can be synthesized at the endoplasmic reticulum by sequential addition of C2 moieties from malonyl-CoA to form C18 acyl groups [115].

We detected a total of 14 genes that are predicted to function in the four reactions of the elongation cycle, including the condensation of long-chain acyl-CoA and malonyl-CoA to form 3-oxoacyl-CoA, the reduction to 3-hydroxyacyl-CoA, the dehydration to (2E)-alkan-2-enoyl-CoA, and the final reduction to an elongated fatty acyl-CoA [115]. We detected 19 candidate genes potentially functioning in the reactions of the initial process (**Fig. S13**), and 14 genes in the subsequent VLCFA biosynthesis pathway (**Fig. 2c**). Interestingly, we found the genes of the VLCFA pathway forming two gene clusters of local duplicates, one composed of 4 genes (Maole_016461,

Maole_016463, Maole_016466, and Maole_016467) and the other of two genes (Maole_017397 and Maole_017398). These six genes occurring in localized clusters are all predicted to be involved in the four key reactions of the chain elongation cycle, suggesting an important effect of local gene duplication on efficient VLCFA production. By comparison, only a few cases (one including Maole_003221.T1 and Maole_003222.T1, the other including Maole_008716.T1 and Maole_008717.T1) of localized gene duplication were found for the initial fatty acid biosynthesis pathway.

Conclusions

In sum, we provide a high quality *de novo* genome assembly, and in-depth characterization for *M. oleifera*, combining PacBio single molecule long-reads and 10x Genomics linked reads. The excellent quality of the genome assembly is supported by both the 92.29% BUSCO analysis-based single-copy gene coverage and the 99.32% (PacBio long reads), 98.5% (10x Genomics linked reads) and 91-98% (Illumina RNA sequencing reads) mapping rates of the genome and transcriptome reads. Of note, the significantly low heterozygosity of the sequenced genome was a key factor for the high continuity in genome assembly of *M. oleifera* obtained in this study. This low level of heterozygosity also suggests a high level of inbreeding in the wild population of trees that was the source of genomic DNA used for genome analysis. The novel genomic resources generated in the present study provide vital foundation for further studies on the genetics of metabolite biogenesis, the genetic basis of the trees endangered status, the significance of local gene duplications in genomes without a recent whole genome

duplication, and for biotechnology aiming at an efficient exploration of valuable plant compounds. The pattern of birth-death dynamics and gene proximity of LTR-RTs, revealed here, provide a basis for future LTR-RTs studies in plants. It will be particularly interesting to investigate whether the observed slow rate of LTR-RT amplification and removal are related to the long-lived perennial lifestyle of this largely undomesticated tree species. As the only whole genome and the second genome released for the Olacaceae family and in the Santalales order, the present data resource is also of critical value for phylogenomic and comparative genomic studies.

Availability of supporting data

The genome assembly, annotations, and other supporting data will be available via the *Giga*Science database *Giga*DB. The raw sequence data have been deposited in the Short Read Archive (SRA) under NCBI BioProject ID PRJNA472200.

Abbreviations

bp: base pair; kb: kilobases; Mb: megabases; Gb: gigabases; TE: transposable element; BUSCO: benchmarking universal single-copy orthologs; CDS: coding sequence; mya: million years ago.

Acknowledgments

This study was funded by Fundamental Research Funds for the Central Universities (No. YX2013-41), by the construction of the workstation for Academician Bennetzen

(No. 2015AC018), by the Science Fund of China's Yunnan government (No. 2015BB018), and by the State Key Laboratory of Phytochemistry and Plant Resources in West China (No. P2015-KF11).

Author Contributions

JFM, YM and JLB conceived and designed the study; CQX, HL, SSZ, ZW, SQJ, SW, FC, YQS, SN, KHJ, DZ, RGZ, WG and QZY prepared the materials, conducted the experiments and analyzed all data; JFM, CQX and YM wrote the manuscript; XW, FM, IP, YVP, JLB and XRW were involved in data interpretation and finalizing the manuscript draft. All authors read and approved the final draft.

Conflict of Interest

The authors declare that they have no competing financial interests.

References

1. Wu Z, Raven P and Hong D. Flora of China. Vol. 5 (Ulmaceae through Basellaceae). Science Press, Beijing, and Missouri Botanical Garden Press, St. Louis, 2003.
2. Sun W: *Malania oleifera*. The IUCN Red List of Threatened Species 1998: e.T32361A9701100. http://dx.doi.org/10.2305/IUCN.UK.1998.RLTS.T32361A9701100.en. Accessed 08 July 2018.
3. Ma Y, Chen G, Edward Grumbine R, Dao Z, Sun W and Guo H. Conserving plant species with extremely small populations (PSESP) in China. Biodiversity and Conservation. 2013;22 3:803-9. doi:10.1007/s10531-013-0434-3.
4. Xie WD, Chen JH, Lai JY, Shi HM, Huang KX, Liu JB, et al. Analysis on relationship between geographic distribution of *Malania oleifera* and hydro-thermal factors. Journal of Tropical & Subtropical Botany. 2009.
5. Xie WD, Chen JH, Lai JY, Shi HM, Lin SF, Liu B, et al. Life-table Analysis of *Malania oleifera*, A Rare and Endangered Plant. Journal of Central South University of Forestry & Technology. 2009;29 2:73-6.
6. Wu Y, Li X and Hu Y. Reproductive biology of *Malania oeifera*. Acta Scientiarum Naturalium Universitatis Sunyatseni. 2004;43 2:81-3.
7. Lai JY, Shi HM, Pan CL, Chen SW, Yan zuan YE, Ming LI, et al. Pollination biology of rare and endangered species *Malania oleifera* Chun et Lee. Journal of Beijing Forestry University. 2008.
8. Xiong Y, Hong L, Li H and Li X. Bionomics of the pathogens of *Malania oleifera* seed rot. Forest Pest & Disease. 2003;22:1-4.
9. Liu S-S, Hu Y-H, Maghuly F, Porth IM and Mao J-F. The complete chloroplast genome sequence annotation for *Malania oleifera*, a critically endangered and important bioresource tree. Conservation Genetics Resources. 2018; doi:10.1007/s12686-018-1005-4.
10. Tang T-F, Liu X-M, Ling M, Lai F, Zhang L, Zhou Y-H, et al. Constituents of the essential oil and fatty acid from *Malania oleifera*. Industrial Crops and Products. 2013;43:1-5. doi:https://doi.org/10.1016/j.indcrop.2012.07.003.
11. Ma B-L, Liang S-F, Zhao D-Y, Ai-Xia XU and Zhang K-J. Study on plants containing nervonic acid. Acta Botanica Boreali-occidentalia Sinica. 2004;24 12:2362-5.
12. Sandhir R, Khan M, Chahal A and Singh I. Localization of nervonic acid beta-oxidation in human and rodent peroxisomes: impaired oxidation in Zellweger syndrome and X-linked adrenoleukodystrophy. Journal of Lipid Research. 1998;39 11:2161-71.
13. Oda E, Hatada K, Kimura J, Aizawa Y, Thanikachalam PV and Watanabe K. Relationships between serum unsaturated fatty acids and coronary risk factors: negative relations between nervonic acid and obesity-related risk factors. International Heart Journal. 2005;46 6:975-85.
14. Sargent JR, Coupland K and Wilson R. Nervonic acid and demyelinating

disease. Medical Hypotheses. 1994;42 4:237-42.

15. Yuan Y, Dai X, Wang D and Zeng X. Purification, characterization and cytotoxicity of malanin, a novel plant toxin from the seeds of *Malania oleifera*. Toxicon. 2009;54 2:121-7. doi:https://doi.org/10.1016/j.toxicon.2009.03.024.

16. Preparing *Arabidopsis* Genomic DNA for Size-Selected ~20 kb SMRTbell™ Libraries. http://www.pacb.com/wp-content/uploads/2015/09/Shared-Protocol-Preparing-Arabidopsis-DNA-for-20-kb-SMRTbell-Libraries.pdf. Accessed 20 Sept 2017.

17. Zheng GXY, Lau BT, Schnall-Levin M, Jarosz M, Bell JM, Hindson CM, et al. Haplotyping germline and cancer genomes with high-throughput linked-read sequencing. Nature Biotechnology. 2016;34:303. doi:10.1038/nbt.3432.

18. An open-source release of Long Ranger 2.2.0. https://github.com/10xGenomics/longranger. Accessed 01 Dec 2017.

19. Koren S, Walenz BP, Berlin K, Miller JR, Bergman NH and Phillippy AM. Canu: scalable and accurate long-read assembly via adaptive k-mer weighting and repeat separation. Genome Research. 2017;27 5:722-36.

20. Marcais G and Kingsford C. A fast, lock-free approach for efficient parallel counting of occurrences of k-mers. Bioinformatics. 2011;27 6:764-70. doi:10.1093/bioinformatics/btr011.

21. Liu B, Shi Y, Yuan J, Hu X, Zhang H, Li N, et al. Estimation of genomic characteristics by analyzing k-mer frequency in *de novo* genome projects. arXiv preprint arXiv:13082012. 2013.

22. Xiao C-L, Chen Y, Xie S-Q, Chen K-N, Wang Y, Han Y, et al. MECAT: fast mapping, error correction, and *de novo* assembly for single-molecule sequencing reads. Nature Methods. 2017;14:1072. doi:10.1038/nmeth.4432.

23. Li H. Minimap and miniasm: fast mapping and de novo assembly for noisy long sequences. Bioinformatics. 2016;32 14:2103-10. doi:10.1093/bioinformatics/btw152.

24. Chin CS, Peluso P, Sedlazeck FJ, Nattestad M, Concepcion GT, Clum A, et al. Phased diploid genome assembly with single-molecule real-time sequencing. Nature Methods. 2016;13 12:1050-4. doi:10.1038/nmeth.4035.

25. FALCON: experimental PacBio diploid assembler. https://github.com/PacificBiosciences/FALCON/. Accessed 01 Dec 2017.

26. Ultra-fast *de novo* assembler using long noisy reads. https://github.com/ruanjue/smartdenovo. Accessed 01 Dec 2017.

27. A fuzzy Bruijn graph (FBG) approach to long noisy reads assembly. https://github.com/ruanjue/wtdbg-1.2.8. Accessed 01 Dec 2017.

28. Weisenfeld NI, Kumar V, Shah P, Church DM and Jaffe DB. Direct determination of diploid genome sequences. Genome Research. 2017;27 5:757-67. doi:10.1101/gr.214874.116.

29. Pipelines for a *De Novo* Assembly Software: Supernova. https://support.10xgenomics.com/de-novo-assembly/software/overview/latest/welcome. Accessed 01 Dec 2017.

30. A variantCaller tool to get consensus and variant calls from mapped PacBio

reads. https://github.com/PacificBiosciences/GenomicConsensus. Accessed 01 Dec 2017.
31. Li H and Durbin R. Fast and accurate long-read alignment with Burrows–Wheeler transform. Bioinformatics. 2010;26 5:589-95. doi:10.1093/bioinformatics/btp698.
32. Yeo S, Coombe L, Warren RL, Chu J and Birol I. ARCS: scaffolding genome drafts with linked reads. Bioinformatics. 2018;34 5:725-31. doi:10.1093/bioinformatics/btx675.
33. Walker BJ, Abeel T, Shea T, Priest M, Abouelliel A, Sakthikumar S, et al. Pilon: An Integrated Tool for Comprehensive Microbial Variant Detection and Genome Assembly Improvement. PLoS One. 2014;9 11:e112963. doi:10.1371/journal.pone.0112963.
34. Chaisson MJ and Tesler G. Mapping single molecule sequencing reads using basic local alignment with successive refinement (BLASR): application and theory. BMC Bioinformatics. 2012;13 1:238. doi:10.1186/1471-2105-13-238.
35. A long read aligner tool for PacBio. https://github.com/PacificBiosciences/blasr. Accessed 01 Dec 2017.
36. Langmead B and Salzberg SL. Fast gapped-read alignment with Bowtie 2. Nature Methods. 2012;9:357. doi:10.1038/nmeth.1923.
37. RepeatModeler: a *de novo* repeat family identification and modeling package. http://www.repeatmasker.org/RepeatModeler/. Accessed 01 Dec 2017.
38. Bao W, Kojima KK and Kohany O. Repbase Update, a database of repetitive elements in eukaryotic genomes. Mobile DNA. 2015;6:11. doi:10.1186/s13100-015-0041-9.
39. A program that screens DNA sequences for interspersed repeats and low complexity DNA sequences: RepeatMasker. http://www.repeatmasker.org/. Accessed 01 Dec 2017.
40. Bolger AM, Lohse M and Usadel B. Trimmomatic: a flexible trimmer for Illumina sequence data. Bioinformatics. 2014;30 15:2114-20. doi:10.1093/bioinformatics/btu170.
41. Martin M. Cutadapt removes adapter sequences from high-throughput sequencing reads. EMBnetjournal. 2011;17 1 doi:10.14806/ej.17.1.200.
42. Kim D, Langmead B and Salzberg SL. HISAT: a fast spliced aligner with low memory requirements. Nature Methods. 2015;12 4:357-60.
43. A quality control tool for high throughput sequence data. https://www.bioinformatics.babraham.ac.uk/projects/fastqc/. Accessed 01 Dec 2017.
44. Pertea M, Pertea GM, Antonescu CM, Chang T-C, Mendell JT and Salzberg SL. StringTie enables improved reconstruction of a transcriptome from RNA-seq reads. Nature Biotechnology. 2015;33 3:290-5.
45. Grabherr MG, Haas BJ, Yassour M, Levin JZ, Thompson DA, Amit I, et al. Full-length transcriptome assembly from RNA-Seq data without a reference genome. Nature Biotechnology. 2011;29:644. doi:10.1038/nbt.1883.
46. Fu L, Niu B, Zhu Z, Wu S and Li W. CD-HIT: accelerated for clustering the

next-generation sequencing data. Bioinformatics. 2012;28 23:3150-2.
47. Keller O, Kollmar M, Stanke M and Waack S. A novel hybrid gene prediction method employing protein multiple sequence alignments. Bioinformatics. 2011;27 6:757-63. doi:10.1093/bioinformatics/btr010.
48. Stanke M, Diekhans M, Baertsch R and Haussler D. Using native and syntenically mapped cDNA alignments to improve de novo gene finding. Bioinformatics. 2008;24 5:637-44. doi:10.1093/bioinformatics/btn013.
49. Boratyn GM, Schäffer AA, Agarwala R, Altschul SF, Lipman DJ and Madden TL. Domain enhanced lookup time accelerated BLAST. Biology Direct. 2012;7 1:12. doi:10.1186/1745-6150-7-12.
50. Swarbreck D, Wilks C, Lamesch P, Berardini T, Garcia-Hernandez M and Foerster H. The Arabidopsis Information Resource (TAIR): gene structure and function annotation. Nucleic Acids Research. 2007;36:D1009 - 14.
51. Jaillon O, Aury JM, Noel B, Policriti A, Clepet C, Casagrande A, et al. The grapevine genome sequence suggests ancestral hexaploidization in major angiosperm phyla. Nature. 2007;449 7161:463-7. doi:10.1038/nature06148.
52. Tomato Genome Consortium. The tomato genome sequence provides insights into fleshy fruit evolution. Nature. 2012;485 7400:635-41.
53. Cruz F, Julca I, Gómez-Garrido J, Loska D, Marcet-Houben M, Cano E, et al. Genome sequence of the olive tree, *Olea europaea*. GigaScience. 2016;5 1:29. doi:10.1186/s13742-016-0134-5.
54. Slater GSC and Birney E. Automated generation of heuristics for biological sequence comparison. BMC Bioinformatics. 2005. doi:10.1186/1471-2105-6-31.
55. A generic tool for sequence alignment. https://www.ebi.ac.uk/about/vertebrate-genomics/software/exonerate. Accessed 01 Dec 2017.
56. Cantarel BL, Korf I, Robb SM, Parra G, Ross E, Moore B, et al. MAKER: an easy-to-use annotation pipeline designed for emerging model organism genomes. Genome Research. 2008;18 1:188-96. doi:10.1101/gr.6743907.
57. Kent WJ. BLAT--the BLAST-like alignment tool. Genome Research. 2002;12 4:656-64. doi:10.1101/gr.229202.
58. Bairoch A and Apweiler R. The SWISS-PROT protein sequence database and its supplement TrEMBL in 2000. Nucleic Acids Research. 2000;28 1:45-8.
59. Bateman A, Birney E, Cerruti L, Durbin R, Etwiller L, Eddy SR, et al. The Pfam Protein Families Database. Nucleic Acids Research. 2002;30 1:276-80.
60. Punta M, Coggill P, Eberhardt R, Mistry J, Tate J and Boursnell C. The Pfam protein families database. Nucleic Acids Research. 2011;40:D290 - 301.
61. Quevillon E, Silventoinen V, Pillai S, Harte N, Mulder N, Apweiler R, et al. InterProScan: protein domains identifier. Nucleic Acids Research. 2005;33 Web Server issue:W116-W20. doi:10.1093/nar/gki442.
62. National Center for Biotechnology Information. https://www.ncbi.nlm.nih.gov/. Accessed 01 Dec 2017.
63. ExPASy Bioinformatics Resources Portal. http://www.expasy.ch/sprot. Accessed 01 Dec 2017.

64. UniProt. http://www.ebi.ac.uk/uniprot. Accessed 01 Dec 2017.

65. Pfam. http://pfam.xfam.org/. Accessed 01 Dec 2017.

66. The KOG Browser. http://genome.jgi-psf.org/help/kogbrowser.jsf. Accessed 01 Dec 2017.

67. Kanehisa M and Goto S. KEGG: Kyoto Encyclopedia of Genes and Genomes. Nucleic Acids Research. 2000;28 1:27-30.

68. KO (KEGG ORTHOLOGY) Database. http://www.genome.jp/kegg/ko.html. Accessed 01 Dec 2017.

69. Harris MA, Clark J, Ireland A, Lomax J, Ashburner M, Foulger R, et al. The Gene Ontology (GO) database and informatics resource. Nucleic Acids Research. 2004;32 Database issue:D258-61. doi:10.1093/nar/gkh036.

70. Gene Ontology Consortium. http://www.geneontology.org. Accessed 01 Dec 2017.

71. Ellinghaus D, Kurtz S and Willhoeft U. LTRharvest, an efficient and flexible software for *de novo* detection of LTR retrotransposons. BMC Bioinformatics. 2008;9 1:18. doi:10.1186/1471-2105-9-18.

72. Steinbiss S, Willhoeft U, Gremme G and Kurtz S. Fine-grained annotation and classification of *de novo* predicted LTR retrotransposons. Nucleic Acids Research. 2009;37 21:7002-13. doi:10.1093/nar/gkp759.

73. Llorens C, Futami R, Covelli L, Domínguez-Escribá L, Viu JM, Tamarit D, et al. The *Gypsy* Database (GyDB) of mobile genetic elements: release 2.0. Nucleic Acids Research. 2011;39 suppl_1:D70-D4. doi:10.1093/nar/gkq1061.

74. Lloréns C, Futami R, Bezemer D and Moya A. The *Gypsy* Database (GyDB) of mobile genetic elements. Nucleic Acids Research. 2008;36 suppl_1:D38-D46. doi:10.1093/nar/gkm697.

75. SanMiguel P, Gaut BS, Tikhonov A, Nakajima Y and Bennetzen JL. The paleontology of intergene retrotransposons of maize. Nature Genetics. 1998;20 1:43-5. doi:10.1038/1695.

76. Edgar RC. MUSCLE: multiple sequence alignment with high accuracy and high throughput. Nucleic Acids Research. 2004;32 5:1792-7. doi:10.1093/nar/gkh340.

77. Kimura M. A simple method for estimating evolutionary rates of base substitutions through comparative studies of nucleotide sequences. Journal of Molecular Evolution. 1980;16 2:111-20. doi:10.1007/bf01731581.

78. Ma J and Bennetzen JL. Rapid recent growth and divergence of rice nuclear genomes. Proceedings of the National Academy of Sciences of the United States of America. 2004;101 34:12404-10. doi:10.1073/pnas.0403715101.

79. Thomas FS, Aurélie HV, Jeffrey LB, Pierre C, Boulos C, Andrew F, et al. A unified classification system for eukaryotic transposable elements. Nature Reviews Genetics. 2007;8 12:973-82.

80. Miele V, Penel S and Duret L. Ultra-fast sequence clustering from similarity networks with SiLiX. BMC Bioinformatics. 2011;12 1:116. doi:10.1186/1471-2105-12-116.

81. Lyu H, He Z, Wu CI and Shi S. Convergent adaptive evolution in marginal

environments: unloading transposable elements as a common strategy among mangrove genomes. New Phytologist. 2018;217 1:428-38. doi:10.1111/nph.14784.

82. Li L, Stoeckert CJ and Roos DS. OrthoMCL: Identification of Ortholog Groups for Eukaryotic Genomes. Genome Research. 2003;13 9:2178-89. doi:10.1101/gr.1224503.

83. Cheng CY, Krishnakumar V, Chan AP, Thibaud-Nissen F, Schobel S and Town CD. Araport11: a complete reannotation of the *Arabidopsis thaliana* reference genome. The Plant Journal: for cell and molecular biology. 2017;89 4:789-804. doi:10.1111/tpj.13415.

84. Motamayor JC, Mockaitis K, Schmutz J, Haiminen N, Iii DL, Cornejo O, et al. The genome sequence of the most widely cultivated cacao type and its use to identify candidate genes regulating pod color. Genome Biology. 2013;14 6:r53. doi:10.1186/gb-2013-14-6-r53.

85. Wang X, Xu Y, Zhang S, Cao L, Huang Y, Cheng J, et al. Genomic analyses of primitive, wild and cultivated citrus provide insights into asexual reproduction. Nature Genetics. 2017;49:765. doi:10.1038/ng.3839.

86. Tuskan GA, Difazio S, Jansson S, Bohlmann J, Grigoriev I, Hellsten U, et al. The genome of black cottonwood, *Populus trichocarpa* (Torr. & Gray). Science. 2006;313 5793:1596-604. doi:10.1126/science.1128691.

87. Myburg AA, Grattapaglia D, Tuskan GA, Hellsten U, Hayes RD, Grimwood J, et al. The genome of *Eucalyptus grandis*. Nature. 2014; advance online publication. doi:10.1038/nature13308.

88. Bartholome J, Mandrou E, Mabiala A, Jenkins J, Nabihoudine I, Klopp C, et al. High-resolution genetic maps of Eucalyptus improve *Eucalyptus grandis* genome assembly. New Phytologist. 2015;206 4:1283-96. doi:10.1111/nph.13150.

89. Schmutz J, McClean PE, Mamidi S, Wu GA, Cannon SB, Grimwood J, et al. A reference genome for common bean and genome-wide analysis of dual domestications. Nature Genetics. 2014; doi:10.1038/ng.3008.

90. The French–Italian Public Consortium for Grapevine Genome Characterization. The grapevine genome sequence suggests ancestral hexaploidization in major angiosperm phyla. Nature. 2007;449:463. doi:10.1038/nature06148.

91. Canaguier A, Grimplet J, Di Gaspero G, Scalabrin S, Duchêne E, Choisne N, et al. A new version of the grapevine reference genome assembly (12X.v2) and of its annotation (VCost.v3). Genomics Data. 2017;14:56-62. doi:10.1016/j.gdata.2017.09.002.

92. Denoeud F, Carretero-Paulet L, Dereeper A, Droc G, Guyot R, Pietrella M, et al. The coffee genome provides insight into the convergent evolution of caffeine biosynthesis. Science. 2014;345 6201:1181-4. doi:10.1126/science.1255274.

93. Badouin H, Gouzy J, Grassa CJ, Murat F, Staton SE, Cottret L, et al. The sunflower genome provides insights into oil metabolism, flowering and Asterid evolution. Nature. 2017;546:148. doi:10.1038/nature22380.

94. Dohm JC, Minoche AE, Holtgrawe D, Capella-Gutierrez S, Zakrzewski F, Tafer

H, et al. The genome of the recently domesticated crop plant sugar beet (*Beta vulgaris*). Nature. 2013; doi:10.1038/nature12817.

95. Ming R, VanBuren R, Liu Y, Yang M, Han Y, Li L-T, et al. Genome of the long-living sacred lotus (*Nelumbo nucifera* Gaertn.). Genome Biology. 2013;14 5:R41. doi:10.1186/gb-2013-14-5-r41.

96. Filiault D, Ballerini E, Mandakova T, Akoz G, Derieg N, Schmutz J, et al. The *Aquilegia* genome: adaptive radiation and an extraordinarily polymorphic chromosome with a unique history. bioRxiv. 2018; doi:10.1101/264101.

97. Ouyang S, Zhu W, Hamilton J, Lin H, Campbell M, Childs K, et al. The TIGR Rice Genome Annotation Resource: improvements and new features. Nucleic Acids Research. 2007;35 Database issue:D883-7. doi:10.1093/nar/gkl976.

98. Edgar RC. MUSCLE: multiple sequence alignment with high accuracy and high throughput. Nucleic Acids Research. 2004;32 5:1792-7. doi:10.1093/nar/gkh340.

99. Guindon S, Dufayard J-F, Lefort V, Anisimova M, Hordijk W and Gascuel O. New Algorithms and Methods to Estimate Maximum-Likelihood Phylogenies: Assessing the Performance of PhyML 3.0. Systematic Biology. 2010;59 3:307-21. doi:10.1093/sysbio/syq010.

100. Sanderson MJ. r8s: inferring absolute rates of molecular evolution and divergence times in the absence of a molecular clock. Bioinformatics. 2003;19 2:301-2.

101. Wang H, Moore MJ, Soltis PS, Bell CD, Brockington SF, Alexandre R, et al. Rosid radiation and the rapid rise of angiosperm-dominated forests. Proceedings of the National Academy of Sciences of the United States of America. 2009;106 10:3853-8. doi:10.1073/pnas.0813376106.

102. Doyle JA. Molecular and Fossil Evidence on the Origin of Angiosperms. Annual Review of Earth and Planetary Sciences. 2012;40 1:301-26. doi:10.1146/annurev-earth-042711-105313.

103. Wang Y, Tang H, Debarry JD, Tan X, Li J, Wang X, et al. MCScanX: a toolkit for detection and evolutionary analysis of gene synteny and collinearity. Nucleic Acids Research. 2012;40 7:e49. doi:10.1093/nar/gkr1293.

104. Wang D, Zhang Y, Zhang Z, Zhu J and Yu J. KaKs_Calculator 2.0: A Toolkit Incorporating Gamma-Series Methods and Sliding Window Strategies. Genomics, Proteomics & Bioinformatics. 2010;8 1:77-80. doi:https://doi.org/10.1016/S1672-0229(10)60008-3.

105. Suyama M, Torrents D and Bork P. PAL2NAL: robust conversion of protein sequence alignments into the corresponding codon alignments. Nucleic Acids Research. 2006;34 Web Server issue:W609-W12. doi:10.1093/nar/gkl315.

106. De Bie T, Cristianini N, Demuth JP and Hahn MW. CAFE: a computational tool for the study of gene family evolution. Bioinformatics. 2006;22 10:1269-71. doi:10.1093/bioinformatics/btl097.

107. Chae L, Kim T, Nilo-Poyanco R and Rhee SY. Genomic signatures of specialized metabolism in plants. Science. 2014;344 6183:510-3.

108. Nützmann H-W, Huang A and Osbourn A. Plant metabolic clusters – from

genetics to genomics. New Phytologist. 2016;211 3:771-89. doi:10.1111/nph.13981.

109. Nützmann H-W and Osbourn A. Gene clustering in plant specialized metabolism. Current Opinion in Biotechnology. 2014;26:91-9. doi:https://doi.org/10.1016/j.copbio.2013.10.009.

110. Kautsar SA, Suarez Duran HG, Blin K, Osbourn A and Medema MH. plantiSMASH: automated identification, annotation and expression analysis of plant biosynthetic gene clusters. Nucleic Acids Research. 2017;45 W1:W55-W63. doi:10.1093/nar/gkx305.

111. Schlapfer P, Zhang P, Wang C, Kim T, Banf M, Chae L, et al. Genome-wide prediction of metabolic enzymes, pathways and gene clusters in plants. Plant Physiology. 2017; doi:10.1104/pp.16.01942.

112. PMN: A plant metabolic pathway databases. https://www.plantcyc.org/. Accessed 01 Dec 2017.

113. E2P2: An enzyme annotation pipeline used to generate the species-specific metabolic databases. https://gitlab.com/rhee-lab/E2P2/tree/master. Accessed 01 Dec 2017.

114. Yasuno R, von Wettstein-Knowles P and Wada H. Identification and molecular characterization of the β-ketoacyl-[acyl carrier protein] synthase component of the Arabidopsis mitochondrial fatty acid synthase. Journal of Biological Chemistry. 2004;279 9:8242-51.

115. Jakobsson A, Westerberg R and Jacobsson A. Fatty acid elongases in mammals: their regulation and roles in metabolism. Progress in Lipid Research. 2006;45 3:237-49.

Figures

Fig. 1 Images of *M. oleifera*, recorded distribution range and sampling sites.

a-d, mature tree (a), flower (b), fruit (c) and naturally germinated seedling (d); e, blue shaded region denotes the reported distribution range of *M. oleifera*, while the red circle denotes the position (N23.90°, E104.09°, Guangnan County, Yunnan) where one tree was sampled for whole genome sequencing, and the red triangle and square denote the positions (N23.9°, E106.00°, Funing County, Yunnan and N24.78°, E106.57°, Leye County, Guangxi) where trees were sampled for RNA sequencing.

Fig. 2 Repeat composition, phylogenomic inferences and biosynthesis pathway for very long chain fatty acids synthesis in *M. oleifera*.

a. genome proportions of genic and various repeat sequences; b. phylogenetic tree, divergence time, and profiles of gene families that underwent expansion or contraction; c. annotated genes involved in the biosynthesis pathway of very long chain fatty acids (a fatty acid with minimum 22 carbon moieties) in *M. oleifera*.

Table

Table 1. Statistics of the final genome assembly for *M. oleifera*.

Supplementary Figures

Fig. S1. Length distribution of PacBio subreads.

Fig. S2. K-mer frequency distribution estimated from PacBio sequences after filtering and correction at k-mer size of 17. A k-mer refers to an artificial sequence division of K nucleotides. From k-mer frequencies, genomic characteristics (genome size, repeat structure and heterozygous rate) could be estimated. Peaks at depth of 21 are annotated with dashed lines.

Fig. S3. Distribution of AED (annotation edit distance) scores from gene prediction. AED = 0 indicates perfect agreement between the gene prediction and the transcript and protein evidence; AED = 1 indicates no evidence support for annotation.

Fig. S4. Proliferation history of different superfamilies of the *Gypsy* class of LTR-RTs (long terminal repeat-retrotransposons) in the *M. oleifera* genome.

Fig. S5. Proliferation history of different superfamilies of the *Copia* class of LTR-RTs in the *M. oleifera* genome.

Fig. S6. Gene proximity for different superfamilies of the *Gypsy* class of LTR-RTs in the *M. oleifera* genome.

The natural logarithm of the base distance between an LTR-RT and an adjacent gene (plus one) was used as the X axis.

Fig. S7. Gene proximity for different superfamilies of the *Copia* class of LTR-RTs in the *M. oleifera* genome.

The natural logarithm of the base distance between an LTR-RT and an adjacent gene

(plus one) was used as the X axis.

Fig. S8. Gene proximity and insertion time for major superfamilies of the *Gypsy* class of LTR-RTs in the *M. oleifera* genome.

The natural logarithm of the base distance between an LTR-RT and an adjacent gene (plus one) was used as the Y axis, time in mya as X axis.

Fig. S9. Gene proximity and insertion time for major superfamilies of the *Copia* class of LTR-RTs in the *M. oleifera* genome.

The natural logarithm of the base distance between an LTR-RT and an adjacent gene (plus one) was used as the Y axis, time in mya as X axis.

Fig. S10. Birth and death of LTR-RTs (long terminal repeat-retrotransposons) in the *M. oleifera* genome compared to six other members of Rosids and two members from Asterids. (a) total numbers of intact LTR-RTs in the genome; (b) comparison of $S + T$ values among these nine plant species; (c) total numbers of intact LTR-RTs and traces of LTR-RT death; (d) ratios of solo-LTR to intact LTR-RT ($S{:}I$); (e) proportions of LTR-RTs found in the clusters with high removal rates (filtered $S{:}I \geqslant 3$). S, number of solo-LTRs; T, number of truncated LTR-RTs I, number of intact LTR-RTs.

Fig. S11. *Ks* distribution of paralogs in synteny blocks within the *M. oleifera* genome.

Fig. S12. Gene:synteny-block pattern in the *M. oleifera* genome.

Fig. S13. Genes annotation for the initial (*de novo*) fatty acid biosynthesis process in the *M. oleifera* genome.

Supplementary Tables

Table S1. Summary of Pacbio and Illumina sequencing data (10x Genomics and RNA sequencing) generated in the present study. IDs of the study, sample, library and accessions in NCBI SRA and employed sequencing platform, material origins of the sequenced DNA or RNA, statistics of raw and cleaned data, and mapping rates are shown.

Table S2. Data summary from 10x Genomics analysis based on GemCode index multiplicity. Read subsets are based on the number of associated reads for each index. For raw reads, all indices (including those with N's) are included in the count. For all other read sets, only the indices without N's were used for binning.

Table S3. Estimation of genome characteristics based on 17-mer statistics.

Table S4. Statistics of the different versions of *M. oleifera* genome assembly in ascending order. N50: shortest sequence length at 50% of the genome; L50: smallest number of contigs whose length sum produces N50. NA: data not available; * statistics for contigs/scaffolds. Gene completeness was generated by assessment with 1,440 single copy orthologs from the BUSCO embryophyta_odb9 database.

Table S5. Summary of the annotated TEs in the genome assembly for *M. oleifera*. LTR: Long Terminal Repeat retrotransposons; LINE: Long Interspersed Nuclear Element, a category of non-LTR (long terminal repeat) retroelements; SINE: Short Interspersed Nuclear Element, a category of non-autonomous and non-coding retroelements (TEs); RC: Rolling-circle transposons.

Table S6. Summary of transcriptome assemblies using three different analysis pipelines.

Table S7. Summary of annotated genes.

AED: Annotation Edit Distance; gene region (including 5', 3' UTRs, exons and introns).

Table S8. Summary of BUSCO evaluation for gene prediction.

Table S9. Summary of functional annotation of predicted genes.

Table S10. Superfamilies within the *Gypsy* and *Copia* LTR-RTs classes of TEs.

Table S11. Gene proximity of superfamilies of *Gypsy* and *Copia* classes of LTR-RTs.

Table S12. Comparison of the number of original and filtered intact LTR-RT, solo-LTR and Truncated LTR TEs among 9 plant species.

Table S13. Genomic data used for phylogenomic and gene family analyses. Origins, download links, assembly versions, genome properties and references of 14 genomes are shown.

Table S14. Summary of gene family analyses. Unique groups and genes, single-copy and duplicated groups and genes are summarized for the 15 analyzed plant genomes.

Table S15. GO enrichment of expanded gene families. (A) 'Category' is the Gene Ontology (GO) term ID; (B) 'P_value' is the overrepresentation p-value indicating the observed frequency of a given term among analyzed genes is equal to the expected frequency based on the null distribution; i.e. lower p-values indicate stronger evidence for overrepresentation; (C) 'Q_value' is the Benjamini and Hochberg adjusted p-value, (D) 'numEPInCat' is the number of expanded gene families in the corresponding GO category; (E) 'numInCat' is the number of detected gene families in the corresponding GO category; (F) 'Term' is the GO term; (G) 'Ontology' indicates which ontology the

term comes from. Significant at q < 0.05.

Table S16. KEGG enrichment of expanded gene families. (A) ‘KO category’ is the KEGG Orthology (KO) category ID; (B) ‘P_value’ is the over represented p-value indicating the observed frequency of a given term among analyzed genes is equal to the expected frequency based on the null distribution; i.e. lower p-values indicate stronger evidence for overrepresentation; (C) ‘Q_value’ is the Benjamini and Hochberg adjusted p-value, (D) ‘numEPInCat’ is the number of expanded gene families in the corresponding KO category; (E) ‘numInCat’ is the number of detected gene families in the corresponding KO category; (F) ‘Pathway’ is the KEGG pathway; (G) ‘Class’ indicates which KEGG class the pathway comes from. Significant at q < 0.05.

Table S17. GO enrichment of contracted gene families. (A) ‘Category’ is the Gene Ontology (GO) term ID; (B) ‘P_value’ is the over represented p-value indicating the observed frequency of a given term among analyzed genes is equal to the expected frequency based on the null distribution; i.e. lower p-values indicate stronger evidence for overrepresentation; (C) ‘Q_value’ is the Benjamini and Hochberg adjusted p-value, (D) ‘numEPInCat’ is the number of expanded gene families in the corresponding GO category; (E) ‘numInCat’ is the number of detected gene families in the corresponding GO category; (F) ‘Term’ is the GO term; (G) ‘Ontology’ indicates which ontology the term comes from. Significant at q < 0.05.

Table S18. KEGG enrichment of contracted gene families. (A) ‘KO category’ is the KEGG Orthology (KO) category ID; (B) ‘P_value’ is the over represented p-value indicating the observed frequency of a given term among analyzed genes is equal to the

expected frequency based on the null distribution; i.e. lower p-values indicate stronger evidence for overrepresentation; (C) ‘Q_value’ is the Benjamini and Hochberg adjusted p-value, (D) ‘numEPInCat’ is the number of expanded gene families in the corresponding KO category; (E) ‘numInCat’ is the number of detected gene families in the corresponding KO category; (F) ‘Pathway’ is the KEGG pathway; (G) ‘Class’ indicates which KEGG class the pathway comes from. Significant at q < 0.05.

Table S19. GO enrichment of fast evolving gene families. (A) ‘Category’ is the Gene Ontology (GO) term ID; (B) ‘P_value’ is the over represented p-value indicating the observed frequency of a given term among analyzed genes is equal to the expected frequency based on the null distribution; i.e. lower p-values indicate stronger evidence for overrepresentation; (C) ‘Q_value’ is the Benjamini and Hochberg adjusted p-value, (D) ‘numEPInCat’ is the number of expanded gene families in the corresponding GO category; (E) ‘numInCat’ is the number of detected gene families in the corresponding GO category; (F) ‘Term’ is the GO term; (G) ‘Ontology’ indicates which ontology the term comes from. Significant at q < 0.05.

Table S20. KEGG enrichment of fast evolving gene families. (A) ‘KO category’ is the KEGG Orthology (KO) category ID; (B) ‘P_value’ is the over represented p-value indicating the observed frequency of a given term among analyzed genes is equal to the expected frequency based on the null distribution; i.e. lower p-values indicate stronger evidence for overrepresentation; (C) ‘Q_value’ is the Benjamini and Hochberg adjusted p-value, (D) ‘numEPInCat’ is the number of expanded gene families in the corresponding KO category; (E) ‘numInCat’ is the number of detected gene families in

the corresponding KO category; (F) ‘Pathway’ is the KEGG pathway; (G) ‘Class’ indicates which KEGG class the pathway comes from. Significant at $q < 0.05$.

Table S21. Summary of 23 metabolic gene clusters in the *M. oleifera* genome. Genomic coordinates, gene composition, core protein domains related to metabolism and pathway assignments are shown.

Supplementary Files

Supplementary File 1. Visualization of each metabolic gene cluster detected within the *M. oleifera* genome.

Figures

Fig. 1 Images of *M. oleifera*, recorded distribution range and sampling sites.

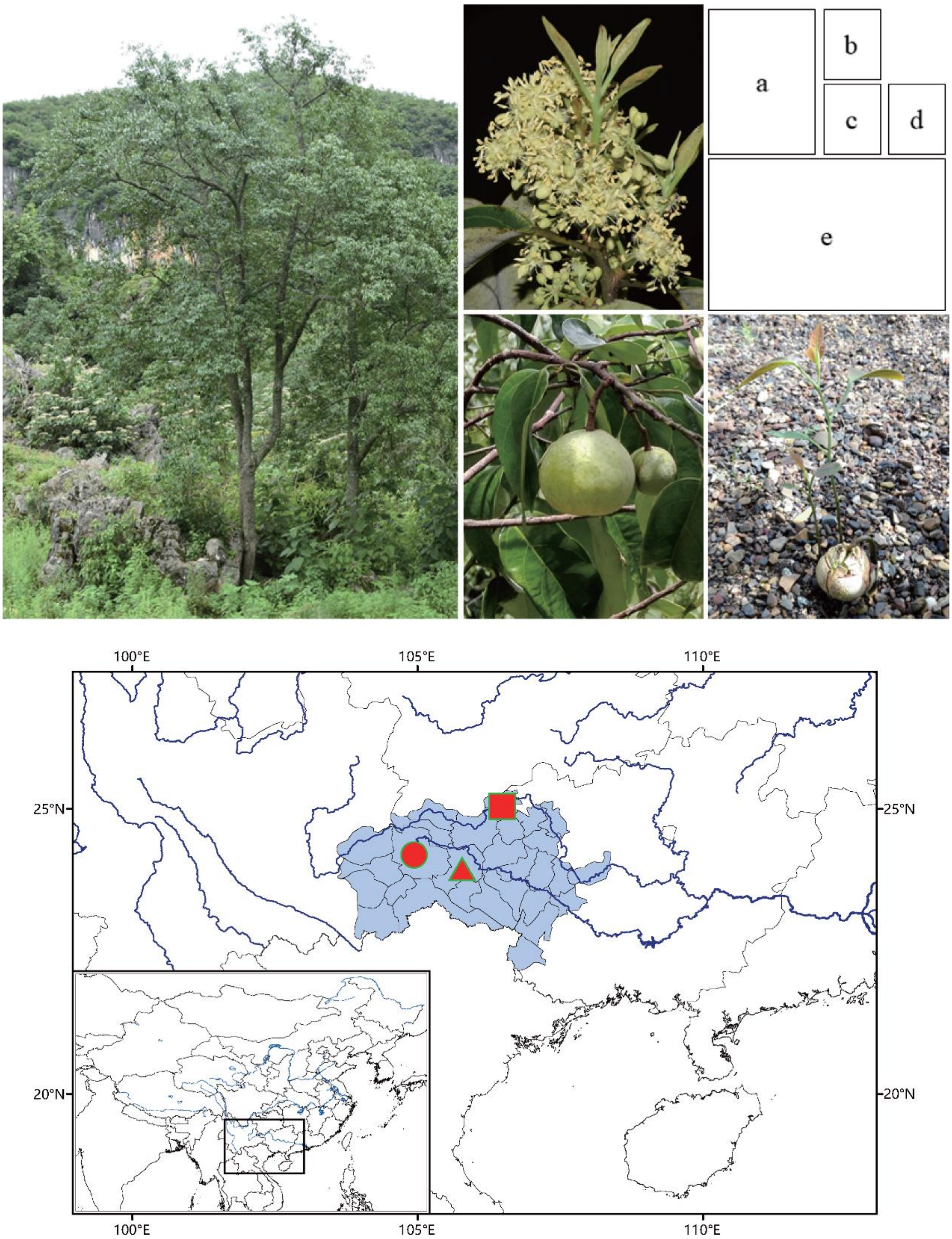

Fig. 2 Repeat composition for *M. oleifera*, phylogenomic depiction of gene family expansions and contractions, and biosynthesis pathway for very long chain fatty acids synthesis in *M. oleifera*.

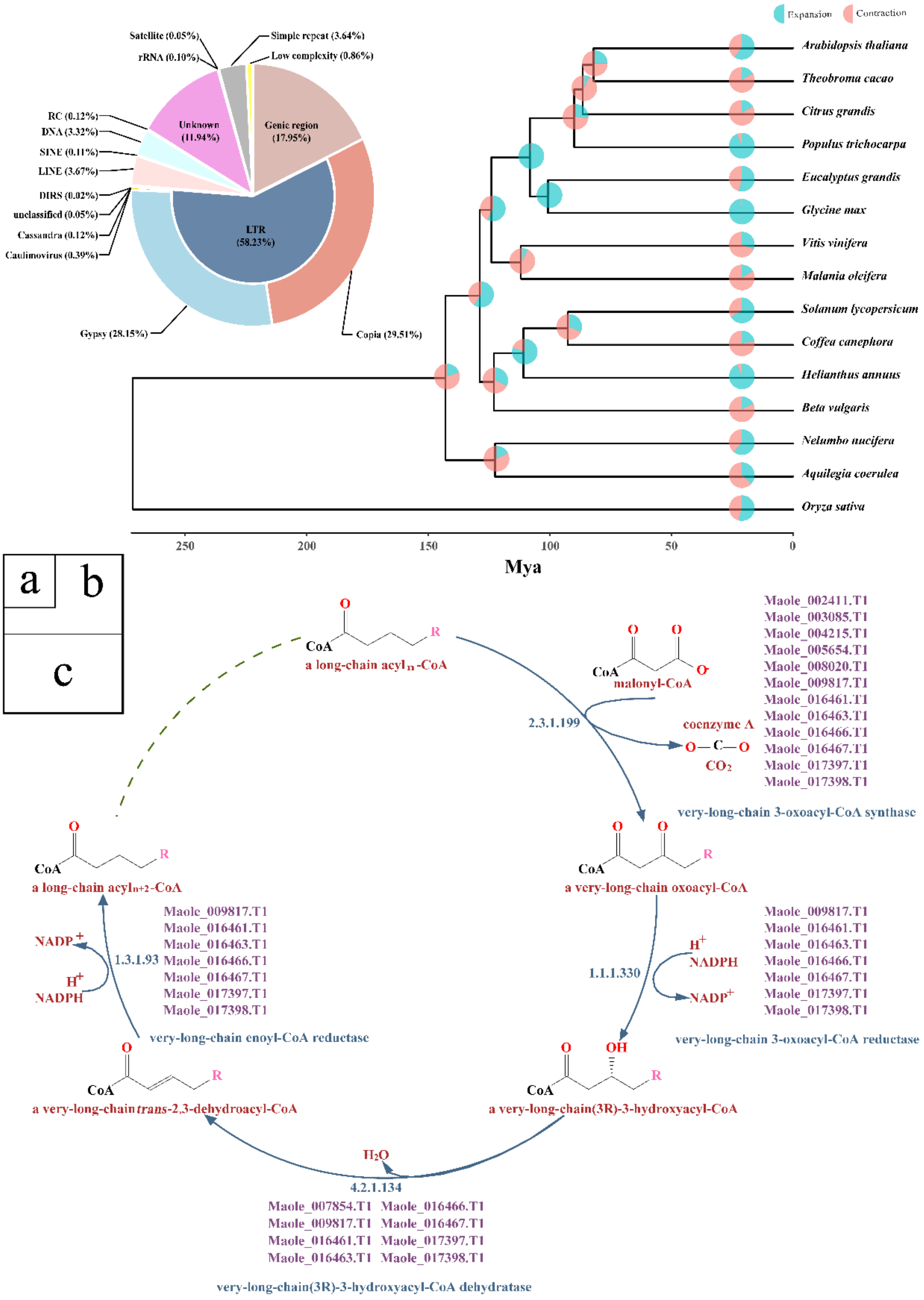

Tables

Table 1. Statistics of the final genome assembly for *M. oleifera*.

	Contig		Scaffold	
	**Size (bp)**	**Number**	**Size (bp)**	**Number**
**Total Size**	1,509,344,141	-	1,519,782,615	-
**Total Number**	-	2,987	-	1,277
**N10**	2,959,726	39	11,755,999	10
**N50**	1,218,690	376	4,647,296	94
**N90**	272,293	1,337	1,153,659	339
**Max.**	6,703,356	-	25,060,663	-
**Min.**	334	-	8256	-
**Mean**	505,304	-	1,190,119	-
**Median**	200,407	-	85,436	-
**Gap**	-	-	10,438,474 (0.69%)	1,710
**GC Content**	36.07%	-	35.82%	-

-, Data not available.

Click here to download Table Table S1.xlsx

Accession	Study	Bioprojec	Biosample_accession	Library_l	Library_s	Library_s
SRR7221533	SRP14901	PRJNA47	SAMN09235853	DS-pacbic	WGS	GENOMI
SRR7221532	SRP14901	PRJNA47	SAMN09235853	DS-10x	WGS	GENOMI
SRR7221530	SRP14901	PRJNA47	SAMN09235854	MoF1	RNA-Seq	TRANSC
SRR7221531	SRP14901	PRJNA47	SAMN09235855	MoF2	RNA-Seq	TRANSC
SRR7221537	SRP14901	PRJNA47	SAMN09235856	MoF3	RNA-Seq	TRANSC
SRR7221536	SRP14901	PRJNA47	SAMN09235857	MoF4	RNA-Seq	TRANSC
SRR7221535	SRP14901	PRJNA47	SAMN09235858	Mole	RNA-Seq	TRANSC
SRR7221534	SRP14901	PRJNA47	SAMN09235859	leaf	RNA-Seq	TRANSC
SRR7221538	SRP14901	PRJNA47	SAMN09235860	seed	RNA-Seq	TRANSC

Library_s	Library_l	Platform	Instrume	Sample
RANDOM	single	PACBIO_SMI	PacBio Se	leaves
RANDOM	paired	ILLUMINA	HiSeq X T	leaves
RANDOM	paired	ILLUMINA	Illumina H	fruits
RANDOM	paired	ILLUMINA	Illumina H	fruits
RANDOM	paired	ILLUMINA	Illumina H	fruits
RANDOM	paired	ILLUMINA	Illumina H	fruits
RANDOM	paired	ILLUMINA	Illumina H	leaves
RANDOM	paired	ILLUMINA	Illumina H	leaves
RANDOM	paired	ILLUMINA	Illumina H	seeds

Geological_location	Collectors	Dates
China:Guangnan county, Yunnan, China	Yongpeng Ma, Jian-Feng Mao	2017.09.1
China:Guangnan county, Yunnan, China	Yongpeng Ma, Jian-Feng Mao	2017.09.1
China:Funing county, Yunnan, China	Sihai Wang, Yongpeng Ma	2016.07.0
China:Funing county, Yunnan, China	Sihai Wang, Yongpeng Ma	2016.07.2
China:Funing county, Yunnan, China	Sihai Wang, Yongpeng Ma	2016.08.1
China:Funing county, Yunnan, China	Sihai Wang, Yongpeng Ma	2016.08.2
China:Funing county, Yunnan, China	Sihai Wang, Yongpeng Ma	2016.08.2
China:Leye county, Guangxi, China	Dong-Xu Zhang	2013.10.0
China:Leye county, Guangxi, China	Dong-Xu Zhang	2014.09.0

Latitude-	Raw Reads (Million)	Raw Bases (Gb)	Raw Q20 (Gb)	Raw Q30 (Gb)
N23.90, E	5.778	51.15	NA	NA
N23.90, E	899.778	134.967	120.195(89.1 %)	107.959(80.0 %)
N23.9, E1	40.972	6.146	6.059(98.6 %)	5.914(96.2 %)
N23.9, E1	48.203	7.23	7.112(98.4 %)	6.903(95.5 %)
N23.9, E1	43.559	6.534	6.451(98.7 %)	6.321(96.7 %)
N23.9, E1	42.383	6.357	6.254(98.4 %)	6.071(95.5 %)
N23.9, E1	47.835	7.175	7.054(98.3 %)	6.842(95.3 %)
N24.78, E	46.365	4.59	4.553(99.2 %)	4.356(94.9 %)
N24.78, E	44.041	4.404	4.331(98.3 %)	4.115(93.4 %)

Clean Reads (Million)	Clean Bases (Gb)	Clean Q2	Clean Q3	Average Length (bp)
NA	NA	NA	NA	8,852
NA	NA	NA	NA	150
40.181(98.1 %)	5.930(96.5 %)	5.883(99.[illegible]	5.774(97.[illegible]	147.6
47.097(97.7 %)	6.867(95.0 %)	6.812(99.[illegible]	6.667(97.[illegible]	145.8
42.690(98.0 %)	6.245(95.6 %)	6.207(99.[illegible]	6.117(98.[illegible]	146.3
41.475(97.9 %)	6.063(95.4 %)	6.014(99.[illegible]	5.886(97.[illegible]	146.2
46.688(97.6 %)	6.850(95.5 %)	6.793(99.[illegible]	6.645(97.[illegible]	146.7
44.637(96.3 %)	4.386(95.6 %)	4.365(99.[illegible]	4.229(96.[illegible]	98.3
41.339(93.9 %)	4.097(93.0 %)	4.047(98.[illegible]	3.911(95.[illegible]	99.1

Multiple Mapped (M)	Uniquely Mapped (M)
NA	NA
NA	NA
7.486(18.63 %)	31.861(79.29 %)
0.726(1.54 %)	45.286(96.15 %)
4.921(11.53 %)	36.389(85.24 %)
1.027(2.48 %)	39.583(95.44 %)
1.012(2.17 %)	43.978(94.20 %)
3.871(8.67 %)	4.603(10.31 %)
2.305(5.58 %)	35.394(85.62 %)

 Click here to download Table Table S2.xlsx

**Number of read pairs per index**	**Raw reads**	**>=1**	**>=1000**
**Number of indices**	19,394,967	19,319,151	27,368
**Total number of read pairs**	449,888,927	449,809,812	42,975,267
**Percent of total read pairs**	100%	99.98%	9.55%
**Average number of read pairs per index**	23.20	23.28	1,570.27

**>=3000**	**>=5000**
830	450
9,529,752	8,090,850
2.12%	1.80%
11,481.63	17,979.67

K-mer value	Amount of K-mer used	Coverage	Genome size	Heterozygosity rate (%)
17	29,971,959,192	21	1498597960	0.056626386

Repeat rate (%)	Error rate (%)
54.60826	0.336212454

Versions of assembly	Strategy	Assemblied genome size (Gb)
v0.1	Canu	1.53
v0.2	MECAT	1.19
v0.3	minimap+miniasm	1.55
v0.4	Canu+FALCON	1.54
v0.5	Canu+SMARTdenovo	1.45
v0.6	Canu+Wtdbg	1.46
v0.7	SMARTdenov	1.51
v0.8	Wtdbg	1.61
v0.9	Supernova	1.56/1.63*
v1.0	v0.7+arrow×2	1.51
v1.1	v1.0+arcs	1.52/1.51*
v1.2f	v1.1+arrow+pilon×3	1.52/1.51*

Sequence number	N50	L50	Max. length
4,396	1.12 Mb	381	9.05 Mb
14,019	117 Kb	2,968	1.37 Mb
4,276	744 Kb	631	3.73 Mb
7,860	665 Kb	646	4.94 Mb
4,341	611 Kb	674	4.5 Mb
5,709	782 Kb	538	5.13 Mb
3,038	1.21 Mb	380	6.72 Mb
9,743	549 Kb	885	3.52 Mb
110,395/58,910*	46 Kb/1.57 Mb*	9397/277*	570 Kb/9.29 Mb*
3,038	1.20 Mb	380	6.7 Mb
3,038/1,274*	1.20 Mb/4.65 Mb*	380/94*	6.7 Mb/25.1 Mb*
2,987/1,277*	1.22 Mb/4.65 Mb*	376/94*	6.7 Mb/25.1 Mb*

Gene completeness (%)
86.00%
NA
NA
NA
85.20%
79.90%
71.80%
56.10%
90.00%
88.30%
88.60%
90.60%

**Class**	**Family**	**Number**	**Length (bp)**	**Percent (%)**
LTR		981,473	884,911,643	58.23
	Cassandra	3,798	1,780,693	0.12
	Caulimovirus	6,736	5,864,500	0.39
	Copia	537,029	448,422,064	29.51
	DIRS	1,298	265,737	0.02
	Gypsy	430,047	427,864,686	28.15
LINE		89,562	55,769,410	3.67
	CR1	6,538	2,307,810	0.15
	L1	58,839	36,673,807	2.41
	L1-Tx1	290	88,189	0.01
	L2	3,942	1,546,597	0.10
	RTE-BovB	19,953	15,153,007	1.00
SINE		11,788	1,700,153	0.11
	tRNA-Core-RTE	1,959	267,428	0.02
	tRNA-RTE	9,829	1,432,725	0.09
DNA		114,267	50,512,738	3.32
	CMC-Chapaev	1,991	190,839	0.01
	CMC-EnSpm	44,914	23,758,719	1.56
	MuLE-MuDR	6,966	6,239,911	0.41
	TcMar-Tc1	738	143,208	0.01
	hAT-Ac	28,249	8,002,000	0.53
	hAT-Tag1	22,706	9,286,682	0.61
	hAT-Tip100	3,359	1,172,790	0.08
RC		2,464	1,832,449	0.12
	Helitron	2,464	1,832,449	0.12
Unknown		495,092	181,461,434	11.94
rRNA		3,065	1,467,141	0.10
Satellite		4,125	815,956	0.05
Simple_repeat		469,716	55,388,128	3.64
Low_complexity		51,628	13,100,851	0.86
Total		2,223,180	1,246,959,903	82.05

Mean_length (bp)
901.62
468.85
870.62
835.01
204.73
994.93
622.69
352.98
623.29
304.10
392.34
759.44
144.23
136.51
145.77
442.06
95.85
528.98
895.77
194.05
283.27
409.00
349.15
743.69
743.69
366.52
478.68
197.81
117.92
253.75
560.89

 Click here to download Table Table S6.xlsx

**Pipelines**	**Number of genes**	**Total length of genes (bp)**
Trinity *de novo*	49,316	33,821,803
Trinity genome-guided	33,891	27,185,969
StringTie	28,782	45,886,824
Merged transcriptome	NA	NA

Average length of genes (bp	Number of transcripts	Total length of transcripts (
685.82	52,509	36,893,680
802.16	35,493	28,915,705
1,594.29	42,176	61,063,687
NA	57,299	65,681,138

Average length of transcrip	N50 of transcripts (bp)
702.62	938
814.69	1,136
1,447.83	2,003
1,146.29	1,760

Gene Number	Transcript Number	Transcript Number (AED<(
24,094	24,094	20,092

Average Gene Region Lengt	Average Transcript Length	Average CDS Length (bp)
11,809.11	1,460.47	1281.10

Average Exons per Transcri	Average Exon Length (bp)	Average Intron Length (bp)
5.97	244.46	650.22

Table Click here to download Table Table S8.xlsx

	BUSCO groups	Percentage
Complete BUSCOs	1,258	87.36%
Complete and single-copy BUSCOs	1,217	84.51%
Complete and duplicated BUSCOs	41	2.85%
Fragmented BUSCOs	71	4.93%
Missing BUSCOs	111	7.71%
Total BUSCO groups searched	1,440	100.00%

Click here to download Table Table S9.xlsx

Total genes
Annotated

Unannotated

Databases	Count	Percentage
	24,094	100.00%
NR	13,792	57.20%
Swiss_Prot	21,833	90.60%
TrEMBL	22,026	91.40%
Pfam	18,512	76.80%
KOG	21,102	87.60%
GO	18,972	78.70%
KO	7,923	32.90%
	2,023	8.40%

Table

Click here to download Table Table S10.xlsx

**Super families**	**Clade ID**	**Count**	**Average length (bp)**
Gypsy	Del	12,194	8,706.42
Gypsy	Galadriel	452	6,318.11
Gypsy	Tat	343	10,297.52
Gypsy	Athila	335	10,362.77
Gypsy	CRM	318	7,645.90
Gypsy	Reina	85	7,271.95
Gypsy	GRhodo	25	9,071.04
Gypsy	412/mdg1	24	9,184.25
Gypsy	Mag	19	8,713.16
Gypsy	V_clade	13	8,207.62
Gypsy	Maggy	13	9,320.46
Gypsy	Osvaldo	12	8,243.75
Gypsy	Pyret	11	8,567.18
Gypsy	Gmr1	9	8,627.89
Gypsy	MGLR3	8	9,371.75
Gypsy	Tse3	7	8,304.14
Gypsy	Cer1	7	8,044.00
Gypsy	Pyggy	3	9,703.33
Gypsy	Micropia/mdg3	3	8,355.00
Gypsy	Ty3	2	7,028.00
Gypsy	Tor1	1	4,848.00
Gypsy	REM	1	7,502.00
Gypsy	Gypsy	1	7,359.00
Gypsy	CsRN1	1	8,983.00
Gypsy	Cer2_3	1	11,592.00
Gypsy	17.6	1	7,832.00
Gypsy	unclassified	24	8,115.83
Gypsy	total	13,913	8,674.40
Copia	Sire	6,051	9,473.96
Copia	Tork	2,890	6,094.98
Copia	Oryco	756	6,427.50
Copia	Retrofit	681	5,886.53
Copia	Osser	33	8,140.48
Copia	Ty	24	8,040.83
Copia	Copia	17	6,142.82
Copia	CoDi_D	16	6,681.44
Copia	GalEA	14	8,214.71
Copia	pCretro	13	6,857.62
Copia	Hydra	13	7,729.23
Copia	CoDi_I	9	7,401.11
Copia	Mtanga	8	5,132.75
Copia	Tricopia	6	8,549.17
Copia	1731	6	7,171.83
Copia	CoDi_C	2	4,823.00
Copia	PyRE1G1	1	5,020.00
Copia	total	10,540	8,064.86

Total length (bp)	Proportion of genome (%)
106,166,026	6.99
2,855,785	0.19
3,532,051	0.23
3,471,527	0.23
2,431,396	0.16
618,116	0.04
226,776	0.01
220,422	0.01
165,550	0.01
106,699	0.01
121,166	0.01
98,925	0.01
94,239	0.01
77,651	0.01
74,974	0.00
58,129	0.00
56,308	0.00
29,110	0.00
25,065	0.00
14,056	0.00
4,848	0.00
7,502	0.00
7,359	0.00
8,983	0.00
11,592	0.00
7,832	0.00
194,780	0.01
120,686,867	7.94
57,326,922	3.77
17,614,500	1.16
4,859,193	0.32
4,008,728	0.26
268,636	0.02
192,980	0.01
104,428	0.01
106,903	0.01
115,006	0.01
89,149	0.01
100,480	0.01
66,610	0.00
41,062	0.00
51,295	0.00
43,031	0.00
9,646	0.00
5,020	0.00
85,003,589	5.59

 Click here to download Table Table S11.xlsx

**Super families**	**Clade ID**	**Overlapping**	**< 100 bp**	**101-1,000 bp**
Gypsy	Del	1,539	7	134
Gypsy	Galadriel	218	3	31
Gypsy	Tat	63		25
Gypsy	Athila	68	1	5
Gypsy	CRM	95	2	9
Gypsy	Reina	51		2
Gypsy	GRhodo	1	0	0
Gypsy	412/mdg1	3	0	0
Gypsy	Mag	6	0	1
Gypsy	V_clade	3	0	0
Gypsy	Maggy	4	0	0
Gypsy	Osvaldo	3	0	0
Gypsy	Pyret	2	0	0
Gypsy	Gmr1	0	0	0
Gypsy	Tse3	1	0	0
Gypsy	MGLR3	1	0	0
Gypsy	Cer1	3	0	0
Gypsy	Pyggy	1	0	0
Gypsy	Micropia/mdg3	2	0	0
Gypsy	Ty3	1	0	0
Gypsy	Tor1	0	0	0
Gypsy	REM	0	0	0
Gypsy	CsRN1	0	0	0
Gypsy	Cer2_3	1	0	0
Gypsy	17.6	0	0	0
Copia	Sire	474	6	64
Copia	Tork	913	23	240
Copia	Oryco	257	5	46
Copia	Retrofit	326	9	70
Copia	Osser	5	0	1
Copia	Ty	6	0	4
Copia	Copia	6	0	0
Copia	CoDi_D	10	0	1
Copia	GalEA	6	0	1
Copia	pCretro	5	0	2
Copia	Hydra	4	0	1
Copia	CoDi_I	3	0	0
Copia	Mtanga	2	0	0
Copia	Tricopia	2	0	0
Copia	1731	3	0	1
Copia	CoDi_C	2	0	0
Copia	PyRE1G1	0	0	0

Distance to the closest gene			
**1,001-10,000 bp**	**10,001-100,000 bp**	**100,001-1,000,000 bp**	**Total**
3,682	6,389	384	12,135
111	83	5	451
99	138	16	341
54	179	22	329
69	117	19	311
11	19	2	85
13	11	0	25
6	12	2	23
5	7	0	19
6	4	0	13
5	4	0	13
3	5	1	12
4	5	0	11
3	6	0	9
1	5	0	7
2	4	0	7
1	3	0	7
1	1	0	3
1	0	0	3
0	1	0	2
0	1	0	1
0	1	0	1
0	1	0	1
0	0	0	1
1	0	0	1
1,151	3,662	625	5,982
963	711	33	2,883
224	190	25	747
154	115	5	679
6	17	3	32
7	7	0	24
4	6	1	17
3	2	0	16
4	3	0	14
3	3	0	13
1	7	0	13
1	3	2	9
4	2	0	8
3	1	0	6
1	1	0	6
0	0	0	2
0	1	0	1

**Species**	**Intact LTR-RT (*I*)**	**Cluster number**	**Solo-LTR (*S*)**
Arabidopsis thaliana	237	142	222
Coffea canephora	1,843	438	9,337
Eucalyptus grandis	3,353	536	7,991
Glycine max	4,847	663	55,534
Populus trichocarpa	1,290	429	4,843
Solanum lycopersicum	3,899	379	22,775
Theobroma cacao	1,497	272	13,202
Vitis vinifera	2,411	357	9,738
Malania oleifera	24,660	4,960	56,170

Truncated LTR (*T*)	*S* +*T*	*I* +*S* +*T*	Filtered scaffold length (kb)	Filtered *I*
203	425	662	0	237
2,829	12,166	14,009	0	1,843
2,500	10,491	13,844	70	3,203
8,481	64,015	68,862	900	4,777
2,243	7,086	8,376	2,000	1,098
5,839	28,614	32,513	0	3,899
2,288	15,490	16,987	50	1,485
3,684	13,422	15,833	0	2,411
8,196	64,366	89,026	0	24,660

**Filtered *S***	**Filtered *T***	**Filtered *S* /*I***	**Filtered *T* /*I***	**Filtered (*S* +*T*)/*I***
222	203	0.94	0.86	1.79
9,337	2,829	5.07	1.54	6.60
7,116	2,209	2.22	0.69	2.91
52,980	8,269	11.09	1.73	12.82
3,827	1,730	3.49	1.58	5.06
22,775	5,839	5.84	1.50	7.34
13,020	2,256	8.77	1.52	10.29
9,738	3,684	4.04	1.53	5.57
56,170	8,196	2.28	0.33	2.61

**LTR-RT (*S* /*I* >=3)**
11.39%
39.34%
18.30%
46.58%
33.61%
69.15%
72.32%
40.36%
16.77%

Table Click here to download Table Table S13.docx

Species	Links	Version	Genes	Genome size (Mb)	Scaffold N50 (Mb)	References
Beta vulgaris	ftp://ftp.ncbi.nlm.nih.gov/genomes/all/GCF/000/511/025/GCF_000511025.2_RefBeet-1.2.2	RefBeet-1.2.2	28120	566	2	[1]
Aquilegia coerulea	https://phytozome.jgi.doe.gov/pz/portal.html#!info?alias=Org_Acoerulea	v3.1	30023	292	44	[2]
Nelumbo nucifera	https://bioinformatics.psb.ugent.be/plaza/versions/plaza_v4_dicots/download/index	v1.1	26685	929	3	[3]
Vitis vinifera	https://phytozome.jgi.doe.gov	Genoscope.12X	26346	486	23	[4]
Populus trichocarpa	https://phytozome.jgi.doe.gov	v3.0	41335	423	20	[5]
Glycine max	https://phytozome.jgi.doe.gov	v1.0	56044	978	49	[6]
Citrus grandis	http://citrus.hzau.edu.cn/orange/	version1	30123	346	40	[7]
Theobroma cacao	https://phytozome.jgi.doe.gov	v1.1	29452	346	34	[8]
Arabidopsis thaliana	https://phytozome.jgi.doe.gov	TAIR10	27416	135	22	[9]
Eucalyptus grandis	https://phytozome.jgi.doe.gov	v2.0	36349	691	58	[10, 11]
Solanum lycopersicum	https://phytozome.jgi.doe.gov	ITAG2.4	34725	823	66	[12]
Coffea canephora	http://coffee-genome.org/	-	25574	569	38	[13]
Helianthus annuus	ftp://ftp.ncbi.nlm.nih.gov/genomes/all/GCF/002/127/325/GCF_002127325.1_HanXRQr1.0	HanXRQr1.0	73728	3000	178	[14]
Oryza sativa	https://phytozome.jgi.doe.gov	MSU_v7.0	42189	372	29	[15]

-: data no available.

1. Dohm JC, Minoche AE, Holtgrawe D, Capella-Gutierrez S, Zakrzewski F, Tafer H, et al. The genome of the recently domesticated crop plant sugar beet (*Beta vulgaris*). Nature. 2013; doi:10.1038/nature12817.
2. Filiault D, Ballerini E, Mandakova T, Akoz G, Derieg N, Schmutz J, et al. The *Aquilegia* genome: adaptive radiation and an extraordinarily polymorphic chromosome with a unique history. BioRxiv. 2018; doi:10.1101/264101.
3. Ming R, VanBuren R, Liu Y, Yang M, Han Y, Li L-T, et al. Genome of the long-living sacred lotus (*Nelumbo nucifera* Gaertn.). Genome Biology. 2013;14 5:R41. doi:10.1186/gb-2013-14-5-r41.
4. The French–Italian Public Consortium for Grapevine Genome Characterization. The grapevine genome sequence suggests ancestral hexaploidization in major angiosperm phyla. Nature. 2007;449:463. doi:10.1038/nature06148.
5. Tuskan GA, Difazio S, Jansson S, Bohlmann J, Grigoriev I, Hellsten U, et al. The genome of black cottonwood, *Populus trichocarpa* (Torr. & Gray). Science. 2006;313 5793:1596-604. doi:10.1126/science.1128691.
6. Schmutz J, Cannon SB, Schlueter J, Ma J, Mitros T, Nelson W, et al. Genome sequence of the palaeopolyploid soybean. Nature. 2010;463 7278:178-83. doi:10.1038/nature08670.
7. Wang X, Xu Y, Zhang S, Cao L, Huang Y, Cheng J, et al. Genomic analyses of primitive, wild and cultivated citrus provide insights into asexual reproduction. Nature Genetics. 2017;49:765. doi:10.1038/ng.3839.
8. Motamayor JC, Mockaitis K, Schmutz J, Haiminen N, Iii DL, Cornejo O, et al. The genome sequence of the most widely cultivated cacao type and its use to identify candidate genes regulating pod color. Genome Biology. 2013;14 6:r53. doi:10.1186/gb-2013-14-6-r53.
9. Cheng CY, Krishnakumar V, Chan AP, Thibaud-Nissen F, Schobel S and Town CD. Araport11: a complete reannotation of the *Arabidopsis thaliana* reference genome. Plant Journal. 2017;89 4:789-804. doi:10.1111/tpj.13415.
10. Myburg AA, Grattapaglia D, Tuskan GA, Hellsten U, Hayes RD, Grimwood J, et al. The genome of *Eucalyptus grandis*. Nature. 2014. doi:10.1038/nature13308.
11. Bartholome J, Mandrou E, Mabiala A, Jenkins J, Nabihoudine I, Klopp C, et al. High-resolution genetic maps of Eucalyptus improve *Eucalyptus grandis* genome assembly. New phytologist. 2015;206 4:1283-96. doi:10.1111/nph.13150.
12. Tomato Genome Consortium. The tomato genome sequence provides insights into fleshy fruit evolution. Nature. 2012;485 7400:635-41.
13. Denoeud F, Carretero-Paulet L, Dereeper A, Droc G, Guyot R, Pietrella M, et al. The coffee genome provides insight into the convergent evolution of caffeine biosynthesis. Science. 2014;345 6201:1181-4. doi:10.1126/science.1255274.
14. Badouin H, Gouzy J, Grassa CJ, Murat F, Staton SE, Cottret L, et al. The sunflower genome provides insights into oil metabolism, flowering

and Asterid evolution. Nature. 2017;546:148. doi:10.1038/nature22380.

15. Ouyang S, Zhu W, Hamilton J, Lin H, Campbell M, Childs K, et al. The TIGR Rice Genome Annotation Resource: improvements and new features. Nucleic Acids Research. 2007;35 Database issue:D883-7. doi:10.1093/nar/gkl976.

 Click here to download Table Table S14.xlsx

Species	Unique groups	Unique genes	Single-copy groups
Aquilegia coerulea	963	3,413	9,457
Arabidopsis thaliana	677	2,426	8,136
Beta vulgaris	497	2,261	9,638
Citrus grandis	967	4,639	10,351
Coffea canephora	527	1,681	10,199
Eucalyptus grandis	757	2,666	8,943
Glycine max	1,615	4,525	2,532
Helianthus annuus	1,538	10,629	6,230
Malania oleifera	520	2,097	9,329
Nelumbo nucifera	420	1,267	7,369
Oryza sativa	2,017	9,688	7,599
Populus trichocarpa	809	2,418	5,877
Solanum lycopersicum	905	3,947	9,052
Theobroma cacao	510	2,189	10,901
Vitis vinifera	563	1,525	9,651

Duplicated groups	Duplicated genes
4,042	14,514
4,704	15,322
2,735	12,197
3,655	14,306
3,088	10,728
4,524	19,975
12,500	43,778
7,767	46,852
2,624	10,568
4,725	12,733
5,452	21,302
8,695	27,601
4,902	16,916
3,409	12,462
3,203	10,000

Table

Click here to download Table Table S15.xlsx

Category	P_value	Q_value	numDEInCat	numInCat	Term
GO:0050468	5.79E-36	5.03E-32	28	28	reticuline oxidase activ
GO:0009410	1.49E-31	6.47E-28	39	66	response to xenobiotic
GO:0009741	3.61E-26	1.05E-22	42	99	response to brassinoste
GO:0008762	2.77E-25	6.02E-22	31	53	UDP-N-acetylmuramat
GO:0080156	6.70E-22	1.16E-18	37	92	mitochondrial mRNA r
GO:1900865	2.61E-21	3.78E-18	28	52	chloroplast RNA modi
GO:0016023	1.75E-19	2.17E-16	27	55	cytoplasmic membrane
GO:0009880	3.00E-18	3.26E-15	27	59	embryonic pattern spec
GO:0031425	1.25E-17	1.21E-14	25	52	chloroplast RNA proce
GO:0000963	5.15E-17	4.17E-14	19	29	mitochondrial RNA pr
GO:0050660	5.29E-17	4.17E-14	39	137	flavin adenine dinuclec
GO:0005739	3.52E-16	2.55E-13	193	1,830	mitochondrion
GO:0090404	8.87E-16	5.93E-13	17	25	pollen tube tip
GO:0043680	1.33E-15	8.24E-13	12	12	filiform apparatus
GO:0009826	4.21E-15	2.44E-12	43	186	unidimensional cell gro
GO:0010200	1.90E-14	1.03E-11	57	322	response to chitin
GO:0009820	2.54E-14	1.30E-11	25	69	alkaloid metabolic proc
GO:0016554	8.41E-14	4.06E-11	22	54	cytidine to uridine editi
GO:0007338	1.98E-13	9.08E-11	18	36	single fertilization
GO:0009451	1.96E-12	8.51E-10	23	68	RNA modification
GO:0010483	2.31E-12	9.56E-10	12	16	pollen tube reception
GO:0006814	6.98E-11	2.76E-08	17	42	sodium ion transport
GO:0005576	5.20E-10	1.96E-07	97	881	extracellular region
GO:0018685	5.83E-10	2.11E-07	12	22	alkane 1-monooxygena
GO:0016131	7.37E-10	2.56E-07	10	15	brassinosteroid metabo
GO:0043531	1.03E-09	3.45E-07	32	161	ADP binding
GO:0016998	1.53E-09	4.94E-07	12	24	cell wall macromolecul
GO:0005451	1.67E-09	5.17E-07	11	19	monovalent cation:prot
GO:0004058	1.86E-09	5.38E-07	7	7	aromatic-L-amino-acid
GO:0004837	1.86E-09	5.38E-07	7	7	tyrosine decarboxylase
GO:0046029	2.03E-09	5.69E-07	10	16	mannitol dehydrogenas
GO:0009835	2.56E-09	6.94E-07	20	72	fruit ripening
GO:0010248	2.80E-09	7.37E-07	10	16	establishment or maint
GO:0006952	4.11E-09	1.05E-06	78	678	defense response
GO:0016491	4.41E-09	1.09E-06	59	447	oxidoreductase activity
GO:0006355	4.63E-09	1.12E-06	145	1,552	regulation of transcript
GO:0006397	5.12E-09	1.20E-06	54	390	mRNA processing
GO:0003825	5.57E-09	1.27E-06	9	13	alpha,alpha-trehalose-p
GO:0006351	7.48E-09	1.67E-06	158	1,745	transcription, DNA-ten
GO:0005773	1.18E-08	2.55E-06	84	757	vacuole
GO:0016705	1.21E-08	2.57E-06	40	260	oxidoreductase activity
GO:0048364	2.52E-08	5.21E-06	37	230	root development
GO:0047924	4.12E-08	8.33E-06	6	6	geraniol dehydrogenase
GO:0009607	4.24E-08	8.38E-06	21	93	response to biotic stimu
GO:0010051	4.96E-08	9.58E-06	23	107	xylem and phloem patt
GO:0009678	5.70E-08	1.08E-05	10	20	hydrogen-translocating
GO:0031225	8.39E-08	1.55E-05	28	156	anchored component of

GO:0009733	9.76E-08	1.77E-05	43	307	response to auxin
GO:0045851	1.44E-07	2.55E-05	9	17	pH reduction
GO:0004497	1.80E-07	3.08E-05	40	283	monooxygenase activit
GO:0016324	1.81E-07	3.08E-05	14	45	apical plasma membrar
GO:0004805	2.75E-07	4.60E-05	9	18	trehalose-phosphatase a
GO:0043231	2.92E-07	4.79E-05	34	225	intracellular membrane
GO:0009507	4.80E-07	7.71E-05	188	2,265	chloroplast
GO:0006885	5.08E-07	8.03E-05	10	24	regulation of pH
GO:0033946	5.19E-07	8.05E-05	8	15	xyloglucan-specific enc
GO:0004427	6.79E-07	0.000103462	10	25	inorganic diphosphatas
GO:0009734	7.82E-07	0.000117122	37	264	auxin-activated signali
GO:0006259	1.24E-06	0.000182747	8	16	DNA metabolic proces
GO:0042761	1.69E-06	0.000244132	11	33	very long-chain fatty ac
GO:0030308	1.76E-06	0.000250647	11	34	negative regulation of c
GO:0070330	1.89E-06	0.000264856	9	22	aromatase activity
GO:0052597	3.06E-06	0.000415131	7	13	diamine oxidase activit
GO:0097185	3.06E-06	0.000415131	7	13	cellular response to azi
GO:0042178	3.55E-06	0.000474293	5	6	xenobiotic catabolic pr
GO:0004888	3.74E-06	0.000492131	9	23	transmembrane signali
GO:0008061	3.87E-06	0.000496058	12	43	chitin binding
GO:0005992	3.88E-06	0.000496058	10	29	trehalose biosynthetic p
GO:0004568	4.04E-06	0.000501773	12	43	chitinase activity
GO:0006032	4.04E-06	0.000501773	12	43	chitin catabolic process
GO:0005829	4.94E-06	0.00060477	189	2,333	cytosol
GO:0052593	5.94E-06	0.000688325	7	14	tryptamine:oxygen oxic
GO:0052594	5.94E-06	0.000688325	7	14	aminoacetone:oxygen c
GO:0052595	5.94E-06	0.000688325	7	14	aliphatic-amine oxidase
GO:0052596	5.94E-06	0.000688325	7	14	phenethylamine:oxyger
GO:0006809	6.18E-06	0.000706331	7	14	nitric oxide biosyntheti
GO:0006813	7.08E-06	0.00079843	14	59	potassium ion transport
GO:0031426	7.47E-06	0.000824013	6	10	polycistronic mRNA pr
GO:0045551	7.59E-06	0.000824013	8	20	cinnamyl-alcohol dehy
GO:0052747	7.59E-06	0.000824013	8	20	sinapyl alcohol dehydr
GO:0003700	7.82E-06	0.00083843	105	1,185	transcription factor acti
GO:0007165	1.01E-05	0.001067572	29	202	signal transduction
GO:0003849	1.40E-05	0.001469137	4	4	3-deoxy-7-phosphohep
GO:0008131	1.74E-05	0.001799307	7	16	primary amine oxidase
GO:0080110	2.09E-05	0.002140955	8	22	sporopollenin biosynth
GO:0005789	2.38E-05	0.002407312	61	593	endoplasmic reticulum
GO:0000145	2.54E-05	0.002541337	9	29	exocyst
GO:0046835	2.87E-05	0.002831045	8	22	carbohydrate phosphory
GO:0016831	4.09E-05	0.003993684	7	18	carboxy-lyase activity
GO:0030007	4.31E-05	0.004163037	7	18	cellular potassium ion l
GO:0046527	4.94E-05	0.004714724	7	19	glucosyltransferase acti
GO:0003872	5.68E-05	0.005365185	6	13	6-phosphofructokinase
GO:0009926	6.46E-05	0.006039303	15	80	auxin polar transport
GO:0071398	6.88E-05	0.006360158	5	9	cellular response to fatt
GO:0080043	7.51E-05	0.006773208	18	115	quercetin 3-O-glucosyl

GO:0080044	7.51E-05	0.006773208	18	115	quercetin 7-O-glucosyl
GO:0047196	7.56E-05	0.006773208	5	9	long-chain-alcohol O-fa
GO:0010337	8.30E-05	0.007355518	7	20	regulation of salicylic a
GO:0052696	8.47E-05	0.007405402	18	116	flavonoid glucuronidati
GO:0009522	8.52E-05	0.007405402	9	34	photosystem I
GO:0009791	8.94E-05	0.007690702	13	65	post-embryonic develop
GO:0006002	9.53E-05	0.008117931	6	14	fructose 6-phosphate m
GO:0030104	0.000122816	0.010359461	5	10	water homeostasis
GO:0042349	0.000156675	0.012820277	4	6	guiding stereospecific s
GO:1901599	0.000156675	0.012820277	4	6	(-)-pinoresinol biosynth
GO:0080046	0.000157322	0.012820277	7	22	quercetin 4'-O-glucosyl
GO:0006979	0.000157892	0.012820277	37	333	response to oxidative st
GO:0004040	0.000161658	0.01300446	5	10	amidase activity
GO:0000272	0.000166335	0.01325793	12	60	polysaccharide catabol
GO:0043078	0.000171335	0.013532329	9	37	polar nucleus
GO:0045892	0.00017474	0.013676921	30	251	negative regulation of t
GO:0033521	0.000179461	0.013797856	3	3	phytyl diphosphate bios
GO:0045550	0.000179461	0.013797856	3	3	geranylgeranyl reductas
GO:0006073	0.00018503	0.013978648	9	37	cellular glucan metabol
GO:0016762	0.00018503	0.013978648	9	37	xyloglucan:xyloglucosy
GO:0004853	0.000188733	0.014135426	4	6	uroporphyrinogen deca
GO:0006721	0.000195864	0.014331396	3	3	terpenoid metabolic pro
GO:0052582	0.000195864	0.014331396	3	3	(+)-menthofuran syntha
GO:0080166	0.000196298	0.014331396	3	3	stomium development
GO:0008395	0.000205507	0.01487873	3	3	steroid hydroxylase act
GO:0050505	0.000223693	0.016008119	5	11	hydroquinone glucosyl
GO:0048046	0.000224792	0.016008119	57	597	apoplast
GO:0051365	0.00023022	0.016206043	5	11	cellular response to pot
GO:0009259	0.000232922	0.016206043	3	3	ribonucleotide metabol
GO:0031520	0.000236898	0.016206043	3	3	plasma membrane of ce
GO:0052866	0.000236898	0.016206043	3	3	phosphatidylinositol ph
GO:0008840	0.000236898	0.016206043	3	3	4-hydroxy-tetrahydrodi
GO:0050645	0.00026055	0.017684846	4	7	limonoid glucosyltrans
GO:0050502	0.000270991	0.018250908	7	24	cis-zeatin O-beta-D-glu
GO:0009308	0.000283046	0.018916156	7	23	amine metabolic proces
GO:0020037	0.000289949	0.019229612	40	383	heme binding
GO:0005506	0.00030931	0.020358218	40	383	iron ion binding
GO:0004185	0.000325014	0.021231013	13	72	serine-type carboxypep
GO:0010053	0.000352996	0.02288677	9	38	root epidermal cell diff
GO:0010345	0.000408846	0.026311497	9	40	suberin biosynthetic pro
GO:0035197	0.000448506	0.028651593	5	12	siRNA binding
GO:0045893	0.000516139	0.032731503	53	561	positive regulation of tr
GO:0071732	0.000525987	0.0331143	8	33	cellular response to nitr
GO:0009626	0.00059718	0.037325914	18	128	plant-type hypersensitiv
GO:0016773	0.000611046	0.037772357	9	41	phosphotransferase acti
GO:0009612	0.000613018	0.037772357	7	26	response to mechanical
GO:0047746	0.000667838	0.040860429	3	4	chlorophyllase activity
GO:0016765	0.000712052	0.043260914	4	8	transferase activity, tran

GO:0050403	0.000734456	0.044312195	7	28	trans-zeatin O-beta-D-g
GO:0055129	0.000741541	0.044431119	4	8	L-proline biosynthetic
GO:0050267	0.000756268	0.045003148	3	4	rubber cis-polyprenylci
GO:0003984	0.000773227	0.045699315	3	4	acetolactate synthase a
GO:0010266	0.000788315	0.046276246	3	4	response to vitamin B1
GO:0046522	0.000803231	0.046835401	3	4	S-methyl-5-thioribose k
GO:0019859	0.000815905	0.046986717	3	4	thymine metabolic proc
GO:0030955	0.000816643	0.046986717	6	20	potassium ion binding
GO:0043812	0.00088542	0.049704368	3	4	phosphatidylinositol-4-
GO:0004318	0.000890431	0.049704368	3	4	enoyl-[acyl-carrier-prot
GO:0005835	0.000890431	0.049704368	3	4	fatty acid synthase com
GO:0016631	0.000890431	0.049704368	3	4	enoyl-[acyl-carrier-prot
GO:0005971	0.000892482	0.049704368	3	4	ribonucleoside-diphosp
GO:0006805	0.000902109	0.049920517	5	14	xenobiotic metabolic p

Class	Gene ID
molecular_function	Maole_000947.T1, Maole_000949.T1, Maole_000952.T1, Maole_0009[illegible]
biological_process	Maole_005120.T1, Maole_005178.T1, Maole_005179.T1, Maole_0051[illegible]
biological_process	Maole_002978.T1, Maole_003404.T1, Maole_003405.T1, Maole_0034[illegible]
molecular_function	Maole_000947.T1, Maole_000949.T1, Maole_000952.T1, Maole_0009[illegible]
biological_process	Maole_000261.T1, Maole_001054.T1, Maole_002026.T1, Maole_0021[illegible]
biological_process	Maole_003493.T1, Maole_004679.T1, Maole_005107.T1, Maole_0052[illegible]
cellular_component	Maole_000947.T1, Maole_000949.T1, Maole_000952.T1, Maole_0009[illegible]
biological_process	Maole_000378.T1, Maole_000437.T1, Maole_000677.T1, Maole_0014[illegible]
biological_process	Maole_000089.T1, Maole_001995.T1, Maole_004679.T1, Maole_0056[illegible]
biological_process	Maole_001054.T1, Maole_005145.T1, Maole_005991.T1, Maole_0062[illegible]
molecular_function	Maole_000947.T1, Maole_000949.T1, Maole_000952.T1, Maole_0009[illegible]
cellular_component	Maole_000261.T1, Maole_000438.T1, Maole_000439.T1, Maole_0008[illegible]
cellular_component	Maole_005499.T1, Maole_007741.T1, Maole_009345.T1, Maole_0113[illegible]
cellular_component	Maole_003404.T1, Maole_003485.T1, Maole_005499.T1, Maole_0147[illegible]
biological_process	Maole_000015.T1, Maole_001212.T1, Maole_001213.T1, Maole_0014[illegible]
biological_process	Maole_000378.T1, Maole_002027.T1, Maole_003161.T1, Maole_0051[illegible]
biological_process	Maole_000947.T1, Maole_000949.T1, Maole_000952.T1, Maole_0009[illegible]
biological_process	Maole_001054.T1, Maole_002538.T1, Maole_005087.T1, Maole_0051[illegible]
biological_process	Maole_005499.T1, Maole_007741.T1, Maole_011302.T1, Maole_0125[illegible]
biological_process	Maole_002026.T1, Maole_005087.T1, Maole_005107.T1, Maole_0065[illegible]
biological_process	Maole_003404.T1, Maole_003485.T1, Maole_005499.T1, Maole_0147[illegible]
biological_process	Maole_003069.T1, Maole_003305.T1, Maole_003945.T1, Maole_0075[illegible]
cellular_component	Maole_000596.T1, Maole_002245.T1, Maole_002247.T1, Maole_0022[illegible]
molecular_function	Maole_002169.T1, Maole_002172.T1, Maole_002180.T1, Maole_0021[illegible]
biological_process	Maole_004856.T1, Maole_005111.T1, Maole_012033.T1, Maole_0120[illegible]
molecular_function	Maole_000688.T1, Maole_000715.T1, Maole_000726.T1, Maole_0007[illegible]
biological_process	Maole_020579.T1, Maole_023238.T1, Maole_023239.T1, Maole_0232[illegible]
molecular_function	Maole_007592.T1, Maole_007594.T1, Maole_007595.T1, Maole_0075[illegible]
molecular_function	Maole_021238.T1, Maole_023059.T1, Maole_023061.T1, Maole_0230[illegible]
molecular_function	Maole_021238.T1, Maole_023059.T1, Maole_023061.T1, Maole_0230[illegible]
molecular_function	Maole_005882.T1, Maole_005883.T1, Maole_005885.T1, Maole_0058[illegible]
biological_process	Maole_002319.T1, Maole_002322.T1, Maole_002329.T1, Maole_0023[illegible]
biological_process	Maole_003069.T1, Maole_003305.T1, Maole_003945.T1, Maole_0065[illegible]
biological_process	Maole_000688.T1, Maole_000715.T1, Maole_000726.T1, Maole_0007[illegible]
molecular_function	Maole_000212.T1, Maole_000369.T1, Maole_001766.T1, Maole_0020[illegible]
biological_process	Maole_000003.T1, Maole_000378.T1, Maole_000437.T1, Maole_0006[illegible]
biological_process	Maole_001611.T1, Maole_002269.T1, Maole_003493.T1, Maole_0046[illegible]
molecular_function	Maole_001031.T1, Maole_006121.T1, Maole_006541.T1, Maole_0151[illegible]
biological_process	Maole_000003.T1, Maole_000378.T1, Maole_000437.T1, Maole_0006[illegible]
cellular_component	Maole_000947.T1, Maole_000949.T1, Maole_000952.T1, Maole_0009[illegible]
molecular_function	Maole_002169.T1, Maole_002172.T1, Maole_002180.T1, Maole_0021[illegible]
biological_process	Maole_000378.T1, Maole_000437.T1, Maole_000677.T1, Maole_0014[illegible]
molecular_function	Maole_005886.T1, Maole_005890.T1, Maole_005893.T1, Maole_0058[illegible]
biological_process	Maole_012895.T1, Maole_012897.T1, Maole_019956.T1, Maole_0199[illegible]
biological_process	Maole_000378.T1, Maole_000437.T1, Maole_000677.T1, Maole_0014[illegible]
molecular_function	Maole_003069.T1, Maole_003305.T1, Maole_003945.T1, Maole_0065[illegible]
cellular_component	Maole_000947.T1, Maole_000949.T1, Maole_000952.T1, Maole_0009[illegible]

biological_process	Maole_000378.T1, Maole_000437.T1, Maole_000677.T1, Maole_0014
biological_process	Maole_001461.T1, Maole_004639.T1, Maole_015166.T1, Maole_0225
molecular_function	Maole_002169.T1, Maole_002172.T1, Maole_002180.T1, Maole_0021
cellular_component	Maole_005499.T1, Maole_007741.T1, Maole_011302.T1, Maole_0147
molecular_function	Maole_001031.T1, Maole_006121.T1, Maole_006541.T1, Maole_0151
cellular_component	Maole_002169.T1, Maole_002172.T1, Maole_002319.T1, Maole_0023
cellular_component	Maole_000089.T1, Maole_000156.T1, Maole_000158.T1, Maole_0005
biological_process	Maole_007592.T1, Maole_009013.T1, Maole_013459.T1, Maole_0142
molecular_function	Maole_002978.T1, Maole_003782.T1, Maole_003783.T1, Maole_0037
molecular_function	Maole_003069.T1, Maole_003305.T1, Maole_003945.T1, Maole_0065
biological_process	Maole_000378.T1, Maole_000437.T1, Maole_000677.T1, Maole_0014
biological_process	Maole_000788.T1, Maole_000790.T1, Maole_006224.T1, Maole_0062
biological_process	Maole_002180.T1, Maole_002181.T1, Maole_002183.T1, Maole_0052
biological_process	Maole_000439.T1, Maole_005499.T1, Maole_014799.T1, Maole_0176
molecular_function	Maole_002180.T1, Maole_002181.T1, Maole_002183.T1, Maole_0052
molecular_function	Maole_001779.T1, Maole_001782.T1, Maole_001785.T1, Maole_0160
biological_process	Maole_001779.T1, Maole_001782.T1, Maole_001785.T1, Maole_0160
biological_process	Maole_004561.T1, Maole_013805.T1, Maole_013806.T1, Maole_0138
molecular_function	Maole_001461.T1, Maole_004639.T1, Maole_015166.T1, Maole_0225
molecular_function	Maole_020579.T1, Maole_023238.T1, Maole_023239.T1, Maole_0232
biological_process	Maole_001031.T1, Maole_006121.T1, Maole_006541.T1, Maole_0068
molecular_function	Maole_020579.T1, Maole_023238.T1, Maole_023239.T1, Maole_0232
biological_process	Maole_020579.T1, Maole_023238.T1, Maole_023239.T1, Maole_0232
cellular_component	Maole_000015.T1, Maole_000369.T1, Maole_000596.T1, Maole_0009
molecular_function	Maole_001779.T1, Maole_001782.T1, Maole_001785.T1, Maole_0160
molecular_function	Maole_001779.T1, Maole_001782.T1, Maole_001785.T1, Maole_0160
molecular_function	Maole_001779.T1, Maole_001782.T1, Maole_001785.T1, Maole_0160
molecular_function	Maole_001779.T1, Maole_001782.T1, Maole_001785.T1, Maole_0160
biological_process	Maole_001779.T1, Maole_001782.T1, Maole_001785.T1, Maole_0160
biological_process	Maole_007592.T1, Maole_007594.T1, Maole_007595.T1, Maole_0075
biological_process	Maole_000089.T1, Maole_001995.T1, Maole_005631.T1, Maole_0059
molecular_function	Maole_005882.T1, Maole_005883.T1, Maole_005885.T1, Maole_0058
molecular_function	Maole_005882.T1, Maole_005883.T1, Maole_005885.T1, Maole_0058
molecular_function	Maole_000003.T1, Maole_000378.T1, Maole_000437.T1, Maole_0006
biological_process	Maole_003404.T1, Maole_003483.T1, Maole_003485.T1, Maole_0034
molecular_function	Maole_001279.T1, Maole_007364.T1, Maole_018830.T1, Maole_0193
molecular_function	Maole_001779.T1, Maole_001782.T1, Maole_001785.T1, Maole_0160
biological_process	Maole_002180.T1, Maole_002183.T1, Maole_005259.T1, Maole_0077
cellular_component	Maole_002172.T1, Maole_002180.T1, Maole_002181.T1, Maole_0021
cellular_component	Maole_000596.T1, Maole_002809.T1, Maole_004342.T1, Maole_0086
biological_process	Maole_000911.T1, Maole_001047.T1, Maole_008161.T1, Maole_0202
molecular_function	Maole_021238.T1, Maole_023059.T1, Maole_023061.T1, Maole_0230
biological_process	Maole_009013.T1, Maole_019829.T1, Maole_019870.T1, Maole_0204
molecular_function	Maole_005111.T1, Maole_012033.T1, Maole_012034.T1, Maole_0120
molecular_function	Maole_000911.T1, Maole_001047.T1, Maole_008161.T1, Maole_0205
biological_process	Maole_003052.T1, Maole_003069.T1, Maole_003305.T1, Maole_0038
biological_process	Maole_008096.T1, Maole_008097.T1, Maole_008098.T1, Maole_0081
molecular_function	Maole_004561.T1, Maole_004613.T1, Maole_004614.T1, Maole_0046

molecular_function	Maole_004561.T1, Maole_004613.T1, Maole_004614.T1, Maole_0046
molecular_function	Maole_010272.T1, Maole_010273.T1, Maole_010274.T1, Maole_0102
biological_process	Maole_002999.T1, Maole_003050.T1, Maole_012089.T1, Maole_0139
biological_process	Maole_004561.T1, Maole_004613.T1, Maole_004614.T1, Maole_0046
cellular_component	Maole_000212.T1, Maole_015203.T1, Maole_021341.T1, Maole_0213
biological_process	Maole_003404.T1, Maole_003485.T1, Maole_005499.T1, Maole_0097
biological_process	Maole_000911.T1, Maole_001047.T1, Maole_008161.T1, Maole_0205
biological_process	Maole_009013.T1, Maole_019829.T1, Maole_019870.T1, Maole_0204
molecular_function	Maole_019956.T1, Maole_019959.T1, Maole_019963.T1, Maole_0235
biological_process	Maole_019956.T1, Maole_019959.T1, Maole_019963.T1, Maole_0235
molecular_function	Maole_005111.T1, Maole_012033.T1, Maole_012034.T1, Maole_0120
biological_process	Maole_000947.T1, Maole_000949.T1, Maole_000952.T1, Maole_0009
molecular_function	Maole_003538.T1, Maole_003540.T1, Maole_010786.T1, Maole_0107
biological_process	Maole_020579.T1, Maole_023238.T1, Maole_023239.T1, Maole_0232
cellular_component	Maole_011635.T1, Maole_013907.T1, Maole_013908.T1, Maole_0139
biological_process	Maole_000378.T1, Maole_000437.T1, Maole_000439.T1, Maole_0006
biological_process	Maole_020036.T1, Maole_022028.T1, Maole_022954.T1
molecular_function	Maole_020036.T1, Maole_022028.T1, Maole_022954.T1
biological_process	Maole_002978.T1, Maole_003782.T1, Maole_003783.T1, Maole_0037
molecular_function	Maole_002978.T1, Maole_003782.T1, Maole_003783.T1, Maole_0037
molecular_function	Maole_002758.T1, Maole_010530.T1, Maole_010532.T1, Maole_0105
biological_process	Maole_002319.T1, Maole_023205.T1, Maole_023211.T1
molecular_function	Maole_002319.T1, Maole_023205.T1, Maole_023211.T1
biological_process	Maole_000003.T1, Maole_023892.T1, Maole_024107.T1
molecular_function	Maole_004856.T1, Maole_021133.T1, Maole_021135.T1
molecular_function	Maole_004561.T1, Maole_013805.T1, Maole_013806.T1, Maole_0138
cellular_component	Maole_000947.T1, Maole_000949.T1, Maole_000952.T1, Maole_0009
biological_process	Maole_008096.T1, Maole_008097.T1, Maole_008098.T1, Maole_0081
biological_process	Maole_000369.T1, Maole_001766.T1, Maole_015853.T1
cellular_component	Maole_009345.T1, Maole_012742.T1, Maole_022416.T1
molecular_function	Maole_009345.T1, Maole_012742.T1, Maole_022416.T1
molecular_function	Maole_004967.T1, Maole_016326.T1, Maole_016327.T1
molecular_function	Maole_005715.T1, Maole_015708.T1, Maole_015709.T1, Maole_0241
molecular_function	Maole_005111.T1, Maole_012033.T1, Maole_012034.T1, Maole_0120
biological_process	Maole_001779.T1, Maole_001782.T1, Maole_001785.T1, Maole_0160
molecular_function	Maole_002169.T1, Maole_002172.T1, Maole_002180.T1, Maole_0021
molecular_function	Maole_002169.T1, Maole_002172.T1, Maole_002180.T1, Maole_0021
molecular_function	Maole_005171.T1, Maole_007127.T1, Maole_009867.T1, Maole_0110
biological_process	Maole_000911.T1, Maole_001047.T1, Maole_007908.T1, Maole_0079
biological_process	Maole_002180.T1, Maole_002181.T1, Maole_002183.T1, Maole_0052
molecular_function	Maole_001429.T1, Maole_005194.T1, Maole_006987.T1, Maole_0069
biological_process	Maole_002030.T1, Maole_002032.T1, Maole_002805.T1, Maole_0038
biological_process	Maole_008096.T1, Maole_008097.T1, Maole_008098.T1, Maole_0081
biological_process	Maole_002245.T1, Maole_002247.T1, Maole_002253.T1, Maole_0031
molecular_function	Maole_008872.T1, Maole_010839.T1, Maole_010840.T1, Maole_0156
biological_process	Maole_002978.T1, Maole_003786.T1, Maole_003790.T1, Maole_0037
molecular_function	Maole_013425.T1, Maole_013426.T1, Maole_013428.T1
molecular_function	Maole_005614.T1, Maole_012177.T1, Maole_012178.T1, Maole_0157

molecular_function	Maole_005111.T1, Maole_012033.T1, Maole_012034.T1, Maole_0120[illegible]
biological_process	Maole_006145.T1, Maole_017439.T1, Maole_017441.T1, Maole_0235[illegible]
molecular_function	Maole_012177.T1, Maole_012178.T1, Maole_015768.T1
molecular_function	Maole_004372.T1, Maole_004375.T1, Maole_004377.T1
biological_process	Maole_023402.T1, Maole_023780.T1, Maole_023782.T1
molecular_function	Maole_015699.T1, Maole_015702.T1, Maole_015705.T1
biological_process	Maole_007877.T1, Maole_007878.T1, Maole_016334.T1
molecular_function	Maole_003069.T1, Maole_003305.T1, Maole_003945.T1, Maole_0082[illegible]
molecular_function	Maole_009345.T1, Maole_012742.T1, Maole_022416.T1
molecular_function	Maole_008716.T1, Maole_008717.T1, Maole_022203.T1
cellular_component	Maole_008716.T1, Maole_008717.T1, Maole_022203.T1
molecular_function	Maole_008716.T1, Maole_008717.T1, Maole_022203.T1
cellular_component	Maole_000369.T1, Maole_001766.T1, Maole_015853.T1
biological_process	Maole_004561.T1, Maole_013805.T1, Maole_013806.T1, Maole_0138[illegible]

53.T1, Maole_000954.T1, Maole_002491.T1, Maole_003004.T1, Maole_003005.T1, Maole_005
80.T1, Maole_005694.T1, Maole_005695.T1, Maole_005698.T1, Maole_005700.T1, Maole_005
81.T1, Maole_003483.T1, Maole_003485.T1, Maole_003488.T1, Maole_003786.T1, Maole_003
53.T1, Maole_000954.T1, Maole_002491.T1, Maole_003004.T1, Maole_003005.T1, Maole_003
00.T1, Maole_003493.T1, Maole_005145.T1, Maole_005341.T1, Maole_005836.T1, Maole_005
10.T1, Maole_006239.T1, Maole_006824.T1, Maole_007334.T1, Maole_007560.T1, Maole_007
53.T1, Maole_000954.T1, Maole_002491.T1, Maole_003004.T1, Maole_003005.T1, Maole_005
34.T1, Maole_002027.T1, Maole_003161.T1, Maole_003875.T1, Maole_004490.T1, Maole_004
31.T1, Maole_005991.T1, Maole_007389.T1, Maole_008518.T1, Maole_010002.T1, Maole_010
39.T1, Maole_006379.T1, Maole_006380.T1, Maole_006824.T1, Maole_009283.T1, Maole_012
53.T1, Maole_000954.T1, Maole_002491.T1, Maole_003004.T1, Maole_003005.T1, Maole_003
38.T1, Maole_000947.T1, Maole_000949.T1, Maole_000952.T1, Maole_000953.T1, Maole_000
02.T1, Maole_012742.T1, Maole_014799.T1, Maole_016769.T1, Maole_017626.T1, Maole_018
99.T1, Maole_017628.T1, Maole_018434.T1, Maole_020950.T1, Maole_020951.T1, Maole_020
61.T1, Maole_002229.T1, Maole_002311.T1, Maole_003124.T1, Maole_003404.T1, Maole_003
20.T1, Maole_005178.T1, Maole_005179.T1, Maole_005180.T1, Maole_005694.T1, Maole_005
53.T1, Maole_000954.T1, Maole_002491.T1, Maole_003004.T1, Maole_003005.T1, Maole_005
45.T1, Maole_005836.T1, Maole_005991.T1, Maole_006239.T1, Maole_006824.T1, Maole_009
75.T1, Maole_014799.T1, Maole_016769.T1, Maole_017626.T1, Maole_017628.T1, Maole_018
13.T1, Maole_007389.T1, Maole_008116.T1, Maole_008187.T1, Maole_008909.T1, Maole_012
99.T1, Maole_017628.T1, Maole_018434.T1, Maole_020950.T1, Maole_020951.T1, Maole_020
92.T1, Maole_007594.T1, Maole_007595.T1, Maole_007597.T1, Maole_008212.T1, Maole_009
48.T1, Maole_002249.T1, Maole_002250.T1, Maole_002253.T1, Maole_002254.T1, Maole_002
81.T1, Maole_002183.T1, Maole_005259.T1, Maole_007733.T1, Maole_015424.T1, Maole_015
34.T1, Maole_012036.T1, Maole_012037.T1, Maole_017285.T1, Maole_021133.T1, Maole_021
79.T1, Maole_000833.T1, Maole_001049.T1, Maole_001050.T1, Maole_003749.T1, Maole_003
41.T1, Maole_023344.T1, Maole_023347.T1, Maole_023983.T1, Maole_023998.T1, Maole_024
97.T1, Maole_009013.T1, Maole_013458.T1, Maole_013459.T1, Maole_014253.T1, Maole_016
63.T1, Maole_023064.T1, Maole_023690.T1, Maole_024007.T1
63.T1, Maole_023064.T1, Maole_023690.T1, Maole_024007.T1
86.T1, Maole_005890.T1, Maole_005893.T1, Maole_005894.T1, Maole_005895.T1, Maole_007
35.T1, Maole_002340.T1, Maole_002341.T1, Maole_002343.T1, Maole_008096.T1, Maole_008
32.T1, Maole_008212.T1, Maole_010105.T1, Maole_019117.T1, Maole_021175.T1, Maole_021
79.T1, Maole_000833.T1, Maole_001049.T1, Maole_001050.T1, Maole_003751.T1, Maole_005
38.T1, Maole_002039.T1, Maole_002040.T1, Maole_003774.T1, Maole_004053.T1, Maole_004
77.T1, Maole_001428.T1, Maole_001434.T1, Maole_001560.T1, Maole_001767.T1, Maole_002
79.T1, Maole_005107.T1, Maole_005210.T1, Maole_005631.T1, Maole_006239.T1, Maole_006
29.T1, Maole_017478.T1, Maole_017931.T1, Maole_018177.T1, Maole_019080.T1, Maole_019
77.T1, Maole_001428.T1, Maole_001429.T1, Maole_001434.T1, Maole_001560.T1, Maole_001
53.T1, Maole_000954.T1, Maole_002180.T1, Maole_002183.T1, Maole_003004.T1, Maole_003
81.T1, Maole_002183.T1, Maole_002318.T1, Maole_002319.T1, Maole_002320.T1, Maole_002
34.T1, Maole_002027.T1, Maole_003161.T1, Maole_003875.T1, Maole_004490.T1, Maole_004
94.T1, Maole_005895.T1, Maole_007887.T1
59.T1, Maole_019963.T1, Maole_020579.T1, Maole_023090.T1, Maole_023091.T1, Maole_023
34.T1, Maole_002027.T1, Maole_003161.T1, Maole_003875.T1, Maole_004490.T1, Maole_004
32.T1, Maole_008212.T1, Maole_010105.T1, Maole_019117.T1, Maole_021175.T1, Maole_021
53.T1, Maole_000954.T1, Maole_003004.T1, Maole_003005.T1, Maole_003774.T1, Maole_005

34.T1, Maole_001560.T1, Maole_002027.T1, Maole_002249.T1, Maole_002250.T1, Maole_002
00.T1, Maole_022504.T1, Maole_022507.T1, Maole_022510.T1, Maole_022511.T1, Maole_022
81.T1, Maole_002183.T1, Maole_002318.T1, Maole_002319.T1, Maole_002320.T1, Maole_002
99.T1, Maole_016769.T1, Maole_017626.T1, Maole_018434.T1, Maole_020950.T1, Maole_020
29.T1, Maole_017478.T1, Maole_017931.T1, Maole_018177.T1, Maole_019080.T1, Maole_019
41.T1, Maole_002342.T1, Maole_002343.T1, Maole_004561.T1, Maole_004613.T1, Maole_004
29.T1, Maole_000531.T1, Maole_000838.T1, Maole_000911.T1, Maole_001047.T1, Maole_001
53.T1, Maole_016516.T1, Maole_019829.T1, Maole_019870.T1, Maole_020460.T1, Maole_021
86.T1, Maole_003790.T1, Maole_003791.T1, Maole_003793.T1, Maole_019618.T1
32.T1, Maole_008212.T1, Maole_010105.T1, Maole_019117.T1, Maole_021175.T1, Maole_021
34.T1, Maole_001560.T1, Maole_002027.T1, Maole_003001.T1, Maole_003161.T1, Maole_003
26.T1, Maole_006227.T1, Maole_006262.T1, Maole_019907.T1, Maole_019908.T1
59.T1, Maole_007733.T1, Maole_015424.T1, Maole_015426.T1, Maole_015427.T1, Maole_015
28.T1, Maole_018434.T1, Maole_020950.T1, Maole_020951.T1, Maole_020952.T1, Maole_023
59.T1, Maole_007733.T1, Maole_015424.T1, Maole_015426.T1, Maole_015427.T1, Maole_019
84.T1, Maole_016085.T1, Maole_021078.T1, Maole_021081.T1
84.T1, Maole_016085.T1, Maole_021078.T1, Maole_021081.T1
07.T1, Maole_013808.T1
00.T1, Maole_022504.T1, Maole_022507.T1, Maole_022510.T1, Maole_022511.T1, Maole_022
41.T1, Maole_023344.T1, Maole_023347.T1, Maole_023983.T1, Maole_023998.T1, Maole_024
28.T1, Maole_015129.T1, Maole_017478.T1, Maole_017931.T1, Maole_018177.T1, Maole_019
41.T1, Maole_023344.T1, Maole_023347.T1, Maole_023983.T1, Maole_023998.T1, Maole_024
41.T1, Maole_023344.T1, Maole_023347.T1, Maole_023983.T1, Maole_023998.T1, Maole_024
11.T1, Maole_000947.T1, Maole_000949.T1, Maole_000952.T1, Maole_000953.T1, Maole_000
84.T1, Maole_016085.T1, Maole_021078.T1, Maole_021081.T1
84.T1, Maole_016085.T1, Maole_021078.T1, Maole_021081.T1
84.T1, Maole_016085.T1, Maole_021078.T1, Maole_021081.T1
84.T1, Maole_016085.T1, Maole_021078.T1, Maole_021081.T1
84.T1, Maole_016085.T1, Maole_021078.T1, Maole_021081.T1
97.T1, Maole_009013.T1, Maole_013458.T1, Maole_013459.T1, Maole_014253.T1, Maole_016
91.T1, Maole_010025.T1, Maole_022920.T1
90.T1, Maole_005893.T1, Maole_005895.T1, Maole_007887.T1, Maole_007888.T1
90.T1, Maole_005893.T1, Maole_005895.T1, Maole_007887.T1, Maole_007888.T1
77.T1, Maole_001428.T1, Maole_001434.T1, Maole_001560.T1, Maole_002027.T1, Maole_003
88.T1, Maole_005499.T1, Maole_007741.T1, Maole_009743.T1, Maole_009762.T1, Maole_010
05.T1
84.T1, Maole_016085.T1, Maole_021078.T1, Maole_021081.T1
33.T1, Maole_015426.T1, Maole_015427.T1, Maole_017521.T1, Maole_019116.T1
83.T1, Maole_002319.T1, Maole_002322.T1, Maole_002329.T1, Maole_002335.T1, Maole_002
73.T1, Maole_008917.T1, Maole_017045.T1, Maole_017791.T1, Maole_018969.T1, Maole_019
66.T1, Maole_020268.T1, Maole_020576.T1, Maole_022877.T1, Maole_023342.T1
63.T1, Maole_023064.T1, Maole_023690.T1, Maole_024007.T1
60.T1, Maole_021816.T1, Maole_022916.T1, Maole_022917.T1
36.T1, Maole_012037.T1, Maole_017285.T1, Maole_024088.T1
76.T1, Maole_022877.T1, Maole_023342.T1
34.T1, Maole_003836.T1, Maole_003945.T1, Maole_006532.T1, Maole_008212.T1, Maole_010
00.T1, Maole_008101.T1
15.T1, Maole_005111.T1, Maole_005712.T1, Maole_005715.T1, Maole_012033.T1, Maole_012

15.T1, Maole_005111.T1, Maole_005712.T1, Maole_005715.T1, Maole_012033.T1, Maole_012
76.T1, Maole_021480.T1
92.T1, Maole_013993.T1, Maole_013994.T1, Maole_014003.T1
15.T1, Maole_005111.T1, Maole_005712.T1, Maole_005715.T1, Maole_012033.T1, Maole_012
42.T1, Maole_022379.T1, Maole_023736.T1, Maole_023737.T1, Maole_023741.T1, Maole_023
43.T1, Maole_014799.T1, Maole_017628.T1, Maole_018434.T1, Maole_020950.T1, Maole_020
76.T1, Maole_022877.T1, Maole_023342.T1
60.T1, Maole_021816.T1
74.T1
74.T1
36.T1, Maole_012037.T1, Maole_017285.T1, Maole_024088.T1
53.T1, Maole_000954.T1, Maole_003004.T1, Maole_003005.T1, Maole_003774.T1, Maole_004
87.T1, Maole_010789.T1
41.T1, Maole_023344.T1, Maole_023347.T1, Maole_023983.T1, Maole_023998.T1, Maole_024
09.T1, Maole_013911.T1, Maole_013912.T1, Maole_013913.T1, Maole_013914.T1, Maole_020
77.T1, Maole_001434.T1, Maole_002027.T1, Maole_003161.T1, Maole_003875.T1, Maole_004

86.T1, Maole_003790.T1, Maole_003791.T1, Maole_003793.T1, Maole_003796.T1, Maole_019
86.T1, Maole_003790.T1, Maole_003791.T1, Maole_003793.T1, Maole_003796.T1, Maole_019
33.T1

07.T1, Maole_013808.T1
53.T1, Maole_000954.T1, Maole_002491.T1, Maole_002978.T1, Maole_002999.T1, Maole_003
00.T1, Maole_008101.T1

12.T1
36.T1, Maole_012037.T1, Maole_017285.T1, Maole_024088.T1
84.T1, Maole_016085.T1, Maole_021078.T1, Maole_021081.T1
81.T1, Maole_002183.T1, Maole_002318.T1, Maole_002319.T1, Maole_002320.T1, Maole_002
81.T1, Maole_002183.T1, Maole_002318.T1, Maole_002319.T1, Maole_002320.T1, Maole_002
61.T1, Maole_011062.T1, Maole_013627.T1, Maole_020845.T1, Maole_020847.T1, Maole_020
09.T1, Maole_008161.T1, Maole_017777.T1, Maole_020576.T1, Maole_022877.T1, Maole_023
59.T1, Maole_007733.T1, Maole_015424.T1, Maole_015426.T1, Maole_015427.T1, Maole_017
90.T1, Maole_021600.T1
08.T1, Maole_005120.T1, Maole_005178.T1, Maole_005179.T1, Maole_005180.T1, Maole_005
00.T1, Maole_008101.T1, Maole_015699.T1, Maole_015702.T1, Maole_015705.T1
97.T1, Maole_005149.T1, Maole_015936.T1, Maole_015986.T1, Maole_016870.T1, Maole_017
49.T1, Maole_020266.T1, Maole_020268.T1, Maole_023486.T1, Maole_023487.T1, Maole_023
91.T1, Maole_003793.T1, Maole_003796.T1, Maole_019618.T1

68.T1

36.T1, Maole_012037.T1, Maole_017285.T1, Maole_024088.T1
80.T1

12.T1, Maole_021175.T1, Maole_021594.T1

07.T1, Maole_013808.T1

495.T1, Maole_005948.T1, Maole_006013.T1, Maole_006014.T1, Maole_006015.T1, Maole_0
702.T1, Maole_007182.T1, Maole_007191.T1, Maole_007193.T1, Maole_007194.T1, Maole_0
790.T1, Maole_003791.T1, Maole_003793.T1, Maole_003796.T1, Maole_004175.T1, Maole_0
774.T1, Maole_005495.T1, Maole_005948.T1, Maole_006013.T1, Maole_006014.T1, Maole_0
991.T1, Maole_006239.T1, Maole_006824.T1, Maole_008187.T1, Maole_009283.T1, Maole_0
706.T1, Maole_009182.T1, Maole_009283.T1, Maole_009647.T1, Maole_010002.T1, Maole_0
495.T1, Maole_006013.T1, Maole_006014.T1, Maole_006015.T1, Maole_006016.T1, Maole_0
776.T1, Maole_005713.T1, Maole_007054.T1, Maole_009104.T1, Maole_009186.T1, Maole_0
025.T1, Maole_012936.T1, Maole_013071.T1, Maole_015433.T1, Maole_016034.T1, Maole_0
810.T1, Maole_012936.T1, Maole_016782.T1, Maole_020743.T1, Maole_021244.T1, Maole_0
774.T1, Maole_004372.T1, Maole_004375.T1, Maole_004377.T1, Maole_005495.T1, Maole_0
954.T1, Maole_001031.T1, Maole_001054.T1, Maole_001363.T1, Maole_001611.T1, Maole_0
434.T1, Maole_020950.T1, Maole_020951.T1, Maole_021052.T1, Maole_022416.T1, Maole_0
952.T1, Maole_023348.T1, Maole_023352.T1, Maole_023550.T1
405.T1, Maole_003481.T1, Maole_003483.T1, Maole_003485.T1, Maole_003488.T1, Maole_0
695.T1, Maole_005698.T1, Maole_005700.T1, Maole_005702.T1, Maole_007182.T1, Maole_0
495.T1, Maole_006013.T1, Maole_006014.T1, Maole_006015.T1, Maole_006016.T1, Maole_0
283.T1, Maole_012810.T1, Maole_012929.T1, Maole_012936.T1, Maole_016782.T1, Maole_0
434.T1, Maole_019745.T1, Maole_020950.T1, Maole_020951.T1, Maole_020952.T1, Maole_0
607.T1, Maole_015102.T1, Maole_016670.T1, Maole_016728.T1, Maole_016782.T1, Maole_0
952.T1, Maole_023348.T1, Maole_023352.T1, Maole_023550.T1
013.T1, Maole_013458.T1, Maole_013459.T1, Maole_014253.T1, Maole_016516.T1, Maole_0
311.T1, Maole_002312.T1, Maole_002491.T1, Maole_002809.T1, Maole_002999.T1, Maole_0
426.T1, Maole_015427.T1, Maole_017521.T1, Maole_019116.T1
135.T1, Maole_024088.T1
750.T1, Maole_003751.T1, Maole_003752.T1, Maole_005746.T1, Maole_005748.T1, Maole_0
019.T1, Maole_024051.T1, Maole_024069.T1, Maole_024098.T1
516.T1, Maole_019870.T1, Maole_022795.T1

887.T1, Maole_007888.T1
097.T1, Maole_008098.T1, Maole_008100.T1, Maole_008101.T1, Maole_016629.T1, Maole_0
178.T1, Maole_021594.T1
194.T1, Maole_005746.T1, Maole_005748.T1, Maole_006987.T1, Maole_007919.T1, Maole_0
056.T1, Maole_004057.T1, Maole_004058.T1, Maole_004207.T1, Maole_004208.T1, Maole_0
027.T1, Maole_003001.T1, Maole_003161.T1, Maole_003875.T1, Maole_004490.T1, Maole_0
379.T1, Maole_006380.T1, Maole_006824.T1, Maole_007032.T1, Maole_007334.T1, Maole_0
808.T1
767.T1, Maole_002027.T1, Maole_002301.T1, Maole_003001.T1, Maole_003161.T1, Maole_0
005.T1, Maole_003069.T1, Maole_003305.T1, Maole_003774.T1, Maole_003945.T1, Maole_0
321.T1, Maole_002322.T1, Maole_002329.T1, Maole_002335.T1, Maole_002340.T1, Maole_0
776.T1, Maole_005499.T1, Maole_005713.T1, Maole_007054.T1, Maole_009104.T1, Maole_0

241.T1, Maole_023344.T1, Maole_023347.T1, Maole_023400.T1, Maole_023402.T1, Maole_0
776.T1, Maole_005713.T1, Maole_007054.T1, Maole_009104.T1, Maole_009186.T1, Maole_0
178.T1, Maole_021594.T1
495.T1, Maole_006013.T1, Maole_006014.T1, Maole_006015.T1, Maole_006016.T1, Maole_0

2978.T1, Maole_003161.T1, Maole_003786.T1, Maole_003790.T1, Maole_003791.T1, Maole_0
2513.T1
2321.T1, Maole_002322.T1, Maole_002329.T1, Maole_002335.T1, Maole_002340.T1, Maole_0
0951.T1, Maole_021052.T1, Maole_023348.T1, Maole_023352.T1, Maole_023550.T1, Maole_0
0808.T1
4614.T1, Maole_004615.T1, Maole_005111.T1, Maole_005712.T1, Maole_005715.T1, Maole_0
1054.T1, Maole_001212.T1, Maole_001213.T1, Maole_001279.T1, Maole_001461.T1, Maole_0
1816.T1, Maole_022795.T1

1178.T1, Maole_021594.T1
3875.T1, Maole_004490.T1, Maole_004776.T1, Maole_005062.T1, Maole_005713.T1, Maole_0

5774.T1, Maole_017521.T1, Maole_017527.T1
3348.T1, Maole_023352.T1, Maole_023550.T1
0750.T1

2513.T1
4019.T1, Maole_024051.T1, Maole_024069.T1, Maole_024098.T1
0080.T1, Maole_019808.T1
4019.T1, Maole_024051.T1, Maole_024069.T1, Maole_024098.T1
4019.T1, Maole_024051.T1, Maole_024069.T1, Maole_024098.T1
0954.T1, Maole_001031.T1, Maole_001047.T1, Maole_001766.T1, Maole_001983.T1, Maole_0

5516.T1, Maole_019829.T1, Maole_019870.T1, Maole_020460.T1, Maole_021816.T1, Maole_0

3161.T1, Maole_003875.T1, Maole_004490.T1, Maole_004776.T1, Maole_005120.T1, Maole_0
0839.T1, Maole_011302.T1, Maole_011737.T1, Maole_011738.T1, Maole_014148.T1, Maole_0

2340.T1, Maole_002341.T1, Maole_002342.T1, Maole_002343.T1, Maole_004829.T1, Maole_0
0059.T1

0105.T1, Maole_017068.T1, Maole_019117.T1, Maole_021175.T1, Maole_021178.T1, Maole_0

2034.T1, Maole_012036.T1, Maole_012037.T1, Maole_013805.T1, Maole_013806.T1, Maole_0

2034.T1, Maole_012036.T1, Maole_012037.T1, Maole_013805.T1, Maole_013806.T1, Maole_0

2034.T1, Maole_012036.T1, Maole_012037.T1, Maole_013805.T1, Maole_013806.T1, Maole_0
3742.T1
0951.T1, Maole_020952.T1, Maole_023348.T1, Maole_023352.T1, Maole_023550.T1

4207.T1, Maole_004208.T1, Maole_005495.T1, Maole_006013.T1, Maole_006014.T1, Maole_0

4019.T1, Maole_024051.T1, Maole_024069.T1, Maole_024098.T1
0459.T1
4490.T1, Maole_004776.T1, Maole_005269.T1, Maole_005713.T1, Maole_007054.T1, Maole_0

0618.T1
0618.T1

3004.T1, Maole_003005.T1, Maole_003050.T1, Maole_003774.T1, Maole_003782.T1, Maole_0

2321.T1, Maole_002322.T1, Maole_002329.T1, Maole_002335.T1, Maole_002340.T1, Maole_0
2321.T1, Maole_002322.T1, Maole_002329.T1, Maole_002335.T1, Maole_002340.T1, Maole_0
0848.T1, Maole_020850.T1, Maole_022017.T1, Maole_022019.T1, Maole_022020.T1
3342.T1
7521.T1

5694.T1, Maole_005695.T1, Maole_005698.T1, Maole_005700.T1, Maole_005702.T1, Maole_0

7068.T1, Maole_017439.T1, Maole_017441.T1, Maole_020579.T1, Maole_023241.T1, Maole_0
3489.T1

006016.T1, Maole_006017.T1, Maole_009664.T1, Maole_009665.T1, Maole_009666.T1, Maole
007195.T1, Maole_007196.T1, Maole_007198.T1, Maole_007199.T1, Maole_007201.T1, Maole
004856.T1, Maole_005499.T1, Maole_007741.T1, Maole_008287.T1, Maole_008288.T1, Maole
006015.T1, Maole_006016.T1, Maole_006017.T1, Maole_008011.T1, Maole_009664.T1, Maole
009647.T1, Maole_010318.T1, Maole_012710.T1, Maole_012810.T1, Maole_012929.T1, Maole
010025.T1, Maole_010318.T1, Maole_011520.T1, Maole_012607.T1, Maole_012936.T1, Maole
006017.T1, Maole_009665.T1, Maole_009666.T1, Maole_009762.T1, Maole_010178.T1, Maole
010649.T1, Maole_012880.T1, Maole_013187.T1, Maole_014526.T1, Maole_015982.T1, Maole
016670.T1, Maole_018871.T1, Maole_018906.T1, Maole_020217.T1, Maole_021244.T1, Maole
021627.T1, Maole_021799.T1, Maole_021801.T1, Maole_022097.T1, Maole_022164.T1, Maole
005948.T1, Maole_006013.T1, Maole_006014.T1, Maole_006015.T1, Maole_006016.T1, Maole
001774.T1, Maole_001995.T1, Maole_002026.T1, Maole_002030.T1, Maole_002032.T1, Maole
023348.T1, Maole_023352.T1, Maole_023550.T1, Maole_024105.T1

004175.T1, Maole_004639.T1, Maole_006968.T1, Maole_007741.T1, Maole_008287.T1, Maole
007191.T1, Maole_007193.T1, Maole_007194.T1, Maole_007195.T1, Maole_007196.T1, Maole
006017.T1, Maole_009665.T1, Maole_009666.T1, Maole_010178.T1, Maole_010179.T1, Maole
018090.T1, Maole_020743.T1, Maole_021244.T1, Maole_021627.T1, Maole_021799.T1, Maole
021052.T1, Maole_023348.T1, Maole_023352.T1, Maole_023550.T1, Maole_024105.T1
017248.T1, Maole_017511.T1, Maole_020144.T1, Maole_020217.T1, Maole_020658.T1, Maole

019870.T1, Maole_021175.T1, Maole_021594.T1, Maole_022795.T1
003005.T1, Maole_003050.T1, Maole_003197.T1, Maole_004342.T1, Maole_004456.T1, Maole

007919.T1, Maole_008738.T1, Maole_012519.T1, Maole_012618.T1, Maole_012902.T1, Maole

016630.T1, Maole_016636.T1, Maole_023204.T1, Maole_023205.T1, Maole_023206.T1, Maole

008096.T1, Maole_008097.T1, Maole_008098.T1, Maole_008100.T1, Maole_008101.T1, Maole
005649.T1, Maole_005882.T1, Maole_005883.T1, Maole_005885.T1, Maole_005886.T1, Maole
004776.T1, Maole_005062.T1, Maole_005120.T1, Maole_005178.T1, Maole_005179.T1, Maole
007389.T1, Maole_007560.T1, Maole_007706.T1, Maole_008518.T1, Maole_009182.T1, Maole

003875.T1, Maole_004083.T1, Maole_004084.T1, Maole_004490.T1, Maole_004776.T1, Maole
005171.T1, Maole_005495.T1, Maole_006013.T1, Maole_006014.T1, Maole_006015.T1, Maole
002341.T1, Maole_002342.T1, Maole_002343.T1, Maole_004856.T1, Maole_005259.T1, Maole
009186.T1, Maole_010559.T1, Maole_010560.T1, Maole_010649.T1, Maole_012880.T1, Maole

023574.T1, Maole_023778.T1, Maole_023780.T1, Maole_023782.T1, Maole_023983.T1, Maole
010649.T1, Maole_012880.T1, Maole_013187.T1, Maole_014526.T1, Maole_018119.T1, Maole

006017.T1, Maole_007730.T1, Maole_009664.T1, Maole_009665.T1, Maole_009666.T1, Maole

03793.T1, Maole_003796.T1, Maole_003875.T1, Maole_004490.T1, Maole_004856.T1, Maole

02341.T1, Maole_002342.T1, Maole_002343.T1, Maole_004856.T1, Maole_005259.T1, Maole
24105.T1

08767.T1, Maole_008769.T1, Maole_011157.T1, Maole_011402.T1, Maole_012033.T1, Maole
01995.T1, Maole_002030.T1, Maole_002032.T1, Maole_002100.T1, Maole_002758.T1, Maole

07054.T1, Maole_007081.T1, Maole_009104.T1, Maole_009186.T1, Maole_010649.T1, Maole

01984.T1, Maole_002038.T1, Maole_002039.T1, Maole_002229.T1, Maole_002434.T1, Maole

22795.T1

05178.T1, Maole_005179.T1, Maole_005180.T1, Maole_005269.T1, Maole_005694.T1, Maole
14799.T1, Maole_016769.T1, Maole_017243.T1, Maole_017626.T1, Maole_017627.T1, Maole

05259.T1, Maole_005840.T1, Maole_005841.T1, Maole_007733.T1, Maole_007908.T1, Maole

21594.T1, Maole_024061.T1

13807.T1, Maole_013808.T1, Maole_017285.T1, Maole_024088.T1, Maole_024112.T1

13807.T1, Maole_013808.T1, Maole_017285.T1, Maole_024088.T1, Maole_024112.T1

13807.T1, Maole_013808.T1, Maole_017285.T1, Maole_024088.T1, Maole_024112.T1

06015.T1, Maole_006016.T1, Maole_006017.T1, Maole_006939.T1, Maole_007877.T1, Maole

08609.T1, Maole_009104.T1, Maole_009186.T1, Maole_010649.T1, Maole_012880.T1, Maole

03783.T1, Maole_003786.T1, Maole_003790.T1, Maole_003791.T1, Maole_003793.T1, Maole

02341.T1, Maole_002342.T1, Maole_002343.T1, Maole_004856.T1, Maole_005259.T1, Maole

02341.T1, Maole_002342.T1, Maole_002343.T1, Maole_004856.T1, Maole_005259.T1, Maole

06938.T1, Maole_007182.T1, Maole_007191.T1, Maole_007193.T1, Maole_007194.T1, Maole

23344.T1, Maole_023347.T1, Maole_023983.T1, Maole_024051.T1, Maole_024069.T1

_010178.T1, Maole_010179.T1, Maole_010180.T1, Maole_010181.T1, Maole_010184.T1, Mac
_007202.T1, Maole_007203.T1, Maole_007204.T1, Maole_007205.T1, Maole_007208.T1, Mac
_009743.T1, Maole_011302.T1, Maole_014799.T1, Maole_016769.T1, Maole_017243.T1, Mac
_009665.T1, Maole_009666.T1, Maole_010178.T1, Maole_010179.T1, Maole_010180.T1, Mac
_012936.T1, Maole_013143.T1, Maole_013537.T1, Maole_013876.T1, Maole_013981.T1, Mac
_013071.T1, Maole_016670.T1, Maole_016782.T1, Maole_018906.T1, Maole_020418.T1, Mac
_010179.T1, Maole_010180.T1, Maole_010181.T1, Maole_010531.T1, Maole_010533.T1, Mac
_015983.T1, Maole_018119.T1, Maole_018229.T1, Maole_018251.T1, Maole_019211.T1, Mac
_021626.T1, Maole_021627.T1, Maole_021897.T1, Maole_022097.T1, Maole_022164.T1, Mac
_023127.T1
_006017.T1, Maole_008011.T1, Maole_009664.T1, Maole_009665.T1, Maole_009666.T1, Mac
_002100.T1, Maole_002435.T1, Maole_002538.T1, Maole_002617.T1, Maole_003004.T1, Mac

_008288.T1, Maole_009743.T1, Maole_011302.T1, Maole_014331.T1, Maole_014799.T1, Mac
_007198.T1, Maole_007199.T1, Maole_007201.T1, Maole_007202.T1, Maole_007203.T1, Mac
_010180.T1, Maole_010181.T1, Maole_010531.T1, Maole_010533.T1, Maole_012412.T1, Mac
_021801.T1, Maole_022097.T1, Maole_022164.T1, Maole_023127.T1

_020874.T1, Maole_021574.T1, Maole_021799.T1, Maole_021801.T1, Maole_021897.T1

_005171.T1, Maole_005495.T1, Maole_005948.T1, Maole_005966.T1, Maole_005968.T1, Mac

_014131.T1, Maole_014132.T1, Maole_014133.T1, Maole_014134.T1, Maole_014135.T1, Mac

_023208.T1, Maole_023211.T1

_008287.T1, Maole_008288.T1, Maole_008738.T1, Maole_011014.T1, Maole_011817.T1, Mac
_005890.T1, Maole_005893.T1, Maole_005894.T1, Maole_005895.T1, Maole_006598.T1, Mac
_005180.T1, Maole_005266.T1, Maole_005269.T1, Maole_005694.T1, Maole_005695.T1, Mac
_009283.T1, Maole_009647.T1, Maole_010002.T1, Maole_010025.T1, Maole_010318.T1, Mac

_004968.T1, Maole_004969.T1, Maole_005062.T1, Maole_005120.T1, Maole_005178.T1, Mac
_006016.T1, Maole_006017.T1, Maole_006532.T1, Maole_007363.T1, Maole_008212.T1, Mac
_007733.T1, Maole_015424.T1, Maole_015426.T1, Maole_015427.T1, Maole_017521.T1, Mac
_013187.T1, Maole_013274.T1, Maole_014526.T1, Maole_014799.T1, Maole_017628.T1, Mac

_024051.T1, Maole_024069.T1, Maole_024102.T1
_018229.T1, Maole_018251.T1, Maole_019211.T1, Maole_019276.T1, Maole_023134.T1

_010178.T1, Maole_010179.T1, Maole_010180.T1, Maole_010181.T1, Maole_010184.T1, Mac

_005713.T1, Maole_007054.T1, Maole_007081.T1, Maole_009104.T1, Maole_009186.T1, Mac

_007733.T1, Maole_015424.T1, Maole_015426.T1, Maole_015427.T1, Maole_017521.T1, Mac

_012034.T1, Maole_012036.T1, Maole_012037.T1, Maole_013805.T1, Maole_013806.T1, Mac
_003052.T1, Maole_003069.T1, Maole_003079.T1, Maole_003305.T1, Maole_003363.T1, Mac

_012880.T1, Maole_013187.T1, Maole_014526.T1, Maole_015815.T1, Maole_015816.T1, Mac

_002435.T1, Maole_002758.T1, Maole_002809.T1, Maole_003004.T1, Maole_003005.T1, Mac

_005695.T1, Maole_005698.T1, Maole_005700.T1, Maole_005702.T1, Maole_005713.T1, Mac
_017628.T1, Maole_017630.T1, Maole_017632.T1, Maole_018434.T1, Maole_019907.T1, Mac

_007909.T1, Maole_009345.T1, Maole_010272.T1, Maole_010273.T1, Maole_010274.T1, Mac

_007878.T1, Maole_008537.T1, Maole_008974.T1, Maole_009664.T1, Maole_009665.T1, Mac

_013187.T1, Maole_013491.T1, Maole_013493.T1, Maole_014526.T1, Maole_014786.T1, Mac

_003796.T1, Maole_005495.T1, Maole_005948.T1, Maole_006013.T1, Maole_006014.T1, Mac

_007733.T1, Maole_015424.T1, Maole_015426.T1, Maole_015427.T1, Maole_017521.T1, Mac
_007733.T1, Maole_015424.T1, Maole_015426.T1, Maole_015427.T1, Maole_017521.T1, Mac

_007195.T1, Maole_007196.T1, Maole_007198.T1, Maole_007199.T1, Maole_007201.T1, Mac

ole_010531.T1, Maole_010533.T1, Maole_012412.T1, Maole_014489.T1, Maole_014652.T1
ole_007209.T1, Maole_007211.T1, Maole_007214.T1, Maole_009325.T1, Maole_009326.T1, M
ole_017439.T1, Maole_017441.T1, Maole_017626.T1, Maole_017627.T1, Maole_017628.T1, M
ole_010181.T1, Maole_010184.T1, Maole_010531.T1, Maole_010533.T1, Maole_012412.T1, M
ole_014503.T1, Maole_016782.T1, Maole_018093.T1, Maole_020743.T1, Maole_021152.T1, M
ole_020448.T1, Maole_020658.T1, Maole_021291.T1, Maole_022328.T1, Maole_023960.T1
ole_010839.T1, Maole_012412.T1, Maole_014489.T1, Maole_014652.T1
ole_019276.T1, Maole_021645.T1, Maole_023134.T1, Maole_023892.T1
ole_022328.T1, Maole_022920.T1

ole_010178.T1, Maole_010179.T1, Maole_010180.T1, Maole_010181.T1, Maole_010184.T1, M
ole_003005.T1, Maole_003069.T1, Maole_003305.T1, Maole_003405.T1, Maole_003493.T1, M

ole_015166.T1, Maole_015292.T1, Maole_016769.T1, Maole_017243.T1, Maole_017626.T1, M
ole_007205.T1, Maole_007206.T1, Maole_007208.T1, Maole_007209.T1, Maole_007211.T1, M
ole_014489.T1, Maole_014652.T1

ole_005969.T1, Maole_006013.T1, Maole_006014.T1, Maole_006015.T1, Maole_006016.T1, M

ole_014148.T1, Maole_014150.T1, Maole_016802.T1, Maole_016803.T1, Maole_016807.T1, M

ole_012083.T1, Maole_012519.T1, Maole_012618.T1, Maole_012895.T1, Maole_012897.T1, M
ole_006603.T1, Maole_006604.T1, Maole_007053.T1, Maole_007055.T1, Maole_007441.T1, M
ole_005698.T1, Maole_005700.T1, Maole_005702.T1, Maole_005713.T1, Maole_006486.T1, M
ole_011520.T1, Maole_012607.T1, Maole_012929.T1, Maole_012936.T1, Maole_013071.T1, M

ole_005179.T1, Maole_005180.T1, Maole_005194.T1, Maole_005266.T1, Maole_005269.T1, M
ole_009664.T1, Maole_009665.T1, Maole_009666.T1, Maole_009867.T1, Maole_010105.T1, M
ole_019116.T1, Maole_019531.T1, Maole_019748.T1, Maole_019749.T1, Maole_019750.T1, M
ole_018119.T1, Maole_018229.T1, Maole_018251.T1, Maole_018434.T1, Maole_019211.T1, M

ole_011817.T1, Maole_012412.T1, Maole_014489.T1, Maole_015503.T1, Maole_020026.T1

ole_010649.T1, Maole_012880.T1, Maole_013187.T1, Maole_014526.T1, Maole_017439.T1, M

ole_019116.T1, Maole_019531.T1, Maole_019748.T1, Maole_019749.T1, Maole_019750.T1, M

ole_013807.T1, Maole_013808.T1, Maole_017285.T1, Maole_017521.T1, Maole_018137.T1, M
ole_003481.T1, Maole_003488.T1, Maole_003493.T1, Maole_003681.T1, Maole_003774.T1, M

ole_018022.T1, Maole_018119.T1, Maole_018229.T1, Maole_018251.T1, Maole_018328.T1, M

ole_003102.T1, Maole_003124.T1, Maole_003296.T1, Maole_003401.T1, Maole_003538.T1, M

ole_006011.T1, Maole_006150.T1, Maole_006961.T1, Maole_007054.T1, Maole_007081.T1, M
ole_019908.T1, Maole_020950.T1, Maole_020951.T1, Maole_021052.T1, Maole_023550.T1, M

ole_010276.T1, Maole_010517.T1, Maole_010889.T1, Maole_010890.T1, Maole_012742.T1, M

le_009666.T1, Maole_010178.T1, Maole_010179.T1, Maole_010180.T1, Maole_010181.T1, M

le_018119.T1, Maole_018229.T1, Maole_018251.T1, Maole_019211.T1, Maole_019276.T1, M

le_006015.T1, Maole_006016.T1, Maole_006017.T1, Maole_009664.T1, Maole_009665.T1, M

le_019116.T1, Maole_019531.T1, Maole_019748.T1, Maole_019749.T1, Maole_019750.T1, M
le_019116.T1, Maole_019531.T1, Maole_019748.T1, Maole_019749.T1, Maole_019750.T1, M

le_007202.T1, Maole_007203.T1, Maole_007204.T1, Maole_007205.T1, Maole_007208.T1, M

[aole_010377.T1, Maole_013041.T1, Maole_015999.T1, Maole_020337.T1, Maole_020346.T1,
[aole_017630.T1, Maole_017632.T1, Maole_018434.T1, Maole_019618.T1, Maole_020950.T1,
[aole_014489.T1, Maole_014652.T1, Maole_015503.T1
[aole_021244.T1, Maole_021574.T1, Maole_021627.T1, Maole_021799.T1, Maole_021801.T1,

[aole_010531.T1, Maole_010533.T1, Maole_012412.T1, Maole_014489.T1, Maole_014652.T1,
[aole_003684.T1, Maole_003774.T1, Maole_003945.T1, Maole_004079.T1, Maole_004489.T1,

[aole_017627.T1, Maole_017628.T1, Maole_017630.T1, Maole_017632.T1, Maole_018434.T1,
[aole_007214.T1, Maole_009325.T1, Maole_009326.T1, Maole_010377.T1, Maole_011014.T1,

[aole_006017.T1, Maole_006903.T1, Maole_007127.T1, Maole_007726.T1, Maole_007728.T1,

[aole_016809.T1, Maole_017692.T1, Maole_019473.T1, Maole_022440.T1

[aole_012902.T1, Maole_014131.T1, Maole_014132.T1, Maole_014133.T1, Maole_014134.T1,
[aole_007887.T1, Maole_007888.T1, Maole_008287.T1, Maole_008476.T1, Maole_010889.T1,
[aole_006487.T1, Maole_006961.T1, Maole_006987.T1, Maole_007054.T1, Maole_007081.T1,
[aole_013581.T1, Maole_013713.T1, Maole_013714.T1, Maole_014126.T1, Maole_015433.T1,

[aole_005694.T1, Maole_005695.T1, Maole_005698.T1, Maole_005700.T1, Maole_005702.T1,
[aole_010178.T1, Maole_010179.T1, Maole_010180.T1, Maole_010181.T1, Maole_010184.T1,
[aole_019753.T1, Maole_019754.T1, Maole_019755.T1, Maole_019756.T1, Maole_019757.T1,
[aole_019276.T1, Maole_020950.T1, Maole_020951.T1, Maole_020952.T1, Maole_023127.T1,

[aole_017441.T1, Maole_018119.T1, Maole_018229.T1, Maole_018251.T1, Maole_019211.T1,

[aole_019753.T1, Maole_019754.T1, Maole_019755.T1, Maole_019756.T1, Maole_019757.T1,

[aole_019907.T1, Maole_019908.T1, Maole_023205.T1, Maole_023211.T1, Maole_024088.T1,
[aole_003921.T1, Maole_003945.T1, Maole_004053.T1, Maole_004056.T1, Maole_004057.T1,

[aole_019065.T1, Maole_019106.T1, Maole_019211.T1, Maole_019276.T1, Maole_020748.T1,

[aole_003540.T1, Maole_003774.T1, Maole_003834.T1, Maole_003836.T1, Maole_004233.T1,

[aole_007182.T1, Maole_007191.T1, Maole_007193.T1, Maole_007194.T1, Maole_007195.T1,
[aole_024105.T1

[aole_014253.T1, Maole_015424.T1, Maole_015426.T1, Maole_015427.T1, Maole_015768.T1,

[illegible]aole_010184.T1, Maole_012412.T1, Maole_013028.T1, Maole_014489.T1, Maole_015503.T1,

[illegible]aole_022946.T1, Maole_023134.T1

[illegible]aole_009666.T1, Maole_010178.T1, Maole_010179.T1, Maole_010180.T1, Maole_010181.T1,

[illegible]aole_019753.T1, Maole_019754.T1, Maole_019755.T1, Maole_019756.T1, Maole_019757.T1,
[illegible]aole_019753.T1, Maole_019754.T1, Maole_019755.T1, Maole_019756.T1, Maole_019757.T1,

[illegible]aole_007209.T1, Maole_007211.T1, Maole_007214.T1, Maole_008287.T1, Maole_008288.T1,

Maole_021033.T1, Maole_022371.T1, Maole_022372.T1, Maole_022373.T1, Maole_023452.T
Maole_020951.T1, Maole_020952.T1, Maole_021052.T1, Maole_021133.T1, Maole_021135.T

Maole_022097.T1, Maole_022164.T1, Maole_023127.T1, Maole_023666.T1

Maole_014826.T1, Maole_015503.T1, Maole_015784.T1, Maole_017955.T1, Maole_017956.T
Maole_004491.T1, Maole_004679.T1, Maole_004870.T1, Maole_004968.T1, Maole_004969.T

Maole_020900.T1, Maole_021052.T1, Maole_022500.T1, Maole_022504.T1, Maole_022507.T
Maole_012027.T1, Maole_012028.T1, Maole_013042.T1, Maole_014526.T1, Maole_015999.T

Maole_007730.T1, Maole_008673.T1, Maole_008917.T1, Maole_009664.T1, Maole_009665.T

Maole_014135.T1, Maole_014148.T1, Maole_014150.T1, Maole_015427.T1, Maole_016802.T
Maole_010890.T1, Maole_012383.T1, Maole_012384.T1, Maole_015203.T1, Maole_015774.T
Maole_007182.T1, Maole_007191.T1, Maole_007193.T1, Maole_007194.T1, Maole_007195.T
Maole_015455.T1, Maole_016034.T1, Maole_016670.T1, Maole_016782.T1, Maole_018871.T

Maole_005713.T1, Maole_006011.T1, Maole_006150.T1, Maole_006486.T1, Maole_006487.T
Maole_011036.T1, Maole_011062.T1, Maole_011817.T1, Maole_012383.T1, Maole_012384.T
Maole_021133.T1, Maole_021135.T1, Maole_023204.T1, Maole_023205.T1, Maole_023206.T
Maole_023134.T1, Maole_023348.T1, Maole_023352.T1, Maole_023550.T1

Maole_019276.T1, Maole_019418.T1, Maole_019419.T1, Maole_019420.T1, Maole_019618.T

Maole_021133.T1, Maole_021135.T1, Maole_023204.T1, Maole_023205.T1, Maole_023206.T

Maole_024112.T1
Maole_004058.T1, Maole_004175.T1, Maole_004372.T1, Maole_004375.T1, Maole_004377.T

Maole_020900.T1, Maole_021963.T1, Maole_023024.T1, Maole_023134.T1

Maole_004288.T1, Maole_004342.T1, Maole_004354.T1, Maole_004362.T1, Maole_004613.T

Maole_007196.T1, Maole_007198.T1, Maole_007199.T1, Maole_007201.T1, Maole_007202.T

Maole_015774.T1, Maole_015815.T1, Maole_015816.T1, Maole_016516.T1, Maole_017521.T

Maole_016334.T1, Maole_016766.T1, Maole_017439.T1, Maole_017441.T1

Maole_010184.T1, Maole_010531.T1, Maole_010533.T1, Maole_011817.T1, Maole_012089.T

Maole_021133.T1, Maole_021135.T1, Maole_023204.T1, Maole_023205.T1, Maole_023206.T
Maole_021133.T1, Maole_021135.T1, Maole_023204.T1, Maole_023205.T1, Maole_023206.T

Maole_008931.T1, Maole_009325.T1, Maole_009326.T1, Maole_010377.T1, Maole_010787.T

'1, Maole_023453.T1
'1, Maole_023348.T1, Maole_023352.T1, Maole_023550.T1, Maole_024105.T1

'1, Maole_023511.T1
'1, Maole_004987.T1, Maole_005107.T1, Maole_005145.T1, Maole_005210.T1, Maole_005341

'1, Maole_022510.T1, Maole_022511.T1, Maole_022513.T1, Maole_022974.T1, Maole_024105
'1, Maole_018119.T1, Maole_020114.T1, Maole_020115.T1, Maole_020117.T1, Maole_020119

'1, Maole_009666.T1, Maole_009867.T1, Maole_010178.T1, Maole_010179.T1, Maole_010180

'1, Maole_016803.T1, Maole_016807.T1, Maole_016809.T1, Maole_017692.T1, Maole_019473
'1, Maole_015784.T1, Maole_015853.T1, Maole_017527.T1, Maole_017955.T1, Maole_017956
'1, Maole_007196.T1, Maole_007198.T1, Maole_007199.T1, Maole_007201.T1, Maole_007202
'1, Maole_018906.T1, Maole_020020.T1, Maole_020217.T1, Maole_020418.T1, Maole_020448

'1, Maole_006961.T1, Maole_006987.T1, Maole_006990.T1, Maole_007054.T1, Maole_007081
'1, Maole_012412.T1, Maole_012510.T1, Maole_012895.T1, Maole_013425.T1, Maole_013426
'1, Maole_023208.T1, Maole_023211.T1

'1, Maole_020748.T1, Maole_021133.T1, Maole_021135.T1, Maole_021963.T1, Maole_02313[illegible]

'1, Maole_023208.T1, Maole_023211.T1

'1, Maole_004639.T1, Maole_004679.T1, Maole_004870.T1, Maole_004967.T1, Maole_00496[illegible]

'1, Maole_004614.T1, Maole_004615.T1, Maole_004752.T1, Maole_004753.T1, Maole_00512[illegible]

'1, Maole_007203.T1, Maole_007204.T1, Maole_007205.T1, Maole_007206.T1, Maole_00720[illegible]

'1, Maole_017527.T1, Maole_018137.T1, Maole_018667.T1, Maole_019531.T1, Maole_01974[illegible]

1, Maole_012412.T1, Maole_013992.T1, Maole_013993.T1, Maole_013994.T1, Maole_01400

1, Maole_023208.T1, Maole_023211.T1
1, Maole_023208.T1, Maole_023211.T1

1, Maole_010789.T1, Maole_013041.T1, Maole_013640.T1, Maole_015999.T1, Maole_01764

.T1, Maole_005416.T1, Maole_005495.T1, Maole_005619.T1, Maole_005691.T1, Maole_0056

.T1
.T1, Maole_020346.T1, Maole_021033.T1, Maole_022368.T1, Maole_022371.T1, Maole_0223

.T1, Maole_010181.T1, Maole_010184.T1, Maole_010531.T1, Maole_011036.T1, Maole_0110

.T1, Maole_019956.T1, Maole_019959.T1, Maole_019963.T1, Maole_020026.T1, Maole_0201
.T1, Maole_018022.T1, Maole_019065.T1, Maole_019067.T1, Maole_021248.T1, Maole_0212
.T1, Maole_007203.T1, Maole_007204.T1, Maole_007205.T1, Maole_007206.T1, Maole_0072
.T1, Maole_020658.T1, Maole_020839.T1, Maole_021244.T1, Maole_021291.T1, Maole_0216

.T1, Maole_007182.T1, Maole_007191.T1, Maole_007193.T1, Maole_007194.T1, Maole_0071
.T1, Maole_013428.T1, Maole_013627.T1, Maole_014489.T1, Maole_014581.T1, Maole_0145

ı.T1

ı.T1, Maole_004969.T1, Maole_005107.T1, Maole_005145.T1, Maole_005210.T1, Maole_0052

).T1, Maole_005178.T1, Maole_005179.T1, Maole_005180.T1, Maole_005495.T1, Maole_0056

ı.T1, Maole_007209.T1, Maole_007211.T1, Maole_007214.T1, Maole_007563.T1, Maole_0078

ı.T1, Maole_019749.T1, Maole_019750.T1, Maole_019753.T1, Maole_019754.T1, Maole_0197

.T1, Maole_014489.T1, Maole_014681.T1, Maole_015503.T1, Maole_016630.T1, Maole_0196

.T1, Maole_020337.T1, Maole_020346.T1, Maole_021033.T1, Maole_022371.T1, Maole_0223

96.T1, Maole_005759.T1, Maole_005836.T1, Maole_005991.T1, Maole_006013.T1, Maole_00

72.T1, Maole_022373.T1, Maole_023450.T1, Maole_023452.T1, Maole_023453.T1, Maole_02

61.T1, Maole_011062.T1, Maole_011817.T1, Maole_012089.T1, Maole_012412.T1, Maole_01

14.T1, Maole_020115.T1, Maole_020117.T1, Maole_020119.T1, Maole_020579.T1, Maole_02
53.T1, Maole_021341.T1, Maole_021342.T1, Maole_021438.T1, Maole_021896.T1, Maole_02
08.T1, Maole_007209.T1, Maole_007211.T1, Maole_007214.T1, Maole_007441.T1, Maole_00
26.T1, Maole_021627.T1, Maole_021897.T1, Maole_022097.T1, Maole_022164.T1, Maole_02

95.T1, Maole_007196.T1, Maole_007198.T1, Maole_007199.T1, Maole_007201.T1, Maole_00
82.T1, Maole_014612.T1, Maole_015115.T1, Maole_015424.T1, Maole_015426.T1, Maole_01

38.T1, Maole_005341.T1, Maole_005631.T1, Maole_005649.T1, Maole_005752.T1, Maole_00

94.T1, Maole_005695.T1, Maole_005698.T1, Maole_005700.T1, Maole_005702.T1, Maole_00

37.T1, Maole_008287.T1, Maole_008288.T1, Maole_009104.T1, Maole_009186.T1, Maole_00

55.T1, Maole_019756.T1, Maole_019757.T1, Maole_021234.T1, Maole_021480.T1, Maole_02

18.T1, Maole_019956.T1, Maole_019959.T1, Maole_019963.T1, Maole_023090.T1, Maole_02

72.T1, Maole_022373.T1, Maole_023452.T1, Maole_023453.T1, Maole_023752.T1, Maole_02

6014.T1, Maole_006015.T1, Maole_006016.T1, Maole_006017.T1, Maole_006167.T1, Maole_(

3556.T1, Maole_023558.T1, Maole_023986.T1, Maole_023988.T1, Maole_024067.T1

2510.T1, Maole_012895.T1, Maole_012897.T1, Maole_013491.T1, Maole_013627.T1, Maole_(

0900.T1, Maole_021600.T1, Maole_022440.T1, Maole_022503.T1, Maole_022508.T1, Maole_(
1898.T1, Maole_022379.T1, Maole_023511.T1, Maole_023736.T1, Maole_023737.T1, Maole_(
7523.T1, Maole_007563.T1, Maole_007837.T1, Maole_008287.T1, Maole_008288.T1, Maole_(
2328.T1, Maole_023960.T1

7202.T1, Maole_007203.T1, Maole_007204.T1, Maole_007205.T1, Maole_007206.T1, Maole_(
5427.T1, Maole_015503.T1, Maole_018581.T1, Maole_018972.T1, Maole_018973.T1, Maole_(

5991.T1, Maole_006239.T1, Maole_006342.T1, Maole_006343.T1, Maole_006464.T1, Maole_[illegible]

5712.T1, Maole_005715.T1, Maole_006013.T1, Maole_006014.T1, Maole_006015.T1, Maole_[illegible]

9325.T1, Maole_009326.T1, Maole_010102.T1, Maole_010377.T1, Maole_010649.T1, Maole_[illegible]

1613.T1, Maole_022416.T1, Maole_022935.T1, Maole_023204.T1, Maole_023205.T1, Maole_[illegible]

3092.T1, Maole_023402.T1, Maole_023574.T1, Maole_023780.T1, Maole_023782.T1

4037.T1

006239.T1, Maole_006379.T1, Maole_006380.T1, Maol

013992.T1, Maole_013993.T1, Maole_013994.T1, Maol

023090.T1, Maole_023091.T1, Maole_023092.T1, Maol
023741.T1, Maole_023742.T1
008519.T1, Maole_008609.T1, Maole_008931.T1, Maol

007208.T1, Maole_007209.T1, Maole_007211.T1, Maol
018974.T1, Maole_019117.T1, Maole_020579.T1, Maol

)06513.T1, Maole_006532.T1, Maole_006824.T1, Maol

)06016.T1, Maole_006017.T1, Maole_006121.T1, Maol

)11635.T1, Maole_012880.T1, Maole_013041.T1, Maol

)23206.T1, Maole_023208.T1, Maole_023211.T1, Maol

 Click here to download Table Table S16.xlsx

Category	P_value	Q_value	numDEInCat	numInCat	Term
ko04075	3.37E-11	4.41E-09	50	264	Plant hormone signal tr
ko00350	1.97E-09	1.29E-07	20	59	Tyrosine metabolism
ko00950	3.04E-08	1.33E-06	14	34	Isoquinoline alkaloid b
ko00073	2.63E-06	8.62E-05	14	47	Cutin, suberine and wa
ko00900	3.70E-05	0.00097039	13	50	Terpenoid backbone bi
ko00770	0.000524016	0.011441026	8	27	Pantothenate and CoA
ko00010	0.000663247	0.012412186	19	115	Glycolysis/Gluconeoge

**Class**	**Gene ID**
Environm	Maole_000378.T1, Maole_000437.T1, Maole_000677.T1, Maole_001428.T1, Maole_(
Metabolis	Maole_001779.T1, Maole_001782.T1, Maole_001785.T1, Maole_006598.T1, Maole_(
Metabolis	Maole_001779.T1, Maole_001782.T1, Maole_001785.T1, Maole_016084.T1, Maole_(
Metabolis	Maole_002180.T1, Maole_002181.T1, Maole_002183.T1, Maole_005259.T1, Maole_(
Metabolis	Maole_004288.T1, Maole_005614.T1, Maole_010786.T1, Maole_012177.T1, Maole_(
Metabolis	Maole_004372.T1, Maole_004375.T1, Maole_004377.T1, Maole_004489.T1, Maole_(
Metabolis	Maole_000911.T1, Maole_001047.T1, Maole_001348.T1, Maole_003102.T1, Maole_(

)01434.T1, Maole_001560.T1, Maole_002027.T1, Maole_002978.T1, Maole_003001.T1, M
)06603.T1, Maole_006604.T1, Maole_008476.T1, Maole_016084.T1, Maole_016085.T1, M
)16085.T1, Maole_021078.T1, Maole_021081.T1, Maole_021238.T1, Maole_023059.T1, M
)07733.T1, Maole_010272.T1, Maole_010273.T1, Maole_010274.T1, Maole_010276.T1, M
)12178.T1, Maole_015768.T1, Maole_020036.T1, Maole_022028.T1, Maole_022128.T1, M
)04491.T1, Maole_006869.T1, Maole_010845.T1, Maole_010846.T1
)04233.T1, Maole_006598.T1, Maole_006603.T1, Maole_006604.T1, Maole_008161.T1, M

[illegible]aole_003161.T1, Maole_003786.T1, Maole_003790.T1, Maole_003791.T1, Maole_003793.T1,
[illegible]aole_021078.T1, Maole_021081.T1, Maole_021238.T1, Maole_021896.T1, Maole_021898.T1,
[illegible]aole_023061.T1, Maole_023063.T1, Maole_023064.T1, Maole_023690.T1, Maole_024007.T1
[illegible]aole_015424.T1, Maole_015426.T1, Maole_015427.T1, Maole_017521.T1, Maole_021480.T1
[illegible]aole_022130.T1, Maole_022264.T1, Maole_022954.T1, Maole_023795.T1

[illegible]aole_008476.T1, Maole_011566.T1, Maole_014681.T1, Maole_014682.T1, Maole_014826.T1,

Maole_003796.T1, Maole_003875.T1, Maole_004490.T1, Maole_004776.T1, Maole_005062.T
Maole_023059.T1, Maole_023061.T1, Maole_023063.T1, Maole_023064.T1, Maole_023690.T

Maole_020576.T1, Maole_021896.T1, Maole_021898.T1, Maole_022877.T1, Maole_023342.T

'1, Maole_005713.T1, Maole_007054.T1, Maole_007081.T1, Maole_007563.T1, Maole_009104
'1, Maole_024007.T1

'1

[illegible].T1, Maole_009186.T1, Maole_010102.T1, Maole_010649.T1, Maole_012880.T1, Maole_0128

95.T1, Maole_012897.T1, Maole_013187.T1, Maole_014526.T1, Maole_018119.T1, Maole_01[illegible]

8229.T1, Maole_018251.T1, Maole_018328.T1, Maole_019106.T1, Maole_019211.T1, Maole_(

)19276.T1, Maole_019618.T1, Maole_020748.T1, Maole_021963.T1, Maole_023024.T1, Maole

e_023134.T1, Maole_023400.T1, Maole_023402.T1, Maole_023778.T1, Maole_023780.T1, Ma

ole_023782.T1, Maole_024102.T1

 Click here to download Table Table S17.xlsx

Category	P_value	Q_value	numDEInCat	numInCat
GO:0005886	7.56E-176	6.57E-172	1,006	3,020
GO:0016021	1.45E-122	6.32E-119	1,011	3,575
GO:0009506	4.94E-112	1.43E-108	598	1,668
GO:0004674	4.45E-84	9.67E-81	388	981
GO:0005524	2.48E-71	4.30E-68	745	2,706
GO:0046777	3.27E-70	4.74E-67	305	742
GO:0001653	2.43E-64	3.01E-61	246	558
GO:0009755	4.12E-64	4.47E-61	253	587
GO:0004675	1.67E-63	1.62E-60	242	548
GO:0007178	2.74E-63	2.38E-60	242	549
GO:0031625	4.26E-61	3.37E-58	260	627
GO:0016020	3.76E-58	2.72E-55	548	1,950
GO:0004672	8.75E-58	5.85E-55	330	928
GO:0030246	5.03E-30	3.12E-27	124	300
GO:0005516	1.75E-26	1.01E-23	98	217
GO:0015293	1.86E-25	1.01E-22	60	101
GO:0006468	3.10E-24	1.58E-21	90	195
GO:0005774	6.34E-23	3.06E-20	197	655
GO:0005773	2.21E-22	1.01E-19	213	757
GO:0048544	2.92E-22	1.27E-19	68	138
GO:0005768	9.34E-20	3.87E-17	125	359
GO:0009624	5.64E-18	2.23E-15	59	122
GO:0000139	3.07E-16	1.16E-13	115	347
GO:0035556	3.60E-16	1.30E-13	55	115
GO:0004714	5.10E-16	1.77E-13	43	84
GO:0005794	2.22E-15	7.43E-13	225	902
GO:0005215	3.31E-14	1.07E-11	80	222
GO:0009738	5.65E-14	1.75E-11	94	296
GO:0005618	7.14E-14	2.14E-11	185	783
GO:0005802	1.36E-13	3.93E-11	98	302
GO:0071555	5.75E-13	1.61E-10	120	432
GO:0042742	6.91E-13	1.87E-10	116	416
GO:0006855	7.11E-13	1.87E-10	21	26
GO:0046274	2.58E-12	6.58E-10	18	21
GO:0016747	3.93E-12	9.75E-10	35	78
GO:0009932	6.46E-12	1.56E-09	33	63
GO:0010540	9.84E-12	2.31E-09	26	42
GO:0006865	1.39E-11	3.18E-09	27	47
GO:0015112	1.53E-11	3.41E-09	16	18
GO:0016887	1.79E-11	3.88E-09	78	231
GO:0010359	2.27E-11	4.80E-09	23	38
GO:0052716	2.89E-11	5.97E-09	18	23
GO:0048767	3.46E-11	6.99E-09	44	109
GO:0033612	4.34E-11	8.58E-09	25	46
GO:0009742	5.36E-11	1.04E-08	46	122
GO:0048046	6.81E-11	1.29E-08	142	597
GO:0071215	1.44E-10	2.66E-08	32	65

GO:0018105	2.11E-10	3.82E-08	29	53
GO:0042626	2.92E-10	5.18E-08	29	55
GO:0016045	3.84E-10	6.59E-08	18	27
GO:0005737	3.87E-10	6.59E-08	512	2,690
GO:0080167	5.35E-10	8.94E-08	51	154
GO:0000325	5.95E-10	9.75E-08	40	96
GO:0010152	1.02E-09	1.65E-07	20	34
GO:0042802	1.27E-09	2.00E-07	77	264
GO:0019199	1.38E-09	2.14E-07	24	46
GO:0008559	1.52E-09	2.31E-07	18	25
GO:0033807	1.67E-09	2.50E-07	13	17
GO:0055085	2.06E-09	3.03E-07	74	246
GO:0009556	2.32E-09	3.35E-07	32	75
GO:0051258	3.26E-09	4.65E-07	15	21
GO:0022626	3.42E-09	4.79E-07	84	309
GO:0004565	4.43E-09	6.10E-07	18	26
GO:0005887	8.12E-09	1.10E-06	62	211
GO:0034613	1.19E-08	1.59E-06	20	33
GO:0010008	1.44E-08	1.87E-06	48	139
GO:0015171	1.44E-08	1.87E-06	25	56
GO:0009737	1.50E-08	1.91E-06	124	552
GO:0009860	1.51E-08	1.91E-06	51	160
GO:0010025	1.92E-08	2.39E-06	25	54
GO:0016760	2.11E-08	2.58E-06	21	36
GO:0004683	2.20E-08	2.65E-06	21	37
GO:0006857	2.55E-08	3.04E-06	25	53
GO:0009931	2.60E-08	3.05E-06	20	35
GO:0009651	2.75E-08	3.18E-06	146	670
GO:0015172	3.79E-08	4.28E-06	10	11
GO:0015175	3.79E-08	4.28E-06	10	11
GO:0007165	4.04E-08	4.50E-06	60	202
GO:0008289	4.99E-08	5.49E-06	36	95
GO:0009825	5.35E-08	5.81E-06	27	60
GO:0046658	5.42E-08	5.82E-06	37	103
GO:0009505	5.80E-08	6.15E-06	100	422
GO:0015079	5.99E-08	6.27E-06	18	32
GO:0009620	8.02E-08	8.27E-06	45	139
GO:0000271	8.10E-08	8.27E-06	20	40
GO:0005975	1.22E-07	1.23E-05	110	471
GO:0016757	1.34E-07	1.32E-05	49	156
GO:0009734	1.35E-07	1.32E-05	68	264
GO:0043481	1.35E-07	1.32E-05	23	49
GO:0005938	1.40E-07	1.35E-05	19	34
GO:0004702	1.42E-07	1.35E-05	21	42
GO:0010227	1.45E-07	1.35E-05	26	64
GO:0080168	1.45E-07	1.35E-05	10	11
GO:0009922	1.89E-07	1.75E-05	12	19
GO:0015399	2.04E-07	1.86E-05	9	10

GO:0015804	2.09E-07	1.89E-05	10	12
GO:0015833	2.17E-07	1.95E-05	12	17
GO:0009294	2.29E-07	2.03E-05	23	49
GO:0005509	2.48E-07	2.18E-05	74	291
GO:0048513	2.64E-07	2.30E-05	15	25
GO:0009873	3.14E-07	2.70E-05	52	200
GO:0009850	3.18E-07	2.71E-05	14	22
GO:0010222	3.63E-07	3.06E-05	10	12
GO:0010192	3.70E-07	3.09E-05	11	14
GO:0015145	3.78E-07	3.13E-05	12	18
GO:0009705	4.53E-07	3.72E-05	44	138
GO:0009416	4.71E-07	3.82E-05	40	133
GO:0005874	5.30E-07	4.27E-05	57	192
GO:0005313	5.98E-07	4.77E-05	10	13
GO:0046556	9.39E-07	7.41E-05	9	10
GO:0003333	9.56E-07	7.41E-05	20	45
GO:0015802	9.63E-07	7.41E-05	12	18
GO:0046686	9.64E-07	7.41E-05	104	441
GO:0015800	1.05E-06	7.98E-05	9	11
GO:0010315	1.08E-06	8.15E-05	17	34
GO:0090332	1.09E-06	8.20E-05	13	21
GO:0042128	1.18E-06	8.74E-05	17	32
GO:0015238	1.30E-06	9.59E-05	23	53
GO:0010075	1.44E-06	0.000104988	35	107
GO:0005200	1.49E-06	0.000107131	14	25
GO:0016032	1.49E-06	0.000107131	51	181
GO:0043622	1.69E-06	0.000120064	18	35
GO:0015198	1.75E-06	0.000122463	9	12
GO:0006810	1.76E-06	0.000122463	60	211
GO:0002237	1.76E-06	0.000122463	23	57
GO:0051753	1.83E-06	0.000126482	10	13
GO:0032440	1.86E-06	0.000127458	39	132
GO:0009617	1.94E-06	0.000131929	47	167
GO:0009611	2.45E-06	0.00016507	76	330
GO:1902600	2.64E-06	0.0001767	12	18
GO:0015297	2.75E-06	0.000182399	34	104
GO:0045490	2.89E-06	0.000190331	29	88
GO:0023014	2.91E-06	0.000190353	22	52
GO:0009674	3.01E-06	0.000194858	7	7
GO:0015992	3.08E-06	0.000198194	31	89
GO:0080051	3.12E-06	0.000199398	7	7
GO:0015416	3.44E-06	0.000216882	7	7
GO:0005388	3.44E-06	0.000216882	12	19
GO:0080092	3.69E-06	0.000230707	16	33
GO:0033956	3.86E-06	0.000239309	7	7
GO:0005355	4.46E-06	0.000271073	21	50
GO:0035428	4.46E-06	0.000271073	21	50
GO:0046323	4.46E-06	0.000271073	21	50

GO:0004012	4.67E-06	0.000281802	7	7
GO:0005507	5.24E-06	0.000313705	55	197
GO:0009813	5.46E-06	0.000324763	46	192
GO:0008422	5.85E-06	0.00034419	16	31
GO:0009555	5.91E-06	0.00034419	63	241
GO:0000148	5.98E-06	0.00034419	8	9
GO:0003843	5.98E-06	0.00034419	8	9
GO:0006075	5.98E-06	0.00034419	8	9
GO:0035251	6.13E-06	0.000350188	25	85
GO:0046470	6.70E-06	0.000380488	9	12
GO:0090333	6.86E-06	0.000384194	11	16
GO:0008810	6.86E-06	0.000384194	13	25
GO:0016049	6.90E-06	0.000384194	23	60
GO:0006898	7.25E-06	0.000401427	11	17
GO:0052544	8.05E-06	0.000442499	19	48
GO:0004497	9.07E-06	0.000495868	65	283
GO:0043090	1.14E-05	0.000617307	18	42
GO:0052696	1.14E-05	0.000617307	30	116
GO:0022891	1.20E-05	0.000645395	20	49
GO:0005544	1.27E-05	0.000677376	13	26
GO:0015696	1.40E-05	0.000744123	10	16
GO:0004713	1.45E-05	0.000763765	21	51
GO:0005496	1.52E-05	0.000797568	13	26
GO:0005546	1.73E-05	0.000897744	8	10
GO:0030245	1.74E-05	0.000897744	13	26
GO:0006096	1.79E-05	0.000920982	37	116
GO:0071944	2.04E-05	0.001040475	12	21
GO:0031225	2.11E-05	0.001069741	40	156
GO:0000390	2.13E-05	0.001077205	6	6
GO:0008272	2.16E-05	0.001083647	11	18
GO:0015180	2.33E-05	0.001157829	7	9
GO:0015808	2.33E-05	0.001157829	7	9
GO:0008360	2.51E-05	0.001225663	15	30
GO:0002764	2.54E-05	0.001225663	8	12
GO:0010328	2.54E-05	0.001225663	8	11
GO:0060919	2.54E-05	0.001225663	8	11
GO:0080043	2.56E-05	0.001225663	29	115
GO:0080044	2.56E-05	0.001225663	29	115
GO:0012510	2.57E-05	0.001225663	8	10
GO:0010329	3.16E-05	0.001498084	13	26
GO:0010541	3.24E-05	0.001531115	10	16
GO:0016311	3.33E-05	0.001565734	31	100
GO:0005351	3.62E-05	0.001690859	21	56
GO:0016702	3.67E-05	0.001703234	9	13
GO:0048443	3.89E-05	0.001799378	22	67
GO:0015193	4.14E-05	0.001892095	7	9
GO:0035524	4.14E-05	0.001892095	7	9
GO:1900994	4.59E-05	0.002089898	10	19

GO:0007112	4.79E-05	0.002149492	9	13
GO:0006868	4.87E-05	0.002149492	6	7
GO:0015186	4.87E-05	0.002149492	6	7
GO:0015194	4.87E-05	0.002149492	6	7
GO:0015825	4.87E-05	0.002149492	6	7
GO:0098712	4.87E-05	0.002149492	6	7
GO:0009823	5.03E-05	0.002197296	7	9
GO:0019139	5.03E-05	0.002197296	7	9
GO:0009911	6.06E-05	0.002632713	25	73
GO:0030244	6.24E-05	0.002698213	31	94
GO:0009816	6.38E-05	0.002743469	30	98
GO:1901684	6.95E-05	0.002973867	6	8
GO:0009753	7.33E-05	0.003122455	54	235
GO:0045548	7.47E-05	0.003165783	5	5
GO:0030570	7.53E-05	0.003174828	9	16
GO:0048437	7.65E-05	0.003209579	11	23
GO:0043231	7.94E-05	0.003315814	51	225
GO:0010588	8.46E-05	0.003504444	14	32
GO:0002020	8.47E-05	0.003504444	9	15
GO:0009733	8.51E-05	0.003504444	68	307
GO:0016036	9.13E-05	0.003739722	33	113
GO:0015749	9.30E-05	0.00379456	7	10
GO:0010105	9.65E-05	0.003916818	14	33
GO:0047274	9.98E-05	0.004032799	5	5
GO:0010011	0.000105357	0.00423769	6	7
GO:0009723	0.000107276	0.004294994	52	229
GO:0001944	0.000111657	0.004449882	18	52
GO:0008643	0.000115091	0.004565789	29	98
GO:0048507	0.000116319	0.004593534	11	25
GO:0090342	0.000121161	0.004763097	7	9
GO:0050378	0.000122431	0.004791342	4	4
GO:0050829	0.00012362	0.004816204	9	16
GO:0034440	0.000127587	0.004948549	6	7
GO:0005684	0.000131772	0.005088148	6	7
GO:0006882	0.000134691	0.005177869	9	15
GO:0005829	0.000137401	0.005258783	413	2,333
GO:0009926	0.000139215	0.005303496	25	80
GO:0035195	0.000139791	0.005303496	5	5
GO:0004630	0.000141829	0.005334227	9	15
GO:0070290	0.000141829	0.005334227	9	15
GO:0016165	0.000143338	0.005367771	5	5
GO:0060548	0.000145956	0.005442334	13	29
GO:0010345	0.00014816	0.005500927	15	40
GO:1902358	0.000150058	0.00554767	5	5
GO:0080172	0.000152884	0.00562819	5	5
GO:0010234	0.000154994	0.005669898	10	20
GO:0010290	0.000155334	0.005669898	5	5
GO:0071366	0.000155974	0.005669898	5	5

GO:0035252	0.000161731	0.005854647	7	11
GO:0005615	0.000165813	0.005977542	31	110
GO:0009846	0.000171474	0.006156046	20	55
GO:0048229	0.000173504	0.006203293	11	22
GO:0015706	0.00017734	0.006314451	21	66
GO:0008519	0.000189671	0.006725967	4	4
GO:0006097	0.00021778	0.007664162	8	13
GO:0009626	0.000217892	0.007664162	35	128
GO:0035435	0.000230731	0.008083045	10	19
GO:0034004	0.000232833	0.008091417	6	8
GO:0052577	0.000232833	0.008091417	6	8
GO:0006897	0.000241862	0.008371698	28	90
GO:0090558	0.000248627	0.008571705	6	8
GO:0010231	0.000252743	0.008662866	8	13
GO:0047760	0.000253692	0.008662866	4	4
GO:0008219	0.000254262	0.008662866	22	66
GO:0042218	0.000267452	0.009076657	4	4
GO:0031408	0.000268983	0.009093096	22	66
GO:0009927	0.000283258	0.00953855	9	17
GO:0010103	0.000288383	0.009673629	38	146
GO:0005262	0.000294927	0.009855089	12	25
GO:0004715	0.000314035	0.01045341	19	55
GO:0060320	0.000316608	0.010498828	7	11
GO:0008271	0.000324775	0.010728685	7	10
GO:0042335	0.000327016	0.01073487	15	38
GO:0009729	0.000327433	0.01073487	7	11
GO:0000287	0.000333676	0.010863331	51	205
GO:0071281	0.000333852	0.010863331	19	59
GO:0031514	0.000337398	0.010937734	8	15
GO:0009409	0.000344596	0.011129557	89	447
GO:0010023	0.000373974	0.01203364	11	24
GO:0016324	0.00038	0.012182422	16	45
GO:0000159	0.000383875	0.012261435	9	17
GO:0090408	0.000396712	0.012625046	4	4
GO:0045087	0.000403922	0.012807582	37	144
GO:0016758	0.000426274	0.013442086	35	162
GO:0009861	0.000427028	0.013442086	5	6
GO:0016705	0.000433455	0.013595157	55	260
GO:0047203	0.000435573	0.013612428	4	4
GO:0006878	0.000441924	0.013761411	6	8
GO:0009266	0.000463495	0.014333532	16	46
GO:1901683	0.000463596	0.014333532	5	7
GO:0008553	0.000467008	0.014387813	7	11
GO:0010311	0.000476251	0.014620738	23	73
GO:0046688	0.000501178	0.015295355	10	21
GO:0008378	0.000501747	0.015295355	11	23
GO:0010255	0.000524006	0.015918047	6	9
GO:0007017	0.000539997	0.01627428	13	35

GO:0010256	0.000540271	0.01627428	7	12
GO:1900424	0.000541352	0.01627428	6	8
GO:0006555	0.000548632	0.016419958	6	8
GO:0008017	0.000549978	0.016419958	21	61
GO:0005358	0.00055657	0.016503356	4	4
GO:0009679	0.00055657	0.016503356	4	4
GO:0042391	0.000563601	0.016654983	11	24
GO:0004629	0.000568925	0.016698704	4	4
GO:0034480	0.000568925	0.016698704	4	4
GO:0015245	0.00058773	0.017192575	5	6
GO:0010119	0.000590019	0.017201614	22	71
GO:1900140	0.000595157	0.017293381	11	25
GO:0009944	0.00060137	0.017378092	21	68
GO:0000822	0.000602072	0.017378092	4	4
GO:0016102	0.000608193	0.017496628	12	31
GO:0010204	0.000612111	0.017551217	11	26
GO:0033302	0.000627735	0.017881195	5	6
GO:0033329	0.000627735	0.017881195	5	6
GO:0015692	0.000641995	0.018168236	5	6
GO:0046865	0.000641995	0.018168236	5	6
GO:0006862	0.000666727	0.018806909	7	12
GO:0042936	0.000677291	0.018859954	7	11
GO:0042937	0.000677291	0.018859954	7	11
GO:0042938	0.000677291	0.018859954	7	11
GO:0042939	0.000677291	0.018859954	7	11
GO:0033773	0.000695891	0.019315983	6	10
GO:0009414	0.000700839	0.019391374	82	415
GO:0004435	0.000712923	0.019663106	5	6
GO:0010071	0.000725255	0.019939921	5	6
GO:0098655	0.000731888	0.020058812	10	21
GO:0005789	0.000779884	0.021307028	118	593
GO:0010245	0.00079416	0.021629037	5	6
GO:0015431	0.000798223	0.021671753	5	6
GO:0018108	0.000810487	0.021924829	8	14
GO:0006811	0.000812592	0.021924829	23	75
GO:0016847	0.000836913	0.022511139	5	7
GO:0010080	0.000841978	0.022577492	6	10
GO:0033500	0.000848645	0.022686241	6	9
GO:0009044	0.000852679	0.022724156	6	9
GO:0015691	0.000881149	0.023295761	7	12
GO:0005242	0.000884372	0.023295761	6	9
GO:0005249	0.000887113	0.023295761	11	25
GO:0004471	0.000892534	0.023295761	4	4
GO:0004721	0.000894383	0.023295761	27	96
GO:0042344	0.000900941	0.023295761	4	4
GO:0071275	0.000900941	0.023295761	4	4
GO:0015850	0.000900941	0.023295761	4	4
GO:0045332	0.000900941	0.023295761	4	4

GO:1901140	0.000900941	0.023295761	4	4
GO:0009833	0.000910197	0.023465256	6	9
GO:0016857	0.000914294	0.023501133	4	5
GO:0010583	0.000930921	0.023817222	12	31
GO:0005216	0.000932074	0.023817222	8	16
GO:0008026	0.000940678	0.023966601	15	40
GO:0043069	0.000945881	0.023998856	19	58
GO:0004383	0.000950231	0.023998856	7	13
GO:0006182	0.000950231	0.023998856	7	13
GO:0010319	0.000961215	0.024205903	17	50
GO:0016310	0.00099183	0.024904689	10	22
GO:0006559	0.001015926	0.02543621	7	12
GO:0004553	0.00102336	0.025546076	52	242
GO:0008285	0.001026195	0.025546076	14	39
GO:0003979	0.001037	0.02566797	3	3
GO:0006065	0.001037	0.02566797	3	3
GO:0008447	0.001054244	0.02602065	6	10
GO:0009524	0.001066178	0.026240656	37	145
GO:0051015	0.001075968	0.02640682	11	26
GO:0010067	0.001132877	0.027725161	10	23
GO:0010148	0.001140526	0.027833962	9	19
GO:0006812	0.001150228	0.027968039	10	22
GO:0048657	0.001152458	0.027968039	7	13
GO:0009958	0.001177891	0.028505622	16	47
GO:0006598	0.001182321	0.028533348	7	12
GO:0009925	0.001211234	0.029150135	9	19
GO:0002221	0.001221054	0.029257703	8	16
GO:0048497	0.001222439	0.029257703	6	9
GO:0055072	0.001249097	0.029741865	13	35
GO:0010398	0.001249514	0.029741865	5	7
GO:0006633	0.00126321	0.029985714	32	124
GO:0008601	0.001268998	0.030041012	8	16
GO:0009970	0.001297982	0.030641315	8	17
GO:0008152	0.001301409	0.030641315	64	294
GO:0047259	0.001327053	0.031160648	5	7
GO:0071249	0.001377802	0.032265089	4	5
GO:0080054	0.001395816	0.032511671	7	14
GO:0080055	0.001395816	0.032511671	7	14
GO:0090406	0.001409594	0.032744791	14	41
GO:1901141	0.001430131	0.033133272	5	7
GO:0008066	0.001453295	0.033491322	8	15
GO:0071230	0.001453295	0.033491322	8	15
GO:0005354	0.001467364	0.033726073	5	8
GO:0030173	0.001499604	0.034324497	9	21
GO:0006099	0.001503073	0.034324497	14	38
GO:0000976	0.001505252	0.034324497	5	7
GO:0019605	0.001516882	0.034499135	4	5
GO:0050321	0.001552952	0.03522727	5	7

GO:0006754	0.001615676	0.036554673	6	11
GO:0040009	0.001657955	0.037314188	3	3
GO:0046622	0.001657955	0.037314188	3	3
GO:0015116	0.001662131	0.037314188	5	7
GO:0004693	0.001748474	0.039151401	13	34
GO:0004315	0.001783582	0.039834856	5	7
GO:0006952	0.001817687	0.040419396	120	678
GO:0006869	0.001829613	0.040419396	19	64
GO:0090448	0.001833016	0.040419396	5	8
GO:0090449	0.001833016	0.040419396	5	8
GO:1901349	0.001833016	0.040419396	5	8
GO:0052638	0.001840872	0.040489863	6	11
GO:0009969	0.001898863	0.041659913	6	11
GO:0044550	0.001905975	0.041710609	23	94
GO:0047213	0.001935182	0.042243374	10	31
GO:0010167	0.001958089	0.042575818	16	54
GO:0010107	0.001960213	0.042575818	7	13
GO:0006108	0.002015978	0.043677855	7	13
GO:0071805	0.002073743	0.044817609	15	46
GO:0015398	0.002124553	0.045801767	3	3
GO:0016307	0.002134139	0.045894551	6	10
GO:0080117	0.002159104	0.046316771	4	5
GO:0003006	0.002228751	0.047693072	5	7
GO:0009864	0.002235436	0.047718588	10	28
GO:0097502	0.002247854	0.047866074	10	23
GO:0009934	0.002275801	0.048342679	13	37
GO:0005543	0.002343918	0.049668198	13	36

**Term**
plasma membrane
integral component of membrane
plasmodesma
protein serine/threonine kinase activity
ATP binding
protein autophosphorylation
peptide receptor activity
hormone-mediated signaling pathway
transmembrane receptor protein serine/threonine kinase activity
transmembrane receptor protein serine/threonine kinase signaling pathway
ubiquitin protein ligase binding
membrane
protein kinase activity
carbohydrate binding
calmodulin binding
symporter activity
protein phosphorylation
vacuolar membrane
vacuole
recognition of pollen
endosome
response to nematode
Golgi membrane
intracellular signal transduction
transmembrane receptor protein tyrosine kinase activity
Golgi apparatus
transporter activity
abscisic acid-activated signaling pathway
cell wall
trans-Golgi network
cell wall organization
defense response to bacterium
drug transmembrane transport
lignin catabolic process
transferase activity, transferring acyl groups other than amino-acyl groups
cell tip growth
basipetal auxin transport
amino acid transport
nitrate transmembrane transporter activity
ATPase activity
regulation of anion channel activity
hydroquinone:oxygen oxidoreductase activity
root hair elongation
receptor serine/threonine kinase binding
brassinosteroid mediated signaling pathway
apoplast
cellular response to abscisic acid stimulus

peptidyl-serine phosphorylation
ATPase activity, coupled to transmembrane movement of substances
detection of bacterium
cytoplasm
response to karrikin
plant-type vacuole
pollen maturation
identical protein binding
transmembrane receptor protein kinase activity
xenobiotic-transporting ATPase activity
icosanoyl-CoA synthase activity
transmembrane transport
microsporogenesis
protein polymerization
cytosolic ribosome
beta-galactosidase activity
integral component of plasma membrane
cellular protein localization
endosome membrane
amino acid transmembrane transporter activity
response to abscisic acid
pollen tube growth
wax biosynthetic process
cellulose synthase (UDP-forming) activity
calmodulin-dependent protein kinase activity
oligopeptide transport
calcium-dependent protein serine/threonine kinase activity
response to salt stress
acidic amino acid transmembrane transporter activity
neutral amino acid transmembrane transporter activity
signal transduction
lipid binding
multidimensional cell growth
anchored component of plasma membrane
plant-type cell wall
potassium ion transmembrane transporter activity
response to fungus
polysaccharide biosynthetic process
carbohydrate metabolic process
transferase activity, transferring glycosyl groups
auxin-activated signaling pathway
anthocyanin accumulation in tissues in response to UV light
cell cortex
receptor signaling protein serine/threonine kinase activity
floral organ abscission
abscisic acid transport
fatty acid elongase activity
primary active transmembrane transporter activity

neutral amino acid transport
peptide transport
DNA mediated transformation
calcium ion binding
organ development
ethylene-activated signaling pathway
auxin metabolic process
stem vascular tissue pattern formation
mucilage biosynthetic process
monosaccharide transmembrane transporter activity
plant-type vacuole membrane
response to light stimulus
microtubule
L-glutamate transmembrane transporter activity
alpha-L-arabinofuranosidase activity
amino acid transmembrane transport
basic amino acid transport
response to cadmium ion
acidic amino acid transport
auxin efflux
stomatal closure
nitrate assimilation
drug transmembrane transporter activity
regulation of meristem growth
structural constituent of cytoskeleton
viral process
cortical microtubule organization
oligopeptide transporter activity
transport
response to molecule of bacterial origin
mannan synthase activity
2-alkenal reductase [NAD(P)] activity
response to bacterium
response to wounding
hydrogen ion transmembrane transport
antiporter activity
pectin catabolic process
signal transduction by protein phosphorylation
potassium:sodium symporter activity
proton transport
cutin transport
organic phosphonate transmembrane-transporting ATPase activity
calcium-transporting ATPase activity
regulation of pollen tube growth
beta-apiosyl-beta-glucosidase activity
glucose transmembrane transporter activity
hexose transmembrane transport
glucose import

phospholipid-translocating ATPase activity
copper ion binding
flavonoid biosynthetic process
beta-glucosidase activity
pollen development
1,3-beta-D-glucan synthase complex
1,3-beta-D-glucan synthase activity
(1->3)-beta-D-glucan biosynthetic process
UDP-glucosyltransferase activity
phosphatidylcholine metabolic process
regulation of stomatal closure
cellulase activity
cell growth
receptor-mediated endocytosis
defense response by callose deposition in cell wall
monooxygenase activity
amino acid import
flavonoid glucuronidation
substrate-specific transmembrane transporter activity
calcium-dependent phospholipid binding
ammonium transport
protein tyrosine kinase activity
steroid binding
phosphatidylinositol-4,5-bisphosphate binding
cellulose catabolic process
glycolytic process
cell periphery
anchored component of membrane
spliceosomal complex disassembly
sulfate transport
L-alanine transmembrane transporter activity
L-alanine transport
regulation of cell shape
immune response-regulating signaling pathway
auxin influx transmembrane transporter activity
auxin influx
quercetin 3-O-glucosyltransferase activity
quercetin 7-O-glucosyltransferase activity
trans-Golgi network transport vesicle membrane
auxin efflux transmembrane transporter activity
acropetal auxin transport
dephosphorylation
sugar:proton symporter activity
oxidoreductase activity, acting on single donors with incorporation of molecular oxygen, incor
stamen development
L-proline transmembrane transporter activity
proline transmembrane transport
(-)-secologanin biosynthetic process

male meiosis cytokinesis
glutamine transport
L-glutamine transmembrane transporter activity
L-serine transmembrane transporter activity
L-serine transport
L-glutamate import across plasma membrane
cytokinin catabolic process
cytokinin dehydrogenase activity
positive regulation of flower development
cellulose biosynthetic process
defense response to bacterium, incompatible interaction
arsenate ion transmembrane transport
response to jasmonic acid
phenylalanine ammonia-lyase activity
pectate lyase activity
floral organ development
intracellular membrane-bounded organelle
cotyledon vascular tissue pattern formation
protease binding
response to auxin
cellular response to phosphate starvation
monosaccharide transport
negative regulation of ethylene-activated signaling pathway
galactinol-sucrose galactosyltransferase activity
auxin binding
response to ethylene
vasculature development
carbohydrate transport
meristem development
regulation of cell aging
UDP-glucuronate 4-epimerase activity
defense response to Gram-negative bacterium
lipid oxidation
U2-type spliceosomal complex
cellular zinc ion homeostasis
cytosol
auxin polar transport
gene silencing by miRNA
phospholipase D activity
N-acylphosphatidylethanolamine-specific phospholipase D activity
linoleate 13S-lipoxygenase activity
negative regulation of cell death
suberin biosynthetic process
sulfate transmembrane transport
petal epidermis patterning
anther wall tapetum cell fate specification
chlorophyll catabolite transmembrane transporter activity
cellular response to indolebutyric acid stimulus

UDP-xylosyltransferase activity
extracellular space
pollen germination
gametophyte development
nitrate transport
ammonium transmembrane transporter activity
glyoxylate cycle
plant-type hypersensitive response
phosphate ion transmembrane transport
germacradienol synthase activity
germacrene-D synthase activity
endocytosis
plant epidermis development
maintenance of seed dormancy
butyrate-CoA ligase activity
cell death
1-aminocyclopropane-1-carboxylate biosynthetic process
oxylipin biosynthetic process
histidine phosphotransfer kinase activity
stomatal complex morphogenesis
calcium channel activity
non-membrane spanning protein tyrosine kinase activity
rejection of self pollen
secondary active sulfate transmembrane transporter activity
cuticle development
detection of brassinosteroid stimulus
magnesium ion binding
cellular response to iron ion
motile cilium
response to cold
proanthocyanidin biosynthetic process
apical plasma membrane
protein phosphatase type 2A complex
phloem nitrate loading
innate immune response
transferase activity, transferring hexosyl groups
jasmonic acid and ethylene-dependent systemic resistance
oxidoreductase activity, acting on paired donors, with incorporation or reduction of molecular
13-hydroxylupinine O-tigloyltransferase activity
cellular copper ion homeostasis
response to temperature stimulus
arsenate ion transmembrane transporter activity
hydrogen-exporting ATPase activity, phosphorylative mechanism
lateral root formation
response to copper ion
galactosyltransferase activity
glucose mediated signaling pathway
microtubule-based process

endomembrane system organization
regulation of defense response to bacterium
methionine metabolic process
microtubule binding
high-affinity hydrogen:glucose symporter activity
hexose:proton symporter activity
regulation of membrane potential
phospholipase C activity
phosphatidylcholine phospholipase C activity
fatty acid transporter activity
regulation of stomatal movement
regulation of seedling development
polarity specification of adaxial/abaxial axis
inositol hexakisphosphate binding
diterpenoid biosynthetic process
defense response signaling pathway, resistance gene-independent
quercetin O-glucoside metabolic process
kaempferol O-glucoside metabolic process
lead ion transport
terpenoid transport
nucleotide transport
dipeptide transporter activity
tripeptide transporter activity
dipeptide transport
tripeptide transport
isoflavone 2'-hydroxylase activity
response to water deprivation
phosphatidylinositol phospholipase C activity
root meristem specification
cation transmembrane transport
endoplasmic reticulum membrane
radial microtubular system formation
glutathione S-conjugate-exporting ATPase activity
peptidyl-tyrosine phosphorylation
ion transport
1-aminocyclopropane-1-carboxylate synthase activity
regulation of floral meristem growth
carbohydrate homeostasis
xylan 1,4-beta-xylosidase activity
cadmium ion transport
inward rectifier potassium channel activity
voltage-gated potassium channel activity
malate dehydrogenase (decarboxylating) (NAD+) activity
phosphoprotein phosphatase activity
indole glucosinolate catabolic process
cellular response to aluminum ion
organic hydroxy compound transport
phospholipid translocation

p-coumaryl alcohol transport
plant-type primary cell wall biogenesis
racemase and epimerase activity, acting on carbohydrates and derivatives
response to cyclopentenone
ion channel activity
ATP-dependent helicase activity
negative regulation of programmed cell death
guanylate cyclase activity
cGMP biosynthetic process
stromule
phosphorylation
L-phenylalanine catabolic process
hydrolase activity, hydrolyzing O-glycosyl compounds
negative regulation of cell proliferation
UDP-glucose 6-dehydrogenase activity
UDP-glucuronate biosynthetic process
L-ascorbate oxidase activity
phragmoplast
actin filament binding
procambium histogenesis
transpiration
cation transport
anther wall tapetum cell differentiation
positive gravitropism
polyamine catabolic process
basal plasma membrane
pattern recognition receptor signaling pathway
maintenance of floral organ identity
iron ion homeostasis
xylogalacturonan metabolic process
fatty acid biosynthetic process
protein phosphatase type 2A regulator activity
cellular response to sulfate starvation
metabolic process
glucomannan 4-beta-mannosyltransferase activity
cellular response to nitrate
low-affinity nitrate transmembrane transporter activity
low-affinity nitrate transport
pollen tube
regulation of lignin biosynthetic process
glutamate receptor activity
cellular response to amino acid stimulus
galactose transmembrane transporter activity
integral component of Golgi membrane
tricarboxylic acid cycle
transcription regulatory region sequence-specific DNA binding
butyrate metabolic process
tau-protein kinase activity

ATP biosynthetic process
regulation of growth rate
positive regulation of organ growth
sulfate transmembrane transporter activity
cyclin-dependent protein serine/threonine kinase activity
3-oxoacyl-[acyl-carrier-protein] synthase activity
defense response
lipid transport
glucosinolate:proton symporter activity
phloem glucosinolate loading
glucosinolate transport
indole-3-butyrate beta-glucosyltransferase activity
xyloglucan biosynthetic process
secondary metabolite biosynthetic process
anthocyanidin 3-O-glucosyltransferase activity
response to nitrate
potassium ion import
malate metabolic process
potassium ion transmembrane transport
high-affinity secondary active ammonium transmembrane transporter activity
phosphatidylinositol phosphate kinase activity
secondary growth
developmental process involved in reproduction
induced systemic resistance, jasmonic acid mediated signaling pathway
mannosylation
regulation of meristem structural organization
phospholipid binding

Class
cellular_component
cellular_component
cellular_component
molecular_function
molecular_function
biological_process
molecular_function
biological_process
molecular_function
biological_process
molecular_function
cellular_component
molecular_function
molecular_function
molecular_function
molecular_function
biological_process
cellular_component
cellular_component
biological_process
cellular_component
biological_process
cellular_component
biological_process
molecular_function
cellular_component
molecular_function
biological_process
cellular_component
cellular_component
biological_process
biological_process
biological_process
biological_process
molecular_function
biological_process
biological_process
biological_process
molecular_function
molecular_function
biological_process
molecular_function
biological_process
molecular_function
biological_process
cellular_component
biological_process

biological_process
molecular_function
biological_process
cellular_component
biological_process
cellular_component
biological_process
molecular_function
molecular_function
molecular_function
molecular_function
biological_process
biological_process
biological_process
cellular_component
molecular_function
cellular_component
biological_process
cellular_component
molecular_function
biological_process
biological_process
biological_process
molecular_function
molecular_function
biological_process
molecular_function
biological_process
molecular_function
molecular_function
biological_process
molecular_function
biological_process
cellular_component
cellular_component
molecular_function
biological_process
biological_process
biological_process
molecular_function
biological_process
biological_process
cellular_component
molecular_function
biological_process
biological_process
molecular_function
molecular_function

biological_process
biological_process
biological_process
molecular_function
biological_process
biological_process
biological_process
biological_process
biological_process
molecular_function
cellular_component
biological_process
cellular_component
molecular_function
molecular_function
biological_process
biological_process
biological_process
biological_process
biological_process
biological_process
biological_process
molecular_function
biological_process
molecular_function
biological_process
biological_process
molecular_function
biological_process
biological_process
molecular_function
molecular_function
biological_process
biological_process
biological_process
molecular_function
biological_process
biological_process
molecular_function
biological_process
biological_process
molecular_function
molecular_function
biological_process
molecular_function
molecular_function
biological_process
biological_process

molecular_function
molecular_function
biological_process
molecular_function
biological_process
cellular_component
molecular_function
biological_process
molecular_function
biological_process
biological_process
molecular_function
biological_process
biological_process
biological_process
molecular_function
biological_process
biological_process
molecular_function
molecular_function
biological_process
molecular_function
molecular_function
molecular_function
biological_process
biological_process
cellular_component
cellular_component
biological_process
biological_process
molecular_function
biological_process
biological_process
biological_process
molecular_function
biological_process
molecular_function
molecular_function
cellular_component
molecular_function
biological_process
biological_process
molecular_function
molecular_function
biological_process
molecular_function
biological_process
biological_process

biological_process
biological_process
molecular_function
molecular_function
biological_process
biological_process
biological_process
molecular_function
biological_process
biological_process
biological_process
biological_process
biological_process
molecular_function
molecular_function
biological_process
cellular_component
biological_process
molecular_function
biological_process
biological_process
biological_process
biological_process
molecular_function
molecular_function
biological_process
biological_process
biological_process
biological_process
biological_process
molecular_function
biological_process
biological_process
cellular_component
biological_process
cellular_component
biological_process
biological_process
molecular_function
molecular_function
molecular_function
biological_process
biological_process
biological_process
biological_process
biological_process
molecular_function
biological_process

molecular_function
cellular_component
biological_process
biological_process
biological_process
molecular_function
biological_process
biological_process
biological_process
molecular_function
molecular_function
biological_process
biological_process
biological_process
molecular_function
biological_process
biological_process
biological_process
molecular_function
biological_process
molecular_function
molecular_function
biological_process
molecular_function
biological_process
biological_process
molecular_function
biological_process
cellular_component
biological_process
biological_process
cellular_component
cellular_component
biological_process
biological_process
molecular_function
biological_process
molecular_function
molecular_function
biological_process
biological_process
molecular_function
molecular_function
biological_process
biological_process
molecular_function
biological_process
biological_process

biological_process
biological_process
biological_process
molecular_function
molecular_function
molecular_function
biological_process
molecular_function
molecular_function
molecular_function
biological_process
biological_process
biological_process
molecular_function
biological_process
biological_process
biological_process
biological_process
biological_process
biological_process
biological_process
molecular_function
molecular_function
biological_process
biological_process
molecular_function
biological_process
molecular_function
biological_process
biological_process
cellular_component
biological_process
molecular_function
biological_process
biological_process
molecular_function
biological_process
biological_process
molecular_function
biological_process
molecular_function
molecular_function
molecular_function
molecular_function
biological_process
biological_process
biological_process
biological_process

biological_process
biological_process
molecular_function
biological_process
molecular_function
molecular_function
biological_process
molecular_function
biological_process
cellular_component
biological_process
biological_process
molecular_function
biological_process
molecular_function
biological_process
molecular_function
cellular_component
molecular_function
biological_process
biological_process
biological_process
biological_process
biological_process
biological_process
cellular_component
biological_process
biological_process
biological_process
biological_process
biological_process
molecular_function
biological_process
biological_process
molecular_function
biological_process
molecular_function
biological_process
cellular_component
biological_process
molecular_function
biological_process
molecular_function
cellular_component
biological_process
molecular_function
biological_process
molecular_function

biological_process
biological_process
biological_process
molecular_function
molecular_function
molecular_function
biological_process
biological_process
molecular_function
biological_process
biological_process
molecular_function
biological_process
biological_process
molecular_function
biological_process
biological_process
biological_process
biological_process
molecular_function
molecular_function
biological_process
biological_process
biological_process
biological_process
biological_process
molecular_function

Gene ID

Maole_000080.T1, Maole_000100.T1, Maole_000104.T1, Maole_000162.T1, Maole_000211.T1, Maole_00
Maole_000042.T1, Maole_000059.T1, Maole_000080.T1, Maole_000100.T1, Maole_000132.T1, Maole_00
Maole_000026.T1, Maole_000059.T1, Maole_000065.T1, Maole_000080.T1, Maole_000100.T1, Maole_00
Maole_000080.T1, Maole_000100.T1, Maole_000328.T1, Maole_000379.T1, Maole_000381.T1, Maole_00
Maole_000026.T1, Maole_000080.T1, Maole_000100.T1, Maole_000110.T1, Maole_000116.T1, Maole_00
Maole_000080.T1, Maole_000100.T1, Maole_000379.T1, Maole_000473.T1, Maole_000519.T1, Maole_00
Maole_000080.T1, Maole_000100.T1, Maole_000379.T1, Maole_000473.T1, Maole_000519.T1, Maole_00
Maole_000080.T1, Maole_000100.T1, Maole_000379.T1, Maole_000519.T1, Maole_000690.T1, Maole_00
Maole_000080.T1, Maole_000100.T1, Maole_000379.T1, Maole_000473.T1, Maole_000519.T1, Maole_00
Maole_000080.T1, Maole_000100.T1, Maole_000379.T1, Maole_000473.T1, Maole_000519.T1, Maole_00
Maole_000080.T1, Maole_000100.T1, Maole_000379.T1, Maole_000473.T1, Maole_000518.T1, Maole_00
Maole_000099.T1, Maole_000132.T1, Maole_000311.T1, Maole_000328.T1, Maole_000493.T1, Maole_00
Maole_000080.T1, Maole_000100.T1, Maole_000328.T1, Maole_000455.T1, Maole_000473.T1, Maole_00
Maole_000065.T1, Maole_000080.T1, Maole_000100.T1, Maole_000517.T1, Maole_000518.T1, Maole_00
Maole_000080.T1, Maole_000100.T1, Maole_000517.T1, Maole_000518.T1, Maole_000519.T1, Maole_00
Maole_000211.T1, Maole_001403.T1, Maole_001533.T1, Maole_001538.T1, Maole_002313.T1, Maole_00
Maole_000698.T1, Maole_001017.T1, Maole_001137.T1, Maole_001631.T1, Maole_002396.T1, Maole_00
Maole_000311.T1, Maole_000390.T1, Maole_000747.T1, Maole_000749.T1, Maole_000806.T1, Maole_00
Maole_000390.T1, Maole_000465.T1, Maole_000558.T1, Maole_000803.T1, Maole_000806.T1, Maole_00
Maole_000080.T1, Maole_000100.T1, Maole_000517.T1, Maole_000518.T1, Maole_000519.T1, Maole_00
Maole_000059.T1, Maole_000384.T1, Maole_000473.T1, Maole_000735.T1, Maole_000886.T1, Maole_00
Maole_000400.T1, Maole_000474.T1, Maole_000613.T1, Maole_000747.T1, Maole_000749.T1, Maole_00
Maole_000059.T1, Maole_000104.T1, Maole_000474.T1, Maole_000922.T1, Maole_001108.T1, Maole_00
Maole_002742.T1, Maole_005388.T1, Maole_006437.T1, Maole_006773.T1, Maole_006774.T1, Maole_00
Maole_000473.T1, Maole_001301.T1, Maole_001631.T1, Maole_001757.T1, Maole_002721.T1, Maole_00
Maole_000026.T1, Maole_000059.T1, Maole_000384.T1, Maole_000474.T1, Maole_000497.T1, Maole_00
Maole_000104.T1, Maole_000455.T1, Maole_000747.T1, Maole_000749.T1, Maole_001432.T1, Maole_00
Maole_001237.T1, Maole_001426.T1, Maole_001561.T1, Maole_002240.T1, Maole_002389.T1, Maole_00
Maole_000065.T1, Maole_000311.T1, Maole_000465.T1, Maole_000612.T1, Maole_000619.T1, Maole_00
Maole_000059.T1, Maole_000384.T1, Maole_000735.T1, Maole_000922.T1, Maole_001108.T1, Maole_00
Maole_000432.T1, Maole_000465.T1, Maole_000473.T1, Maole_000497.T1, Maole_000613.T1, Maole_00
Maole_000285.T1, Maole_000473.T1, Maole_000737.T1, Maole_000806.T1, Maole_001020.T1, Maole_00
Maole_001019.T1, Maole_001020.T1, Maole_001021.T1, Maole_001022.T1, Maole_001931.T1, Maole_00
Maole_002588.T1, Maole_002636.T1, Maole_002645.T1, Maole_002648.T1, Maole_003245.T1, Maole_00
Maole_002124.T1, Maole_002411.T1, Maole_003085.T1, Maole_004215.T1, Maole_004810.T1, Maole_00
Maole_000104.T1, Maole_000311.T1, Maole_000432.T1, Maole_000473.T1, Maole_000844.T1, Maole_00
Maole_001432.T1, Maole_004047.T1, Maole_004980.T1, Maole_004982.T1, Maole_005925.T1, Maole_00
Maole_001848.T1, Maole_002556.T1, Maole_002862.T1, Maole_003000.T1, Maole_004301.T1, Maole_00
Maole_001538.T1, Maole_002816.T1, Maole_003203.T1, Maole_006386.T1, Maole_006896.T1, Maole_00
Maole_000026.T1, Maole_000636.T1, Maole_001019.T1, Maole_001020.T1, Maole_001021.T1, Maole_00
Maole_002102.T1, Maole_004972.T1, Maole_006779.T1, Maole_009317.T1, Maole_009320.T1, Maole_01
Maole_002588.T1, Maole_002636.T1, Maole_002645.T1, Maole_002648.T1, Maole_003245.T1, Maole_00
Maole_000104.T1, Maole_000432.T1, Maole_000473.T1, Maole_001106.T1, Maole_001610.T1, Maole_00
Maole_001301.T1, Maole_001631.T1, Maole_002777.T1, Maole_002782.T1, Maole_003283.T1, Maole_00
Maole_000411.T1, Maole_000473.T1, Maole_001301.T1, Maole_001631.T1, Maole_001879.T1, Maole_00
Maole_000065.T1, Maole_000099.T1, Maole_000311.T1, Maole_000643.T1, Maole_000644.T1, Maole_00
Maole_002461.T1, Maole_003601.T1, Maole_003602.T1, Maole_003604.T1, Maole_003605.T1, Maole_00

Maole_001017.T1, Maole_001137.T1, Maole_002747.T1, Maole_005388.T1, Maole_007462.T1, Maole_00
Maole_000390.T1, Maole_001096.T1, Maole_001545.T1, Maole_001931.T1, Maole_004980.T1, Maole_00
Maole_004972.T1, Maole_006779.T1, Maole_009317.T1, Maole_009320.T1, Maole_014265.T1, Maole_01
Maole_000121.T1, Maole_000304.T1, Maole_000416.T1, Maole_000490.T1, Maole_000564.T1, Maole_00
Maole_001123.T1, Maole_001124.T1, Maole_001125.T1, Maole_001210.T1, Maole_001277.T1, Maole_00
Maole_000390.T1, Maole_000806.T1, Maole_001096.T1, Maole_001404.T1, Maole_001854.T1, Maole_00
Maole_001301.T1, Maole_001631.T1, Maole_002950.T1, Maole_006885.T1, Maole_009289.T1, Maole_00
Maole_000473.T1, Maole_000724.T1, Maole_001017.T1, Maole_001126.T1, Maole_001380.T1, Maole_00
Maole_000698.T1, Maole_002191.T1, Maole_002469.T1, Maole_002782.T1, Maole_005818.T1, Maole_00
Maole_000390.T1, Maole_001096.T1, Maole_001545.T1, Maole_001931.T1, Maole_004982.T1, Maole_00
Maole_002411.T1, Maole_003085.T1, Maole_004215.T1, Maole_005654.T1, Maole_005846.T1, Maole_00
Maole_000390.T1, Maole_001317.T1, Maole_001390.T1, Maole_001432.T1, Maole_001722.T1, Maole_00
Maole_001301.T1, Maole_001631.T1, Maole_002469.T1, Maole_002777.T1, Maole_002782.T1, Maole_00
Maole_002056.T1, Maole_002627.T1, Maole_003562.T1, Maole_006933.T1, Maole_007270.T1, Maole_00
Maole_000099.T1, Maole_000945.T1, Maole_001948.T1, Maole_002221.T1, Maole_002411.T1, Maole_00
Maole_000065.T1, Maole_001106.T1, Maole_001209.T1, Maole_001210.T1, Maole_004167.T1, Maole_00
Maole_000211.T1, Maole_001533.T1, Maole_001610.T1, Maole_002313.T1, Maole_002477.T1, Maole_00
Maole_002747.T1, Maole_003593.T1, Maole_004156.T1, Maole_008959.T1, Maole_011670.T1, Maole_01
Maole_000473.T1, Maole_000886.T1, Maole_001108.T1, Maole_001301.T1, Maole_001488.T1, Maole_00
Maole_002556.T1, Maole_002862.T1, Maole_004301.T1, Maole_006692.T1, Maole_006693.T1, Maole_00
Maole_000099.T1, Maole_000285.T1, Maole_001020.T1, Maole_001021.T1, Maole_001022.T1, Maole_00
Maole_000738.T1, Maole_001163.T1, Maole_002401.T1, Maole_002591.T1, Maole_002950.T1, Maole_00
Maole_000498.T1, Maole_001277.T1, Maole_002411.T1, Maole_003085.T1, Maole_004215.T1, Maole_00
Maole_000497.T1, Maole_001360.T1, Maole_002136.T1, Maole_002783.T1, Maole_005453.T1, Maole_00
Maole_000328.T1, Maole_005388.T1, Maole_007462.T1, Maole_009364.T1, Maole_009509.T1, Maole_00
Maole_000747.T1, Maole_000749.T1, Maole_002591.T1, Maole_003203.T1, Maole_005013.T1, Maole_00
Maole_005388.T1, Maole_007462.T1, Maole_009364.T1, Maole_009509.T1, Maole_009644.T1, Maole_01
Maole_000099.T1, Maole_000390.T1, Maole_000672.T1, Maole_000737.T1, Maole_000932.T1, Maole_00
Maole_006692.T1, Maole_006693.T1, Maole_006694.T1, Maole_006695.T1, Maole_009102.T1, Maole_01
Maole_006692.T1, Maole_006693.T1, Maole_006694.T1, Maole_006695.T1, Maole_009102.T1, Maole_01
Maole_000379.T1, Maole_000381.T1, Maole_001017.T1, Maole_001091.T1, Maole_001137.T1, Maole_00
Maole_001229.T1, Maole_001301.T1, Maole_001631.T1, Maole_003204.T1, Maole_004697.T1, Maole_00
Maole_000432.T1, Maole_000473.T1, Maole_002239.T1, Maole_002556.T1, Maole_003232.T1, Maole_00
Maole_000311.T1, Maole_000313.T1, Maole_000465.T1, Maole_002239.T1, Maole_003232.T1, Maole_00
Maole_000065.T1, Maole_000311.T1, Maole_000313.T1, Maole_000465.T1, Maole_000672.T1, Maole_00
Maole_004582.T1, Maole_006036.T1, Maole_006829.T1, Maole_006830.T1, Maole_009242.T1, Maole_01
Maole_000619.T1, Maole_001375.T1, Maole_001481.T1, Maole_001561.T1, Maole_002003.T1, Maole_00
Maole_000432.T1, Maole_000473.T1, Maole_002239.T1, Maole_002556.T1, Maole_003232.T1, Maole_00
Maole_000065.T1, Maole_000465.T1, Maole_001106.T1, Maole_001123.T1, Maole_001124.T1, Maole_00
Maole_000162.T1, Maole_000384.T1, Maole_000418.T1, Maole_000922.T1, Maole_000995.T1, Maole_00
Maole_000060.T1, Maole_001538.T1, Maole_001907.T1, Maole_002389.T1, Maole_002556.T1, Maole_00
Maole_000432.T1, Maole_000473.T1, Maole_002239.T1, Maole_002556.T1, Maole_003232.T1, Maole_00
Maole_000432.T1, Maole_000687.T1, Maole_002202.T1, Maole_002742.T1, Maole_004230.T1, Maole_00
Maole_002461.T1, Maole_002779.T1, Maole_002780.T1, Maole_003283.T1, Maole_003557.T1, Maole_00
Maole_001631.T1, Maole_001757.T1, Maole_002762.T1, Maole_003560.T1, Maole_003905.T1, Maole_00
Maole_005385.T1, Maole_009883.T1, Maole_012520.T1, Maole_012595.T1, Maole_012596.T1, Maole_01
Maole_002411.T1, Maole_004215.T1, Maole_005654.T1, Maole_005846.T1, Maole_008020.T1, Maole_00
Maole_006692.T1, Maole_006693.T1, Maole_006694.T1, Maole_006695.T1, Maole_015626.T1, Maole_02

Maole_006692.T1, Maole_006693.T1, Maole_006694.T1, Maole_006695.T1, Maole_009102.T1, Maole_01
Maole_002591.T1, Maole_006581.T1, Maole_006583.T1, Maole_007599.T1, Maole_009063.T1, Maole_00
Maole_001126.T1, Maole_001161.T1, Maole_002043.T1, Maole_002689.T1, Maole_003437.T1, Maole_00
Maole_000162.T1, Maole_000270.T1, Maole_000328.T1, Maole_000418.T1, Maole_000886.T1, Maole_00
Maole_000162.T1, Maole_000418.T1, Maole_000995.T1, Maole_005925.T1, Maole_007391.T1, Maole_00
Maole_001561.T1, Maole_001907.T1, Maole_002297.T1, Maole_002365.T1, Maole_002389.T1, Maole_00
Maole_003601.T1, Maole_003602.T1, Maole_003604.T1, Maole_003605.T1, Maole_003607.T1, Maole_00
Maole_001277.T1, Maole_003007.T1, Maole_003101.T1, Maole_009725.T1, Maole_015802.T1, Maole_01
Maole_002689.T1, Maole_004880.T1, Maole_005453.T1, Maole_008447.T1, Maole_008616.T1, Maole_01
Maole_000211.T1, Maole_001533.T1, Maole_002510.T1, Maole_003989.T1, Maole_004942.T1, Maole_01
Maole_000806.T1, Maole_001120.T1, Maole_001432.T1, Maole_002390.T1, Maole_002591.T1, Maole_00
Maole_001422.T1, Maole_002411.T1, Maole_002826.T1, Maole_003085.T1, Maole_004215.T1, Maole_00
Maole_000026.T1, Maole_000636.T1, Maole_000687.T1, Maole_001935.T1, Maole_001957.T1, Maole_00
Maole_006693.T1, Maole_009102.T1, Maole_012082.T1, Maole_016686.T1, Maole_019159.T1, Maole_01
Maole_002406.T1, Maole_006066.T1, Maole_007409.T1, Maole_012828.T1, Maole_014831.T1, Maole_01
Maole_002556.T1, Maole_002862.T1, Maole_004301.T1, Maole_006692.T1, Maole_006693.T1, Maole_00
Maole_002707.T1, Maole_003886.T1, Maole_006692.T1, Maole_006693.T1, Maole_006694.T1, Maole_00
Maole_000099.T1, Maole_000490.T1, Maole_000643.T1, Maole_000644.T1, Maole_000870.T1, Maole_00
Maole_006692.T1, Maole_006693.T1, Maole_006694.T1, Maole_006695.T1, Maole_015626.T1, Maole_02
Maole_004980.T1, Maole_004982.T1, Maole_008447.T1, Maole_011110.T1, Maole_011111.T1, Maole_01
Maole_001037.T1, Maole_001488.T1, Maole_007143.T1, Maole_007145.T1, Maole_008181.T1, Maole_01
Maole_001538.T1, Maole_002401.T1, Maole_003203.T1, Maole_006386.T1, Maole_006896.T1, Maole_00
Maole_001013.T1, Maole_001120.T1, Maole_002207.T1, Maole_004430.T1, Maole_004565.T1, Maole_00
Maole_002288.T1, Maole_002831.T1, Maole_002854.T1, Maole_003557.T1, Maole_003905.T1, Maole_00
Maole_002627.T1, Maole_003562.T1, Maole_006933.T1, Maole_007270.T1, Maole_007272.T1, Maole_00
Maole_001356.T1, Maole_001853.T1, Maole_002102.T1, Maole_002221.T1, Maole_003656.T1, Maole_00
Maole_000432.T1, Maole_000497.T1, Maole_000687.T1, Maole_002202.T1, Maole_003315.T1, Maole_00
Maole_007599.T1, Maole_009063.T1, Maole_009534.T1, Maole_011321.T1, Maole_011322.T1, Maole_01
Maole_001019.T1, Maole_001020.T1, Maole_001021.T1, Maole_001022.T1, Maole_001277.T1, Maole_00
Maole_000379.T1, Maole_000381.T1, Maole_000473.T1, Maole_001757.T1, Maole_002102.T1, Maole_00
Maole_002136.T1, Maole_002689.T1, Maole_006085.T1, Maole_008616.T1, Maole_010934.T1, Maole_01
Maole_000698.T1, Maole_001025.T1, Maole_001533.T1, Maole_001631.T1, Maole_001639.T1, Maole_00
Maole_000285.T1, Maole_000619.T1, Maole_001561.T1, Maole_002240.T1, Maole_002689.T1, Maole_00
Maole_000121.T1, Maole_001071.T1, Maole_001123.T1, Maole_001124.T1, Maole_001125.T1, Maole_00
Maole_001120.T1, Maole_002816.T1, Maole_004003.T1, Maole_007749.T1, Maole_011444.T1, Maole_01
Maole_000474.T1, Maole_001013.T1, Maole_001120.T1, Maole_002207.T1, Maole_004430.T1, Maole_00
Maole_000612.T1, Maole_000619.T1, Maole_001481.T1, Maole_001757.T1, Maole_002236.T1, Maole_00
Maole_002461.T1, Maole_003283.T1, Maole_003557.T1, Maole_003560.T1, Maole_005013.T1, Maole_00
Maole_017648.T1, Maole_017866.T1, Maole_017867.T1, Maole_017868.T1, Maole_017870.T1, Maole_01
Maole_000211.T1, Maole_001467.T1, Maole_001533.T1, Maole_002313.T1, Maole_002510.T1, Maole_00
Maole_001277.T1, Maole_003007.T1, Maole_015027.T1, Maole_019303.T1, Maole_022244.T1, Maole_02
Maole_001020.T1, Maole_003007.T1, Maole_009336.T1, Maole_012596.T1, Maole_012598.T1, Maole_02
Maole_000806.T1, Maole_002899.T1, Maole_005633.T1, Maole_005935.T1, Maole_013454.T1, Maole_01
Maole_002950.T1, Maole_003848.T1, Maole_005388.T1, Maole_012448.T1, Maole_012507.T1, Maole_01
Maole_004819.T1, Maole_018863.T1, Maole_018864.T1, Maole_019762.T1, Maole_019764.T1, Maole_01
Maole_000211.T1, Maole_001533.T1, Maole_002313.T1, Maole_002510.T1, Maole_003000.T1, Maole_00
Maole_000211.T1, Maole_001533.T1, Maole_002313.T1, Maole_002510.T1, Maole_003000.T1, Maole_00
Maole_000211.T1, Maole_001533.T1, Maole_002313.T1, Maole_002510.T1, Maole_003000.T1, Maole_00

Maole_001136.T1, Maole_001730.T1, Maole_005514.T1, Maole_012276.T1, Maole_012537.T1, Maole_01
Maole_000311.T1, Maole_000313.T1, Maole_000672.T1, Maole_002239.T1, Maole_002588.T1, Maole_00
Maole_001120.T1, Maole_001286.T1, Maole_001288.T1, Maole_001919.T1, Maole_001920.T1, Maole_00
Maole_002141.T1, Maole_002146.T1, Maole_004819.T1, Maole_009724.T1, Maole_011052.T1, Maole_01
Maole_000026.T1, Maole_001404.T1, Maole_001488.T1, Maole_001608.T1, Maole_002704.T1, Maole_00
Maole_003848.T1, Maole_004268.T1, Maole_011987.T1, Maole_012448.T1, Maole_013030.T1, Maole_01
Maole_003848.T1, Maole_004268.T1, Maole_011987.T1, Maole_012448.T1, Maole_013030.T1, Maole_01
Maole_003848.T1, Maole_004268.T1, Maole_011987.T1, Maole_012448.T1, Maole_013030.T1, Maole_01
Maole_001286.T1, Maole_001288.T1, Maole_001920.T1, Maole_002029.T1, Maole_003601.T1, Maole_00
Maole_001100.T1, Maole_001616.T1, Maole_008809.T1, Maole_009636.T1, Maole_016612.T1, Maole_01
Maole_001432.T1, Maole_001610.T1, Maole_001616.T1, Maole_001854.T1, Maole_004862.T1, Maole_00
Maole_000613.T1, Maole_002235.T1, Maole_003315.T1, Maole_004142.T1, Maole_004721.T1, Maole_00
Maole_000432.T1, Maole_000497.T1, Maole_001100.T1, Maole_001826.T1, Maole_004927.T1, Maole_00
Maole_004230.T1, Maole_004972.T1, Maole_009317.T1, Maole_009320.T1, Maole_014265.T1, Maole_01
Maole_001020.T1, Maole_001021.T1, Maole_001022.T1, Maole_002761.T1, Maole_003602.T1, Maole_00
Maole_000411.T1, Maole_000498.T1, Maole_001534.T1, Maole_001535.T1, Maole_002003.T1, Maole_00
Maole_000390.T1, Maole_002707.T1, Maole_002862.T1, Maole_003886.T1, Maole_004301.T1, Maole_00
Maole_001286.T1, Maole_001288.T1, Maole_001919.T1, Maole_001920.T1, Maole_003601.T1, Maole_00
Maole_000211.T1, Maole_001533.T1, Maole_002313.T1, Maole_002510.T1, Maole_003084.T1, Maole_00
Maole_000162.T1, Maole_000418.T1, Maole_000995.T1, Maole_007391.T1, Maole_008670.T1, Maole_00
Maole_000400.T1, Maole_002056.T1, Maole_002707.T1, Maole_003886.T1, Maole_006692.T1, Maole_00
Maole_001017.T1, Maole_004246.T1, Maole_005326.T1, Maole_006670.T1, Maole_006766.T1, Maole_00
Maole_000473.T1, Maole_002721.T1, Maole_002724.T1, Maole_006040.T1, Maole_011930.T1, Maole_01
Maole_001616.T1, Maole_005612.T1, Maole_008809.T1, Maole_009636.T1, Maole_016612.T1, Maole_01
Maole_000613.T1, Maole_002235.T1, Maole_003315.T1, Maole_004142.T1, Maole_004721.T1, Maole_00
Maole_000099.T1, Maole_000643.T1, Maole_000644.T1, Maole_000870.T1, Maole_001037.T1, Maole_00
Maole_000902.T1, Maole_002742.T1, Maole_003056.T1, Maole_004073.T1, Maole_005925.T1, Maole_01
Maole_000311.T1, Maole_000313.T1, Maole_000465.T1, Maole_000932.T1, Maole_002239.T1, Maole_00
Maole_002505.T1, Maole_004814.T1, Maole_007923.T1, Maole_011216.T1, Maole_018279.T1, Maole_02
Maole_004916.T1, Maole_004918.T1, Maole_005101.T1, Maole_006559.T1, Maole_013416.T1, Maole_01
Maole_006693.T1, Maole_009102.T1, Maole_019159.T1, Maole_020423.T1, Maole_022732.T1, Maole_02
Maole_006693.T1, Maole_009102.T1, Maole_019159.T1, Maole_020423.T1, Maole_022732.T1, Maole_02
Maole_001017.T1, Maole_001137.T1, Maole_002747.T1, Maole_003848.T1, Maole_004268.T1, Maole_00
Maole_006779.T1, Maole_015116.T1, Maole_021416.T1, Maole_023259.T1, Maole_023260.T1, Maole_02
Maole_002556.T1, Maole_004982.T1, Maole_006799.T1, Maole_011813.T1, Maole_013561.T1, Maole_01
Maole_002556.T1, Maole_004982.T1, Maole_006799.T1, Maole_011813.T1, Maole_013561.T1, Maole_01
Maole_001286.T1, Maole_001288.T1, Maole_001919.T1, Maole_001920.T1, Maole_003601.T1, Maole_00
Maole_001286.T1, Maole_001288.T1, Maole_001919.T1, Maole_001920.T1, Maole_003601.T1, Maole_00
Maole_001561.T1, Maole_002693.T1, Maole_006351.T1, Maole_009038.T1, Maole_012805.T1, Maole_01
Maole_004980.T1, Maole_004982.T1, Maole_011110.T1, Maole_011111.T1, Maole_011159.T1, Maole_01
Maole_004980.T1, Maole_004982.T1, Maole_011110.T1, Maole_011111.T1, Maole_011159.T1, Maole_01
Maole_000132.T1, Maole_000326.T1, Maole_001093.T1, Maole_001516.T1, Maole_002241.T1, Maole_00
Maole_000211.T1, Maole_001533.T1, Maole_002313.T1, Maole_002510.T1, Maole_003000.T1, Maole_00
Maole_007386.T1, Maole_007927.T1, Maole_010760.T1, Maole_010762.T1, Maole_010763.T1, Maole_01
Maole_000379.T1, Maole_000381.T1, Maole_001757.T1, Maole_002102.T1, Maole_002433.T1, Maole_00
Maole_006693.T1, Maole_009102.T1, Maole_012845.T1, Maole_020423.T1, Maole_022732.T1, Maole_02
Maole_006693.T1, Maole_009102.T1, Maole_012845.T1, Maole_020423.T1, Maole_022732.T1, Maole_02
Maole_001286.T1, Maole_001288.T1, Maole_010554.T1, Maole_011514.T1, Maole_011533.T1, Maole_01

Maole_000026.T1, Maole_004136.T1, Maole_004457.T1, Maole_004750.T1, Maole_011642.T1, Maole_01
Maole_006693.T1, Maole_009102.T1, Maole_020423.T1, Maole_022732.T1, Maole_022733.T1, Maole_02
Maole_006693.T1, Maole_009102.T1, Maole_020423.T1, Maole_022732.T1, Maole_022733.T1, Maole_02
Maole_006693.T1, Maole_009102.T1, Maole_020423.T1, Maole_022732.T1, Maole_022733.T1, Maole_02
Maole_006693.T1, Maole_009102.T1, Maole_020423.T1, Maole_022732.T1, Maole_022733.T1, Maole_02
Maole_006693.T1, Maole_009102.T1, Maole_020423.T1, Maole_022732.T1, Maole_022733.T1, Maole_02
Maole_000803.T1, Maole_009962.T1, Maole_016181.T1, Maole_016914.T1, Maole_018122.T1, Maole_01
Maole_000803.T1, Maole_009962.T1, Maole_016181.T1, Maole_016914.T1, Maole_018122.T1, Maole_01
Maole_000473.T1, Maole_001725.T1, Maole_005301.T1, Maole_005925.T1, Maole_006040.T1, Maole_00
Maole_000497.T1, Maole_001360.T1, Maole_002136.T1, Maole_002783.T1, Maole_003315.T1, Maole_00
Maole_001616.T1, Maole_001879.T1, Maole_003326.T1, Maole_006095.T1, Maole_007723.T1, Maole_00
Maole_001403.T1, Maole_015757.T1, Maole_015759.T1, Maole_015760.T1, Maole_015762.T1, Maole_01
Maole_000839.T1, Maole_000932.T1, Maole_001310.T1, Maole_001458.T1, Maole_001907.T1, Maole_00
Maole_008291.T1, Maole_014706.T1, Maole_014707.T1, Maole_014708.T1, Maole_020182.T1
Maole_004255.T1, Maole_005335.T1, Maole_008961.T1, Maole_012008.T1, Maole_014209.T1, Maole_01
Maole_003557.T1, Maole_003905.T1, Maole_007894.T1, Maole_011163.T1, Maole_019167.T1, Maole_01
Maole_000886.T1, Maole_001286.T1, Maole_001288.T1, Maole_001919.T1, Maole_001920.T1, Maole_00
Maole_001277.T1, Maole_002596.T1, Maole_003007.T1, Maole_009725.T1, Maole_011813.T1, Maole_01
Maole_007143.T1, Maole_007145.T1, Maole_011377.T1, Maole_011378.T1, Maole_020605.T1, Maole_02
Maole_001907.T1, Maole_001918.T1, Maole_003496.T1, Maole_003920.T1, Maole_004554.T1, Maole_00
Maole_000959.T1, Maole_001093.T1, Maole_001100.T1, Maole_001516.T1, Maole_001971.T1, Maole_00
Maole_002510.T1, Maole_010427.T1, Maole_011595.T1, Maole_011678.T1, Maole_016696.T1, Maole_02
Maole_002693.T1, Maole_005101.T1, Maole_006559.T1, Maole_009038.T1, Maole_009043.T1, Maole_01
Maole_006391.T1, Maole_011709.T1, Maole_015629.T1, Maole_017109.T1, Maole_018437.T1
Maole_002556.T1, Maole_006799.T1, Maole_009289.T1, Maole_010021.T1, Maole_022187.T1, Maole_02
Maole_000285.T1, Maole_001310.T1, Maole_001561.T1, Maole_002389.T1, Maole_002408.T1, Maole_00
Maole_000379.T1, Maole_002191.T1, Maole_002288.T1, Maole_003283.T1, Maole_003994.T1, Maole_00
Maole_000211.T1, Maole_000474.T1, Maole_001533.T1, Maole_002264.T1, Maole_002313.T1, Maole_00
Maole_000803.T1, Maole_009962.T1, Maole_014592.T1, Maole_018122.T1, Maole_018123.T1, Maole_01
Maole_002874.T1, Maole_004885.T1, Maole_006080.T1, Maole_007956.T1, Maole_011880.T1, Maole_01
Maole_007035.T1, Maole_009796.T1, Maole_016082.T1, Maole_020896.T1
Maole_000612.T1, Maole_000619.T1, Maole_001481.T1, Maole_005625.T1, Maole_005626.T1, Maole_00
Maole_007386.T1, Maole_007927.T1, Maole_010760.T1, Maole_010762.T1, Maole_013630.T1, Maole_01
Maole_002505.T1, Maole_007910.T1, Maole_011216.T1, Maole_014702.T1, Maole_018279.T1, Maole_02
Maole_001108.T1, Maole_004072.T1, Maole_005633.T1, Maole_007690.T1, Maole_013454.T1, Maole_01
Maole_000026.T1, Maole_000381.T1, Maole_000490.T1, Maole_000564.T1, Maole_000569.T1, Maole_00
Maole_002389.T1, Maole_002556.T1, Maole_003905.T1, Maole_004878.T1, Maole_004982.T1, Maole_00
Maole_002276.T1, Maole_004874.T1, Maole_005229.T1, Maole_023171.T1, Maole_024003.T1
Maole_001100.T1, Maole_001616.T1, Maole_008809.T1, Maole_009636.T1, Maole_016612.T1, Maole_01
Maole_001100.T1, Maole_001616.T1, Maole_008809.T1, Maole_009636.T1, Maole_016612.T1, Maole_01
Maole_007386.T1, Maole_007927.T1, Maole_010763.T1, Maole_017544.T1, Maole_022398.T1
Maole_000473.T1, Maole_001631.T1, Maole_002777.T1, Maole_002782.T1, Maole_003326.T1, Maole_00
Maole_002558.T1, Maole_004409.T1, Maole_005654.T1, Maole_008946.T1, Maole_010457.T1, Maole_01
Maole_004916.T1, Maole_004918.T1, Maole_013416.T1, Maole_018524.T1, Maole_022120.T1
Maole_003007.T1, Maole_015027.T1, Maole_022244.T1, Maole_022842.T1, Maole_022844.T1
Maole_002469.T1, Maole_002782.T1, Maole_007101.T1, Maole_007703.T1, Maole_009317.T1, Maole_01
Maole_007944.T1, Maole_013780.T1, Maole_020501.T1, Maole_020502.T1, Maole_022100.T1
Maole_001020.T1, Maole_001021.T1, Maole_001022.T1, Maole_011288.T1, Maole_017673.T1

Maole_003148.T1, Maole_005232.T1, Maole_013116.T1, Maole_013119.T1, Maole_013926.T1, Maole_01
Maole_000311.T1, Maole_000313.T1, Maole_000803.T1, Maole_001404.T1, Maole_002276.T1, Maole_00
Maole_000738.T1, Maole_002136.T1, Maole_002950.T1, Maole_003322.T1, Maole_003592.T1, Maole_00
Maole_000026.T1, Maole_003557.T1, Maole_004136.T1, Maole_004750.T1, Maole_011163.T1, Maole_01
Maole_001538.T1, Maole_001971.T1, Maole_002365.T1, Maole_002816.T1, Maole_003000.T1, Maole_00
Maole_000400.T1, Maole_002056.T1, Maole_002707.T1, Maole_016665.T1
Maole_005071.T1, Maole_008489.T1, Maole_008800.T1, Maole_009587.T1, Maole_014829.T1, Maole_01
Maole_000724.T1, Maole_002381.T1, Maole_002477.T1, Maole_003356.T1, Maole_003886.T1, Maole_00
Maole_001403.T1, Maole_001971.T1, Maole_002264.T1, Maole_003978.T1, Maole_007342.T1, Maole_00
Maole_010832.T1, Maole_013319.T1, Maole_013321.T1, Maole_017586.T1, Maole_023762.T1, Maole_02
Maole_010832.T1, Maole_013319.T1, Maole_013321.T1, Maole_017586.T1, Maole_023762.T1, Maole_02
Maole_000738.T1, Maole_000886.T1, Maole_001017.T1, Maole_001137.T1, Maole_001639.T1, Maole_00
Maole_010336.T1, Maole_012100.T1, Maole_014337.T1, Maole_015667.T1, Maole_020436.T1, Maole_02
Maole_001120.T1, Maole_007749.T1, Maole_018033.T1, Maole_018034.T1, Maole_018035.T1, Maole_01
Maole_005058.T1, Maole_005059.T1, Maole_009587.T1, Maole_014829.T1
Maole_001561.T1, Maole_001631.T1, Maole_002408.T1, Maole_002693.T1, Maole_002777.T1, Maole_00
Maole_008122.T1, Maole_017911.T1, Maole_023406.T1, Maole_023943.T1
Maole_000121.T1, Maole_001422.T1, Maole_002003.T1, Maole_006203.T1, Maole_007386.T1, Maole_00
Maole_001025.T1, Maole_001826.T1, Maole_005101.T1, Maole_006559.T1, Maole_013870.T1, Maole_01
Maole_000379.T1, Maole_000381.T1, Maole_000440.T1, Maole_000473.T1, Maole_000690.T1, Maole_00
Maole_001424.T1, Maole_004073.T1, Maole_005877.T1, Maole_005880.T1, Maole_005935.T1, Maole_00
Maole_002461.T1, Maole_004238.T1, Maole_004602.T1, Maole_006670.T1, Maole_007126.T1, Maole_00
Maole_000518.T1, Maole_000519.T1, Maole_014480.T1, Maole_019406.T1, Maole_019409.T1, Maole_01
Maole_004916.T1, Maole_004918.T1, Maole_013416.T1, Maole_016575.T1, Maole_016576.T1, Maole_01
Maole_000864.T1, Maole_002411.T1, Maole_003085.T1, Maole_004881.T1, Maole_005846.T1, Maole_00
Maole_000473.T1, Maole_006040.T1, Maole_011930.T1, Maole_017922.T1, Maole_020499.T1, Maole_02
Maole_000870.T1, Maole_001038.T1, Maole_001136.T1, Maole_001467.T1, Maole_001730.T1, Maole_00
Maole_002693.T1, Maole_007391.T1, Maole_007455.T1, Maole_008703.T1, Maole_009038.T1, Maole_01
Maole_004972.T1, Maole_014265.T1, Maole_014267.T1, Maole_014269.T1, Maole_014270.T1, Maole_01
Maole_000643.T1, Maole_000644.T1, Maole_001316.T1, Maole_001422.T1, Maole_001616.T1, Maole_00
Maole_001120.T1, Maole_001467.T1, Maole_001469.T1, Maole_007749.T1, Maole_010114.T1, Maole_01
Maole_000738.T1, Maole_002596.T1, Maole_004798.T1, Maole_005343.T1, Maole_007590.T1, Maole_01
Maole_001091.T1, Maole_002389.T1, Maole_002874.T1, Maole_004885.T1, Maole_007956.T1, Maole_00
Maole_017085.T1, Maole_017086.T1, Maole_017087.T1, Maole_017092.T1
Maole_001424.T1, Maole_001749.T1, Maole_001757.T1, Maole_001879.T1, Maole_002304.T1, Maole_00
Maole_001286.T1, Maole_001288.T1, Maole_001919.T1, Maole_001920.T1, Maole_003601.T1, Maole_00
Maole_004457.T1, Maole_013450.T1, Maole_017660.T1, Maole_017900.T1, Maole_023885.T1
Maole_000498.T1, Maole_001534.T1, Maole_001535.T1, Maole_004412.T1, Maole_004771.T1, Maole_00
Maole_004810.T1, Maole_015819.T1, Maole_019477.T1, Maole_019478.T1
Maole_001108.T1, Maole_004072.T1, Maole_007427.T1, Maole_007690.T1, Maole_016925.T1, Maole_02
Maole_006933.T1, Maole_007143.T1, Maole_007145.T1, Maole_008706.T1, Maole_008854.T1, Maole_01
Maole_015757.T1, Maole_015759.T1, Maole_015760.T1, Maole_015762.T1, Maole_016010.T1
Maole_001467.T1, Maole_001469.T1, Maole_004003.T1, Maole_011444.T1, Maole_016457.T1, Maole_01
Maole_000211.T1, Maole_001533.T1, Maole_002056.T1, Maole_002556.T1, Maole_004942.T1, Maole_00
Maole_002636.T1, Maole_002648.T1, Maole_004626.T1, Maole_005110.T1, Maole_006703.T1, Maole_00
Maole_001227.T1, Maole_003589.T1, Maole_013231.T1, Maole_013988.T1, Maole_014629.T1, Maole_01
Maole_001037.T1, Maole_008181.T1, Maole_011198.T1, Maole_014370.T1, Maole_018100.T1, Maole_02
Maole_002627.T1, Maole_003562.T1, Maole_004598.T1, Maole_006933.T1, Maole_007270.T1, Maole_00

Maole_000384.T1, Maole_001488.T1, Maole_007183.T1, Maole_017220.T1, Maole_017719.T1, Maole_02
Maole_001561.T1, Maole_002693.T1, Maole_009038.T1, Maole_012805.T1, Maole_013299.T1, Maole_02
Maole_002874.T1, Maole_004885.T1, Maole_007956.T1, Maole_011880.T1, Maole_012004.T1, Maole_02
Maole_000636.T1, Maole_001957.T1, Maole_002202.T1, Maole_004598.T1, Maole_004704.T1, Maole_00
Maole_011678.T1, Maole_016696.T1, Maole_021905.T1, Maole_023851.T1
Maole_011678.T1, Maole_016696.T1, Maole_021905.T1, Maole_023851.T1
Maole_001610.T1, Maole_004073.T1, Maole_004862.T1, Maole_010308.T1, Maole_012375.T1, Maole_01
Maole_001516.T1, Maole_004385.T1, Maole_016666.T1, Maole_020979.T1
Maole_001516.T1, Maole_004385.T1, Maole_016666.T1, Maole_020979.T1
Maole_001277.T1, Maole_003007.T1, Maole_018563.T1, Maole_019303.T1, Maole_022842.T1
Maole_001467.T1, Maole_002389.T1, Maole_005195.T1, Maole_006936.T1, Maole_007427.T1, Maole_00
Maole_000473.T1, Maole_001631.T1, Maole_002777.T1, Maole_002782.T1, Maole_006040.T1, Maole_00
Maole_002191.T1, Maole_002288.T1, Maole_002854.T1, Maole_004874.T1, Maole_004880.T1, Maole_00
Maole_009289.T1, Maole_010021.T1, Maole_022187.T1, Maole_022607.T1
Maole_012497.T1, Maole_012499.T1, Maole_012501.T1, Maole_013616.T1, Maole_016086.T1, Maole_01
Maole_003356.T1, Maole_006211.T1, Maole_006779.T1, Maole_015116.T1, Maole_017452.T1, Maole_02
Maole_004819.T1, Maole_018863.T1, Maole_018864.T1, Maole_019766.T1, Maole_019767.T1
Maole_004819.T1, Maole_018863.T1, Maole_018864.T1, Maole_019766.T1, Maole_019767.T1
Maole_012520.T1, Maole_012595.T1, Maole_012596.T1, Maole_012598.T1, Maole_016455.T1
Maole_012520.T1, Maole_012595.T1, Maole_012596.T1, Maole_012598.T1, Maole_016455.T1
Maole_002707.T1, Maole_003886.T1, Maole_006692.T1, Maole_006693.T1, Maole_006694.T1, Maole_00
Maole_000747.T1, Maole_001986.T1, Maole_001987.T1, Maole_002401.T1, Maole_002591.T1, Maole_00
Maole_000747.T1, Maole_001986.T1, Maole_001987.T1, Maole_002401.T1, Maole_002591.T1, Maole_00
Maole_000747.T1, Maole_001986.T1, Maole_001987.T1, Maole_002401.T1, Maole_002591.T1, Maole_00
Maole_000747.T1, Maole_001986.T1, Maole_001987.T1, Maole_002401.T1, Maole_002591.T1, Maole_00
Maole_018465.T1, Maole_019629.T1, Maole_019630.T1, Maole_021407.T1, Maole_021422.T1, Maole_02
Maole_000411.T1, Maole_001432.T1, Maole_001467.T1, Maole_001561.T1, Maole_001610.T1, Maole_00
Maole_006773.T1, Maole_006774.T1, Maole_009992.T1, Maole_022983.T1, Maole_022984.T1
Maole_010505.T1, Maole_015631.T1, Maole_019167.T1, Maole_019514.T1, Maole_021520.T1
Maole_001469.T1, Maole_002761.T1, Maole_009880.T1, Maole_011357.T1, Maole_011358.T1, Maole_01
Maole_000059.T1, Maole_000498.T1, Maole_000672.T1, Maole_000856.T1, Maole_001011.T1, Maole_00
Maole_000026.T1, Maole_004136.T1, Maole_004750.T1, Maole_011642.T1, Maole_019320.T1
Maole_007944.T1, Maole_013780.T1, Maole_020501.T1, Maole_020502.T1, Maole_022100.T1
Maole_001017.T1, Maole_004246.T1, Maole_006766.T1, Maole_009038.T1, Maole_010336.T1, Maole_01
Maole_001108.T1, Maole_002750.T1, Maole_003498.T1, Maole_004072.T1, Maole_004444.T1, Maole_00
Maole_004480.T1, Maole_008122.T1, Maole_017911.T1, Maole_023406.T1, Maole_023943.T1
Maole_002779.T1, Maole_002780.T1, Maole_011163.T1, Maole_014267.T1, Maole_014592.T1, Maole_02
Maole_005101.T1, Maole_006559.T1, Maole_008775.T1, Maole_017644.T1, Maole_019939.T1, Maole_02
Maole_002406.T1, Maole_006066.T1, Maole_011085.T1, Maole_012828.T1, Maole_015464.T1, Maole_01
Maole_001020.T1, Maole_001021.T1, Maole_001022.T1, Maole_001120.T1, Maole_002207.T1, Maole_00
Maole_001610.T1, Maole_004862.T1, Maole_012375.T1, Maole_014228.T1, Maole_016473.T1, Maole_02
Maole_001610.T1, Maole_004073.T1, Maole_004862.T1, Maole_010308.T1, Maole_012375.T1, Maole_01
Maole_000963.T1, Maole_001023.T1, Maole_020426.T1, Maole_021181.T1
Maole_000473.T1, Maole_002458.T1, Maole_002721.T1, Maole_003813.T1, Maole_004051.T1, Maole_00
Maole_001020.T1, Maole_001021.T1, Maole_001022.T1, Maole_002761.T1
Maole_001316.T1, Maole_006031.T1, Maole_014828.T1, Maole_023361.T1
Maole_001019.T1, Maole_001020.T1, Maole_001021.T1, Maole_001022.T1
Maole_005514.T1, Maole_012276.T1, Maole_012537.T1, Maole_015061.T1

Maole_001019.T1, Maole_001020.T1, Maole_001021.T1, Maole_001022.T1
Maole_000474.T1, Maole_000497.T1, Maole_005453.T1, Maole_008706.T1, Maole_008707.T1, Maole_02
Maole_007035.T1, Maole_009796.T1, Maole_016082.T1, Maole_020896.T1
Maole_002461.T1, Maole_003496.T1, Maole_011642.T1, Maole_011691.T1, Maole_011692.T1, Maole_01
Maole_001610.T1, Maole_004073.T1, Maole_010308.T1, Maole_017476.T1, Maole_020187.T1, Maole_02
Maole_001380.T1, Maole_002505.T1, Maole_006176.T1, Maole_007910.T1, Maole_007923.T1, Maole_00
Maole_000806.T1, Maole_002707.T1, Maole_003886.T1, Maole_005344.T1, Maole_006356.T1, Maole_00
Maole_002304.T1, Maole_006651.T1, Maole_006652.T1, Maole_007101.T1, Maole_007703.T1, Maole_00
Maole_002304.T1, Maole_006651.T1, Maole_006652.T1, Maole_007101.T1, Maole_007703.T1, Maole_00
Maole_000643.T1, Maole_000644.T1, Maole_001422.T1, Maole_002634.T1, Maole_005005.T1, Maole_00
Maole_001025.T1, Maole_001826.T1, Maole_004457.T1, Maole_005940.T1, Maole_011211.T1, Maole_01
Maole_008291.T1, Maole_014706.T1, Maole_014707.T1, Maole_014708.T1, Maole_020182.T1, Maole_02
Maole_000465.T1, Maole_001123.T1, Maole_001124.T1, Maole_001125.T1, Maole_001609.T1, Maole_00
Maole_001038.T1, Maole_005505.T1, Maole_008868.T1, Maole_010293.T1, Maole_013647.T1, Maole_01
Maole_003773.T1, Maole_012353.T1, Maole_012766.T1
Maole_003773.T1, Maole_012353.T1, Maole_012766.T1
Maole_000311.T1, Maole_003232.T1, Maole_010356.T1, Maole_013216.T1, Maole_016506.T1, Maole_02
Maole_000026.T1, Maole_000432.T1, Maole_000636.T1, Maole_000687.T1, Maole_001907.T1, Maole_00
Maole_003610.T1, Maole_005152.T1, Maole_005343.T1, Maole_005612.T1, Maole_014517.T1, Maole_01
Maole_000379.T1, Maole_000381.T1, Maole_002288.T1, Maole_003283.T1, Maole_003994.T1, Maole_00
Maole_001037.T1, Maole_002191.T1, Maole_005818.T1, Maole_008181.T1, Maole_011198.T1, Maole_01
Maole_002761.T1, Maole_005816.T1, Maole_009880.T1, Maole_011357.T1, Maole_011358.T1, Maole_01
Maole_000473.T1, Maole_006040.T1, Maole_011930.T1, Maole_017214.T1, Maole_020499.T1, Maole_02
Maole_000902.T1, Maole_002556.T1, Maole_005024.T1, Maole_005343.T1, Maole_005421.T1, Maole_00
Maole_001538.T1, Maole_004167.T1, Maole_008424.T1, Maole_008734.T1, Maole_009465.T1, Maole_01
Maole_006437.T1, Maole_011128.T1, Maole_012819.T1, Maole_014835.T1, Maole_015354.T1, Maole_01
Maole_001879.T1, Maole_003326.T1, Maole_009303.T1, Maole_011953.T1, Maole_017166.T1, Maole_01
Maole_005064.T1, Maole_012785.T1, Maole_015343.T1, Maole_017992.T1, Maole_018063.T1, Maole_02
Maole_002750.T1, Maole_003498.T1, Maole_004444.T1, Maole_004445.T1, Maole_006712.T1, Maole_00
Maole_003148.T1, Maole_013116.T1, Maole_013119.T1, Maole_013926.T1, Maole_017124.T1
Maole_001023.T1, Maole_001551.T1, Maole_001553.T1, Maole_001992.T1, Maole_002411.T1, Maole_00
Maole_001091.T1, Maole_002389.T1, Maole_002874.T1, Maole_004885.T1, Maole_007956.T1, Maole_01
Maole_011211.T1, Maole_013416.T1, Maole_013449.T1, Maole_016271.T1, Maole_016575.T1, Maole_01
Maole_000026.T1, Maole_000285.T1, Maole_000416.T1, Maole_000636.T1, Maole_002028.T1, Maole_00
Maole_002689.T1, Maole_008616.T1, Maole_017313.T1, Maole_020684.T1, Maole_022720.T1
Maole_009289.T1, Maole_010021.T1, Maole_010599.T1, Maole_022187.T1
Maole_013147.T1, Maole_013149.T1, Maole_017086.T1, Maole_019768.T1, Maole_019770.T1, Maole_02
Maole_013147.T1, Maole_013149.T1, Maole_017086.T1, Maole_019768.T1, Maole_019770.T1, Maole_02
Maole_000211.T1, Maole_000738.T1, Maole_001533.T1, Maole_002950.T1, Maole_003000.T1, Maole_00
Maole_001019.T1, Maole_001020.T1, Maole_001021.T1, Maole_001022.T1, Maole_002818.T1
Maole_001424.T1, Maole_005877.T1, Maole_005880.T1, Maole_007827.T1, Maole_012162.T1, Maole_01
Maole_001424.T1, Maole_005877.T1, Maole_005880.T1, Maole_007827.T1, Maole_012162.T1, Maole_01
Maole_000211.T1, Maole_001533.T1, Maole_004942.T1, Maole_013121.T1, Maole_022760.T1
Maole_000384.T1, Maole_003066.T1, Maole_006085.T1, Maole_007183.T1, Maole_010423.T1, Maole_01
Maole_003030.T1, Maole_004117.T1, Maole_005071.T1, Maole_008489.T1, Maole_008800.T1, Maole_01
Maole_002728.T1, Maole_003249.T1, Maole_005064.T1, Maole_012785.T1, Maole_013186.T1
Maole_005058.T1, Maole_005059.T1, Maole_009587.T1, Maole_014829.T1
Maole_001137.T1, Maole_002747.T1, Maole_004878.T1, Maole_007074.T1, Maole_023299.T1

Maole_001467.T1, Maole_001469.T1, Maole_004003.T1, Maole_011444.T1, Maole_017089.T1, Maole_02
Maole_007894.T1, Maole_019219.T1, Maole_022820.T1
Maole_007894.T1, Maole_019219.T1, Maole_022820.T1
Maole_004916.T1, Maole_004918.T1, Maole_013416.T1, Maole_018524.T1, Maole_022120.T1
Maole_001703.T1, Maole_001859.T1, Maole_003901.T1, Maole_008890.T1, Maole_010735.T1, Maole_01
Maole_003221.T1, Maole_006203.T1, Maole_007633.T1, Maole_014142.T1, Maole_014250.T1
Maole_000465.T1, Maole_000473.T1, Maole_000636.T1, Maole_001419.T1, Maole_001879.T1, Maole_00
Maole_001229.T1, Maole_001277.T1, Maole_003204.T1, Maole_003543.T1, Maole_005344.T1, Maole_00
Maole_013149.T1, Maole_017085.T1, Maole_017086.T1, Maole_017087.T1, Maole_017092.T1
Maole_013149.T1, Maole_017085.T1, Maole_017086.T1, Maole_017087.T1, Maole_017092.T1
Maole_013149.T1, Maole_017085.T1, Maole_017086.T1, Maole_017087.T1, Maole_017092.T1
Maole_003601.T1, Maole_003602.T1, Maole_003604.T1, Maole_003605.T1, Maole_003607.T1, Maole_01
Maole_000384.T1, Maole_005244.T1, Maole_007183.T1, Maole_007656.T1, Maole_014553.T1, Maole_01
Maole_005983.T1, Maole_007894.T1, Maole_012497.T1, Maole_012499.T1, Maole_012501.T1, Maole_01
Maole_001919.T1, Maole_001920.T1, Maole_006469.T1, Maole_007394.T1, Maole_010914.T1, Maole_01
Maole_001538.T1, Maole_001971.T1, Maole_002365.T1, Maole_002816.T1, Maole_003203.T1, Maole_00
Maole_001610.T1, Maole_001854.T1, Maole_004862.T1, Maole_010016.T1, Maole_012342.T1, Maole_01
Maole_000963.T1, Maole_001023.T1, Maole_004117.T1, Maole_008489.T1, Maole_016016.T1, Maole_02
Maole_001432.T1, Maole_001610.T1, Maole_004073.T1, Maole_004862.T1, Maole_010308.T1, Maole_01
Maole_000400.T1, Maole_002056.T1, Maole_002707.T1
Maole_000738.T1, Maole_001488.T1, Maole_001649.T1, Maole_005343.T1, Maole_017719.T1, Maole_02
Maole_005101.T1, Maole_006559.T1, Maole_018459.T1, Maole_023357.T1
Maole_005343.T1, Maole_009522.T1, Maole_012022.T1, Maole_012405.T1, Maole_020854.T1
Maole_001424.T1, Maole_004554.T1, Maole_007320.T1, Maole_007827.T1, Maole_012162.T1, Maole_01
Maole_002136.T1, Maole_002689.T1, Maole_006085.T1, Maole_008616.T1, Maole_010934.T1, Maole_01
Maole_002779.T1, Maole_002780.T1, Maole_003283.T1, Maole_003557.T1, Maole_003560.T1, Maole_00
Maole_004627.T1, Maole_005307.T1, Maole_005630.T1, Maole_006702.T1, Maole_008292.T1, Maole_00

0256.T1, Maole_000311.T1, Maole_000313.T1, Maole_000328.T1, Maole_000379.T1, Mao
0162.T1, Maole_000198.T1, Maole_000211.T1, Maole_000270.T1, Maole_000379.T1, Mao
0162.T1, Maole_000311.T1, Maole_000313.T1, Maole_000379.T1, Maole_000400.T1, Mao
0473.T1, Maole_000517.T1, Maole_000518.T1, Maole_000519.T1, Maole_000690.T1, Mao
0328.T1, Maole_000379.T1, Maole_000381.T1, Maole_000390.T1, Maole_000416.T1, Mao
0690.T1, Maole_000698.T1, Maole_000893.T1, Maole_001017.T1, Maole_001097.T1, Mao
0690.T1, Maole_000698.T1, Maole_000893.T1, Maole_001097.T1, Maole_001301.T1, Mao
0698.T1, Maole_000839.T1, Maole_000893.T1, Maole_001097.T1, Maole_001375.T1, Mao
0690.T1, Maole_000698.T1, Maole_000893.T1, Maole_001097.T1, Maole_001301.T1, Mao
0690.T1, Maole_000698.T1, Maole_000893.T1, Maole_001097.T1, Maole_001301.T1, Mao
0519.T1, Maole_000690.T1, Maole_000698.T1, Maole_000893.T1, Maole_001097.T1, Mao
0497.T1, Maole_000619.T1, Maole_000643.T1, Maole_000644.T1, Maole_000690.T1, Mao
0517.T1, Maole_000518.T1, Maole_000519.T1, Maole_000690.T1, Maole_000698.T1, Mao
0519.T1, Maole_000839.T1, Maole_001008.T1, Maole_001106.T1, Maole_001209.T1, Mao
0806.T1, Maole_002899.T1, Maole_004432.T1, Maole_005388.T1, Maole_005935.T1, Mao
2510.T1, Maole_002556.T1, Maole_002862.T1, Maole_003203.T1, Maole_003989.T1, Mao
2742.T1, Maole_002747.T1, Maole_002777.T1, Maole_002782.T1, Maole_004457.T1, Mao
0934.T1, Maole_000945.T1, Maole_001020.T1, Maole_001021.T1, Maole_001022.T1, Mao
0934.T1, Maole_001037.T1, Maole_001096.T1, Maole_001237.T1, Maole_001467.T1, Mao
4432.T1, Maole_006992.T1, Maole_007381.T1, Maole_007382.T1, Maole_007383.T1, Mao
0922.T1, Maole_001108.T1, Maole_001488.T1, Maole_001566.T1, Maole_001609.T1, Mao
0806.T1, Maole_001432.T1, Maole_001481.T1, Maole_001931.T1, Maole_001986.T1, Mao
1137.T1, Maole_001227.T1, Maole_001356.T1, Maole_001512.T1, Maole_001514.T1, Mao
7462.T1, Maole_007590.T1, Maole_007834.T1, Maole_008596.T1, Maole_009067.T1, Mao
2724.T1, Maole_002777.T1, Maole_002782.T1, Maole_002950.T1, Maole_002955.T1, Mao
0558.T1, Maole_000672.T1, Maole_000806.T1, Maole_000921.T1, Maole_000922.T1, Mao
1538.T1, Maole_001722.T1, Maole_001986.T1, Maole_001987.T1, Maole_002264.T1, Mao
2693.T1, Maole_002707.T1, Maole_003227.T1, Maole_003813.T1, Maole_004304.T1, Mao
0643.T1, Maole_000644.T1, Maole_000864.T1, Maole_000870.T1, Maole_000934.T1, Mao
1561.T1, Maole_001566.T1, Maole_001609.T1, Maole_001971.T1, Maole_002136.T1, Mao
0922.T1, Maole_001360.T1, Maole_001992.T1, Maole_002136.T1, Maole_002235.T1, Mao
1021.T1, Maole_001022.T1, Maole_001316.T1, Maole_001422.T1, Maole_001879.T1, Mao
4881.T1, Maole_004982.T1, Maole_011288.T1, Maole_011401.T1, Maole_012520.T1, Mao
4210.T1, Maole_004626.T1, Maole_006703.T1, Maole_010114.T1, Maole_010115.T1, Mao
5654.T1, Maole_005846.T1, Maole_006203.T1, Maole_007633.T1, Maole_008020.T1, Mao
1426.T1, Maole_002239.T1, Maole_002556.T1, Maole_003232.T1, Maole_003905.T1, Mao
6437.T1, Maole_008596.T1, Maole_011110.T1, Maole_011111.T1, Maole_011128.T1, Mao
6692.T1, Maole_006693.T1, Maole_006694.T1, Maole_006695.T1, Maole_006799.T1, Mao
9465.T1, Maole_010599.T1, Maole_011873.T1, Maole_014059.T1, Maole_017085.T1, Mao
1022.T1, Maole_001277.T1, Maole_001467.T1, Maole_001469.T1, Maole_001878.T1, Mao
2158.T1, Maole_014265.T1, Maole_014267.T1, Maole_014269.T1, Maole_014270.T1, Mao
4210.T1, Maole_004626.T1, Maole_006703.T1, Maole_010114.T1, Maole_010115.T1, Mao
2239.T1, Maole_002556.T1, Maole_003232.T1, Maole_003905.T1, Maole_004189.T1, Mao
3326.T1, Maole_003557.T1, Maole_003560.T1, Maole_006885.T1, Maole_009320.T1, Mao
2070.T1, Maole_002652.T1, Maole_002762.T1, Maole_002777.T1, Maole_002782.T1, Mao
1106.T1, Maole_001123.T1, Maole_001124.T1, Maole_001125.T1, Maole_001209.T1, Mao
3607.T1, Maole_005101.T1, Maole_006559.T1, Maole_007973.T1, Maole_009509.T1, Mao

7590.T1, Maole_009364.T1, Maole_009509.T1, Maole_009644.T1, Maole_011710.T1, Mao
4982.T1, Maole_005385.T1, Maole_007944.T1, Maole_011110.T1, Maole_011111.T1, Mao
4267.T1, Maole_014269.T1, Maole_014270.T1, Maole_014272.T1, Maole_014274.T1, Mao
0636.T1, Maole_000643.T1, Maole_000644.T1, Maole_000687.T1, Maole_000724.T1, Mao
1432.T1, Maole_002056.T1, Maole_002146.T1, Maole_002862.T1, Maole_003007.T1, Mao
1931.T1, Maole_002240.T1, Maole_002591.T1, Maole_003227.T1, Maole_003283.T1, Mao
9320.T1, Maole_009432.T1, Maole_010021.T1, Maole_010110.T1, Maole_010336.T1, Mao
1610.T1, Maole_001631.T1, Maole_002381.T1, Maole_002505.T1, Maole_003065.T1, Mao
6779.T1, Maole_007101.T1, Maole_007703.T1, Maole_009317.T1, Maole_011589.T1, Mao
7944.T1, Maole_011159.T1, Maole_011357.T1, Maole_011358.T1, Maole_011401.T1, Mao
8020.T1, Maole_009817.T1, Maole_016461.T1, Maole_016463.T1, Maole_016467.T1, Mao
1947.T1, Maole_002264.T1, Maole_003027.T1, Maole_003543.T1, Maole_003978.T1, Mao
2792.T1, Maole_003326.T1, Maole_003848.T1, Maole_006885.T1, Maole_007101.T1, Mao
7272.T1, Maole_009100.T1, Maole_009213.T1, Maole_010479.T1, Maole_011181.T1, Mao
2620.T1, Maole_003656.T1, Maole_003976.T1, Maole_004139.T1, Maole_004215.T1, Mao
6194.T1, Maole_006295.T1, Maole_008763.T1, Maole_010083.T1, Maole_011832.T1, Mao
2510.T1, Maole_003000.T1, Maole_003084.T1, Maole_003989.T1, Maole_004073.T1, Mao
2116.T1, Maole_013130.T1, Maole_014477.T1, Maole_014488.T1, Maole_017155.T1, Mao
1566.T1, Maole_001631.T1, Maole_003987.T1, Maole_004072.T1, Maole_004407.T1, Mao
6694.T1, Maole_006695.T1, Maole_006799.T1, Maole_007939.T1, Maole_009029.T1, Mao
1277.T1, Maole_001310.T1, Maole_001426.T1, Maole_001467.T1, Maole_001610.T1, Mao
3000.T1, Maole_003848.T1, Maole_004073.T1, Maole_004430.T1, Maole_004695.T1, Mao
5654.T1, Maole_005846.T1, Maole_006444.T1, Maole_006445.T1, Maole_008020.T1, Mao
6085.T1, Maole_006235.T1, Maole_006236.T1, Maole_007631.T1, Maole_008706.T1, Mao
9644.T1, Maole_011710.T1, Maole_012158.T1, Maole_012507.T1, Maole_013112.T1, Mao
6581.T1, Maole_006583.T1, Maole_007599.T1, Maole_009063.T1, Maole_009534.T1, Mao
1710.T1, Maole_012158.T1, Maole_012507.T1, Maole_014602.T1, Maole_014641.T1, Mao
1277.T1, Maole_001310.T1, Maole_001610.T1, Maole_001854.T1, Maole_002003.T1, Mao
5626.T1, Maole_020423.T1, Maole_022732.T1, Maole_022733.T1, Maole_022737.T1
5626.T1, Maole_020423.T1, Maole_022732.T1, Maole_022733.T1, Maole_022737.T1
1757.T1, Maole_002102.T1, Maole_002742.T1, Maole_002747.T1, Maole_002874.T1, Mao
5064.T1, Maole_005344.T1, Maole_005531.T1, Maole_005534.T1, Maole_005535.T1, Mao
3905.T1, Maole_004230.T1, Maole_004334.T1, Maole_006799.T1, Maole_006933.T1, Mao
3369.T1, Maole_003426.T1, Maole_004896.T1, Maole_006485.T1, Maole_007024.T1, Mao
1106.T1, Maole_001108.T1, Maole_001210.T1, Maole_001404.T1, Maole_002239.T1, Mao
3596.T1, Maole_015073.T1, Maole_015516.T1, Maole_016278.T1, Maole_017277.T1, Mao
2433.T1, Maole_002693.T1, Maole_003848.T1, Maole_004457.T1, Maole_004880.T1, Mao
3905.T1, Maole_004230.T1, Maole_005232.T1, Maole_006799.T1, Maole_006933.T1, Mao
1125.T1, Maole_001209.T1, Maole_001210.T1, Maole_001609.T1, Maole_001700.T1, Mao
1201.T1, Maole_002689.T1, Maole_002818.T1, Maole_003148.T1, Maole_003152.T1, Mao
3203.T1, Maole_003496.T1, Maole_003754.T1, Maole_003813.T1, Maole_003920.T1, Mao
3905.T1, Maole_004230.T1, Maole_006790.T1, Maole_006799.T1, Maole_006933.T1, Mao
4704.T1, Maole_005307.T1, Maole_006574.T1, Maole_007898.T1, Maole_009038.T1, Mao
3560.T1, Maole_003994.T1, Maole_005013.T1, Maole_006688.T1, Maole_007322.T1, Mao
6885.T1, Maole_007703.T1, Maole_008182.T1, Maole_008868.T1, Maole_009099.T1, Mao
2598.T1, Maole_015802.T1, Maole_016455.T1, Maole_019968.T1, Maole_024010.T1
9817.T1, Maole_016461.T1, Maole_016463.T1, Maole_016467.T1, Maole_016855.T1, Mao
0423.T1, Maole_022732.T1, Maole_022733.T1, Maole_022737.T1

5626.T1, Maole_020423.T1, Maole_022732.T1, Maole_022733.T1, Maole_022737.T1
9534.T1, Maole_011321.T1, Maole_011322.T1, Maole_016343.T1, Maole_016345.T1, Mao
5911.T1, Maole_006168.T1, Maole_008616.T1, Maole_010257.T1, Maole_013332.T1, Mao
0995.T1, Maole_001100.T1, Maole_001375.T1, Maole_001616.T1, Maole_002003.T1, Mao
8670.T1, Maole_011601.T1, Maole_011761.T1, Maole_011764.T1, Maole_014580.T1, Mao
2408.T1, Maole_002693.T1, Maole_003444.T1, Maole_003754.T1, Maole_006095.T1, Mao
3920.T1, Maole_004237.T1, Maole_004874.T1, Maole_006311.T1, Maole_006471.T1, Mao
9177.T1, Maole_019303.T1, Maole_021844.T1, Maole_022842.T1, Maole_024010.T1
4867.T1, Maole_017163.T1, Maole_017313.T1, Maole_022720.T1, Maole_023419.T1, Mao
0427.T1, Maole_011595.T1, Maole_011678.T1, Maole_016696.T1, Maole_021905.T1, Mao
2816.T1, Maole_003084.T1, Maole_005483.T1, Maole_006351.T1, Maole_006386.T1, Mao
4554.T1, Maole_005654.T1, Maole_005846.T1, Maole_005902.T1, Maole_006203.T1, Mao
2202.T1, Maole_002627.T1, Maole_003562.T1, Maole_004136.T1, Maole_004156.T1, Mao
9300.T1, Maole_020423.T1, Maole_022732.T1, Maole_022733.T1, Maole_022737.T1
4832.T1, Maole_015464.T1, Maole_015610.T1, Maole_016115.T1
6694.T1, Maole_006695.T1, Maole_006799.T1, Maole_007939.T1, Maole_008831.T1, Mao
6695.T1, Maole_014397.T1, Maole_015626.T1, Maole_020423.T1, Maole_022732.T1, Mao
0932.T1, Maole_001310.T1, Maole_001422.T1, Maole_001485.T1, Maole_001538.T1, Mao
0423.T1, Maole_022732.T1, Maole_022733.T1, Maole_022737.T1
1159.T1, Maole_012819.T1, Maole_013561.T1, Maole_014744.T1, Maole_014867.T1, Mao
1198.T1, Maole_011377.T1, Maole_011378.T1, Maole_017719.T1, Maole_020605.T1, Mao
9465.T1, Maole_009883.T1, Maole_010599.T1, Maole_011873.T1, Maole_012405.T1, Mao
5543.T1, Maole_006169.T1, Maole_006681.T1, Maole_006682.T1, Maole_007455.T1, Mao
4167.T1, Maole_004878.T1, Maole_005102.T1, Maole_005432.T1, Maole_005520.T1, Mao
9100.T1, Maole_010479.T1, Maole_011181.T1, Maole_012665.T1, Maole_014746.T1, Mao
3754.T1, Maole_003805.T1, Maole_004874.T1, Maole_004972.T1, Maole_005408.T1, Mao
4334.T1, Maole_004457.T1, Maole_004704.T1, Maole_005453.T1, Maole_006574.T1, Mao
6343.T1, Maole_016345.T1, Maole_020028.T1, Maole_020657.T1
1578.T1, Maole_002495.T1, Maole_003007.T1, Maole_004156.T1, Maole_004881.T1, Mao
2707.T1, Maole_003283.T1, Maole_007074.T1, Maole_007101.T1, Maole_007504.T1, Mao
2144.T1, Maole_012145.T1, Maole_016155.T1, Maole_017313.T1, Maole_022720.T1
2469.T1, Maole_002634.T1, Maole_003557.T1, Maole_003596.T1, Maole_003994.T1, Mao
2693.T1, Maole_003227.T1, Maole_003498.T1, Maole_004444.T1, Maole_004445.T1, Mao
1277.T1, Maole_001310.T1, Maole_001424.T1, Maole_001931.T1, Maole_001971.T1, Mao
7089.T1, Maole_017128.T1, Maole_018033.T1, Maole_018034.T1, Maole_018035.T1, Mao
4565.T1, Maole_005543.T1, Maole_006169.T1, Maole_006681.T1, Maole_006682.T1, Mao
3560.T1, Maole_003905.T1, Maole_004255.T1, Maole_005335.T1, Maole_005625.T1, Mao
5101.T1, Maole_006559.T1, Maole_006688.T1, Maole_007590.T1, Maole_009505.T1, Mao
7871.T1, Maole_017874.T1
2618.T1, Maole_002924.T1, Maole_003000.T1, Maole_003084.T1, Maole_003989.T1, Mao
2842.T1, Maole_022844.T1
1844.T1, Maole_024010.T1
3456.T1, Maole_014963.T1, Maole_014964.T1, Maole_015044.T1, Maole_019510.T1, Mao
2857.T1, Maole_013030.T1, Maole_013163.T1, Maole_014161.T1, Maole_016444.T1, Mao
9766.T1, Maole_019767.T1
3084.T1, Maole_003989.T1, Maole_004942.T1, Maole_009968.T1, Maole_010427.T1, Mao
3084.T1, Maole_003989.T1, Maole_004942.T1, Maole_009968.T1, Maole_010427.T1, Mao
3084.T1, Maole_003989.T1, Maole_004942.T1, Maole_009968.T1, Maole_010427.T1, Mao

3721.T1, Maole_015061.T1
2636.T1, Maole_002645.T1, Maole_002648.T1, Maole_002680.T1, Maole_002761.T1, Mao
2124.T1, Maole_003601.T1, Maole_003602.T1, Maole_003604.T1, Maole_003605.T1, Mao
4080.T1, Maole_014832.T1, Maole_015610.T1, Maole_017223.T1, Maole_018863.T1, Mao
2715.T1, Maole_002728.T1, Maole_002884.T1, Maole_002899.T1, Maole_003249.T1, Mao
9253.T1, Maole_019256.T1, Maole_019257.T1
9253.T1, Maole_019256.T1, Maole_019257.T1
9253.T1, Maole_019256.T1, Maole_019257.T1
3602.T1, Maole_003604.T1, Maole_003605.T1, Maole_003607.T1, Maole_010554.T1, Mao
6977.T1, Maole_016978.T1, Maole_023496.T1, Maole_023771.T1
8809.T1, Maole_009636.T1, Maole_010016.T1, Maole_012342.T1, Maole_015677.T1, Mao
6480.T1, Maole_007285.T1, Maole_007473.T1, Maole_011207.T1, Maole_011872.T1, Mao
5453.T1, Maole_005574.T1, Maole_006203.T1, Maole_006315.T1, Maole_010425.T1, Mao
4267.T1, Maole_014269.T1, Maole_014270.T1, Maole_014272.T1, Maole_014274.T1, Mao
3848.T1, Maole_004972.T1, Maole_009317.T1, Maole_009320.T1, Maole_012093.T1, Mao
2070.T1, Maole_003218.T1, Maole_004412.T1, Maole_004771.T1, Maole_004939.T1, Mao
6692.T1, Maole_006693.T1, Maole_006694.T1, Maole_006695.T1, Maole_007939.T1, Mao
3602.T1, Maole_003604.T1, Maole_003605.T1, Maole_003607.T1, Maole_006469.T1, Mao
3989.T1, Maole_004942.T1, Maole_009968.T1, Maole_010427.T1, Maole_010920.T1, Mao
8854.T1, Maole_011601.T1, Maole_011761.T1, Maole_011764.T1, Maole_014580.T1, Mao
6693.T1, Maole_006694.T1, Maole_006695.T1, Maole_014397.T1, Maole_016665.T1
7126.T1, Maole_007170.T1, Maole_008105.T1, Maole_009038.T1, Maole_010336.T1, Mao
8021.T1, Maole_020499.T1, Maole_020500.T1, Maole_023561.T1, Maole_023562.T1, Mao
6977.T1, Maole_016978.T1, Maole_023496.T1
6480.T1, Maole_007285.T1, Maole_007473.T1, Maole_011207.T1, Maole_011872.T1, Mao
1240.T1, Maole_002028.T1, Maole_004139.T1, Maole_004787.T1, Maole_005372.T1, Mao
0308.T1, Maole_016032.T1, Maole_016358.T1, Maole_019075.T1, Maole_020187.T1, Mao
3100.T1, Maole_003232.T1, Maole_003369.T1, Maole_004896.T1, Maole_006485.T1, Mao
0321.T1
6271.T1, Maole_016575.T1, Maole_016576.T1, Maole_018524.T1, Maole_022120.T1, Mao
2733.T1, Maole_022737.T1
2733.T1, Maole_022737.T1
5514.T1, Maole_011987.T1, Maole_012448.T1, Maole_013030.T1, Maole_013130.T1, Mao
3776.T1, Maole_024056.T1, Maole_024121.T1
4744.T1, Maole_017309.T1, Maole_017713.T1
4744.T1, Maole_017309.T1, Maole_017713.T1
3602.T1, Maole_003604.T1, Maole_003605.T1, Maole_003607.T1, Maole_006469.T1, Mao
3602.T1, Maole_003604.T1, Maole_003605.T1, Maole_003607.T1, Maole_006469.T1, Mao
3299.T1, Maole_016166.T1, Maole_021727.T1
2819.T1, Maole_013561.T1, Maole_014744.T1, Maole_017309.T1, Maole_019453.T1, Mao
3561.T1, Maole_014744.T1, Maole_017309.T1, Maole_021185.T1, Maole_023076.T1
2477.T1, Maole_002558.T1, Maole_002634.T1, Maole_003226.T1, Maole_003813.T1, Mao
3084.T1, Maole_003989.T1, Maole_004942.T1, Maole_009968.T1, Maole_010427.T1, Mao
3630.T1, Maole_017544.T1, Maole_019799.T1, Maole_022398.T1
3283.T1, Maole_007074.T1, Maole_007504.T1, Maole_009096.T1, Maole_009099.T1, Mao
2733.T1, Maole_022737.T1
2733.T1, Maole_022737.T1
1536.T1, Maole_018810.T1, Maole_019308.T1, Maole_019310.T1, Maole_022414.T1

7660.T1, Maole_017900.T1, Maole_018513.T1, Maole_019320.T1
2737.T1
2737.T1
2737.T1
2737.T1
2737.T1
8123.T1, Maole_018139.T1
8123.T1, Maole_018139.T1
7602.T1, Maole_008868.T1, Maole_010241.T1, Maole_010293.T1, Maole_010920.T1, Mao
5453.T1, Maole_005607.T1, Maole_006085.T1, Maole_006235.T1, Maole_006236.T1, Mao
7826.T1, Maole_007979.T1, Maole_007982.T1, Maole_009303.T1, Maole_009636.T1, Mao
6010.T1
1986.T1, Maole_001987.T1, Maole_002288.T1, Maole_002408.T1, Maole_002433.T1, Mao

4584.T1, Maole_018577.T1, Maole_020639.T1, Maole_021894.T1
9219.T1, Maole_020819.T1, Maole_021520.T1, Maole_022090.T1, Maole_022471.T1, Mao
1968.T1, Maole_002003.T1, Maole_002950.T1, Maole_003601.T1, Maole_003602.T1, Mao
3408.T1, Maole_015802.T1, Maole_017713.T1, Maole_018533.T1, Maole_019177.T1, Mao
0610.T1, Maole_020826.T1, Maole_023519.T1, Maole_023523.T1
4874.T1, Maole_004880.T1, Maole_004982.T1, Maole_005024.T1, Maole_005664.T1, Mao
4164.T1, Maole_004385.T1, Maole_005101.T1, Maole_005276.T1, Maole_006315.T1, Mao
1905.T1, Maole_023851.T1
2805.T1, Maole_013299.T1, Maole_013870.T1, Maole_013950.T1, Maole_016971.T1, Mao

2607.T1
2433.T1, Maole_002693.T1, Maole_005221.T1, Maole_005344.T1, Maole_005421.T1, Mao
5818.T1, Maole_007320.T1, Maole_009099.T1, Maole_011589.T1, Maole_012467.T1, Mao
2510.T1, Maole_003084.T1, Maole_003978.T1, Maole_003989.T1, Maole_004942.T1, Mao
8139.T1, Maole_023259.T1, Maole_023260.T1, Maole_023776.T1, Maole_024056.T1, Mao
2004.T1, Maole_020919.T1

5709.T1, Maole_008264.T1, Maole_018644.T1, Maole_024085.T1
7544.T1
0321.T1
6657.T1, Maole_016925.T1, Maole_021285.T1, Maole_021718.T1
0643.T1, Maole_000644.T1, Maole_000737.T1, Maole_000738.T1, Maole_000870.T1, Mao
5520.T1, Maole_005796.T1, Maole_006799.T1, Maole_008706.T1, Maole_009248.T1, Mao

6977.T1, Maole_016978.T1, Maole_023496.T1, Maole_023771.T1
6977.T1, Maole_016978.T1, Maole_023496.T1, Maole_023771.T1

6040.T1, Maole_006885.T1, Maole_008854.T1, Maole_010110.T1, Maole_011930.T1, Mao
1380.T1, Maole_013709.T1, Maole_016687.T1, Maole_016855.T1, Maole_017228.T1, Mao

4516.T1, Maole_015735.T1, Maole_015737.T1, Maole_015738.T1, Maole_024056.T1

6237.T1, Maole_017124.T1
3232.T1, Maole_004161.T1, Maole_004164.T1, Maole_004251.T1, Maole_004697.T1, Mao
3848.T1, Maole_005152.T1, Maole_005741.T1, Maole_010601.T1, Maole_012144.T1, Mao
1642.T1, Maole_011716.T1, Maole_019320.T1, Maole_020819.T1, Maole_022090.T1, Mao
3203.T1, Maole_003978.T1, Maole_006386.T1, Maole_006896.T1, Maole_007342.T1, Mao

6525.T1, Maole_018783.T1, Maole_023290.T1
5012.T1, Maole_005902.T1, Maole_006211.T1, Maole_006356.T1, Maole_006539.T1, Mao
8874.T1, Maole_008875.T1, Maole_012283.T1, Maole_015759.T1, Maole_016981.T1
3849.T1
3849.T1
2747.T1, Maole_004627.T1, Maole_004673.T1, Maole_004714.T1, Maole_005198.T1, Mao
3262.T1
9820.T1, Maole_022419.T1, Maole_023245.T1

2782.T1, Maole_003886.T1, Maole_005005.T1, Maole_005012.T1, Maole_006539.T1, Mao

7757.T1, Maole_007758.T1, Maole_007927.T1, Maole_009921.T1, Maole_010760.T1, Mao
7331.T1, Maole_017679.T1, Maole_018459.T1, Maole_023357.T1
0698.T1, Maole_000803.T1, Maole_001757.T1, Maole_002102.T1, Maole_002191.T1, Mao
7827.T1, Maole_012162.T1, Maole_012375.T1, Maole_014199.T1, Maole_016541.T1, Mao
8105.T1, Maole_009096.T1, Maole_009918.T1, Maole_010383.T1, Maole_011885.T1, Mao
9412.T1, Maole_019422.T1
8524.T1, Maole_022120.T1
6203.T1, Maole_007360.T1, Maole_009025.T1, Maole_009817.T1, Maole_014142.T1, Mao
3564.T1, Maole_023753.T1
1836.T1, Maole_002742.T1, Maole_004334.T1, Maole_005071.T1, Maole_005514.T1, Mao
2554.T1, Maole_012805.T1, Maole_013299.T1, Maole_015202.T1, Maole_017911.T1, Mao
4272.T1, Maole_014274.T1, Maole_014275.T1
1968.T1, Maole_002003.T1, Maole_002136.T1, Maole_002411.T1, Maole_002634.T1, Mao
0115.T1, Maole_018033.T1, Maole_018034.T1, Maole_018035.T1, Maole_019820.T1, Mao
1401.T1, Maole_012022.T1, Maole_012857.T1, Maole_015061.T1, Maole_016444.T1, Mao
9027.T1, Maole_011880.T1, Maole_012004.T1, Maole_020919.T1

3326.T1, Maole_003754.T1, Maole_004874.T1, Maole_006992.T1, Maole_007101.T1, Mao
3602.T1, Maole_003604.T1, Maole_003605.T1, Maole_003607.T1, Maole_005232.T1, Mao

5807.T1, Maole_006444.T1, Maole_006445.T1, Maole_007894.T1, Maole_008023.T1, Mao

1718.T1
1377.T1, Maole_011378.T1, Maole_012617.T1, Maole_016525.T1, Maole_016953.T1, Mao

7089.T1, Maole_021942.T1
5343.T1, Maole_006199.T1, Maole_006799.T1, Maole_009289.T1, Maole_010021.T1, Mao
7427.T1, Maole_010114.T1, Maole_010115.T1, Maole_015932.T1, Maole_017222.T1
5078.T1, Maole_016517.T1, Maole_017104.T1, Maole_017746.T1, Maole_023332.T1, Mao
1130.T1
7272.T1, Maole_009100.T1, Maole_010479.T1, Maole_011181.T1, Maole_012665.T1, Mao

1993.T1, Maole_023850.T1
1727.T1
0919.T1
6521.T1, Maole_006574.T1, Maole_007898.T1, Maole_008242.T1, Maole_010472.T1, Mao

4228.T1, Maole_016473.T1, Maole_017476.T1, Maole_020187.T1, Maole_020738.T1, Mao

7964.T1, Maole_009883.T1, Maole_012158.T1, Maole_015873.T1, Maole_015932.T1, Mao
6885.T1, Maole_010110.T1, Maole_011930.T1, Maole_020499.T1, Maole_023564.T1, Mao
5818.T1, Maole_006269.T1, Maole_006421.T1, Maole_008305.T1, Maole_010389.T1, Mao

6087.T1, Maole_016088.T1, Maole_018928.T1, Maole_018929.T1, Maole_018931.T1, Mao
1416.T1, Maole_023259.T1, Maole_023260.T1, Maole_023776.T1, Maole_024056.T1, Mao

6695.T1, Maole_014397.T1
6581.T1, Maole_006583.T1
6581.T1, Maole_006583.T1
6581.T1, Maole_006583.T1
6581.T1, Maole_006583.T1
3931.T1
1931.T1, Maole_002003.T1, Maole_002070.T1, Maole_002113.T1, Maole_002588.T1, Mao

1512.T1, Maole_012931.T1, Maole_013184.T1, Maole_013596.T1, Maole_015254.T1
1394.T1, Maole_001512.T1, Maole_001514.T1, Maole_001534.T1, Maole_001535.T1, Mao

9580.T1, Maole_021727.T1, Maole_023299.T1
4445.T1, Maole_007455.T1, Maole_007690.T1, Maole_008703.T1, Maole_009121.T1, Mao

0819.T1
3357.T1
5610.T1
6682.T1, Maole_008396.T1
0517.T1
4228.T1, Maole_016473.T1, Maole_017476.T1, Maole_020187.T1, Maole_020738.T1, Mao

4164.T1, Maole_004785.T1, Maole_005101.T1, Maole_006559.T1, Maole_008243.T1, Mao

0226.T1

1813.T1, Maole_013579.T1, Maole_014669.T1, Maole_016155.T1, Maole_017237.T1, Mao
0517.T1, Maole_020738.T1, Maole_023468.T1
9180.T1, Maole_011216.T1, Maole_011626.T1, Maole_012767.T1, Maole_014546.T1, Mao
6692.T1, Maole_006693.T1, Maole_006694.T1, Maole_006695.T1, Maole_007148.T1, Mao
9505.T1, Maole_021063.T1
9505.T1, Maole_021063.T1
5902.T1, Maole_007236.T1, Maole_008053.T1, Maole_008489.T1, Maole_009064.T1, Mao
3449.T1, Maole_017660.T1, Maole_017679.T1, Maole_017900.T1, Maole_018614.T1
0384.T1, Maole_023283.T1
1700.T1, Maole_002146.T1, Maole_002406.T1, Maole_003056.T1, Maole_003100.T1, Mao
3650.T1, Maole_014926.T1, Maole_017432.T1, Maole_018847.T1, Maole_018849.T1, Mao

1202.T1
2202.T1, Maole_003754.T1, Maole_004136.T1, Maole_004673.T1, Maole_004704.T1, Mao
5016.T1, Maole_015679.T1, Maole_015838.T1, Maole_018100.T1, Maole_018307.T1, Mao
9099.T1, Maole_013408.T1, Maole_018391.T1, Maole_018533.T1, Maole_020438.T1
1589.T1, Maole_014603.T1, Maole_015140.T1, Maole_020436.T1
1512.T1, Maole_012931.T1, Maole_013184.T1, Maole_013596.T1, Maole_015254.T1
3564.T1, Maole_023753.T1
5925.T1, Maole_006799.T1, Maole_012022.T1, Maole_012819.T1, Maole_014744.T1, Mao
1827.T1, Maole_012136.T1
9453.T1, Maole_020655.T1, Maole_021573.T1, Maole_022866.T1
7261.T1, Maole_017653.T1, Maole_022283.T1
1240.T1
8396.T1, Maole_009091.T1, Maole_009522.T1, Maole_015202.T1, Maole_016525.T1, Mao

3085.T1, Maole_003221.T1, Maole_004215.T1, Maole_005654.T1, Maole_005846.T1, Mao
1880.T1, Maole_012004.T1, Maole_020919.T1
6576.T1, Maole_018524.T1, Maole_020092.T1
2067.T1, Maole_003496.T1, Maole_004136.T1, Maole_004156.T1, Maole_004334.T1, Mao

1249.T1, Maole_021255.T1
1249.T1, Maole_021255.T1
4942.T1, Maole_005343.T1, Maole_011954.T1, Maole_012360.T1, Maole_013163.T1, Mao

4199.T1, Maole_016541.T1, Maole_016542.T1
4199.T1, Maole_016541.T1, Maole_016542.T1

0934.T1, Maole_012144.T1, Maole_012145.T1, Maole_017220.T1
0540.T1, Maole_013292.T1, Maole_014705.T1, Maole_016016.T1, Maole_016525.T1, Mao

1942.T1

2400.T1, Maole_012797.T1, Maole_014519.T1, Maole_017055.T1, Maole_017814.T1, Mao

2003.T1, Maole_002433.T1, Maole_003326.T1, Maole_003688.T1, Maole_003848.T1, Mao
5531.T1, Maole_005534.T1, Maole_005535.T1, Maole_009025.T1, Maole_014400.T1, Mao

6806.T1
7220.T1
3616.T1, Maole_018460.T1, Maole_018461.T1, Maole_018463.T1, Maole_018465.T1, Mao
5615.T1, Maole_015908.T1, Maole_015909.T1, Maole_020367.T1, Maole_022414.T1
7108.T1, Maole_009067.T1, Maole_009465.T1, Maole_010599.T1, Maole_011873.T1, Mao
5677.T1, Maole_016473.T1
0426.T1, Maole_021181.T1
2375.T1, Maole_013596.T1, Maole_014228.T1, Maole_016473.T1, Maole_017277.T1, Mao

1993.T1

4199.T1, Maole_016541.T1, Maole_016542.T1, Maole_021530.T1, Maole_021763.T1
2144.T1, Maole_012145.T1, Maole_016155.T1, Maole_017313.T1, Maole_022720.T1
3994.T1, Maole_005102.T1, Maole_009505.T1, Maole_011163.T1, Maole_014267.T1, Mao
9473.T1, Maole_009758.T1, Maole_011161.T1, Maole_012820.T1, Maole_014711.T1, Mao

le_000400.T1, Maole_000418.T1, Maole_000432.T1, Maole_000465.T1, Maole_000473.T1,
le_000381.T1, Maole_000390.T1, Maole_000400.T1, Maole_000418.T1, Maole_000473.T1,
le_000418.T1, Maole_000432.T1, Maole_000465.T1, Maole_000473.T1, Maole_000518.T1,
le_000698.T1, Maole_001017.T1, Maole_001097.T1, Maole_001137.T1, Maole_001301.T1,
le_000455.T1, Maole_000473.T1, Maole_000490.T1, Maole_000517.T1, Maole_000518.T1,
le_001301.T1, Maole_001375.T1, Maole_001561.T1, Maole_001631.T1, Maole_001703.T1,
le_001375.T1, Maole_001631.T1, Maole_001757.T1, Maole_001879.T1, Maole_002191.T1,
le_001631.T1, Maole_001757.T1, Maole_001879.T1, Maole_002191.T1, Maole_002288.T1,
le_001375.T1, Maole_001631.T1, Maole_001757.T1, Maole_001879.T1, Maole_002191.T1,
le_001375.T1, Maole_001631.T1, Maole_001757.T1, Maole_001879.T1, Maole_002191.T1,
le_001301.T1, Maole_001375.T1, Maole_001631.T1, Maole_001757.T1, Maole_001879.T1,
le_000698.T1, Maole_000856.T1, Maole_000864.T1, Maole_000870.T1, Maole_000886.T1,
le_000893.T1, Maole_001017.T1, Maole_001097.T1, Maole_001137.T1, Maole_001561.T1,
le_001210.T1, Maole_001703.T1, Maole_002406.T1, Maole_002704.T1, Maole_002715.T1,
le_006992.T1, Maole_007322.T1, Maole_007323.T1, Maole_007381.T1, Maole_007383.T1,
le_004301.T1, Maole_004916.T1, Maole_004918.T1, Maole_004942.T1, Maole_006692.T1,
le_005101.T1, Maole_005388.T1, Maole_005505.T1, Maole_005612.T1, Maole_006437.T1,
le_001037.T1, Maole_001096.T1, Maole_001108.T1, Maole_001237.T1, Maole_001501.T1,
le_001469.T1, Maole_001516.T1, Maole_001616.T1, Maole_001854.T1, Maole_001931.T1,
le_007979.T1, Maole_007982.T1, Maole_008636.T1, Maole_009003.T1, Maole_009440.T1,
le_002136.T1, Maole_002556.T1, Maole_002736.T1, Maole_002854.T1, Maole_002913.T1,
le_001987.T1, Maole_002202.T1, Maole_002556.T1, Maole_002899.T1, Maole_003498.T1,
le_001566.T1, Maole_001578.T1, Maole_001609.T1, Maole_001854.T1, Maole_002136.T1,
le_009364.T1, Maole_009509.T1, Maole_009644.T1, Maole_009992.T1, Maole_011128.T1,
le_003560.T1, Maole_003905.T1, Maole_004246.T1, Maole_004798.T1, Maole_006040.T1,
le_001096.T1, Maole_001108.T1, Maole_001137.T1, Maole_001227.T1, Maole_001356.T1,
le_002401.T1, Maole_002591.T1, Maole_002750.T1, Maole_003203.T1, Maole_003498.T1,
le_005344.T1, Maole_005388.T1, Maole_005418.T1, Maole_005557.T1, Maole_005622.T1,
le_001106.T1, Maole_001123.T1, Maole_001124.T1, Maole_001125.T1, Maole_001209.T1,
le_002693.T1, Maole_002736.T1, Maole_002854.T1, Maole_002913.T1, Maole_003092.T1,
le_002239.T1, Maole_002406.T1, Maole_002556.T1, Maole_002689.T1, Maole_002783.T1,
le_001986.T1, Maole_001987.T1, Maole_002191.T1, Maole_002288.T1, Maole_002634.T1,
le_012595.T1, Maole_012596.T1, Maole_012598.T1, Maole_013561.T1, Maole_014847.T1,
le_010254.T1, Maole_010356.T1, Maole_010359.T1, Maole_010362.T1, Maole_012789.T1,
le_009248.T1, Maole_009294.T1, Maole_009817.T1, Maole_010035.T1, Maole_011380.T1,
le_003953.T1, Maole_004189.T1, Maole_004230.T1, Maole_006799.T1, Maole_006933.T1,
le_011159.T1, Maole_011407.T1, Maole_013561.T1, Maole_014744.T1, Maole_014835.T1,
le_007830.T1, Maole_007939.T1, Maole_008122.T1, Maole_008318.T1, Maole_008618.T1,
le_017086.T1, Maole_017087.T1, Maole_017092.T1, Maole_017128.T1, Maole_021494.T1,
le_001935.T1, Maole_003007.T1, Maole_004003.T1, Maole_004136.T1, Maole_004156.T1,
le_014272.T1, Maole_014274.T1, Maole_014275.T1, Maole_015116.T1, Maole_015873.T1,
le_010254.T1, Maole_010356.T1, Maole_010359.T1, Maole_010362.T1, Maole_012789.T1,
le_004230.T1, Maole_004398.T1, Maole_004564.T1, Maole_004862.T1, Maole_004982.T1,
le_009505.T1, Maole_010110.T1, Maole_010336.T1, Maole_011163.T1, Maole_011519.T1,
le_004939.T1, Maole_005023.T1, Maole_006040.T1, Maole_006624.T1, Maole_006885.T1,
le_001210.T1, Maole_001422.T1, Maole_001481.T1, Maole_002239.T1, Maole_002406.T1,
le_011670.T1, Maole_011691.T1, Maole_011692.T1, Maole_012116.T1, Maole_014466.T1,

le_012158.T1, Maole_012507.T1, Maole_013130.T1, Maole_013299.T1, Maole_014602.T1,
le_011159.T1, Maole_011357.T1, Maole_011358.T1, Maole_011401.T1, Maole_013561.T1,
le_014275.T1, Maole_015116.T1, Maole_021416.T1, Maole_023259.T1, Maole_023260.T1,
le_000839.T1, Maole_000844.T1, Maole_000870.T1, Maole_000886.T1, Maole_000899.T1,
le_003601.T1, Maole_003602.T1, Maole_003604.T1, Maole_003605.T1, Maole_003607.T1,
le_003557.T1, Maole_003560.T1, Maole_004251.T1, Maole_005483.T1, Maole_006036.T1,
le_011519.T1, Maole_015116.T1, Maole_018059.T1, Maole_022187.T1, Maole_022607.T1,
le_004548.T1, Maole_004862.T1, Maole_005101.T1, Maole_005102.T1, Maole_005110.T1,
le_011885.T1, Maole_014516.T1, Maole_014730.T1, Maole_015116.T1, Maole_015735.T1,
le_013561.T1, Maole_013780.T1, Maole_014744.T1, Maole_016253.T1, Maole_020501.T1,
le_016855.T1, Maole_017397.T1, Maole_017398.T1
le_004980.T1, Maole_004982.T1, Maole_005385.T1, Maole_005514.T1, Maole_005555.T1,
le_007703.T1, Maole_008432.T1, Maole_009317.T1, Maole_009320.T1, Maole_010110.T1,
le_012665.T1, Maole_014746.T1, Maole_016665.T1, Maole_019070.T1, Maole_020238.T1
le_004337.T1, Maole_005005.T1, Maole_005372.T1, Maole_005461.T1, Maole_005483.T1,
le_012061.T1, Maole_016177.T1, Maole_016571.T1, Maole_016933.T1, Maole_017223.T1,
le_004862.T1, Maole_004942.T1, Maole_005018.T1, Maole_005555.T1, Maole_006614.T1,
le_018592.T1, Maole_018597.T1, Maole_018598.T1, Maole_018866.T1, Maole_019404.T1,
le_004696.T1, Maole_004714.T1, Maole_004972.T1, Maole_005074.T1, Maole_006040.T1,
le_009102.T1, Maole_009610.T1, Maole_009888.T1, Maole_011345.T1, Maole_011352.T1,
le_001986.T1, Maole_001987.T1, Maole_002003.T1, Maole_002056.T1, Maole_002433.T1,
le_004819.T1, Maole_005152.T1, Maole_006581.T1, Maole_006583.T1, Maole_007425.T1,
le_009817.T1, Maole_015027.T1, Maole_016461.T1, Maole_016463.T1, Maole_016467.T1,
le_008707.T1, Maole_010934.T1, Maole_011523.T1, Maole_012144.T1, Maole_012145.T1,
le_014602.T1, Maole_014641.T1, Maole_016011.T1, Maole_017094.T1, Maole_018026.T1,
le_009883.T1, Maole_009884.T1, Maole_009885.T1, Maole_011321.T1, Maole_011322.T1,
le_015873.T1, Maole_016011.T1, Maole_017094.T1, Maole_018026.T1, Maole_019951.T1,
le_002028.T1, Maole_002241.T1, Maole_002433.T1, Maole_002627.T1, Maole_002680.T1,

le_003283.T1, Maole_004047.T1, Maole_004885.T1, Maole_005119.T1, Maole_005557.T1,
le_006100.T1, Maole_006269.T1, Maole_008292.T1, Maole_008299.T1, Maole_008305.T1,
le_007108.T1, Maole_008706.T1, Maole_008873.T1, Maole_011813.T1, Maole_014744.T1,
le_008505.T1, Maole_008512.T1, Maole_008790.T1, Maole_012531.T1, Maole_012626.T1,
le_002406.T1, Maole_002736.T1, Maole_002782.T1, Maole_003227.T1, Maole_003232.T1,
le_017648.T1, Maole_017866.T1, Maole_017867.T1, Maole_017868.T1, Maole_017870.T1,
le_005622.T1, Maole_005625.T1, Maole_005626.T1, Maole_006651.T1, Maole_006652.T1,
le_011813.T1, Maole_014744.T1, Maole_016169.T1, Maole_016237.T1, Maole_017713.T1,
le_002029.T1, Maole_002146.T1, Maole_002240.T1, Maole_002406.T1, Maole_002634.T1,
le_003310.T1, Maole_005237.T1, Maole_005998.T1, Maole_006467.T1, Maole_007183.T1,
le_004100.T1, Maole_004101.T1, Maole_004104.T1, Maole_004105.T1, Maole_004982.T1,
le_008706.T1, Maole_011813.T1, Maole_012819.T1, Maole_014744.T1, Maole_015343.T1,
le_009432.T1, Maole_013413.T1, Maole_015214.T1, Maole_016032.T1, Maole_016169.T1,
le_007323.T1, Maole_007590.T1, Maole_009505.T1, Maole_011163.T1, Maole_014267.T1,
le_009320.T1, Maole_010110.T1, Maole_010293.T1, Maole_010336.T1, Maole_011519.T1,

le_017397.T1, Maole_017398.T1

le_020028.T1, Maole_020657.T1
le_013450.T1, Maole_013694.T1, Maole_014796.T1, Maole_016271.T1, Maole_017210.T1,
le_002913.T1, Maole_003259.T1, Maole_003261.T1, Maole_003356.T1, Maole_003625.T1,
le_017220.T1, Maole_018579.T1, Maole_019075.T1, Maole_021717.T1, Maole_023840.T1
le_007108.T1, Maole_007303.T1, Maole_007320.T1, Maole_007391.T1, Maole_007427.T1,
le_012682.T1, Maole_014919.T1, Maole_016382.T1, Maole_016806.T1

le_023466.T1
le_022760.T1, Maole_023851.T1
le_006581.T1, Maole_006583.T1, Maole_006896.T1, Maole_007687.T1, Maole_007749.T1,
le_007236.T1, Maole_008020.T1, Maole_009064.T1, Maole_009817.T1, Maole_009921.T1,
le_004334.T1, Maole_004457.T1, Maole_004598.T1, Maole_004656.T1, Maole_004704.T1,

le_009102.T1, Maole_009610.T1, Maole_009888.T1, Maole_011813.T1, Maole_015626.T1,
le_022733.T1, Maole_022737.T1
le_001608.T1, Maole_001616.T1, Maole_002028.T1, Maole_002389.T1, Maole_002406.T1,

le_016169.T1, Maole_017309.T1, Maole_019453.T1, Maole_021185.T1, Maole_021573.T1,
le_020610.T1, Maole_020826.T1, Maole_021993.T1
le_015423.T1, Maole_017086.T1, Maole_017734.T1, Maole_019231.T1, Maole_019968.T1,
le_007749.T1, Maole_008178.T1, Maole_008703.T1, Maole_009091.T1, Maole_010452.T1,
le_005818.T1, Maole_006885.T1, Maole_008387.T1, Maole_009888.T1, Maole_011163.T1,
le_016953.T1, Maole_019070.T1, Maole_019929.T1, Maole_020238.T1
le_005414.T1, Maole_006095.T1, Maole_006885.T1, Maole_007323.T1, Maole_007703.T1,
le_007285.T1, Maole_007898.T1, Maole_014858.T1, Maole_017660.T1, Maole_017900.T1,

le_005236.T1, Maole_005385.T1, Maole_005935.T1, Maole_007843.T1, Maole_008874.T1,
le_009096.T1, Maole_009099.T1, Maole_009289.T1, Maole_010021.T1, Maole_011406.T1,

le_004548.T1, Maole_005414.T1, Maole_005818.T1, Maole_007074.T1, Maole_007143.T1,
le_004880.T1, Maole_005625.T1, Maole_005626.T1, Maole_006068.T1, Maole_006356.T1,
le_001986.T1, Maole_001987.T1, Maole_002288.T1, Maole_002411.T1, Maole_002779.T1,
le_019820.T1, Maole_023245.T1
le_007455.T1, Maole_007749.T1, Maole_008178.T1, Maole_008703.T1, Maole_009029.T1,
le_005626.T1, Maole_005709.T1, Maole_007703.T1, Maole_008182.T1, Maole_008264.T1,
le_011163.T1, Maole_013413.T1, Maole_013870.T1, Maole_017331.T1, Maole_018459.T1,

le_004003.T1, Maole_004942.T1, Maole_007843.T1, Maole_008313.T1, Maole_009968.T1,

le_019512.T1, Maole_021285.T1
le_016890.T1, Maole_018026.T1, Maole_018075.T1, Maole_019951.T1, Maole_020968.T1,

le_010920.T1, Maole_011595.T1, Maole_011678.T1, Maole_012299.T1, Maole_012301.T1,
le_010920.T1, Maole_011595.T1, Maole_011678.T1, Maole_012299.T1, Maole_012301.T1,
le_010920.T1, Maole_011595.T1, Maole_011678.T1, Maole_012299.T1, Maole_012301.T1,

le_003030.T1, Maole_003232.T1, Maole_003245.T1, Maole_004210.T1, Maole_004319.T1,
le_003607.T1, Maole_006469.T1, Maole_007425.T1, Maole_007749.T1, Maole_009248.T1,
le_018864.T1, Maole_019391.T1, Maole_019762.T1, Maole_019764.T1, Maole_019766.T1,
le_003848.T1, Maole_004136.T1, Maole_004251.T1, Maole_004457.T1, Maole_004750.T1,

le_011514.T1, Maole_011533.T1, Maole_011536.T1, Maole_012227.T1, Maole_013045.T1,

le_016473.T1
le_013830.T1, Maole_015154.T1, Maole_020869.T1
le_013359.T1, Maole_014142.T1, Maole_014640.T1, Maole_015471.T1, Maole_018948.T1,
le_014275.T1
le_012448.T1, Maole_014265.T1, Maole_014267.T1, Maole_014269.T1, Maole_014270.T1,
le_005807.T1, Maole_005983.T1, Maole_006211.T1, Maole_006444.T1, Maole_006445.T1,
le_009102.T1, Maole_013780.T1, Maole_014397.T1, Maole_020423.T1, Maole_022732.T1,
le_010554.T1, Maole_011514.T1, Maole_011533.T1, Maole_011536.T1, Maole_012227.T1,
le_011595.T1, Maole_011678.T1, Maole_012299.T1, Maole_012301.T1, Maole_013121.T1,
le_017220.T1, Maole_018579.T1, Maole_023840.T1

le_010383.T1, Maole_013689.T1, Maole_014174.T1, Maole_014466.T1, Maole_014474.T1,
le_023563.T1, Maole_023564.T1, Maole_023753.T1

le_013830.T1, Maole_015154.T1, Maole_020869.T1
le_005908.T1, Maole_006257.T1, Maole_006337.T1, Maole_006933.T1, Maole_007942.T1,
le_020738.T1, Maole_021717.T1
le_008505.T1, Maole_008790.T1, Maole_009025.T1, Maole_011051.T1, Maole_012008.T1,

le_023357.T1

le_019253.T1, Maole_019256.T1, Maole_019257.T1, Maole_019580.T1, Maole_023299.T1

le_010554.T1, Maole_011514.T1, Maole_011533.T1, Maole_011536.T1, Maole_012227.T1,
le_010554.T1, Maole_011514.T1, Maole_011533.T1, Maole_011536.T1, Maole_012227.T1,

le_021185.T1, Maole_021573.T1, Maole_023076.T1

le_004164.T1, Maole_004363.T1, Maole_004409.T1, Maole_005101.T1, Maole_005741.T1,
le_010920.T1, Maole_011595.T1, Maole_011678.T1, Maole_012299.T1, Maole_012301.T1,

le_009289.T1, Maole_010021.T1, Maole_010661.T1, Maole_010662.T1, Maole_012819.T1,

le_011026.T1, Maole_011930.T1, Maole_013856.T1, Maole_016012.T1, Maole_017214.T1,
le_007108.T1, Maole_007285.T1, Maole_007631.T1, Maole_008706.T1, Maole_008707.T1,
le_011670.T1, Maole_011953.T1, Maole_012673.T1, Maole_014430.T1, Maole_014477.T1,

le_002779.T1, Maole_003203.T1, Maole_003560.T1, Maole_004129.T1, Maole_005221.T1,

le_022820.T1
le_003604.T1, Maole_003605.T1, Maole_003607.T1, Maole_004714.T1, Maole_006469.T1,
le_019303.T1, Maole_021844.T1, Maole_022842.T1, Maole_024010.T1

le_006199.T1, Maole_006311.T1, Maole_008382.T1, Maole_009289.T1, Maole_010021.T1,
le_006559.T1, Maole_009043.T1, Maole_009242.T1, Maole_009289.T1, Maole_010021.T1,

le_017331.T1, Maole_018459.T1, Maole_021727.T1, Maole_023357.T1

le_006068.T1, Maole_006095.T1, Maole_006199.T1, Maole_007189.T1, Maole_007391.T1,
le_014640.T1, Maole_015766.T1, Maole_017911.T1, Maole_018391.T1, Maole_019480.T1,
le_006614.T1, Maole_007342.T1, Maole_009968.T1, Maole_010423.T1, Maole_010427.T1,
le_024121.T1

le_000893.T1, Maole_000945.T1, Maole_001017.T1, Maole_001025.T1, Maole_001037.T1,
le_011288.T1, Maole_011813.T1, Maole_012819.T1, Maole_013413.T1, Maole_013561.T1,

le_020499.T1, Maole_023564.T1, Maole_023753.T1
le_017245.T1, Maole_017398.T1, Maole_022347.T1, Maole_022350.T1, Maole_022678.T1

le_005276.T1, Maole_009619.T1, Maole_009962.T1, Maole_012626.T1, Maole_012627.T1,
le_012145.T1, Maole_012448.T1, Maole_013030.T1, Maole_014314.T1, Maole_014685.T1,
le_022471.T1
le_009465.T1, Maole_010599.T1, Maole_011873.T1, Maole_014059.T1, Maole_016473.T1,

le_006670.T1, Maole_006779.T1, Maole_007057.T1, Maole_007108.T1, Maole_007126.T1,

le_006702.T1, Maole_008292.T1, Maole_008734.T1, Maole_011161.T1, Maole_013130.T1,

le_006885.T1, Maole_009038.T1, Maole_010110.T1, Maole_012649.T1, Maole_012673.T1,

le_010762.T1, Maole_010763.T1, Maole_013630.T1, Maole_014142.T1, Maole_017051.T1,

le_002779.T1, Maole_003283.T1, Maole_003994.T1, Maole_005478.T1, Maole_005818.T1,
le_016542.T1, Maole_021285.T1
le_013571.T1, Maole_013689.T1, Maole_014174.T1, Maole_014431.T1, Maole_014883.T1,

le_015343.T1, Maole_016401.T1, Maole_017460.T1, Maole_017992.T1, Maole_020479.T1

le_006126.T1, Maole_006766.T1, Maole_006915.T1, Maole_007590.T1, Maole_007834.T1,
le_018460.T1, Maole_018461.T1, Maole_018463.T1, Maole_018465.T1, Maole_019627.T1,

le_002707.T1, Maole_003085.T1, Maole_004129.T1, Maole_004215.T1, Maole_004304.T1,
le_023245.T1
le_017476.T1, Maole_018075.T1, Maole_019427.T1, Maole_022482.T1, Maole_023468.T1,

le_007703.T1, Maole_007827.T1, Maole_007834.T1, Maole_009303.T1, Maole_009505.T1,
le_006469.T1, Maole_007394.T1, Maole_010554.T1, Maole_010914.T1, Maole_011514.T1,

le_008615.T1, Maole_009890.T1, Maole_009921.T1, Maole_010457.T1, Maole_010943.T1,

le_016978.T1, Maole_018651.T1, Maole_020238.T1, Maole_020605.T1, Maole_020610.T1,

le_010760.T1, Maole_010762.T1, Maole_011813.T1, Maole_012022.T1, Maole_013630.T1,

le_023688.T1

le_014746.T1, Maole_019070.T1, Maole_020238.T1

le_013157.T1, Maole_013335.T1, Maole_014435.T1, Maole_014517.T1, Maole_015032.T1,

le_023468.T1

le_016612.T1, Maole_016977.T1, Maole_016978.T1, Maole_017222.T1, Maole_017644.T1,
le_023753.T1
le_011318.T1, Maole_011589.T1, Maole_012819.T1, Maole_014580.T1, Maole_017163.T1,

le_018932.T1, Maole_023409.T1
le_024121.T1

le_002693.T1, Maole_003084.T1, Maole_003203.T1, Maole_004047.T1, Maole_004862.T1,

le_001551.T1, Maole_001553.T1, Maole_001578.T1, Maole_001610.T1, Maole_001631.T1,

le_009522.T1, Maole_012406.T1, Maole_012931.T1, Maole_013184.T1, Maole_015202.T1,

le_023468.T1

le_009027.T1, Maole_009473.T1, Maole_011020.T1, Maole_012318.T1, Maole_012865.T1,

le_017713.T1, Maole_023761.T1

le_017076.T1, Maole_018279.T1, Maole_018847.T1, Maole_019223.T1, Maole_020122.T1
le_009096.T1, Maole_014397.T1, Maole_014516.T1, Maole_015044.T1, Maole_015729.T1,

le_010337.T1, Maole_012649.T1, Maole_013127.T1, Maole_013357.T1, Maole_016735.T1,

le_003227.T1, Maole_004231.T1, Maole_004819.T1, Maole_004896.T1, Maole_005944.T1,
le_019149.T1, Maole_020066.T1, Maole_020616.T1, Maole_022041.T1

le_004750.T1, Maole_005930.T1, Maole_006521.T1, Maole_006574.T1, Maole_007898.T1,
le_018627.T1

le_016358.T1, Maole_017309.T1, Maole_019075.T1, Maole_019453.T1, Maole_021573.T1,

le_020854.T1, Maole_021425.T1, Maole_023076.T1

le_006203.T1, Maole_006257.T1, Maole_007244.T1, Maole_007633.T1, Maole_008020.T1,

le_004598.T1, Maole_004750.T1, Maole_004936.T1, Maole_005237.T1, Maole_005567.T1,

le_013339.T1, Maole_016890.T1, Maole_019427.T1, Maole_022760.T1

le_016775.T1, Maole_018783.T1, Maole_021847.T1, Maole_023290.T1

le_020993.T1, Maole_022078.T1, Maole_023612.T1

le_004246.T1, Maole_004412.T1, Maole_004881.T1, Maole_004896.T1, Maole_005058.T1,
le_015027.T1, Maole_016398.T1, Maole_018540.T1, Maole_018541.T1, Maole_018563.T1,

le_018928.T1, Maole_018929.T1, Maole_018931.T1, Maole_018932.T1, Maole_019031.T1,

le_014059.T1, Maole_016473.T1, Maole_016970.T1, Maole_017128.T1, Maole_021494.T1,

le_017476.T1, Maole_020187.T1, Maole_020517.T1, Maole_020738.T1, Maole_023468.T1

le_014978.T1, Maole_020819.T1, Maole_022471.T1
le_015214.T1, Maole_017129.T1, Maole_018140.T1

, Maole_000497.T1, Maole_000517.T1, Maole_000518.T1, Maole_000519.T1, Maole_00055
, Maole_000474.T1, Maole_000497.T1, Maole_000498.T1, Maole_000517.T1, Maole_00051
, Maole_000519.T1, Maole_000687.T1, Maole_000690.T1, Maole_000698.T1, Maole_00074
, Maole_001375.T1, Maole_001631.T1, Maole_001703.T1, Maole_001757.T1, Maole_00185
, Maole_000519.T1, Maole_000636.T1, Maole_000643.T1, Maole_000644.T1, Maole_00069
, Maole_001757.T1, Maole_001879.T1, Maole_002191.T1, Maole_002288.T1, Maole_00230
, Maole_002288.T1, Maole_002304.T1, Maole_002326.T1, Maole_002461.T1, Maole_00246
, Maole_002304.T1, Maole_002326.T1, Maole_002461.T1, Maole_002469.T1, Maole_00265
, Maole_002288.T1, Maole_002304.T1, Maole_002326.T1, Maole_002461.T1, Maole_00246
, Maole_002288.T1, Maole_002304.T1, Maole_002326.T1, Maole_002461.T1, Maole_00246
, Maole_002191.T1, Maole_002288.T1, Maole_002304.T1, Maole_002326.T1, Maole_00246
, Maole_000934.T1, Maole_000945.T1, Maole_001019.T1, Maole_001020.T1, Maole_00102
, Maole_001703.T1, Maole_001757.T1, Maole_001859.T1, Maole_001879.T1, Maole_00209
, Maole_003356.T1, Maole_004167.T1, Maole_004238.T1, Maole_004432.T1, Maole_00589
, Maole_007462.T1, Maole_007979.T1, Maole_007982.T1, Maole_008636.T1, Maole_00900
, Maole_006693.T1, Maole_006694.T1, Maole_006695.T1, Maole_006799.T1, Maole_00793
, Maole_006559.T1, Maole_006766.T1, Maole_006885.T1, Maole_007422.T1, Maole_00759
, Maole_001545.T1, Maole_001609.T1, Maole_001854.T1, Maole_001931.T1, Maole_00201
, Maole_002003.T1, Maole_002240.T1, Maole_002510.T1, Maole_002591.T1, Maole_00265
, Maole_009441.T1, Maole_010148.T1, Maole_011670.T1, Maole_012114.T1, Maole_01211
, Maole_003092.T1, Maole_003101.T1, Maole_003148.T1, Maole_003152.T1, Maole_00331
, Maole_004704.T1, Maole_004880.T1, Maole_005633.T1, Maole_006480.T1, Maole_00669
, Maole_002141.T1, Maole_002689.T1, Maole_002736.T1, Maole_002747.T1, Maole_00285
, Maole_011710.T1, Maole_011885.T1, Maole_012158.T1, Maole_012405.T1, Maole_01250
, Maole_006885.T1, Maole_007703.T1, Maole_008182.T1, Maole_009099.T1, Maole_00932
, Maole_001467.T1, Maole_001566.T1, Maole_001609.T1, Maole_001639.T1, Maole_00193
, Maole_003901.T1, Maole_003978.T1, Maole_004188.T1, Maole_004189.T1, Maole_00427
, Maole_006080.T1, Maole_006356.T1, Maole_006706.T1, Maole_007322.T1, Maole_00732
, Maole_001210.T1, Maole_001404.T1, Maole_001481.T1, Maole_001957.T1, Maole_00222
, Maole_003101.T1, Maole_003148.T1, Maole_003152.T1, Maole_003315.T1, Maole_00377
, Maole_003100.T1, Maole_003148.T1, Maole_003232.T1, Maole_003315.T1, Maole_00359
, Maole_002707.T1, Maole_002761.T1, Maole_002779.T1, Maole_002783.T1, Maole_00332
, Maole_015061.T1, Maole_016253.T1, Maole_016455.T1, Maole_016662.T1, Maole_01767
, Maole_012830.T1, Maole_017647.T1, Maole_019130.T1
, Maole_013961.T1, Maole_014142.T1, Maole_014250.T1, Maole_015819.T1, Maole_01582
, Maole_008706.T1, Maole_011813.T1, Maole_012627.T1, Maole_013216.T1, Maole_01361
, Maole_015354.T1, Maole_017309.T1, Maole_017395.T1, Maole_018862.T1, Maole_01907
, Maole_008831.T1, Maole_009610.T1, Maole_009888.T1, Maole_011813.T1, Maole_01284
, Maole_022202.T1
, Maole_004334.T1, Maole_004598.T1, Maole_004750.T1, Maole_004881.T1, Maole_00493
, Maole_019951.T1, Maole_020968.T1, Maole_021416.T1, Maole_023259.T1, Maole_02326
, Maole_012830.T1, Maole_017647.T1, Maole_019130.T1
, Maole_005232.T1, Maole_006085.T1, Maole_006799.T1, Maole_006933.T1, Maole_00870
, Maole_015116.T1, Maole_020819.T1, Maole_022090.T1, Maole_022471.T1, Maole_02303
, Maole_009303.T1, Maole_009320.T1, Maole_009466.T1, Maole_009598.T1, Maole_01011
, Maole_002588.T1, Maole_002634.T1, Maole_002636.T1, Maole_002645.T1, Maole_00264
, Maole_014475.T1, Maole_014477.T1, Maole_014487.T1, Maole_014488.T1, Maole_01680

, Maole_014641.T1, Maole_016011.T1, Maole_017094.T1, Maole_018026.T1, Maole_0180
, Maole_013780.T1, Maole_014744.T1, Maole_015802.T1, Maole_016253.T1, Maole_0167
, Maole_023776.T1, Maole_024056.T1, Maole_024121.T1
, Maole_000905.T1, Maole_001017.T1, Maole_001023.T1, Maole_001025.T1, Maole_0010
, Maole_004129.T1, Maole_004301.T1, Maole_004500.T1, Maole_005846.T1, Maole_0068
, Maole_006351.T1, Maole_006581.T1, Maole_006583.T1, Maole_006830.T1, Maole_0079
, Maole_023032.T1, Maole_023259.T1, Maole_023260.T1, Maole_023776.T1, Maole_0241
, Maole_005195.T1, Maole_005823.T1, Maole_006040.T1, Maole_006559.T1, Maole_0068
, Maole_015737.T1, Maole_015738.T1, Maole_020436.T1, Maole_021416.T1, Maole_0232
, Maole_020502.T1, Maole_022100.T1, Maole_023076.T1

, Maole_005595.T1, Maole_006386.T1, Maole_006896.T1, Maole_007342.T1, Maole_0074
, Maole_010336.T1, Maole_011519.T1, Maole_012448.T1, Maole_013030.T1, Maole_0145

, Maole_005654.T1, Maole_005814.T1, Maole_005846.T1, Maole_005902.T1, Maole_0067
, Maole_019391.T1, Maole_019762.T1, Maole_021085.T1
, Maole_006692.T1, Maole_006693.T1, Maole_006694.T1, Maole_006695.T1, Maole_0070
, Maole_019405.T1, Maole_019406.T1, Maole_019407.T1, Maole_019416.T1, Maole_0200
, Maole_006885.T1, Maole_007690.T1, Maole_008292.T1, Maole_008299.T1, Maole_0093
, Maole_011813.T1, Maole_011975.T1, Maole_012082.T1, Maole_015626.T1, Maole_0177
, Maole_002652.T1, Maole_003007.T1, Maole_003084.T1, Maole_004129.T1, Maole_0041
, Maole_008596.T1, Maole_009043.T1, Maole_010308.T1, Maole_010601.T1, Maole_0111
, Maole_016855.T1, Maole_017397.T1, Maole_017398.T1, Maole_017460.T1, Maole_0193
, Maole_013763.T1, Maole_016155.T1, Maole_020212.T1, Maole_020226.T1, Maole_0206
, Maole_019951.T1, Maole_020000.T1, Maole_020968.T1, Maole_021679.T1, Maole_0226
, Maole_011614.T1, Maole_011616.T1, Maole_011827.T1, Maole_011873.T1, Maole_0154
, Maole_020000.T1, Maole_020968.T1, Maole_021679.T1, Maole_022600.T1, Maole_0231
, Maole_002689.T1, Maole_003007.T1, Maole_003065.T1, Maole_003084.T1, Maole_0033

, Maole_006561.T1, Maole_007046.T1, Maole_007074.T1, Maole_007504.T1, Maole_0079
, Maole_009025.T1, Maole_010110.T1, Maole_011161.T1, Maole_011318.T1, Maole_0127
, Maole_016169.T1, Maole_017713.T1, Maole_017922.T1, Maole_018075.T1, Maole_0199
, Maole_012627.T1, Maole_012678.T1, Maole_013067.T1, Maole_013216.T1, Maole_0132
, Maole_004072.T1, Maole_004164.T1, Maole_004167.T1, Maole_004251.T1, Maole_0048
, Maole_017871.T1, Maole_017874.T1, Maole_019950.T1
, Maole_006992.T1, Maole_007170.T1, Maole_007386.T1, Maole_007927.T1, Maole_0080
, Maole_018075.T1, Maole_020238.T1, Maole_020517.T1, Maole_023564.T1, Maole_0237
, Maole_002826.T1, Maole_003056.T1, Maole_003100.T1, Maole_003227.T1, Maole_0035
, Maole_007391.T1, Maole_007855.T1, Maole_008616.T1, Maole_008670.T1, Maole_0104
, Maole_005664.T1, Maole_006199.T1, Maole_006311.T1, Maole_006437.T1, Maole_0067
, Maole_016169.T1, Maole_017309.T1, Maole_017713.T1, Maole_017992.T1, Maole_0180
, Maole_019947.T1, Maole_021441.T1, Maole_021727.T1, Maole_023802.T1
, Maole_019167.T1, Maole_020819.T1, Maole_021008.T1, Maole_021164.T1, Maole_0215
, Maole_011589.T1, Maole_013413.T1, Maole_018021.T1, Maole_020296.T1, Maole_0206

, Maole_017313.T1, Maole_018443.T1, Maole_019171.T1, Maole_022716.T1, Maole_02271
, Maole_004331.T1, Maole_004714.T1, Maole_005237.T1, Maole_005388.T1, Maole_00551

, Maole_007964.T1, Maole_009038.T1, Maole_009289.T1, Maole_010021.T1, Maole_01003

, Maole_007966.T1, Maole_009067.T1, Maole_009092.T1, Maole_009248.T1, Maole_00988
, Maole_010337.T1, Maole_014142.T1, Maole_014400.T1, Maole_016011.T1, Maole_01601
, Maole_004750.T1, Maole_004936.T1, Maole_006521.T1, Maole_006574.T1, Maole_00693

, Maole_017713.T1, Maole_020423.T1, Maole_022732.T1, Maole_022733.T1, Maole_02273

, Maole_002680.T1, Maole_002761.T1, Maole_002826.T1, Maole_003030.T1, Maole_00306

, Maole_023076.T1, Maole_023466.T1

, Maole_021494.T1, Maole_022202.T1
, Maole_018033.T1, Maole_018034.T1, Maole_018035.T1, Maole_019820.T1, Maole_02226
, Maole_011813.T1, Maole_012336.T1, Maole_013515.T1, Maole_013785.T1, Maole_01458

, Maole_007834.T1, Maole_008292.T1, Maole_008523.T1, Maole_009272.T1, Maole_00935
, Maole_019947.T1, Maole_021441.T1, Maole_023802.T1

, Maole_008875.T1, Maole_008946.T1, Maole_009725.T1, Maole_010549.T1, Maole_01060
, Maole_015735.T1, Maole_015737.T1, Maole_015738.T1, Maole_021900.T1, Maole_02218

, Maole_007145.T1, Maole_007360.T1, Maole_007703.T1, Maole_007707.T1, Maole_00794
, Maole_006651.T1, Maole_006652.T1, Maole_007143.T1, Maole_007145.T1, Maole_00738
, Maole_003007.T1, Maole_003203.T1, Maole_003560.T1, Maole_003813.T1, Maole_00412

, Maole_009091.T1, Maole_010452.T1, Maole_010780.T1, Maole_011345.T1, Maole_01135
, Maole_008713.T1, Maole_008961.T1, Maole_009099.T1, Maole_011589.T1, Maole_01200
, Maole_019167.T1, Maole_020819.T1, Maole_021008.T1, Maole_021164.T1, Maole_02152

, Maole_010427.T1, Maole_010920.T1, Maole_011444.T1, Maole_011595.T1, Maole_01167

, Maole_022845.T1

, Maole_013121.T1, Maole_013153.T1, Maole_016696.T1, Maole_021905.T1, Maole_02276
, Maole_013121.T1, Maole_013153.T1, Maole_016696.T1, Maole_021905.T1, Maole_02276
, Maole_013121.T1, Maole_013153.T1, Maole_016696.T1, Maole_021905.T1, Maole_02276

, Maole_004626.T1, Maole_005670.T1, Maole_006703.T1, Maole_007427.T1, Maole_00742
, Maole_009367.T1, Maole_010554.T1, Maole_011514.T1, Maole_011533.T1, Maole_0115
, Maole_019767.T1
, Maole_004787.T1, Maole_005046.T1, Maole_005221.T1, Maole_005935.T1, Maole_0063

, Maole_016671.T1, Maole_016806.T1, Maole_018253.T1, Maole_018810.T1, Maole_0193

, Maole_019480.T1, Maole_020438.T1, Maole_020690.T1, Maole_021075.T1, Maole_0210

, Maole_014272.T1, Maole_014274.T1, Maole_014275.T1, Maole_016806.T1
, Maole_006624.T1, Maole_007894.T1, Maole_008018.T1, Maole_008023.T1, Maole_00861
, Maole_022733.T1, Maole_022737.T1, Maole_023851.T1
, Maole_012853.T1, Maole_013045.T1, Maole_015908.T1, Maole_015909.T1, Maole_0166
, Maole_013153.T1, Maole_016696.T1, Maole_021905.T1, Maole_022760.T1, Maole_0238

, Maole_014475.T1, Maole_019406.T1, Maole_019580.T1, Maole_021727.T1, Maole_0232

, Maole_008143.T1, Maole_008181.T1, Maole_008625.T1, Maole_008706.T1, Maole_0111

, Maole_012531.T1, Maole_012626.T1, Maole_012627.T1, Maole_012678.T1, Maole_01321

, Maole_012853.T1, Maole_013045.T1, Maole_015908.T1, Maole_015909.T1, Maole_0166
, Maole_012853.T1, Maole_013045.T1, Maole_015908.T1, Maole_015909.T1, Maole_0166

, Maole_006417.T1, Maole_006559.T1, Maole_009138.T1, Maole_009142.T1, Maole_0094
, Maole_013121.T1, Maole_013153.T1, Maole_016696.T1, Maole_021905.T1, Maole_0227

, Maole_014744.T1, Maole_017135.T1, Maole_017309.T1, Maole_021900.T1, Maole_0221

, Maole_019075.T1, Maole_019175.T1, Maole_020499.T1, Maole_020616.T1, Maole_02171
, Maole_010934.T1, Maole_011523.T1, Maole_012144.T1, Maole_012145.T1, Maole_01368
, Maole_014484.T1, Maole_014487.T1, Maole_014488.T1, Maole_014490.T1, Maole_01565

, Maole_005607.T1, Maole_005983.T1, Maole_007189.T1, Maole_007236.T1, Maole_00738

, Maole_009150.T1, Maole_009337.T1, Maole_010554.T1, Maole_011345.T1, Maole_01135

, Maole_010293.T1, Maole_010389.T1, Maole_010505.T1, Maole_011813.T1, Maole_01209
, Maole_010540.T1, Maole_011406.T1, Maole_012283.T1, Maole_013292.T1, Maole_01575

, Maole_007427.T1, Maole_007964.T1, Maole_009038.T1, Maole_009719.T1, Maole_01209
, Maole_020436.T1, Maole_023406.T1, Maole_023943.T1
, Maole_010920.T1, Maole_011595.T1, Maole_011678.T1, Maole_012299.T1, Maole_01230

, Maole_001091.T1, Maole_001126.T1, Maole_001137.T1, Maole_001192.T1, Maole_00121
, Maole_014744.T1, Maole_016169.T1, Maole_016266.T1, Maole_017309.T1, Maole_01767

, Maole_014519.T1, Maole_016167.T1, Maole_016171.T1, Maole_016175.T1, Maole_01618
, Maole_015679.T1, Maole_016155.T1, Maole_016890.T1, Maole_019427.T1, Maole_02163

, Maole_016686.T1, Maole_017086.T1, Maole_017128.T1, Maole_021494.T1, Maole_02220

, Maole_009064.T1, Maole_012375.T1, Maole_013413.T1, Maole_013571.T1, Maole_01368

, Maole_013942.T1, Maole_014711.T1, Maole_016953.T1, Maole_017122.T1, Maole_01751

, Maole_012805.T1, Maole_013299.T1, Maole_015932.T1, Maole_017889.T1, Maole_02009

, Maole_017544.T1, Maole_018651.T1, Maole_022121.T1, Maole_022398.T1, Maole_02312

, Maole_006040.T1, Maole_007074.T1, Maole_007101.T1, Maole_007360.T1, Maole_00750

, Maole_016337.T1, Maole_017166.T1, Maole_017261.T1, Maole_017547.T1

, Maole_008625.T1, Maole_010832.T1, Maole_011950.T1, Maole_012276.T1, Maole_01253
, Maole_021614.T1, Maole_021727.T1, Maole_023406.T1, Maole_023943.T1

, Maole_004457.T1, Maole_004564.T1, Maole_004589.T1, Maole_005005.T1, Maole_00510

, Maole_023479.T1

, Maole_011885.T1, Maole_011953.T1, Maole_012114.T1, Maole_012162.T1, Maole_01419
, Maole_011533.T1, Maole_011536.T1, Maole_012227.T1, Maole_012853.T1, Maole_01304

, Maole_010944.T1, Maole_010945.T1, Maole_010946.T1, Maole_012497.T1, Maole_01249

, Maole_020826.T1

, Maole_016665.T1, Maole_017660.T1, Maole_017713.T1, Maole_022121.T1, Maole_02218

, Maole_015653.T1, Maole_015838.T1, Maole_018627.T1, Maole_019439.T1, Maole_02144

, Maole_019939.T1, Maole_019951.T1, Maole_019968.T1, Maole_020517.T1, Maole_02056

, Maole_020436.T1, Maole_020438.T1, Maole_021763.T1, Maole_022716.T1, Maole_02271

, Maole_004880.T1, Maole_005023.T1, Maole_005101.T1, Maole_005102.T1, Maole_00598

, Maole_001730.T1, Maole_001853.T1, Maole_001854.T1, Maole_002141.T1, Maole_00239

, Maole_015254.T1, Maole_016686.T1, Maole_016925.T1, Maole_020854.T1, Maole_02142

, Maole_013884.T1, Maole_014517.T1, Maole_015838.T1, Maole_016007.T1, Maole_01617

, Maole_017644.T1, Maole_019939.T1, Maole_021877.T1, Maole_022970.T1

, Maole_017889.T1, Maole_020092.T1

, Maole_006066.T1, Maole_006083.T1, Maole_006485.T1, Maole_007473.T1, Maole_0085[illegible]

, Maole_009432.T1, Maole_009928.T1, Maole_010277.T1, Maole_010601.T1, Maole_0115[illegible]

, Maole_021717.T1

, Maole_008873.T1, Maole_009360.T1, Maole_009817.T1, Maole_012405.T1, Maole_0124[illegible]

, Maole_005823.T1, Maole_006068.T1, Maole_006315.T1, Maole_007855.T1, Maole_0082[illegible]

, Maole_005059.T1, Maole_005221.T1, Maole_005534.T1, Maole_005535.T1, Maole_0058[illegible]
, Maole_019303.T1, Maole_022244.T1, Maole_022842.T1, Maole_022844.T1

, Maole_019219.T1, Maole_019627.T1, Maole_019629.T1, Maole_020860.T1, Maole_0214[illegible]

, Maole_022202.T1

8.T1, Maole_000672.T1, Maole_000690.T1, Maole_000698.T1, Maole_000737.T1, Maole_
8.T1, Maole_000519.T1, Maole_000672.T1, Maole_000690.T1, Maole_000698.T1, Maole_
7.T1, Maole_000886.T1, Maole_000893.T1, Maole_000934.T1, Maole_000995.T1, Maole_
9.T1, Maole_001879.T1, Maole_002093.T1, Maole_002102.T1, Maole_002191.T1, Maole_
0.T1, Maole_000698.T1, Maole_000738.T1, Maole_000806.T1, Maole_000870.T1, Maole_
4.T1, Maole_002326.T1, Maole_002461.T1, Maole_002469.T1, Maole_002652.T1, Maole_
9.T1, Maole_002652.T1, Maole_002704.T1, Maole_002715.T1, Maole_002721.T1, Maole_
2.T1, Maole_002704.T1, Maole_002715.T1, Maole_002721.T1, Maole_002724.T1, Maole_
9.T1, Maole_002652.T1, Maole_002704.T1, Maole_002715.T1, Maole_002721.T1, Maole_
9.T1, Maole_002652.T1, Maole_002704.T1, Maole_002715.T1, Maole_002721.T1, Maole_
1.T1, Maole_002469.T1, Maole_002652.T1, Maole_002704.T1, Maole_002715.T1, Maole_
1.T1, Maole_001022.T1, Maole_001037.T1, Maole_001100.T1, Maole_001277.T1, Maole_
3.T1, Maole_002102.T1, Maole_002288.T1, Maole_002304.T1, Maole_002326.T1, Maole_
6.T1, Maole_005897.T1, Maole_005898.T1, Maole_006194.T1, Maole_006295.T1, Maole_
3.T1, Maole_009364.T1, Maole_009440.T1, Maole_009441.T1, Maole_009509.T1, Maole_
9.T1, Maole_008831.T1, Maole_008874.T1, Maole_008875.T1, Maole_009029.T1, Maole_
0.T1, Maole_007602.T1, Maole_007834.T1, Maole_008596.T1, Maole_009067.T1, Maole_
2.T1, Maole_002239.T1, Maole_002264.T1, Maole_002390.T1, Maole_002591.T1, Maole_
2.T1, Maole_002855.T1, Maole_003084.T1, Maole_003218.T1, Maole_003227.T1, Maole_
6.T1, Maole_014466.T1, Maole_014469.T1, Maole_014470.T1, Maole_014471.T1, Maole_
5.T1, Maole_003778.T1, Maole_003953.T1, Maole_003987.T1, Maole_004072.T1, Maole_
3.T1, Maole_006799.T1, Maole_007024.T1, Maole_007898.T1, Maole_008382.T1, Maole_
4.T1, Maole_002913.T1, Maole_003092.T1, Maole_003148.T1, Maole_003778.T1, Maole_
7.T1, Maole_013299.T1, Maole_013579.T1, Maole_014602.T1, Maole_014641.T1, Maole_
0.T1, Maole_010110.T1, Maole_010336.T1, Maole_011519.T1, Maole_011589.T1, Maole_
1.T1, Maole_001971.T1, Maole_002136.T1, Maole_002556.T1, Maole_002591.T1, Maole_
5.T1, Maole_005018.T1, Maole_005555.T1, Maole_006386.T1, Maole_006581.T1, Maole_
3.T1, Maole_007366.T1, Maole_007462.T1, Maole_009038.T1, Maole_009068.T1, Maole_
1.T1, Maole_002236.T1, Maole_002239.T1, Maole_002389.T1, Maole_002406.T1, Maole_
8.T1, Maole_003953.T1, Maole_003987.T1, Maole_004072.T1, Maole_005074.T1, Maole_
4.T1, Maole_003848.T1, Maole_004142.T1, Maole_004230.T1, Maole_004231.T1, Maole_
6.T1, Maole_003356.T1, Maole_003498.T1, Maole_003501.T1, Maole_003560.T1, Maole_
3.T1, Maole_022100.T1

0.T1, Maole_016461.T1, Maole_016463.T1, Maole_016467.T1, Maole_016855.T1, Maole_
0.T1, Maole_013625.T1, Maole_014744.T1, Maole_015471.T1, Maole_016169.T1, Maole_
5.T1, Maole_020351.T1, Maole_020655.T1, Maole_021185.T1, Maole_021717.T1, Maole_
5.T1, Maole_015626.T1, Maole_017713.T1, Maole_020423.T1, Maole_022722.T1, Maole_

6.T1, Maole_005102.T1, Maole_005385.T1, Maole_006369.T1, Maole_007723.T1, Maole_
0.T1, Maole_023776.T1, Maole_024056.T1, Maole_024121.T1

6.T1, Maole_010934.T1, Maole_011813.T1, Maole_012022.T1, Maole_012144.T1, Maole_
2.T1, Maole_023259.T1, Maole_023260.T1, Maole_023776.T1, Maole_024056.T1, Maole_
0.T1, Maole_010336.T1, Maole_010480.T1, Maole_011020.T1, Maole_011519.T1, Maole_
8.T1, Maole_002680.T1, Maole_003232.T1, Maole_003245.T1, Maole_003356.T1, Maole_
6.T1, Maole_018590.T1, Maole_018592.T1, Maole_018597.T1, Maole_018598.T1, Maole_

36.T1, Maole_019580.T1, Maole_019951.T1, Maole_020000.T1, Maole_020968.T1, Maole_
58.T1, Maole_017309.T1, Maole_018563.T1, Maole_020501.T1, Maole_020502.T1, Maole_

38.T1, Maole_001060.T1, Maole_001100.T1, Maole_001108.T1, Maole_001126.T1, Maole_
06.T1, Maole_007145.T1, Maole_007939.T1, Maole_009063.T1, Maole_009152.T1, Maole_
44.T1, Maole_008313.T1, Maole_009505.T1, Maole_010016.T1, Maole_011163.T1, Maole_
21.T1
35.T1, Maole_007602.T1, Maole_007923.T1, Maole_007964.T1, Maole_008122.T1, Maole_
59.T1, Maole_023260.T1, Maole_023776.T1, Maole_024056.T1, Maole_024121.T1

29.T1, Maole_007599.T1, Maole_007843.T1, Maole_009029.T1, Maole_009063.T1, Maole_
16.T1, Maole_014517.T1, Maole_015116.T1, Maole_015735.T1, Maole_015737.T1, Maole_

13.T1, Maole_006972.T1, Maole_007143.T1, Maole_007145.T1, Maole_008020.T1, Maole_

24.T1, Maole_007973.T1, Maole_008775.T1, Maole_008874.T1, Maole_008875.T1, Maole_
51.T1
17.T1, Maole_009320.T1, Maole_009720.T1, Maole_010110.T1, Maole_010401.T1, Maole_
13.T1, Maole_019159.T1, Maole_020423.T1, Maole_022732.T1, Maole_022733.T1, Maole_
39.T1, Maole_004304.T1, Maole_004457.T1, Maole_004529.T1, Maole_005023.T1, Maole_
28.T1, Maole_011836.T1, Maole_011954.T1, Maole_012360.T1, Maole_012448.T1, Maole_
03.T1, Maole_022244.T1, Maole_022347.T1, Maole_022678.T1, Maole_022842.T1, Maole_
51.T1, Maole_020652.T1
00.T1, Maole_023194.T1
23.T1, Maole_016343.T1, Maole_016345.T1, Maole_020028.T1, Maole_020657.T1, Maole_
04.T1
56.T1, Maole_003593.T1, Maole_003813.T1, Maole_003886.T1, Maole_004047.T1, Maole_

56.T1, Maole_008546.T1, Maole_009067.T1, Maole_009096.T1, Maole_009099.T1, Maole_
35.T1, Maole_014400.T1, Maole_014711.T1, Maole_015116.T1, Maole_015137.T1, Maole_
47.T1, Maole_020238.T1, Maole_021075.T1, Maole_021076.T1, Maole_021441.T1, Maole_
22.T1, Maole_013359.T1, Maole_013625.T1, Maole_015083.T1, Maole_015471.T1, Maole_
19.T1, Maole_004896.T1, Maole_005074.T1, Maole_005293.T1, Maole_005432.T1, Maole_

53.T1, Maole_008077.T1, Maole_008338.T1, Maole_009038.T1, Maole_009367.T1, Maole_
53.T1
04.T1, Maole_003688.T1, Maole_003773.T1, Maole_004117.T1, Maole_004167.T1, Maole_
34.T1, Maole_011601.T1, Maole_011709.T1, Maole_011761.T1, Maole_011764.T1, Maole_
09.T1, Maole_008596.T1, Maole_008868.T1, Maole_009068.T1, Maole_009289.T1, Maole_
75.T1, Maole_020238.T1, Maole_023564.T1, Maole_023753.T1

20.T1, Maole_022471.T1
16.T1, Maole_021763.T1, Maole_023032.T1, Maole_023259.T1, Maole_023260.T1, Maole_

8.T1, Maole_022720.T1, Maole_022911.T1, Maole_023885.T1
8.T1, Maole_006214.T1, Maole_006647.T1, Maole_006651.T1, Maole_006652.T1, Maole_

5.T1, Maole_011406.T1, Maole_012467.T1, Maole_012554.T1, Maole_012666.T1, Maole_

3.T1, Maole_009968.T1, Maole_010920.T1, Maole_012299.T1, Maole_012301.T1, Maole_
2.T1, Maole_016230.T1, Maole_016461.T1, Maole_016463.T1, Maole_016467.T1, Maole_
3.T1, Maole_007270.T1, Maole_007272.T1, Maole_007898.T1, Maole_008242.T1, Maole_

7.T1

7.T1, Maole_003596.T1, Maole_003754.T1, Maole_004070.T1, Maole_004073.T1, Maole_

5.T1, Maole_022266.T1, Maole_023245.T1, Maole_023837.T1
0.T1, Maole_016012.T1, Maole_016169.T1, Maole_016266.T1, Maole_016278.T1, Maole_

4.T1, Maole_009506.T1, Maole_009552.T1, Maole_010035.T1, Maole_011161.T1, Maole_

6.T1, Maole_010780.T1, Maole_011288.T1, Maole_012520.T1, Maole_012595.T1, Maole_
7.T1, Maole_022607.T1, Maole_023564.T1, Maole_023753.T1

4.T1, Maole_009945.T1, Maole_010427.T1, Maole_010540.T1, Maole_011589.T1, Maole_
6.T1, Maole_007927.T1, Maole_008382.T1, Maole_008616.T1, Maole_008734.T1, Maole_
9.T1, Maole_004880.T1, Maole_005250.T1, Maole_005276.T1, Maole_005622.T1, Maole_

2.T1, Maole_011975.T1, Maole_012082.T1, Maole_013596.T1, Maole_017623.T1, Maole_
8.T1, Maole_012531.T1, Maole_014209.T1, Maole_014584.T1, Maole_018021.T1, Maole_
0.T1, Maole_022471.T1, Maole_023357.T1

8.T1, Maole_012299.T1, Maole_012301.T1, Maole_013121.T1, Maole_013153.T1, Maole_

0.T1, Maole_023851.T1
0.T1, Maole_023851.T1
0.T1, Maole_023851.T1

29.T1, Maole_008800.T1, Maole_009619.T1, Maole_010098.T1, Maole_010114.T1, Maole_
36.T1, Maole_012227.T1, Maole_012328.T1, Maole_012853.T1, Maole_013045.T1, Maole_

56.T1, Maole_006561.T1, Maole_006722.T1, Maole_006960.T1, Maole_007037.T1, Maole_

08.T1, Maole_019310.T1, Maole_020367.T1, Maole_020368.T1, Maole_020370.T1, Maole_

76.T1, Maole_021503.T1, Maole_023053.T1, Maole_023449.T1

15.T1, Maole_009890.T1, Maole_009921.T1, Maole_010457.T1, Maole_010943.T1, Maole_

71.T1, Maole_016806.T1, Maole_018253.T1, Maole_018810.T1, Maole_019308.T1, Maole_
51.T1

09.T1, Maole_023388.T1

08.T1, Maole_011946.T1, Maole_012640.T1, Maole_014904.T1, Maole_016349.T1, Maole_

16.T1, Maole_013222.T1, Maole_013359.T1, Maole_013625.T1, Maole_014209.T1, Maole_

71.T1, Maole_016806.T1, Maole_018253.T1, Maole_018810.T1, Maole_019308.T1, Maole_
71.T1, Maole_016806.T1, Maole_018253.T1, Maole_018810.T1, Maole_019308.T1, Maole_

33.T1, Maole_009473.T1, Maole_009740.T1, Maole_014893.T1, Maole_016171.T1, Maole_
50.T1, Maole_023851.T1

37.T1, Maole_022607.T1, Maole_023753.T1

17.T1, Maole_022051.T1, Maole_023054.T1, Maole_023418.T1, Maole_023564.T1, Maole_
31.T1, Maole_013763.T1, Maole_016155.T1, Maole_018140.T1, Maole_020212.T1, Maole_
59.T1, Maole_017166.T1, Maole_017261.T1, Maole_017653.T1, Maole_019412.T1, Maole_

36.T1, Maole_007927.T1, Maole_009505.T1, Maole_009506.T1, Maole_009921.T1, Maole_

52.T1, Maole_011514.T1, Maole_011533.T1, Maole_011536.T1, Maole_011975.T1, Maole_

93.T1, Maole_012819.T1, Maole_013422.T1, Maole_013561.T1, Maole_013616.T1, Maole_
59.T1, Maole_015762.T1, Maole_016011.T1, Maole_016171.T1, Maole_016666.T1, Maole_

93.T1, Maole_012520.T1, Maole_012554.T1, Maole_012595.T1, Maole_012596.T1, Maole_

01.T1, Maole_013121.T1, Maole_013153.T1, Maole_016696.T1, Maole_016944.T1, Maole_

16.T1, Maole_001240.T1, Maole_001356.T1, Maole_001516.T1, Maole_001523.T1, Maole_
73.T1, Maole_017713.T1, Maole_018075.T1, Maole_019453.T1, Maole_019799.T1, Maole_

31.T1, Maole_016393.T1, Maole_016505.T1, Maole_016506.T1, Maole_016914.T1, Maole_
32.T1

02.T1, Maole_023761.T1

39.T1, Maole_015116.T1, Maole_015577.T1, Maole_018886.T1, Maole_019703.T1, Maole_

15.T1, Maole_018625.T1, Maole_018627.T1, Maole_018783.T1, Maole_019427.T1, Maole_

02.T1, Maole_021727.T1, Maole_023357.T1

29.T1, Maole_023130.T1, Maole_023131.T1

04.T1, Maole_008182.T1, Maole_009096.T1, Maole_009099.T1, Maole_011589.T1, Maole_

37.T1, Maole_012640.T1, Maole_013319.T1, Maole_013321.T1, Maole_013322.T1, Maole_

01.T1, Maole_005102.T1, Maole_005344.T1, Maole_005483.T1, Maole_005505.T1, Maole_

09.T1, Maole_014480.T1, Maole_016382.T1, Maole_016410.T1, Maole_016541.T1, Maole_
45.T1, Maole_015615.T1, Maole_015908.T1, Maole_015909.T1, Maole_016237.T1, Maole_

09.T1, Maole_012501.T1, Maole_012757.T1, Maole_013082.T1, Maole_013616.T1, Maole_

37.T1, Maole_022760.T1, Maole_023130.T1, Maole_023131.T1

41.T1, Maole_023802.T1

65.T1, Maole_020968.T1, Maole_022452.T1

18.T1, Maole_023419.T1

33.T1, Maole_006095.T1, Maole_006315.T1, Maole_006391.T1, Maole_006559.T1, Maole_0

00.T1, Maole_002465.T1, Maole_002512.T1, Maole_002693.T1, Maole_003028.T1, Maole_0

25.T1, Maole_021718.T1, Maole_022460.T1, Maole_023761.T1

71.T1, Maole_017131.T1, Maole_019936.T1, Maole_020051.T1, Maole_020851.T1, Maole_0

)5.T1, Maole_009114.T1, Maole_009181.T1, Maole_009724.T1, Maole_011051.T1, Maole_(

.5.T1, Maole_011642.T1, Maole_011836.T1, Maole_013335.T1, Maole_014314.T1, Maole_(

)6.T1, Maole_013054.T1, Maole_014142.T1, Maole_014250.T1, Maole_016461.T1, Maole_(

!2.T1, Maole_008800.T1, Maole_009213.T1, Maole_010277.T1, Maole_010472.T1, Maole_(

38.T1, Maole_005996.T1, Maole_006315.T1, Maole_006356.T1, Maole_006382.T1, Maole_(

)7.T1, Maole_021422.T1, Maole_022820.T1, Maole_023409.T1

000738.T1, Maole_000747.T1, Maole_000806.T1, Maole_000886.T1, Maole_000893.T1, Ma
000747.T1, Maole_000749.T1, Maole_000806.T1, Maole_000856.T1, Maole_000893.T1, Ma
001097.T1, Maole_001106.T1, Maole_001137.T1, Maole_001301.T1, Maole_001375.T1, Ma
002288.T1, Maole_002304.T1, Maole_002326.T1, Maole_002396.T1, Maole_002461.T1, Ma
000886.T1, Maole_000893.T1, Maole_000905.T1, Maole_000963.T1, Maole_001017.T1, Ma
002693.T1, Maole_002704.T1, Maole_002715.T1, Maole_002721.T1, Maole_002724.T1, Ma
002724.T1, Maole_002777.T1, Maole_002779.T1, Maole_002782.T1, Maole_002792.T1, Ma
002777.T1, Maole_002778.T1, Maole_002779.T1, Maole_002780.T1, Maole_002782.T1, Ma
002724.T1, Maole_002777.T1, Maole_002779.T1, Maole_002782.T1, Maole_002950.T1, Ma
002724.T1, Maole_002777.T1, Maole_002779.T1, Maole_002782.T1, Maole_002950.T1, Ma
002721.T1, Maole_002724.T1, Maole_002777.T1, Maole_002779.T1, Maole_002782.T1, Ma
001380.T1, Maole_001422.T1, Maole_001467.T1, Maole_001469.T1, Maole_001516.T1, Ma
002396.T1, Maole_002469.T1, Maole_002652.T1, Maole_002704.T1, Maole_002715.T1, Ma
006722.T1, Maole_006992.T1, Maole_007000.T1, Maole_007322.T1, Maole_007323.T1, Ma
009644.T1, Maole_010148.T1, Maole_011670.T1, Maole_011710.T1, Maole_012114.T1, Ma
009092.T1, Maole_009102.T1, Maole_009465.T1, Maole_009883.T1, Maole_009888.T1, Ma
009272.T1, Maole_009320.T1, Maole_010110.T1, Maole_010241.T1, Maole_010336.T1, Ma
002618.T1, Maole_002680.T1, Maole_002736.T1, Maole_002855.T1, Maole_002902.T1, Ma
003356.T1, Maole_003498.T1, Maole_003987.T1, Maole_004070.T1, Maole_004161.T1, Ma
014474.T1, Maole_014475.T1, Maole_014477.T1, Maole_014480.T1, Maole_014484.T1, Ma
004407.T1, Maole_004627.T1, Maole_004673.T1, Maole_004714.T1, Maole_004972.T1, Ma
008946.T1, Maole_009102.T1, Maole_009522.T1, Maole_009883.T1, Maole_009884.T1, Ma
003953.T1, Maole_003987.T1, Maole_004072.T1, Maole_004188.T1, Maole_004189.T1, Ma
014835.T1, Maole_015354.T1, Maole_015835.T1, Maole_015873.T1, Maole_016011.T1, Ma
011930.T1, Maole_013785.T1, Maole_014470.T1, Maole_018021.T1, Maole_019476.T1, Ma
002618.T1, Maole_002627.T1, Maole_002689.T1, Maole_002736.T1, Maole_002854.T1, Ma
006583.T1, Maole_007024.T1, Maole_007342.T1, Maole_007429.T1, Maole_007455.T1, Ma
009364.T1, Maole_009509.T1, Maole_009644.T1, Maole_009740.T1, Maole_010425.T1, Ma
003030.T1, Maole_003100.T1, Maole_003232.T1, Maole_003594.T1, Maole_003610.T1, Ma
005232.T1, Maole_005237.T1, Maole_005293.T1, Maole_005518.T1, Maole_005675.T1, Ma
004268.T1, Maole_004721.T1, Maole_004896.T1, Maole_005244.T1, Maole_005261.T1, Ma
003602.T1, Maole_003848.T1, Maole_003886.T1, Maole_004161.T1, Maole_004972.T1, Ma

017397.T1, Maole_017398.T1, Maole_018850.T1, Maole_018852.T1, Maole_019045.T1, Ma
016201.T1, Maole_016505.T1, Maole_016506.T1, Maole_017713.T1, Maole_019950.T1, Ma
022866.T1, Maole_023076.T1
022732.T1, Maole_022733.T1, Maole_022737.T1

008242.T1, Maole_008946.T1, Maole_009336.T1, Maole_009599.T1, Maole_009725.T1, Ma

012145.T1, Maole_013561.T1, Maole_013863.T1, Maole_014744.T1, Maole_016169.T1, Ma
024121.T1
011930.T1, Maole_011953.T1, Maole_012210.T1, Maole_012318.T1, Maole_012906.T1, Ma
004139.T1, Maole_004167.T1, Maole_004210.T1, Maole_004626.T1, Maole_004787.T1, Ma
018866.T1, Maole_019404.T1, Maole_019405.T1, Maole_019406.T1, Maole_019407.T1, Ma

)21164.T1, Maole_021679.T1, Maole_022600.T1, Maole_023194.T1, Maole_023299.T1
)21185.T1, Maole_022100.T1, Maole_022821.T1, Maole_023076.T1, Maole_024010.T1

)01137.T1, Maole_001163.T1, Maole_001168.T1, Maole_001216.T1, Maole_001240.T1, Ma
)11321.T1, Maole_011322.T1, Maole_011709.T1, Maole_013149.T1, Maole_015027.T1, Ma
)11357.T1, Maole_011358.T1, Maole_012342.T1, Maole_013780.T1, Maole_014534.T1, Ma

)09213.T1, Maole_009320.T1, Maole_009724.T1, Maole_009852.T1, Maole_010110.T1, Ma

)09245.T1, Maole_009522.T1, Maole_009534.T1, Maole_009880.T1, Maole_010418.T1, Ma
)15738.T1, Maole_015838.T1, Maole_023032.T1, Maole_023259.T1, Maole_023260.T1, Ma

)08271.T1, Maole_008809.T1, Maole_008825.T1, Maole_009064.T1, Maole_009178.T1, Ma

)09029.T1, Maole_009092.T1, Maole_009968.T1, Maole_010308.T1, Maole_010427.T1, Ma

)11161.T1, Maole_011519.T1, Maole_011930.T1, Maole_013596.T1, Maole_013889.T1, Ma
)22737.T1
)05101.T1, Maole_005385.T1, Maole_005557.T1, Maole_006068.T1, Maole_006080.T1, Ma
)12817.T1, Maole_012857.T1, Maole_013030.T1, Maole_013163.T1, Maole_013339.T1, Ma
)22844.T1

)21494.T1

)04129.T1, Maole_004161.T1, Maole_004304.T1, Maole_004457.T1, Maole_004564.T1, Ma

)09644.T1, Maole_009918.T1, Maole_010994.T1, Maole_011407.T1, Maole_011507.T1, Ma
)15343.T1, Maole_015577.T1, Maole_016398.T1, Maole_017992.T1, Maole_018063.T1, Ma
)23564.T1, Maole_023753.T1, Maole_023802.T1
)16505.T1, Maole_016506.T1, Maole_016544.T1, Maole_016854.T1, Maole_018032.T1, Ma
)05492.T1, Maole_005709.T1, Maole_006066.T1, Maole_006194.T1, Maole_006468.T1, Ma

)09921.T1, Maole_010383.T1, Maole_012448.T1, Maole_012805.T1, Maole_013174.T1, Ma

)04231.T1, Maole_004721.T1, Maole_004819.T1, Maole_004896.T1, Maole_005896.T1, Ma
)12180.T1, Maole_013116.T1, Maole_013118.T1, Maole_013119.T1, Maole_013610.T1, Ma
)09465.T1, Maole_010021.T1, Maole_010035.T1, Maole_010293.T1, Maole_011128.T1, Ma

)23776.T1, Maole_024121.T1

007391.T1, Maole_007462.T1, Maole_007973.T1, Maole_008670.T1, Maole_008809.T1, Ma

012805.T1, Maole_013299.T1, Maole_013870.T1, Maole_013950.T1, Maole_015577.T1, Ma

014047.T1, Maole_014059.T1, Maole_015044.T1, Maole_015423.T1, Maole_016166.T1, Ma
016686.T1, Maole_016855.T1, Maole_017397.T1, Maole_017398.T1, Maole_018140.T1, Ma
009100.T1, Maole_009432.T1, Maole_009720.T1, Maole_010277.T1, Maole_010472.T1, Ma

004139.T1, Maole_004206.T1, Maole_004319.T1, Maole_004601.T1, Maole_004787.T1, Ma

017713.T1, Maole_018075.T1, Maole_019031.T1, Maole_019219.T1, Maole_020438.T1, Ma

011519.T1, Maole_011589.T1, Maole_011929.T1, Maole_012257.T1, Maole_012405.T1, Ma

012596.T1, Maole_012598.T1, Maole_012707.T1, Maole_012819.T1, Maole_014400.T1, Ma

012308.T1, Maole_012428.T1, Maole_013121.T1, Maole_014269.T1, Maole_014270.T1, Ma
009038.T1, Maole_010120.T1, Maole_011377.T1, Maole_011378.T1, Maole_012093.T1, Ma
005654.T1, Maole_005983.T1, Maole_006356.T1, Maole_007386.T1, Maole_007757.T1, Ma

018033.T1, Maole_018034.T1, Maole_018035.T1, Maole_018059.T1, Maole_019159.T1, Ma
018577.T1, Maole_018644.T1, Maole_020639.T1, Maole_021894.T1, Maole_024085.T1

016457.T1, Maole_016696.T1, Maole_018100.T1, Maole_020721.T1, Maole_021905.T1, Ma

010115.T1, Maole_010254.T1, Maole_010356.T1, Maole_010359.T1, Maole_010362.T1, Ma
013616.T1, Maole_015908.T1, Maole_015909.T1, Maole_016086.T1, Maole_016671.T1, Ma

007046.T1, Maole_007386.T1, Maole_007927.T1, Maole_008546.T1, Maole_009011.T1, Ma

022414.T1

010944.T1, Maole_010945.T1, Maole_010946.T1, Maole_012497.T1, Maole_012499.T1, Ma

019310.T1, Maole_020367.T1, Maole_020368.T1, Maole_020370.T1, Maole_021892.T1, Ma

016416.T1, Maole_016448.T1, Maole_016521.T1, Maole_016525.T1, Maole_016775.T1, Ma

015083.T1, Maole_015471.T1, Maole_016505.T1, Maole_016506.T1, Maole_016544.T1, Ma

019310.T1, Maole_020367.T1, Maole_020368.T1, Maole_020370.T1, Maole_022414.T1
019310.T1, Maole_020367.T1, Maole_020368.T1, Maole_020370.T1, Maole_022414.T1

017131.T1, Maole_019235.T1, Maole_019936.T1, Maole_020979.T1, Maole_021632.T1, Ma

023753.T1
020226.T1, Maole_020651.T1, Maole_020652.T1, Maole_020940.T1, Maole_021069.T1, Ma
019416.T1, Maole_020721.T1, Maole_022283.T1, Maole_023496.T1, Maole_023917.T1, Ma

010337.T1, Maole_010389.T1, Maole_011827.T1, Maole_011873.T1, Maole_012093.T1, Ma

012082.T1, Maole_012227.T1, Maole_012853.T1, Maole_013045.T1, Maole_013709.T1, Ma

014088.T1, Maole_014555.T1, Maole_014721.T1, Maole_014744.T1, Maole_014766.T1, Ma
016981.T1, Maole_017131.T1, Maole_018571.T1, Maole_019950.T1, Maole_020092.T1, Ma

012598.T1, Maole_012666.T1, Maole_012805.T1, Maole_012819.T1, Maole_013299.T1, Ma

017623.T1, Maole_018059.T1, Maole_021905.T1, Maole_022760.T1, Maole_023851.T1

001561.T1, Maole_001608.T1, Maole_001749.T1, Maole_001826.T1, Maole_001845.T1, Ma
021573.T1

018073.T1, Maole_018122.T1, Maole_018123.T1, Maole_018139.T1, Maole_019057.T1, Ma

021416.T1, Maole_022121.T1, Maole_022326.T1, Maole_023129.T1, Maole_023130.T1, Ma

019580.T1, Maole_020830.T1, Maole_022482.T1, Maole_023299.T1

012707.T1, Maole_015735.T1, Maole_015737.T1, Maole_015738.T1, Maole_016181.T1, Ma

013721.T1, Maole_014451.T1, Maole_014453.T1, Maole_014705.T1, Maole_015061.T1, Ma

005654.T1, Maole_005846.T1, Maole_005902.T1, Maole_006031.T1, Maole_006085.T1, Ma

016542.T1, Maole_017166.T1, Maole_017261.T1, Maole_017653.T1, Maole_018595.T1, Ma
016671.T1, Maole_016806.T1, Maole_018253.T1, Maole_018810.T1, Maole_019308.T1, Ma

015393.T1, Maole_016086.T1, Maole_016087.T1, Maole_016088.T1, Maole_016687.T1, Ma

007108.T1, Maole_007294.T1, Maole_007322.T1, Maole_007323.T1, Maole_007500.T1, Ma

003092.T1, Maole_003356.T1, Maole_003778.T1, Maole_004150.T1, Maole_004500.T1, Ma

022052.T1, Maole_023357.T1, Maole_023563.T1

011052.T1, Maole_012524.T1, Maole_012678.T1, Maole_012828.T1, Maole_013222.T1, Ma

014435.T1, Maole_014517.T1, Maole_014685.T1, Maole_015032.T1, Maole_015137.T1, Ma

016463.T1, Maole_016467.T1, Maole_016855.T1, Maole_017397.T1, Maole_017398.T1, Ma

010606.T1, Maole_011642.T1, Maole_011804.T1, Maole_011964.T1, Maole_012220.T1, Ma

)06485.T1, Maole_006651.T1, Maole_006652.T1, Maole_006885.T1, Maole_007126.T1, Ma

aole_000945.T1, Maole_000995.T1, Maole_001011.T1, Maole_001017.T1, Maole_001019.T
aole_000900.T1, Maole_000921.T1, Maole_000922.T1, Maole_000934.T1, Maole_000944.T
aole_001467.T1, Maole_001469.T1, Maole_001501.T1, Maole_001516.T1, Maole_001545.T
aole_002469.T1, Maole_002516.T1, Maole_002534.T1, Maole_002693.T1, Maole_002704.T
aole_001019.T1, Maole_001020.T1, Maole_001021.T1, Maole_001022.T1, Maole_001037.T
aole_002777.T1, Maole_002778.T1, Maole_002779.T1, Maole_002780.T1, Maole_002782.T
aole_002950.T1, Maole_002955.T1, Maole_003283.T1, Maole_003326.T1, Maole_003557.T
aole_002792.T1, Maole_002950.T1, Maole_002955.T1, Maole_003283.T1, Maole_003326.T
aole_002955.T1, Maole_003283.T1, Maole_003326.T1, Maole_003557.T1, Maole_003560.T
aole_002955.T1, Maole_003283.T1, Maole_003326.T1, Maole_003557.T1, Maole_003560.T
aole_002950.T1, Maole_002955.T1, Maole_003283.T1, Maole_003326.T1, Maole_003557.T
aole_001538.T1, Maole_001566.T1, Maole_001608.T1, Maole_001609.T1, Maole_001616.T
aole_002747.T1, Maole_002777.T1, Maole_002778.T1, Maole_002779.T1, Maole_002780.T
aole_007381.T1, Maole_007383.T1, Maole_007473.T1, Maole_007979.T1, Maole_007982.T
aole_012116.T1, Maole_012158.T1, Maole_012375.T1, Maole_012507.T1, Maole_012809.T
aole_010427.T1, Maole_011595.T1, Maole_011678.T1, Maole_011813.T1, Maole_011873.T
aole_011128.T1, Maole_011519.T1, Maole_011670.T1, Maole_011885.T1, Maole_012116.T
aole_003084.T1, Maole_003232.T1, Maole_003498.T1, Maole_003778.T1, Maole_003825.T
aole_004251.T1, Maole_004337.T1, Maole_004500.T1, Maole_004696.T1, Maole_004697.T
aole_014487.T1, Maole_014488.T1, Maole_014490.T1, Maole_016363.T1, Maole_018590.T
aole_005074.T1, Maole_005232.T1, Maole_005237.T1, Maole_005293.T1, Maole_005518.T
aole_009885.T1, Maole_010920.T1, Maole_011614.T1, Maole_011616.T1, Maole_011813.T
aole_005074.T1, Maole_005232.T1, Maole_005237.T1, Maole_005293.T1, Maole_005518.T
aole_016032.T1, Maole_016059.T1, Maole_016410.T1, Maole_016970.T1, Maole_017094.T
aole_020499.T1, Maole_020500.T1, Maole_021520.T1, Maole_022973.T1, Maole_023032.T
aole_002913.T1, Maole_003066.T1, Maole_003084.T1, Maole_003092.T1, Maole_003101.T
aole_008703.T1, Maole_009336.T1, Maole_009465.T1, Maole_009880.T1, Maole_009883.T
aole_011670.T1, Maole_011710.T1, Maole_011885.T1, Maole_012116.T1, Maole_012158.T
aole_003773.T1, Maole_003976.T1, Maole_004113.T1, Maole_004164.T1, Maole_004167.T
aole_006085.T1, Maole_006351.T1, Maole_006468.T1, Maole_006649.T1, Maole_006718.T
aole_005453.T1, Maole_005675.T1, Maole_005998.T1, Maole_006083.T1, Maole_006085.T
aole_005101.T1, Maole_005250.T1, Maole_005421.T1, Maole_005483.T1, Maole_005607.T

aole_019046.T1, Maole_019047.T1, Maole_019049.T1, Maole_019477.T1, Maole_019478.T
aole_020238.T1, Maole_020690.T1, Maole_023564.T1, Maole_023753.T1

aole_010472.T1, Maole_010549.T1, Maole_010606.T1, Maole_011275.T1, Maole_011288.T

aole_016201.T1, Maole_016237.T1, Maole_016473.T1, Maole_016478.T1, Maole_016978.T

aole_015116.T1, Maole_017166.T1, Maole_017261.T1, Maole_017653.T1, Maole_017922.T
aole_004819.T1, Maole_005005.T1, Maole_005102.T1, Maole_005902.T1, Maole_006066.T
aole_019416.T1, Maole_021679.T1, Maole_023357.T1

aole_001419.T1, Maole_001481.T1, Maole_001523.T1, Maole_001608.T1, Maole_001616.T
aole_015085.T1, Maole_015629.T1, Maole_016343.T1, Maole_016345.T1, Maole_016665.T
aole_015044.T1, Maole_015677.T1, Maole_016166.T1, Maole_016253.T1, Maole_016393.T

aole_010241.T1, Maole_010293.T1, Maole_010336.T1, Maole_010601.T1, Maole_010642.T

aole_010423.T1, Maole_010569.T1, Maole_010599.T1, Maole_010780.T1, Maole_011110.T
aole_023776.T1, Maole_024056.T1, Maole_024121.T1

aole_009209.T1, Maole_009494.T1, Maole_009791.T1, Maole_009817.T1, Maole_009961.T

aole_010461.T1, Maole_010849.T1, Maole_010920.T1, Maole_011345.T1, Maole_011352.T

aole_014265.T1, Maole_014267.T1, Maole_014269.T1, Maole_014270.T1, Maole_014272.T

aole_006391.T1, Maole_006559.T1, Maole_007024.T1, Maole_007189.T1, Maole_007322.T
aole_013689.T1, Maole_014314.T1, Maole_014602.T1, Maole_014685.T1, Maole_015679.T

aole_004589.T1, Maole_004862.T1, Maole_005046.T1, Maole_005101.T1, Maole_005102.T

aole_011508.T1, Maole_011804.T1, Maole_011880.T1, Maole_012004.T1, Maole_013130.T
aole_018540.T1, Maole_018541.T1, Maole_019901.T1, Maole_020438.T1, Maole_021240.T

aole_018073.T1, Maole_019590.T1, Maole_020525.T1, Maole_021075.T1, Maole_021076.T
aole_006561.T1, Maole_006647.T1, Maole_007046.T1, Maole_007360.T1, Maole_008264.T

aole_013299.T1, Maole_017163.T1, Maole_017544.T1, Maole_017660.T1, Maole_017900.T

aole_005897.T1, Maole_005944.T1, Maole_006066.T1, Maole_006189.T1, Maole_006194.T
aole_013744.T1, Maole_013763.T1, Maole_013926.T1, Maole_014580.T1, Maole_015629.T
aole_011813.T1, Maole_011873.T1, Maole_012335.T1, Maole_012819.T1, Maole_013422.T

aole_008839.T1, Maole_009245.T1, Maole_009364.T1, Maole_009509.T1, Maole_009636.T

aole_015766.T1, Maole_015932.T1, Maole_016525.T1, Maole_016612.T1, Maole_016971.T

aole_016169.T1, Maole_016446.T1, Maole_016657.T1, Maole_016770.T1, Maole_016970.T
aole_018651.T1, Maole_018850.T1, Maole_018852.T1, Maole_019425.T1, Maole_019929.T
aole_010479.T1, Maole_011181.T1, Maole_011515.T1, Maole_011642.T1, Maole_012665.T

aole_005046.T1, Maole_005102.T1, Maole_005408.T1, Maole_005902.T1, Maole_005908.T

aole_020819.T1, Maole_022051.T1, Maole_022090.T1, Maole_022471.T1, Maole_023753.T

aole_014270.T1, Maole_014275.T1, Maole_014312.T1, Maole_014516.T1, Maole_014704.T

aole_014603.T1, Maole_014847.T1, Maole_015027.T1, Maole_015140.T1, Maole_015785.T

aole_016175.T1, Maole_016266.T1, Maole_016349.T1, Maole_016696.T1, Maole_021415.T
aole_012805.T1, Maole_013299.T1, Maole_013332.T1, Maole_015844.T1, Maole_017163.T
aole_007758.T1, Maole_007827.T1, Maole_007927.T1, Maole_008077.T1, Maole_008318.T

aole_019820.T1, Maole_022265.T1, Maole_022266.T1, Maole_023245.T1, Maole_023837.T

aole_022760.T1, Maole_023851.T1

aole_010419.T1, Maole_011059.T1, Maole_011264.T1, Maole_012626.T1, Maole_012627.T
aole_016806.T1, Maole_018033.T1, Maole_018034.T1, Maole_018035.T1, Maole_018253.T

aole_009918.T1, Maole_010338.T1, Maole_010339.T1, Maole_010982.T1, Maole_010994.T

aole_012501.T1, Maole_012757.T1, Maole_013082.T1, Maole_013616.T1, Maole_015393.T

aole_022414.T1

aole_016978.T1, Maole_017403.T1, Maole_019513.T1, Maole_020238.T1, Maole_020402.T

aole_016854.T1, Maole_018032.T1, Maole_018073.T1, Maole_019590.T1, Maole_020525.T

aole_023357.T1, Maole_023491.T1

aole_021208.T1, Maole_022768.T1
aole_023968.T1

aole_012222.T1, Maole_012520.T1, Maole_012595.T1, Maole_012596.T1, Maole_012598.T

aole_014904.T1, Maole_015908.T1, Maole_015909.T1, Maole_016226.T1, Maole_016671.T

aole_014948.T1, Maole_015921.T1, Maole_016230.T1, Maole_016525.T1, Maole_016657.T
aole_020107.T1, Maole_020979.T1, Maole_022187.T1, Maole_023357.T1

aole_013579.T1, Maole_013950.T1, Maole_014400.T1, Maole_014555.T1, Maole_015577.T

aole_001853.T1, Maole_001907.T1, Maole_001920.T1, Maole_001947.T1, Maole_001948.T

aole_019306.T1, Maole_021091.T1

aole_023131.T1, Maole_023259.T1, Maole_023260.T1, Maole_023776.T1, Maole_024056.T

aole_018063.T1, Maole_018123.T1, Maole_020436.T1, Maole_020479.T1, Maole_021900.T

aole_016032.T1, Maole_016245.T1, Maole_016410.T1, Maole_016416.T1, Maole_016470.T

aole_006356.T1, Maole_006559.T1, Maole_006756.T1, Maole_007143.T1, Maole_007145.T

aole_018604.T1, Maole_018605.T1, Maole_020548.T1, Maole_021877.T1, Maole_022283.T
aole_019310.T1, Maole_020367.T1, Maole_020368.T1, Maole_020370.T1, Maole_021892.T

aole_017214.T1, Maole_018460.T1, Maole_018461.T1, Maole_018463.T1, Maole_018465.T

aole_008401.T1, Maole_009038.T1, Maole_010356.T1, Maole_010359.T1, Maole_010362.T

aole_004771.T1, Maole_005101.T1, Maole_005236.T1, Maole_005293.T1, Maole_005514.T

aole_013359.T1, Maole_014579.T1, Maole_014831.T1, Maole_014832.T1, Maole_015154.T

aole_015838.T1, Maole_016067.T1, Maole_017861.T1, Maole_018625.T1, Maole_018627.T

aole_017460.T1, Maole_018850.T1, Maole_018852.T1

aole_013065.T1, Maole_013253.T1, Maole_013335.T1, Maole_013744.T1, Maole_013863.T

aole_007148.T1, Maole_007320.T1, Maole_007323.T1, Maole_007823.T1, Maole_008299.T

1, Maole_001020.T1, Maole_001021.T1, Maole_001022.T1, Maole_001071.T1, Maole_001
1, Maole_000995.T1, Maole_001011.T1, Maole_001013.T1, Maole_001019.T1, Maole_001
1, Maole_001608.T1, Maole_001610.T1, Maole_001612.T1, Maole_001616.T1, Maole_001
1, Maole_002715.T1, Maole_002721.T1, Maole_002724.T1, Maole_002742.T1, Maole_002
1, Maole_001038.T1, Maole_001096.T1, Maole_001097.T1, Maole_001136.T1, Maole_001
1, Maole_002792.T1, Maole_002950.T1, Maole_002955.T1, Maole_003283.T1, Maole_003
1, Maole_003560.T1, Maole_003905.T1, Maole_003994.T1, Maole_004229.T1, Maole_004
1, Maole_003557.T1, Maole_003560.T1, Maole_003905.T1, Maole_003994.T1, Maole_004
1, Maole_003905.T1, Maole_003994.T1, Maole_004229.T1, Maole_004238.T1, Maole_004
1, Maole_003905.T1, Maole_003994.T1, Maole_004229.T1, Maole_004238.T1, Maole_004
1, Maole_003560.T1, Maole_003905.T1, Maole_003994.T1, Maole_004229.T1, Maole_004
1, Maole_001722.T1, Maole_001948.T1, Maole_001968.T1, Maole_001986.T1, Maole_001
1, Maole_002782.T1, Maole_002792.T1, Maole_002950.T1, Maole_002955.T1, Maole_003
1, Maole_008636.T1, Maole_008747.T1, Maole_008763.T1, Maole_008839.T1, Maole_009
1, Maole_013456.T1, Maole_014466.T1, Maole_014469.T1, Maole_014470.T1, Maole_014
1, Maole_013121.T1, Maole_013416.T1, Maole_015626.T1, Maole_015757.T1, Maole_015
1, Maole_012405.T1, Maole_012507.T1, Maole_013130.T1, Maole_013299.T1, Maole_014
1, Maole_003987.T1, Maole_004003.T1, Maole_004072.T1, Maole_004230.T1, Maole_004
1, Maole_004896.T1, Maole_005102.T1, Maole_005152.T1, Maole_005421.T1, Maole_005
1, Maole_018592.T1, Maole_018595.T1, Maole_018597.T1, Maole_018598.T1, Maole_018
1, Maole_005675.T1, Maole_006040.T1, Maole_006085.T1, Maole_006351.T1, Maole_006
1, Maole_012819.T1, Maole_013454.T1, Maole_014744.T1, Maole_014963.T1, Maole_014
1, Maole_005675.T1, Maole_005998.T1, Maole_006085.T1, Maole_006468.T1, Maole_006
1, Maole_017395.T1, Maole_018026.T1, Maole_018086.T1, Maole_018847.T1, Maole_019
1, Maole_023259.T1, Maole_023260.T1, Maole_023561.T1, Maole_023562.T1, Maole_023
1, Maole_003148.T1, Maole_003315.T1, Maole_003498.T1, Maole_003522.T1, Maole_003
1, Maole_009884.T1, Maole_009885.T1, Maole_010461.T1, Maole_010606.T1, Maole_011
1, Maole_012405.T1, Maole_012507.T1, Maole_012805.T1, Maole_012809.T1, Maole_012
1, Maole_004210.T1, Maole_004231.T1, Maole_004363.T1, Maole_004819.T1, Maole_004
1, Maole_007035.T1, Maole_007183.T1, Maole_007285.T1, Maole_007427.T1, Maole_007
1, Maole_006189.T1, Maole_006235.T1, Maole_006236.T1, Maole_006480.T1, Maole_006
1, Maole_005818.T1, Maole_005902.T1, Maole_006031.T1, Maole_006211.T1, Maole_006

1, Maole_022891.T1

1, Maole_011444.T1, Maole_011642.T1, Maole_012520.T1, Maole_012595.T1, Maole_012

1, Maole_017713.T1, Maole_018075.T1, Maole_018141.T1, Maole_019299.T1, Maole_019

1, Maole_019057.T1, Maole_020499.T1, Maole_022090.T1, Maole_022283.T1, Maole_023
1, Maole_006083.T1, Maole_006194.T1, Maole_006295.T1, Maole_006703.T1, Maole_006

[1, Maole_001725.T1, Maole_001749.T1, Maole_001826.T1, Maole_001907.T1, Maole_001
[1, Maole_016736.T1, Maole_016806.T1, Maole_016855.T1, Maole_017085.T1, Maole_017
[1, Maole_020501.T1, Maole_020502.T1, Maole_020819.T1, Maole_022100.T1, Maole_022

[1, Maole_011216.T1, Maole_011406.T1, Maole_011519.T1, Maole_011930.T1, Maole_013

[1, Maole_011111.T1, Maole_011159.T1, Maole_011288.T1, Maole_011321.T1, Maole_011

[1, Maole_010589.T1, Maole_010601.T1, Maole_011377.T1, Maole_011378.T1, Maole_011

[1, Maole_011401.T1, Maole_011595.T1, Maole_011678.T1, Maole_011975.T1, Maole_012

[1, Maole_014274.T1, Maole_014275.T1, Maole_014711.T1, Maole_015577.T1, Maole_016

[1, Maole_007323.T1, Maole_007462.T1, Maole_007859.T1, Maole_007912.T1, Maole_008
[1, Maole_016444.T1, Maole_016890.T1, Maole_017476.T1, Maole_017839.T1, Maole_018

[1, Maole_005408.T1, Maole_005483.T1, Maole_005505.T1, Maole_005607.T1, Maole_005

[1, Maole_013486.T1, Maole_013607.T1, Maole_013889.T1, Maole_014641.T1, Maole_015
[1, Maole_023519.T1, Maole_023523.T1

[1, Maole_021089.T1, Maole_021207.T1, Maole_022973.T1
[1, Maole_008512.T1, Maole_008546.T1, Maole_008839.T1, Maole_009267.T1, Maole_009

[1, Maole_018604.T1, Maole_018605.T1, Maole_018644.T1, Maole_019175.T1, Maole_019

[1, Maole_006295.T1, Maole_006391.T1, Maole_006485.T1, Maole_006897.T1, Maole_007
[1, Maole_017109.T1, Maole_017124.T1, Maole_017220.T1, Maole_017313.T1, Maole_017
[1, Maole_013561.T1, Maole_014088.T1, Maole_014721.T1, Maole_014744.T1, Maole_014

1, Maole_009644.T1, Maole_009945.T1, Maole_010569.T1, Maole_011601.T1, Maole_011

1, Maole_016977.T1, Maole_016978.T1, Maole_017222.T1, Maole_017331.T1, Maole_017

1, Maole_017128.T1, Maole_018033.T1, Maole_018034.T1, Maole_018035.T1, Maole_018
1, Maole_020092.T1, Maole_020441.T1, Maole_021130.T1, Maole_021530.T1, Maole_021
1, Maole_013157.T1, Maole_013230.T1, Maole_013335.T1, Maole_013695.T1, Maole_014

1, Maole_006933.T1, Maole_007037.T1, Maole_007143.T1, Maole_007145.T1, Maole_007

1, Maole_023893.T1

1, Maole_014711.T1, Maole_015305.T1, Maole_015657.T1, Maole_015737.T1, Maole_015

1, Maole_015802.T1, Maole_015962.T1, Maole_016169.T1, Maole_016455.T1, Maole_016

1, Maole_022760.T1, Maole_023332.T1, Maole_023728.T1, Maole_023851.T1, Maole_024
1, Maole_017313.T1, Maole_017544.T1, Maole_017900.T1, Maole_018644.T1, Maole_020
1, Maole_008734.T1, Maole_009505.T1, Maole_009506.T1, Maole_009817.T1, Maole_009

1

T1, Maole_012789.T1, Maole_012830.T1, Maole_013216.T1, Maole_013625.T1, Maole_014
T1, Maole_018810.T1, Maole_019308.T1, Maole_019310.T1, Maole_019820.T1, Maole_020

T1, Maole_011289.T1, Maole_011642.T1, Maole_012360.T1, Maole_012448.T1, Maole_012

T1, Maole_016086.T1, Maole_016087.T1, Maole_016088.T1, Maole_016687.T1, Maole_017

T1, Maole_020589.T1, Maole_021475.T1, Maole_022497.T1

T1, Maole_020690.T1, Maole_021075.T1, Maole_021076.T1, Maole_021089.T1, Maole_021

1, Maole_012809.T1, Maole_013630.T1, Maole_014400.T1, Maole_014496.T1, Maole_014

1, Maole_016806.T1, Maole_018253.T1, Maole_018563.T1, Maole_018810.T1, Maole_019

1, Maole_016951.T1, Maole_017163.T1, Maole_017237.T1, Maole_017309.T1, Maole_017

1, Maole_015835.T1, Maole_015844.T1, Maole_015932.T1, Maole_016455.T1, Maole_016

1, Maole_002162.T1, Maole_002221.T1, Maole_002276.T1, Maole_002389.T1, Maole_002

1, Maole_024121.T1

1, Maole_022187.T1, Maole_022452.T1, Maole_023564.T1, Maole_023753.T1

1, Maole_016521.T1, Maole_016735.T1, Maole_016877.T1, Maole_017403.T1, Maole_017

1, Maole_007236.T1, Maole_007322.T1, Maole_007323.T1, Maole_008020.T1, Maole_008

1, Maole_022700.T1, Maole_022906.T1, Maole_023053.T1
1, Maole_022414.T1

1, Maole_018928.T1, Maole_018929.T1, Maole_018931.T1, Maole_018932.T1, Maole_019

[illegible]1, Maole_010425.T1, Maole_010461.T1, Maole_011407.T1, Maole_011523.T1, Maole_011

[illegible]1, Maole_005935.T1, Maole_006444.T1, Maole_006445.T1, Maole_006559.T1, Maole_006

[illegible]1, Maole_015610.T1, Maole_016115.T1, Maole_016544.T1, Maole_016700.T1, Maole_016

[illegible]1, Maole_019320.T1, Maole_019439.T1, Maole_021300.T1

[illegible]1, Maole_014435.T1, Maole_014926.T1, Maole_015032.T1, Maole_015137.T1, Maole_015

[illegible]1, Maole_008315.T1, Maole_008338.T1, Maole_008505.T1, Maole_008706.T1, Maole_008

097.T1, Maole_001100.T1, Maole_001120.T1, Maole_001123.T1, Maole_001124.T1, Maole
020.T1, Maole_001021.T1, Maole_001022.T1, Maole_001037.T1, Maole_001096.T1, Maole
631.T1, Maole_001749.T1, Maole_001757.T1, Maole_001879.T1, Maole_001904.T1, Maole
747.T1, Maole_002777.T1, Maole_002778.T1, Maole_002779.T1, Maole_002780.T1, Maole
137.T1, Maole_001138.T1, Maole_001240.T1, Maole_001277.T1, Maole_001301.T1, Maole
326.T1, Maole_003557.T1, Maole_003560.T1, Maole_003905.T1, Maole_003994.T1, Maole
238.T1, Maole_004246.T1, Maole_004406.T1, Maole_004432.T1, Maole_004459.T1, Maole
229.T1, Maole_004238.T1, Maole_004246.T1, Maole_004406.T1, Maole_004432.T1, Maole
246.T1, Maole_004406.T1, Maole_004432.T1, Maole_004459.T1, Maole_004600.T1, Maole
246.T1, Maole_004406.T1, Maole_004432.T1, Maole_004459.T1, Maole_004600.T1, Maole
238.T1, Maole_004246.T1, Maole_004406.T1, Maole_004432.T1, Maole_004459.T1, Maole
987.T1, Maole_002162.T1, Maole_002189.T1, Maole_002191.T1, Maole_002221.T1, Maole
283.T1, Maole_003309.T1, Maole_003326.T1, Maole_003475.T1, Maole_003525.T1, Maole
003.T1, Maole_009053.T1, Maole_009054.T1, Maole_009056.T1, Maole_009267.T1, Maole
471.T1, Maole_014474.T1, Maole_014475.T1, Maole_014477.T1, Maole_014480.T1, Maole
759.T1, Maole_015760.T1, Maole_015762.T1, Maole_016010.T1, Maole_016575.T1, Maole
466.T1, Maole_014475.T1, Maole_014477.T1, Maole_014487.T1, Maole_014488.T1, Maole
319.T1, Maole_004337.T1, Maole_004407.T1, Maole_004582.T1, Maole_004697.T1, Maole
432.T1, Maole_005461.T1, Maole_005483.T1, Maole_005518.T1, Maole_005877.T1, Maole
599.T1, Maole_018603.T1, Maole_018604.T1, Maole_018605.T1, Maole_018866.T1, Maole
468.T1, Maole_006649.T1, Maole_006702.T1, Maole_006718.T1, Maole_006799.T1, Maole
964.T1, Maole_015044.T1, Maole_015423.T1, Maole_016253.T1, Maole_016657.T1, Maole
649.T1, Maole_006718.T1, Maole_006756.T1, Maole_007631.T1, Maole_007690.T1, Maole
951.T1, Maole_020000.T1, Maole_020066.T1, Maole_020351.T1, Maole_020655.T1, Maole
563.T1, Maole_023564.T1, Maole_023753.T1, Maole_023776.T1, Maole_024121.T1
778.T1, Maole_003848.T1, Maole_003953.T1, Maole_003987.T1, Maole_004072.T1, Maole
288.T1, Maole_011512.T1, Maole_011614.T1, Maole_011616.T1, Maole_011867.T1, Maole
820.T1, Maole_013174.T1, Maole_013299.T1, Maole_013408.T1, Maole_013579.T1, Maole
896.T1, Maole_005102.T1, Maole_005152.T1, Maole_005276.T1, Maole_005432.T1, Maole
690.T1, Maole_007855.T1, Maole_008706.T1, Maole_008707.T1, Maole_009038.T1, Maole
521.T1, Maole_006799.T1, Maole_006850.T1, Maole_006933.T1, Maole_007285.T1, Maole
356.T1, Maole_006559.T1, Maole_007143.T1, Maole_007145.T1, Maole_007148.T1, Maole

596.T1, Maole_012598.T1, Maole_012707.T1, Maole_013335.T1, Maole_014339.T1, Maole

929.T1, Maole_020238.T1, Maole_021503.T1, Maole_021573.T1, Maole_023564.T1, Maole

032.T1, Maole_023259.T1, Maole_023260.T1, Maole_023418.T1, Maole_023564.T1, Maole
850.T1, Maole_007108.T1, Maole_007143.T1, Maole_007145.T1, Maole_007236.T1, Maole

918.T1, Maole_001936.T1, Maole_001947.T1, Maole_001971.T1, Maole_002067.T1, Maole
086.T1, Maole_017087.T1, Maole_017092.T1, Maole_017109.T1, Maole_018059.T1, Maole
471.T1, Maole_022821.T1

045.T1, Maole_013163.T1, Maole_013792.T1, Maole_013863.T1, Maole_014088.T1, Maole

322.T1, Maole_011512.T1, Maole_011867.T1, Maole_012819.T1, Maole_013561.T1, Maole

706.T1, Maole_011762.T1, Maole_012434.T1, Maole_012649.T1, Maole_012661.T1, Maole

082.T1, Maole_012299.T1, Maole_012301.T1, Maole_012375.T1, Maole_013121.T1, Maole

217.T1, Maole_016457.T1, Maole_016925.T1, Maole_017719.T1, Maole_020499.T1, Maole

382.T1, Maole_008512.T1, Maole_008800.T1, Maole_008868.T1, Maole_009337.T1, Maole
075.T1, Maole_018863.T1, Maole_018864.T1, Maole_019427.T1, Maole_019764.T1, Maole

908.T1, Maole_006095.T1, Maole_006365.T1, Maole_006559.T1, Maole_007108.T1, Maole

305.T1, Maole_016032.T1, Maole_016242.T1, Maole_016970.T1, Maole_017133.T1, Maole

268.T1, Maole_009446.T1, Maole_009918.T1, Maole_010994.T1, Maole_011085.T1, Maole

562.T1, Maole_021063.T1, Maole_021212.T1, Maole_021727.T1, Maole_023235.T1, Maole

035.T1, Maole_007108.T1, Maole_007473.T1, Maole_008489.T1, Maole_008505.T1, Maole
718.T1, Maole_018140.T1, Maole_018579.T1, Maole_020107.T1, Maole_020684.T1, Maole
835.T1, Maole_014948.T1, Maole_015324.T1, Maole_015354.T1, Maole_016104.T1, Maole

710.T1, Maole_011761.T1, Maole_011764.T1, Maole_012088.T1, Maole_012158.T1, Maole

644.T1, Maole_017861.T1, Maole_018571.T1, Maole_019118.T1, Maole_019137.T1, Maole

327.T1, Maole_019820.T1, Maole_020517.T1, Maole_021909.T1, Maole_023245.T1, Maole
573.T1, Maole_022051.T1
435.T1, Maole_014746.T1, Maole_015032.T1, Maole_015137.T1, Maole_015543.T1, Maole

176.T1, Maole_007189.T1, Maole_008141.T1, Maole_008143.T1, Maole_008465.T1, Maole

794.T1, Maole_016410.T1, Maole_017065.T1, Maole_017432.T1, Maole_017922.T1, Maole

662.T1, Maole_017245.T1, Maole_017515.T1, Maole_017595.T1, Maole_017673.T1, Maole

091.T1
605.T1, Maole_020610.T1, Maole_020826.T1, Maole_021063.T1, Maole_021425.T1, Maole
921.T1, Maole_011406.T1, Maole_011827.T1, Maole_011873.T1, Maole_012162.T1, Maole

669.T1, Maole_014704.T1, Maole_015932.T1, Maole_016226.T1, Maole_016505.T1, Maole
367.T1, Maole_020368.T1, Maole_020370.T1, Maole_021407.T1, Maole_021892.T1, Maole

817.T1, Maole_013339.T1, Maole_013456.T1, Maole_013607.T1, Maole_013763.T1, Maole

214.T1, Maole_018460.T1, Maole_018461.T1, Maole_018463.T1, Maole_018465.T1, Maole

207.T1, Maole_022089.T1

919.T1, Maole_015753.T1, Maole_016181.T1, Maole_016455.T1, Maole_017135.T1, Maole

308.T1, Maole_019310.T1, Maole_020367.T1, Maole_020368.T1, Maole_020370.T1, Maole

383.T1, Maole_017415.T1, Maole_017515.T1, Maole_017713.T1, Maole_017861.T1, Maole

971.T1, Maole_017135.T1, Maole_017222.T1, Maole_017644.T1, Maole_017911.T1, Maole

426.T1, Maole_002458.T1, Maole_002505.T1, Maole_002620.T1, Maole_002680.T1, Maole

586.T1, Maole_019149.T1, Maole_019215.T1, Maole_019513.T1, Maole_021008.T1, Maole

053.T1, Maole_008401.T1, Maole_008489.T1, Maole_008737.T1, Maole_008809.T1, Maole

031.T1, Maole_019219.T1, Maole_019435.T1, Maole_019627.T1, Maole_019629.T1, Maole

678.T1, Maole_011873.T1, Maole_012236.T1, Maole_012467.T1, Maole_012666.T1, Maole

649.T1, Maole_006718.T1, Maole_006885.T1, Maole_007244.T1, Maole_007826.T1, Maole

854.T1, Maole_017223.T1, Maole_018863.T1, Maole_018864.T1, Maole_019146.T1, Maole

653.T1, Maole_015844.T1, Maole_016230.T1, Maole_016242.T1, Maole_016245.T1, Maole

707.T1, Maole_008737.T1, Maole_008842.T1, Maole_008854.T1, Maole_009289.T1, Maole

e_001125.T1, Maole_001136.T1, Maole_001137.T1, Maole_001216.T1, Maole_001240.T1, M
e_001097.T1, Maole_001108.T1, Maole_001120.T1, Maole_001136.T1, Maole_001227.T1, M
e_001931.T1, Maole_001948.T1, Maole_001957.T1, Maole_002056.T1, Maole_002191.T1, M
e_002782.T1, Maole_002792.T1, Maole_002950.T1, Maole_003283.T1, Maole_003309.T1, M
e_001375.T1, Maole_001380.T1, Maole_001467.T1, Maole_001469.T1, Maole_001485.T1, M
e_004229.T1, Maole_004238.T1, Maole_004246.T1, Maole_004406.T1, Maole_004432.T1, M
e_004600.T1, Maole_004798.T1, Maole_004878.T1, Maole_004972.T1, Maole_005013.T1, M
e_004459.T1, Maole_004600.T1, Maole_004798.T1, Maole_004878.T1, Maole_004972.T1, M
e_004798.T1, Maole_004878.T1, Maole_004972.T1, Maole_005013.T1, Maole_005326.T1, M
e_004798.T1, Maole_004878.T1, Maole_004972.T1, Maole_005013.T1, Maole_005326.T1, M
e_004600.T1, Maole_004798.T1, Maole_004878.T1, Maole_004972.T1, Maole_005013.T1, M
e_002239.T1, Maole_002276.T1, Maole_002401.T1, Maole_002411.T1, Maole_002414.T1, M
e_003557.T1, Maole_003560.T1, Maole_003905.T1, Maole_003994.T1, Maole_004229.T1, M
e_009268.T1, Maole_009440.T1, Maole_009441.T1, Maole_010083.T1, Maole_010120.T1, M
e_014484.T1, Maole_014487.T1, Maole_014488.T1, Maole_014490.T1, Maole_014602.T1, M
e_016576.T1, Maole_016686.T1, Maole_016696.T1, Maole_017086.T1, Maole_017713.T1, M
e_014602.T1, Maole_014730.T1, Maole_014835.T1, Maole_015354.T1, Maole_015543.T1, M
e_005074.T1, Maole_005102.T1, Maole_005293.T1, Maole_005461.T1, Maole_005483.T1, M
e_005880.T1, Maole_006036.T1, Maole_006194.T1, Maole_006247.T1, Maole_006351.T1, M
e_019404.T1, Maole_019405.T1, Maole_019406.T1, Maole_019407.T1, Maole_019408.T1, M
e_007035.T1, Maole_007183.T1, Maole_007285.T1, Maole_007427.T1, Maole_007690.T1, M
e_016686.T1, Maole_017163.T1, Maole_017245.T1, Maole_017309.T1, Maole_018059.T1, M
e_007855.T1, Maole_008292.T1, Maole_008616.T1, Maole_009446.T1, Maole_009754.T1, M
e_020968.T1, Maole_021008.T1, Maole_021164.T1, Maole_021679.T1, Maole_022452.T1, M

e_004500.T1, Maole_004598.T1, Maole_004627.T1, Maole_004696.T1, Maole_004819.T1, M
e_011873.T1, Maole_012819.T1, Maole_013147.T1, Maole_013149.T1, Maole_014931.T1, M
e_013870.T1, Maole_014466.T1, Maole_014475.T1, Maole_014477.T1, Maole_014487.T1, M
e_005461.T1, Maole_005492.T1, Maole_005625.T1, Maole_005626.T1, Maole_005709.T1, M
e_009446.T1, Maole_009754.T1, Maole_009796.T1, Maole_010934.T1, Maole_012088.T1, M
e_007473.T1, Maole_007631.T1, Maole_007656.T1, Maole_008447.T1, Maole_008616.T1, M
e_007236.T1, Maole_007366.T1, Maole_007391.T1, Maole_008382.T1, Maole_009064.T1, M

e_014435.T1, Maole_014603.T1, Maole_014847.T1, Maole_015027.T1, Maole_015032.T1, M

e_023753.T1

e_023753.T1, Maole_023776.T1, Maole_024121.T1
e_007360.T1, Maole_007723.T1, Maole_007853.T1, Maole_007944.T1, Maole_008489.T1, M

e_002202.T1, Maole_002221.T1, Maole_002381.T1, Maole_002389.T1, Maole_002408.T1, M
e_019303.T1, Maole_019946.T1, Maole_020206.T1, Maole_020826.T1, Maole_022244.T1, M

e_014228.T1, Maole_014314.T1, Maole_014685.T1, Maole_015236.T1, Maole_016012.T1, M

e_013596.T1, Maole_013780.T1, Maole_014744.T1, Maole_014931.T1, Maole_015061.T1, M

e_012840.T1, Maole_013061.T1, Maole_013497.T1, Maole_013520.T1, Maole_013763.T1, M

e_013153.T1, Maole_013889.T1, Maole_014228.T1, Maole_015061.T1, Maole_016169.T1, M

e_021718.T1, Maole_021969.T1, Maole_021993.T1, Maole_023030.T1, Maole_023753.T1

e_009364.T1, Maole_009466.T1, Maole_009598.T1, Maole_010293.T1, Maole_010480.T1, M
e_019766.T1, Maole_019767.T1, Maole_020187.T1, Maole_022866.T1, Maole_023468.T1, M

e_007272.T1, Maole_007322.T1, Maole_007323.T1, Maole_007462.T1, Maole_007944.T1, M

e_017395.T1, Maole_017679.T1, Maole_018026.T1, Maole_018862.T1, Maole_019509.T1, M

e_012324.T1, Maole_012531.T1, Maole_012627.T1, Maole_012819.T1, Maole_012828.T1, M

e_023419.T1, Maole_024085.T1

e_008713.T1, Maole_008736.T1, Maole_008763.T1, Maole_009053.T1, Maole_009054.T1, M
e_020797.T1, Maole_020940.T1, Maole_021069.T1, Maole_022720.T1, Maole_023469.T1, M
e_016169.T1, Maole_016226.T1, Maole_017280.T1, Maole_017309.T1, Maole_017383.T1, M

e_012507.T1, Maole_012672.T1, Maole_012878.T1, Maole_013112.T1, Maole_014580.T1, M

e_019939.T1, Maole_020683.T1, Maole_020807.T1, Maole_021614.T1, Maole_021727.T1, M

e_023761.T1

e_015653.T1, Maole_017595.T1, Maole_017660.T1, Maole_017900.T1, Maole_018160.T1, M

e_008523.T1, Maole_008706.T1, Maole_008800.T1, Maole_009064.T1, Maole_009432.T1, M

e_018021.T1, Maole_018513.T1, Maole_019476.T1, Maole_020878.T1, Maole_021900.T1, M

e_019177.T1, Maole_019223.T1, Maole_019227.T1, Maole_019303.T1, Maole_019453.T1, M

e_021727.T1, Maole_022720.T1, Maole_023419.T1, Maole_024085.T1
e_012809.T1, Maole_013174.T1, Maole_013332.T1, Maole_013709.T1, Maole_014199.T1, M

e_016506.T1, Maole_016521.T1, Maole_016525.T1, Maole_017222.T1, Maole_017647.T1,
e_022414.T1, Maole_022628.T1, Maole_023245.T1

e_014370.T1, Maole_014963.T1, Maole_014964.T1, Maole_016393.T1, Maole_016696.T1,

e_018928.T1, Maole_018929.T1, Maole_018931.T1, Maole_018932.T1, Maole_019031.T1,

e_017383.T1, Maole_017544.T1, Maole_017861.T1, Maole_017911.T1, Maole_019118.T1, M

e_021407.T1, Maole_021892.T1, Maole_021969.T1, Maole_022347.T1, Maole_022350.T1, M

e_017911.T1, Maole_017922.T1, Maole_018676.T1, Maole_018847.T1, Maole_018869.T1, M

e_019118.T1, Maole_019453.T1, Maole_019939.T1, Maole_020558.T1, Maole_021573.T1, M

e_002693.T1, Maole_002747.T1, Maole_002761.T1, Maole_002818.T1, Maole_002826.T1, M

e_021164.T1, Maole_021289.T1, Maole_021942.T1, Maole_022906.T1, Maole_023290.T1, M

e_009064.T1, Maole_009360.T1, Maole_009636.T1, Maole_009817.T1, Maole_010337.T1, M

e_019630.T1, Maole_020612.T1, Maole_020860.T1, Maole_021407.T1, Maole_021422.T1, M

e_012805.T1, Maole_013299.T1, Maole_014042.T1, Maole_014603.T1, Maole_015140.T1, M

e_008299.T1, Maole_008615.T1, Maole_008839.T1, Maole_009038.T1, Maole_009320.T1, M

e_019391.T1, Maole_019590.T1, Maole_019762.T1, Maole_019764.T1, Maole_019766.T1, M

e_017595.T1, Maole_017718.T1, Maole_017770.T1, Maole_018140.T1, Maole_018625.T1, M

e_009303.T1, Maole_009367.T1, Maole_009921.T1, Maole_010021.T1, Maole_010098.T1, M

Maole_001277.T1, Maole_001301.T1, Maole_001360.T1, Maole_001375.T1, Maole_001403
Maole_001277.T1, Maole_001301.T1, Maole_001317.T1, Maole_001360.T1, Maole_001375
Maole_002221.T1, Maole_002239.T1, Maole_002288.T1, Maole_002304.T1, Maole_002326
Maole_003326.T1, Maole_003475.T1, Maole_003525.T1, Maole_003560.T1, Maole_003905
Maole_001488.T1, Maole_001545.T1, Maole_001561.T1, Maole_001631.T1, Maole_001649
Maole_004459.T1, Maole_004600.T1, Maole_004798.T1, Maole_004878.T1, Maole_004972
Maole_005326.T1, Maole_005478.T1, Maole_005492.T1, Maole_005520.T1, Maole_005622
Maole_005013.T1, Maole_005326.T1, Maole_005478.T1, Maole_005492.T1, Maole_005520
Maole_005478.T1, Maole_005492.T1, Maole_005520.T1, Maole_005622.T1, Maole_005818
Maole_005478.T1, Maole_005492.T1, Maole_005520.T1, Maole_005622.T1, Maole_005818
Maole_005326.T1, Maole_005329.T1, Maole_005478.T1, Maole_005492.T1, Maole_005520
Maole_002456.T1, Maole_002469.T1, Maole_002477.T1, Maole_002620.T1, Maole_002627
Maole_004246.T1, Maole_004406.T1, Maole_004432.T1, Maole_004457.T1, Maole_004548
Maole_010148.T1, Maole_010338.T1, Maole_010339.T1, Maole_011670.T1, Maole_011832
Maole_014641.T1, Maole_014963.T1, Maole_014964.T1, Maole_015044.T1, Maole_016011
Maole_018524.T1, Maole_019968.T1, Maole_020423.T1, Maole_021494.T1, Maole_021905
Maole_015873.T1, Maole_016011.T1, Maole_016032.T1, Maole_016410.T1, Maole_016970
Maole_005675.T1, Maole_005807.T1, Maole_005814.T1, Maole_005908.T1, Maole_006036
Maole_006581.T1, Maole_006583.T1, Maole_006647.T1, Maole_006830.T1, Maole_007024
Maole_019409.T1, Maole_019410.T1, Maole_019412.T1, Maole_019413.T1, Maole_019415
Maole_007855.T1, Maole_008706.T1, Maole_008707.T1, Maole_009317.T1, Maole_009320
Maole_019300.T1, Maole_019968.T1, Maole_020423.T1, Maole_020854.T1, Maole_021285
Maole_010016.T1, Maole_010934.T1, Maole_011155.T1, Maole_011161.T1, Maole_012088
Maole_022600.T1, Maole_022866.T1, Maole_022906.T1, Maole_022983.T1, Maole_022984

Maole_005074.T1, Maole_005232.T1, Maole_005237.T1, Maole_005244.T1, Maole_005293
Maole_015423.T1, Maole_015591.T1, Maole_015794.T1, Maole_016201.T1, Maole_016457
Maole_014488.T1, Maole_014602.T1, Maole_014641.T1, Maole_014721.T1, Maole_015294
Maole_005908.T1, Maole_005930.T1, Maole_006066.T1, Maole_006083.T1, Maole_006194
Maole_012144.T1, Maole_012145.T1, Maole_012180.T1, Maole_012283.T1, Maole_012426
Maole_008706.T1, Maole_008707.T1, Maole_008713.T1, Maole_009267.T1, Maole_009268
Maole_009303.T1, Maole_009317.T1, Maole_009320.T1, Maole_009506.T1, Maole_009921

Maole_015140.T1, Maole_015653.T1, Maole_015659.T1, Maole_015794.T1, Maole_015802

Maole_008713.T1, Maole_008763.T1, Maole_008800.T1, Maole_009064.T1, Maole_009181

Maole_002505.T1, Maole_002516.T1, Maole_002627.T1, Maole_002680.T1, Maole_002706
Maole_022628.T1, Maole_022842.T1, Maole_022844.T1

Maole_016217.T1, Maole_016473.T1, Maole_017043.T1, Maole_017383.T1, Maole_017911

Maole_015591.T1, Maole_015794.T1, Maole_015802.T1, Maole_016343.T1, Maole_016345

Maole_014128.T1, Maole_014236.T1, Maole_014314.T1, Maole_014685.T1, Maole_014838

Maole_016473.T1, Maole_016696.T1, Maole_016770.T1, Maole_017476.T1, Maole_019159

Maole_011381.T1, Maole_011507.T1, Maole_011678.T1, Maole_011710.T1, Maole_012093
Maole_023479.T1, Maole_023519.T1, Maole_023523.T1

Maole_008053.T1, Maole_008143.T1, Maole_008401.T1, Maole_008523.T1, Maole_008706

Maole_019580.T1, Maole_019951.T1, Maole_020103.T1, Maole_020873.T1, Maole_020919

Maole_013216.T1, Maole_013515.T1, Maole_013607.T1, Maole_013625.T1, Maole_013889

Maole_009056.T1, Maole_009114.T1, Maole_009181.T1, Maole_009724.T1, Maole_009796
Maole_023840.T1
Maole_017415.T1, Maole_017713.T1, Maole_017809.T1, Maole_017861.T1, Maole_018069

Maole_014602.T1, Maole_014641.T1, Maole_015407.T1, Maole_015612.T1, Maole_01661[illegible]

Maole_022187.T1, Maole_022367.T1, Maole_022898.T1, Maole_023357.T1

Maole_019070.T1, Maole_019165.T1, Maole_019320.T1, Maole_019439.T1, Maole_01994[illegible]

Maole_009465.T1, Maole_009636.T1, Maole_010277.T1, Maole_010308.T1, Maole_01038[illegible]

Maole_022906.T1, Maole_023447.T1, Maole_023776.T1

Maole_019901.T1, Maole_020823.T1, Maole_020830.T1, Maole_021285.T1, Maole_02145[illegible]

Maole_014397.T1, Maole_014555.T1, Maole_016102.T1, Maole_016253.T1, Maole_01654[illegible]

Maole_018073.T1, Maole_019130.T1, Maole_020589.T1, Maole_021202.T1, Maole_021821

Maole_016881.T1, Maole_017197.T1, Maole_017544.T1, Maole_017660.T1, Maole_017719

Maole_019219.T1, Maole_019435.T1, Maole_019627.T1, Maole_019629.T1, Maole_019630

Maole_020558.T1, Maole_021494.T1, Maole_022367.T1, Maole_022776.T1, Maole_023235

Maole_022414.T1, Maole_022678.T1, Maole_023030.T1

Maole_019137.T1, Maole_019399.T1, Maole_019453.T1, Maole_019520.T1, Maole_019799

Maole_021614.T1, Maole_021727.T1, Maole_022367.T1, Maole_023235.T1

Maole_002955.T1, Maole_003259.T1, Maole_003261.T1, Maole_003286.T1, Maole_003501

Maole_023612.T1, Maole_023762.T1, Maole_023849.T1

Maole_010425.T1, Maole_010934.T1, Maole_011377.T1, Maole_011378.T1, Maole_011411

Maole_022350.T1, Maole_022352.T1, Maole_022678.T1, Maole_022820.T1, Maole_023409

Maole_015294.T1, Maole_015629.T1, Maole_015873.T1, Maole_016012.T1, Maole_01615[illegible]

Maole_009446.T1, Maole_009992.T1, Maole_010016.T1, Maole_010110.T1, Maole_01033[illegible]

Maole_019767.T1, Maole_021014.T1, Maole_021015.T1, Maole_021089.T1

Maole_018627.T1, Maole_018869.T1, Maole_019045.T1, Maole_019046.T1, Maole_019047

Maole_010336.T1, Maole_010457.T1, Maole_010661.T1, Maole_010662.T1, Maole_01083[illegible]

.T1, Maole_001404.T1, Maole_001419.T1, Maole_001420.T1, Maole_001424.T1, Maole_0
.T1, Maole_001390.T1, Maole_001394.T1, Maole_001403.T1, Maole_001419.T1, Maole_0
.T1, Maole_002401.T1, Maole_002406.T1, Maole_002461.T1, Maole_002469.T1, Maole_0
.T1, Maole_003994.T1, Maole_004047.T1, Maole_004229.T1, Maole_004238.T1, Maole_0
.T1, Maole_001703.T1, Maole_001730.T1, Maole_001757.T1, Maole_001859.T1, Maole_0
.T1, Maole_005013.T1, Maole_005326.T1, Maole_005388.T1, Maole_005478.T1, Maole_0
.T1, Maole_005818.T1, Maole_005898.T1, Maole_006040.T1, Maole_006561.T1, Maole_0
.T1, Maole_005622.T1, Maole_005818.T1, Maole_005898.T1, Maole_006040.T1, Maole_0
.T1, Maole_005898.T1, Maole_006040.T1, Maole_006561.T1, Maole_006647.T1, Maole_0
.T1, Maole_005898.T1, Maole_006040.T1, Maole_006561.T1, Maole_006647.T1, Maole_0
.T1, Maole_005622.T1, Maole_005818.T1, Maole_005898.T1, Maole_006040.T1, Maole_0
.T1, Maole_002652.T1, Maole_002680.T1, Maole_002750.T1, Maole_002778.T1, Maole_0
.T1, Maole_004600.T1, Maole_004602.T1, Maole_004878.T1, Maole_004972.T1, Maole_0
.T1, Maole_011872.T1, Maole_012061.T1, Maole_012114.T1, Maole_012116.T1, Maole_0
.T1, Maole_016363.T1, Maole_017094.T1, Maole_018026.T1, Maole_018590.T1, Maole_0
.T1, Maole_022120.T1, Maole_022202.T1, Maole_022732.T1, Maole_022733.T1, Maole_0
.T1, Maole_017395.T1, Maole_017660.T1, Maole_017679.T1, Maole_017900.T1, Maole_0
.T1, Maole_006194.T1, Maole_006351.T1, Maole_006468.T1, Maole_006581.T1, Maole_0
.T1, Maole_007108.T1, Maole_007145.T1, Maole_007294.T1, Maole_007429.T1, Maole_0
.T1, Maole_019416.T1, Maole_019417.T1, Maole_019422.T1, Maole_019476.T1, Maole_0
.T1, Maole_009446.T1, Maole_009754.T1, Maole_009796.T1, Maole_010849.T1, Maole_0
.T1, Maole_021425.T1, Maole_022100.T1, Maole_022732.T1, Maole_022733.T1, Maole_0
.T1, Maole_012144.T1, Maole_012145.T1, Maole_012180.T1, Maole_012285.T1, Maole_0
.T1, Maole_023194.T1, Maole_023612.T1

.T1, Maole_005453.T1, Maole_005483.T1, Maole_005518.T1, Maole_005675.T1, Maole_0
.T1, Maole_016770.T1, Maole_017085.T1, Maole_017086.T1, Maole_017087.T1, Maole_0
.T1, Maole_015835.T1, Maole_015873.T1, Maole_016011.T1, Maole_016612.T1, Maole_0
.T1, Maole_006295.T1, Maole_006819.T1, Maole_006850.T1, Maole_007143.T1, Maole_0
.T1, Maole_012602.T1, Maole_012716.T1, Maole_012805.T1, Maole_013116.T1, Maole_0
.T1, Maole_009754.T1, Maole_010934.T1, Maole_011207.T1, Maole_011523.T1, Maole_0
.T1, Maole_010098.T1, Maole_010110.T1, Maole_010337.T1, Maole_010389.T1, Maole_0

.T1, Maole_015921.T1, Maole_016217.T1, Maole_016245.T1, Maole_016455.T1, Maole_0

.T1, Maole_009268.T1, Maole_009606.T1, Maole_009607.T1, Maole_009608.T1, Maole_0

.T1, Maole_002707.T1, Maole_002742.T1, Maole_002747.T1, Maole_002762.T1, Maole_0

.T1, Maole_018086.T1, Maole_019071.T1, Maole_019580.T1, Maole_020161.T1, Maole_0

.T1, Maole_016457.T1, Maole_016686.T1, Maole_017309.T1, Maole_017673.T1, Maole_0

.T1, Maole_014993.T1, Maole_015671.T1, Maole_015896.T1, Maole_016043.T1, Maole_0

.T1, Maole_020187.T1, Maole_020298.T1, Maole_020517.T1, Maole_020738.T1, Maole_0

.T1, Maole_012158.T1, Maole_012210.T1, Maole_012255.T1, Maole_013301.T1, Maole_0

.T1, Maole_008800.T1, Maole_008874.T1, Maole_008875.T1, Maole_008959.T1, Maole_0

.T1, Maole_021091.T1, Maole_021900.T1, Maole_022187.T1, Maole_023299.T1, Maole_0

.T1, Maole_014506.T1, Maole_014728.T1, Maole_014831.T1, Maole_014832.T1, Maole_0

.T1, Maole_010083.T1, Maole_010768.T1, Maole_011051.T1, Maole_011052.T1, Maole_0

.T1, Maole_018869.T1, Maole_019399.T1, Maole_019453.T1, Maole_020198.T1, Maole_0

2.T1, Maole_016977.T1, Maole_016978.T1, Maole_017094.T1, Maole_017220.T1, Maole_01

7.T1, Maole_020238.T1, Maole_021441.T1, Maole_023802.T1, Maole_024121.T1

0.T1, Maole_010601.T1, Maole_011059.T1, Maole_011211.T1, Maole_011264.T1, Maole_01

2.T1, Maole_021844.T1, Maole_022244.T1, Maole_022842.T1, Maole_022844.T1, Maole_02

1.T1, Maole_016542.T1, Maole_016855.T1, Maole_017163.T1, Maole_017383.T1, Maole_01

..T1, Maole_022779.T1, Maole_023248.T1

).T1, Maole_017900.T1, Maole_017929.T1, Maole_019320.T1, Maole_019423.T1, Maole_0[illegible]

).T1, Maole_020612.T1, Maole_020860.T1, Maole_021407.T1, Maole_021422.T1, Maole_0[illegible]

[illegible].T1, Maole_023728.T1

[illegible].T1, Maole_019929.T1, Maole_020198.T1, Maole_020436.T1, Maole_020438.T1, Maole_[illegible]

[illegible].T1, Maole_003525.T1, Maole_003596.T1, Maole_003597.T1, Maole_003652.T1, Maole_[illegible]

.T1, Maole_012144.T1, Maole_012145.T1, Maole_012649.T1, Maole_013127.T1, Maole_01

).T1, Maole_023931.T1, Maole_024025.T1

[illegible].T1, Maole_016226.T1, Maole_016253.T1, Maole_016473.T1, Maole_016696.T1, Maole_0[illegible]

[illegible].T1, Maole_010401.T1, Maole_010614.T1, Maole_010733.T1, Maole_011519.T1, Maole_0[illegible]

[illegible].T1, Maole_019320.T1, Maole_019399.T1, Maole_019764.T1, Maole_019947.T1, Maole_0[illegible]

[illegible].T1, Maole_011411.T1, Maole_011519.T1, Maole_011885.T1, Maole_011953.T1, Maole_0[illegible]

01432.T1, Maole_001467.T1, Maole_001469.T1, Maol
01420.T1, Maole_001424.T1, Maole_001432.T1, Maol
02505.T1, Maole_002510.T1, Maole_002591.T1, Maol
04246.T1, Maole_004406.T1, Maole_004432.T1, Maol
01878.T1, Maole_001879.T1, Maole_001931.T1, Maol
05492.T1, Maole_005520.T1, Maole_005622.T1, Maol
06647.T1, Maole_006651.T1, Maole_006652.T1, Maol
06561.T1, Maole_006647.T1, Maole_006651.T1, Maol
06651.T1, Maole_006652.T1, Maole_006670.T1, Maol
06651.T1, Maole_006652.T1, Maole_006670.T1, Maol
06561.T1, Maole_006647.T1, Maole_006651.T1, Maol
02782.T1, Maole_002855.T1, Maole_002884.T1, Maol
05013.T1, Maole_005326.T1, Maole_005388.T1, Maol
12845.T1, Maole_013673.T1, Maole_013830.T1, Maol
18592.T1, Maole_018595.T1, Maole_018597.T1, Maol
22737.T1, Maole_022760.T1, Maole_023851.T1
17922.T1, Maole_018086.T1, Maole_018590.T1, Maol
06583.T1, Maole_006713.T1, Maole_006829.T1, Maol
07687.T1, Maole_007690.T1, Maole_007944.T1, Maol
20715.T1, Maole_022215.T1, Maole_022216.T1, Maol
10934.T1, Maole_011930.T1, Maole_012088.T1, Maol
22737.T1, Maole_023419.T1
12342.T1, Maole_012360.T1, Maole_012716.T1, Maol

06085.T1, Maole_006351.T1, Maole_006468.T1, Maol
17092.T1, Maole_017673.T1, Maole_018059.T1, Maol
16977.T1, Maole_016978.T1, Maole_017094.T1, Maol
07145.T1, Maole_007236.T1, Maole_007360.T1, Maol
13119.T1, Maole_013153.T1, Maole_013299.T1, Maol
11813.T1, Maole_011872.T1, Maole_011987.T1, Maol
11377.T1, Maole_011378.T1, Maole_011406.T1, Maol

16662.T1, Maole_017089.T1, Maole_017245.T1, Maol

09738.T1, Maole_010083.T1, Maole_010114.T1, Maol

02818.T1, Maole_003067.T1, Maole_003087.T1, Maol

20499.T1, Maole_020616.T1, Maole_021199.T1, Maol

18563.T1, Maole_018630.T1, Maole_018701.T1, Maol

16461.T1, Maole_016463.T1, Maole_016467.T1, Maol

21905.T1, Maole_022760.T1, Maole_023468.T1, Maol

13408.T1, Maole_013450.T1, Maole_013486.T1, Maol

09067.T1, Maole_009364.T1, Maole_009738.T1, Maol

23479.T1, Maole_023513.T1, Maole_023612.T1

15154.T1, Maole_015464.T1, Maole_015579.T1, Maol

11211.T1, Maole_011709.T1, Maole_011832.T1, Maol

20351.T1, Maole_020616.T1, Maole_020655.T1, Maol

17644.T1, Maole_018026.T1, Maole_018327.T1, Maol

11377.T1, Maole_011378.T1, Maole_013253.T1, Maol

23519.T1, Maole_023523.T1, Maole_024010.T1

17398.T1, Maole_017544.T1, Maole_017911.T1, Maol

20103.T1, Maole_021285.T1, Maole_021993.T1, Maol

22350.T1, Maole_022352.T1, Maole_022678.T1, Maol

20565.T1, Maole_020616.T1, Maole_020830.T1, Maol

03656.T1, Maole_003754.T1, Maole_003773.T1, Maol

13971.T1, Maole_014828.T1, Maole_014838.T1, Maol

17163.T1, Maole_017249.T1, Maole_017378.T1, Maol

12285.T1, Maole_012342.T1, Maole_012496.T1, Maol

20161.T1, Maole_020797.T1, Maole_020807.T1, Maol

12257.T1, Maole_012375.T1, Maole_012448.T1, Maol

 Click here to download Table Table S18.xlsx

Category	P_value	Q_value	numDEInCat	numInCat
ko04626	6.98E-09	9.14E-07	79	236
ko00520	6.12E-07	4.01E-05	48	124
ko00565	6.45E-06	0.000226438	16	26
ko04075	6.91E-06	0.000226438	75	264
ko00052	1.78E-05	0.000466397	27	62
ko00591	3.88E-05	0.000847414	9	11
ko00562	0.000141543	0.002648878	23	53
ko00500	0.00035492	0.005811815	72	251
ko00062	0.000603499	0.008784269	15	36
ko02010	0.001184455	0.015467687	15	33
ko04145	0.001298813	0.015467687	26	74

**Term**	**Class**
Plant-pathogen interaction	Organismal Systems; E
Amino sugar and nucleotide sugar metabolism	Metabolism; Carbohydr
Ether lipid metabolism	Metabolism; Lipid meta
Plant hormone signal transduction	Environmental Informa
Galactose metabolism	Metabolism; Carbohydr
Linoleic acid metabolism	Metabolism; Lipid meta
Inositol phosphate metabolism	Metabolism; Carbohydr
Starch and sucrose metabolism	Metabolism; Carbohydr
Fatty acid elongation	Metabolism; Lipid meta
ABC transporters	Environmental Informa
Phagosome	Cellular Processes; Tra

**Gene ID**
Maole_000905.T1, Maole_001631.T1, Maole_001879.T1, Maole_002777.T1, Maole_002782.T1, Maole_00
Maole_000672.T1, Maole_000921.T1, Maole_000922.T1, Maole_001037.T1, Maole_002029.T1, Maole_00
Maole_001011.T1, Maole_001100.T1, Maole_001516.T1, Maole_001616.T1, Maole_004385.T1, Maole_00
Maole_000473.T1, Maole_000724.T1, Maole_001025.T1, Maole_001163.T1, Maole_001631.T1, Maole_00
Maole_001037.T1, Maole_001123.T1, Maole_001124.T1, Maole_001125.T1, Maole_001240.T1, Maole_00
Maole_007386.T1, Maole_007927.T1, Maole_010760.T1, Maole_010762.T1, Maole_010763.T1, Maole_01
Maole_000738.T1, Maole_001060.T1, Maole_001488.T1, Maole_001516.T1, Maole_001649.T1, Maole_00
Maole_000612.T1, Maole_000619.T1, Maole_000922.T1, Maole_001037.T1, Maole_001123.T1, Maole_00
Maole_002411.T1, Maole_003085.T1, Maole_004215.T1, Maole_005654.T1, Maole_005846.T1, Maole_00
Maole_001545.T1, Maole_004980.T1, Maole_004982.T1, Maole_011110.T1, Maole_011111.T1, Maole_01
Maole_001488.T1, Maole_002627.T1, Maole_002924.T1, Maole_003356.T1, Maole_003562.T1, Maole_00

3326.T1, Maole_003625.T1, Maole_004073.T1, Maole_004331.T1, Maole_004972.T1, Mao
2826.T1, Maole_003688.T1, Maole_003773.T1, Maole_005046.T1, Maole_005607.T1, Mao
8809.T1, Maole_009636.T1, Maole_016612.T1, Maole_016666.T1, Maole_016977.T1, Mao
1826.T1, Maole_002381.T1, Maole_002556.T1, Maole_002652.T1, Maole_002693.T1, Mao
2240.T1, Maole_002406.T1, Maole_003227.T1, Maole_005046.T1, Maole_006391.T1, Mao
3630.T1, Maole_017544.T1, Maole_021768.T1, Maole_022398.T1
4385.T1, Maole_005343.T1, Maole_005612.T1, Maole_006126.T1, Maole_006773.T1, Mao
1124.T1, Maole_001125.T1, Maole_001481.T1, Maole_002146.T1, Maole_002235.T1, Mao
8020.T1, Maole_009817.T1, Maole_016461.T1, Maole_016463.T1, Maole_016467.T1, Mao
1159.T1, Maole_011401.T1, Maole_013561.T1, Maole_014744.T1, Maole_016768.T1, Mao
5483.T1, Maole_006351.T1, Maole_006933.T1, Maole_007270.T1, Maole_007272.T1, Mao

le_005102.T1, Maole_005221.T1, Maole_005388.T1, Maole_005996.T1, Maole_006214.T1,
le_005675.T1, Maole_006000.T1, Maole_006066.T1, Maole_007035.T1, Maole_007108.T1,
le_016978.T1, Maole_019503.T1, Maole_020979.T1, Maole_021768.T1, Maole_023496.T1,
le_003496.T1, Maole_003920.T1, Maole_004100.T1, Maole_004101.T1, Maole_004104.T1,
le_007656.T1, Maole_008181.T1, Maole_009181.T1, Maole_011198.T1, Maole_011709.T1,

le_006774.T1, Maole_009740.T1, Maole_010670.T1, Maole_012022.T1, Maole_016666.T1,
le_002236.T1, Maole_002240.T1, Maole_002241.T1, Maole_002406.T1, Maole_003226.T1,
le_016855.T1, Maole_017397.T1, Maole_017398.T1, Maole_018850.T1, Maole_018852.T1
le_017309.T1, Maole_021185.T1, Maole_022100.T1, Maole_022821.T1, Maole_023076.T1
le_008313.T1, Maole_008839.T1, Maole_009100.T1, Maole_010479.T1, Maole_011181.T1,

, Maole_006670.T1, Maole_006779.T1, Maole_006885.T1, Maole_007126.T1, Maole_0074[illegible]
, Maole_007656.T1, Maole_008181.T1, Maole_008447.T1, Maole_008465.T1, Maole_0097[illegible]
, Maole_023771.T1
, Maole_004105.T1, Maole_004111.T1, Maole_004554.T1, Maole_005101.T1, Maole_0056[illegible]
, Maole_015629.T1, Maole_016735.T1, Maole_016933.T1, Maole_017109.T1, Maole_0171[illegible]

, Maole_017719.T1, Maole_019427.T1, Maole_020589.T1, Maole_020979.T1, Maole_0211[illegible]
, Maole_003227.T1, Maole_003315.T1, Maole_003594.T1, Maole_003773.T1, Maole_0041[illegible]

, Maole_011836.T1, Maole_012665.T1, Maole_014746.T1, Maole_016166.T1, Maole_0164[illegible]

[illegible]2.T1, Maole_007723.T1, Maole_008105.T1, Maole_009096.T1, Maole_009303.T1, Maole_[illegible]
[illegible]4.T1, Maole_009796.T1, Maole_011198.T1, Maole_012180.T1, Maole_012353.T1, Maole_[illegible]

[illegible]4.T1, Maole_006040.T1, Maole_006199.T1, Maole_006311.T1, Maole_006356.T1, Maole_[illegible]
[illegible]7.T1, Maole_018437.T1, Maole_018561.T1, Maole_019175.T1, Maole_021014.T1, Maole_[illegible]

[illegible]5.T1, Maole_021993.T1, Maole_022983.T1, Maole_022984.T1
[illegible]2.T1, Maole_004819.T1, Maole_005046.T1, Maole_005625.T1, Maole_005626.T1, Maole_[illegible]

[illegible]7.T1, Maole_017719.T1, Maole_018100.T1, Maole_019070.T1, Maole_019324.T1, Maole_[illegible]

)09317.T1, Maole_009320.T1, Maole_009364.T1, Maole_009509.T1, Maole_009644.T1, Ma
)12360.T1, Maole_012766.T1, Maole_012828.T1, Maole_013339.T1, Maole_013763.T1, Ma

)06559.T1, Maole_006706.T1, Maole_006799.T1, Maole_007230.T1, Maole_007320.T1, Ma
)21015.T1, Maole_021289.T1, Maole_022497.T1

)05675.T1, Maole_005709.T1, Maole_006066.T1, Maole_007035.T1, Maole_007285.T1, Ma

)20238.T1, Maole_021993.T1

aole_009945.T1, Maole_010110.T1, Maole_010308.T1, Maole_011710.T1, Maole_011885.T
aole_014831.T1, Maole_014832.T1, Maole_014867.T1, Maole_015464.T1, Maole_015610.T

aole_007366.T1, Maole_007391.T1, Maole_007964.T1, Maole_009038.T1, Maole_009289.T

aole_008181.T1, Maole_008264.T1, Maole_008465.T1, Maole_008713.T1, Maole_009181.T

T1, Maole_011953.T1, Maole_012158.T1, Maole_012236.T1, Maole_012375.T1, Maole_012
T1, Maole_016082.T1, Maole_016155.T1, Maole_016733.T1, Maole_016735.T1, Maole_016

T1, Maole_009466.T1, Maole_009598.T1, Maole_010021.T1, Maole_010293.T1, Maole_010

T1, Maole_009724.T1, Maole_009754.T1, Maole_009796.T1, Maole_011051.T1, Maole_011

507.T1, Maole_013413.T1, Maole_013571.T1, Maole_013689.T1, Maole_014265.T1, Maole
763.T1, Maole_017197.T1, Maole_018679.T1, Maole_020896.T1, Maole_020940.T1, Maole

480.T1, Maole_011813.T1, Maole_011930.T1, Maole_012210.T1, Maole_012554.T1, Maole

052.T1, Maole_011198.T1, Maole_012180.T1, Maole_012353.T1, Maole_012360.T1, Maole

e_014267.T1, Maole_014269.T1, Maole_014270.T1, Maole_014272.T1, Maole_014274.T1, M
e_021069.T1, Maole_021289.T1, Maole_022034.T1, Maole_022497.T1, Maole_023466.T1

e_012805.T1, Maole_013299.T1, Maole_013870.T1, Maole_013950.T1, Maole_014088.T1, M

e_012531.T1, Maole_012766.T1, Maole_012828.T1, Maole_013339.T1, Maole_014579.T1, M

Maole_014275.T1, Maole_014602.T1, Maole_014641.T1, Maole_015116.T1, Maole_015407

Maole_014948.T1, Maole_015324.T1, Maole_015873.T1, Maole_016104.T1, Maole_01623[illegible]

Maole_014831.T1, Maole_014832.T1, Maole_015610.T1, Maole_016082.T1, Maole_01615[illegible]

[illegible].T1, Maole_015659.T1, Maole_017094.T1, Maole_017166.T1, Maole_017261.T1, Maole_0[illegible]

[illegible].T1, Maole_016971.T1, Maole_017331.T1, Maole_017383.T1, Maole_017713.T1, Maole_0[illegible]

[illegible].T1, Maole_016700.T1, Maole_016733.T1, Maole_016735.T1, Maole_017223.T1, Maole_0[illegible]

17476.T1, Maole_017644.T1, Maole_017653.T1, Maol

17922.T1, Maole_018869.T1, Maole_018886.T1, Maol

18644.T1, Maole_018863.T1, Maole_018864.T1, Maol

Table

Click here to download Table Table S19.xlsx

Category	P_value	Q_value	numDEIr	numInCa	Term	Class	Gene ID
GO:00094	2.33E-52	2.03E-48	39	66	response t	biological_process	Maole_00
GO:00102	2.41E-34	1.05E-30	51	322	response t	biological_process	Maole_00
GO:00068	5.86E-31	1.67E-27	20	26	drug trans	biological_process	Maole_00
GO:00160	7.68E-31	1.67E-27	152	3,575	integral co	cellular_component	Maole_00
GO:00037	5.46E-30	9.49E-27	84	1,185	transcripti	molecular_function	Maole_00
GO:00462	1.35E-29	1.93E-26	18	21	lignin cata	biological_process	Maole_00
GO:00097	1.56E-29	1.93E-26	43	264	auxin-acti	biological_process	Maole_00
GO:00426	2.83E-28	3.07E-25	24	55	ATPase a	molecular_function	Maole_00
GO:00527	3.31E-28	3.19E-25	18	23	hydroquin	molecular_function	Maole_00
GO:00160	4.31E-26	3.75E-23	18	27	detection	biological_process	Maole_00
GO:00098	9.44E-26	7.45E-23	23	59	embryonic	biological_process	Maole_00
GO:00063	3.53E-25	2.56E-22	89	1,552	regulation	biological_process	Maole_00
GO:00085	6.20E-25	4.14E-22	17	25	xenobiotic	molecular_function	Maole_00
GO:00103	4.49E-24	2.79E-21	19	38	regulation	biological_process	Maole_00
GO:00003	7.17E-23	4.16E-20	25	96	plant-type	cellular_component	Maole_00
GO:00097	9.14E-23	4.96E-20	39	307	response t	biological_process	Maole_00
GO:00063	1.12E-21	5.73E-19	89	1,745	transcripti	biological_process	Maole_00
GO:00095	1.27E-20	6.13E-18	85	1,668	plasmodes	cellular_component	Maole_00
GO:00100	4.31E-19	1.97E-16	23	107	xylem and	biological_process	Maole_00
GO:00097	8.28E-19	3.60E-16	47	587	hormone-i	biological_process	Maole_00
GO:00055	1.90E-17	7.87E-15	106	2,706	ATP bind	molecular_function	Maole_00
GO:00016	2.09E-17	8.24E-15	44	558	peptide re	molecular_function	Maole_00
GO:00058	3.08E-17	1.16E-14	113	3,020	plasma me	cellular_component	Maole_00
GO:00483	4.23E-17	1.53E-14	29	230	root devel	biological_process	Maole_00
GO:00046	3.23E-16	1.12E-13	42	548	transmem	molecular_function	Maole_00
GO:00071	3.44E-16	1.15E-13	42	549	transmem	biological_process	Maole_00
GO:00169	1.20E-15	3.86E-13	12	24	cell wall r	biological_process	Maole_02
GO:00480	1.27E-15	3.95E-13	43	597	apoplast	cellular_component	Maole_00
GO:00467	1.57E-15	4.70E-13	48	742	protein au	biological_process	Maole_00
GO:00096	4.71E-15	1.36E-12	11	20	hydrogen-	molecular_function	Maole_00
GO:00096	4.89E-15	1.37E-12	21	128	plant-type	biological_process	Maole_00
GO:00102	1.42E-14	3.85E-12	10	16	establishn	biological_process	Maole_00
GO:00046	1.87E-14	4.91E-12	54	981	protein se	molecular_function	Maole_00
GO:00099	2.35E-14	6.01E-12	17	80	auxin pola	biological_process	Maole_00
GO:00100	2.67E-14	6.62E-12	21	139	endosome	cellular_component	Maole_00
GO:00316	3.32E-14	7.94E-12	42	627	ubiquitin	molecular_function	Maole_00
GO:00068	3.38E-14	7.94E-12	10	17	receptor-m	biological_process	Maole_00
GO:00458	9.09E-14	2.08E-11	39	561	positive re	biological_process	Maole_00
GO:00044	1.15E-13	2.56E-11	11	25	inorganic	molecular_function	Maole_00
GO:00525	9.66E-13	2.10E-10	13	48	defense re	biological_process	Maole_00
GO:00105	1.19E-12	2.52E-10	9	16	acropetal	biological_process	Maole_00
GO:00458	2.49E-12	5.14E-10	9	17	pH reduct	biological_process	Maole_00
GO:00027	3.23E-12	6.52E-10	8	12	immune re	biological_process	Maole_00
GO:00045	4.95E-12	9.35E-10	12	43	chitinase a	molecular_function	Maole_02
GO:00060	4.95E-12	9.35E-10	12	43	chitin cata	biological_process	Maole_02
GO:00080	4.95E-12	9.35E-10	12	43	chitin bin	molecular_function	Maole_02
GO:00080	3.99E-11	6.93E-09	8	15	glutamate	molecular_function	Maole_00

GO:00315	3.99E-11	6.93E-09	8	15	motile cili	cellular_component	Maole_00
GO:00339	3.99E-11	6.93E-09	8	15	xylogluca	molecular_function	Maole_00
GO:00712	3.99E-11	6.93E-09	8	15	cellular re	biological_process	Maole_00
GO:00458	6.18E-11	1.05E-08	23	251	negative r	biological_process	Maole_00
GO:00048	7.55E-11	1.26E-08	9	23	transmem	molecular_function	Maole_00
GO:00057	2.01E-10	3.29E-08	40	757	vacuole	cellular_component	Maole_00
GO:00046	2.16E-10	3.48E-08	45	928	protein ki	molecular_function	Maole_00
GO:00103	2.83E-10	4.46E-08	9	26	auxin effl	molecular_function	Maole_00
GO:00002	3.61E-10	5.60E-08	12	60	polysacch	biological_process	Maole_02
GO:00055	1.44E-09	2.14E-07	19	197	copper ion	molecular_function	Maole_00
GO:00102	1.45E-09	2.14E-07	5	5	chlorophy	molecular_function	Maole_00
GO:00713	1.45E-09	2.14E-07	5	5	cellular re	biological_process	Maole_00
GO:00049	1.81E-09	2.62E-07	8	22	ionotropic	molecular_function	Maole_00
GO:00095	4.20E-09	5.89E-07	9	34	photosyst	cellular_component	Maole_00
GO:00103	4.20E-09	5.89E-07	9	34	auxin effl	biological_process	Maole_00
GO:00052	5.85E-09	8.07E-07	8	25	calcium cl	molecular_function	Maole_00
GO:00102	8.32E-09	1.11E-06	8	26	defense re	biological_process	Maole_00
GO:00154	8.60E-09	1.11E-06	5	6	glutathion	molecular_function	Maole_00
GO:00156	8.60E-09	1.11E-06	5	6	lead ion tr	biological_process	Maole_01
GO:00468	8.60E-09	1.11E-06	5	6	terpenoid	biological_process	Maole_01
GO:00060	9.53E-09	1.18E-06	9	37	cellular gl	biological_process	Maole_00
GO:00167	9.53E-09	1.18E-06	9	37	xylogluca	molecular_function	Maole_00
GO:00430	9.53E-09	1.18E-06	9	37	polar nucl	cellular_component	Maole_01
GO:00427	1.58E-08	1.94E-06	26	416	defense re	biological_process	Maole_00
GO:00103	2.65E-08	3.20E-06	7	20	regulation	biological_process	Maole_00
GO:00105	3.17E-08	3.77E-06	9	42	basipetal a	biological_process	Maole_00
GO:00043	3.81E-08	4.42E-06	6	13	guanylate	molecular_function	Maole_00
GO:00061	3.81E-08	4.42E-06	6	13	cGMP bio	biological_process	Maole_00
GO:00705	6.66E-08	7.62E-06	8	33	calcium io	biological_process	Maole_00
GO:00191	7.37E-08	8.21E-06	9	46	transmem	molecular_function	Maole_00
GO:00336	7.37E-08	8.21E-06	9	46	receptor s	molecular_function	Maole_00
GO:00158	8.56E-08	9.30E-06	4	4	organic hy	biological_process	Maole_00
GO:19011	8.56E-08	9.30E-06	4	4	p-coumary	biological_process	Maole_00
GO:00161	1.89E-07	2.02E-05	9	51	chlorophy	molecular_function	Maole_00
GO:00098	2.05E-07	2.16E-05	12	103	lignin bios	biological_process	Maole_00
GO:00096	2.06E-07	2.16E-05	7	26	response t	biological_process	Maole_00
GO:00704	2.18E-07	2.25E-05	8	38	cellular re	biological_process	Maole_00
GO:00159	3.61E-07	3.69E-05	11	89	proton tra	biological_process	Maole_00
GO:00429	6.17E-07	5.95E-05	5	11	dipeptide	molecular_function	Maole_00
GO:00429	6.17E-07	5.95E-05	5	11	tripeptide	molecular_function	Maole_00
GO:00429	6.17E-07	5.95E-05	5	11	dipeptide	biological_process	Maole_00
GO:00429	6.17E-07	5.95E-05	5	11	tripeptide	biological_process	Maole_00
GO:00801	6.17E-07	5.95E-05	5	11	abscisic a	biological_process	Maole_01
GO:00097	7.71E-07	7.34E-05	13	138	plant-type	cellular_component	Maole_00
GO:00309	7.78E-07	7.34E-05	6	20	potassium	molecular_function	Maole_00
GO:00466	1.07E-06	0.0001	6	21	response t	biological_process	Maole_00
GO:00057	1.15E-06	0.000106	30	655	vacuolar r	cellular_component	Maole_00
GO:00101	1.50E-06	0.000137	7	34	pollen ma	biological_process	Maole_00

GO:00508	2.22E-06	0.000201	20	343	defense re	biological_process	Maole_00
GO:00197	2.34E-06	0.000209	8	51	calcium-n	biological_process	Maole_00
GO:00182	2.37E-06	0.00021	9	68	protein-ch	biological_process	Maole_00
GO:00096	2.74E-06	0.000241	7	37	phenylpro	biological_process	Maole_00
GO:00002	2.77E-06	0.000241	15	205	magnesiu	molecular_function	Maole_00
GO:19011	2.88E-06	0.000247	4	7	regulation	biological_process	Maole_00
GO:00068	3.95E-06	0.000336	15	211	transport	biological_process	Maole_00
GO:00095	5.43E-06	0.000458	9	75	microspor	biological_process	Maole_00
GO:00515	6.06E-06	0.000506	9	76	4 iron, 4 s	molecular_function	Maole_00
GO:00098	6.73E-06	0.000557	6	28	induced sy	biological_process	Maole_00
GO:00164	1.07E-05	0.000874	22	447	oxidoredu	molecular_function	Maole_00
GO:00056	1.46E-05	0.001187	31	783	cell wall	cellular_component	Maole_00
GO:00082	1.99E-05	0.001568	3	4	sulfonylu	molecular_function	Maole_00
GO:00506	1.99E-05	0.001568	3	4	oxidoredu	molecular_function	Maole_00
GO:00423	1.99E-05	0.001568	3	4	indole glu	biological_process	Maole_00
GO:00160	2.23E-05	0.001742	58	1,950	membrane	cellular_component	Maole_00
GO:00550	2.48E-05	0.001923	15	246	transmem	biological_process	Maole_00
GO:00103	2.57E-05	0.001957	4	11	auxin infl	molecular_function	Maole_00
GO:00609	2.57E-05	0.001957	4	11	auxin infl	biological_process	Maole_00
GO:00068	3.55E-05	0.002684	8	73	calcium io	biological_process	Maole_00
GO:00151	4.90E-05	0.003626	3	5	peptide tr	molecular_function	Maole_00
GO:00153	4.90E-05	0.003626	3	5	high-affin	molecular_function	Maole_00
GO:00168	4.92E-05	0.003626	14	231	ATPase ac	molecular_function	Maole_00
GO:00103	5.24E-05	0.003826	8	77	regulation	biological_process	Maole_00
GO:00485	5.82E-05	0.004212	5	25	meristem	biological_process	Maole_02
GO:00125	6.31E-05	0.004528	8	79	endomem	cellular_component	Maole_00
GO:00168	7.48E-05	0.005325	4	14	intramole	molecular_function	Maole_00
GO:00068	7.66E-05	0.005381	6	42	sodium io	biological_process	Maole_00
GO:00098	7.68E-05	0.005381	9	104	plant-type	biological_process	Maole_00
GO:00161	9.68E-05	0.006619	3	6	NAD(P)H	molecular_function	Maole_00
GO:19023	9.68E-05	0.006619	3	6	11-oxo-be	biological_process	Maole_01
GO:19023	9.68E-05	0.006619	3	6	glycyrrhet	biological_process	Maole_01
GO:00047	9.78E-05	0.006641	8	84	transmem	molecular_function	Maole_00
GO:00096	0.0001	0.006741	6	44	cold accli	biological_process	Maole_00
GO:00096	0.000111	0.007336	9	109	systemic a	biological_process	Maole_00
GO:00102	0.000111	0.007336	7	64	floral orga	biological_process	Maole_00
GO:00094	0.000111	0.007336	19	415	response t	biological_process	Maole_00
GO:00197	0.000132	0.008578	4	16	pentacycli	biological_process	Maole_00
GO:00062	0.000132	0.008578	4	16	DNA meta	biological_process	Maole_00
GO:00096	0.000156	0.01004	9	114	plant-type	biological_process	Maole_00
GO:00096	0.000164	0.010494	6	48	induced sy	biological_process	Maole_00
GO:00154	0.000167	0.010601	3	7	organic ph	molecular_function	Maole_00
GO:00159	0.000178	0.011212	9	116	photosynt	biological_process	Maole_00
GO:00483	0.000187	0.011705	11	171	leaf devel	biological_process	Maole_00
GO:00096	0.000215	0.013369	8	94	gravitropi	biological_process	Maole_00
GO:00096	0.000251	0.015442	25	670	response t	biological_process	Maole_00
GO:00152	0.000294	0.01728	2	2	ligand-gat	molecular_function	Maole_00
GO:00422	0.000294	0.01728	2	2	lupeol syn	molecular_function	Maole_01

GO:00713	0.000294	0.01728	2	2	cellular re	biological_process	Maole_00
GO:00040	0.000294	0.01728	2	2	adenylate	molecular_function	Maole_02
GO:00061	0.000294	0.01728	2	2	cAMP bic	biological_process	Maole_02
GO:19024	0.000294	0.01728	2	2	(+)-abscis	molecular_function	Maole_01
GO:19024	0.000294	0.01728	2	2	(+)-abscis	biological_process	Maole_01
GO:00069	0.000299	0.017458	25	678	defense re	biological_process	Maole_00
GO:00150	0.000391	0.022352	3	9	cadmium	molecular_function	Maole_00
GO:00705	0.000391	0.022352	3	9	cadmium	biological_process	Maole_00
GO:00049	0.000391	0.022352	3	9	G-protein	molecular_function	Maole_00
GO:00052	0.000481	0.027307	12	222	transporte	molecular_function	Maole_00
GO:00301	0.000486	0.027403	7	81	protein ca	biological_process	Maole_00
GO:00090	0.000645	0.036136	9	138	electron c	molecular_function	Maole_00
GO:00094	0.000776	0.04319	18	447	response t	biological_process	Maole_00
GO:00161	0.000873	0.045776	2	3	triterpeno	biological_process	Maole_00
GO:00432	0.000873	0.045776	2	3	response t	biological_process	Maole_00
GO:00800	0.000873	0.045776	2	3	response t	biological_process	Maole_00
GO:00800	0.000873	0.045776	2	3	response t	biological_process	Maole_00
GO:00429	0.000873	0.045776	2	3	xenobiotic	biological_process	Maole_00
GO:00469	0.000873	0.045776	2	3	tetrahydrc	biological_process	Maole_01
GO:00719	0.000873	0.045776	2	3	phytochel	molecular_function	Maole_01
GO:00719	0.000873	0.045776	2	3	phytochel	biological_process	Maole_01
GO:00423	0.000873	0.045776	2	3	beta-amyr	molecular_function	Maole_00
GO:00450	0.000875	0.045776	9	144	innate imı	biological_process	Maole_00

5120.T1, Maole_005178.T1, Maole_005179.T1, Maole_005180.T1, Maole_005694.T1, Maole_
0378.T1, Maole_002027.T1, Maole_002707.T1, Maole_003161.T1, Maole_005120.T1, Maole_
1019.T1, Maole_001020.T1, Maole_001021.T1, Maole_001022.T1, Maole_001931.T1, Maole_
0212.T1, Maole_000390.T1, Maole_001019.T1, Maole_001020.T1, Maole_001021.T1, Maole_
0378.T1, Maole_000437.T1, Maole_000677.T1, Maole_001434.T1, Maole_001560.T1, Maole_
2588.T1, Maole_002636.T1, Maole_002645.T1, Maole_002648.T1, Maole_003245.T1, Maole_
0378.T1, Maole_000437.T1, Maole_000677.T1, Maole_001434.T1, Maole_001538.T1, Maole_
0390.T1, Maole_001096.T1, Maole_001545.T1, Maole_001931.T1, Maole_004980.T1, Maole_
2588.T1, Maole_002636.T1, Maole_002645.T1, Maole_002648.T1, Maole_003245.T1, Maole_
4972.T1, Maole_006779.T1, Maole_009317.T1, Maole_009320.T1, Maole_014265.T1, Maole_
0378.T1, Maole_000437.T1, Maole_000677.T1, Maole_001434.T1, Maole_002027.T1, Maole_
0378.T1, Maole_000437.T1, Maole_000677.T1, Maole_001434.T1, Maole_001560.T1, Maole_
0390.T1, Maole_001096.T1, Maole_001545.T1, Maole_001931.T1, Maole_004982.T1, Maole_
4972.T1, Maole_006779.T1, Maole_009317.T1, Maole_009320.T1, Maole_014265.T1, Maole_
0390.T1, Maole_001096.T1, Maole_001931.T1, Maole_002591.T1, Maole_003069.T1, Maole_
0378.T1, Maole_000437.T1, Maole_000677.T1, Maole_001434.T1, Maole_001560.T1, Maole_
0378.T1, Maole_000437.T1, Maole_000677.T1, Maole_001434.T1, Maole_001560.T1, Maole_
1375.T1, Maole_001461.T1, Maole_001545.T1, Maole_001931.T1, Maole_002245.T1, Maole_
0378.T1, Maole_000437.T1, Maole_000677.T1, Maole_001434.T1, Maole_002027.T1, Maole_
1375.T1, Maole_001461.T1, Maole_004639.T1, Maole_004972.T1, Maole_005013.T1, Maole_
0390.T1, Maole_001019.T1, Maole_001020.T1, Maole_001021.T1, Maole_001022.T1, Maole_
1375.T1, Maole_001461.T1, Maole_004639.T1, Maole_004972.T1, Maole_005013.T1, Maole_
1019.T1, Maole_001020.T1, Maole_001021.T1, Maole_001022.T1, Maole_001375.T1, Maole_
0378.T1, Maole_000437.T1, Maole_000677.T1, Maole_001434.T1, Maole_002027.T1, Maole_
1375.T1, Maole_001461.T1, Maole_004639.T1, Maole_004972.T1, Maole_005013.T1, Maole_
1375.T1, Maole_001461.T1, Maole_004639.T1, Maole_004972.T1, Maole_005013.T1, Maole_
0579.T1, Maole_023238.T1, Maole_023239.T1, Maole_023241.T1, Maole_023344.T1, Maole_
2588.T1, Maole_002636.T1, Maole_002645.T1, Maole_002648.T1, Maole_002978.T1, Maole_
1375.T1, Maole_001461.T1, Maole_004639.T1, Maole_004972.T1, Maole_005013.T1, Maole_
3069.T1, Maole_003305.T1, Maole_003945.T1, Maole_006532.T1, Maole_008212.T1, Maole_
2245.T1, Maole_002247.T1, Maole_002253.T1, Maole_003197.T1, Maole_006779.T1, Maole_
3069.T1, Maole_003305.T1, Maole_003945.T1, Maole_006532.T1, Maole_008212.T1, Maole_
1375.T1, Maole_001461.T1, Maole_004639.T1, Maole_004972.T1, Maole_005013.T1, Maole_
3069.T1, Maole_003305.T1, Maole_003945.T1, Maole_004982.T1, Maole_006532.T1, Maole_
3069.T1, Maole_003305.T1, Maole_003945.T1, Maole_004972.T1, Maole_006532.T1, Maole_
1375.T1, Maole_001461.T1, Maole_004639.T1, Maole_004972.T1, Maole_005013.T1, Maole_
4972.T1, Maole_009317.T1, Maole_009320.T1, Maole_014265.T1, Maole_014267.T1, Maole_
5120.T1, Maole_005178.T1, Maole_005179.T1, Maole_005180.T1, Maole_005694.T1, Maole_
3069.T1, Maole_003305.T1, Maole_003945.T1, Maole_006532.T1, Maole_008212.T1, Maole_
1020.T1, Maole_001021.T1, Maole_001022.T1, Maole_004972.T1, Maole_009317.T1, Maole_
4980.T1, Maole_004982.T1, Maole_011110.T1, Maole_011111.T1, Maole_011159.T1, Maole_
1461.T1, Maole_004639.T1, Maole_015166.T1, Maole_022500.T1, Maole_022504.T1, Maole_
6779.T1, Maole_015116.T1, Maole_021416.T1, Maole_023259.T1, Maole_023260.T1, Maole_
0579.T1, Maole_023238.T1, Maole_023239.T1, Maole_023241.T1, Maole_023344.T1, Maole_
0579.T1, Maole_023238.T1, Maole_023239.T1, Maole_023241.T1, Maole_023344.T1, Maole_
0579.T1, Maole_023238.T1, Maole_023239.T1, Maole_023241.T1, Maole_023344.T1, Maole_
1424.T1, Maole_005877.T1, Maole_005880.T1, Maole_007827.T1, Maole_012162.T1, Maole_

4972.T1, Maole_014265.T1, Maole_014267.T1, Maole_014269.T1, Maole_014270.T1, Maole_
2978.T1, Maole_003782.T1, Maole_003783.T1, Maole_003786.T1, Maole_003790.T1, Maole_
1424.T1, Maole_005877.T1, Maole_005880.T1, Maole_007827.T1, Maole_012162.T1, Maole_
0378.T1, Maole_000437.T1, Maole_000677.T1, Maole_001434.T1, Maole_002027.T1, Maole_
1461.T1, Maole_004639.T1, Maole_015166.T1, Maole_022500.T1, Maole_022504.T1, Maole_
0390.T1, Maole_001096.T1, Maole_001931.T1, Maole_002591.T1, Maole_003069.T1, Maole_
1461.T1, Maole_004639.T1, Maole_004972.T1, Maole_005013.T1, Maole_006647.T1, Maole_
4980.T1, Maole_004982.T1, Maole_011110.T1, Maole_011111.T1, Maole_011159.T1, Maole_
0579.T1, Maole_023238.T1, Maole_023239.T1, Maole_023241.T1, Maole_023344.T1, Maole_
2588.T1, Maole_002636.T1, Maole_002645.T1, Maole_002648.T1, Maole_003245.T1, Maole_
7944.T1, Maole_013780.T1, Maole_020501.T1, Maole_020502.T1, Maole_022100.T1
1020.T1, Maole_001021.T1, Maole_001022.T1, Maole_011288.T1, Maole_017673.T1
1424.T1, Maole_005877.T1, Maole_005880.T1, Maole_007827.T1, Maole_012162.T1, Maole_
0212.T1, Maole_015203.T1, Maole_021341.T1, Maole_021342.T1, Maole_022379.T1, Maole_
4980.T1, Maole_004982.T1, Maole_011110.T1, Maole_011111.T1, Maole_011159.T1, Maole_
1424.T1, Maole_005877.T1, Maole_005880.T1, Maole_007827.T1, Maole_012162.T1, Maole_
6779.T1, Maole_015116.T1, Maole_021416.T1, Maole_023259.T1, Maole_023260.T1, Maole_
7944.T1, Maole_013780.T1, Maole_020501.T1, Maole_020502.T1, Maole_022100.T1
2520.T1, Maole_012595.T1, Maole_012596.T1, Maole_012598.T1, Maole_016455.T1
2520.T1, Maole_012595.T1, Maole_012596.T1, Maole_012598.T1, Maole_016455.T1
2978.T1, Maole_003782.T1, Maole_003783.T1, Maole_003786.T1, Maole_003790.T1, Maole_
2978.T1, Maole_003782.T1, Maole_003783.T1, Maole_003786.T1, Maole_003790.T1, Maole_
1635.T1, Maole_013907.T1, Maole_013908.T1, Maole_013909.T1, Maole_013911.T1, Maole_
1020.T1, Maole_001021.T1, Maole_001022.T1, Maole_001986.T1, Maole_001987.T1, Maole_
2999.T1, Maole_003050.T1, Maole_012089.T1, Maole_013992.T1, Maole_013993.T1, Maole_
4980.T1, Maole_004982.T1, Maole_011110.T1, Maole_011111.T1, Maole_011159.T1, Maole_
1461.T1, Maole_004639.T1, Maole_006651.T1, Maole_006652.T1, Maole_021063.T1, Maole_
1461.T1, Maole_004639.T1, Maole_006651.T1, Maole_006652.T1, Maole_021063.T1, Maole_
1424.T1, Maole_005877.T1, Maole_005880.T1, Maole_007827.T1, Maole_012162.T1, Maole_
6779.T1, Maole_009317.T1, Maole_015116.T1, Maole_021416.T1, Maole_023259.T1, Maole_
9320.T1, Maole_010336.T1, Maole_015116.T1, Maole_022503.T1, Maole_023259.T1, Maole_
1019.T1, Maole_001020.T1, Maole_001021.T1, Maole_001022.T1
1019.T1, Maole_001020.T1, Maole_001021.T1, Maole_001022.T1
0212.T1, Maole_015203.T1, Maole_021341.T1, Maole_021342.T1, Maole_022379.T1, Maole_
2588.T1, Maole_003245.T1, Maole_010114.T1, Maole_010115.T1, Maole_010254.T1, Maole_
2978.T1, Maole_003786.T1, Maole_003790.T1, Maole_003791.T1, Maole_003793.T1, Maole_
1424.T1, Maole_002245.T1, Maole_002247.T1, Maole_002253.T1, Maole_003197.T1, Maole_
3069.T1, Maole_003305.T1, Maole_003945.T1, Maole_006532.T1, Maole_008212.T1, Maole_
1986.T1, Maole_001987.T1, Maole_002591.T1, Maole_006581.T1, Maole_006583.T1
1986.T1, Maole_001987.T1, Maole_002591.T1, Maole_006581.T1, Maole_006583.T1
1986.T1, Maole_001987.T1, Maole_002591.T1, Maole_006581.T1, Maole_006583.T1
1986.T1, Maole_001987.T1, Maole_002591.T1, Maole_006581.T1, Maole_006583.T1
2520.T1, Maole_012595.T1, Maole_012596.T1, Maole_012598.T1, Maole_016455.T1
2591.T1, Maole_003069.T1, Maole_003305.T1, Maole_003945.T1, Maole_006532.T1, Maole_
3069.T1, Maole_003305.T1, Maole_003945.T1, Maole_008212.T1, Maole_021175.T1, Maole_
2636.T1, Maole_002648.T1, Maole_004626.T1, Maole_006703.T1, Maole_010114.T1, Maole_
0390.T1, Maole_001020.T1, Maole_001021.T1, Maole_001022.T1, Maole_001096.T1, Maole_
9320.T1, Maole_010336.T1, Maole_015116.T1, Maole_023259.T1, Maole_023260.T1, Maole_

1424.T1, Maole_002245.T1, Maole_002247.T1, Maole_002248.T1, Maole_002249.T1, Maole_
1424.T1, Maole_005877.T1, Maole_005880.T1, Maole_007827.T1, Maole_012162.T1, Maole_
0212.T1, Maole_015203.T1, Maole_021341.T1, Maole_021342.T1, Maole_022379.T1, Maole_
1538.T1, Maole_009465.T1, Maole_010356.T1, Maole_010359.T1, Maole_010362.T1, Maole_
0212.T1, Maole_003069.T1, Maole_003305.T1, Maole_003945.T1, Maole_008212.T1, Maole_
1019.T1, Maole_001020.T1, Maole_001021.T1, Maole_001022.T1
1019.T1, Maole_001020.T1, Maole_001021.T1, Maole_001022.T1, Maole_004881.T1, Maole_
9317.T1, Maole_009320.T1, Maole_010336.T1, Maole_015116.T1, Maole_023259.T1, Maole_
0212.T1, Maole_015203.T1, Maole_021341.T1, Maole_021342.T1, Maole_022379.T1, Maole_
1424.T1, Maole_007827.T1, Maole_012162.T1, Maole_014199.T1, Maole_016541.T1, Maole_
0212.T1, Maole_002588.T1, Maole_002645.T1, Maole_002648.T1, Maole_003245.T1, Maole_
2245.T1, Maole_002247.T1, Maole_002248.T1, Maole_002249.T1, Maole_002250.T1, Maole_
0390.T1, Maole_007944.T1, Maole_013780.T1
6214.T1, Maole_017644.T1, Maole_019939.T1
1020.T1, Maole_001021.T1, Maole_001022.T1
0182.T1, Maole_001019.T1, Maole_001020.T1, Maole_001021.T1, Maole_001022.T1, Maole_
0390.T1, Maole_004980.T1, Maole_004982.T1, Maole_011110.T1, Maole_011111.T1, Maole_
4982.T1, Maole_013561.T1, Maole_014744.T1, Maole_017309.T1
4982.T1, Maole_013561.T1, Maole_014744.T1, Maole_017309.T1
1424.T1, Maole_005877.T1, Maole_005880.T1, Maole_007827.T1, Maole_012162.T1, Maole_
2591.T1, Maole_006581.T1, Maole_006583.T1
2591.T1, Maole_006581.T1, Maole_006583.T1
1019.T1, Maole_001020.T1, Maole_001021.T1, Maole_001022.T1, Maole_004881.T1, Maole_
2707.T1, Maole_002999.T1, Maole_003050.T1, Maole_012089.T1, Maole_013992.T1, Maole_
3259.T1, Maole_023260.T1, Maole_023776.T1, Maole_024056.T1, Maole_024121.T1
6779.T1, Maole_015116.T1, Maole_021416.T1, Maole_023259.T1, Maole_023260.T1, Maole_
2953.T1, Maole_016874.T1, Maole_016875.T1, Maole_016881.T1
3069.T1, Maole_003305.T1, Maole_003945.T1, Maole_008212.T1, Maole_021175.T1, Maole_
2978.T1, Maole_003782.T1, Maole_003783.T1, Maole_003786.T1, Maole_003790.T1, Maole_
6214.T1, Maole_017644.T1, Maole_019939.T1
0943.T1, Maole_010944.T1, Maole_010945.T1
0943.T1, Maole_010944.T1, Maole_010945.T1
9320.T1, Maole_010336.T1, Maole_022500.T1, Maole_022503.T1, Maole_023259.T1, Maole_
2245.T1, Maole_002247.T1, Maole_002253.T1, Maole_003197.T1, Maole_015936.T1, Maole_
1020.T1, Maole_001021.T1, Maole_001022.T1, Maole_002245.T1, Maole_002247.T1, Maole_
9320.T1, Maole_010336.T1, Maole_022500.T1, Maole_023259.T1, Maole_023260.T1, Maole_
1931.T1, Maole_002588.T1, Maole_003069.T1, Maole_003305.T1, Maole_003945.T1, Maole_
2953.T1, Maole_016874.T1, Maole_016875.T1, Maole_016881.T1
6224.T1, Maole_006226.T1, Maole_006227.T1, Maole_006262.T1
2978.T1, Maole_003786.T1, Maole_003790.T1, Maole_003791.T1, Maole_003793.T1, Maole_
2245.T1, Maole_002247.T1, Maole_002253.T1, Maole_003197.T1, Maole_015936.T1, Maole_
1020.T1, Maole_012596.T1, Maole_012598.T1
0212.T1, Maole_015203.T1, Maole_021341.T1, Maole_021342.T1, Maole_022379.T1, Maole_
3069.T1, Maole_003305.T1, Maole_003945.T1, Maole_006532.T1, Maole_008212.T1, Maole_
1424.T1, Maole_004982.T1, Maole_007827.T1, Maole_012162.T1, Maole_013561.T1, Maole_
0390.T1, Maole_002245.T1, Maole_002247.T1, Maole_002248.T1, Maole_002249.T1, Maole_
1424.T1, Maole_014199.T1
6874.T1, Maole_016875.T1

1424.T1, Maole_014199.T1
1367.T1, Maole_021368.T1
1367.T1, Maole_021368.T1
1357.T1, Maole_011358.T1
1357.T1, Maole_011358.T1
4881.T1, Maole_005996.T1, Maole_006651.T1, Maole_006652.T1, Maole_010336.T1, Maole_
1020.T1, Maole_001021.T1, Maole_001022.T1
1020.T1, Maole_001021.T1, Maole_001022.T1
1424.T1, Maole_016541.T1, Maole_016542.T1
0182.T1, Maole_001538.T1, Maole_001986.T1, Maole_001987.T1, Maole_002591.T1, Maole_
2999.T1, Maole_003050.T1, Maole_012089.T1, Maole_013992.T1, Maole_013993.T1, Maole_
0212.T1, Maole_015203.T1, Maole_021341.T1, Maole_021342.T1, Maole_022379.T1, Maole_
2245.T1, Maole_002247.T1, Maole_002248.T1, Maole_002249.T1, Maole_002250.T1, Maole_
2953.T1, Maole_016874.T1
1986.T1, Maole_001987.T1
1986.T1, Maole_001987.T1
1986.T1, Maole_001987.T1
4982.T1, Maole_013561.T1
6478.T1, Maole_018141.T1
1357.T1, Maole_011358.T1
1357.T1, Maole_011358.T1
2953.T1, Maole_016874.T1
1424.T1, Maole_001461.T1, Maole_004639.T1, Maole_007827.T1, Maole_012162.T1, Maole_

005695.T1, Maole_005698.T1, Maole_005700.T1, Maole_005702.T1, Maole_007182.T1, Maol
005178.T1, Maole_005179.T1, Maole_005180.T1, Maole_005694.T1, Maole_005695.T1, Maol
004881.T1, Maole_004982.T1, Maole_011288.T1, Maole_011401.T1, Maole_012520.T1, Maol
001022.T1, Maole_001096.T1, Maole_001375.T1, Maole_001424.T1, Maole_001461.T1, Maol
002027.T1, Maole_003161.T1, Maole_003875.T1, Maole_004490.T1, Maole_004776.T1, Maol
004210.T1, Maole_004626.T1, Maole_006703.T1, Maole_010114.T1, Maole_010115.T1, Maol
001560.T1, Maole_002027.T1, Maole_003001.T1, Maole_003161.T1, Maole_003875.T1, Maol
004982.T1, Maole_007944.T1, Maole_011110.T1, Maole_011111.T1, Maole_011159.T1, Maol
004210.T1, Maole_004626.T1, Maole_006703.T1, Maole_010114.T1, Maole_010115.T1, Maol
014267.T1, Maole_014269.T1, Maole_014270.T1, Maole_014272.T1, Maole_014274.T1, Maol
003161.T1, Maole_003875.T1, Maole_004490.T1, Maole_004776.T1, Maole_005713.T1, Maol
002027.T1, Maole_003001.T1, Maole_003161.T1, Maole_003875.T1, Maole_004490.T1, Maol
007944.T1, Maole_011159.T1, Maole_011357.T1, Maole_011358.T1, Maole_011401.T1, Maol
014267.T1, Maole_014269.T1, Maole_014270.T1, Maole_014272.T1, Maole_014274.T1, Maol
003305.T1, Maole_003945.T1, Maole_006532.T1, Maole_006581.T1, Maole_006583.T1, Maol
002027.T1, Maole_002249.T1, Maole_002250.T1, Maole_002978.T1, Maole_003161.T1, Maol
002027.T1, Maole_003001.T1, Maole_003161.T1, Maole_003875.T1, Maole_004490.T1, Maol
002247.T1, Maole_002253.T1, Maole_002591.T1, Maole_002707.T1, Maole_003197.T1, Maol
003161.T1, Maole_003875.T1, Maole_004490.T1, Maole_004776.T1, Maole_005713.T1, Maol
006647.T1, Maole_006651.T1, Maole_006652.T1, Maole_006779.T1, Maole_009317.T1, Maol
001096.T1, Maole_001375.T1, Maole_001461.T1, Maole_001545.T1, Maole_001931.T1, Maol
006647.T1, Maole_006651.T1, Maole_006652.T1, Maole_006779.T1, Maole_009317.T1, Maol
001424.T1, Maole_001461.T1, Maole_001538.T1, Maole_001931.T1, Maole_002591.T1, Maol
003161.T1, Maole_003875.T1, Maole_004490.T1, Maole_004776.T1, Maole_005713.T1, Maol
006647.T1, Maole_006651.T1, Maole_006652.T1, Maole_006779.T1, Maole_009317.T1, Maol
006647.T1, Maole_006651.T1, Maole_006652.T1, Maole_006779.T1, Maole_009317.T1, Maol
023347.T1, Maole_023983.T1, Maole_023998.T1, Maole_024019.T1, Maole_024051.T1, Maol
002999.T1, Maole_003050.T1, Maole_003245.T1, Maole_003782.T1, Maole_003783.T1, Maol
006647.T1, Maole_006651.T1, Maole_006652.T1, Maole_006779.T1, Maole_009317.T1, Maol
010105.T1, Maole_019117.T1, Maole_020721.T1, Maole_021175.T1, Maole_021178.T1, Maol
015116.T1, Maole_015936.T1, Maole_017068.T1, Maole_020579.T1, Maole_021416.T1, Maol
010105.T1, Maole_019117.T1, Maole_021175.T1, Maole_021178.T1, Maole_021594.T1
006647.T1, Maole_006651.T1, Maole_006652.T1, Maole_006779.T1, Maole_007366.T1, Maol
008212.T1, Maole_010105.T1, Maole_011288.T1, Maole_013561.T1, Maole_014744.T1, Maol
008212.T1, Maole_009317.T1, Maole_009320.T1, Maole_010105.T1, Maole_014265.T1, Maol
006647.T1, Maole_006651.T1, Maole_006652.T1, Maole_006779.T1, Maole_009317.T1, Maol
014269.T1, Maole_014270.T1, Maole_014272.T1, Maole_014274.T1, Maole_014275.T1
005695.T1, Maole_005698.T1, Maole_005700.T1, Maole_005702.T1, Maole_007182.T1, Maol
010105.T1, Maole_019117.T1, Maole_020721.T1, Maole_021175.T1, Maole_021178.T1, Maol
009320.T1, Maole_014265.T1, Maole_014267.T1, Maole_014269.T1, Maole_014270.T1, Maol
013561.T1, Maole_014744.T1, Maole_017309.T1, Maole_021185.T1
022507.T1, Maole_022510.T1, Maole_022511.T1, Maole_022513.T1
023776.T1, Maole_024056.T1, Maole_024121.T1
023347.T1, Maole_023983.T1, Maole_023998.T1, Maole_024019.T1, Maole_024051.T1, Maol
023347.T1, Maole_023983.T1, Maole_023998.T1, Maole_024019.T1, Maole_024051.T1, Maol
023347.T1, Maole_023983.T1, Maole_023998.T1, Maole_024019.T1, Maole_024051.T1, Maol
014199.T1, Maole_016541.T1, Maole_016542.T1

014272.T1, Maole_014274.T1, Maole_014275.T1
003791.T1, Maole_003793.T1, Maole_019618.T1
014199.T1, Maole_016541.T1, Maole_016542.T1
003161.T1, Maole_003875.T1, Maole_004490.T1, Maole_004776.T1, Maole_005713.T1, Maol
022507.T1, Maole_022510.T1, Maole_022511.T1, Maole_022513.T1
003305.T1, Maole_003945.T1, Maole_005877.T1, Maole_005880.T1, Maole_006532.T1, Maol
006651.T1, Maole_006652.T1, Maole_006779.T1, Maole_007366.T1, Maole_009317.T1, Maol
013561.T1, Maole_014744.T1, Maole_017309.T1, Maole_021185.T1
023347.T1, Maole_023983.T1, Maole_023998.T1, Maole_024019.T1, Maole_024051.T1, Maol
004210.T1, Maole_004626.T1, Maole_006167.T1, Maole_006703.T1, Maole_010114.T1, Maol

014199.T1, Maole_016541.T1, Maole_016542.T1
023736.T1, Maole_023737.T1, Maole_023741.T1, Maole_023742.T1
013561.T1, Maole_014744.T1, Maole_017309.T1, Maole_021185.T1
014199.T1, Maole_016541.T1, Maole_016542.T1
023776.T1, Maole_024056.T1, Maole_024121.T1

003791.T1, Maole_003793.T1, Maole_003796.T1, Maole_019618.T1
003791.T1, Maole_003793.T1, Maole_003796.T1, Maole_019618.T1
013912.T1, Maole_013913.T1, Maole_013914.T1, Maole_020459.T1
002707.T1, Maole_002999.T1, Maole_003050.T1, Maole_004972.T1, Maole_005250.T1, Maol
013994.T1, Maole_014003.T1
013561.T1, Maole_014744.T1, Maole_017309.T1, Maole_021185.T1
022507.T1
022507.T1
014199.T1, Maole_016541.T1, Maole_016542.T1
023260.T1, Maole_023776.T1, Maole_024056.T1, Maole_024121.T1
023260.T1, Maole_023776.T1, Maole_024056.T1, Maole_024121.T1

023736.T1, Maole_023737.T1, Maole_023741.T1, Maole_023742.T1
010356.T1, Maole_010359.T1, Maole_010362.T1, Maole_012789.T1, Maole_012830.T1, Maol
003796.T1, Maole_019618.T1
014199.T1, Maole_015936.T1, Maole_017068.T1
010105.T1, Maole_019117.T1, Maole_020721.T1, Maole_021175.T1, Maole_021178.T1, Maol

006581.T1, Maole_006583.T1, Maole_008212.T1, Maole_010105.T1, Maole_019117.T1, Maol
021594.T1
010115.T1
001545.T1, Maole_001931.T1, Maole_002591.T1, Maole_003069.T1, Maole_003305.T1, Maol
023776.T1, Maole_024121.T1

002250.T1, Maole_002253.T1, Maole_002254.T1, Maole_002707.T1, Maole_003197.T1, Maol
014199.T1, Maole_016541.T1, Maole_016542.T1
023736.T1, Maole_023737.T1, Maole_023741.T1, Maole_023742.T1
012789.T1, Maole_017647.T1
015203.T1, Maole_021175.T1, Maole_021341.T1, Maole_021342.T1, Maole_021594.T1, Maol

011288.T1, Maole_012520.T1, Maole_012595.T1, Maole_012596.T1, Maole_012598.T1, Maol
023260.T1, Maole_023776.T1, Maole_024056.T1, Maole_024121.T1
023736.T1, Maole_023737.T1, Maole_023741.T1, Maole_023742.T1
016542.T1
003596.T1, Maole_010254.T1, Maole_010356.T1, Maole_010359.T1, Maole_010362.T1, Maol
002253.T1, Maole_002254.T1, Maole_002978.T1, Maole_003197.T1, Maole_003782.T1, Maol

001538.T1, Maole_001986.T1, Maole_001987.T1, Maole_003069.T1, Maole_003305.T1, Maol
011159.T1, Maole_011288.T1, Maole_013561.T1, Maole_013780.T1, Maole_014744.T1, Maol

014199.T1, Maole_016541.T1, Maole_016542.T1

011288.T1, Maole_012520.T1, Maole_012595.T1, Maole_012596.T1, Maole_012598.T1, Maol
013993.T1, Maole_013994.T1, Maole_014003.T1

023776.T1, Maole_024056.T1, Maole_024121.T1

021594.T1
003791.T1, Maole_003793.T1, Maole_003796.T1, Maole_019618.T1

023260.T1, Maole_023776.T1, Maole_024121.T1
017068.T1
002253.T1, Maole_003197.T1, Maole_015936.T1, Maole_017068.T1
023776.T1, Maole_024121.T1
006532.T1, Maole_008212.T1, Maole_010105.T1, Maole_010356.T1, Maole_010359.T1, Maol

003796.T1, Maole_019618.T1, Maole_022510.T1, Maole_022511.T1
017068.T1

023736.T1, Maole_023737.T1, Maole_023741.T1, Maole_023742.T1
010105.T1, Maole_015873.T1, Maole_019117.T1, Maole_021175.T1, Maole_021178.T1, Maol
014199.T1, Maole_016541.T1, Maole_016542.T1
002250.T1, Maole_002253.T1, Maole_002254.T1, Maole_003069.T1, Maole_003197.T1, Maol

012520.T1, Maole_012595.T1, Maole_012596.T1, Maole_012598.T1, Maole_016455.T1, Maol

006581.T1, Maole_006583.T1, Maole_009465.T1, Maole_011288.T1, Maole_017673.T1, Maol
013994.T1, Maole_014003.T1
023736.T1, Maole_023737.T1, Maole_023741.T1, Maole_023742.T1
002253.T1, Maole_002254.T1, Maole_002707.T1, Maole_002978.T1, Maole_003197.T1, Maol

014199.T1, Maole_016541.T1, Maole_016542.T1, Maole_022507.T1

e_007191.T1, Maole_007193.T1, Maole_007194.T1, Maole_007195.T1, Maole_007196.T1, Ma
e_005698.T1, Maole_005700.T1, Maole_005702.T1, Maole_007182.T1, Maole_007191.T1, Ma
e_012595.T1, Maole_012596.T1, Maole_012598.T1, Maole_013561.T1, Maole_014847.T1, Ma
e_001538.T1, Maole_001545.T1, Maole_001931.T1, Maole_001986.T1, Maole_001987.T1, Ma
e_005120.T1, Maole_005178.T1, Maole_005179.T1, Maole_005180.T1, Maole_005694.T1, Ma
e_010254.T1, Maole_010356.T1, Maole_010359.T1, Maole_010362.T1, Maole_012789.T1, Ma
e_004100.T1, Maole_004101.T1, Maole_004104.T1, Maole_004105.T1, Maole_004490.T1, Ma
e_011357.T1, Maole_011358.T1, Maole_011401.T1, Maole_013561.T1, Maole_013780.T1, Ma
e_010254.T1, Maole_010356.T1, Maole_010359.T1, Maole_010362.T1, Maole_012789.T1, Ma
e_014275.T1, Maole_015116.T1, Maole_021416.T1, Maole_023259.T1, Maole_023260.T1, Ma
e_007054.T1, Maole_009104.T1, Maole_009186.T1, Maole_010649.T1, Maole_012880.T1, Ma
e_004776.T1, Maole_005062.T1, Maole_005120.T1, Maole_005178.T1, Maole_005179.T1, Ma
e_013561.T1, Maole_013780.T1, Maole_014744.T1, Maole_016253.T1, Maole_020501.T1, Ma
e_014275.T1, Maole_015116.T1, Maole_015873.T1, Maole_021416.T1, Maole_023259.T1, Ma
e_007944.T1, Maole_008212.T1, Maole_010105.T1, Maole_011357.T1, Maole_011358.T1, Ma
e_003786.T1, Maole_003790.T1, Maole_003791.T1, Maole_003793.T1, Maole_003796.T1, Ma
e_004776.T1, Maole_005062.T1, Maole_005120.T1, Maole_005178.T1, Maole_005179.T1, Ma
e_004639.T1, Maole_004972.T1, Maole_004980.T1, Maole_004982.T1, Maole_005013.T1, Ma
e_007054.T1, Maole_009104.T1, Maole_009186.T1, Maole_010649.T1, Maole_012880.T1, Ma
e_009320.T1, Maole_010200.T1, Maole_010336.T1, Maole_011524.T1, Maole_011670.T1, Ma
e_004639.T1, Maole_004881.T1, Maole_004972.T1, Maole_004980.T1, Maole_004982.T1, Ma
e_009320.T1, Maole_010200.T1, Maole_010336.T1, Maole_011524.T1, Maole_011670.T1, Ma
e_002707.T1, Maole_003069.T1, Maole_003305.T1, Maole_003596.T1, Maole_003945.T1, Ma
e_007054.T1, Maole_009104.T1, Maole_009186.T1, Maole_010649.T1, Maole_011288.T1, Ma
e_009320.T1, Maole_010200.T1, Maole_010336.T1, Maole_011524.T1, Maole_011670.T1, Ma
e_009320.T1, Maole_010200.T1, Maole_010336.T1, Maole_011524.T1, Maole_011670.T1, Ma
e_024069.T1, Maole_024098.T1
e_003786.T1, Maole_003790.T1, Maole_003791.T1, Maole_003793.T1, Maole_003796.T1, Ma
e_009320.T1, Maole_010200.T1, Maole_010336.T1, Maole_011524.T1, Maole_011670.T1, Ma
e_021594.T1
e_023241.T1, Maole_023259.T1, Maole_023260.T1, Maole_023344.T1, Maole_023347.T1, Ma

e_009317.T1, Maole_009320.T1, Maole_010200.T1, Maole_010203.T1, Maole_010336.T1, Ma
e_017068.T1, Maole_017309.T1, Maole_017673.T1, Maole_019117.T1, Maole_021175.T1, Ma
e_014267.T1, Maole_014269.T1, Maole_014270.T1, Maole_014272.T1, Maole_014274.T1, Ma
e_009320.T1, Maole_010200.T1, Maole_010336.T1, Maole_011524.T1, Maole_011670.T1, Ma

e_007191.T1, Maole_007193.T1, Maole_007194.T1, Maole_007195.T1, Maole_007196.T1, Ma
e_021594.T1
e_014272.T1, Maole_014274.T1, Maole_014275.T1

e_024069.T1, Maole_024098.T1
e_024069.T1, Maole_024098.T1
e_024069.T1, Maole_024098.T1

e_007054.T1, Maole_009104.T1, Maole_009186.T1, Maole_010649.T1, Maole_012880.T1, Ma

e_006581.T1, Maole_006583.T1, Maole_006647.T1, Maole_007944.T1, Maole_008212.T1, Ma
e_009320.T1, Maole_010200.T1, Maole_010203.T1, Maole_011524.T1, Maole_011670.T1, Ma

e_024069.T1, Maole_024098.T1
e_010115.T1, Maole_010254.T1, Maole_010356.T1, Maole_010359.T1, Maole_010362.T1, Ma

e_007366.T1, Maole_009317.T1, Maole_009320.T1, Maole_012089.T1, Maole_013992.T1, Ma

e_017647.T1, Maole_019130.T1

e_021594.T1

e_021175.T1, Maole_021178.T1, Maole_021594.T1

e_003945.T1, Maole_005807.T1, Maole_006532.T1, Maole_006581.T1, Maole_006583.T1, Ma

e_006647.T1, Maole_007827.T1, Maole_012162.T1, Maole_014199.T1, Maole_015936.T1, Ma

e_022379.T1, Maole_023736.T1, Maole_023737.T1, Maole_023741.T1, Maole_023742.T1

e_014847.T1, Maole_016455.T1, Maole_016662.T1, Maole_017673.T1, Maole_021234.T1

e_012789.T1, Maole_012830.T1, Maole_015203.T1, Maole_017647.T1, Maole_019130.T1, Ma
e_003783.T1, Maole_003786.T1, Maole_003790.T1, Maole_003791.T1, Maole_003793.T1, Ma

e_003945.T1, Maole_004881.T1, Maole_004972.T1, Maole_004982.T1, Maole_006169.T1, Ma
e_017309.T1, Maole_017673.T1, Maole_021185.T1, Maole_022100.T1, Maole_022821.T1

e_014847.T1, Maole_016455.T1, Maole_016662.T1, Maole_017673.T1

e_010362.T1, Maole_015873.T1, Maole_016253.T1, Maole_017647.T1, Maole_019117.T1, Ma

e_021594.T1

e_003305.T1, Maole_003945.T1, Maole_006532.T1, Maole_007944.T1, Maole_008212.T1, Ma

e_020579.T1, Maole_021063.T1, Maole_022503.T1, Maole_022508.T1, Maole_023238.T1, Ma

e_022202.T1, Maole_022821.T1

e_003786.T1, Maole_003790.T1, Maole_003791.T1, Maole_003793.T1, Maole_003796.T1, Ma

ole_007198.T1, Maole_007199.T1, Maole_007201.T1, Maole_007202.T1, Maole_007203.T1, M
ole_007193.T1, Maole_007194.T1, Maole_007195.T1, Maole_007196.T1, Maole_007198.T1, M
ole_016253.T1, Maole_016455.T1, Maole_016662.T1, Maole_017673.T1, Maole_022100.T1
ole_002245.T1, Maole_002247.T1, Maole_002248.T1, Maole_002249.T1, Maole_002250.T1, M
ole_005695.T1, Maole_005698.T1, Maole_005700.T1, Maole_005702.T1, Maole_005713.T1, M
ole_012830.T1, Maole_017647.T1, Maole_019130.T1
ole_004776.T1, Maole_004982.T1, Maole_005062.T1, Maole_005713.T1, Maole_007054.T1, M
ole_014744.T1, Maole_016253.T1, Maole_016768.T1, Maole_017309.T1, Maole_020501.T1, M
ole_012830.T1, Maole_017647.T1, Maole_019130.T1
ole_023776.T1, Maole_024056.T1, Maole_024121.T1
ole_013187.T1, Maole_014526.T1, Maole_018119.T1, Maole_018229.T1, Maole_018251.T1, M
ole_005180.T1, Maole_005694.T1, Maole_005695.T1, Maole_005698.T1, Maole_005700.T1, M
ole_020502.T1, Maole_022100.T1
ole_023260.T1, Maole_023776.T1, Maole_024056.T1, Maole_024121.T1
ole_013780.T1, Maole_016253.T1, Maole_019117.T1, Maole_020501.T1, Maole_020502.T1, M
ole_003875.T1, Maole_004490.T1, Maole_004982.T1, Maole_005713.T1, Maole_007054.T1, M
ole_005180.T1, Maole_005694.T1, Maole_005695.T1, Maole_005698.T1, Maole_005700.T1, M
ole_005250.T1, Maole_006581.T1, Maole_006583.T1, Maole_006647.T1, Maole_006651.T1, M
ole_013187.T1, Maole_014526.T1, Maole_018119.T1, Maole_018229.T1, Maole_018251.T1, M
ole_014265.T1, Maole_014267.T1, Maole_014269.T1, Maole_014270.T1, Maole_014272.T1, M
ole_005013.T1, Maole_005250.T1, Maole_005996.T1, Maole_006167.T1, Maole_006224.T1, M
ole_014265.T1, Maole_014267.T1, Maole_014269.T1, Maole_014270.T1, Maole_014272.T1, M
ole_004100.T1, Maole_004101.T1, Maole_004104.T1, Maole_004105.T1, Maole_004639.T1, M
ole_012880.T1, Maole_013187.T1, Maole_014526.T1, Maole_014744.T1, Maole_016478.T1, M
ole_014265.T1, Maole_014267.T1, Maole_014269.T1, Maole_014270.T1, Maole_014272.T1, M
ole_014265.T1, Maole_014267.T1, Maole_014269.T1, Maole_014270.T1, Maole_014272.T1, M

ole_004210.T1, Maole_004626.T1, Maole_006703.T1, Maole_007944.T1, Maole_009606.T1, M
ole_014265.T1, Maole_014267.T1, Maole_014269.T1, Maole_014270.T1, Maole_014272.T1, M

ole_023776.T1, Maole_023983.T1, Maole_024051.T1, Maole_024056.T1, Maole_024069.T1, M

ole_011524.T1, Maole_011670.T1, Maole_014265.T1, Maole_014267.T1, Maole_014270.T1, M
ole_021178.T1, Maole_021594.T1
ole_014275.T1, Maole_019117.T1, Maole_021175.T1, Maole_021178.T1, Maole_021594.T1, M
ole_014265.T1, Maole_014267.T1, Maole_014269.T1, Maole_014270.T1, Maole_014272.T1, M

ole_007198.T1, Maole_007199.T1, Maole_007201.T1, Maole_007202.T1, Maole_007203.T1, M

ole_013187.T1, Maole_014526.T1, Maole_018119.T1, Maole_018229.T1, Maole_018251.T1, M

ole_010105.T1, Maole_011357.T1, Maole_011358.T1, Maole_013780.T1, Maole_016253.T1, M
ole_014265.T1, Maole_014267.T1, Maole_014269.T1, Maole_014270.T1, Maole_014272.T1, M

ole_012789.T1, Maole_012830.T1, Maole_017647.T1, Maole_019130.T1

ole_013993.T1, Maole_013994.T1, Maole_014003.T1, Maole_014265.T1, Maole_014267.T1, M

ole_007944.T1, Maole_008212.T1, Maole_010105.T1, Maole_011357.T1, Maole_011358.T1, M

ole_016541.T1, Maole_016542.T1, Maole_017068.T1, Maole_023238.T1, Maole_023998.T1

ole_021341.T1, Maole_021342.T1, Maole_022379.T1, Maole_023736.T1, Maole_023737.T1, M
ole_003796.T1, Maole_004210.T1, Maole_009606.T1, Maole_009607.T1, Maole_009608.T1, M

ole_006532.T1, Maole_008212.T1, Maole_009317.T1, Maole_009320.T1, Maole_009465.T1, M

ole_021175.T1, Maole_021178.T1, Maole_021594.T1, Maole_022100.T1

ole_009738.T1, Maole_010105.T1, Maole_013780.T1, Maole_015873.T1, Maole_015936.T1, M

ole_023239.T1, Maole_023241.T1, Maole_023344.T1, Maole_023347.T1, Maole_023983.T1, M

ole_015936.T1, Maole_017068.T1, Maole_019618.T1

Maole_007204.T1, Maole_007205.T1, Maole_007208.T1, Maole_007209.T1, Maole_007211.T1
Maole_007199.T1, Maole_007201.T1, Maole_007202.T1, Maole_007203.T1, Maole_007205.T1

Maole_002253.T1, Maole_002254.T1, Maole_002591.T1, Maole_002707.T1, Maole_003069.T1
Maole_007054.T1, Maole_007081.T1, Maole_007182.T1, Maole_007191.T1, Maole_007193.T1

Maole_007081.T1, Maole_009104.T1, Maole_009186.T1, Maole_009465.T1, Maole_010649.T1
Maole_020502.T1, Maole_021185.T1, Maole_022100.T1, Maole_022821.T1

Maole_019211.T1, Maole_019276.T1, Maole_023134.T1
Maole_005702.T1, Maole_005713.T1, Maole_007054.T1, Maole_007081.T1, Maole_007182.T1

Maole_021175.T1, Maole_021178.T1, Maole_021594.T1, Maole_022100.T1, Maole_022821.T1
Maole_007081.T1, Maole_009104.T1, Maole_009186.T1, Maole_010649.T1, Maole_012880.T1
Maole_005702.T1, Maole_005713.T1, Maole_007054.T1, Maole_007081.T1, Maole_007182.T1
Maole_006652.T1, Maole_006779.T1, Maole_007944.T1, Maole_009317.T1, Maole_009320.T1
Maole_019211.T1, Maole_019276.T1, Maole_023134.T1
Maole_014274.T1, Maole_014275.T1, Maole_014430.T1, Maole_014431.T1, Maole_015116.T1
Maole_006226.T1, Maole_006227.T1, Maole_006262.T1, Maole_006647.T1, Maole_006651.T1
Maole_014274.T1, Maole_014275.T1, Maole_014430.T1, Maole_014431.T1, Maole_015116.T1
Maole_004881.T1, Maole_004972.T1, Maole_004980.T1, Maole_004982.T1, Maole_005013.T1
Maole_017309.T1, Maole_017673.T1, Maole_018119.T1, Maole_018141.T1, Maole_018229.T1
Maole_014274.T1, Maole_014275.T1, Maole_014430.T1, Maole_014431.T1, Maole_015116.T1
Maole_014274.T1, Maole_014275.T1, Maole_014430.T1, Maole_014431.T1, Maole_015116.T1

Maole_009607.T1, Maole_009608.T1, Maole_009738.T1, Maole_010114.T1, Maole_010115.T1
Maole_014274.T1, Maole_014275.T1, Maole_014430.T1, Maole_014431.T1, Maole_015116.T1

Maole_024121.T1

Maole_014272.T1, Maole_014274.T1, Maole_014275.T1, Maole_014430.T1, Maole_014431.T1

Maole_022503.T1
Maole_014274.T1, Maole_014275.T1, Maole_014430.T1, Maole_014431.T1, Maole_015116.T1

Maole_007204.T1, Maole_007205.T1, Maole_007208.T1, Maole_007209.T1, Maole_007211.T1

Maole_019211.T1, Maole_019276.T1, Maole_023134.T1

Maole_016881.T1, Maole_019117.T1, Maole_020501.T1, Maole_020502.T1, Maole_020579.T1
Maole_014274.T1, Maole_014275.T1, Maole_014430.T1, Maole_014431.T1, Maole_014883.T1

Maole_014269.T1, Maole_014270.T1, Maole_014272.T1, Maole_014274.T1, Maole_014275.T1

Maole_013780.T1, Maole_016253.T1, Maole_019117.T1, Maole_020501.T1, Maole_020502.T1

Maole_023741.T1, Maole_023742.T1
Maole_009738.T1, Maole_015936.T1, Maole_017068.T1, Maole_019130.T1, Maole_019618.T1

Maole_010105.T1, Maole_011288.T1, Maole_011401.T1, Maole_011670.T1, Maole_012520.T1

Maole_017068.T1, Maole_019117.T1, Maole_021175.T1, Maole_021178.T1, Maole_021594.T1

[illegible]aole_023998.T1, Maole_024019.T1, Maole_024051.T1, Maole_024069.T1, Maole_024098.T1

, Maole_007214.T1, Maole_009325.T1, Maole_009326.T1, Maole_010377.T1, Maole_013041.T
, Maole_007206.T1, Maole_007208.T1, Maole_007209.T1, Maole_007211.T1, Maole_007214.T

, Maole_003197.T1, Maole_003305.T1, Maole_003945.T1, Maole_004639.T1, Maole_004881.T
, Maole_007194.T1, Maole_007195.T1, Maole_007196.T1, Maole_007198.T1, Maole_007199.T

, Maole_012880.T1, Maole_013187.T1, Maole_013561.T1, Maole_014526.T1, Maole_014744.T

, Maole_007191.T1, Maole_007193.T1, Maole_007194.T1, Maole_007195.T1, Maole_007196.T

, Maole_013187.T1, Maole_013561.T1, Maole_014526.T1, Maole_014744.T1, Maole_017309.T
, Maole_007191.T1, Maole_007193.T1, Maole_007194.T1, Maole_007195.T1, Maole_007196.T
, Maole_010200.T1, Maole_010203.T1, Maole_010336.T1, Maole_011111.T1, Maole_011288.T

, Maole_015166.T1, Maole_015167.T1, Maole_015667.T1, Maole_020715.T1, Maole_020721.T
, Maole_006652.T1, Maole_006779.T1, Maole_007366.T1, Maole_007944.T1, Maole_009317.T
, Maole_015166.T1, Maole_015167.T1, Maole_015667.T1, Maole_020715.T1, Maole_020721.T
, Maole_005250.T1, Maole_005877.T1, Maole_005880.T1, Maole_006214.T1, Maole_006532.T
, Maole_018251.T1, Maole_019211.T1, Maole_019276.T1, Maole_023134.T1
, Maole_015167.T1, Maole_015667.T1, Maole_020715.T1, Maole_020721.T1, Maole_021063.T
, Maole_015167.T1, Maole_015667.T1, Maole_020715.T1, Maole_020721.T1, Maole_021063.T

, Maole_010254.T1, Maole_010356.T1, Maole_010359.T1, Maole_010362.T1, Maole_012089.T
, Maole_015166.T1, Maole_015167.T1, Maole_015667.T1, Maole_015873.T1, Maole_020715.T

, Maole_014883.T1, Maole_014887.T1, Maole_015116.T1, Maole_015166.T1, Maole_015167.T

, Maole_015167.T1, Maole_015667.T1, Maole_020715.T1, Maole_020721.T1, Maole_021063.T

, Maole_007214.T1, Maole_009325.T1, Maole_009326.T1, Maole_010377.T1, Maole_013041.T

, Maole_021175.T1, Maole_021178.T1, Maole_021594.T1, Maole_022100.T1, Maole_022821.[illegible]
, Maole_014887.T1, Maole_015166.T1, Maole_015873.T1, Maole_018787.T1, Maole_020715.[illegible]

, Maole_015873.T1

, Maole_021175.T1, Maole_021178.T1, Maole_021594.T1, Maole_022100.T1, Maole_022821.[illegible]

, Maole_022503.T1, Maole_022508.T1, Maole_023239.T1, Maole_023259.T1, Maole_023260.[illegible]

, Maole_012595.T1, Maole_012596.T1, Maole_012598.T1, Maole_013561.T1, Maole_014265.[illegible]

T1, Maole_015999.T1, Maole_020337.T1, Maole_020346.T1, Maole_021033.T1, Maole_02237
T1, Maole_009325.T1, Maole_009326.T1, Maole_010377.T1, Maole_013042.T1, Maole_01452

T1, Maole_004972.T1, Maole_004980.T1, Maole_004982.T1, Maole_005013.T1, Maole_00580
T1, Maole_007201.T1, Maole_007202.T1, Maole_007203.T1, Maole_007204.T1, Maole_00720

T1, Maole_017309.T1, Maole_018069.T1, Maole_018119.T1, Maole_018229.T1, Maole_01825

T1, Maole_007198.T1, Maole_007199.T1, Maole_007201.T1, Maole_007202.T1, Maole_00720

T1, Maole_018119.T1, Maole_018229.T1, Maole_018251.T1, Maole_019211.T1, Maole_01927
T1, Maole_007198.T1, Maole_007199.T1, Maole_007201.T1, Maole_007202.T1, Maole_00720
T1, Maole_011357.T1, Maole_011358.T1, Maole_011524.T1, Maole_011670.T1, Maole_01356

T1, Maole_021063.T1, Maole_021416.T1, Maole_022500.T1, Maole_022503.T1, Maole_02250
T1, Maole_009320.T1, Maole_009984.T1, Maole_010200.T1, Maole_010203.T1, Maole_01033
T1, Maole_021063.T1, Maole_021416.T1, Maole_022500.T1, Maole_022504.T1, Maole_02250
T1, Maole_006581.T1, Maole_006583.T1, Maole_006647.T1, Maole_006651.T1, Maole_00665

T1, Maole_021416.T1, Maole_022500.T1, Maole_022507.T1, Maole_022510.T1, Maole_02251
T1, Maole_021416.T1, Maole_022500.T1, Maole_022507.T1, Maole_022510.T1, Maole_02251

T1, Maole_012789.T1, Maole_012830.T1, Maole_013780.T1, Maole_013992.T1, Maole_01399
T1, Maole_020721.T1, Maole_021063.T1, Maole_021416.T1, Maole_022500.T1, Maole_02250

T1, Maole_015667.T1, Maole_015873.T1, Maole_018787.T1, Maole_020715.T1, Maole_02072

T1, Maole_021416.T1, Maole_022500.T1, Maole_022507.T1, Maole_022510.T1, Maole_02251

T1, Maole_015999.T1, Maole_020337.T1, Maole_020346.T1, Maole_021033.T1, Maole_02237

Γ1, Maole_023238.T1, Maole_023239.T1, Maole_023241.T1, Maole_023344.T1, Maole_02334
Γ1, Maole_020721.T1, Maole_021063.T1, Maole_021415.T1, Maole_021416.T1, Maole_02250

Γ1

Γ1, Maole_024019.T1

Γ1, Maole_014267.T1, Maole_014269.T1, Maole_014270.T1, Maole_014272.T1, Maole_01427

1.T1, Maole_022372.T1, Maole_022373.T1, Maole_023452.T1, Maole_023453.T1
6.T1, Maole_015999.T1, Maole_018119.T1, Maole_020346.T1, Maole_021033.T1, Maole_022

7.T1, Maole_005877.T1, Maole_005880.T1, Maole_006169.T1, Maole_006214.T1, Maole_006
5.T1, Maole_007206.T1, Maole_007208.T1, Maole_007209.T1, Maole_007211.T1, Maole_007

1.T1, Maole_018328.T1, Maole_019106.T1, Maole_019211.T1, Maole_019276.T1, Maole_020

3.T1, Maole_007204.T1, Maole_007205.T1, Maole_007206.T1, Maole_007208.T1, Maole_007

6.T1, Maole_019618.T1, Maole_020748.T1, Maole_021963.T1, Maole_023134.T1
3.T1, Maole_007204.T1, Maole_007205.T1, Maole_007206.T1, Maole_007208.T1, Maole_007
1.T1, Maole_013780.T1, Maole_014265.T1, Maole_014267.T1, Maole_014269.T1, Maole_014

4.T1, Maole_022507.T1, Maole_022508.T1, Maole_022510.T1, Maole_022511.T1, Maole_022
6.T1, Maole_011110.T1, Maole_011111.T1, Maole_011159.T1, Maole_011288.T1, Maole_011
7.T1, Maole_022510.T1, Maole_022511.T1, Maole_022513.T1, Maole_023259.T1, Maole_023
2.T1, Maole_006779.T1, Maole_007827.T1, Maole_008212.T1, Maole_009317.T1, Maole_009

1.T1, Maole_022513.T1, Maole_023259.T1, Maole_023260.T1, Maole_023776.T1, Maole_023
1.T1, Maole_022513.T1, Maole_023259.T1, Maole_023260.T1, Maole_023776.T1, Maole_023

3.T1, Maole_013994.T1, Maole_014003.T1, Maole_017647.T1, Maole_019130.T1, Maole_019
3.T1, Maole_022504.T1, Maole_022507.T1, Maole_022508.T1, Maole_022510.T1, Maole_022

1.T1, Maole_021063.T1, Maole_021415.T1, Maole_021416.T1, Maole_022500.T1, Maole_022

1.T1, Maole_022513.T1, Maole_023259.T1, Maole_023260.T1, Maole_023776.T1, Maole_023

1.T1, Maole_022372.T1, Maole_022373.T1, Maole_023452.T1, Maole_023453.T1

7.T1, Maole_023983.T1, Maole_024019.T1, Maole_024051.T1, Maole_024069.T1, Maole_024[illegible]
0.T1, Maole_022503.T1, Maole_022504.T1, Maole_022507.T1, Maole_022508.T1, Maole_022[illegible]

4.T1, Maole_014275.T1, Maole_014430.T1, Maole_014744.T1, Maole_014847.T1, Maole_016[illegible]

368.T1, Maole_022371.T1, Maole_022372.T1, Maole_022373.T1, Maole_023450.T1, Maole_02

532.T1, Maole_006581.T1, Maole_006583.T1, Maole_006647.T1, Maole_006651.T1, Maole_00
214.T1, Maole_009104.T1, Maole_009186.T1, Maole_009325.T1, Maole_009326.T1, Maole_01

748.T1, Maole_021963.T1, Maole_023024.T1, Maole_023134.T1

209.T1, Maole_007211.T1, Maole_007214.T1, Maole_009104.T1, Maole_009186.T1, Maole_00

209.T1, Maole_007211.T1, Maole_007214.T1, Maole_009104.T1, Maole_009186.T1, Maole_00
270.T1, Maole_014272.T1, Maole_014274.T1, Maole_014275.T1, Maole_014430.T1, Maole_01

512.T1, Maole_022513.T1, Maole_023259.T1, Maole_023260.T1, Maole_023776.T1, Maole_02
357.T1, Maole_011358.T1, Maole_011401.T1, Maole_011524.T1, Maole_011670.T1, Maole_01
260.T1, Maole_023776.T1, Maole_023917.T1, Maole_024056.T1, Maole_024121.T1
320.T1, Maole_009465.T1, Maole_010105.T1, Maole_010200.T1, Maole_010203.T1, Maole_01

917.T1, Maole_024056.T1, Maole_024121.T1
917.T1, Maole_024056.T1, Maole_024121.T1

618.T1, Maole_020501.T1, Maole_020502.T1, Maole_022100.T1
511.T1, Maole_022512.T1, Maole_022513.T1, Maole_023259.T1, Maole_023260.T1, Maole_02

503.T1, Maole_022504.T1, Maole_022507.T1, Maole_022508.T1, Maole_022510.T1, Maole_02

917.T1, Maole_024056.T1, Maole_024121.T1

098.T1
510.T1, Maole_022511.T1, Maole_022512.T1, Maole_023385.T1, Maole_023917.T1, Maole_0[illegible]

455.T1, Maole_016662.T1, Maole_017309.T1, Maole_017644.T1, Maole_017673.T1, Maole_01

23452.T1, Maole_023453.T1, Maole_023556.T1, Maole_023558.T1, Maole_023986.T1, Maole_

06652.T1, Maole_006779.T1, Maole_007827.T1, Maole_007944.T1, Maole_008212.T1, Maole_
10377.T1, Maole_010649.T1, Maole_011635.T1, Maole_012880.T1, Maole_013041.T1, Maole_

09325.T1, Maole_009326.T1, Maole_010377.T1, Maole_010649.T1, Maole_011635.T1, Maole_

09325.T1, Maole_009326.T1, Maole_010377.T1, Maole_010649.T1, Maole_011635.T1, Maole_
14431.T1, Maole_014744.T1, Maole_014883.T1, Maole_014887.T1, Maole_015116.T1, Maole_

23917.T1, Maole_024056.T1, Maole_024121.T1
12520.T1, Maole_012595.T1, Maole_012596.T1, Maole_012598.T1, Maole_012798.T1, Maole_

10336.T1, Maole_011110.T1, Maole_011111.T1, Maole_011288.T1, Maole_011524.T1, Maole_

23776.T1, Maole_023917.T1, Maole_024056.T1, Maole_024121.T1

22511.T1, Maole_022512.T1, Maole_022513.T1, Maole_023259.T1, Maole_023260.T1, Maole_

24121.T1

19117.T1, Maole_020721.T1, Maole_021175.T1, Maole_021178.T1, Maole_021594.T1, Maole_

_023988.T1, Maole_024067.T1

_009317.T1, Maole_009320.T1, Maole_009465.T1, Maole_010105.T1, Maole_010200.T1, Mao
_013042.T1, Maole_013187.T1, Maole_013907.T1, Maole_013908.T1, Maole_013909.T1, Mao

_012880.T1, Maole_013041.T1, Maole_013042.T1, Maole_013187.T1, Maole_013907.T1, Mao

_012880.T1, Maole_013041.T1, Maole_013042.T1, Maole_013187.T1, Maole_013907.T1, Mao
_015166.T1, Maole_015167.T1, Maole_015667.T1, Maole_015936.T1, Maole_016253.T1, Mao

_013561.T1, Maole_013780.T1, Maole_014265.T1, Maole_014267.T1, Maole_014269.T1, Mao

_011670.T1, Maole_012162.T1, Maole_012520.T1, Maole_012595.T1, Maole_012596.T1, Mao

_023385.T1, Maole_023776.T1, Maole_023917.T1, Maole_024056.T1, Maole_024121.T1

_022202.T1, Maole_022503.T1, Maole_022508.T1, Maole_023259.T1, Maole_023260.T1, Mao

le_010203.T1, Maole_010336.T1, Maole_010943.T1, Maole_010944.T1, Maole_010945.T1, Ma
le_013911.T1, Maole_013912.T1, Maole_013913.T1, Maole_013914.T1, Maole_014526.T1, Ma

le_013908.T1, Maole_013909.T1, Maole_013911.T1, Maole_013912.T1, Maole_013913.T1, Ma

le_013908.T1, Maole_013909.T1, Maole_013911.T1, Maole_013912.T1, Maole_013913.T1, Ma
le_016541.T1, Maole_016542.T1, Maole_016662.T1, Maole_016768.T1, Maole_017068.T1, Ma

le_014270.T1, Maole_014272.T1, Maole_014274.T1, Maole_014275.T1, Maole_014430.T1, Ma

le_012598.T1, Maole_013561.T1, Maole_014199.T1, Maole_014265.T1, Maole_014267.T1, Ma

le_023776.T1, Maole_023917.T1, Maole_024056.T1, Maole_024121.T1

aole_010946.T1, Maol
aole_015999.T1, Maol

aole_013914.T1, Maol

aole_013914.T1, Maol
aole_017309.T1, Maol

aole_014431.T1, Maol

aole_014269.T1, Maol

Table

Click here to download Table Table S20.xlsx

**Category**	**P_value**	**Q_value**	**numDEIr**	**numInCa**	**Term**
ko04075	6.65E-29	8.71E-27	45	264	Plant horr
ko02010	3.11E-15	2.04E-13	14	33	ABC trans
ko04626	1.25E-08	5.46E-07	22	236	Plant-path
ko00190	0.000127	0.004173	11	125	Oxidative

**Class**	**Gene ID**
Environmental Information Processing; Signal transduction	Maole_000378.T1, Maole_0004[illegible]
Environmental Information Processing; Membrane transport	Maole_001545.T1, Maole_0049[illegible]
Organismal Systems; Environmental adaptation	Maole_004972.T1, Maole_0059[illegible]
Metabolism; Energy metabolism	Maole_003069.T1, Maole_0033[illegible]

437.T1, Maole_000677.T1, Maole_001434.T1, Maole_001560.T1, Maole_002027.T1, Maole_00
980.T1, Maole_004982.T1, Maole_011110.T1, Maole_011111.T1, Maole_011159.T1, Maole_01
996.T1, Maole_006214.T1, Maole_006779.T1, Maole_009317.T1, Maole_009320.T1, Maole_01
305.T1, Maole_003945.T1, Maole_006167.T1, Maole_006532.T1, Maole_008212.T1, Maole_01

)2978.T1, Maole_003001.T1, Maole_003161.T1, Maole_003786.T1, Maole_003790.T1, Maole_
11401.T1, Maole_013561.T1, Maole_014744.T1, Maole_016768.T1, Maole_017309.T1, Maole_
14265.T1, Maole_014267.T1, Maole_014269.T1, Maole_014270.T1, Maole_014272.T1, Maole_
10105.T1, Maole_012798.T1, Maole_021175.T1, Maole_021178.T1, Maole_021594.T1

_003791.T1, Maole_003793.T1, Maole_003796.T1, Maole_003875.T1, Maole_004100.T1, Maol
_021185.T1, Maole_022100.T1, Maole_022821.T1
_014274.T1, Maole_014275.T1, Maole_015116.T1, Maole_017644.T1, Maole_019939.T1, Maol

le_004101.T1, Maole_004104.T1, Maole_004105.T1, Maole_004490.T1, Maole_004776.T1, Ma

le_021416.T1, Maole_023259.T1, Maole_023260.T1, Maole_023776.T1, Maole_024056.T1, Ma

[illegible]aole_005062.T1, Maole_005713.T1, Maole_007054.T1, Maole_007081.T1, Maole_007366.T1, [illegible]

[illegible]aole_024121.T1

Maole_009104.T1, Maole_009186.T1, Maole_010649.T1, Maole_012880.T1, Maole_013187.T1

l, Maole_014526.T1, Maole_015873.T1, Maole_018119.T1, Maole_018229.T1, Maole_018251.

T1, Maole_018328.T1, Maole_019106.T1, Maole_019211.T1, Maole_019276.T1, Maole_0196

18.T1, Maole_020748.T1, Maole_021963.T1, Maole_023024.T1, Maole_023134.T1

Table Click here to download Table Table S21.xlsx

Cluster	Record	Type	From	To	Size (kb)
Cluster 17	scaffold38	Alkaloid	1	389,285	389.28
Cluster 21	scaffold67	Alkaloid	2,978,547	3,622,210	643.66
Cluster 1	scaffold13	Lignan-Polyketide	981,159	1,376,309	395.15
Cluster 23	scaffold8	Polyketide	4,529,957	5,812,302	1282.35
Cluster 2	scaffold13	Putative	34,174	689,685	655.51
Cluster 4	scaffold15	Putative	2,232,746	2,490,786	258.04
Cluster 9	scaffold26	Putative	1,079,703	2,003,831	924.13
Cluster 12	scaffold31	Putative	295,028	941,249	646.22
Cluster 14	scaffold33	Putative	760,835	1,070,430	309.6
Cluster 5	scaffold16	Saccharide	580,781	1,087,231	506.45
Cluster 6	scaffold17	Saccharide	1,847,925	2,973,087	1125.16
Cluster 10	scaffold28	Saccharide	511,674	1,729,579	1217.9
Cluster 13	scaffold33	Saccharide	7,536,717	8,466,817	930.1
Cluster 15	scaffold34	Saccharide	5,724,863	6,208,948	484.08
Cluster 16	scaffold36	Saccharide	2,373,331	2,926,426	553.1
Cluster 18	scaffold39	Saccharide	578,831	793,915	215.08
Cluster 20	scaffold54	Saccharide	7,265,034	8,021,036	756
Cluster 22	scaffold73	Saccharide	1,429,564	2,050,061	620.5
Cluster 8	scaffold21	Saccharide-Polyketide	9,844,715	10,494,316	649.6
Cluster 3	scaffold14	Terpene	234,014	673,747	439.73
Cluster 7	scaffold17	Terpene	582,938	1,206,986	624.05
Cluster 11	scaffold30	Terpene	1,065,427	1,391,381	325.95
Cluster 19	scaffold42	Terpene	1,159,540	1,602,377	442.84

Core domains	Gene cluster type	Gene clus
AMP-binding, Str_synth, p450	alkaloid	Maole_02
Cu_amine_oxid, Epimerase, p450	alkaloid	Maole_01
Chal_sti_synt_C, Dirigent, p450	lignan-polyketide	Maole_01
Chal_sti_synt_C, Chal_sti_synt_N, p450	polyketide	Maole_00
HMGL-like, Peptidase_S10, adh_short	putative	Maole_00
Transferase, adh_short	putative	Maole_00
2OG-FeII_Oxy, Aminotran_1_2, DIOX_N	putative	Maole_01
Methyltransf_11, p450	putative	Maole_01
Methyltransf_11, adh_short, p450	putative	Maole_01
Peptidase_S10, UDPGT_2	saccharide	Maole_01
Amino_oxidase, DAHP_synth_2, UDPGT_2, UbiA	saccharide	Maole_01
Acetyltransf_1, UDPGT_2, p450	saccharide	Maole_01
UDPGT_2, p450	saccharide	Maole_00
Glycos_transf_1, UDPGT_2	saccharide	Maole_00
Glycos_transf_2, SE	saccharide	Maole_02
Epimerase, FA_desaturase, UDPGT_2, p450	saccharide	Maole_02
Acetyltransf_1, Methyltransf_11, Peptidase_S10, UDPGT_2	saccharide	Maole_00
Epimerase, Glycos_transf_1, adh_short, adh_short_C2	saccharide	Maole_00
Chal_sti_synt_C, Chal_sti_synt_N, SE, UDPGT_2	saccharide-polyketide	Maole_00
Terpene_synth, Terpene_synth_C, p450	terpene	Maole_00
Epimerase, Prenyltrans, Terpene_synth, Terpene_synth_C	terpene	Maole_01
Acetyltransf_1, Terpene_synth, Terpene_synth_C	terpene	Maole_01
SQHop_cyclase_C, SQHop_cyclase_N, Terpene_synth, Terpe	terpene	Maole_01

ster genes

3229;Maole_023230;Maole_023231;Maole_023232;Maole_023233;Maole_023234;Maole_0232
6080;Maole_016081;Maole_016082;Maole_016083;Maole_016084;Maole_016085;Maole_0160
7148;Maole_017149;Maole_017150;Maole_017151;Maole_017152;Maole_017153;Maole_0171
8020;Maole_008021;Maole_008022;Maole_008023;Maole_008024
6918;Maole_006919;Maole_006920;Maole_006921;Maole_006922;Maole_006923;Maole_0069
8905;Maole_008906;Maole_008907;Maole_008908;Maole_008909;Maole_008910;Maole_0089
2577;Maole_012578;Maole_012579;Maole_012580;Maole_012581;Maole_012582;Maole_0125
7973;Maole_017974;Maole_017975;Maole_017976;Maole_017977;Maole_017978;Maole_0179
7520;Maole_017521;Maole_017522;Maole_017523;Maole_017524;Maole_017525;Maole_0175
5577;Maole_015578;Maole_015579;Maole_015580;Maole_015581;Maole_015582;Maole_0155
9301;Maole_019302;Maole_019303;Maole_019304;Maole_019305;Maole_019306;Maole_0193
7274;Maole_017275;Maole_017276;Maole_017277;Maole_017278;Maole_017279;Maole_0172
1301;Maole_001302;Maole_001303;Maole_001304;Maole_001305;Maole_001306;Maole_0013
7253;Maole_007254;Maole_007255;Maole_007256;Maole_007257;Maole_007258;Maole_0072
0684;Maole_020685;Maole_020686;Maole_020687;Maole_020688;Maole_020689
1736;Maole_021737;Maole_021738;Maole_021739;Maole_021740;Maole_021741;Maole_0217
0812;Maole_000813;Maole_000814;Maole_000815;Maole_000816;Maole_000817;Maole_0008
0957;Maole_000958;Maole_000959;Maole_000960;Maole_000961;Maole_000962;Maole_0009
2119;Maole_002120;Maole_002121;Maole_002122;Maole_002123;Maole_002124;Maole_0021
2169;Maole_002170;Maole_002171;Maole_002172;Maole_002173;Maole_002174;Maole_0021
4441;Maole_014442;Maole_014443;Maole_014444;Maole_014445;Maole_014446;Maole_0144
7612;Maole_017613;Maole_017614;Maole_017615;Maole_017616;Maole_017617;Maole_0176
6872;Maole_016873;Maole_016874;Maole_016875;Maole_016876;Maole_016877;Maole_0168

235;Maole_023236
086;Maole_016087;Maole_016088;Maole_016089;Maole_016090;Maole_016091
154

924;Maole_006925;Maole_006926;Maole_006927;Maole_006928;Maole_006929;Maole_00693
911;Maole_008912;Maole_008913;Maole_008914;Maole_008915;Maole_008916
583;Maole_012584;Maole_012585;Maole_012586;Maole_012587;Maole_012588;Maole_01258
979;Maole_017980;Maole_017981;Maole_017982;Maole_017983;Maole_017984;Maole_01798
526;Maole_017527;Maole_017528;Maole_017529;Maole_017530;Maole_017531;Maole_01753
583;Maole_015584;Maole_015585
307;Maole_019308;Maole_019309;Maole_019310;Maole_019311;Maole_019312;Maole_01931
280;Maole_017281;Maole_017282;Maole_017283;Maole_017284;Maole_017285;Maole_01728
307
259;Maole_007260;Maole_007261;Maole_007262;Maole_007263;Maole_007264;Maole_00726

742
318;Maole_000819;Maole_000820;Maole_000821;Maole_000822;Maole_000823;Maole_00082
963;Maole_000964;Maole_000965;Maole_000966;Maole_000967;Maole_000968
125;Maole_002126;Maole_002127;Maole_002128;Maole_002129
175;Maole_002176;Maole_002177;Maole_002178;Maole_002179;Maole_002180;Maole_00218
447;Maole_014448;Maole_014449;Maole_014450;Maole_014451;Maole_014452;Maole_01445
518
378

30

39;Maole_012590;Maole_012591;Maole_012592;Maole_012593;Maole_012594;Maole_012595
35;Maole_017986;Maole_017987
32;Maole_017533

13;Maole_019314
36

55;Maole_007266

24;Maole_000825;Maole_000826;Maole_000827;Maole_000828;Maole_000829;Maole_000830

31;Maole_002182;Maole_002183;Maole_002184;Maole_002185
53;Maole_014454;Maole_014455;Maole_014456

;Maole_000831;Maole_000832;Maole_000833;Maole_000834

Figure

Click here to download Figure Fig_S1.pdf

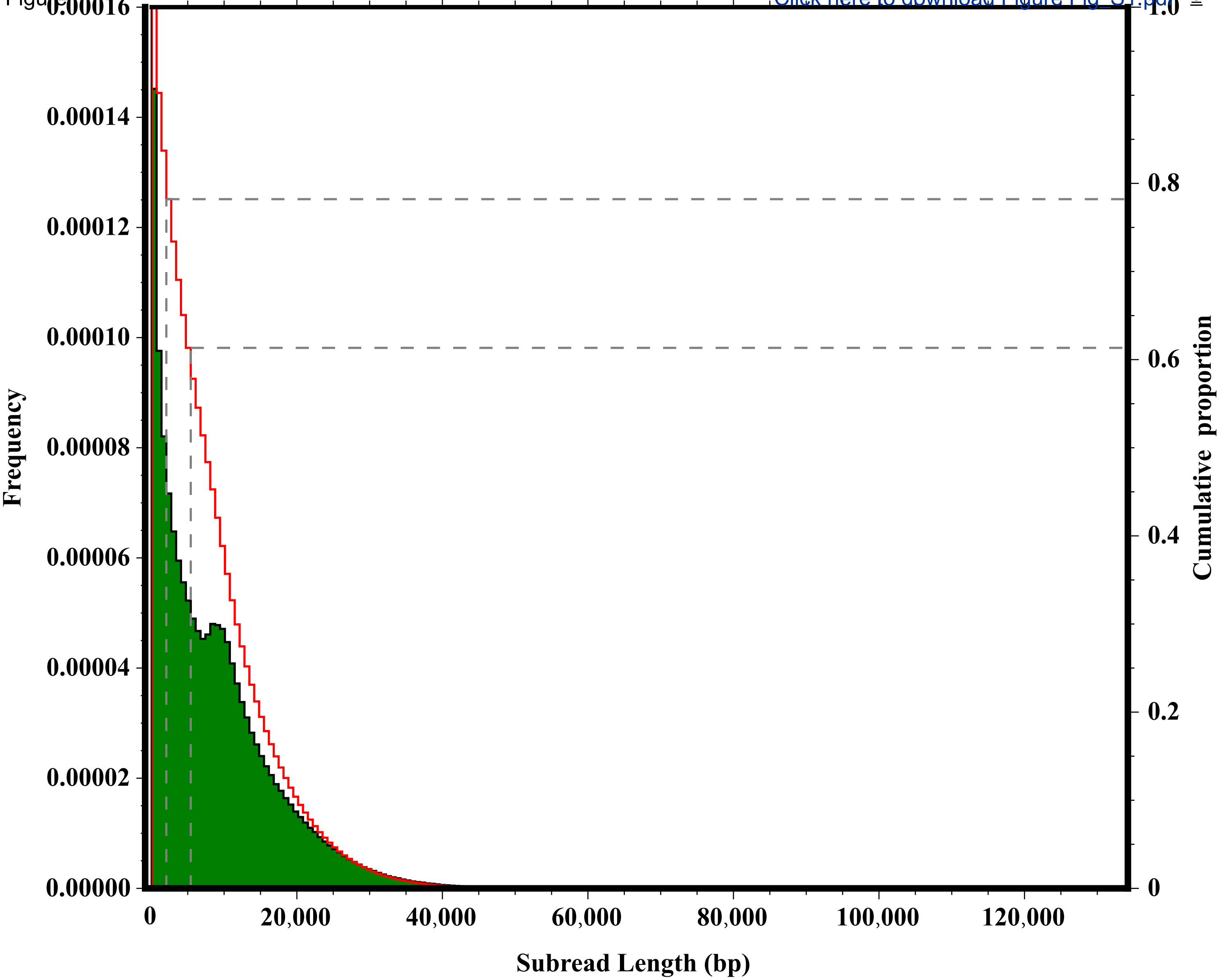

Figure

Click here to download Figure Fig_S2.pdf

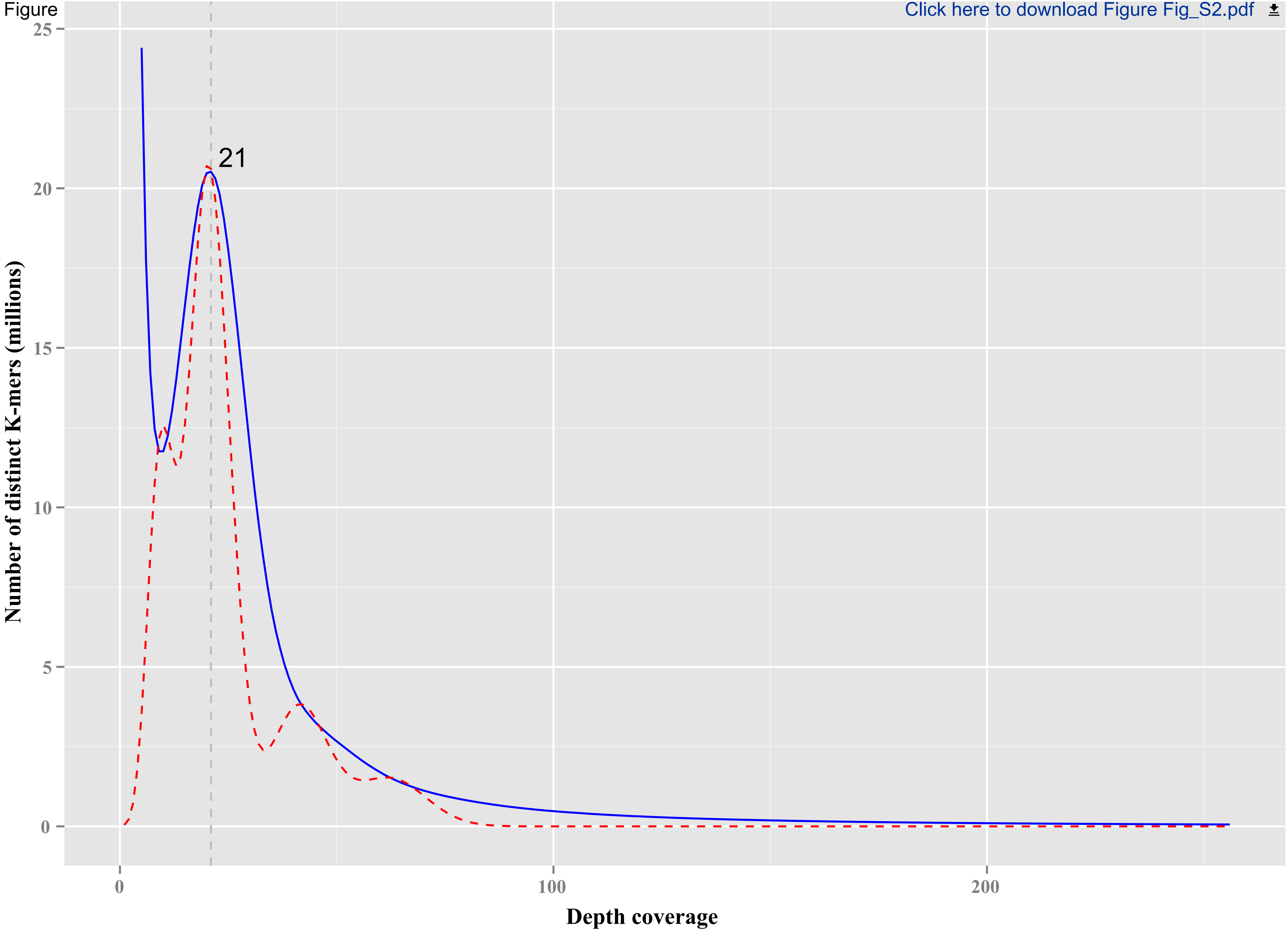

Figure

Click here to download Figure Fig_S3.pdf

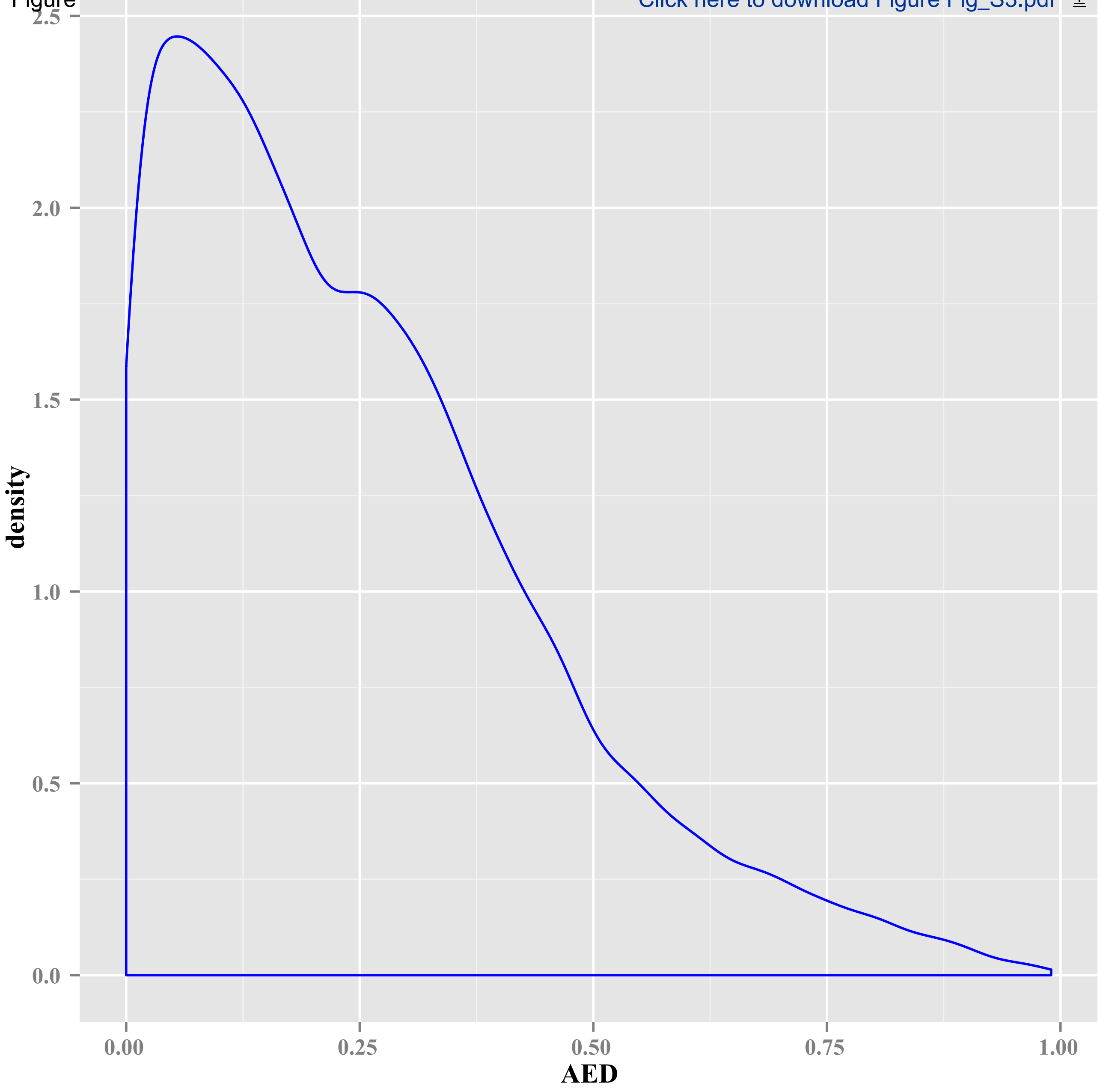

Figure

Click here to download Figure Fig_S4.pdf

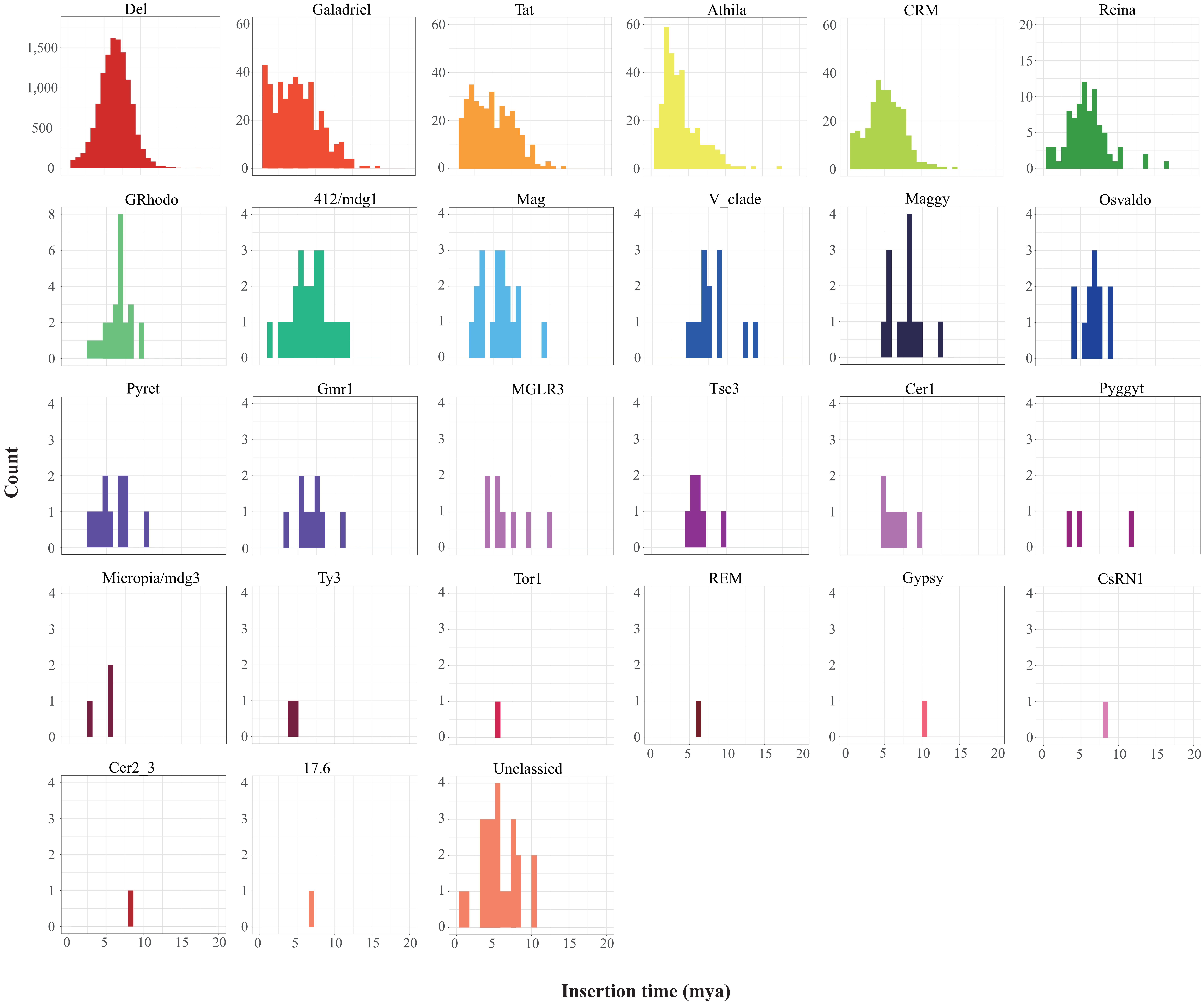

Figure

Click here to download Figure Fig_S5.pdf

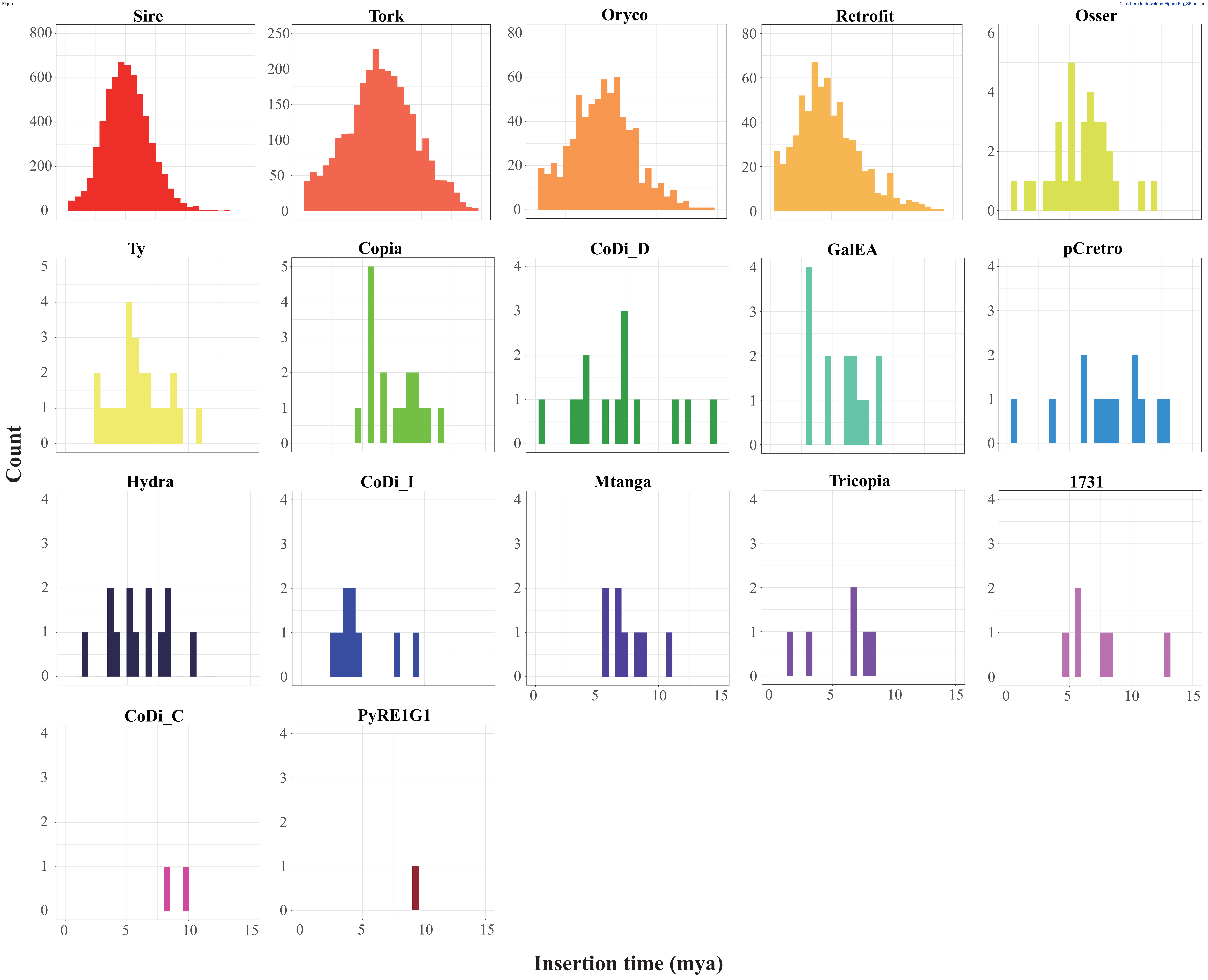

Figure

Click here to download Figure Fig_S6.pdf

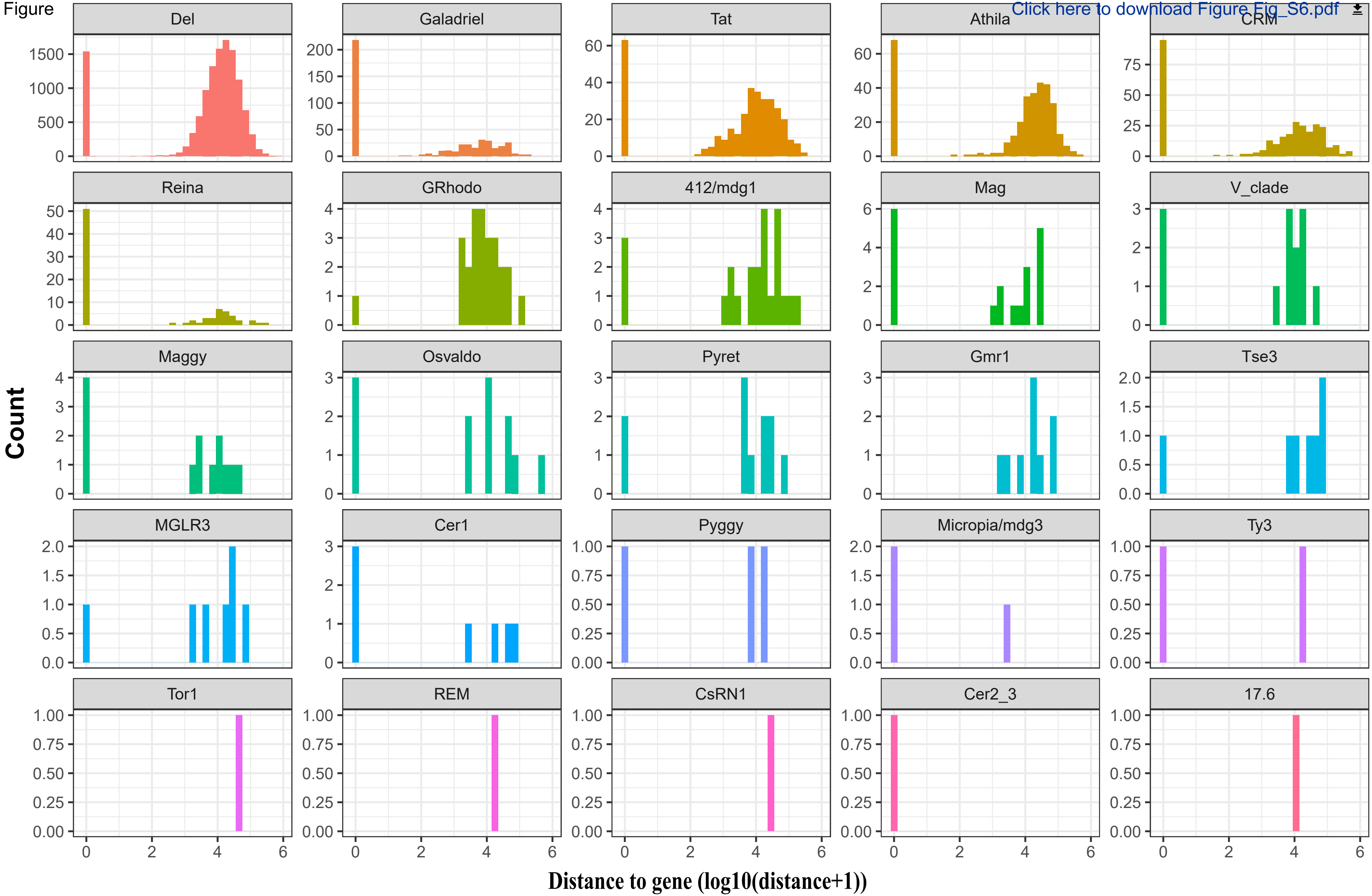

Figure

Click here to download Figure Fig_S7.pdf

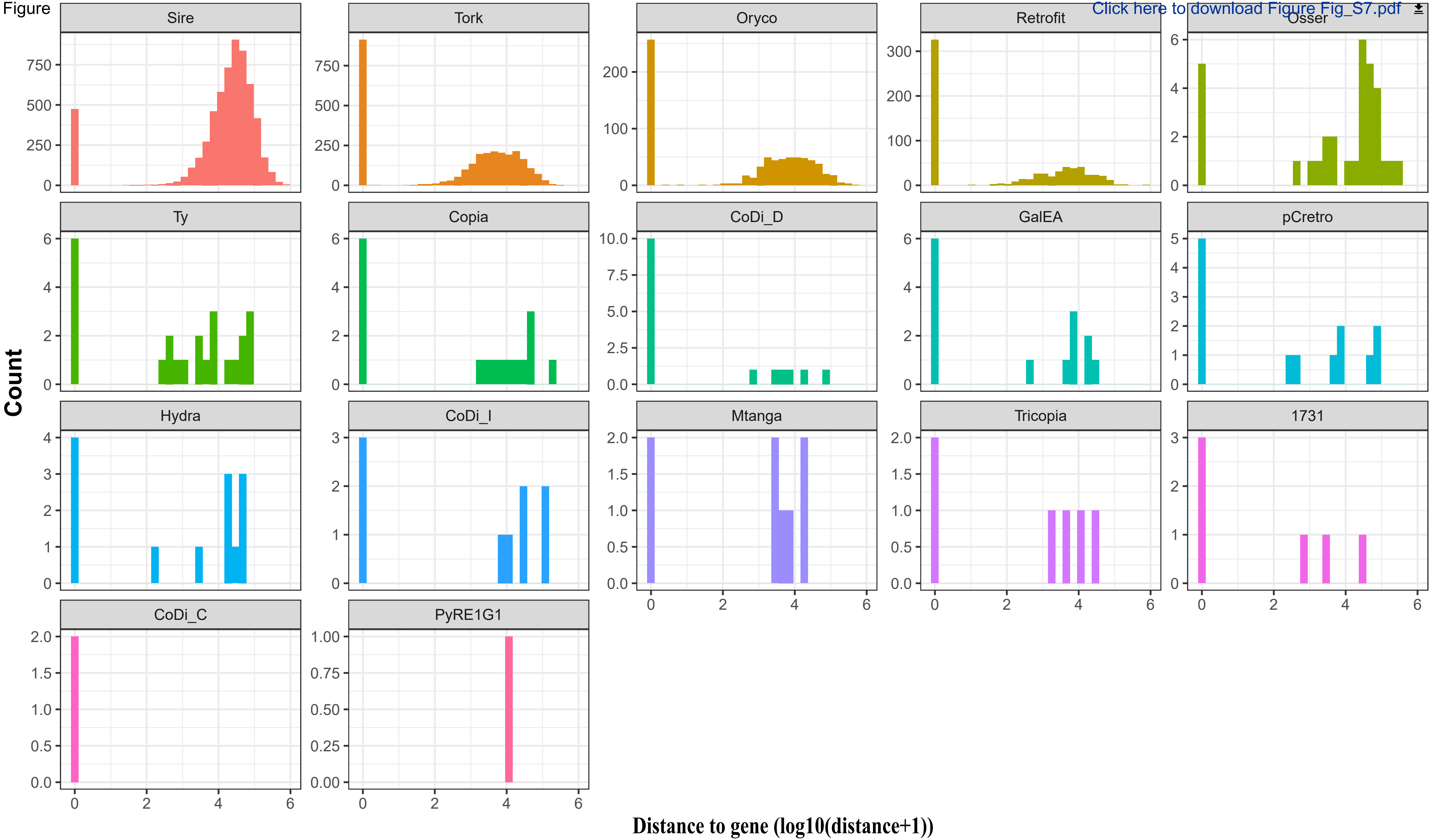

Figure

Click here to download Figure Fig_S8.png

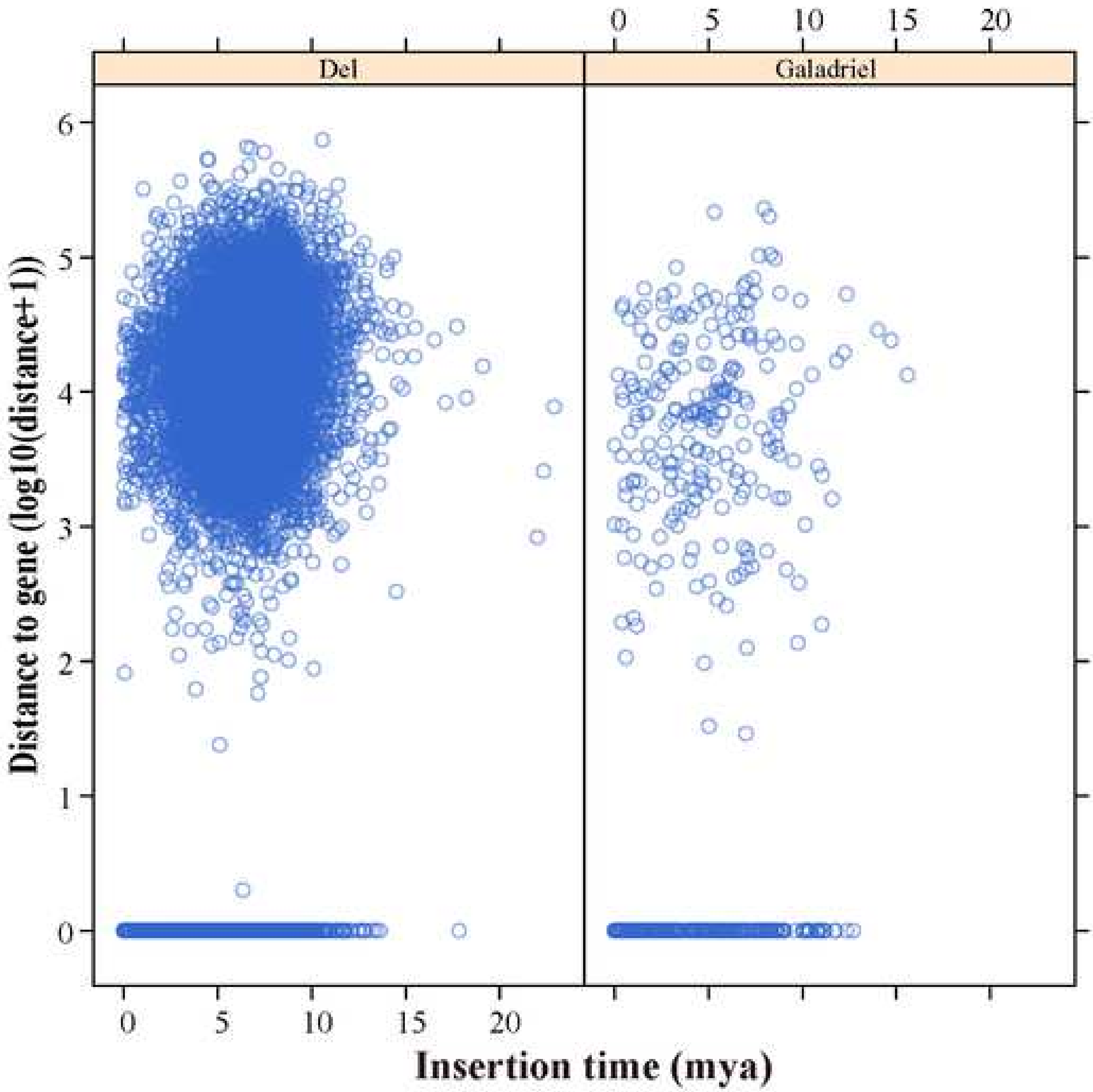

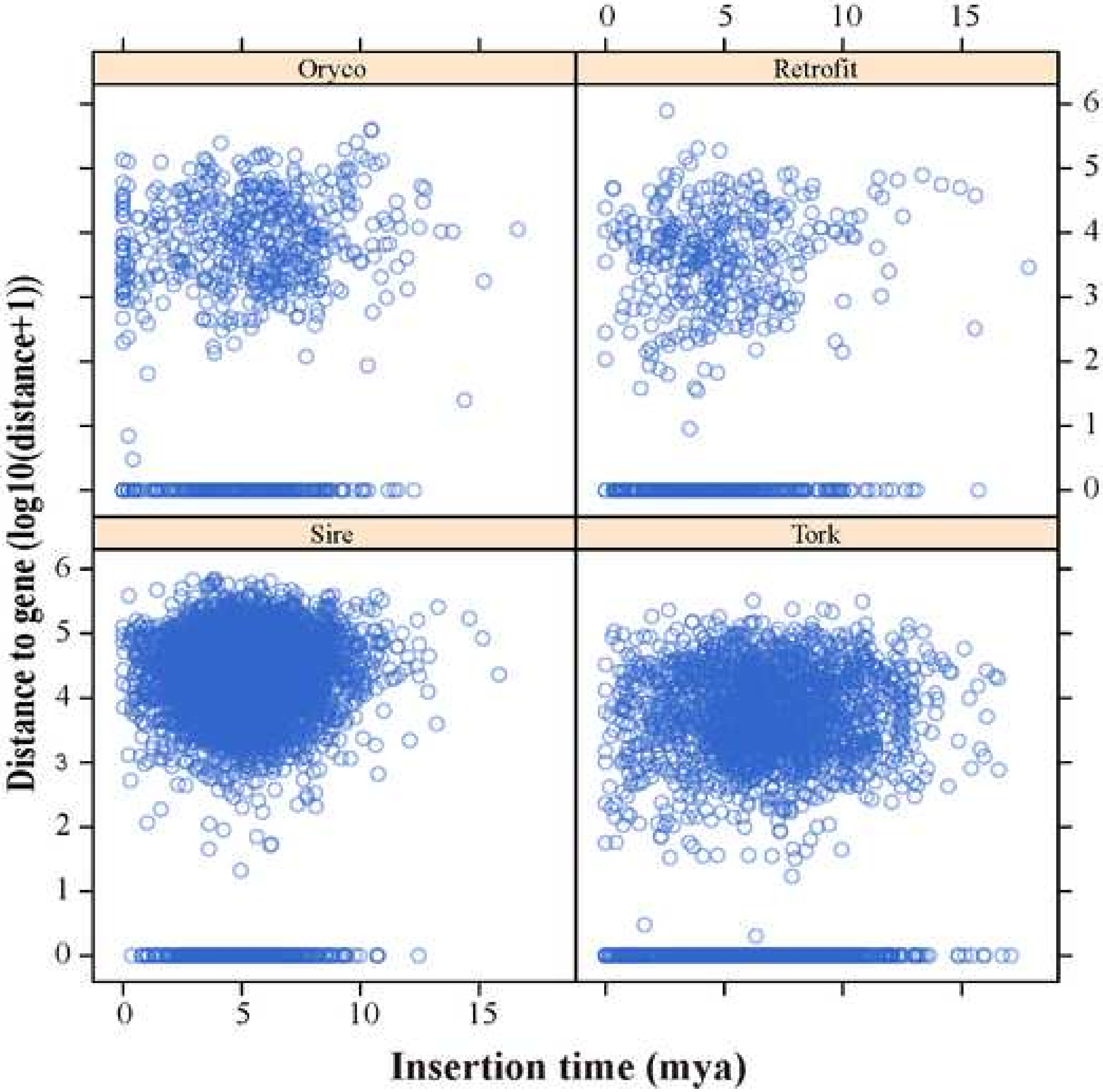
Oryco
Retrofit
Sire
Tork
Distance to gene (log10(distance+1))
Insertion time (mya)
0
5
10
15
0
1
2
3
4
5
6

Figure

Click here to download Figure Fig_S10.pdf

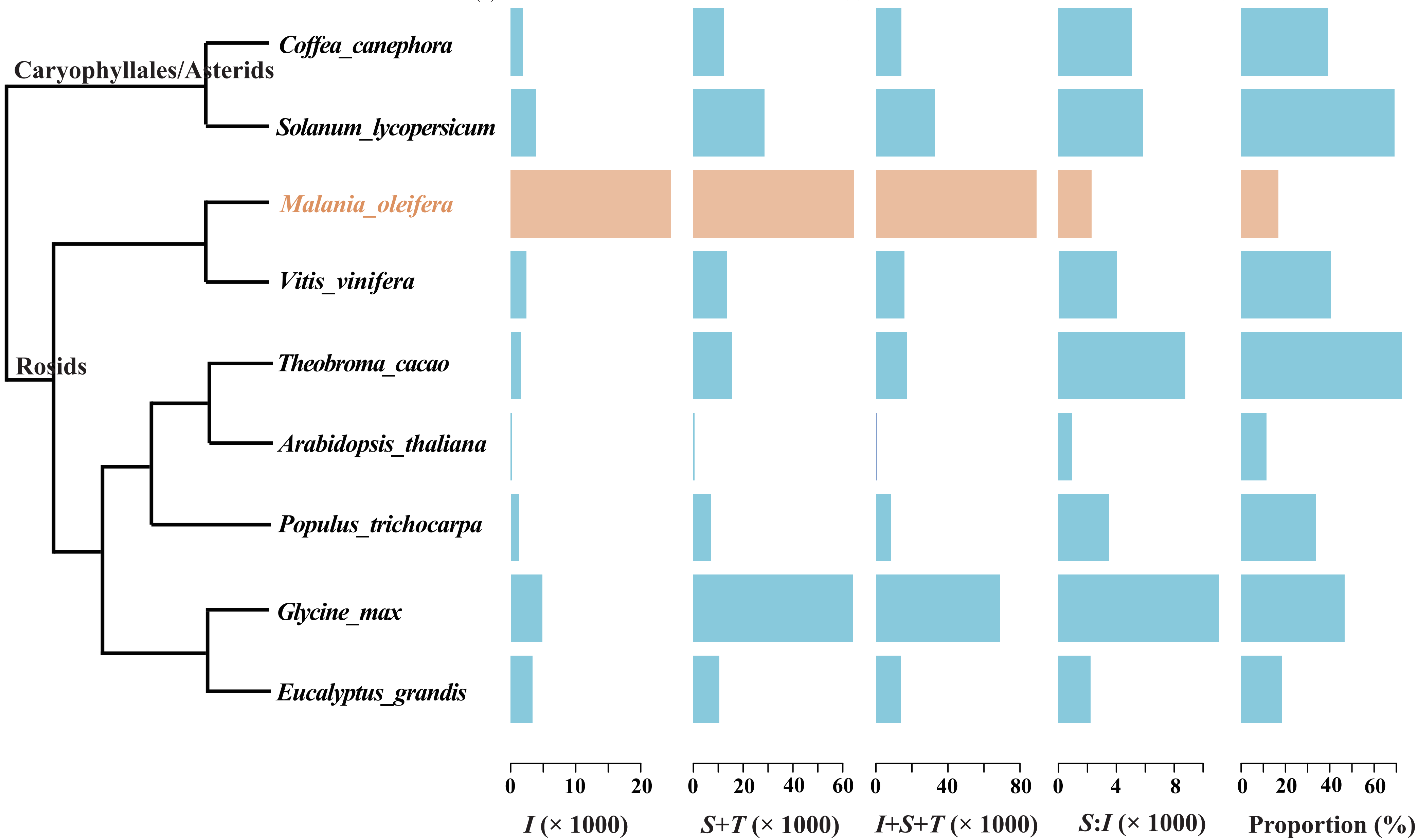

Figure

Click here to download Figure Fig_S11.pdf

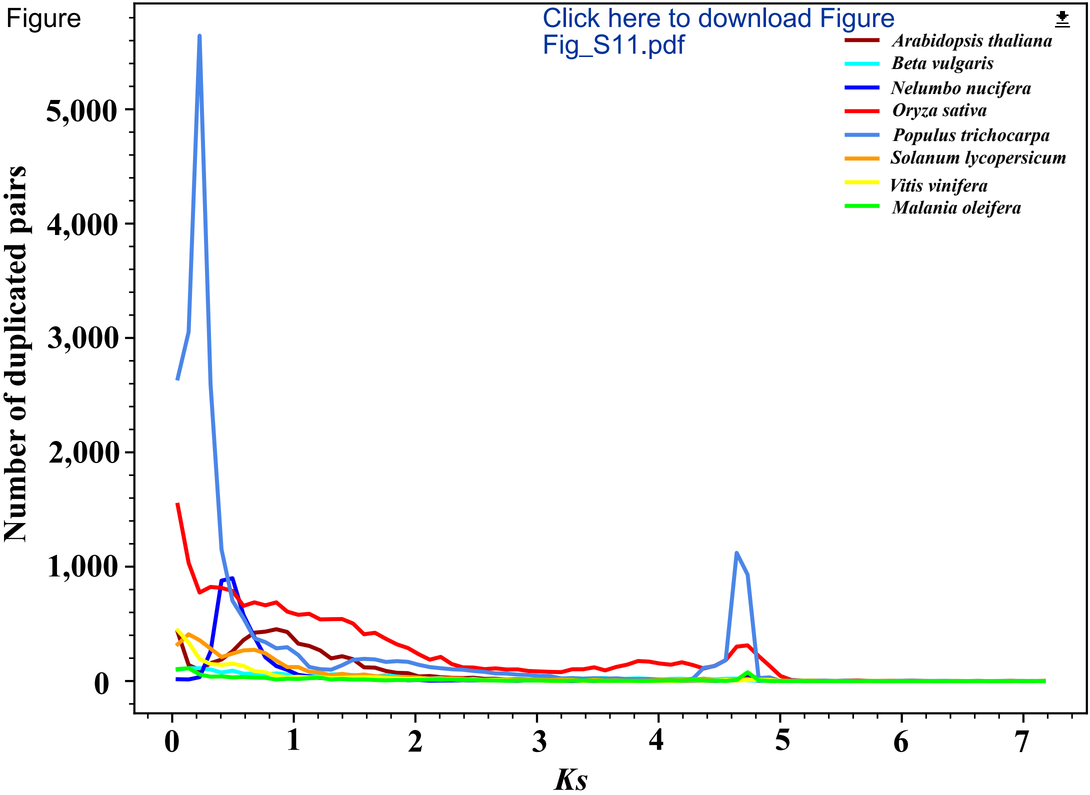

Figure

Click here to download Figure Fig_S12.pdf

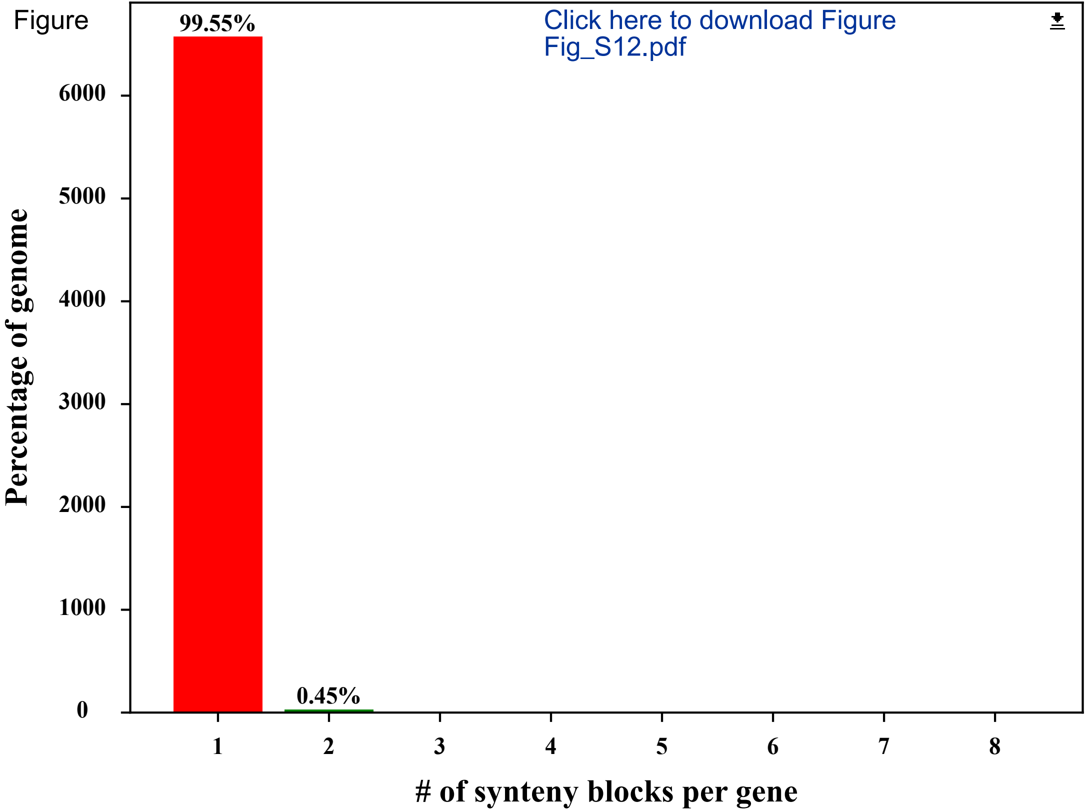

Figure

Click here to download

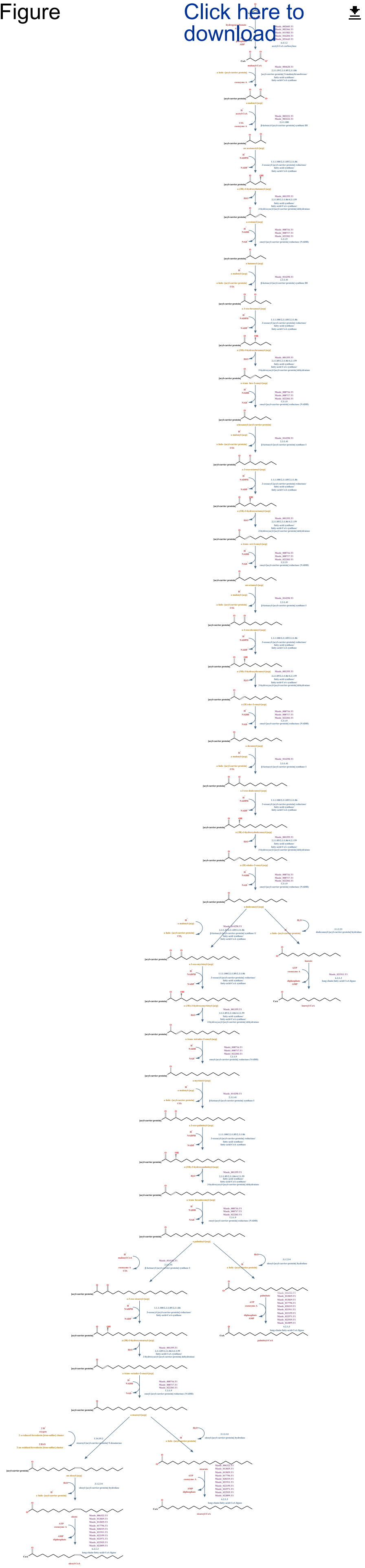

Click here to access/download
Supplementary Material
Supplementary_File_1.docx
