# Supplementary material for: Genome sequence of Malania oleifera, a tree with great value for nervonic acid production
Source: Gigascience. 2019 Jan 24;8(2):giy164. doi: 10.1093/gigascience/giy164 (PMC6377399; doi:10.1093/gigascience/giy164)
Supplement: GIGA-D-18-00301_Revision_1.pdf [file giy164_giga-d-18-00301_revision_1.pdf]

GigaScience

Genome sequence of Malania oleifera, a tree with great value for nervonic acid production

--Manuscript Draft--

**Manuscript Number:**	GIGA-D-18-00301R1
**Full Title:**	Genome sequence of Malania oleifera, a tree with great value for nervonic acid production
**Article Type:**	Data Note
**Funding Information:**	Fundamental Research Funds for the Central Universities (YX2013-41) — Mr. Jian-Feng Mao<br>the construction of the workstation for Academician Bennetzen (2015AC018) — Mr. Fu Chen<br>Yunnan Provincial Science and Technology Department (CN) (2015BB018) — Mr. Sihai Wang<br>the State Key Laboratory of Phytochemistry and Plant Resources in West China (P2015-KF11) — Mr. Sihai Wang
**Abstract:**	Background: Malania oleifera, a member of the Olacaceae family, is an IUCN Red Listed tree, endemic and restricted to the Karst region of southwest China. This tree's seed is valued for its high content of precious fatty acids (especially nervonic acid). However, studies on its genetic make-up, and fatty acid biogenesis are severely hampered by a lack of molecular and genetic tools.<br>Findings: We generated 51 Gigabases (Gb) and 135 Gb of raw DNA sequences, using PacBio Single-Molecule Real-Time (SMRT) and 10x Genomics sequencing, respectively. A final genome assembly, with a scaffold N50 size of 4.65 Megabases (Mb) and a total length of 1.51 Gb, was obtained by primary assembly based on PacBio long reads plus scaffolding with 10x Genomics reads. Identified repeats constituted ~82% of the genome, and 24,064 protein-coding genes were predicted with high support. The genome has low heterozygosity and shows no evidence for recent whole genome duplication. Metabolic pathway genes relating to the accumulation of long chain fatty acid were identified and studied in detail.<br>Conclusions: Here, we provide the first genome assembly and gene annotation for M. oleifera. The availability of these resources will be of great importance for conservation biology, and for the functional genomics of nervonic acid biosynthesis.
**Corresponding Author:**	Jian-Feng Mao, Ph.D.<br>Beijing Forestry University<br>Beijing, CHINA
**Corresponding Author Secondary Information:**	
**Corresponding Author's Institution:**	Beijing Forestry University
**Corresponding Author's Secondary Institution:**	
**First Author:**	Chao-Qun Xu
**First Author Secondary Information:**	
**Order of Authors:**	Chao-Qun Xu<br>Hui Liu<br>Shan-Shan Zhou<br>Dongxu Zhang<br>Wei Zhao, Ph.D.

Powered by Editorial Manager® and ProduXion Manager® from Aries Systems Corporation

	Sihai Wang
	Fu Chen
	Yan-Qiang Sun
	Shuai Nie
	Kai-Hua Jia
	Si-Qian Jiao
	Ren-Gang Zhang
	Quan-Zheng Yun
	Wenbin Guan, Ph.D.
	Xuewen Wang, Ph.D.
	Qiong Gao
	Jeffrey L. Bennetzen, Ph.D.
	Fatemeh Maghuly, Ph.D.
	Ilga Porth, Ph.D.
	Yves Van de Peer, Ph.D.
	Xiao-Ru Wang, Ph.D.
	Yongpeng Ma, Ph.D.
	Jian-Feng Mao, Ph.D.
**Order of Authors Secondary Information:**	

Response to Reviewers:

Scott Edmunds, Editor
GigaScience

Dear Dr. Edmunds,

RE: ID: GIGA-D-18-00301 - “Genome sequence of Malania oleifera, an endangered tree with great value for nervonic acid production”

Enclosed, please find the revised version of the above-mentioned MS. We would like to start with showing our great appreciation for the helpful and constructive comments and suggestions from you and the reviewers. These comments were helpful in improving and clarifying many parts of the manuscript.

##
Comments from Scott Edmunds, Editor
1. More methodological detail (software version numbers, and we strongly recommend including protocols in protocols.io: https://www.protocols.io/groups/gigascience-journal, or reusing or adapting relevant protocols there).
Response: We now provide detailed information on all commands and parameter settings for genome assembly, quality assessment of genome assembly, transcriptome assembly from RNA-seq, repeat and gene annotation, ortholog identification, phylogenetic reconstruction and dating (see Supplementary File 1). This file has also been uploaded to protocols.io, with a DOI code: dx.doi.org/10.17504/protocols.io.u7nezme.

2. RRIDs are also useful for the reproducibility of the software: ftp://penguin.genomics.cn/pub/10.5524/RRID/RRIDlist.pdf
Response: Yes, we now provide RRID alongside each software where applicable.

##
Comments from Catherine Jane Nock (Reviewer #1)
Reviewer #1: This manuscript provides the first genome assembly and genomic resources for the threatened tree Malania oleifera. The manuscript provides extensive

Powered by Editorial Manager® and ProduXion Manager® from Aries Systems Corporation

novel information for this species and the order Santalales. The extremely high repeat content and low heterozygosity reported are of particular interest, as is the identification of candidate genes in the VLCFA biosynthesis pathway.

1. Despite the species listing on IUCN Red List as vulnerable, the authors incorrectly refer to the species as 'endangered' in the title, keywords and throughout the manuscript. Needs to be corrected to either 'threatened' or 'vulnerable'.
Response: The reviewer is correct, and in our revised version, we have now replaced ‘endangered’ with ‘vulnerable’. The revised title now reads “Genome sequence of Malania oleifera, a tree with great value for nervonic acid production”.

2. Substantial supplementary data is provided with this manuscript, but there is very limited detail beyond which programs were used for the de novo assembly pipelines. More information on the parameters applied is needed, at least for the v0.7 assembly used for scaffolding. In general, limited detail is provided on the analysis methods used in the study.
Response: We now provide detailed information on all commands and parameter settings used for genome assembly, quality assessment of genome assembly, transcriptome assembly, repeat and gene annotation, ortholog identification and phylogenetic reconstruction and dating. All information is provided both as a supplementary file (Supplementary File 1), and is uploaded to protocols.io under DOI code: dx.doi.org/10.17504/protocols.io.u7nezme.

3. A major concern is the phylogenetic analysis and molecular dating based on single copy genes. Details of the alignment and analysis methods and results are not provided. A strict molecular clock is applied to the concatenated alignment. While there is no report of testing for clocklike evolution, previous evidence suggests that this highly unlikely. Many relationships within the tree are not supported by previous studies but this is not discussed e.g. Vitales (Vitis) + Santalales (Malania) clade, Proteales (Nelumbo) + Ranunculales (Acquilegia) clade rather than successive sister groups diverging after appearance of core eudicots rather than before. What outgroup was used? The >250 mya divergence between Orzya and eudicots is substantially older than previous estimates. What were the clade support values? The divergence dates and relationships inferred are, at best, unreliable.
Responses: We have revisited this part of the analyses, using IQ-Tree for phylogenetic reconstruction and MCMCTREE from PAML for dating. Substitution model selection and molecular clock testing were added. In short, tree topologies remained the same and all branches gain good support from bootstrap analysis, while dating was, in our opinion, improved and is now more accurate. We (did) provide names/IDs of the single copy genes identified for each of the plant genomes analyzed (please see Supplementary File 2), the alignment (please see Supplementary File 3), as well as detailed description of the updated phylogenetic reconstruction, dating and molecular evolution testing, and results (lines 416-456. As stated higher, all commands and parameter settings used are provided both as a supplementary file (Supplementary File 1), and is uploaded to protocols.io under DOI code: dx.doi.org/10.17504/protocols.io.u7nezme.

4. The reported repeat content of the genome (82%) is extremely high and a thorough analysis of the most abundant repeats (LTR-RT, 58.23% of genome) is presented. If most were the result of ancient amplification how can the high number of intact LTR-RT (Fig S10) and low removal rates and be explained?
Responses: Well, the fact that there are still many intact LTR-RTs from ancient amplification is exactly the result of low removal rates. We do not think there is a contradiction. The mechanism(s) underlying the both the low recent accumulation of LTR-RT amplifications and the low rate of DNA removal are unknown in this study (and are unknown in all other published studies). We can only make the observation at this time, and hope this encourages future studies to investigate this question.

Minor Comments
5. Sentence at Line 90-94 is too long and difficult to interpret. Distinction between effects in healthy individuals and those with a genetic defect is unclear. Are the authors suggesting a link between demyelination and obesity?
Responses: In the revised version of our manuscript we have changed this sentence to: “Nervonic acid is an important component in myelin biosynthesis in the

central and peripheral nervous system. Myelin is generally localized to the sphingomyelin of animal cell membranes [12], where it has been proposed to enhance human brain function. Treatment of myelin disorders may attenuate or prevent various psychotic disorders [13, 14]".

6. Replace 'Chun et S. Lee' with 'Chun & SK Lee'. Author citation for the species probably unnecessary in abstract.
Responses: Done.

7. Line 91: 'nerve system' correct to nervous system
Responses: Done.

8. Line 104-105: clarify what is meant by 'species' endangering mechanism'
Responses: We replaced "species' endangering mechanism" with "provide valuable tools for the genomic dissection of the species' genetic diversity and its population demography for future conservation purposes". Please see lines 103-105.

9. Line179: report k-mer value of 17 in addition to depth
Responses: Done. Please see line 177.

10. Line 201-202: '71.80% gene completeness as determined by BUSCO assessment (Table S4)' comes before the description of gene annotation methods.
Responses: Yes. BUSCO assessment is a mean we used to assess the quality of different versions of genome assembly, it is not part of gene annotation.

11. Numbering is inconsistent, e.g. Line 141 '51,149,552,938 bases', Line 244 '313.36 million reads'. Suggest using million/billion or Mb/Gb throughout
Responses: "51,149,552,938 bases" was changed to "51.15 Gb", see line 140.

##
Comments from Stephen Tsui (Reviewer #2)
Reviewer #2: This article described the genome sequencing of Malania oleifera Chun et S. Lee, a member of the Olacaceae family. A lot of work has been done but more efforts should be paid to highlight the importance of this genome sequence and related resources. The following comments should be helpful for the revision of this piece of work.

1. The quality of the genome is in general high. However, the gene number (24,094) is a bit less than expected because many plants have 40,000 to 60,000 genes. It is not known whether this is related to the parameters used for the gene prediction. Explanation / support about the gene number should be provided.
Responses: Gene numbers in plant genomes differ widely. Most plant genomes for which 40,000 to 60,000 genes have been reported are either hugely inflated (because they still contain several thousands of TEs), or are from polyploids, or are from paleopolyploids, i.e. genomes that have undergone ancient whole genome duplications. Plant genomes for which no or very few polyploidy events have been reported, usually have genomes encoding about 23,000 to 25,000 genes, so the gene content of Malania, for which there is no evidence for recent or ancient genome duplications, is well within expectations.

2. Quite a lot of paragraphs discuss the LTR-RT in the plant. However, relatively less efforts were put on the metabolic uniqueness of this plant. What make this plant to be so important for genome sequencing? The authors should try to explore those unique features of this plant through the genome they generated. Otherwise, the genome data has not been fully utilized.
Responses: Much effort went into discussing LTR-RT, because these make up the majority of the genome sequence. Dynamics of LTR-RT accumulation and affinity of LTR-RT to genes is potentially important for further functional characterization, given the significant roles of functional innovation revealed for LTR TE. However, the significant accumulation of nervonic acid in the seeds, is what makes Malania an important species to sequence because of its metabolic uniqueness and potential. We have tried to stress that as well.

3. The title has mentioned that Malania oleifera has great value for nervonic acid

	production. However, it is surprising that the results about the production of nervonic acid has not been presented in the main text after the introduction.<br>## Responses: Describing the genes involved in the production of nervonic acid was for sure one of our main objectives for this paper. However, without additional molecular genetic experiments (e.g. knockouts of important biosynthetic steps) and proper biochemical product analyses, we cannot identify the rate limiting steps of nervonic acid production in Malania. Therefore, such analysis is beyond the scope of this current work.<br><br>4. In this study, many genes related to the VLCFA biosynthesis were identified. Since the transcriptomes of different parts of the plant had been determined, I am curious to know whether these genes are differentially expressed in different body parts and how the expression patterns correlates the VLCFA distribution in the plan.<br>## Responses: We agree with the reviewer that gene expression will for sure provide valuable information on genes or the gene regulatory network related to the VLCFA biosynthesis. However, the focus of the study presented here was on genome assembly and genome annotation, and the RNA-seq data that were generated were primarily used to support gene prediction and annotation. Indeed, the current RNA-seq data generated in the present study are suited for gene annotation, but not very suitable for reliable gene expression analyses, which requires a much more elaborate experimental design. Consequently, these generated RNA-seq data cannot be used in proper gene expression analyses. We believe that the exceptional quality and detail of the genome assembly and TE analysis justify publication. Hopefully, when future funding can be secured (being able to publish the assembly and annotation should increase our chances ...), we hope to be able to perform adequate transcriptome analyses to further study and unravel VLCFA biosynthesis.<br><br>Finally, we hope that you will find the revised version meeting the journal standards,<br><br>Sincerely Yours,<br>Jian-Feng Mao
**Additional Information:**	
**Question**	**Response**
Are you submitting this manuscript to a special series or article collection?	No
**Experimental design and statistics**<br><br>Full details of the experimental design and statistical methods used should be given in the Methods section, as detailed in our Minimum Standards Reporting Checklist. Information essential to interpreting the data presented should be made available in the figure legends.<br><br>Have you included all the information requested in your manuscript?	Yes
**Resources**<br><br>A description of all resources used, including antibodies, cell lines, animals and software tools, with enough	Yes

information to allow them to be uniquely identified, should be included in the Methods section. Authors are strongly encouraged to cite Research Resource Identifiers (RRIDs) for antibodies, model organisms and tools, where possible.<br><br>Have you included the information requested as detailed in our Minimum Standards Reporting Checklist?	
**Availability of data and materials**<br><br>All datasets and code on which the conclusions of the paper rely must be either included in your submission or deposited in publicly available repositories (where available and ethically appropriate), referencing such data using a unique identifier in the references and in the "Availability of Data and Materials" section of your manuscript.<br><br>Have you have met the above requirement as detailed in our Minimum Standards Reporting Checklist?	Yes

Manuscript
Click here to access/download;Manuscript;Malania_genome_mao11_xu2.do
Click here to view linked References

Genome sequence of *Malania oleifera*, a tree with great value for nervonic acid production

Chao-Qun Xu[1‡], Hui Liu[1‡], Shan-Shan Zhou[1‡], Dong-Xu Zhang[2], Wei Zhao[1], Sihai Wang[3], Fu Chen[4], Yan-Qiang Sun[1], Shuai Nie[1], Kai-Hua Jia[1], Si-Qian Jiao[1], Ren-Gang Zhang[5], Quan-Zheng Yun[5], Wenbin Guan[1], Xuewen Wang[4,6], Qiong Gao[1], Jeffrey L. Bennetzen[4,6], Fatemeh Maghuly[7], Ilga Porth[8,9,10], Yves Van de Peer[11,12,13], Xiao-Ru Wang[1,14], Yongpeng Ma[15]*, Jian-Feng Mao[1]*

[1] Beijing Advanced Innovation Center for Tree Breeding by Molecular Design, National Engineering Laboratory for Tree Breeding, School of Nature Conservation, College of Biological Sciences and Technology, Beijing Forestry University, Beijing, 100083, China.

[2] College of Life Science, Datong University, Datong, 037009, Shanxi, China.

[3] Yunnan Key Laboratory of Forest Plant Cultivation and Utilization, State Forestry Administration Key Laboratory of Yunnan Rare and Endangered Species Conservation and Propagation, Yunnan Academy of Forestry, Kunming, 650201, Yunnan, China.

[4] The Camellia Institute, Yunnan Academy of Forestry, Guangnan, 663300, Yunnan, China.

[5] Beijing Ori-Gene Science and Technology Co. Ltd, Beijing, 102206, China.

[6] Department of Genetics, University of Georgia, Athens, GA 30602, USA

[7] Plant Biotechnology Unit (PBU), Dept. Biotechnology, BOKU-VIBT, University of Natural Resources and Life Sciences, Muthgasse 18, 1190 Vienna, Austria.

[8] Département des sciences du bois et de la forêt, 1030, Avenue de la Médecine, Université Laval, Québec (Québec) G1V 0A6, Canada.

[9] Institute for System and Integrated Biology, Pavillon Charles-Eugène-Marchand, 1030, Avenue de la Médecine, Université Laval, Québec (Québec) G1V 0A6, Canada.

[10] Centre d'Étude de la Forêt, 1030, Avenue de la Médecine, Université Laval, Québec (Québec) G1V 0A6, Canada.

[11] Department of Plant Biotechnology and Bioinformatics, Ghent University, 9052 Ghent, Belgium

[12] VIB Center for Plant Systems Biology, 9052 Ghent, Belgium

[13] Centre for Microbial Ecology and Genomics, Department of Biochemistry, Genetics and Microbiology Genetics, University of Pretoria, Private bag X20, Pretoria 0028, South Africa

[14] Department of Ecology and Environmental Science, UPSC, Umeå University, SE-901 87 Umeå, Sweden.

[15] Yunnan Key Laboratory for Integrative Conservation of Plant Species with Extremely Small Population, Kunming Institute of Botany, Chinese Academy of Sciences, Kunming, 650201, China.

ORCIDs: Jeffrey Bennetzen: 0000-0003-1762-8307; Fatemeh Maghuly; 0000-0001-5433-0070; Ilga Porth: 0000-0002-9344-6348; Xiao-Ru Wang: 0000-0002-6150-7046; Yves Van de Peer: 0000-0003-4327-3730; Jian-Feng Mao:

0000-0001-9735-8516

‡These authors contributed equally to this paper.

*Correspondence to: mayongpeng@mail.kib.ac.cn (YPM); jianfeng.mao@bjfu.edu.cn (JFM)

Abstract

Background: *Malania oleifera*, a member of the Olacaceae family, is an IUCN Red Listed tree, endemic and restricted to the Karst region of South West China. This tree's seed is valued for its high content of precious fatty acids (especially nervonic acid). However, studies on its genetic make-up, and fatty acid biogenesis are severely hampered by a lack of molecular and genetic tools.

Findings: We generated 51 Gigabases (Gb) and 135 Gb of raw DNA sequences, using PacBio Single-Molecule Real-Time (SMRT) and 10x Genomics sequencing, respectively. A final genome assembly, with a scaffold N50 size of 4.65 Megabases (Mb) and a total length of 1.51 Gb, was obtained by primary assembly based on PacBio long reads plus scaffolding with 10x Genomics reads. Identified repeats constituted ~82% of the genome, and 24,064 protein-coding genes were predicted with high support. The genome has low heterozygosity and shows no evidence for recent whole genome duplication. Metabolic pathway genes relating to the accumulation of long chain fatty acid were identified and studied in detail.

Conclusions: Here, we provide the first genome assembly and gene annotation for *M. oleifera*. The availability of these resources will be of great importance for conservation biology, and for the functional genomics of nervonic acid biosynthesis.

Keywords: *de novo* genome assembly, vulnerable plant, *Malania*, nervonic acid, transcriptomes

DATA DESCRIPTION

Background information

Malania oleifera Chun & SK Lee (NCBI:txid397392), a 10-20 m high tree (**Fig. 1a-d**), is from the monotypic genus *Malania* of the Olacaceae family [1]. This tree is endemic to a restricted area within the Karst topography of southwest Guangxi and southeast Yunnan provinces, China. The recorded distribution range is bounded by N23°23′ - N24°28′ in latitude and E105°30′ - E107°30′ in longitude (**Fig. 1e**). This tree is called “garlic-fruit tree” or “suantouguo” (蒜头果) by local communities, due to its garlic shaped fruits. As an endemic tree and because of its natural populations being much reduced because of ongoing logging and habitat clearance, this tree species has been listed in the IUCN Red List as “Vulnerable B1+2c” (extent of occurrence estimated to be < 20,000 km^2 and a continuing decline, observed, projected, or inferred, in numbers of mature individuals) [2], and has been assigned as a plant species with an extremely small population size (PSESP) for urgent conservation action [3]. Different mechanisms that could explain why *M. oleifera* became a vulnerable species have been proposed, such as niche specialization [4], limited germination and regeneration [5, 6], or pollination/mating system [7], as well as the biology of its pathogens [8]. However, until now, apart from a recent

chloroplast genome sequence [9], only a few molecular genetic resources are available for *M. oleifera* to investigate its population structure and genetic makeup.

Besides conservation urgency, *M. oleifera* is also notable for its substantial phytochemical and phytopharmaceutical value: its seed has very high (64.5%) oil content [10, 11], and the highest-known proportion (55.70-67%) of nervonic acid ($C_{24}H_{46}O_2$, PubChem CID: 5281120). Nervonic acid is an important component in myelin biosynthesis in the central and peripheral nervous system. Myelin is generally localized to the sphingomyelin of animal cell membranes [12], where it has been proposed to enhance human brain function. Treatment of myelin disorders may attenuate or prevent various psychotic disorders [13, 14]. *M. oleifera* produces essential oils with benzyl alcohol (58.42%) and benzaldehyde (29.66%) as the main constituents as well as benzoic acid (1.49%) [10]. *M. oleifera* seeds also produce the glycoprotein *malania* which has high cytotoxic activity towards tumor cells and is one of the most potent toxins of plant origin [15]. Yet, little is known about the molecular mechanisms underlying the metabolic biosynthesis processes of these promising compounds in *M. oleifera*.

Here, we present a high-quality genome assembly for *M. oleifera*, combining PacBio single molecule long-reads and 10x Genomics linked reads. The assembled genome, its structural and functional annotation and in-depth characterization will provide valuable tools for the genomic dissection of the species' genetic diversity and its population demography for future conservation purposes, as well as for in-depth molecular knowledge regarding biosynthesis and regulation of metabolism to promote

the efficient and sustainable exploitation of this precious biological resource.

Plant material

One mature and healthy tree with abundant fruit (**Fig. 1 a, b, c, d**) was chosen as a tissue source for whole genome sequencing. The selected tree measured ~18 m in height, ~35 cm in diameter (at breast height) and is believed to be ~50 years old. This tree is located within a natural stand close to Diji Village, Jiumoxiang, Guangnan County, Yunnan Province, China (N23.90^o latitude, E104.90^o longitude, 1,402 m elevation) (**Fig. 1e**). The stand, from which the samples were taken, experienced little anthropogenic intervention and consists of trees of the same species but of different ages. Fresh leaves were sampled in September of 2017.

For RNA sequencing, leaves, fruits and seeds were sampled from healthy, high-yielding, mature trees from Funing County, Yunnan province and Leye County, Guangxi province, China, in different seasons during the years 2013-2016 (**Fig. 1e** and **Table S1**). Samples were immediately flash frozen in liquid nitrogen upon collection and transported on dry ice to Beijing Forestry University (BFU) for sequence analysis.

All samples were collected with permission from and under the supervision of local forestry bureaus. See **Table S1** and **Fig. 1** for more details.

PacBio SMRT sequencing

High-quality and high-molecular-weight genomic DNA was extracted from leaves of

the selected tree, following the "~20 kb SMRTbell™ Libraries" protocol [16]. DNA was purified using the Mobio PowerClean® Pro DNA Clean-Up Kit, and its quality was assessed by standard agarose gel electrophoresis and Thermo Fisher Scientific Qubit Fluorometry. Genomic DNA was sheared to a size range of 15-50 Kb using either AMPure beads (Beckman Coulte) or g-TUBE (Covaris), and enzymatically repaired and converted into SMRTbell template libraries according to Pacific Biosciences instructions. Following this procedure, hairpin adapters were ligated after exonuclease-based digestion (of the remaining damaged DNA fragments and those fragments without adapters at both ends). The resulting SMRTbell templates were subsequently size-selected by Blue Pippin electrophoresis (Sage Sciences). Templates ranging from 15 to 50 Kb were sequenced on a PacBio Sequel instrument using S/P2-C2 sequencing chemistry (10 SMRT cells). A total of 5,778,035 PacBio long reads were generated, yielding a total of 51.15 Gb (roughly 30x coverage of the assembled genome) of single-molecule sequencing data with an average read length of 8,852 bp (**Fig. S1** and **Table S1**).

10x Genomics library preparation and Illumina sequencing

Purified high-molecular-weight genomic DNA of high quality was incubated with Proteinase K and RNaseA for 30 min at 25 °C. DNA was further purified, indexed and partitioned into barcoded libraries that were prepared using the GemCode kit (10x Genomics, Pleasanton, CA). Following the GemCode procedure, 1.0 ng of DNA was used for GEM (Gel Beads in Emulsion) reactions in which DNA

fragments were partitioned into molecular reactors to extend the DNA and to introduce specific 14-bp partition barcodes. Subsequently, GEM reactions were PCR-amplified. The PCR cycling protocol was: 95 °C for 5 min; cycled 18x: 4 °C for 30 s, 45 °C for 1 s, 70 °C for 20 s, and 98 °C for 30 s; held at 4 °C. The PCR products were purified as described in the GemCode protocol. Purified DNA was sheared, end-repaired, adenylation tailed, universal adapter ligated and samples indexed according to the manufacturer's recommendations.

The whole genome GemCode library was sequenced using 2x150 paired-end (PE) sequencing on Illumina HiSeq X Ten. A total of 899.778 million reads (~134.97 Gb, roughly 89x coverage of the assembled genome) were obtained, of which 89.1% had base quality values over 20 and 80% over 30 (**Table S1**). There were 19,319,151 (99.98% of total read pairs) indexes assigned to more than one read pair, while 27,368 (9.55%), 830 (2.12%) and 450 (1.80%) had more than 1000, 3000, or 5000 read pairs, respectively (**Table S2**). Sequence data were analyzed using the GemCode Long Ranger Software Suite [17, 18].

RNA sequencing

Frozen tissues were ground with a mortar and pestle, and RNA was isolated using the NEBNext Poly (A) mRNA Magnetic Isolation Module. RNA quality was determined on an Agilent 2100 BioAnalyzer. Seven sequencing libraries were prepared using the NEBNext Ultra RNA Library Prep Kit for Illumina. 150/100 bp PE sequencing was performed on an Illumina HiSeq 2000/2500 machine. See **Table S1** for details.

Estimation of genome size, heterozygosity, and repeat content

Canu v1.6 (Canu, RRID:SCR_015880) [19] was employed to filter and correct the PacBio reads. Next, k-mers were counted using Jellyfish (Jellyfish, RRID:SCR_005491) [20]. Finally, gce v1.0.0 [21] was used to estimate genome size, repeat content and the level of heterozygosity. A total of 29,971,959,192 k-mers (size 17) were identified, and the peak k-mer depth obtained was 21 (**Fig. S2**). The genome size was estimated to be ~1.50 Gb (**Table S3**). The final cleaned data corresponded to about 21-fold coverage. Repeat and error frequencies were estimated to be 54.61% and 0.34%, respectively. Heterozygosity was very low (~0.06%). See **Supplementary File 1** for commands and settings.

***De novo* genome assembly and quality control**

First, primary assemblies (eight from PacBio long reads, one from 10x Genomics linked reads) were prepared by different pipelines. Next, scaffolding and polishing were performed on the optimal primary assemblies in order to obtain a final genome assembly. Primary assembly v0.1 was generated from PacBio long reads after correction by Canu v1.6 [19], assembly v0.2 by MECAT v1.1 [22], assembly v0.3 by miniasm v0.2-r168 [23] after alignment by minimap v0.2-r124 [23], assembly v0.4 by Falcon v0.7 (Falcon, RRID: SCR_016089) [24, 25] after correction with Canu v1.6, assembly v0.5 by SMARTdenovo v1.0.0 [26] after correction with Canu v1.6, assembly v0.6 by Wtdbg v1.2.8 [27] after correction with Canu v1.6, assembly v0.7

by SMARTdenovo v1.0.0 after correction, and assembly v0.8 by Wtdbg v1.2.8. Assembly v0.9 was prepared by Supernova™ assembler 2.0 [28, 29] from 10x Genomics linked reads data. Based on quality control parameters, assembly v0.7 was chosen as optimal for further scaffolding and polishing. It generated a reasonably-sized assembly (1.51 Gb), providing the highest N50 (i.e. the shortest sequence length at 50% of the total genome assembly length) (1.12 Mb), and the lowest number of contigs (3,038) and L50 (i.e. the smallest number of contig sequences whose lengths sum produces the N50 value) (330). Furthermore, genome assembly version v0.7 exhibited the longest contig length (6.72 Mb), as well as 71.80% gene completeness as determined by BUSCO (BUSCO, RRID:SCR_015008) [30] assessment (**Table S4**). This assembly (v0.7) was further polished with raw PacBio long reads using arrow v2.2.1 [31] to produce (in two rounds) assembly v1.0. Subsequently, 10x Genomics linked reads were processed with Long Ranger [17, 18], and were then aligned to v1.0 using BWA mem v0.7.15 (default values, -*t*12) (BWA, RRID:SCR_010910) [32] and subsequently scaffolded by ARCS v1.0.1 [33] to produce assembly v1.1. The final assembly was generated after one further iteration of polishing with arrow v2.21 and three iterations with Pilon v1.22 (Pilon, RRID:SCR_014731) [34]. Before arrow-based polishing, PacBio raw reads were aligned using BLASR v5.1 (BLASR, RRID:SCR_000764) [35, 36], and PacBio raw reads were mapped with Bowtie2 v2.2.6 (Bowtie2, RRID:SCR_016368) [37] before each iteration with Pilon. In the final assembly, a genome size of 1.51 Gb was obtained, consisting of 2,987 contigs, 1,277 scaffolds (with contig N50 of 1.22 Mb,

scaffold N50 of 4.65 Mb, longest contig of 6.7 Mb and longest scaffold of 25.1 Mb), and has a gene completeness of 90.60% (**Table 1** and **Table S4**).

The consistency of the predicted genome size based on k-mer characterization and the assembled genome indicated a good quality for our assembly. Furthermore, when all clean Illumina reads were mapped to the final assembly (v1.2f), a high sequence coverage of 98.5% was obtained. In addition, an even higher sequence coverage of 99.32% was observed for mapping PacBio long reads to the final assembly using BLASR. These two coverage values suggested high sequence completeness and fidelity of the genome assembly. Mapping rates (91-98%) were also very high for transcriptomic datasets mapped to the final assembly, of which most (79-96%) were uniquely mapped (**Table S1**), with the exception of one RNA sequencing library (SRA accession: SRR7221534) that yielded low mapping rates (10.31%), a result that we cannot explain by anything aside from microbial or other contamination (**Supplementary File 1** for commands and settings).

Transposable element and other repeat annotation

De novo repeat identification was pursued with RepeatModeler v1.0.10 (RepeatModeler, RRID:SCR_015027) [38], which employs two complementary computational methods (RECON v1.08 and RepeatScout v1.0.5 (RepeatScout, RRID:SCR_014653) [39]) for identifying repeat element boundaries and family relationships from sequence data. Subsequently, the outputs from RepeatModeler and the RepBase library [40] were combined and used for further characterization of

transposable elements (TEs), many of which are not repetitive, and other repeats by homology-based methods, including identification with RepeatMasker (v4.0.7, rmblast-2.2.28) (RepeatMasker, RRID:SCR_012954) [41]. In sum, a high percentage of the genome (82.05%) was predicted to be TEs and/or repeats in the assembled genome, predominantly (65.45%) known TEs, with 11.94% uncharacterized TEs, and a smaller number (3.64%) of simple repeats. Long terminal repeat-retrotransposons (LTR-RTs) represented the highest proportion (58.23%) of the genome, while LINE (3.67%), SINE (0.11%), DNA (3.32%) and RC (0.12%) TEs made up a minor fraction (7.22%) of the genome. *Copia* (29.51% of the genome sequence) and *Gypsy* (28.15%) LTR-RTs were about equally abundant. Repeat annotations are provided in **Fig. 2a** and **Table S5.**

Transcriptome assembly and candidate gene annotation

In total, 313.36 million raw reads from RNA analyses were generated from leaf, seed, and fruit tissues and used for gene annotation (**Table S1**). Illumina raw reads were processed by Trimmomatic v0.33 (Trimmomatic, RRID:SCR_011848) [42] and Cutadapt v1.13 (Cutadapt, RRID:SCR_011841) [43] and aligned to the genome assembly using HiSat2 v2.1.0 (HiSat2, RRID:SCR_015530) [44]. Base quality was assessed with FastQC (FastQC, RRID:SCR_014583) [45] before and after data cleaning. Statistics for the RNA sequencing data are shown in **Table S1**. Reference genome-guided and *de novo* transcriptome assemblies, respectively, were constructed with StringTie v1.3.3b (StringTie, RRID:SCR_016323) [46] and Trinity v2.0.6

(Trinity, RRID:SCR_013048) [47]. Then, transcriptome assemblies were combined and further refined using CD-HIT v4.6 (CD-HIT, RRID:SCR_007105) [48]. Finally, 57,299 unique transcripts were predicted. The summary of transcriptome assemblies is reported in **Table S6**.

For *ab initio* gene prediction, AUGUSTUS v3.2.3 (AUGUSTUS, RRID:SCR_008417) [49, 50] was employed, using model training based on coding sequences from *Arabidopsis thaliana* and 1,440 single copy orthologs from the BUSCO embryophyta_odb9 database. For evidence-based gene prediction, the individual transcripts from RNA sequencing as well as the transcriptome assembly were aligned to the repeat-masked reference genome assembly with BlastN (BLASTN, RRID:SCR_001598) and TblastX (TBLASTX, RRID:SCR_011823) from BLAST v2.2.28+ (NCBI BLAST, RRID:SCR_004870) [51] (E-value cutoff of 10^{-5}), respectively. Protein sequences from *A. thaliana* [52], *Vitis vinifera* [53], *Solanum lycopersicum* [54] and *Olea europaea* [55] were aligned to the TE-masked and repeat-masked reference genome assembly with BlastX (BLASTX, RRID:SCR_001653) (E-value cutoff of 10^{-5}). After optimization with Exonerate v2.4.0 (Exonerate, RRID:SCR_016088) [56, 57], gene model predictions were finalized using the MAKER package v2.31.9 (MAKER, RRID:SCR_005309) [58] within AUGUSTUS. AED (Annotation Edit Distance) scores were calculated for each of the predicted genes as part of the MAKER pipeline to assess the quality of gene prediction. Putative functions for each identified gene were predicted by homology searches with BLAT (BLAT, RRID:SCR_011919) [59] against the UniProt database

(UniProt, RRID:SCR_002380) [60]. Protein annotation against Pfam (Pfam, RRID:SCR_004726) [61, 62] and InterProScan (InterProScan, RRID:SCR_005829) [63] were also conducted using the scripts provided in the MAKER package. The completeness of gene annotation was checked using the BUSCO dataset (i.e. the 1,440 single-copy orthologs from the embryophyta_odb9 database) with 10^{-5} as BLAST E-value cutoff (**Supplementary File 1** for commands and settings).

A total of 24,094 genes were predicted, with average lengths of gene regions, genes (including 5’, 3’ UTRs, exons and introns), CDS and exons, respectively, of 11,809 bp, 1,460 bp, 1,281 bp and 244 bp (**Table S7**). The distribution of AED tagged by MAKER is shown in **Fig. S3**, in which about 83.39% of the annotated genes (20,092 genes) had an AED < 0.5 (**Table S7**), indicating well-supported gene annotation. The result from BUSCO assessment of genome assembly and annotation qualities are shown in **Table S8**. Identification of 92.29% of the universal single-copy genes (1,329 genes out of the total 1,440 genes) supported the high quality of the genome assembly. Among the 1,329 BUSCO conserved single-copy genes detected in the assembled genome, 1,217 (84.51% of the completed genes) were found to be single-copy, while 41 genes (2.85%) were complete and duplicated (**Table S8**).

The predicted genes were annotated using seven functional databases: (1) the NCBI non-redundant protein database (NR) [64], (2) the Swiss-Prot protein database [60, 65], (3) the Translated EMBL-Bank (part of the International Nucleotide Sequence Database Collaboration, TrEMBL) [60, 66], (4) the protein families database (Pfam) [67], (5) the Cluster of Orthologous Groups for eukaryotic complete

genomes (KOG) database [68], (6) the KO (the Kyoto Encyclopedia of Genes and Genomes, Orthology) database (KEGG, RRID:SCR_012773) [69, 70], and (7) the Gene Ontology (GO) database (GO, RRID:SCR_002811) [71, 72]. By this combined strategy, 91.60% of all predicted genes could be annotated with the following protein related database outcomes: NR (57.20%), Swiss-Prot (90.60%), TrEMBL (91.40%), Pfam (76.80%), KOG (87.60%), KO (32.90%), and GO (78.70%) (**Table S9**).

Differential proliferation, age dynamics and gene proximity of different LTR-RT families

LTR-RTs (58.23% of the annotated genome) represent the most abundant group of TEs in the genome of *M. oleifera*. We further examined their classification, age distribution, birth and death. LTRharvest [73] and LTRdigest [74] were used for *de novo* prediction of LTR-RTs. In this workflow, it was required that a candidate LTR-RT was separated by 1 to 15 Kb from other candidates and flanked by a pair of putative LTRs, which could range from 100 to 3,000 bp, but with a similarity >80%. The LTR-RT candidates that possessed complete Gag-Pol protein sequences were retained as intact LTR-RTs (*I*), while solo-LTRs (*S*) and truncated LTRs (*T*), were identified based on sequence similarity to the intact LTR-RTs. LTR homologies were identified by BLASTN analysis [51] with an E-value cutoff of 1e-10, 90% overlap in length and 90% identity. Further, 3 Kb of sequence data both upstream and downstream of each detected LTR homology were extracted and compared with Gag-Pol protein sequences within the GyDB 2.0 database [75, 76] using TBLASTN

(TBLASTN, RRID:SCR_011822). If at least 50% of any Gag-Pol sequence was covered by the flanking sequences with an identity > 30% and an E-value cutoff of 1e-8, the corresponding LTR was excluded from the solo-LTR list. The LTR homologies that lacked any Gag-Pol homology in both the upstream and downstream sequences were considered to be solo-LTRs. In addition, LTRs with Gag-Pol sequences on one side of flanking sequences were retained as truncated LTR-RTs. The timing of LTR-RT insertion was estimated based on the divergence between the 5′-LTR and 3′-LTR of the same transposon [77]. In this procedure, each LTR pair was aligned using MUSCLE v3.8.31 (MUSCLE, RRID:SCR_011812) [78] with default settings. Kimura's two-parameter method [79] was employed with a mutation rate of 1.3e-8 substitutions yr^{-1} per site to calculate approximate insertion time [80]. Superfamily classifications within the *Gypsy* and *Copia* classes are provided in **Table S10**. Although the actual mode of LTR-RT activation and amplification is manifested at the family level [81], as defined by >80% sequence homology in the LTR-RTs, we focused on overall genome properties that could be more carefully assayed and compared at the LTR-RT superfamily level (>60% homology), with categories such as Tat and Reina of *Gypsy* or Tork and Oryco of *Copia*. The proliferation history of different superfamilies of *Gypsy* and *Copia* LTR-RTs are provided in **Figs. S4** and **S5**. The distances of intact LTR-RTs to adjacent genes were calculated, and the relationships of proximity to gene and insertion time of LTR-RTs was also examined. Gene proximity for different superfamilies of *Gypsy* and *Copia* LTR-RTs are provided in **Figs. S6** and **S7, and Table S11**. The relationship between gene proximity and

insertion time for major LTR-RTs superfamilies are depicted in **Figs. S8** and **S9**.

To obtain further LTR-RT relationship insights, 5′-LTR sequences of all LTR-RTs were compared against each other with BLASTN. Two LTRs were assigned to the same cluster if they mutually covered at least 70% of their lengths with an identity of at least 60% between them. This clustering was performed using Silix v1.2.9 [82]. Solo-LTRs (*S*) and truncated LTR-RTs (*T*) were also mapped to the same cluster containing 5' LTRs from the most similar intact LTR-RTs (*I*). Furthermore, ratios of solo-LTR-RTs and truncated LTR-RTs, respectively, to intact LTR-RTs (*S*:*I*; *T*:*I*) as well as their sums were assessed to study the removal rates of LTR-RTs over the past several million years. We further assessed the proportions of clusters with *S*:*I* values greater than three to evaluate LTR-RT deletions. The abovementioned estimates remained consistent with or without shorter scaffolds, indicating that the draft genome assembly does not affect the results presented. To make an interspecific comparison, we also collected data on LTR-RT accumulation and removal rates for related plant species from a previous study [83], in which the same pipeline as ours was used for LTR-RTs analysis. Results of the interspecific comparison are provided in **Fig S10** and **Table S12**.

A few categories of LTR-RTs were highly abundant within the *M. oleifera* genome. Twenty-six annotated clades and one unclassified clade of *Gypsy* LTR-RTs, as well as 17 annotated clades of *Copia* were identified by querying the GyDB 2.0 database with full-length LTR-RTs of *M. oleifera*. Significant differences in their individual counts, average length, and genomic representation were found for

superfamilies with both *Gypsy* and *Copia* classes of LTR-RTs (**Table S10**). *Del* is the most prevalent clade of *Gypsy* in the *M. oleifera* genome, representing 6.99% of the assembled genome. *Sire* and *Tork* are the two most abundant clades of *Copia*, representing 3.77% and 1.16% of the assembled genome, respectively. More considerable variation in average sequence length was observed for clades of *Gypsy* (4,848 - 11,592 bp) compared to those of *Copia* (4,823-9,473 bp). In sum, for most clades of both *Gypsy* and *Copia* LTR-TRs, few recent amplification were identified while a single peak of ancient amplification 2-10 million years ago (mya) were observed. Exceptionally, *Galadriel* and *Tat* superfamilies of *Gypsy* showed an active recent amplification less than one mya (**Fig. S4** and **S5**). We observed some LTR-RTs overlapping genes for most of the subgroups of *Gypsy* and *Copia*, especially for the prevalent clades: about 1,500 from the *Del* clade of *Gypsy* were found to overlap with genes; > 200 from Galadriel overlapped, and also hundreds from *Sire*, *Tork*, *Oryco* and *Retrofit* of *Copia* overlapped (**Fig. S6, Fig. S7** and **Table S11**). Except for the ones overlapping with genes, LTR-RTs were mostly distributed in regions characterized by 3-5 Kb distance to genes. In addition, we found that gene-overlapping LTR-RTs had been generated over an extended period of time, as revealed by the insertion dates for the most representative sub-groups of *Gypsy* (**Fig. S8**) and *Copia* (**Fig. S9**).

When comparing *M. oleifera* to other related plant species with respect to LTR-RTs accumulation and removal rates, we found that the *M. oleifera* genome is characterized by the largest numbers of intact, solo- and truncated LTR-RTs.

Moreover, the *M. oleifera* genome has experienced relatively low removal rates (*S*:*I* = 2.28, (*S*+*T*)/*I* = 2.61) as evidenced by the lowest proportion of LTR clusters with *S*:*I* > 3 (**Fig. S10** and **Table S12**). Target site duplications (TSDs), usually 5 bp of identical sequence for LTR-RTs, are the direct repeats that occur at the insertion sites of most TEs. TSDs were detected for all (24,660) intact LTR-RTs. However, they were found for only 510 (<0.1% of 56,170) solo-LTRs, indicating that these elements called "solo-LTRs" in our analysis are mostly truncated LTR-RT rather than the products of unequal homologous recombination. As expected, very few (251 out of 8,196, or about 0.3%) of the truncated LTR-RTs had TSDs. Regardless of whether an LTR-RT has been converted into a solo-LTR or a truncated LTR-RT, this still represents decay of a formerly intact LTR-RT into a non-functional (i.e., immobile) status that will eventually be fully removed by the deletions associated with illegitimate recombination [80]. Given the abundance of LTR-RTs and their proximity to genes, it will be interesting to further explore their potential influence on genome evolution and gene expression.

Orthologous genes, whole genome duplication and phylogenetic inference

OrthoMCL v2.0.9 (Ortholog Groups of Protein Sequences, RRID:SCR_007839) [84] was used to identify orthologous and paralogous gene clusters in the assembled genomes of *M. oleifera* and 14 related plant species (**Table S13**), namely *Arabidopsis thaliana* [85], *Theobroma cacao* [86], *Citrus grandis* [87], *Populus trichocarpa* [88], *Eucalyptus grandis* [89, 90], *Glycine max* [91], *Vitis vinifera* [92, 93], *Solanum*

lycopersicum [54], *Coffea canephora* [94], *Helianthus annuus* [95], *Beta vugaris* [96], *Nelumbo nucifera* [97], *Aquilegia coerulea* [98] and *Oryza sativa* [99]. Recommended settings were used for all-against-all BLASTP comparisons (Blast+ v2.3.056) [51] and OrthoMCL analyses. OrthoMCL analyses identified 30,367 gene families (414,518 genes involved in these analyses) based on effective database sizes of all versus all BLASTP with an E-value of 10^{-5} and a Markov Chain Clustering default inflation parameter.

The amino acid sequences of 282 orthologous protein-coding single-copy genes (**Supplementary File 2)**, identified by OrthoMCL among the 15 analyzed genomes, were acquired and aligned with MUSCLE v3.8.31 [78], employing default settings (**Supplementary File 1** for commands and settings**)**. The concatenated amino acid sequences (**Supplementary File 3**) were trimmed using trimAI v1.2 (trimal -gt 0.8 -st 0.001 -cons 60) [100] and were further used for sequence evolution model selection with ModelFinder [101]. JTT+F+R5 was selected as the best model based on all employed criteria (Akaike Information Criterion AIC, corrected AIC and Bayesian Information Criterion). To construct the maximum likelihood phylogenetic tree (**Supplementary File 1** for commands and settings**)**, IQ-TREE v1.6.7 [102] was run with the selected optimal sequence evolution model (-m JTT+F+R5) and with ultrafast bootstrapping (-bb 1000) [103, 104], and employing the Shimodaira-Hasegawa-like approximate likelihood-ratio test (SH-aLRT, -alrt 1000) [105].

Phylogenetic dating (**Supplementary File 1** for commands and settings**)** was

done with the MCMCTree program of PAML v4.9h [106] with the following parameters: “burnin 100000, sampfreq 200, nsample 10000”. Rice (*O. sativa*) was defined as outgroup. The dating was calibrated against the recently summated timing of divergence [107]: the divergence of rice from other plant genomes at 113 - 128.63 Mya (refers to MRCA (most recent common ancestor), Monocotyledoneae: Acorales - [Dioscoreales + [Liliales + [Asparagales + Aracales + Poales]]], 113 - 128.63 Mya), divergence of *N. nucifera* and *A. coerulea* from other dicots at 119.6 - 128.6 Mya (refers to MRCA, Eudicotyledoneae: Ranunculales - [Vitales + Rosids + [Caryophyllales + Asterids]], 119.6 - 128.63 Mya), and divergence of *C. canephora*, *S. lycopersicum* and *H. annuus* to the lineage formed by *A. thaliana*, *V. vinifera* and other related plants at 85.8 - 128.63 Mya (refers to MRCA, Vitales - [Rosids + [Caryophyllales + Asterids]], 85.8 - 128.63 Mya; MRCA, Rosids (minus Vitales) - [Caryophyllales + Asterids], 85.8 - 128.63 Mya; MRCA, Caryophyllales - Asterids, 85.8 - 128.63 Mya). The Molecular Clock test as implemented in MEGA X [108] rejected the null hypothesis that all tips of the tree are equidistant from the root of the tree.

All branches of the reconstructed phylogenetic tree gained high support from both Shimodaira-Hasegawa-like approximate likelihood-ratio and the ultrafast bootstrapping tests with SH-aLRT > 88 % and UFBoot > 85 %, respectively (**Fig. 2b**). The phylogenetic analysis identified the closest relationship of *M. oleifera* (Santalales) to grape (*V. vinifera*, Vitales), with the divergence time between *M. oleifera* and grape estimated at ~ 88.9798 Mya with 95% confidence intervals of 37.7394 - 108.955 Mya.

N. nucifera (Proteales) and *Aquilegia coerulea* (Ranunculales) were forming a sister clade to all other Eudicots. The phylogenetic relationship among Ranunculales, Proteales, Santalales and Vitales is unresolved in the most recent phylogeny of the angiosperms (APG IV) [109] (http://www.mobot.org/MOBOT/Research/APweb/welcome.html, accessed at Oct. 22, 2018) [110].

Amino acid sequences of intra-specific in-paralogs constructed by OrthoMCL analyses were aligned with MUSCLE v3.8.31 [111] employing default settings. *Ks* (the number of synonymous substitutions per synonymous site) was calculated with KaKs_Calculator v2.0 [112] under a YN model, after the conversion of protein sequence alignments into the corresponding codon alignments with PAL2NAL v14 [113]. The *Ks* distribution suggests that the *M. oleifera* genome has not undergone any recent or lineage-specific whole-genome duplication (**Fig. S11**). This finding is also supported by the low number of intra-specific collinear blocks called with MCScanX (**Fig. S12**) [114].

Of the identified OrthoMCL gene families, 6,509 gene families (194,824 genes) were shared among all of the genomes analyzed. A total of 520 gene families (2,097 genes) were found to be specific to the assembled *M. oleifera* genome when compared with the other 14 genomes (**Table S14**). Using CAFE v4.0 [95, 115], 309 gene families were detected that have expanded, while 1,528 gene families were found to have contracted in the *M. oleifera* lineage (**Fig. 2b**). Hypergeometric tests were performed to determine if specific functional categories of KEGG or GO were

significantly overrepresented in the families that were significantly expanded or contracted within the *M. oleifera* genome. The expanded gene families were enriched for > 100 significant (q < 0.05) GO-terms of three different functional categories (Biological Process (BP), Cellular Component (CC), and Molecular Function (MF)) (**Table S15**) and seven KEGG pathways (**Table S16**). Three enriched categories were related to hormone signal transduction and to biosynthesis of tyrosine, isoquinoline alkaloid, cutin and wax, terpenoid, pantothenate and CoA, and glycine. The contracted gene families were enriched for > 400 GO-terms (**Table S17**) and 11 KEGG pathways (**Table S18**) related to various aspects of secondary metabolism, at q < 0.05. Results from functional enrichment analysis of rapidly evolving genes are summarized in **Table S19** (for GO enrichment) and **Table S20** (for KEGG enrichment).

Metabolic gene clusters and candidate genes for fatty acid biosynthesis pathways

It is evident that genes for numerous plant secondary metabolic pathways are sometimes densely clustered in a specific genomic region, generating biosynthetic gene clusters (BGCs) [116-118]. With the newly released and robust computational toolkit, plantiSMASH [119], 23 such BGCs related to various secondary metabolic pathways were detected (**Table S21** and **Supplementary File 4**), such as saccharide- (10 gene clusters), terpene- (4), alkaloid- (2), polyketide- (1), and lignan-polyketide (1)-related. An additional five putative BGCs were identified that could not be assigned to specific secondary metabolic pathways. The identified BGCs spanned 258

to 1,282 Kb and contained 3-8 core protein domains related to secondary metabolism.

Given the importance of fatty acid production in *M. oleifera*, we further annotated genes within the fatty acid biosynthesis pathway by querying the Plant Metabolic Network (PMN v12.5 (Plant Metabolic Network, RRID:SCR_003778) [120, 121], after enzymatic annotations for coding genes through the E2P2 package v3.1 [122]. The initial (*de novo*) fatty acid biosynthesis process mainly occurs in plastids [123] of leaf mesophyll cells, seeds, and oil-accumulating fruits in plants. In this process, acetyl and malonyl groups are condensed and further elongated to give rise to the production of 16:0-ACPs (palmitic acid) and 18:0-ACPs (stearic acid and oleic acids). After this initial process, very long chain fatty acids (VLCFAs, with 22 or more carbons) can be synthesized at the endoplasmic reticulum by sequential addition of C2 moieties from malonyl-CoA to form C18 acyl groups [124].

We detected a total of 14 genes that are predicted to function in the four reactions of the elongation cycle, including the condensation of long-chain acyl-CoA and malonyl-CoA to form 3-oxoacyl-CoA, the reduction to 3-hydroxyacyl-CoA, the dehydration to (2E)-alkan-2-enoyl-CoA, and the final reduction to an elongated fatty acyl-CoA [124]. We detected 19 candidate genes potentially functioning in the reactions of the initial process (**Fig. S13**), and 14 genes in the subsequent VLCFA biosynthesis pathway (**Fig. 2c**). Interestingly, we found the genes of the VLCFA pathway forming two gene clusters of local duplicates, one composed of 4 genes (Maole_016461, Maole_016463, Maole_016466, and Maole_016467) and the other of two genes (Maole_017397 and Maole_017398). These six genes occurring in

localized clusters are all predicted to be involved in the four key reactions of the chain elongation cycle, suggesting an important effect of local gene duplication on efficient VLCFA production. By comparison, only a few cases (one including Maole_003221.T1 and Maole_003222.T1, the other including Maole_008716.T1 and Maole_008717.T1) of localized gene duplication were found for the initial fatty acid biosynthesis pathway.

Conclusions

In sum, we provide a high quality *de novo* genome assembly, and in-depth characterization for *M. oleifera*, combining PacBio single molecule long-reads and 10x Genomics linked reads. The excellent quality of the genome assembly is supported by both the 92.29% BUSCO analysis-based single-copy gene coverage and the 99.32% (PacBio long reads), 98.5% (10x Genomics linked reads) and 91-98% (Illumina RNA sequencing reads) mapping rates of the genome and transcriptome reads. Of note, the significantly low heterozygosity of the sequenced genome was a key factor for the high continuity in genome assembly of *M. oleifera* obtained in this study. This low level of heterozygosity also suggests a high level of inbreeding in the wild population of trees that was the source of genomic DNA used for genome analysis. The novel genomic resources generated in the present study provide vital foundation for further studies on the genetics of metabolite biogenesis, the genetic basis of the vulnerable status, the significance of local gene duplications in genomes without a recent whole genome duplication, and for biotechnology aiming at an

efficient exploration of valuable plant compounds. The pattern of birth-death dynamics and gene proximity of LTR-RTs, revealed here, provide a basis for future LTR-RTs studies in plants. It will be particularly interesting to investigate whether the observed slow rate of LTR-RT amplification and removal are related to the long-lived perennial lifestyle of this largely undomesticated tree species. As the only whole genome and the second genome released for the Olacaceae family and in the Santalales order, the present data resource is also of critical value for phylogenomic and comparative genomic studies.

Availability of supporting data

The genome assembly, annotations, and other supporting data are available via the *Giga*Science database *Giga*DB[125]. The raw sequence data have been deposited in the Short Read Archive (SRA) under NCBI BioProject ID PRJNA472200. All commands and parameter settings for genome assembly, quality assessment of genome assembly, transcriptome assembly from RNA-seq, repeat and gene annotation, ortholog identification, phylogenetic reconstruction and dating been uploaded to protocols.io[126].

Abbreviations

BGCs: biosynthetic gene clusters; bp: base pair; BUSCO: benchmarking universal single-copy orthologs; CDS: coding sequence; Gb: gigabases; Kb: kilobases; LTR: long terminal repeat; Mb: megabases; mya: million years ago; PSESP: plant species

with an extremely small population; RT: retrotransposons; SMRT: Single-Molecule Real-Time; TE: transposable element; VLCFAs: very long chain fatty acids.

Acknowledgments

This study was funded by Fundamental Research Funds for the Central Universities (NO. YX2013-41), by the construction of the workstation for Academician Bennetzen (NO. 2015AC018), by the Science Fund of China's Yunnan government (NO. 2015BB018), and by the State Key Laboratory of Phytochemistry and Plant Resources in West China (NO. P2015-KF11).

Author Contributions

JFM, YM and JLB conceived and designed the study; CQX, HL, SSZ, ZW, SQJ, SW, FC, YQS, SN, KHJ, DZ, RGZ, WG, QG and QZY prepared the materials, conducted the experiments and analyzed all data; JFM, CQX and YM wrote the manuscript; XW, FM, IP, YVP, JLB and XRW were involved in data interpretation and finalizing the manuscript draft. All authors read and approved the final draft.

Conflict of Interest

The authors declare that they have no competing financial interests.

References

1. Wu Z, Raven P and Hong D. Flora of China. Vol. 5 (Ulmaceae through Basellaceae). Science Press, Beijing, and Missouri Botanical Garden Press, St.

Louis, 2003.
2. Sun W: *Malania oleifera*. The IUCN Red List of Threatened Species 1998: e.T32361A9701100. http://dx.doi.org/10.2305/IUCN.UK.1998.RLTS.T32361A9701100.en. Accessed 08 July 2018.
3. Ma Y, Chen G, Edward Grumbine R, Dao Z, Sun W and Guo H. Conserving plant species with extremely small populations (PSESP) in China. Biodiversity and Conservation. 2013;22(3):803-809. doi:10.1007/s10531-013-0434-3.
4. Xie WD, Chen JH, Lai JY, Shi HM, Huang KX, Liu JB, et al. Analysis on relationship between geographic distribution of *Malania oleifera* and hydro-thermal factors. Journal of Tropical & Subtropical Botany. 2009;17(4):388-394. doi:10.3969/j.issn.1005-3395.2009.4.2125.
5. Xie WD, Chen JH, Lai JY, Shi HM, Lin SF, Liu B, et al. Life-table analysis of *Malania oleifera*, a rare and endangered plant. Journal of Central South University of Forestry & Technology. 2009;29(2):73-76.
6. Wu Y, Li X and Hu Y. Reproductive biology of *Malania oeifera*. Acta Scientiarum Naturalium Universitatis Sunyatseni. 2004;43(2):81-83.
7. Lai JY, Shi HM, Pan CL, Chen SW, Ye YZ, Ming LI, et al. Pollination biology of rare and endangered species *Malania oleifera* Chun et Lee. Journal of Beijing Forestry University. 2008.
8. Xiong Y, Hong L, Li H and Li X. Bionomics of the pathogens of *Malania oleifera* seed rot. Forest Pest & Disease. 2003;22:1-4.
9. Liu SS, Hu YH, Maghuly F, Porth IM and Mao JF. The complete chloroplast genome sequence annotation for *Malania oleifera*, a critically endangered and important bioresource tree. Conservation Genetics Resources. 2018; doi:10.1007/s12686-018-1005-4.
10. Tang TF, Liu XM, Ling M, Lai F, Zhang L, Zhou YH, et al. Constituents of the essential oil and fatty acid from *Malania oleifera*. Industrial Crops and Products. 2013;43:1-5. doi:https://doi.org/10.1016/j.indcrop.2012.07.003.
11. Ma BL, Liang SF, Zhao DY, Xu AX and Zhang KJ. Study on plants containing nervonic acid. Acta Botanica Boreali-occidentalia Sinica. 2004;24(12):2362-2365.
12. Sandhir R, Khan M, Chahal A and Singh I. Localization of nervonic acid beta-oxidation in human and rodent peroxisomes: impaired oxidation in Zellweger syndrome and X-linked adrenoleukodystrophy. Journal of Lipid Research. 1998;39(11):2161-2171.
13. Oda E, Hatada K, Kimura J, Aizawa Y, Thanikachalam PV and Watanabe K. Relationships between serum unsaturated fatty acids and coronary risk factors: negative relations between nervonic acid and obesity-related risk factors. International Heart Journal. 2005;46(6):975-85.
14. Amminger GP, Schafer MR, Klier CM, Slavik JM, Holzer I, Holub M, et al. Decreased nervonic acid levels in erythrocyte membranes predict psychosis in help-seeking ultra-high-risk individuals. Molecular Psychiatry.

2012;17(12):1150-1152. doi:10.1038/mp.2011.167.
15. Yuan Y, Dai X, Wang D and Zeng X. Purification, characterization and cytotoxicity of malanin, a novel plant toxin from the seeds of *Malania oleifera*. Toxicon. 2009;54(2):121-7. doi:https://doi.org/10.1016/j.toxicon.2009.03.024.
16. Preparing *Arabidopsis* Genomic DNA for Size-Selected ~20 kb SMRTbell™ Libraries. http://www.pacb.com/wp-content/uploads/2015/09/Shared-Protocol-Preparing-Arabidopsis-DNA-for-20-kb-SMRTbell-Libraries.pdf. Accessed 20 Sept 2017.
17. Zheng GXY, Lau BT, Schnall-Levin M, Jarosz M, Bell JM, Hindson CM, et al. Haplotyping germline and cancer genomes with high-throughput linked-read sequencing. Nature Biotechnology. 2016;34:303. doi:10.1038/nbt.3432.
18. An open-source release of Long Ranger 2.2.0. https://github.com/10xGenomics/longranger. Accessed 01 Dec 2017.
19. Koren S, Walenz BP, Berlin K, Miller JR, Bergman NH and Phillippy AM. Canu: scalable and accurate long-read assembly via adaptive k-mer weighting and repeat separation. Genome Research. 2017;27(5):722-736.
20. Marcais G and Kingsford C. A fast, lock-free approach for efficient parallel counting of occurrences of k-mers. Bioinformatics. 2011;27(6):764-770. doi:10.1093/bioinformatics/btr011.
21. Liu B, Shi Y, Yuan J, Hu X, Zhang H, Li N, et al. Estimation of genomic characteristics by analyzing k-mer frequency in *de novo* genome projects. arXiv preprint arXiv:13082012. 2013.
22. Xiao CL, Chen Y, Xie SQ, Chen KN, Wang Y, Han Y, et al. MECAT: fast mapping, error correction, and *de novo* assembly for single-molecule sequencing reads. Nature Methods. 2017;14:1072. doi:10.1038/nmeth.4432.
23. Li H. Minimap and miniasm: fast mapping and *de novo* assembly for noisy long sequences. Bioinformatics. 2016;32(14):2103-2110. doi:10.1093/bioinformatics/btw152.
24. Chin CS, Peluso P, Sedlazeck FJ, Nattestad M, Concepcion GT, Clum A, et al. Phased diploid genome assembly with single-molecule real-time sequencing. Nature Methods. 2016;13(12):1050-1054. doi:10.1038/nmeth.4035.
25. FALCON: experimental PacBio diploid assembler. https://github.com/PacificBiosciences/FALCON/. Accessed 01 May 2018.
26. Ultra-fast *de novo* assembler using long noisy reads. https://github.com/ruanjue/smartdenovo. Accessed 01 May 2018.
27. A fuzzy bruijn graph (FBG) approach to long noisy reads assembly. https://github.com/ruanjue/wtdbg-1.2.8. Accessed 01 May 2018.
28. Weisenfeld NI, Kumar V, Shah P, Church DM and Jaffe DB. Direct determination of diploid genome sequences. Genome Research. 2017;27(5):757-767. doi:10.1101/gr.214874.116.
29. Pipelines for a *de novo* assembly software: Supernova. https://support.10xgenomics.com/de-novo-assembly/software/overview/latest/welcome. Accessed 01 May 2018.

30. Simao FA, Waterhouse RM, Ioannidis P, Kriventseva EV and Zdobnov EM. BUSCO: assessing genome assembly and annotation completeness with single-copy orthologs. Bioinformatics. 2015;31(19):3210-3212. doi:10.1093/bioinformatics/btv351.
31. A variantCaller tool to get consensus and variant calls from mapped PacBio reads. https://github.com/PacificBiosciences/GenomicConsensus. Accessed 01 Dec 2017.
32. Li H and Durbin R. Fast and accurate long-read alignment with Burrows–Wheeler transform. Bioinformatics. 2010;26(5):589-595. doi:10.1093/bioinformatics/btp698.
33. Yeo S, Coombe L, Warren RL, Chu J and Birol I. ARCS: scaffolding genome drafts with linked reads. Bioinformatics. 2018;34(5):725-731. doi:10.1093/bioinformatics/btx675.
34. Walker BJ, Abeel T, Shea T, Priest M, Abouelliel A, Sakthikumar S, et al. Pilon: an integrated tool for comprehensive microbial variant detection and genome assembly improvement. PLoS One. 2014;9(11):e112963. doi:10.1371/journal.pone.0112963.
35. Chaisson MJ and Tesler G. Mapping single molecule sequencing reads using basic local alignment with successive refinement (BLASR): application and theory. BMC Bioinformatics. 2012;13(1):238. doi:10.1186/1471-2105-13-238.
36. A long read aligner tool for PacBio. https://github.com/PacificBiosciences/blasr. Accessed 01 May 2018.
37. Langmead B and Salzberg SL. Fast gapped-read alignment with Bowtie 2. Nature Methods. 2012;9:357. doi:10.1038/nmeth.1923.
38. RepeatModeler: a *de novo* repeat family identification and modeling package. http://www.repeatmasker.org/RepeatModeler/. Accessed 01 May 2018.
39. Price AL, Jones NC and Pevzner PA. *De novo* identification of repeat families in large genomes. Bioinformatics. 2005;21Suppl 1:i351-i358. doi:10.1093/bioinformatics/bti1018.
40. Bao W, Kojima KK and Kohany O. Repbase Update, a database of repetitive elements in eukaryotic genomes. Mobile DNA. 2015;6:11. doi:10.1186/s13100-015-0041-9.
41. A program that screens DNA sequences for interspersed repeats and low complexity DNA sequences: RepeatMasker. http://www.repeatmasker.org/. Accessed 01 May 2018.
42. Bolger AM, Lohse M and Usadel B. Trimmomatic: a flexible trimmer for Illumina sequence data. Bioinformatics. 2014;30(15):2114-2120. doi:10.1093/bioinformatics/btu170.
43. Martin M. Cutadapt removes adapter sequences from high-throughput sequencing reads. EMBnetjournal. 2011;17(1). doi:10.14806/ej.17.1.200.
44. Kim D, Langmead B and Salzberg SL. HISAT: a fast spliced aligner with low memory requirements. Nature Methods. 2015;12(4):357-360.
45. A quality control tool for high throughput sequence data. https://www.bioinformatics.babraham.ac.uk/projects/fastqc/. Accessed 01 May

2018.
46. Pertea M, Pertea GM, Antonescu CM, Chang TC, Mendell JT and Salzberg SL. StringTie enables improved reconstruction of a transcriptome from RNA-seq reads. Nature Biotechnology. 2015;33(3):290-295.
47. Grabherr MG, Haas BJ, Yassour M, Levin JZ, Thompson DA, Amit I, et al. Full-length transcriptome assembly from RNA-Seq data without a reference genome. Nature Biotechnology. 2011;29:644. doi:10.1038/nbt.1883.
48. Fu L, Niu B, Zhu Z, Wu S and Li W. CD-HIT: accelerated for clustering the next-generation sequencing data. Bioinformatics. 2012;28(23):3150-3152.
49. Keller O, Kollmar M, Stanke M and Waack S. A novel hybrid gene prediction method employing protein multiple sequence alignments. Bioinformatics. 2011;27(6):757-763. doi:10.1093/bioinformatics/btr010.
50. Stanke M, Diekhans M, Baertsch R and Haussler D. Using native and syntenically mapped cDNA alignments to improve *de novo* gene finding. Bioinformatics. 2008;24(5):637-644. doi:10.1093/bioinformatics/btn013.
51. Boratyn GM, Schäffer AA, Agarwala R, Altschul SF, Lipman DJ and Madden TL. Domain enhanced lookup time accelerated BLAST. Biology Direct. 2012;7 1:12. doi:10.1186/1745-6150-7-12.
52. Swarbreck D, Wilks C, Lamesch P, Berardini T, Garcia-Hernandez M and Foerster H. The *Arabidopsis* Information Resource (TAIR): gene structure and function annotation. Nucleic Acids Research. 2007;36:D1009-D1014.
53. Jaillon O, Aury JM, Noel B, Policriti A, Clepet C, Casagrande A, et al. The grapevine genome sequence suggests ancestral hexaploidization in major angiosperm phyla. Nature. 2007;449(7161):463-467. doi:10.1038/nature06148.
54. Tomato Genome Consortium. The tomato genome sequence provides insights into fleshy fruit evolution. Nature. 2012;485(7400):635-641.
55. Cruz F, Julca I, Gómez-Garrido J, Loska D, Marcet-Houben M, Cano E, et al. Genome sequence of the olive tree, *Olea europaea*. GigaScience. 2016;5(1):29. doi:10.1186/s13742-016-0134-5.
56. Slater GSC and Birney E. Automated generation of heuristics for biological sequence comparison. BMC Bioinformatics. 2005;6:31. doi:10.1186/1471-2105-6-31.
57. A generic tool for sequence alignment. https://www.ebi.ac.uk/about/vertebrate-genomics/software/exonerate. Accessed 01 May 2018.
58. Cantarel BL, Korf I, Robb SM, Parra G, Ross E, Moore B, et al. MAKER: an easy-to-use annotation pipeline designed for emerging model organism genomes. Genome Research. 2008;18(1):188-196. doi:10.1101/gr.6743907.
59. Kent WJ. BLAT--the BLAST-like alignment tool. Genome Research. 2002;12(4):656-664. doi:10.1101/gr.229202.
60. Bairoch A and Apweiler R. The SWISS-PROT protein sequence database and its supplement TrEMBL in 2000. Nucleic Acids Research. 2000;28(1):45-48.
61. Bateman A, Birney E, Cerruti L, Durbin R, Etwiller L, Eddy SR, et al. The

Pfam protein families database. Nucleic Acids Research. 2002;30(1):276-280.

62. Punta M, Coggill P, Eberhardt R, Mistry J, Tate J and Boursnell C. The Pfam protein families database. Nucleic Acids Research. 2011;40:D290-D301.

63. Quevillon E, Silventoinen V, Pillai S, Harte N, Mulder N, Apweiler R, et al. InterProScan: protein domains identifier. Nucleic Acids Research. 2005;33(Web Server issue):W116-W120. doi:10.1093/nar/gki442.

64. National Center for Biotechnology Information. https://www.ncbi.nlm.nih.gov/. Accessed 01 Dec 2017.

65. ExPASy Bioinformatics Resources Portal. http://www.expasy.ch/sprot. Accessed 01 May 2018.

66. UniProt. http://www.ebi.ac.uk/uniprot. Accessed 01 May 2018.

67. Pfam. http://pfam.xfam.org/. Accessed 01 May 2018.

68. The KOG Browser. http://genome.jgi-psf.org/help/kogbrowser.jsf. Accessed 01 May 2018.

69. Kanehisa M and Goto S. KEGG: Kyoto Encyclopedia of Genes and Genomes. Nucleic Acids Research. 2000;28 1:27-30.

70. KO (KEGG ORTHOLOGY) Database. http://www.genome.jp/kegg/ko.html. Accessed 01 Dec 2017.

71. Harris MA, Clark J, Ireland A, Lomax J, Ashburner M, Foulger R, et al. The Gene Ontology (GO) database and informatics resource. Nucleic Acids Research. 2004;32(Database issue):D258-D261. doi:10.1093/nar/gkh036.

72. Gene Ontology Consortium. http://www.geneontology.org. Accessed 01 May 2018.

73. Ellinghaus D, Kurtz S and Willhoeft U. LTRharvest, an efficient and flexible software for *de novo* detection of LTR retrotransposons. BMC Bioinformatics. 2008;9(1):18. doi:10.1186/1471-2105-9-18.

74. Steinbiss S, Willhoeft U, Gremme G and Kurtz S. Fine-grained annotation and classification of *de novo* predicted LTR retrotransposons. Nucleic Acids Research. 2009;37(21):7002-7013. doi:10.1093/nar/gkp759.

75. Llorens C, Futami R, Covelli L, Domínguez-Escribá L, Viu JM, Tamarit D, et al. The Gypsy Database (GyDB) of mobile genetic elements: release 2.0. Nucleic Acids Research. 2011;39suppl 1:D70-D74. doi:10.1093/nar/gkq1061.

76. Lloréns C, Futami R, Bezemer D and Moya A. The Gypsy Database (GyDB) of mobile genetic elements. Nucleic Acids Research. 2008;36 suppl 1:D38-D46. doi:10.1093/nar/gkm697.

77. SanMiguel P, Gaut BS, Tikhonov A, Nakajima Y and Bennetzen JL. The paleontology of intergene retrotransposons of maize. Nature Genetics. 1998;20(1):43-45. doi:10.1038/1695.

78. Edgar RC. MUSCLE: multiple sequence alignment with high accuracy and high throughput. Nucleic Acids Research. 2004;32(5):1792-1797. doi:10.1093/nar/gkh340.

79. Kimura M. A simple method for estimating evolutionary rates of base substitutions through comparative studies of nucleotide sequences. Journal of Molecular Evolution. 1980;16(2):111-120. doi:10.1007/bf01731581.

80. Ma J and Bennetzen JL. Rapid recent growth and divergence of rice nuclear genomes. Proceedings of the National Academy of Sciences of the United States of America. 2004;101(34):12404-12410. doi:10.1073/pnas.0403715101.
81. Wicker T, Sabot F, Hua-Van A, Bennetzen JL, Capy P, Chalhoub B, Flavell A, Leroy P, Morgante M, Panaud O, Paux E, SanMiguel P, Schulman AH. A unified classification system for eukaryotic transposable elements. Nature Reviews Genetics. 2007;8(12):973-982.
82. Miele V, Penel S and Duret L. Ultra-fast sequence clustering from similarity networks with SiLiX. BMC Bioinformatics. 2011;12(1):116. doi:10.1186/1471-2105-12-116.
83. Lyu H, He Z, Wu CI and Shi S. Convergent adaptive evolution in marginal environments: unloading transposable elements as a common strategy among mangrove genomes. New phytologist. 2018;217(1):428-438. doi:10.1111/nph.14784.
84. Li L, Stoeckert CJ and Roos DS. OrthoMCL: Identification of ortholog groups for eukaryotic genomes. Genome Research. 2003;13(9):2178-2189. doi:10.1101/gr.1224503.
85. Cheng CY, Krishnakumar V, Chan AP, Thibaud-Nissen F, Schobel S and Town CD. Araport11: a complete reannotation of the *Arabidopsis thaliana* reference genome. The Plant Journal : for cell and molecular biology. 2017;89(4):789-804. doi:10.1111/tpj.13415.
86. Motamayor JC, Mockaitis K, Schmutz J, Haiminen N, Iii DL, Cornejo O, et al. The genome sequence of the most widely cultivated cacao type and its use to identify candidate genes regulating pod color. Genome Biology. 2013;14(6):r53. doi:10.1186/gb-2013-14-6-r53.
87. Wang X, Xu Y, Zhang S, Cao L, Huang Y, Cheng J, et al. Genomic analyses of primitive, wild and cultivated citrus provide insights into asexual reproduction. Nature Genetics. 2017;49:765. doi:10.1038/ng.3839.
88. Tuskan GA, Difazio S, Jansson S, Bohlmann J, Grigoriev I, Hellsten U, et al. The genome of black cottonwood, *Populus trichocarpa* (Torr. & Gray). Science. 2006;313(5793):1596-1604. doi:10.1126/science.1128691.
89. Myburg AA, Grattapaglia D, Tuskan GA, Hellsten U, Hayes RD, Grimwood J, et al. The genome of *Eucalyptus grandis*. Nature. 2014;510:356-362. doi:10.1038/nature13308.
90. Bartholome J, Mandrou E, Mabiala A, Jenkins J, Nabihoudine I, Klopp C, et al. High-resolution genetic maps of Eucalyptus improve *Eucalyptus grandis* genome assembly. New Phytologist. 2015;206(4):1283-1296. doi:10.1111/nph.13150.
91. Schmutz J, McClean PE, Mamidi S, Wu GA, Cannon SB, Grimwood J, et al. A reference genome for common bean and genome-wide analysis of dual domestications. Nature Genetics. 2014;46(7):707-713. doi:10.1038/ng.3008.
92. The French-Italian Public Consortium for Grapevine Genome Characterization. The grapevine genome sequence suggests ancestral hexaploidization in major angiosperm phyla. Nature. 2007;449:463. doi:10.1038/nature06148.

93. Canaguier A, Grimplet J, Di Gaspero G, Scalabrin S, Duchêne E, Choisne N, et al. A new version of the grapevine reference genome assembly (12X.v2) and of its annotation (VCost.v3). Genomics Data. 2017;14:56-62. doi:10.1016/j.gdata.2017.09.002.
94. Denoeud F, Carretero-Paulet L, Dereeper A, Droc G, Guyot R, Pietrella M, et al. The coffee genome provides insight into the convergent evolution of caffeine biosynthesis. Science. 2014;345(6201):1181-1184. doi:10.1126/science.1255274.
95. Badouin H, Gouzy J, Grassa CJ, Murat F, Staton SE, Cottret L, et al. The sunflower genome provides insights into oil metabolism, flowering and Asterid evolution. Nature. 2017;546:148. doi:10.1038/nature22380.
96. Dohm JC, Minoche AE, Holtgrawe D, Capella-Gutierrez S, Zakrzewski F, Tafer H, et al. The genome of the recently domesticated crop plant sugar beet (*Beta vulgaris*). Nature. 2013;505:546-549. doi:10.1038/nature12817.
97. Ming R, VanBuren R, Liu Y, Yang M, Han Y, Li LT, et al. Genome of the long-living sacred lotus (*Nelumbo nucifera* Gaertn.). Genome Biology. 2013;14(5):R41. doi:10.1186/gb-2013-14-5-r41.
98. Filiault D, Ballerini E, Mandakova T, Akoz G, Derieg N, Schmutz J, et al. The *Aquilegia* genome: adaptive radiation and an extraordinarily polymorphic chromosome with a unique history. eLife. 2018; 7:e36426. doi: 10.7554/eLife.36426.
99. Ouyang S, Zhu W, Hamilton J, Lin H, Campbell M, Childs K, et al. The TIGR Rice Genome Annotation Resource: improvements and new features. Nucleic Acids Research. 2007;35(Database issue):D883-887. doi:10.1093/nar/gkl976.
100. Capella-Gutiérrez S, Silla-Martínez JM and Gabaldón T. trimAl: a tool for automated alignment trimming in large-scale phylogenetic analyses. Bioinformatics. 2009;25(15):1972-1973. doi:10.1093/bioinformatics/btp348.
101. Kalyaanamoorthy S, Minh BQ, Wong TKF, von Haeseler A and Jermiin LS. ModelFinder: fast model selection for accurate phylogenetic estimates. Nature Methods. 2017;14:587. doi:10.1038/nmeth.4285.
102. Nguyen LT, Schmidt HA, von Haeseler A and Minh BQ. IQ-TREE: a fast and effective stochastic algorithm for estimating maximum-likelihood phylogenies. Molecular biology and evolution. 2015;32(1):268-274. doi:10.1093/molbev/msu300.
103. Minh BQ, Nguyen MAT and von Haeseler A. Ultrafast approximation for phylogenetic bootstrap. Molecular Biology and Evolution. 2013;30(5):1188-1195. doi:10.1093/molbev/mst024.
104. Hoang DT, Chernomor O, von Haeseler A, Minh BQ and Vinh LS. UFBoot2: Improving the Ultrafast Bootstrap Approximation. Molecular Biology and Evolution. 2018;35(2):518-522. doi:10.1093/molbev/msx281.
105. Guindon S, Dufayard JF, Lefort V, Anisimova M, Hordijk W and Gascuel O. New algorithms and methods to estimate maximum likelihood phylogenies: assessing the performance of PhyML 3.0. Systematic Biology. 2010;59(3):307-321. doi:10.1093/sysbio/syq010.

106. Yang Z. PAML 4: phylogenetic analysis by maximum likelihood. Molecular Biology and Evolution. 2007;24(8):1586-1591. doi:10.1093/molbev/msm088.
107. Morris JL, Puttick MN, Clark JW, Edwards D, Kenrick P, Pressel S, et al. The timescale of early land plant evolution. Proceedings of the National Academy of Sciences of the United States of America. 2018;115(10):E2274-E2283. doi:10.1073/pnas.1719588115.
108. Kumar S, Stecher G, Li M, Knyaz C and Tamura K. MEGA X: Molecular Evolutionary Genetics Analysis across Computing Platforms. Molecular Biology and Evolution. 2018;35(6):1547-1549. doi:10.1093/molbev/msy096.
109. Chase MW, Christenhusz MJM, Fay MF, Byng JW, Judd WS, et al. An update of the angiosperm phylogeny group classification for the orders and families of flowering plants: APG IV. Botanical Journal of the Linnean Society. 2016;181(1):1-20. doi:10.1111/boj.12385.
110. Stevens PF and Davis HM. The angiosperm phylogeny website - a tool for reference and teaching in a time of change. Proceedings of the American Society for Information Science and Technology. 2005;42(1). doi:10.1002/meet.14504201249.
111. Edgar RC. MUSCLE: multiple sequence alignment with high accuracy and high throughput. Nucleic Acids Research. 2004;32(5):1792-1797. doi:10.1093/nar/gkh340.
112. Wang D, Zhang Y, Zhang Z, Zhu J and Yu J. KaKs_Calculator 2.0: a Toolkit incorporating gamma-series methods and sliding window strategies. Genomics, Proteomics & Bioinformatics. 2010;8(1):77-80. doi:https://doi.org/10.1016/S1672-0229(10)60008-3.
113. Suyama M, Torrents D and Bork P. PAL2NAL: robust conversion of protein sequence alignments into the corresponding codon alignments. Nucleic Acids Research. 2006;34(Web Server issue):W609-W612. doi:10.1093/nar/gkl315.
114. Wang Y, Tang H, Debarry JD, Tan X, Li J, Wang X, et al. MCScanX: a toolkit for detection and evolutionary analysis of gene synteny and collinearity. Nucleic Acids Research. 2012;40(7):e49. doi:10.1093/nar/gkr1293.
115. De Bie T, Cristianini N, Demuth JP and Hahn MW. CAFE: a computational tool for the study of gene family evolution. Bioinformatics. 2006;22(10):1269-1271. doi:10.1093/bioinformatics/btl097.
116. Chae L, Kim T, Nilo-Poyanco R and Rhee SY. Genomic signatures of specialized metabolism in plants. Science. 2014;344(6183):510-513.
117. Nützmann HW, Huang A and Osbourn A. Plant metabolic clusters - from genetics to genomics. New phytologist. 2016;211(3):771-789. doi:10.1111/nph.13981.
118. Nützmann HW and Osbourn A. Gene clustering in plant specialized metabolism. Current Opinion in Biotechnology. 2014;26:91-109. doi: 10.1016/j.copbio.2013.10.009.
119. Kautsar SA, Suarez Duran HG, Blin K, Osbourn A and Medema MH. plantiSMASH: automated identification, annotation and expression analysis of plant biosynthetic gene clusters. Nucleic Acids Research.

2017;45(W1):W55-W63. doi:10.1093/nar/gkx305.

120. Schlapfer P, Zhang P, Wang C, Kim T, Banf M, Chae L, et al. Genome-wide prediction of metabolic enzymes, pathways and gene clusters in plants. Plant Physiology. 2017;173(4):2041-2059. doi:10.1104/pp.16.01942.

121. PMN: a plant metabolic pathway databases. https://www.plantcyc.org/. Accessed 01 May 2018.

122. E2P2: An enzyme annotation pipeline used to generate the species-specific metabolic databases. https://gitlab.com/rhee-lab/E2P2/tree/master. Accessed 01 Dec 2017.

123. Yasuno R, von Wettstein-Knowles P and Wada H. Identification and molecular characterization of the β-ketoacyl-[acyl carrier protein] synthase component of the *Arabidopsis* mitochondrial fatty acid synthase. Journal of Biological Chemistry. 2004;279(9):8242-8251.

124. Jakobsson A, Westerberg R and Jacobsson A. Fatty acid elongases in mammals: their regulation and roles in metabolism. Progress in Lipid Research. 2006;45(3):237-249.

125. Xu CQ; Liu H; Zhou SS; Zhang DX; Zhao W; Wang S; Chen F; Sun YQ; Nie S; Jia KH; Jiao SQ; Zhang RG; Yun QZ; Guan W; Wang X; Gao Q; Bennetzen JL; Maghuly F; Porth I; de Peer YV; Wang XR; Ma Y; Mao JF (2018): Supporting data for "Genome sequence of *Malania oleifera*, a tree with great value for nervonic acid production" GigaScience Database. http://dx.doi.org/10.5524/100549

126. Chao-Qun Xu, Hui Liu, Shan-Shan Zhou, Dong-Xu Zhang, Wei Zhao, Sihai Wang, Fu Chen, Yan-Qiang Sun, Shuai Nie, Kai-Hua Jia, Si-Qian Jiao, Ren-Gang Zhang, Quan-Zheng Yun, Wenbin Guan, Xuewen Wang, Jeffrey L. Bennetzen, Fatemeh Maghuly, Ilga Porth, Yves Van de Peer1, Xiao-Ru Wang, Yongpeng Ma, Jian-Feng Mao (2018): Malania oleifera genome assembly and annotation. protocols.io http://dx.doi.org/10.17504/protocols.io.u7nezme

Figures

Fig. 1 Images of *M. oleifera*, recorded distribution range and sampling sites.

a-d, mature tree (a), flower (b), fruit (c) and naturally germinated seedling (d); e, blue shaded region denotes the reported distribution range of *M. oleifera*, while the red circle denotes the position (N23.90°, E104.09°, Guangnan County, Yunnan) where one tree was sampled for whole genome sequencing, and the red triangle and square denote the positions (N23.9°, E106.00°, Funing County, Yunnan and N24.78°,

E106.57°, Leye County, Guangxi) where trees were sampled for RNA sequencing.

Fig. 2 Repeat composition, phylogenomic inferences and biosynthesis pathway for very long chain fatty acids synthesis in *M. oleifera*.

a. genome proportions of genic and various repeat sequences; b. phylogenetic tree, divergence time, and profiles of gene families that underwent expansion or contraction; bootstrapping supports (SH-aLRT/UFBoot) are presented along with the 95% confidence intervals for each dating point in brackets; c. annotated genes involved in the biosynthesis pathway of very long chain fatty acids (a fatty acid with minimum 22 carbon moieties) in *M. oleifera*.

Table

Table 1. Statistics of the final genome assembly for *M. oleifera*.

Supplementary Figures

Fig. S1. Length distribution of PacBio subreads.

Fig. S2. K-mer frequency distribution estimated from PacBio sequences after filtering and correction at k-mer size of 17. A k-mer refers to an artificial sequence division of K nucleotides. From k-mer frequencies, genomic characteristics (genome size, repeat structure and heterozygous rate) could be estimated. Peaks at depth of 21 are annotated with dashed lines.

Fig. S3. Distribution of AED (annotation edit distance) scores from gene prediction. AED = 0 indicates perfect agreement between the gene prediction and the transcript

and protein evidence; AED = 1 indicates no evidence support for annotation.

Fig. S4. Proliferation history of different superfamilies of the *Gypsy* class of LTR-RTs (long terminal repeat-retrotransposons) in the *M. oleifera* genome.

Fig. S5. Proliferation history of different superfamilies of the *Copia* class of LTR-RTs in the *M. oleifera* genome.

Fig. S6. Gene proximity for different superfamilies of the *Gypsy* class of LTR-RTs in the *M. oleifera* genome.

The natural logarithm of the base distance between an LTR-RT and an adjacent gene (plus one) was used as the X axis.

Fig. S7. Gene proximity for different superfamilies of the *Copia* class of LTR-RTs in the *M. oleifera* genome.

The natural logarithm of the base distance between an LTR-RT and an adjacent gene (plus one) was used as the X axis.

Fig. S8. Gene proximity and insertion time for major superfamilies of the *Gypsy* class of LTR-RTs in the *M. oleifera* genome.

The natural logarithm of the base distance between an LTR-RT and an adjacent gene (plus one) was used as the Y axis, time in mya as X axis.

Fig. S9. Gene proximity and insertion time for major superfamilies of the *Copia* class of LTR-RTs in the *M. oleifera* genome.

The natural logarithm of the base distance between an LTR-RT and an adjacent gene (plus one) was used as the Y axis, time in mya as X axis.

Fig. S10. Birth and death of LTR-RTs (long terminal repeat-retrotransposons) in the *M.*

oleifera genome compared to six other members of Rosids and two members from Asterids. (a) total numbers of intact LTR-RTs in the genome; (b) comparison of $S + T$ values among these nine plant species; (c) total numbers of intact LTR-RTs and traces of LTR-RT death; (d) ratios of solo-LTR to intact LTR-RT (S:I). (e) proportions of LTR-RTs found in the clusters with high removal rates (filtered S:$I \geqslant 3$). S, number of solo-LTRs; T, number of truncated LTR-RTs I, number of intact LTR-RTs.

Fig. S11. *Ks* distribution of paralogs in synteny blocks within the *M. oleifera* genome.

Fig. S12. Gene:synteny-block pattern in the *M. oleifera* genome.

Fig. S13. Genes annotation for the initial (*de novo*) fatty acid biosynthesis process in the *M. oleifera* genome.

Supplementary Tables

Table S1. Summary of PacBio and Illumina sequencing data (10x Genomics and RNA sequencing) generated in the present study. IDs of the study, sample, library and accessions in NCBI SRA and employed sequencing platform, material origins of the sequenced DNA or RNA, statistics of raw and cleaned data, and mapping rates are shown.

Table S2. Data summary from 10x Genomics analysis based on GemCode index multiplicity. Read subsets are based on the number of associated reads for each index. For raw reads, all indices (including those with N’s) are included in the count. For all other read sets, only the indices without N’s were used for binning.

Table S3. Estimation of genome characteristics based on 17-mer statistics.

Table S4. Statistics of the different versions of *M. oleifera* genome assembly in ascending order. N50: shortest sequence length at 50% of the genome; L50: smallest number of contigs whose length sum produces N50. NA: data not available; * statistics for contigs/scaffolds. Gene completeness was generated by assessment with 1,440 single copy orthologs from the BUSCO embryophyta_odb9 database.

Table S5. Summary of the annotated TEs in the genome assembly for *M. oleifera*. LTR: Long Terminal Repeat retrotransposons; LINE: Long Interspersed Nuclear Element, a category of non-LTR (long terminal repeat) retroelements; SINE: Short Interspersed Nuclear Element, a category of non-autonomous and non-coding retroelements (TEs); RC: Rolling-circle transposons.

Table S6. Summary of transcriptome assemblies using three different analysis pipelines.

Table S7. Summary of annotated genes.

AED: Annotation Edit Distance; gene region (including 5’, 3’ UTRs, exons and introns).

Table S8. Summary of BUSCO evaluation for gene prediction.

Table S9. Summary of functional annotation of predicted genes.

Table S10. Superfamilies within the *Gypsy* and *Copia* LTR-RTs classes of TEs.

Table S11. Gene proximity of superfamilies of *Gypsy* and *Copia* classes of LTR-RTs.

Table S12. Comparison of the number of original and filtered intact LTR-RT, solo-LTR and Truncated LTR TEs among 9 plant species.

Table S13. Genomic data used for phylogenomic and gene family analyses. Origins,

download links, assembly versions, genome properties and references of 14 genomes are shown.

Table S14. Summary of gene family analyses. Unique groups and genes, single-copy and duplicated groups and genes are summarized for the 15 analyzed plant genomes.

Table S15. GO enrichment of expanded gene families. (A) 'Category' is the Gene Ontology (GO) term ID; (B) 'P_value' is the overrepresentation p-value indicating the observed frequency of a given term among analyzed genes is equal to the expected frequency based on the null distribution; i.e. lower p-values indicate stronger evidence for overrepresentation; (C) 'Q_value' is the Benjamini and Hochberg adjusted p-value, (D) 'numEPInCat' is the number of expanded gene families in the corresponding GO category; (E) 'numInCat' is the number of detected gene families in the corresponding GO category; (F) 'Term' is the GO term; (G) 'Ontology' indicates which ontology the term comes from. Significant at $q < 0.05$.

Table S16. KEGG enrichment of expanded gene families. (A) 'KO category' is the KEGG Orthology (KO) category ID; (B) 'P_value' is the over represented p-value indicating the observed frequency of a given term among analyzed genes is equal to the expected frequency based on the null distribution; i.e. lower p-values indicate stronger evidence for overrepresentation; (C) 'Q_value' is the Benjamini and Hochberg adjusted p-value, (D) 'numEPInCat' is the number of expanded gene families in the corresponding KO category; (E) 'numInCat' is the number of detected gene families in the corresponding KO category; (F) 'Pathway' is the KEGG pathway; (G) 'Class' indicates which KEGG class the pathway comes from. Significant at q <

0.05.

Table S17. GO enrichment of contracted gene families. (A) 'Category' is the Gene Ontology (GO) term ID; (B) 'P_value' is the over represented p-value indicating the observed frequency of a given term among analyzed genes is equal to the expected frequency based on the null distribution; i.e. lower p-values indicate stronger evidence for overrepresentation; (C) 'Q_value' is the Benjamini and Hochberg adjusted p-value, (D) 'numEPInCat' is the number of expanded gene families in the corresponding GO category; (E) 'numInCat' is the number of detected gene families in the corresponding GO category; (F) 'Term' is the GO term; (G) 'Ontology' indicates which ontology the term comes from. Significant at $q < 0.05$.

Table S18. KEGG enrichment of contracted gene families. (A) 'KO category' is the KEGG Orthology (KO) category ID; (B) 'P_value' is the over represented p-value indicating the observed frequency of a given term among analyzed genes is equal to the expected frequency based on the null distribution; i.e. lower p-values indicate stronger evidence for overrepresentation; (C) 'Q_value' is the Benjamini and Hochberg adjusted p-value, (D) 'numEPInCat' is the number of expanded gene families in the corresponding KO category; (E) 'numInCat' is the number of detected gene families in the corresponding KO category; (F) 'Pathway' is the KEGG pathway; (G) 'Class' indicates which KEGG class the pathway comes from. Significant at $q <$ 0.05.

Table S19. GO enrichment of fast evolving gene families. (A) 'Category' is the Gene Ontology (GO) term ID; (B) 'P_value' is the over represented p-value indicating the

observed frequency of a given term among analyzed genes is equal to the expected frequency based on the null distribution; i.e. lower p-values indicate stronger evidence for overrepresentation; (C) ‘Q_value’ is the Benjamini and Hochberg adjusted p-value, (D) ‘numEPInCat’ is the number of expanded gene families in the corresponding GO category; (E) ‘numInCat’ is the number of detected gene families in the corresponding GO category; (F) ‘Term’ is the GO term; (G) ‘Ontology’ indicates which ontology the term comes from. Significant at $q < 0.05$.

Table S20. KEGG enrichment of fast evolving gene families. (A) ‘KO category’ is the KEGG Orthology (KO) category ID; (B) ‘P_value’ is the over represented p-value indicating the observed frequency of a given term among analyzed genes is equal to the expected frequency based on the null distribution; i.e. lower p-values indicate stronger evidence for overrepresentation; (C) ‘Q_value’ is the Benjamini and Hochberg adjusted p-value, (D) ‘numEPInCat’ is the number of expanded gene families in the corresponding KO category; (E) ‘numInCat’ is the number of detected gene families in the corresponding KO category; (F) ‘Pathway’ is the KEGG pathway; (G) ‘Class’ indicates which KEGG class the pathway comes from. Significant at $q < 0.05$.

Table S21. Summary of 23 metabolic gene clusters in the *M. oleifera* genome. Genomic coordinates, gene composition, core protein domains related to metabolism and pathway assignments are shown.

Supplementary Files

Supplementary File 1. The commands and parameter settings for all steps in genome assembly, quality assessment of the genome assembly, transcriptome assembly from RNA-seq data, repeat and gene annotation, ortholog identification and phylogenetic reconstruction and dating.

Supplementary File 2. Gene names/codes for the 282 orthologous protein-encoding single-copy genes used in the phylogenetic analyses.

Supplementary File 3. Concatenated alignment of amino acid sequences used in the phylogenetic analyses.

Supplementary File 4. Visualization of each metabolic gene cluster detected within the *M. oleifera* genome.

Figures

Fig. 1 Images of *M. oleifera*, recorded distribution range and sampling sites.

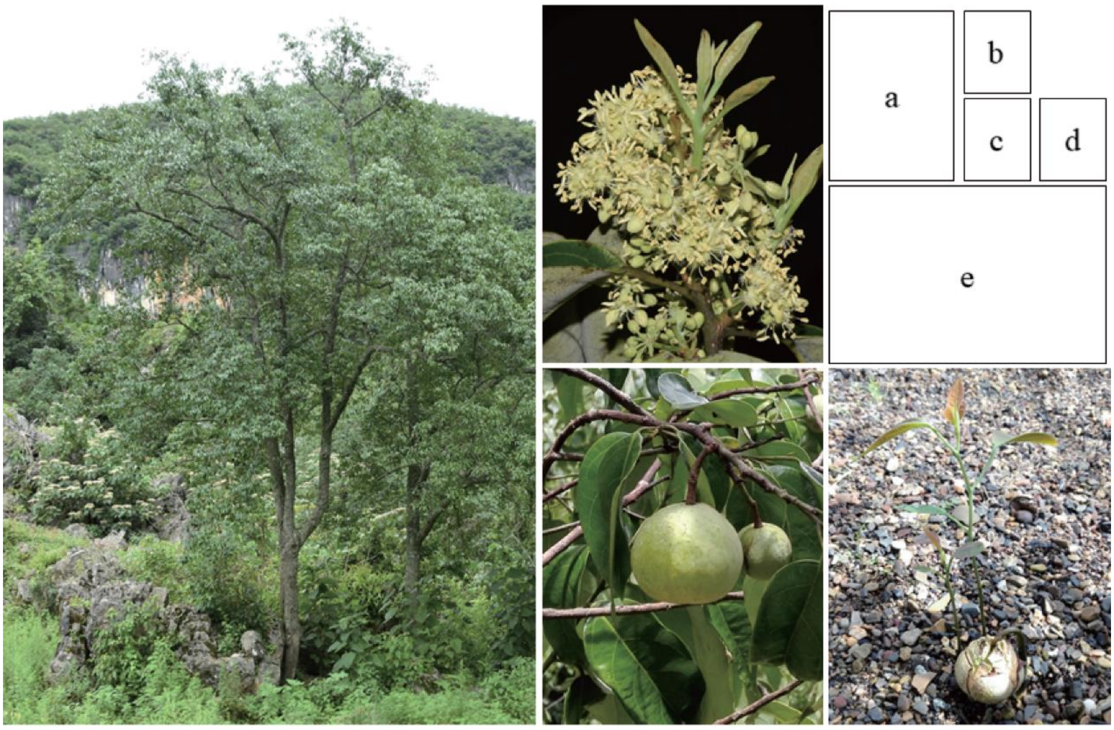

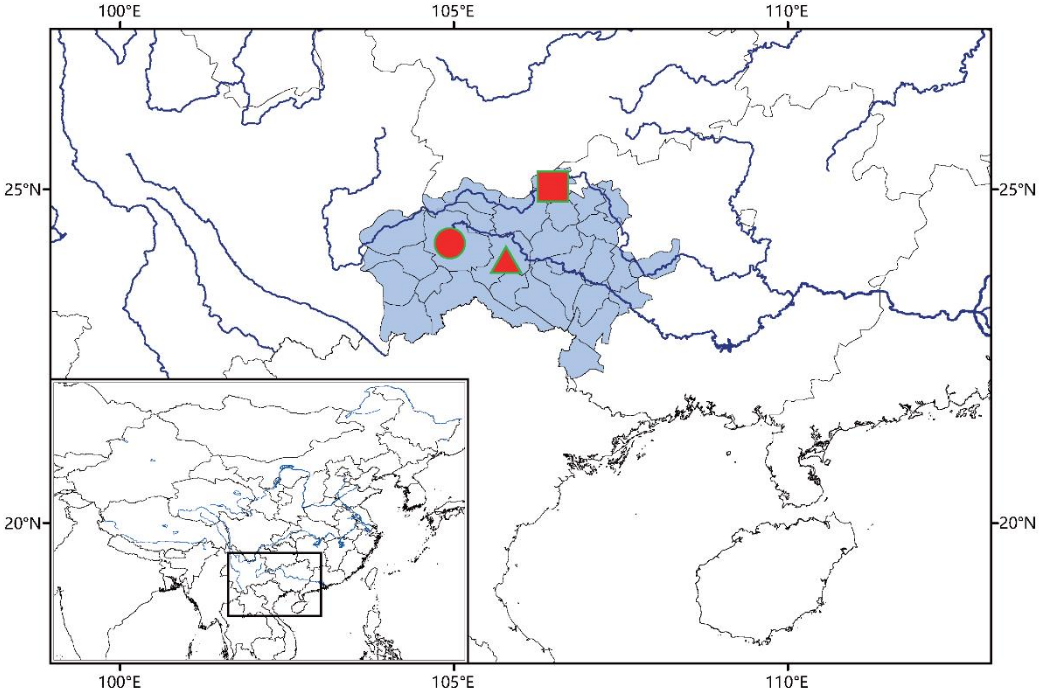

Fig. 2 Repeat composition for *M. oleifera*, phylogenomic depiction of gene family

expansions and contractions, and biosynthesis pathway for very long chain fatty acids synthesis in *M. oleifera*.

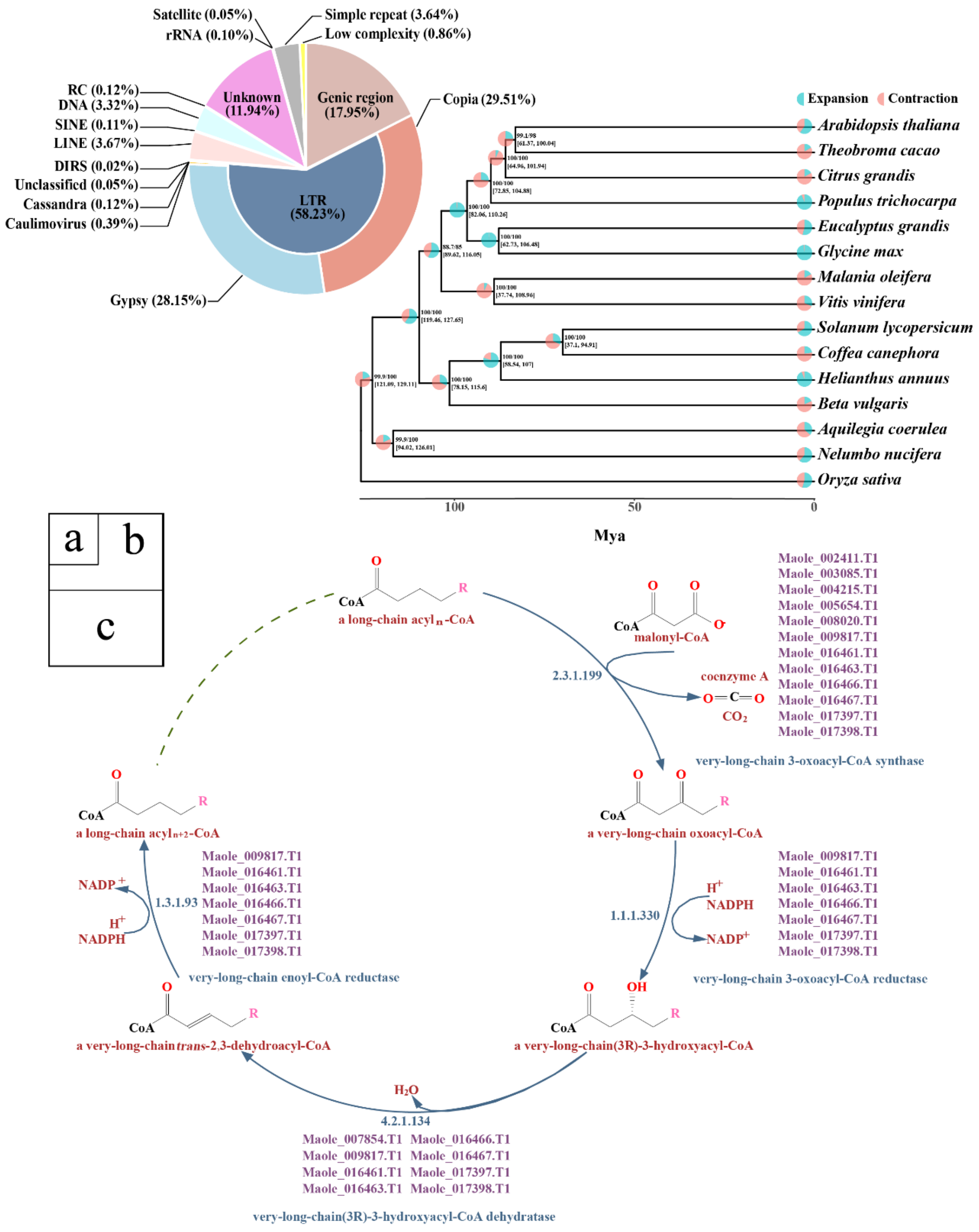

Tables

Table 1. Statistics of the final genome assembly for *M. oleifera*.

	Contig		Scaffold	
	**Size (bp)**	**Number**	**Size (bp)**	**Number**
**Total Size**	1,509,344,141	-	1,519,782,615	-
**Total Number**	-	2,987	-	1,277
**N10**	2,959,726	39	11,755,999	10
**N50**	1,218,690	376	4,647,296	94
**N90**	272,293	1,337	1,153,659	339
**Max.**	6,703,356	-	25,060,663	-
**Min.**	334	-	8256	-
**Mean**	505,304	-	1,190,119	-
**Median**	200,407	-	85,436	-
**Gap**	-	-	10,438,474 (0.69%)	1,710
**GC Content**	36.07%	-	35.82%	-

-, Data not available.

Table S1

**Accession**	**Study**	**Bioproject_accession**	**Biosample_accession**	**Library_ID**
SRR7221!	SRP149014	PRJNA472200	SAMN09235853	DS-pacbio
SRR7221!	SRP149014	PRJNA472200	SAMN09235853	DS-10x
SRR7221!	SRP149014	PRJNA472200	SAMN09235854	MoF1
SRR7221!	SRP149014	PRJNA472200	SAMN09235855	MoF2
SRR7221!	SRP149014	PRJNA472200	SAMN09235856	MoF3
SRR7221!	SRP149014	PRJNA472200	SAMN09235857	MoF4
SRR7221!	SRP149014	PRJNA472200	SAMN09235858	Mole
SRR7221!	SRP149014	PRJNA472200	SAMN09235859	leaf
SRR7221!	SRP149014	PRJNA472200	SAMN09235860	seed

Table S2

**Number (**	**Raw reads**	**>=1**	**>=1000**	**>=3000**
**Number (**	19,394,967	19,319,151	27,368	830
**Total nun**	449,888,927	449,809,812	42,975,267	9,529,752
**Percent o**	100%	99.98%	9.55%	2.12%
**Average I**	23.20	23.28	1,570.27	11,481.63

Table S3

**K-mer va**	**Amount of K-mer use**	**Coverage**	**Genome size**	**Heterozygos**
17	29,971,959,192	21	1498597960	0.0566264

Table S4

**Versions**	**Strategy**	**Assemblied genome si**	**Sequence number**	**N50**
v0.1	Canu	1.53	4,396	1.12 Mb
v0.2	MECAT	1.19	14,019	117 Kb
v0.3	minimap+miniasm	1.55	4,276	744 Kb
v0.4	Canu+FALCON	1.54	7,860	665 Kb
v0.5	Canu+SMARTdenovo	1.45	4,341	611 Kb
v0.6	Canu+Wtdbg	1.46	5,709	782 Kb
v0.7	SMARTdenov	1.51	3,038	1.21 Mb
v0.8	Wtdbg	1.61	9,743	549 Kb
v0.9	Supernova	1.56/1.63*	110,395/58,910*	46 Kb/1.57 M
v1.0	v0.7+arrow×2	1.51	3,038	1.20 Mb
v1.1	v1.0+arcs	1.52/1.51*	3,038/1,274*	1.20 Mb/4.6!
v1.2f	v1.1+arrow+pilon×3	1.52/1.51*	2,987/1,277*	1.22 Mb/4.6!

Table S5

**Class**	**Family**	**Number**	**Length (bp)**	**Percent (%**
LTR		981,473	884,911,643	58.23
	Cassandra	3,798	1,780,693	0.12
	Caulimovirus	6,736	5,864,500	0.39
	Copia	537,029	448,422,064	29.51
	DIRS	1,298	265,737	0.02
	Gypsy	430,047	427,864,686	28.15
LINE		89,562	55,769,410	3.67
	CR1	6,538	2,307,810	0.15

	L1	58,839	36,673,807	2.41
	L1-Tx1	290	88,189	0.01
	L2	3,942	1,546,597	0.10
	RTE-BovB	19,953	15,153,007	1.00
SINE		11,788	1,700,153	0.11
	tRNA-Core-RTE	1,959	267,428	0.02
	tRNA-RTE	9,829	1,432,725	0.09
DNA		114,267	50,512,738	3.32
	CMC-Chapaev	1,991	190,839	0.01
	CMC-EnSpm	44,914	23,758,719	1.56
	MuLE-MuDR	6,966	6,239,911	0.41
	TcMar-Tc1	738	143,208	0.01
	hAT-Ac	28,249	8,002,000	0.53
	hAT-Tag1	22,706	9,286,682	0.61
	hAT-Tip100	3,359	1,172,790	0.08
RC		2,464	1,832,449	0.12
	Helitron	2,464	1,832,449	0.12
Unknown		495,092	181,461,434	11.94
rRNA		3,065	1,467,141	0.10
Satellite		4,125	815,956	0.05
Simple_repeat		469,716	55,388,128	3.64
Low_complexity		51,628	13,100,851	0.86
Total		2,223,180	1,246,959,903	82.05

Table S6

**Pipelines**	**Number of genes**	**Total length of genes (**	**Average length of gen**	**Number of t**
Trinity *de*	49,316	33,821,803	685.82	52,509
Trinity ge	33,891	27,185,969	802.16	35,493
StringTie	28,782	45,886,824	1,594.29	42,176
Merged tr	NA	NA	NA	57,299

Table S7

**Gene Nur**	**Transcript Number**	**Transcript Number (A**	**Average Gene Region**	**Average Tra**
24,094	24,094	20,092	11,809.11	1,460.47

Table S8

	**BUSCO groups**	**Percentage**
Complete	1,258	87.36%
Complete	1,217	84.51%
Complete	41	2.85%
Fragmente	71	4.93%
Missing B	111	7.71%
Total BUS	1,440	100.00%

Table S9

**Annotate**	**Databases**	**Count**	**Percentage**
Total genes		24,094	100.00%
Annotated	NR	13,792	57.20%

	Swiss_Prot	21,833	90.60%
	TrEMBL	22,026	91.40%
	Pfam	18,512	76.80%
	KOG	21,102	87.60%
	GO	18,972	78.70%
	KO	7,923	32.90%
Unannotated		2,023	8.40%

Table S10

**Super fan**	**Clade ID**	**Count**	**Average length (bp)**	**Total length**
Gypsy	Del	12,194	8,706.42	#########
Gypsy	Galadriel	452	6,318.11	2,855,785
Gypsy	Tat	343	10,297.52	3,532,051
Gypsy	Athila	335	10,362.77	3,471,527
Gypsy	CRM	318	7,645.90	2,431,396
Gypsy	Reina	85	7,271.95	618,116
Gypsy	GRhodo	25	9,071.04	226,776
Gypsy	412/mdg1	24	9,184.25	220,422
Gypsy	Mag	19	8,713.16	165,550
Gypsy	V_clade	13	8,207.62	106,699
Gypsy	Maggy	13	9,320.46	121,166
Gypsy	Osvaldo	12	8,243.75	98,925
Gypsy	Pyret	11	8,567.18	94,239
Gypsy	Gmr1	9	8,627.89	77,651
Gypsy	MGLR3	8	9,371.75	74,974
Gypsy	Tse3	7	8,304.14	58,129
Gypsy	Cer1	7	8,044.00	56,308
Gypsy	Pyggy	3	9,703.33	29,110
Gypsy	Micropia/mdg3	3	8,355.00	25,065
Gypsy	Ty3	2	7,028.00	14,056
Gypsy	Tor1	1	4,848.00	4,848
Gypsy	REM	1	7,502.00	7,502
Gypsy	Gypsy	1	7,359.00	7,359
Gypsy	CsRN1	1	8,983.00	8,983
Gypsy	Cer2_3	1	11,592.00	11,592
Gypsy	17.6	1	7,832.00	7,832
Gypsy	unclassified	24	8,115.83	194,780
Gypsy	total	13,913	8,674.40	#########
Copia	Sire	6,051	9,473.96	57,326,922
Copia	Tork	2,890	6,094.98	17,614,500
Copia	Oryco	756	6,427.50	4,859,193
Copia	Retrofit	681	5,886.53	4,008,728
Copia	Osser	33	8,140.48	268,636
Copia	Ty	24	8,040.83	192,980
Copia	Copia	17	6,142.82	104,428
Copia	CoDi_D	16	6,681.44	106,903
Copia	GalEA	14	8,214.71	115,006
Copia	pCretro	13	6,857.62	89,149

Copia	Hydra	13	7,729.23	100,480
Copia	CoDi_I	9	7,401.11	66,610
Copia	Mtanga	8	5,132.75	41,062
Copia	Tricopia	6	8,549.17	51,295
Copia	1731	6	7,171.83	43,031
Copia	CoDi_C	2	4,823.00	9,646
Copia	PyRE1G1	1	5,020.00	5,020
Copia	total	10,540	8,064.86	85,003,589

Table S11

				**Distance to the clo**
**Super fan**	**Clade ID**	**Overlapping**	**< 100 bp**	**101-1,000 b**
Gypsy	Del	1,539	7	134
Gypsy	Galadriel	218	3	31
Gypsy	Tat	63		25
Gypsy	Athila	68	1	5
Gypsy	CRM	95	2	9
Gypsy	Reina	51		2
Gypsy	GRhodo	1	0	0
Gypsy	412/mdg1	3	0	0
Gypsy	Mag	6	0	1
Gypsy	V_clade	3	0	0
Gypsy	Maggy	4	0	0
Gypsy	Osvaldo	3	0	0
Gypsy	Pyret	2	0	0
Gypsy	Gmr1	0	0	0
Gypsy	Tse3	1	0	0
Gypsy	MGLR3	1	0	0
Gypsy	Cer1	3	0	0
Gypsy	Pyggy	1	0	0
Gypsy	Micropia/mdg3	2	0	0
Gypsy	Ty3	1	0	0
Gypsy	Tor1	0	0	0
Gypsy	REM	0	0	0
Gypsy	CsRN1	0	0	0
Gypsy	Cer2_3	1	0	0
Gypsy	17.6	0	0	0
Copia	Sire	474	6	64
Copia	Tork	913	23	240
Copia	Oryco	257	5	46
Copia	Retrofit	326	9	70
Copia	Osser	5	0	1
Copia	Ty	6	0	4
Copia	Copia	6	0	0
Copia	CoDi_D	10	0	1
Copia	GalEA	6	0	1
Copia	pCretro	5	0	2
Copia	Hydra	4	0	1

Copia	CoDi_I	3	0	0
Copia	Mtanga	2	0	0
Copia	Tricopia	2	0	0
Copia	1731	3	0	1
Copia	CoDi_C	2	0	0
Copia	PyRE1G1	0	0	0

Table S12

**Species**	**Intact LTR-RT (*I*)**	**Cluster number**	**Solo-LTR (*S*)**	**Truncated I**
Arabidops	237	142	222	203
Coffea ca	1,843	438	9,337	2,829
Eucalyptu	3,353	536	7,991	2,500
Glycine m	4,847	663	55,534	8,481
Populus tr	1,290	429	4,843	2,243
Solanum l	3,899	379	22,775	5,839
Theobrom	1,497	272	13,202	2,288
Vitis vinif	2,411	357	9,738	3,684
Malania c	24,660	4,960	56,170	8,196

Table S13

**Species**	**Links**	**Version**	**Genes**	**nome size (M**
eta vulgar	s/all/GCF/000/511/025/	RefBeet-1.2.2	28120	566
legia coer	e.gov/pz/portal.html#!ir	v3.1	30023	292
umbo nuci	nt.be/plaza/versions/pla	v1.1	26685	929
itis vinifer	os://phytozome.jgi.doe.g	Genoscope.12X	26346	486
lus trichoc	os://phytozome.jgi.doe.g	v3.0	41335	423
Glycine ma	os://phytozome.jgi.doe.g	v1.0	56044	978
trus grand	//citrus.hzau.edu.cn/ora	version1	30123	346
obroma ca	os://phytozome.jgi.doe.g	v1.1	29452	346
dopsis tha	os://phytozome.jgi.doe.g	TAIR10	27416	135
alyptus gra	os://phytozome.jgi.doe.g	v2.0	36349	691
um lycoper	os://phytozome.jgi.doe.g	ITAG2.4	34725	823
fea caneph	http://coffee-genome.org	-	25574	569
anthus ann	s/all/GCF/002/127/325/	HanXRQr1.0	73728	3000
Oryza sativ	os://phytozome.jgi.doe.g	MSU_v7.0	42189	372

-: data no
available.

1 Dohm JC, Minoche AE, Holtgrawe D, Capella-Gutierrez S, Zakrzewski F, Tafer H,
2 Filiault D, Ballerini E, Mandakova T, Akoz G, Derieg N, Schmutz J, et al. The Aqu
3 Ming R, VanBuren R, Liu Y, Yang M, Han Y, Li L-T, et al. Genome of the long-liv
4 The French–Italian Public Consortium for Grapevine Genome Characterization. The
5 Tuskan GA, Difazio S, Jansson S, Bohlmann J, Grigoriev I, Hellsten U, et al. The ge
6 Schmutz J, Cannon SB, Schlueter J, Ma J, Mitros T, Nelson W, et al. Genome seque
7 Wang X, Xu Y, Zhang S, Cao L, Huang Y, Cheng J, et al. Genomic analyses of prin
8 Motamayor JC, Mockaitis K, Schmutz J, Haiminen N, Iii DL, Cornejo O, et al. The

9 Cheng CY, Krishnakumar V, Chan AP, Thibaud-Nissen F, Schobel S and Town CD
10 Myburg AA, Grattapaglia D, Tuskan GA, Hellsten U, Hayes RD, Grimwood J, et al
11 Bartholome J, Mandrou E, Mabiala A, Jenkins J, Nabihoudine I, Klopp C, et al. Hig
12 Tomato Genome Consortium. The tomato genome sequence provides insights into f
13 Denoeud F, Carretero-Paulet L, Dereeper A, Droc G, Guyot R, Pietrella M, et al. Th
14 Badouin H, Gouzy J, Grassa CJ, Murat F, Staton SE, Cottret L, et al. The sunflower
15 Ouyang S, Zhu W, Hamilton J, Lin H, Campbell M, Childs K, et al. The TIGR Rice

Table S14

Species	Unique groups	Unique genes	Single-copy groups	Duplicated g
Aquilegia	963	3,413	9,457	4,042
Arabidops	677	2,426	8,136	4,704
Beta vulga	497	2,261	9,638	2,735
Citrus gra	967	4,639	10,351	3,655
Coffea ca	527	1,681	10,199	3,088
Eucalyptu	757	2,666	8,943	4,524
Glycine m	1,615	4,525	2,532	12,500
Helianthu	1,538	10,629	6,230	7,767
Malania c	520	2,097	9,329	2,624
Nelumbo n	420	1,267	7,369	4,725
Oryza sati	2,017	9,688	7,599	5,452
Populus tr	809	2,418	5,877	8,695
Solanum l	905	3,947	9,052	4,902
Theobrom	510	2,189	10,901	3,409
Vitis vinif	563	1,525	9,651	3,203

Table S15

Category	P_value	Q_value	numDEInCat	numInCat
GO:00504	5.79E-36	5.03E-32	28	28
GO:00094	1.49E-31	6.47E-28	39	66
GO:00097	3.61E-26	1.05E-22	42	99
GO:00087	2.77E-25	6.02E-22	31	53
GO:00801	6.70E-22	1.16E-18	37	92
GO:19008	2.61E-21	3.78E-18	28	52
GO:00160	1.75E-19	2.17E-16	27	55
GO:00098	3.00E-18	3.26E-15	27	59
GO:00314	1.25E-17	1.21E-14	25	52
GO:00009	5.15E-17	4.17E-14	19	29
GO:00506	5.29E-17	4.17E-14	39	137
GO:00057	3.52E-16	2.55E-13	193	1,830
GO:00904	8.87E-16	5.93E-13	17	25
GO:00436	1.33E-15	8.24E-13	12	12
GO:00098	4.21E-15	2.44E-12	43	186
GO:00102	1.90E-14	1.03E-11	57	322
GO:00098	2.54E-14	1.30E-11	25	69
GO:00165	8.41E-14	4.06E-11	22	54
GO:00073	1.98E-13	9.08E-11	18	36
GO:00094	1.96E-12	8.51E-10	23	68

GO:00104	2.31E-12	9.56E-10	12	16
GO:00068	6.98E-11	2.76E-08	17	42
GO:00055	5.20E-10	1.96E-07	97	881
GO:00186	5.83E-10	2.11E-07	12	22
GO:00161	7.37E-10	2.56E-07	10	15
GO:00435	1.03E-09	3.45E-07	32	161
GO:00169	1.53E-09	4.94E-07	12	24
GO:00054	1.67E-09	5.17E-07	11	19
GO:00040	1.86E-09	5.38E-07	7	7
GO:00048	1.86E-09	5.38E-07	7	7
GO:00460	2.03E-09	5.69E-07	10	16
GO:00098	2.56E-09	6.94E-07	20	72
GO:00102	2.80E-09	7.37E-07	10	16
GO:00069	4.11E-09	1.05E-06	78	678
GO:00164	4.41E-09	1.09E-06	59	447
GO:00063	4.63E-09	1.12E-06	145	1,552
GO:00063	5.12E-09	1.20E-06	54	390
GO:00038	5.57E-09	1.27E-06	9	13
GO:00063	7.48E-09	1.67E-06	158	1,745
GO:00057	1.18E-08	2.55E-06	84	757
GO:00167	1.21E-08	2.57E-06	40	260
GO:00483	2.52E-08	5.21E-06	37	230
GO:00479	4.12E-08	8.33E-06	6	6
GO:00096	4.24E-08	8.38E-06	21	93
GO:00100	4.96E-08	9.58E-06	23	107
GO:00096	5.70E-08	1.08E-05	10	20
GO:00312	8.39E-08	1.55E-05	28	156
GO:00097	9.76E-08	1.77E-05	43	307
GO:00458	1.44E-07	2.55E-05	9	17
GO:00044	1.80E-07	3.08E-05	40	283
GO:00163	1.81E-07	3.08E-05	14	45
GO:00048	2.75E-07	4.60E-05	9	18
GO:00432	2.92E-07	4.79E-05	34	225
GO:00095	4.80E-07	7.71E-05	188	2,265
GO:00068	5.08E-07	8.03E-05	10	24
GO:00339	5.19E-07	8.05E-05	8	15
GO:00044	6.79E-07	0.000103462	10	25
GO:00097	7.82E-07	0.000117122	37	264
GO:00062	1.24E-06	0.000182747	8	16
GO:00427	1.69E-06	0.000244132	11	33
GO:00303	1.76E-06	0.000250647	11	34
GO:00703	1.89E-06	0.000264856	9	22
GO:00525	3.06E-06	0.000415131	7	13
GO:00971	3.06E-06	0.000415131	7	13
GO:00421	3.55E-06	0.000474293	5	6
GO:00048	3.74E-06	0.000492131	9	23
GO:00080	3.87E-06	0.000496058	12	43
GO:00059	3.88E-06	0.000496058	10	29

GO:00045	4.04E-06	0.000501773	12	43
GO:00060	4.04E-06	0.000501773	12	43
GO:00058	4.94E-06	0.00060477	189	2,333
GO:00525	5.94E-06	0.000688325	7	14
GO:00525	5.94E-06	0.000688325	7	14
GO:00525	5.94E-06	0.000688325	7	14
GO:00525	5.94E-06	0.000688325	7	14
GO:00068	6.18E-06	0.000706331	7	14
GO:00068	7.08E-06	0.00079843	14	59
GO:00314	7.47E-06	0.000824013	6	10
GO:00455	7.59E-06	0.000824013	8	20
GO:00527	7.59E-06	0.000824013	8	20
GO:00037	7.82E-06	0.00083843	105	1,185
GO:00071	1.01E-05	0.001067572	29	202
GO:00038	1.40E-05	0.001469137	4	4
GO:00081	1.74E-05	0.001799307	7	16
GO:00801	2.09E-05	0.002140955	8	22
GO:00057	2.38E-05	0.002407312	61	593
GO:00001	2.54E-05	0.002541337	9	29
GO:00468	2.87E-05	0.002831045	8	22
GO:00168	4.09E-05	0.003993684	7	18
GO:00300	4.31E-05	0.004163037	7	18
GO:00465	4.94E-05	0.004714724	7	19
GO:00038	5.68E-05	0.005365185	6	13
GO:00099	6.46E-05	0.006039303	15	80
GO:00713	6.88E-05	0.006360158	5	9
GO:00800	7.51E-05	0.006773208	18	115
GO:00800	7.51E-05	0.006773208	18	115
GO:00471	7.56E-05	0.006773208	5	9
GO:00103	8.30E-05	0.007355518	7	20
GO:00526	8.47E-05	0.007405402	18	116
GO:00095	8.52E-05	0.007405402	9	34
GO:00097	8.94E-05	0.007690702	13	65
GO:00060	9.53E-05	0.008117931	6	14
GO:00301	0.000122816	0.010359461	5	10
GO:00423	0.000156675	0.012820277	4	6
GO:19015	0.000156675	0.012820277	4	6
GO:00800	0.000157322	0.012820277	7	22
GO:00069	0.000157892	0.012820277	37	333
GO:00400	0.000161658	0.01300446	5	10
GO:00002	0.000166335	0.01325793	12	60
GO:00430	0.000171335	0.013532329	9	37
GO:00458	0.00017474	0.013676921	30	251
GO:00335	0.000179461	0.013797856	3	3
GO:00455	0.000179461	0.013797856	3	3
GO:00060	0.00018503	0.013978648	9	37
GO:00167	0.00018503	0.013978648	9	37
GO:00048	0.000188733	0.014135426	4	6

GO:00067	0.000195864	0.014331396	3	3
GO:00525	0.000195864	0.014331396	3	3
GO:00801	0.000196298	0.014331396	3	3
GO:00083	0.000205507	0.01487873	3	3
GO:00505	0.000223693	0.016008119	5	11
GO:00480	0.000224792	0.016008119	57	597
GO:00513	0.00023022	0.016206043	5	11
GO:00092	0.000232922	0.016206043	3	3
GO:00315	0.000236898	0.016206043	3	3
GO:00528	0.000236898	0.016206043	3	3
GO:00088	0.000236898	0.016206043	3	3
GO:00506	0.00026055	0.017684846	4	7
GO:00505	0.000270991	0.018250908	7	24
GO:00093	0.000283046	0.018916156	7	23
GO:00200	0.000289949	0.019229612	40	383
GO:00055	0.00030931	0.020358218	40	383
GO:00041	0.000325014	0.021231013	13	72
GO:00100	0.000352996	0.02288677	9	38
GO:00103	0.000408846	0.026311497	9	40
GO:00351	0.000448506	0.028651593	5	12
GO:00458	0.000516139	0.032731503	53	561
GO:00717	0.000525987	0.0331143	8	33
GO:00096	0.00059718	0.037325914	18	128
GO:00167	0.000611046	0.037772357	9	41
GO:00096	0.000613018	0.037772357	7	26
GO:00477	0.000667838	0.040860429	3	4
GO:00167	0.000712052	0.043260914	4	8
GO:00504	0.000734456	0.044312195	7	28
GO:00551	0.000741541	0.044431119	4	8
GO:00502	0.000756268	0.045003148	3	4
GO:00039	0.000773227	0.045699315	3	4
GO:00102	0.000788315	0.046276246	3	4
GO:00465	0.000803231	0.046835401	3	4
GO:00198	0.000815905	0.046986717	3	4
GO:00309	0.000816643	0.046986717	6	20
GO:00438	0.00088542	0.049704368	3	4
GO:00043	0.000890431	0.049704368	3	4
GO:00058	0.000890431	0.049704368	3	4
GO:00166	0.000890431	0.049704368	3	4
GO:00059	0.000892482	0.049704368	3	4
GO:00068	0.000902109	0.049920517	5	14

Table S16

**Category**	**P_value**	**Q_value**	**numDEInCat**	**numInCat**
ko04075	3.37E-11	4.41E-09	50	264
ko00350	1.97E-09	1.29E-07	20	59
ko00950	3.04E-08	1.33E-06	14	34
ko00073	2.63E-06	8.62E-05	14	47

ko00900	3.70E-05	0.00097039	13	50
ko00770	0.000524016	0.011441026	8	27
ko00010	0.000663247	0.012412186	19	115

Table S17

**Category**	**P_value**	**Q_value**	**numDEInCat**	**numInCat**
GO:00058	7.56E-176	6.57E-172	1,006	3,020
GO:00160	1.45E-122	6.32E-119	1,011	3,575
GO:00095	4.94E-112	1.43E-108	598	1,668
GO:00046	4.45E-84	9.67E-81	388	981
GO:00055	2.48E-71	4.30E-68	745	2,706
GO:00467	3.27E-70	4.74E-67	305	742
GO:00016	2.43E-64	3.01E-61	246	558
GO:00097	4.12E-64	4.47E-61	253	587
GO:00046	1.67E-63	1.62E-60	242	548
GO:00071	2.74E-63	2.38E-60	242	549
GO:00316	4.26E-61	3.37E-58	260	627
GO:00160	3.76E-58	2.72E-55	548	1,950
GO:00046	8.75E-58	5.85E-55	330	928
GO:00302	5.03E-30	3.12E-27	124	300
GO:00055	1.75E-26	1.01E-23	98	217
GO:00152	1.86E-25	1.01E-22	60	101
GO:00064	3.10E-24	1.58E-21	90	195
GO:00057	6.34E-23	3.06E-20	197	655
GO:00057	2.21E-22	1.01E-19	213	757
GO:00485	2.92E-22	1.27E-19	68	138
GO:00057	9.34E-20	3.87E-17	125	359
GO:00096	5.64E-18	2.23E-15	59	122
GO:00001	3.07E-16	1.16E-13	115	347
GO:00355	3.60E-16	1.30E-13	55	115
GO:00047	5.10E-16	1.77E-13	43	84
GO:00057	2.22E-15	7.43E-13	225	902
GO:00052	3.31E-14	1.07E-11	80	222
GO:00097	5.65E-14	1.75E-11	94	296
GO:00056	7.14E-14	2.14E-11	185	783
GO:00058	1.36E-13	3.93E-11	98	302
GO:00715	5.75E-13	1.61E-10	120	432
GO:00427	6.91E-13	1.87E-10	116	416
GO:00068	7.11E-13	1.87E-10	21	26
GO:00462	2.58E-12	6.58E-10	18	21
GO:00167	3.93E-12	9.75E-10	35	78
GO:00099	6.46E-12	1.56E-09	33	63
GO:00105	9.84E-12	2.31E-09	26	42
GO:00068	1.39E-11	3.18E-09	27	47
GO:00151	1.53E-11	3.41E-09	16	18
GO:00168	1.79E-11	3.88E-09	78	231
GO:00103	2.27E-11	4.80E-09	23	38
GO:00527	2.89E-11	5.97E-09	18	23

GO:00487	3.46E-11	6.99E-09	44	109
GO:00336	4.34E-11	8.58E-09	25	46
GO:00097	5.36E-11	1.04E-08	46	122
GO:00480	6.81E-11	1.29E-08	142	597
GO:00712	1.44E-10	2.66E-08	32	65
GO:00181	2.11E-10	3.82E-08	29	53
GO:00426	2.92E-10	5.18E-08	29	55
GO:00160	3.84E-10	6.59E-08	18	27
GO:00057	3.87E-10	6.59E-08	512	2,690
GO:00801	5.35E-10	8.94E-08	51	154
GO:00003	5.95E-10	9.75E-08	40	96
GO:00101	1.02E-09	1.65E-07	20	34
GO:00428	1.27E-09	2.00E-07	77	264
GO:00191	1.38E-09	2.14E-07	24	46
GO:00085	1.52E-09	2.31E-07	18	25
GO:00338	1.67E-09	2.50E-07	13	17
GO:00550	2.06E-09	3.03E-07	74	246
GO:00095	2.32E-09	3.35E-07	32	75
GO:00512	3.26E-09	4.65E-07	15	21
GO:00226	3.42E-09	4.79E-07	84	309
GO:00045	4.43E-09	6.10E-07	18	26
GO:00058	8.12E-09	1.10E-06	62	211
GO:00346	1.19E-08	1.59E-06	20	33
GO:00100	1.44E-08	1.87E-06	48	139
GO:00151	1.44E-08	1.87E-06	25	56
GO:00097	1.50E-08	1.91E-06	124	552
GO:00098	1.51E-08	1.91E-06	51	160
GO:00100	1.92E-08	2.39E-06	25	54
GO:00167	2.11E-08	2.58E-06	21	36
GO:00046	2.20E-08	2.65E-06	21	37
GO:00068	2.55E-08	3.04E-06	25	53
GO:00099	2.60E-08	3.05E-06	20	35
GO:00096	2.75E-08	3.18E-06	146	670
GO:00151	3.79E-08	4.28E-06	10	11
GO:00151	3.79E-08	4.28E-06	10	11
GO:00071	4.04E-08	4.50E-06	60	202
GO:00082	4.99E-08	5.49E-06	36	95
GO:00098	5.35E-08	5.81E-06	27	60
GO:00466	5.42E-08	5.82E-06	37	103
GO:00095	5.80E-08	6.15E-06	100	422
GO:00150	5.99E-08	6.27E-06	18	32
GO:00096	8.02E-08	8.27E-06	45	139
GO:00002	8.10E-08	8.27E-06	20	40
GO:00059	1.22E-07	1.23E-05	110	471
GO:00167	1.34E-07	1.32E-05	49	156
GO:00097	1.35E-07	1.32E-05	68	264
GO:00434	1.35E-07	1.32E-05	23	49
GO:00059	1.40E-07	1.35E-05	19	34

GO:00047	1.42E-07	1.35E-05	21	42
GO:00102	1.45E-07	1.35E-05	26	64
GO:00801	1.45E-07	1.35E-05	10	11
GO:00099	1.89E-07	1.75E-05	12	19
GO:00153	2.04E-07	1.86E-05	9	10
GO:00158	2.09E-07	1.89E-05	10	12
GO:00158	2.17E-07	1.95E-05	12	17
GO:00092	2.29E-07	2.03E-05	23	49
GO:00055	2.48E-07	2.18E-05	74	291
GO:00485	2.64E-07	2.30E-05	15	25
GO:00098	3.14E-07	2.70E-05	52	200
GO:00098	3.18E-07	2.71E-05	14	22
GO:00102	3.63E-07	3.06E-05	10	12
GO:00101	3.70E-07	3.09E-05	11	14
GO:00151	3.78E-07	3.13E-05	12	18
GO:00097	4.53E-07	3.72E-05	44	138
GO:00094	4.71E-07	3.82E-05	40	133
GO:00058	5.30E-07	4.27E-05	57	192
GO:00053	5.98E-07	4.77E-05	10	13
GO:00465	9.39E-07	7.41E-05	9	10
GO:00033	9.56E-07	7.41E-05	20	45
GO:00158	9.63E-07	7.41E-05	12	18
GO:00466	9.64E-07	7.41E-05	104	441
GO:00158	1.05E-06	7.98E-05	9	11
GO:00103	1.08E-06	8.15E-05	17	34
GO:00903	1.09E-06	8.20E-05	13	21
GO:00421	1.18E-06	8.74E-05	17	32
GO:00152	1.30E-06	9.59E-05	23	53
GO:00100	1.44E-06	0.000104988	35	107
GO:00052	1.49E-06	0.000107131	14	25
GO:00160	1.49E-06	0.000107131	51	181
GO:00436	1.69E-06	0.000120064	18	35
GO:00151	1.75E-06	0.000122463	9	12
GO:00068	1.76E-06	0.000122463	60	211
GO:00022	1.76E-06	0.000122463	23	57
GO:00517	1.83E-06	0.000126482	10	13
GO:00324	1.86E-06	0.000127458	39	132
GO:00096	1.94E-06	0.000131929	47	167
GO:00096	2.45E-06	0.00016507	76	330
GO:19026	2.64E-06	0.0001767	12	18
GO:00152	2.75E-06	0.000182399	34	104
GO:00454	2.89E-06	0.000190331	29	88
GO:00230	2.91E-06	0.000190353	22	52
GO:00096	3.01E-06	0.000194858	7	7
GO:00159	3.08E-06	0.000198194	31	89
GO:00800	3.12E-06	0.000199398	7	7
GO:00154	3.44E-06	0.000216882	7	7
GO:00053	3.44E-06	0.000216882	12	19

GO:0080(	3.69E-06	0.000230707	16	33
GO:0033!	3.86E-06	0.000239309	7	7
GO:0005:	4.46E-06	0.000271073	21	50
GO:0035²	4.46E-06	0.000271073	21	50
GO:0046:	4.46E-06	0.000271073	21	50
GO:0004(	4.67E-06	0.000281802	7	7
GO:0005!	5.24E-06	0.000313705	55	197
GO:0009{	5.46E-06	0.000324763	46	192
GO:0008²	5.85E-06	0.00034419	16	31
GO:0009!	5.91E-06	0.00034419	63	241
GO:00001	5.98E-06	0.00034419	8	9
GO:0003{	5.98E-06	0.00034419	8	9
GO:0006(	5.98E-06	0.00034419	8	9
GO:0035:	6.13E-06	0.000350188	25	85
GO:0046²	6.70E-06	0.000380488	9	12
GO:0090:	6.86E-06	0.000384194	11	16
GO:0008{	6.86E-06	0.000384194	13	25
GO:0016(	6.90E-06	0.000384194	23	60
GO:0006{	7.25E-06	0.000401427	11	17
GO:0052!	8.05E-06	0.000442499	19	48
GO:0004²	9.07E-06	0.000495868	65	283
GO:0043(	1.14E-05	0.000617307	18	42
GO:0052(	1.14E-05	0.000617307	30	116
GO:0022{	1.20E-05	0.000645395	20	49
GO:0005!	1.27E-05	0.000677376	13	26
GO:0015(	1.40E-05	0.000744123	10	16
GO:0004?	1.45E-05	0.000763765	21	51
GO:0005²	1.52E-05	0.000797568	13	26
GO:0005!	1.73E-05	0.000897744	8	10
GO:0030:	1.74E-05	0.000897744	13	26
GO:0006(	1.79E-05	0.000920982	37	116
GO:0071!	2.04E-05	0.001040475	12	21
GO:0031:	2.11E-05	0.001069741	40	156
GO:0000:	2.13E-05	0.001077205	6	6
GO:0008:	2.16E-05	0.001083647	11	18
GO:00151	2.33E-05	0.001157829	7	9
GO:0015{	2.33E-05	0.001157829	7	9
GO:0008:	2.51E-05	0.001225663	15	30
GO:0002?	2.54E-05	0.001225663	8	12
GO:0010:	2.54E-05	0.001225663	8	11
GO:0060!	2.54E-05	0.001225663	8	11
GO:0080(	2.56E-05	0.001225663	29	115
GO:0080(	2.56E-05	0.001225663	29	115
GO:0012!	2.57E-05	0.001225663	8	10
GO:0010:	3.16E-05	0.001498084	13	26
GO:0010!	3.24E-05	0.001531115	10	16
GO:0016:	3.33E-05	0.001565734	31	100
GO:0005:	3.62E-05	0.001690859	21	56

GO:0016	3.67E-05	0.001703234	9	13
GO:0048	3.89E-05	0.001799378	22	67
GO:0015	4.14E-05	0.001892095	7	9
GO:0035	4.14E-05	0.001892095	7	9
GO:1900	4.59E-05	0.002089898	10	19
GO:0007	4.79E-05	0.002149492	9	13
GO:0006	4.87E-05	0.002149492	6	7
GO:0015	4.87E-05	0.002149492	6	7
GO:0015	4.87E-05	0.002149492	6	7
GO:0015	4.87E-05	0.002149492	6	7
GO:0098	4.87E-05	0.002149492	6	7
GO:0009	5.03E-05	0.002197296	7	9
GO:0019	5.03E-05	0.002197296	7	9
GO:0009	6.06E-05	0.002632713	25	73
GO:0030	6.24E-05	0.002698213	31	94
GO:0009	6.38E-05	0.002743469	30	98
GO:1901	6.95E-05	0.002973867	6	8
GO:0009	7.33E-05	0.003122455	54	235
GO:0045	7.47E-05	0.003165783	5	5
GO:0030	7.53E-05	0.003174828	9	16
GO:0048	7.65E-05	0.003209579	11	23
GO:0043	7.94E-05	0.003315814	51	225
GO:0010	8.46E-05	0.003504444	14	32
GO:0002	8.47E-05	0.003504444	9	15
GO:0009	8.51E-05	0.003504444	68	307
GO:0016	9.13E-05	0.003739722	33	113
GO:0015	9.30E-05	0.00379456	7	10
GO:0010	9.65E-05	0.003916818	14	33
GO:0047	9.98E-05	0.004032799	5	5
GO:0010	0.000105357	0.00423769	6	7
GO:0009	0.000107276	0.004294994	52	229
GO:0001	0.000111657	0.004449882	18	52
GO:0008	0.000115091	0.004565789	29	98
GO:0048	0.000116319	0.004593534	11	25
GO:0090	0.000121161	0.004763097	7	9
GO:0050	0.000122431	0.004791342	4	4
GO:0050	0.00012362	0.004816204	9	16
GO:0034	0.000127587	0.004948549	6	7
GO:0005	0.000131772	0.005088148	6	7
GO:0006	0.000134691	0.005177869	9	15
GO:0005	0.000137401	0.005258783	413	2,333
GO:0009	0.000139215	0.005303496	25	80
GO:0035	0.000139791	0.005303496	5	5
GO:0004	0.000141829	0.005334227	9	15
GO:0070	0.000141829	0.005334227	9	15
GO:0016	0.000143338	0.005367771	5	5
GO:0060	0.000145956	0.005442334	13	29
GO:0010	0.00014816	0.005500927	15	40

GO:19023	0.000150058	0.00554767	5	5
GO:00801	0.000152884	0.00562819	5	5
GO:00102	0.000154994	0.005669898	10	20
GO:00102	0.000155334	0.005669898	5	5
GO:00713	0.000155974	0.005669898	5	5
GO:00352	0.000161731	0.005854647	7	11
GO:00056	0.000165813	0.005977542	31	110
GO:00098	0.000171474	0.006156046	20	55
GO:00482	0.000173504	0.006203293	11	22
GO:00157	0.00017734	0.006314451	21	66
GO:00085	0.000189671	0.006725967	4	4
GO:00060	0.00021778	0.007664162	8	13
GO:00096	0.000217892	0.007664162	35	128
GO:00354	0.000230731	0.008083045	10	19
GO:00340	0.000232833	0.008091417	6	8
GO:00525	0.000232833	0.008091417	6	8
GO:00068	0.000241862	0.008371698	28	90
GO:00905	0.000248627	0.008571705	6	8
GO:00102	0.000252743	0.008662866	8	13
GO:00477	0.000253692	0.008662866	4	4
GO:00082	0.000254262	0.008662866	22	66
GO:00422	0.000267452	0.009076657	4	4
GO:00314	0.000268983	0.009093096	22	66
GO:00099	0.000283258	0.00953855	9	17
GO:00101	0.000288383	0.009673629	38	146
GO:00052	0.000294927	0.009855089	12	25
GO:00047	0.000314035	0.01045341	19	55
GO:00603	0.000316608	0.010498828	7	11
GO:00082	0.000324775	0.010728685	7	10
GO:00423	0.000327016	0.01073487	15	38
GO:00097	0.000327433	0.01073487	7	11
GO:00002	0.000333676	0.010863331	51	205
GO:00712	0.000333852	0.010863331	19	59
GO:00315	0.000337398	0.010937734	8	15
GO:00094	0.000344596	0.011129557	89	447
GO:00100	0.000373974	0.01203364	11	24
GO:00163	0.00038	0.012182422	16	45
GO:00001	0.000383875	0.012261435	9	17
GO:00904	0.000396712	0.012625046	4	4
GO:00450	0.000403922	0.012807582	37	144
GO:00167	0.000426274	0.013442086	35	162
GO:00098	0.000427028	0.013442086	5	6
GO:00167	0.000433455	0.013595157	55	260
GO:00472	0.000435573	0.013612428	4	4
GO:00068	0.000441924	0.013761411	6	8
GO:00092	0.000463495	0.014333532	16	46
GO:19016	0.000463596	0.014333532	5	7
GO:00085	0.000467008	0.014387813	7	11

GO:00103	0.000476251	0.014620738	23	73
GO:00466	0.000501178	0.015295355	10	21
GO:00083	0.000501747	0.015295355	11	23
GO:00102	0.000524006	0.015918047	6	9
GO:00070	0.000539997	0.01627428	13	35
GO:00102	0.000540271	0.01627428	7	12
GO:19004	0.000541352	0.01627428	6	8
GO:00065	0.000548632	0.016419958	6	8
GO:00080	0.000549978	0.016419958	21	61
GO:00053	0.00055657	0.016503356	4	4
GO:00096	0.00055657	0.016503356	4	4
GO:00423	0.000563601	0.016654983	11	24
GO:00046	0.000568925	0.016698704	4	4
GO:00344	0.000568925	0.016698704	4	4
GO:00152	0.00058773	0.017192575	5	6
GO:00101	0.000590019	0.017201614	22	71
GO:19001	0.000595157	0.017293381	11	25
GO:00099	0.00060137	0.017378092	21	68
GO:00008	0.000602072	0.017378092	4	4
GO:00161	0.000608193	0.017496628	12	31
GO:00102	0.000612111	0.017551217	11	26
GO:00333	0.000627735	0.017881195	5	6
GO:00333	0.000627735	0.017881195	5	6
GO:00156	0.000641995	0.018168236	5	6
GO:00468	0.000641995	0.018168236	5	6
GO:00068	0.000666727	0.018806909	7	12
GO:00429	0.000677291	0.018859954	7	11
GO:00429	0.000677291	0.018859954	7	11
GO:00429	0.000677291	0.018859954	7	11
GO:00429	0.000677291	0.018859954	7	11
GO:00337	0.000695891	0.019315983	6	10
GO:00094	0.000700839	0.019391374	82	415
GO:00044	0.000712923	0.019663106	5	6
GO:00100	0.000725255	0.019939921	5	6
GO:00986	0.000731888	0.020058812	10	21
GO:00057	0.000779884	0.021307028	118	593
GO:00102	0.00079416	0.021629037	5	6
GO:00154	0.000798223	0.021671753	5	6
GO:00181	0.000810487	0.021924829	8	14
GO:00068	0.000812592	0.021924829	23	75
GO:00168	0.000836913	0.022511139	5	7
GO:00100	0.000841978	0.022577492	6	10
GO:00335	0.000848645	0.022686241	6	9
GO:00090	0.000852679	0.022724156	6	9
GO:00156	0.000881149	0.023295761	7	12
GO:00052	0.000884372	0.023295761	6	9
GO:00052	0.000887113	0.023295761	11	25
GO:00044	0.000892534	0.023295761	4	4

GO:00047	0.000894383	0.023295761	27	96
GO:00423	0.000900941	0.023295761	4	4
GO:00712	0.000900941	0.023295761	4	4
GO:00158	0.000900941	0.023295761	4	4
GO:00453	0.000900941	0.023295761	4	4
GO:19011	0.000900941	0.023295761	4	4
GO:00098	0.000910197	0.023465256	6	9
GO:00168	0.000914294	0.023501133	4	5
GO:00105	0.000930921	0.023817222	12	31
GO:00052	0.000932074	0.023817222	8	16
GO:00080	0.000940678	0.023966601	15	40
GO:00430	0.000945881	0.023998856	19	58
GO:00043	0.000950231	0.023998856	7	13
GO:00061	0.000950231	0.023998856	7	13
GO:00103	0.000961215	0.024205903	17	50
GO:00163	0.00099183	0.024904689	10	22
GO:00065	0.001015926	0.02543621	7	12
GO:00045	0.00102336	0.025546076	52	242
GO:00082	0.001026195	0.025546076	14	39
GO:00039	0.001037	0.02566797	3	3
GO:00060	0.001037	0.02566797	3	3
GO:00084	0.001054244	0.02602065	6	10
GO:00095	0.001066178	0.026240656	37	145
GO:00510	0.001075968	0.02640682	11	26
GO:00100	0.001132877	0.027725161	10	23
GO:00101	0.001140526	0.027833962	9	19
GO:00068	0.001150228	0.027968039	10	22
GO:00486	0.001152458	0.027968039	7	13
GO:00099	0.001177891	0.028505622	16	47
GO:00065	0.001182321	0.028533348	7	12
GO:00099	0.001211234	0.029150135	9	19
GO:00022	0.001221054	0.029257703	8	16
GO:00484	0.001222439	0.029257703	6	9
GO:00550	0.001249097	0.029741865	13	35
GO:00103	0.001249514	0.029741865	5	7
GO:00066	0.00126321	0.029985714	32	124
GO:00086	0.001268998	0.030041012	8	16
GO:00099	0.001297982	0.030641315	8	17
GO:00081	0.001301409	0.030641315	64	294
GO:00472	0.001327053	0.031160648	5	7
GO:00712	0.001377802	0.032265089	4	5
GO:00800	0.001395816	0.032511671	7	14
GO:00800	0.001395816	0.032511671	7	14
GO:00904	0.001409594	0.032744791	14	41
GO:19011	0.001430131	0.033133272	5	7
GO:00080	0.001453295	0.033491322	8	15
GO:00712	0.001453295	0.033491322	8	15
GO:00053	0.001467364	0.033726073	5	8

GO:00301	0.001499604	0.034324497	9	21
GO:00060	0.001503073	0.034324497	14	38
GO:00009	0.001505252	0.034324497	5	7
GO:00196	0.001516882	0.034499135	4	5
GO:00503	0.001552952	0.03522727	5	7
GO:00067	0.001615676	0.036554673	6	11
GO:00400	0.001657955	0.037314188	3	3
GO:00466	0.001657955	0.037314188	3	3
GO:00151	0.001662131	0.037314188	5	7
GO:00046	0.001748474	0.039151401	13	34
GO:00043	0.001783582	0.039834856	5	7
GO:00069	0.001817687	0.040419396	120	678
GO:00068	0.001829613	0.040419396	19	64
GO:00904	0.001833016	0.040419396	5	8
GO:00904	0.001833016	0.040419396	5	8
GO:19013	0.001833016	0.040419396	5	8
GO:00526	0.001840872	0.040489863	6	11
GO:00099	0.001898863	0.041659913	6	11
GO:00445	0.001905975	0.041710609	23	94
GO:00472	0.001935182	0.042243374	10	31
GO:00101	0.001958089	0.042575818	16	54
GO:00101	0.001960213	0.042575818	7	13
GO:00061	0.002015978	0.043677855	7	13
GO:00718	0.002073743	0.044817609	15	46
GO:00153	0.002124553	0.045801767	3	3
GO:00163	0.002134139	0.045894551	6	10
GO:00801	0.002159104	0.046316771	4	5
GO:00030	0.002228751	0.047693072	5	7
GO:00098	0.002235436	0.047718588	10	28
GO:00975	0.002247854	0.047866074	10	23
GO:00099	0.002275801	0.048342679	13	37
GO:00055	0.002343918	0.049668198	13	36

Table S18

**Category**	**P_value**	**Q_value**	**numDEInCat**	**numInCat**
ko04626	6.98E-09	9.14E-07	79	236
ko00520	6.12E-07	4.01E-05	48	124
ko00565	6.45E-06	0.000226438	16	26
ko04075	6.91E-06	0.000226438	75	264
ko00052	1.78E-05	0.000466397	27	62
ko00591	3.88E-05	0.000847414	9	11
ko00562	0.000141543	0.002648878	23	53
ko00500	0.00035492	0.005811815	72	251
ko00062	0.000603499	0.008784269	15	36
ko02010	0.001184455	0.015467687	15	33
ko04145	0.001298813	0.015467687	26	74

Table S19

Category	P_value	Q_value	numDEInCat	numInCat
GO:00094	2.33E-52	2.03E-48	39	66
GO:00102	2.41E-34	1.05E-30	51	322
GO:00068	5.86E-31	1.67E-27	20	26
GO:00160	7.68E-31	1.67E-27	152	3,575
GO:00037	5.46E-30	9.49E-27	84	1,185
GO:00462	1.35E-29	1.93E-26	18	21
GO:00097	1.56E-29	1.93E-26	43	264
GO:00426	2.83E-28	3.07E-25	24	55
GO:00527	3.31E-28	3.19E-25	18	23
GO:00160	4.31E-26	3.75E-23	18	27
GO:00098	9.44E-26	7.45E-23	23	59
GO:00063	3.53E-25	2.56E-22	89	1,552
GO:00085	6.20E-25	4.14E-22	17	25
GO:00103	4.49E-24	2.79E-21	19	38
GO:00003	7.17E-23	4.16E-20	25	96
GO:00097	9.14E-23	4.96E-20	39	307
GO:00063	1.12E-21	5.73E-19	89	1,745
GO:00095	1.27E-20	6.13E-18	85	1,668
GO:00100	4.31E-19	1.97E-16	23	107
GO:00097	8.28E-19	3.60E-16	47	587
GO:00055	1.90E-17	7.87E-15	106	2,706
GO:00016	2.09E-17	8.24E-15	44	558
GO:00058	3.08E-17	1.16E-14	113	3,020
GO:00483	4.23E-17	1.53E-14	29	230
GO:00046	3.23E-16	1.12E-13	42	548
GO:00071	3.44E-16	1.15E-13	42	549
GO:00169	1.20E-15	3.86E-13	12	24
GO:00480	1.27E-15	3.95E-13	43	597
GO:00467	1.57E-15	4.70E-13	48	742
GO:00096	4.71E-15	1.36E-12	11	20
GO:00096	4.89E-15	1.37E-12	21	128
GO:00102	1.42E-14	3.85E-12	10	16
GO:00046	1.87E-14	4.91E-12	54	981
GO:00099	2.35E-14	6.01E-12	17	80
GO:00100	2.67E-14	6.62E-12	21	139
GO:00316	3.32E-14	7.94E-12	42	627
GO:00068	3.38E-14	7.94E-12	10	17
GO:00458	9.09E-14	2.08E-11	39	561
GO:00044	1.15E-13	2.56E-11	11	25
GO:00525	9.66E-13	2.10E-10	13	48
GO:00105	1.19E-12	2.52E-10	9	16
GO:00458	2.49E-12	5.14E-10	9	17
GO:00027	3.23E-12	6.52E-10	8	12
GO:00045	4.95E-12	9.35E-10	12	43
GO:00060	4.95E-12	9.35E-10	12	43
GO:00080	4.95E-12	9.35E-10	12	43
GO:00080	3.99E-11	6.93E-09	8	15

GO:0031[illegible]	3.99E-11	6.93E-09	8	15
GO:0033[illegible]	3.99E-11	6.93E-09	8	15
GO:0071[illegible]	3.99E-11	6.93E-09	8	15
GO:0045[illegible]	6.18E-11	1.05E-08	23	251
GO:0004[illegible]	7.55E-11	1.26E-08	9	23
GO:0005[illegible]	2.01E-10	3.29E-08	40	757
GO:0004[illegible]	2.16E-10	3.48E-08	45	928
GO:0010[illegible]	2.83E-10	4.46E-08	9	26
GO:0000[illegible]	3.61E-10	5.60E-08	12	60
GO:0005[illegible]	1.44E-09	2.14E-07	19	197
GO:0010[illegible]	1.45E-09	2.14E-07	5	5
GO:0071[illegible]	1.45E-09	2.14E-07	5	5
GO:0004[illegible]	1.81E-09	2.62E-07	8	22
GO:0009[illegible]	4.20E-09	5.89E-07	9	34
GO:0010[illegible]	4.20E-09	5.89E-07	9	34
GO:0005[illegible]	5.85E-09	8.07E-07	8	25
GO:0010[illegible]	8.32E-09	1.11E-06	8	26
GO:0015[illegible]	8.60E-09	1.11E-06	5	6
GO:0015[illegible]	8.60E-09	1.11E-06	5	6
GO:0046[illegible]	8.60E-09	1.11E-06	5	6
GO:0006[illegible]	9.53E-09	1.18E-06	9	37
GO:0016[illegible]	9.53E-09	1.18E-06	9	37
GO:0043[illegible]	9.53E-09	1.18E-06	9	37
GO:0042[illegible]	1.58E-08	1.94E-06	26	416
GO:0010[illegible]	2.65E-08	3.20E-06	7	20
GO:0010[illegible]	3.17E-08	3.77E-06	9	42
GO:0004[illegible]	3.81E-08	4.42E-06	6	13
GO:00061	3.81E-08	4.42E-06	6	13
GO:0070[illegible]	6.66E-08	7.62E-06	8	33
GO:00191	7.37E-08	8.21E-06	9	46
GO:0033[illegible]	7.37E-08	8.21E-06	9	46
GO:0015[illegible]	8.56E-08	9.30E-06	4	4
GO:19011	8.56E-08	9.30E-06	4	4
GO:00161	1.89E-07	2.02E-05	9	51
GO:0009[illegible]	2.05E-07	2.16E-05	12	103
GO:0009[illegible]	2.06E-07	2.16E-05	7	26
GO:0070[illegible]	2.18E-07	2.25E-05	8	38
GO:0015[illegible]	3.61E-07	3.69E-05	11	89
GO:0042[illegible]	6.17E-07	5.95E-05	5	11
GO:0042[illegible]	6.17E-07	5.95E-05	5	11
GO:0042[illegible]	6.17E-07	5.95E-05	5	11
GO:0042[illegible]	6.17E-07	5.95E-05	5	11
GO:00801	6.17E-07	5.95E-05	5	11
GO:0009[illegible]	7.71E-07	7.34E-05	13	138
GO:0030[illegible]	7.78E-07	7.34E-05	6	20
GO:0046[illegible]	1.07E-06	0.000100254	6	21
GO:0005[illegible]	1.15E-06	0.000105904	30	655
GO:00101	1.50E-06	0.000136955	7	34

GO:00508	2.22E-06	0.000200658	20	343
GO:00197	2.34E-06	0.000209144	8	51
GO:00182	2.37E-06	0.000209704	9	68
GO:00096	2.74E-06	0.000240521	7	37
GO:00002	2.77E-06	0.000240521	15	205
GO:19011	2.88E-06	0.000247397	4	7
GO:00068	3.95E-06	0.000336491	15	211
GO:00095	5.43E-06	0.000457674	9	75
GO:00515	6.06E-06	0.000506486	9	76
GO:00098	6.73E-06	0.000556996	6	28
GO:00164	1.07E-05	0.000873981	22	447
GO:00056	1.46E-05	0.001186874	31	783
GO:00082	1.99E-05	0.001568124	3	4
GO:00506	1.99E-05	0.001568124	3	4
GO:00423	1.99E-05	0.001568124	3	4
GO:00160	2.23E-05	0.001742021	58	1,950
GO:00550	2.48E-05	0.001923007	15	246
GO:00103	2.57E-05	0.001956861	4	11
GO:00609	2.57E-05	0.001956861	4	11
GO:00068	3.55E-05	0.002683667	8	73
GO:00151	4.90E-05	0.003625985	3	5
GO:00153	4.90E-05	0.003625985	3	5
GO:00168	4.92E-05	0.003625985	14	231
GO:00103	5.24E-05	0.003825515	8	77
GO:00485	5.82E-05	0.004212223	5	25
GO:00125	6.31E-05	0.004528129	8	79
GO:00168	7.48E-05	0.005324595	4	14
GO:00068	7.66E-05	0.005381434	6	42
GO:00098	7.68E-05	0.005381434	9	104
GO:00161	9.68E-05	0.006618951	3	6
GO:19023	9.68E-05	0.006618951	3	6
GO:19023	9.68E-05	0.006618951	3	6
GO:00047	9.78E-05	0.006641319	8	84
GO:00096	0.000100097	0.006741434	6	44
GO:00096	0.00011059	0.007335797	9	109
GO:00102	0.000111262	0.007335797	7	64
GO:00094	0.000111455	0.007335797	19	415
GO:00197	0.000132298	0.008577676	4	16
GO:00062	0.000132298	0.008577676	4	16
GO:00096	0.000156014	0.010040376	9	114
GO:00096	0.000164266	0.010493681	6	48
GO:00154	0.000167164	0.010600887	3	7
GO:00159	0.000178091	0.011212014	9	116
GO:00483	0.00018727	0.011705058	11	171
GO:00096	0.000215428	0.013368867	8	94
GO:00096	0.000250618	0.015442343	25	670
GO:00152	0.000294373	0.017280477	2	2
GO:00422	0.000294373	0.017280477	2	2

GO:00713	0.000294373	0.017280477	2	2
GO:00040	0.000294373	0.017280477	2	2
GO:00061	0.000294373	0.017280477	2	2
GO:19024	0.000294373	0.017280477	2	2
GO:19024	0.000294373	0.017280477	2	2
GO:00069	0.000299407	0.017458014	25	678
GO:00150	0.00039105	0.022351573	3	9
GO:00705	0.00039105	0.022351573	3	9
GO:00049	0.00039105	0.022351573	3	9
GO:00052	0.000480884	0.027306653	12	222
GO:00301	0.000485726	0.027402544	7	81
GO:00090	0.000644693	0.036136099	9	138
GO:00094	0.000775503	0.043189538	18	447
GO:00161	0.000873063	0.045776124	2	3
GO:00432	0.000873063	0.045776124	2	3
GO:00800	0.000873063	0.045776124	2	3
GO:00800	0.000873063	0.045776124	2	3
GO:00429	0.000873063	0.045776124	2	3
GO:00469	0.000873063	0.045776124	2	3
GO:00719	0.000873063	0.045776124	2	3
GO:00719	0.000873063	0.045776124	2	3
GO:00423	0.000873063	0.045776124	2	3
GO:00450	0.000874636	0.045776124	9	144

Table S20

Category	P_value	Q_value	numDEInCat	numInCat
ko04075	6.65E-29	8.71E-27	45	264
ko02010	3.11E-15	2.04E-13	14	33
ko04626	1.25E-08	5.46E-07	22	236
ko00190	0.000127426	0.004173201	11	125

Table S21

Cluster	Record	Type	From	To
Cluster 17	scaffold383	Alkaloid	1	389,285
Cluster 21	scaffold67	Alkaloid	2,978,547	3,622,210
Cluster 1	scaffold13	Lignan-Polyketide	981,159	1,376,309
Cluster 23	scaffold8	Polyketide	4,529,957	5,812,302
Cluster 2	scaffold132	Putative	34,174	689,685
Cluster 4	scaffold154	Putative	2,232,746	2,490,786
Cluster 9	scaffold268	Putative	1,079,703	2,003,831
Cluster 12	scaffold317	Putative	295,028	941,249
Cluster 14	scaffold338	Putative	760,835	1,070,430
Cluster 5	scaffold160	Saccharide	580,781	1,087,231
Cluster 6	scaffold170	Saccharide	1,847,925	2,973,087
Cluster 10	scaffold28	Saccharide	511,674	1,729,579
Cluster 13	scaffold33	Saccharide	7,536,717	8,466,817
Cluster 15	scaffold34	Saccharide	5,724,863	6,208,948
Cluster 16	scaffold36	Saccharide	2,373,331	2,926,426

Cluster 18	scaffold394	Saccharide	578,831	793,915
Cluster 20	scaffold54	Saccharide	7,265,034	8,021,036
Cluster 22	scaffold73	Saccharide	1,429,564	2,050,061
Cluster 8	scaffold21	Saccharide-Polyketide	9,844,715	10,494,316
Cluster 3	scaffold148	Terpene	234,014	673,747
Cluster 7	scaffold175	Terpene	582,938	1,206,986
Cluster 11	scaffold304	Terpene	1,065,427	1,391,381
Cluster 19	scaffold42	Terpene	1,159,540	1,602,377

Library_strategy	Library_s	Library_s	Library_l	Platform	Instrume	Sample	Geologica
WGS	GENOMI	RANDOM	single	PACBIO_	PacBio Se	leaves	China:Gua
WGS	GENOMI	RANDOM	paired	ILLUMIN	HiSeq X T	leaves	China:Gua
RNA-Seq	TRANSC	RANDOM	paired	ILLUMIN	Illumina H	fruits	China:Fur
RNA-Seq	TRANSC	RANDOM	paired	ILLUMIN	Illumina H	fruits	China:Fur
RNA-Seq	TRANSC	RANDOM	paired	ILLUMIN	Illumina H	fruits	China:Fur
RNA-Seq	TRANSC	RANDOM	paired	ILLUMIN	Illumina H	fruits	China:Fur
RNA-Seq	TRANSC	RANDOM	paired	ILLUMIN	Illumina H	leaves	China:Fur
RNA-Seq	TRANSC	RANDOM	paired	ILLUMIN	Illumina H	leaves	China:Ley
RNA-Seq	TRANSC	RANDOM	paired	ILLUMIN	Illumina H	seeds	China:Ley

>=5000
450
8,090,850
1.80%
17,979.67

Repeat rate (%)	Error rate (%)
54.60826	0.33621

L50	Max. leng	Gene completeness (%)
381	9.05 Mb	86.00%
2,968	1.37 Mb	NA
631	3.73 Mb	NA
646	4.94 Mb	NA
674	4.5 Mb	85.20%
538	5.13 Mb	79.90%
380	6.72 Mb	71.80%
885	3.52 Mb	56.10%
9397/277*	570 Kb/9.	90.00%
380	6.7 Mb	88.30%
380/94*	6.7 Mb/25	88.60%
376/94*	6.7 Mb/25	90.60%

Mean_length (bp)
901.62
468.85
870.62
835.01
204.73
994.93
622.69
352.98

623.29
304.10
392.34
759.44
144.23
136.51
145.77
442.06
95.85
528.98
895.77
194.05
283.27
409.00
349.15
743.69
743.69
366.52
478.68
197.81
117.92
253.75
560.89

**Total length of tr**	**Average l**	**N50 of transcripts (bp)**
36,893,680	702.62	938
28,915,705	814.69	1,136
61,063,687	1,447.83	2,003
65,681,138	1,146.29	1,760

**Average CDS Le**	**Average l**	**Average l**	**Average Intron Length (bp)**
1281.10	5.97	244.46	650.22

Proportion of genome (%)
6.99
0.19
0.23
0.23
0.16
0.04
0.01
0.01
0.01
0.01
0.01
0.01
0.01
0.01
0.00
0.00
0.00
0.00
0.00
0.00
0.00
0.00
0.00
0.00
0.00
0.00
0.01
7.94
3.77
1.16
0.32
0.26
0.02
0.01
0.01
0.01
0.01
0.01

0.01
0.00
0.00
0.00
0.00
0.00
0.00
5.59

sest gene

1,001-10,000 bp	10,001-10	100,001-1	Total
3,682	6,389	384	12,135
111	83	5	451
99	138	16	341
54	179	22	329
69	117	19	311
11	19	2	85
13	11	0	25
6	12	2	23
5	7	0	19
6	4	0	13
5	4	0	13
3	5	1	12
4	5	0	11
3	6	0	9
1	5	0	7
2	4	0	7
1	3	0	7
1	1	0	3
1	0	0	3
0	1	0	2
0	1	0	1
0	1	0	1
0	1	0	1
0	0	0	1
1	0	0	1
1,151	3,662	625	5,982
963	711	33	2,883
224	190	25	747
154	115	5	679
6	17	3	32
7	7	0	24
4	6	1	17
3	2	0	16
4	3	0	14
3	3	0	13
1	7	0	13

1	3	2	9
4	2	0	8
3	1	0	6
1	1	0	6
0	0	0	2
0	1	0	1

S*+*T	***I*+*S*+*T***	**Filtered s**	**Filtered *I***	**Filtered S**	**Filtered T**	**Filtered S**	**Filtered T**
425	662	0	237	222	203	0.94	0.86
12,166	14,009	0	1,843	9,337	2,829	5.07	1.54
10,491	13,844	70	3,203	7,116	2,209	2.22	0.69
64,015	68,862	900	4,777	52,980	8,269	11.09	1.73
7,086	8,376	2,000	1,098	3,827	1,730	3.49	1.58
28,614	32,513	0	3,899	22,775	5,839	5.84	1.50
15,490	16,987	50	1,485	13,020	2,256	8.77	1.52
13,422	15,833	0	2,411	9,738	3,684	4.04	1.53
64,366	89,026	0	24,660	56,170	8,196	2.28	0.33

**Scaffold N50 (Mb)**	**References**
2	[1]
44	[2]
3	[3]
23	[4]
20	[5]
49	[6]
40	[7]
34	[8]
22	[9]
58	[10, 11]
66	[12]
38	[13]
178	[14]
29	[15]

et al. The genome of the recently domesticated crop plant sugar beet (Beta vulgaris). Nature.
ilegia genome: adaptive radiation and an extraordinarily polymorphic chromosome with a uni
ing sacred lotus (Nelumbo nucifera Gaertn.). Genome Biology. 2013;14 5:R41. doi:10.1186/g
e grapevine genome sequence suggests ancestral hexaploidization in major angiosperm phyla.
enome of black cottonwood, Populus trichocarpa (Torr. & Gray). Science. 2006;313 5793:159
ence of the palaeopolyploid soybean. Nature. 2010;463 7278:178-83. doi:10.1038/nature0867
nitive, wild and cultivated citrus provide insights into asexual reproduction. Nature Genetics.
genome sequence of the most widely cultivated cacao type and its use to identify candidate g

). Araport11: a complete reannotation of the Arabidopsis thaliana reference genome. Plant Jou
. The genome of Eucalyptus grandis. Nature. 2014. doi:10.1038/nature13308.
;h-resolution genetic maps of Eucalyptus improve Eucalyptus grandis genome assembly. New
leshy fruit evolution. Nature. 2012;485 7400:635-41.
ıe coffee genome provides insight into the convergent evolution of caffeine biosynthesis. Scie
genome provides insights into oil metabolism, flowering and Asterid evolution. Nature. 201'
Genome Annotation Resource: improvements and new features. Nucleic Acids Research. 20

Duplicated genes
14,514
15,322
12,197
14,306
10,728
19,975
43,778
46,852
10,568
12,733
21,302
27,601
16,916
12,462
10,000

Term	Class	Gene ID
reticuline oxidase	molecular	Maole_000947.T1, Maole_000949.T1, Maole_000952.T1, Ma
response to xenobi	biological	Maole_005120.T1, Maole_005178.T1, Maole_005179.T1, Ma
response to brassir	biological	Maole_002978.T1, Maole_003404.T1, Maole_003405.T1, Ma
UDP-N-acetylmur	molecular	Maole_000947.T1, Maole_000949.T1, Maole_000952.T1, Ma
mitochondrial mR	biological	Maole_000261.T1, Maole_001054.T1, Maole_002026.T1, Ma
chloroplast RNA n	biological	Maole_003493.T1, Maole_004679.T1, Maole_005107.T1, Ma
cytoplasmic memb	cellular_c	Maole_000947.T1, Maole_000949.T1, Maole_000952.T1, Ma
embryonic pattern	biological	Maole_000378.T1, Maole_000437.T1, Maole_000677.T1, Ma
chloroplast RNA p	biological	Maole_000089.T1, Maole_001995.T1, Maole_004679.T1, Ma
mitochondrial RN	biological	Maole_001054.T1, Maole_005145.T1, Maole_005991.T1, Ma
flavin adenine din	molecular	Maole_000947.T1, Maole_000949.T1, Maole_000952.T1, Ma
mitochondrion	cellular_c	Maole_000261.T1, Maole_000438.T1, Maole_000439.T1, Ma
pollen tube tip	cellular_c	Maole_005499.T1, Maole_007741.T1, Maole_009345.T1, Ma
filiform apparatus	cellular_c	Maole_003404.T1, Maole_003485.T1, Maole_005499.T1, Ma
unidimensional cel	biological	Maole_000015.T1, Maole_001212.T1, Maole_001213.T1, Ma
response to chitin	biological	Maole_000378.T1, Maole_002027.T1, Maole_003161.T1, Ma
alkaloid metabolic	biological	Maole_000947.T1, Maole_000949.T1, Maole_000952.T1, Ma
cytidine to uridine	biological	Maole_001054.T1, Maole_002538.T1, Maole_005087.T1, Ma
single fertilization	biological	Maole_005499.T1, Maole_007741.T1, Maole_011302.T1, Ma
RNA modification	biological	Maole_002026.T1, Maole_005087.T1, Maole_005107.T1, Ma

pollen tube recepti	biological	Maole_003404.T1, Maole_003485.T1, Maole_005499.T1, Ma
sodium ion transpo	biological	Maole_003069.T1, Maole_003305.T1, Maole_003945.T1, Ma
extracellular regio	cellular_c	Maole_000596.T1, Maole_002245.T1, Maole_002247.T1, Ma
alkane 1-monooxy	molecular	Maole_002169.T1, Maole_002172.T1, Maole_002180.T1, Ma
brassinosteroid me	biological	Maole_004856.T1, Maole_005111.T1, Maole_012033.T1, Ma
ADP binding	molecular	Maole_000688.T1, Maole_000715.T1, Maole_000726.T1, Ma
cell wall macromo	biological	Maole_020579.T1, Maole_023238.T1, Maole_023239.T1, Ma
monovalent cation	molecular	Maole_007592.T1, Maole_007594.T1, Maole_007595.T1, Ma
aromatic-L-amino-	molecular	Maole_021238.T1, Maole_023059.T1, Maole_023061.T1, Ma
tyrosine decarboxy	molecular	Maole_021238.T1, Maole_023059.T1, Maole_023061.T1, Ma
mannitol dehydrog	molecular	Maole_005882.T1, Maole_005883.T1, Maole_005885.T1, Ma
fruit ripening	biological	Maole_002319.T1, Maole_002322.T1, Maole_002329.T1, Ma
establishment or m	biological	Maole_003069.T1, Maole_003305.T1, Maole_003945.T1, Ma
defense response	biological	Maole_000688.T1, Maole_000715.T1, Maole_000726.T1, Ma
oxidoreductase act	molecular	Maole_000212.T1, Maole_000369.T1, Maole_001766.T1, Ma
regulation of trans	biological	Maole_000003.T1, Maole_000378.T1, Maole_000437.T1, Ma
mRNA processing	biological	Maole_001611.T1, Maole_002269.T1, Maole_003493.T1, Ma
alpha,alpha-trehal	molecular	Maole_001031.T1, Maole_006121.T1, Maole_006541.T1, Ma
transcription, DNA	biological	Maole_000003.T1, Maole_000378.T1, Maole_000437.T1, Ma
vacuole	cellular_c	Maole_000947.T1, Maole_000949.T1, Maole_000952.T1, Ma
oxidoreductase act	molecular	Maole_002169.T1, Maole_002172.T1, Maole_002180.T1, Ma
root development	biological	Maole_000378.T1, Maole_000437.T1, Maole_000677.T1, Ma
geraniol dehydroge	molecular	Maole_005886.T1, Maole_005890.T1, Maole_005893.T1, Ma
response to biotic	biological	Maole_012895.T1, Maole_012897.T1, Maole_019956.T1, Ma
xylem and phloem	biological	Maole_000378.T1, Maole_000437.T1, Maole_000677.T1, Ma
hydrogen-transloca	molecular	Maole_003069.T1, Maole_003305.T1, Maole_003945.T1, Ma
anchored compone	cellular_c	Maole_000947.T1, Maole_000949.T1, Maole_000952.T1, Ma
response to auxin	biological	Maole_000378.T1, Maole_000437.T1, Maole_000677.T1, Ma
pH reduction	biological	Maole_001461.T1, Maole_004639.T1, Maole_015166.T1, Ma
monooxygenase ac	molecular	Maole_002169.T1, Maole_002172.T1, Maole_002180.T1, Ma
apical plasma mem	cellular_c	Maole_005499.T1, Maole_007741.T1, Maole_011302.T1, Ma
trehalose-phosphat	molecular	Maole_001031.T1, Maole_006121.T1, Maole_006541.T1, Ma
intracellular memb	cellular_c	Maole_002169.T1, Maole_002172.T1, Maole_002319.T1, Ma
chloroplast	cellular_c	Maole_000089.T1, Maole_000156.T1, Maole_000158.T1, Ma
regulation of pH	biological	Maole_007592.T1, Maole_009013.T1, Maole_013459.T1, Ma
xyloglucan-specifi	molecular	Maole_002978.T1, Maole_003782.T1, Maole_003783.T1, Ma
inorganic diphospl	molecular	Maole_003069.T1, Maole_003305.T1, Maole_003945.T1, Ma
auxin-activated sig	biological	Maole_000378.T1, Maole_000437.T1, Maole_000677.T1, Ma
DNA metabolic pr	biological	Maole_000788.T1, Maole_000790.T1, Maole_006224.T1, Ma
very long-chain fa	biological	Maole_002180.T1, Maole_002181.T1, Maole_002183.T1, Ma
negative regulatior	biological	Maole_000439.T1, Maole_005499.T1, Maole_014799.T1, Ma
aromatase activity	molecular	Maole_002180.T1, Maole_002181.T1, Maole_002183.T1, Ma
diamine oxidase ac	molecular	Maole_001779.T1, Maole_001782.T1, Maole_001785.T1, Ma
cellular response t	biological	Maole_001779.T1, Maole_001782.T1, Maole_001785.T1, Ma
xenobiotic catabol	biological	Maole_004561.T1, Maole_013805.T1, Maole_013806.T1, Ma
transmembrane sig	molecular	Maole_001461.T1, Maole_004639.T1, Maole_015166.T1, Ma
chitin binding	molecular	Maole_020579.T1, Maole_023238.T1, Maole_023239.T1, Ma
trehalose biosynth	biological	Maole_001031.T1, Maole_006121.T1, Maole_006541.T1, Ma

chitinase activity	molecular	Maole_020579.T1, Maole_023238.T1, Maole_023239.T1, Ma
chitin catabolic pro	biological	Maole_020579.T1, Maole_023238.T1, Maole_023239.T1, Ma
cytosol	cellular_c	Maole_000015.T1, Maole_000369.T1, Maole_000596.T1, Ma
tryptamine:oxygen	molecular	Maole_001779.T1, Maole_001782.T1, Maole_001785.T1, Ma
aminoacetone:oxyg	molecular	Maole_001779.T1, Maole_001782.T1, Maole_001785.T1, Ma
aliphatic-amine ox	molecular	Maole_001779.T1, Maole_001782.T1, Maole_001785.T1, Ma
phenethylamine:ox	molecular	Maole_001779.T1, Maole_001782.T1, Maole_001785.T1, Ma
nitric oxide biosyn	biological	Maole_001779.T1, Maole_001782.T1, Maole_001785.T1, Ma
potassium ion tran	biological	Maole_007592.T1, Maole_007594.T1, Maole_007595.T1, Ma
polycistronic mRN	biological	Maole_000089.T1, Maole_001995.T1, Maole_005631.T1, Ma
cinnamyl-alcohol d	molecular	Maole_005882.T1, Maole_005883.T1, Maole_005885.T1, Ma
sinapyl alcohol deh	molecular	Maole_005882.T1, Maole_005883.T1, Maole_005885.T1, Ma
transcription facto	molecular	Maole_000003.T1, Maole_000378.T1, Maole_000437.T1, Ma
signal transduction	biological	Maole_003404.T1, Maole_003483.T1, Maole_003485.T1, Ma
3-deoxy-7-phospho	molecular	Maole_001279.T1, Maole_007364.T1, Maole_018830.T1, Ma
primary amine oxi	molecular	Maole_001779.T1, Maole_001782.T1, Maole_001785.T1, Ma
sporopollenin bios	biological	Maole_002180.T1, Maole_002183.T1, Maole_005259.T1, Ma
endoplasmic reticu	cellular_c	Maole_002172.T1, Maole_002180.T1, Maole_002181.T1, Ma
exocyst	cellular_c	Maole_000596.T1, Maole_002809.T1, Maole_004342.T1, Ma
carbohydrate phos	biological	Maole_000911.T1, Maole_001047.T1, Maole_008161.T1, Ma
carboxy-lyase acti	molecular	Maole_021238.T1, Maole_023059.T1, Maole_023061.T1, Ma
cellular potassium	biological	Maole_009013.T1, Maole_019829.T1, Maole_019870.T1, Ma
glucosyltransferase	molecular	Maole_005111.T1, Maole_012033.T1, Maole_012034.T1, Ma
6-phosphofructoki	molecular	Maole_000911.T1, Maole_001047.T1, Maole_008161.T1, Ma
auxin polar transpo	biological	Maole_003052.T1, Maole_003069.T1, Maole_003305.T1, Ma
cellular response to	biological	Maole_008096.T1, Maole_008097.T1, Maole_008098.T1, Ma
quercetin 3-O-gluc	molecular	Maole_004561.T1, Maole_004613.T1, Maole_004614.T1, Ma
quercetin 7-O-gluc	molecular	Maole_004561.T1, Maole_004613.T1, Maole_004614.T1, Ma
long-chain-alcohol	molecular	Maole_010272.T1, Maole_010273.T1, Maole_010274.T1, Ma
regulation of salicy	biological	Maole_002999.T1, Maole_003050.T1, Maole_012089.T1, Ma
flavonoid glucuron	biological	Maole_004561.T1, Maole_004613.T1, Maole_004614.T1, Ma
photosystem I	cellular_c	Maole_000212.T1, Maole_015203.T1, Maole_021341.T1, Ma
post-embryonic de	biological	Maole_003404.T1, Maole_003485.T1, Maole_005499.T1, Ma
fructose 6-phospha	biological	Maole_000911.T1, Maole_001047.T1, Maole_008161.T1, Ma
water homeostasis	biological	Maole_009013.T1, Maole_019829.T1, Maole_019870.T1, Ma
guiding stereospec	molecular	Maole_019956.T1, Maole_019959.T1, Maole_019963.T1, Ma
(-)-pinoresinol bio	biological	Maole_019956.T1, Maole_019959.T1, Maole_019963.T1, Ma
quercetin 4'-O-gluc	molecular	Maole_005111.T1, Maole_012033.T1, Maole_012034.T1, Ma
response to oxidati	biological	Maole_000947.T1, Maole_000949.T1, Maole_000952.T1, Ma
amidase activity	molecular	Maole_003538.T1, Maole_003540.T1, Maole_010786.T1, Ma
polysaccharide cat	biological	Maole_020579.T1, Maole_023238.T1, Maole_023239.T1, Ma
polar nucleus	cellular_c	Maole_011635.T1, Maole_013907.T1, Maole_013908.T1, Ma
negative regulation	biological	Maole_000378.T1, Maole_000437.T1, Maole_000439.T1, Ma
phytyl diphosphate	biological	Maole_020036.T1, Maole_022028.T1, Maole_022954.T1
geranylgeranyl red	molecular	Maole_020036.T1, Maole_022028.T1, Maole_022954.T1
cellular glucan me	biological	Maole_002978.T1, Maole_003782.T1, Maole_003783.T1, Ma
xyloglucan:xylogl	molecular	Maole_002978.T1, Maole_003782.T1, Maole_003783.T1, Ma
uroporphyrinogen	molecular	Maole_002758.T1, Maole_010530.T1, Maole_010532.T1, Ma

terpenoid metaboli	biological	Maole_002319.T1, Maole_023205.T1, Maole_023211.T1
(+)-menthofuran sy	molecular	Maole_002319.T1, Maole_023205.T1, Maole_023211.T1
stomium developm	biological	Maole_000003.T1, Maole_023892.T1, Maole_024107.T1
steroid hydroxylas	molecular	Maole_004856.T1, Maole_021133.T1, Maole_021135.T1
hydroquinone gluc	molecular	Maole_004561.T1, Maole_013805.T1, Maole_013806.T1, Mao
apoplast	cellular_c	Maole_000947.T1, Maole_000949.T1, Maole_000952.T1, Mao
cellular response to	biological	Maole_008096.T1, Maole_008097.T1, Maole_008098.T1, Mao
ribonucleotide met	biological	Maole_000369.T1, Maole_001766.T1, Maole_015853.T1
plasma membrane	cellular_c	Maole_009345.T1, Maole_012742.T1, Maole_022416.T1
phosphatidylinosit	molecular	Maole_009345.T1, Maole_012742.T1, Maole_022416.T1
4-hydroxy-tetrahyd	molecular	Maole_004967.T1, Maole_016326.T1, Maole_016327.T1
limonoid glucosylt	molecular	Maole_005715.T1, Maole_015708.T1, Maole_015709.T1, Mao
cis-zeatin O-beta-I	molecular	Maole_005111.T1, Maole_012033.T1, Maole_012034.T1, Mao
amine metabolic p	biological	Maole_001779.T1, Maole_001782.T1, Maole_001785.T1, Mao
heme binding	molecular	Maole_002169.T1, Maole_002172.T1, Maole_002180.T1, Mao
iron ion binding	molecular	Maole_002169.T1, Maole_002172.T1, Maole_002180.T1, Mao
serine-type carbox	molecular	Maole_005171.T1, Maole_007127.T1, Maole_009867.T1, Mao
root epidermal cell	biological	Maole_000911.T1, Maole_001047.T1, Maole_007908.T1, Mao
suberin biosynthet	biological	Maole_002180.T1, Maole_002181.T1, Maole_002183.T1, Mao
siRNA binding	molecular	Maole_001429.T1, Maole_005194.T1, Maole_006987.T1, Mao
positive regulation	biological	Maole_002030.T1, Maole_002032.T1, Maole_002805.T1, Mao
cellular response to	biological	Maole_008096.T1, Maole_008097.T1, Maole_008098.T1, Mao
plant-type hyperse	biological	Maole_002245.T1, Maole_002247.T1, Maole_002253.T1, Mao
phosphotransferase	molecular	Maole_008872.T1, Maole_010839.T1, Maole_010840.T1, Mao
response to mecha	biological	Maole_002978.T1, Maole_003786.T1, Maole_003790.T1, Mao
chlorophyllase acti	molecular	Maole_013425.T1, Maole_013426.T1, Maole_013428.T1
transferase activity	molecular	Maole_005614.T1, Maole_012177.T1, Maole_012178.T1, Mao
trans-zeatin O-beta	molecular	Maole_005111.T1, Maole_012033.T1, Maole_012034.T1, Mao
L-proline biosynth	biological	Maole_006145.T1, Maole_017439.T1, Maole_017441.T1, Mao
rubber cis-polypren	molecular	Maole_012177.T1, Maole_012178.T1, Maole_015768.T1
acetolactate syntha	molecular	Maole_004372.T1, Maole_004375.T1, Maole_004377.T1
response to vitamin	biological	Maole_023402.T1, Maole_023780.T1, Maole_023782.T1
S-methyl-5-thiorib	molecular	Maole_015699.T1, Maole_015702.T1, Maole_015705.T1
thymine metabolic	biological	Maole_007877.T1, Maole_007878.T1, Maole_016334.T1
potassium ion binc	molecular	Maole_003069.T1, Maole_003305.T1, Maole_003945.T1, Mao
phosphatidylinosit	molecular	Maole_009345.T1, Maole_012742.T1, Maole_022416.T1
enoyl-[acyl-carrier	molecular	Maole_008716.T1, Maole_008717.T1, Maole_022203.T1
fatty acid synthase	cellular_c	Maole_008716.T1, Maole_008717.T1, Maole_022203.T1
enoyl-[acyl-carrier	molecular	Maole_008716.T1, Maole_008717.T1, Maole_022203.T1
ribonucleoside-dip	cellular_c	Maole_000369.T1, Maole_001766.T1, Maole_015853.T1
xenobiotic metabo	biological	Maole_004561.T1, Maole_013805.T1, Maole_013806.T1, Mao

**Term**	**Class**	**Gene ID**
Plant hormone sign	Environm	Maole_000378.T1, Maole_000437.T1, Maole_000677.T1, Mao
Tyrosine metabolis	Metabolis	Maole_001779.T1, Maole_001782.T1, Maole_001785.T1, Mao
Isoquinoline alkalo	Metabolis	Maole_001779.T1, Maole_001782.T1, Maole_001785.T1, Mao
Cutin, suberine and	Metabolis	Maole_002180.T1, Maole_002181.T1, Maole_002183.T1, Mao

Terpenoid backbor	Metabolis	Maole_004288.T1, Maole_005614.T1, Maole_010786.T1, Ma
Pantothenate and (	Metabolis	Maole_004372.T1, Maole_004375.T1, Maole_004377.T1, Ma
Glycolysis/Glucon	Metabolis	Maole_000911.T1, Maole_001047.T1, Maole_001348.T1, Ma

**Term**	**Class**	**Gene ID**
plasma membrane	cellular_c	Maole_000080.T1, Maole_000100.T1, Maole_000104.T1, Ma
integral componen	cellular_c	Maole_000042.T1, Maole_000059.T1, Maole_000080.T1, Ma
plasmodesma	cellular_c	Maole_000026.T1, Maole_000059.T1, Maole_000065.T1, Ma
protein serine/thre	molecular	Maole_000080.T1, Maole_000100.T1, Maole_000328.T1, Ma
ATP binding	molecular	Maole_000026.T1, Maole_000080.T1, Maole_000100.T1, Ma
protein autophospl	biological	Maole_000080.T1, Maole_000100.T1, Maole_000379.T1, Ma
peptide receptor ac	molecular	Maole_000080.T1, Maole_000100.T1, Maole_000379.T1, Ma
hormone-mediated	biological	Maole_000080.T1, Maole_000100.T1, Maole_000379.T1, Ma
transmembrane rec	molecular	Maole_000080.T1, Maole_000100.T1, Maole_000379.T1, Ma
transmembrane rec	biological	Maole_000080.T1, Maole_000100.T1, Maole_000379.T1, Ma
ubiquitin protein li	molecular	Maole_000080.T1, Maole_000100.T1, Maole_000379.T1, Ma
membrane	cellular_c	Maole_000099.T1, Maole_000132.T1, Maole_000311.T1, Ma
protein kinase acti	molecular	Maole_000080.T1, Maole_000100.T1, Maole_000328.T1, Ma
carbohydrate bindi	molecular	Maole_000065.T1, Maole_000080.T1, Maole_000100.T1, Ma
calmodulin binding	molecular	Maole_000080.T1, Maole_000100.T1, Maole_000517.T1, Ma
symporter activity	molecular	Maole_000211.T1, Maole_001403.T1, Maole_001533.T1, Ma
protein phosphoryl	biological	Maole_000698.T1, Maole_001017.T1, Maole_001137.T1, Ma
vacuolar membran	cellular_c	Maole_000311.T1, Maole_000390.T1, Maole_000747.T1, Ma
vacuole	cellular_c	Maole_000390.T1, Maole_000465.T1, Maole_000558.T1, Ma
recognition of poll	biological	Maole_000080.T1, Maole_000100.T1, Maole_000517.T1, Ma
endosome	cellular_c	Maole_000059.T1, Maole_000384.T1, Maole_000473.T1, Ma
response to nemato	biological	Maole_000400.T1, Maole_000474.T1, Maole_000613.T1, Ma
Golgi membrane	cellular_c	Maole_000059.T1, Maole_000104.T1, Maole_000474.T1, Ma
intracellular signal	biological	Maole_002742.T1, Maole_005388.T1, Maole_006437.T1, Ma
transmembrane rec	molecular	Maole_000473.T1, Maole_001301.T1, Maole_001631.T1, Ma
Golgi apparatus	cellular_c	Maole_000026.T1, Maole_000059.T1, Maole_000384.T1, Ma
transporter activity	molecular	Maole_000104.T1, Maole_000455.T1, Maole_000747.T1, Ma
abscisic acid-activ	biological	Maole_001237.T1, Maole_001426.T1, Maole_001561.T1, Ma
cell wall	cellular_c	Maole_000065.T1, Maole_000311.T1, Maole_000465.T1, Ma
trans-Golgi networ	cellular_c	Maole_000059.T1, Maole_000384.T1, Maole_000735.T1, Ma
cell wall organizat	biological	Maole_000432.T1, Maole_000465.T1, Maole_000473.T1, Ma
defense response t	biological	Maole_000285.T1, Maole_000473.T1, Maole_000737.T1, Ma
drug transmembrai	biological	Maole_001019.T1, Maole_001020.T1, Maole_001021.T1, Ma
lignin catabolic pr	biological	Maole_002588.T1, Maole_002636.T1, Maole_002645.T1, Ma
transferase activity	molecular	Maole_002124.T1, Maole_002411.T1, Maole_003085.T1, Ma
cell tip growth	biological	Maole_000104.T1, Maole_000311.T1, Maole_000432.T1, Ma
basipetal auxin tra	biological	Maole_001432.T1, Maole_004047.T1, Maole_004980.T1, Ma
amino acid transpo	biological	Maole_001848.T1, Maole_002556.T1, Maole_002862.T1, Ma
nitrate transmembı	molecular	Maole_001538.T1, Maole_002816.T1, Maole_003203.T1, Ma
ATPase activity	molecular	Maole_000026.T1, Maole_000636.T1, Maole_001019.T1, Ma
regulation of anior	biological	Maole_002102.T1, Maole_004972.T1, Maole_006779.T1, Ma
hydroquinone:oxy	molecular	Maole_002588.T1, Maole_002636.T1, Maole_002645.T1, Ma

root hair elongatio	biological	Maole_000104.T1, Maole_000432.T1, Maole_000473.T1, Ma
receptor serine/thr	molecular	Maole_001301.T1, Maole_001631.T1, Maole_002777.T1, Ma
brassinosteroid me	biological	Maole_000411.T1, Maole_000473.T1, Maole_001301.T1, Ma
apoplast	cellular_c	Maole_000065.T1, Maole_000099.T1, Maole_000311.T1, Ma
cellular response t	biological	Maole_002461.T1, Maole_003601.T1, Maole_003602.T1, Ma
peptidyl-serine ph	biological	Maole_001017.T1, Maole_001137.T1, Maole_002747.T1, Ma
ATPase activity, c	molecular	Maole_000390.T1, Maole_001096.T1, Maole_001545.T1, Ma
detection of bacter	biological	Maole_004972.T1, Maole_006779.T1, Maole_009317.T1, Ma
cytoplasm	cellular_c	Maole_000121.T1, Maole_000304.T1, Maole_000416.T1, Ma
response to karriki	biological	Maole_001123.T1, Maole_001124.T1, Maole_001125.T1, Ma
plant-type vacuole	cellular_c	Maole_000390.T1, Maole_000806.T1, Maole_001096.T1, Ma
pollen maturation	biological	Maole_001301.T1, Maole_001631.T1, Maole_002950.T1, Ma
identical protein b	molecular	Maole_000473.T1, Maole_000724.T1, Maole_001017.T1, Ma
transmembrane rec	molecular	Maole_000698.T1, Maole_002191.T1, Maole_002469.T1, Ma
xenobiotic-transpo	molecular	Maole_000390.T1, Maole_001096.T1, Maole_001545.T1, Ma
icosanoyl-CoA syn	molecular	Maole_002411.T1, Maole_003085.T1, Maole_004215.T1, Ma
transmembrane tra	biological	Maole_000390.T1, Maole_001317.T1, Maole_001390.T1, Ma
microsporogenesis	biological	Maole_001301.T1, Maole_001631.T1, Maole_002469.T1, Ma
protein polymeriza	biological	Maole_002056.T1, Maole_002627.T1, Maole_003562.T1, Ma
cytosolic ribosome	cellular_c	Maole_000099.T1, Maole_000945.T1, Maole_001948.T1, Ma
beta-galactosidase	molecular	Maole_000065.T1, Maole_001106.T1, Maole_001209.T1, Ma
integral componen	cellular_c	Maole_000211.T1, Maole_001533.T1, Maole_001610.T1, Ma
cellular protein lo	biological	Maole_002747.T1, Maole_003593.T1, Maole_004156.T1, Ma
endosome membra	cellular_c	Maole_000473.T1, Maole_000886.T1, Maole_001108.T1, Ma
amino acid transm	molecular	Maole_002556.T1, Maole_002862.T1, Maole_004301.T1, Ma
response to abscisi	biological	Maole_000099.T1, Maole_000285.T1, Maole_001020.T1, Ma
pollen tube growth	biological	Maole_000738.T1, Maole_001163.T1, Maole_002401.T1, Ma
wax biosynthetic p	biological	Maole_000498.T1, Maole_001277.T1, Maole_002411.T1, Ma
cellulose synthase	molecular	Maole_000497.T1, Maole_001360.T1, Maole_002136.T1, Ma
calmodulin-depen	molecular	Maole_000328.T1, Maole_005388.T1, Maole_007462.T1, Ma
oligopeptide trans	biological	Maole_000747.T1, Maole_000749.T1, Maole_002591.T1, Ma
calcium-dependen	molecular	Maole_005388.T1, Maole_007462.T1, Maole_009364.T1, Ma
response to salt str	biological	Maole_000099.T1, Maole_000390.T1, Maole_000672.T1, Ma
acidic amino acid	molecular	Maole_006692.T1, Maole_006693.T1, Maole_006694.T1, Ma
neutral amino acid	molecular	Maole_006692.T1, Maole_006693.T1, Maole_006694.T1, Ma
signal transductio	biological	Maole_000379.T1, Maole_000381.T1, Maole_001017.T1, Ma
lipid binding	molecular	Maole_001229.T1, Maole_001301.T1, Maole_001631.T1, Ma
multidimensional	biological	Maole_000432.T1, Maole_000473.T1, Maole_002239.T1, Ma
anchored compone	cellular_c	Maole_000311.T1, Maole_000313.T1, Maole_000465.T1, Ma
plant-type cell wal	cellular_c	Maole_000065.T1, Maole_000311.T1, Maole_000313.T1, Ma
potassium ion tran	molecular	Maole_004582.T1, Maole_006036.T1, Maole_006829.T1, Ma
response to fungus	biological	Maole_000619.T1, Maole_001375.T1, Maole_001481.T1, Ma
polysaccharide bio	biological	Maole_000432.T1, Maole_000473.T1, Maole_002239.T1, Ma
carbohydrate meta	biological	Maole_000065.T1, Maole_000465.T1, Maole_001106.T1, Ma
transferase activity	molecular	Maole_000162.T1, Maole_000384.T1, Maole_000418.T1, Ma
auxin-activated sig	biological	Maole_000060.T1, Maole_001538.T1, Maole_001907.T1, Ma
anthocyanin accun	biological	Maole_000432.T1, Maole_000473.T1, Maole_002239.T1, Ma
cell cortex	cellular_c	Maole_000432.T1, Maole_000687.T1, Maole_002202.T1, Ma

receptor signaling	molecular	Maole_002461.T1, Maole_002779.T1, Maole_002780.T1, Mac
floral organ abscis	biological	Maole_001631.T1, Maole_001757.T1, Maole_002762.T1, Mac
abscisic acid transı	biological	Maole_005385.T1, Maole_009883.T1, Maole_012520.T1, Mac
fatty acid elongase	molecular	Maole_002411.T1, Maole_004215.T1, Maole_005654.T1, Mac
primary active trar	molecular	Maole_006692.T1, Maole_006693.T1, Maole_006694.T1, Mac
neutral amino acid	biological	Maole_006692.T1, Maole_006693.T1, Maole_006694.T1, Mac
peptide transport	biological	Maole_002591.T1, Maole_006581.T1, Maole_006583.T1, Mac
DNA mediated tra	biological	Maole_001126.T1, Maole_001161.T1, Maole_002043.T1, Mac
calcium ion bindin	molecular	Maole_000162.T1, Maole_000270.T1, Maole_000328.T1, Mac
organ developmen	biological	Maole_000162.T1, Maole_000418.T1, Maole_000995.T1, Mac
ethylene-activated	biological	Maole_001561.T1, Maole_001907.T1, Maole_002297.T1, Mac
auxin metabolic pr	biological	Maole_003601.T1, Maole_003602.T1, Maole_003604.T1, Mac
stem vascular tissu	biological	Maole_001277.T1, Maole_003007.T1, Maole_003101.T1, Mac
mucilage biosynth	biological	Maole_002689.T1, Maole_004880.T1, Maole_005453.T1, Mac
monosaccharide tr	molecular	Maole_000211.T1, Maole_001533.T1, Maole_002510.T1, Mac
plant-type vacuole	cellular_c	Maole_000806.T1, Maole_001120.T1, Maole_001432.T1, Mac
response to light st	biological	Maole_001422.T1, Maole_002411.T1, Maole_002826.T1, Mac
microtubule	cellular_c	Maole_000026.T1, Maole_000636.T1, Maole_000687.T1, Mac
L-glutamate transn	molecular	Maole_006693.T1, Maole_009102.T1, Maole_012082.T1, Mac
alpha-L-arabinofu	molecular	Maole_002406.T1, Maole_006066.T1, Maole_007409.T1, Mac
amino acid transm	biological	Maole_002556.T1, Maole_002862.T1, Maole_004301.T1, Mac
basic amino acid tı	biological	Maole_002707.T1, Maole_003886.T1, Maole_006692.T1, Mac
response to cadmi	biological	Maole_000099.T1, Maole_000490.T1, Maole_000643.T1, Mac
acidic amino acid	biological	Maole_006692.T1, Maole_006693.T1, Maole_006694.T1, Mac
auxin efflux	biological	Maole_004980.T1, Maole_004982.T1, Maole_008447.T1, Mac
stomatal closure	biological	Maole_001037.T1, Maole_001488.T1, Maole_007143.T1, Mac
nitrate assimilatior	biological	Maole_001538.T1, Maole_002401.T1, Maole_003203.T1, Mac
drug transmembrai	molecular	Maole_001013.T1, Maole_001120.T1, Maole_002207.T1, Mac
regulation of meris	biological	Maole_002288.T1, Maole_002831.T1, Maole_002854.T1, Mac
structural constitu	molecular	Maole_002627.T1, Maole_003562.T1, Maole_006933.T1, Mac
viral process	biological	Maole_001356.T1, Maole_001853.T1, Maole_002102.T1, Mac
cortical microtubu	biological	Maole_000432.T1, Maole_000497.T1, Maole_000687.T1, Mac
oligopeptide transı	molecular	Maole_007599.T1, Maole_009063.T1, Maole_009534.T1, Mac
transport	biological	Maole_001019.T1, Maole_001020.T1, Maole_001021.T1, Mac
response to molecı	biological	Maole_000379.T1, Maole_000381.T1, Maole_000473.T1, Mac
mannan synthase a	molecular	Maole_002136.T1, Maole_002689.T1, Maole_006085.T1, Mac
2-alkenal reductas	molecular	Maole_000698.T1, Maole_001025.T1, Maole_001533.T1, Mac
response to bacteri	biological	Maole_000285.T1, Maole_000619.T1, Maole_001561.T1, Mac
response to wound	biological	Maole_000121.T1, Maole_001071.T1, Maole_001123.T1, Mac
hydrogen ion trans	biological	Maole_001120.T1, Maole_002816.T1, Maole_004003.T1, Mac
antiporter activity	molecular	Maole_000474.T1, Maole_001013.T1, Maole_001120.T1, Mac
pectin catabolic pr	biological	Maole_000612.T1, Maole_000619.T1, Maole_001481.T1, Mac
signal transductior	biological	Maole_002461.T1, Maole_003283.T1, Maole_003557.T1, Mac
potassium:sodium	molecular	Maole_017648.T1, Maole_017866.T1, Maole_017867.T1, Mac
proton transport	biological	Maole_000211.T1, Maole_001467.T1, Maole_001533.T1, Mac
cutin transport	biological	Maole_001277.T1, Maole_003007.T1, Maole_015027.T1, Mac
organic phosphona	molecular	Maole_001020.T1, Maole_003007.T1, Maole_009336.T1, Mac
calcium-transporti	molecular	Maole_000806.T1, Maole_002899.T1, Maole_005633.T1, Mac

regulation of polle	biological	Maole_002950.T1, Maole_003848.T1, Maole_005388.T1, Ma
beta-apiosyl-beta-g	molecular	Maole_004819.T1, Maole_018863.T1, Maole_018864.T1, Ma
glucose transmemb	molecular	Maole_000211.T1, Maole_001533.T1, Maole_002313.T1, Ma
hexose transmemb	biological	Maole_000211.T1, Maole_001533.T1, Maole_002313.T1, Ma
glucose import	biological	Maole_000211.T1, Maole_001533.T1, Maole_002313.T1, Ma
phospholipid-trans	molecular	Maole_001136.T1, Maole_001730.T1, Maole_005514.T1, Ma
copper ion binding	molecular	Maole_000311.T1, Maole_000313.T1, Maole_000672.T1, Ma
flavonoid biosynth	biological	Maole_001120.T1, Maole_001286.T1, Maole_001288.T1, Ma
beta-glucosidase a	molecular	Maole_002141.T1, Maole_002146.T1, Maole_004819.T1, Ma
pollen developmen	biological	Maole_000026.T1, Maole_001404.T1, Maole_001488.T1, Ma
1,3-beta-D-glucan	cellular_c	Maole_003848.T1, Maole_004268.T1, Maole_011987.T1, Ma
1,3-beta-D-glucan	molecular	Maole_003848.T1, Maole_004268.T1, Maole_011987.T1, Ma
(1->3)-beta-D-gluc	biological	Maole_003848.T1, Maole_004268.T1, Maole_011987.T1, Ma
UDP-glucosyltrans	molecular	Maole_001286.T1, Maole_001288.T1, Maole_001920.T1, Ma
phosphatidylcholir	biological	Maole_001100.T1, Maole_001616.T1, Maole_008809.T1, Ma
regulation of stom	biological	Maole_001432.T1, Maole_001610.T1, Maole_001616.T1, Ma
cellulase activity	molecular	Maole_000613.T1, Maole_002235.T1, Maole_003315.T1, Ma
cell growth	biological	Maole_000432.T1, Maole_000497.T1, Maole_001100.T1, Ma
receptor-mediated	biological	Maole_004230.T1, Maole_004972.T1, Maole_009317.T1, Ma
defense response b	biological	Maole_001020.T1, Maole_001021.T1, Maole_001022.T1, Ma
monooxygenase ac	molecular	Maole_000411.T1, Maole_000498.T1, Maole_001534.T1, Ma
amino acid import	biological	Maole_000390.T1, Maole_002707.T1, Maole_002862.T1, Ma
flavonoid glucuror	biological	Maole_001286.T1, Maole_001288.T1, Maole_001919.T1, Ma
substrate-specific t	molecular	Maole_000211.T1, Maole_001533.T1, Maole_002313.T1, Ma
calcium-dependent	molecular	Maole_000162.T1, Maole_000418.T1, Maole_000995.T1, Ma
ammonium transpo	biological	Maole_000400.T1, Maole_002056.T1, Maole_002707.T1, Ma
protein tyrosine ki	molecular	Maole_001017.T1, Maole_004246.T1, Maole_005326.T1, Ma
steroid binding	molecular	Maole_000473.T1, Maole_002721.T1, Maole_002724.T1, Ma
phosphatidylinosit	molecular	Maole_001616.T1, Maole_005612.T1, Maole_008809.T1, Ma
cellulose catabolic	biological	Maole_000613.T1, Maole_002235.T1, Maole_003315.T1, Ma
glycolytic process	biological	Maole_000099.T1, Maole_000643.T1, Maole_000644.T1, Ma
cell periphery	cellular_c	Maole_000902.T1, Maole_002742.T1, Maole_003056.T1, Ma
anchored compone	cellular_c	Maole_000311.T1, Maole_000313.T1, Maole_000465.T1, Ma
spliceosomal comp	biological	Maole_002505.T1, Maole_004814.T1, Maole_007923.T1, Ma
sulfate transport	biological	Maole_004916.T1, Maole_004918.T1, Maole_005101.T1, Ma
L-alanine transmer	molecular	Maole_006693.T1, Maole_009102.T1, Maole_019159.T1, Ma
L-alanine transpor	biological	Maole_006693.T1, Maole_009102.T1, Maole_019159.T1, Ma
regulation of cell s	biological	Maole_001017.T1, Maole_001137.T1, Maole_002747.T1, Ma
immune response-i	biological	Maole_006779.T1, Maole_015116.T1, Maole_021416.T1, Ma
auxin influx transn	molecular	Maole_002556.T1, Maole_004982.T1, Maole_006799.T1, Ma
auxin influx	biological	Maole_002556.T1, Maole_004982.T1, Maole_006799.T1, Ma
quercetin 3-O-gluc	molecular	Maole_001286.T1, Maole_001288.T1, Maole_001919.T1, Ma
quercetin 7-O-gluc	molecular	Maole_001286.T1, Maole_001288.T1, Maole_001919.T1, Ma
trans-Golgi networ	cellular_c	Maole_001561.T1, Maole_002693.T1, Maole_006351.T1, Ma
auxin efflux transn	molecular	Maole_004980.T1, Maole_004982.T1, Maole_011110.T1, Ma
acropetal auxin tra	biological	Maole_004980.T1, Maole_004982.T1, Maole_011110.T1, Ma
dephosphorylation	biological	Maole_000132.T1, Maole_000326.T1, Maole_001093.T1, Ma
sugar:proton symp	molecular	Maole_000211.T1, Maole_001533.T1, Maole_002313.T1, Ma

oxidoreductase act	molecular	Maole_007386.T1, Maole_007927.T1, Maole_010760.T1, Ma
stamen developme	biological	Maole_000379.T1, Maole_000381.T1, Maole_001757.T1, Ma
L-proline transmer	molecular	Maole_006693.T1, Maole_009102.T1, Maole_012845.T1, Ma
proline transmemb	biological	Maole_006693.T1, Maole_009102.T1, Maole_012845.T1, Ma
(-)-secologanin bic	biological	Maole_001286.T1, Maole_001288.T1, Maole_010554.T1, Ma
male meiosis cytol	biological	Maole_000026.T1, Maole_004136.T1, Maole_004457.T1, Ma
glutamine transpor	biological	Maole_006693.T1, Maole_009102.T1, Maole_020423.T1, Ma
L-glutamine transr	molecular	Maole_006693.T1, Maole_009102.T1, Maole_020423.T1, Ma
L-serine transmem	molecular	Maole_006693.T1, Maole_009102.T1, Maole_020423.T1, Ma
L-serine transport	biological	Maole_006693.T1, Maole_009102.T1, Maole_020423.T1, Ma
L-glutamate impor	biological	Maole_006693.T1, Maole_009102.T1, Maole_020423.T1, Ma
cytokinin catabolic	biological	Maole_000803.T1, Maole_009962.T1, Maole_016181.T1, Ma
cytokinin dehydro	molecular	Maole_000803.T1, Maole_009962.T1, Maole_016181.T1, Ma
positive regulation	biological	Maole_000473.T1, Maole_001725.T1, Maole_005301.T1, Ma
cellulose biosynth	biological	Maole_000497.T1, Maole_001360.T1, Maole_002136.T1, Ma
defense response t	biological	Maole_001616.T1, Maole_001879.T1, Maole_003326.T1, Ma
arsenate ion transn	biological	Maole_001403.T1, Maole_015757.T1, Maole_015759.T1, Ma
response to jasmo	biological	Maole_000839.T1, Maole_000932.T1, Maole_001310.T1, Ma
phenylalanine amn	molecular	Maole_008291.T1, Maole_014706.T1, Maole_014707.T1, Ma
pectate lyase activ	molecular	Maole_004255.T1, Maole_005335.T1, Maole_008961.T1, Ma
floral organ develo	biological	Maole_003557.T1, Maole_003905.T1, Maole_007894.T1, Ma
intracellular memb	cellular_c	Maole_000886.T1, Maole_001286.T1, Maole_001288.T1, Ma
cotyledon vascular	biological	Maole_001277.T1, Maole_002596.T1, Maole_003007.T1, Ma
protease binding	molecular	Maole_007143.T1, Maole_007145.T1, Maole_011377.T1, Ma
response to auxin	biological	Maole_001907.T1, Maole_001918.T1, Maole_003496.T1, Ma
cellular response t	biological	Maole_000959.T1, Maole_001093.T1, Maole_001100.T1, Ma
monosaccharide tr	biological	Maole_002510.T1, Maole_010427.T1, Maole_011595.T1, Ma
negative regulatio	biological	Maole_002693.T1, Maole_005101.T1, Maole_006559.T1, Ma
galactinol-sucrose	molecular	Maole_006391.T1, Maole_011709.T1, Maole_015629.T1, Ma
auxin binding	molecular	Maole_002556.T1, Maole_006799.T1, Maole_009289.T1, Ma
response to ethyle	biological	Maole_000285.T1, Maole_001310.T1, Maole_001561.T1, Ma
vasculature develo	biological	Maole_000379.T1, Maole_002191.T1, Maole_002288.T1, Ma
carbohydrate trans	biological	Maole_000211.T1, Maole_000474.T1, Maole_001533.T1, Ma
meristem develop	biological	Maole_000803.T1, Maole_009962.T1, Maole_014592.T1, Ma
regulation of cell a	biological	Maole_002874.T1, Maole_004885.T1, Maole_006080.T1, Ma
UDP-glucuronate	molecular	Maole_007035.T1, Maole_009796.T1, Maole_016082.T1, Ma
defense response t	biological	Maole_000612.T1, Maole_000619.T1, Maole_001481.T1, Ma
lipid oxidation	biological	Maole_007386.T1, Maole_007927.T1, Maole_010760.T1, Ma
U2-type spliceoso	cellular_c	Maole_002505.T1, Maole_007910.T1, Maole_011216.T1, Ma
cellular zinc ion h	biological	Maole_001108.T1, Maole_004072.T1, Maole_005633.T1, Ma
cytosol	cellular_c	Maole_000026.T1, Maole_000381.T1, Maole_000490.T1, Ma
auxin polar transp	biological	Maole_002389.T1, Maole_002556.T1, Maole_003905.T1, Ma
gene silencing by	biological	Maole_002276.T1, Maole_004874.T1, Maole_005229.T1, Ma
phospholipase D a	molecular	Maole_001100.T1, Maole_001616.T1, Maole_008809.T1, Ma
N-acylphosphatidy	molecular	Maole_001100.T1, Maole_001616.T1, Maole_008809.T1, Ma
linoleate 13S-lipo	molecular	Maole_007386.T1, Maole_007927.T1, Maole_010763.T1, Ma
negative regulatio	biological	Maole_000473.T1, Maole_001631.T1, Maole_002777.T1, Ma
suberin biosynthet	biological	Maole_002558.T1, Maole_004409.T1, Maole_005654.T1, Ma

sulfate transmemb	biological	Maole_004916.T1, Maole_004918.T1, Maole_013416.T1, Ma
petal epidermis pa	biological	Maole_003007.T1, Maole_015027.T1, Maole_022244.T1, Ma
anther wall tapetu	biological	Maole_002469.T1, Maole_002782.T1, Maole_007101.T1, Ma
chlorophyll catabo	molecular	Maole_007944.T1, Maole_013780.T1, Maole_020501.T1, Ma
cellular response t	biological	Maole_001020.T1, Maole_001021.T1, Maole_001022.T1, Ma
UDP-xylosyltransf	molecular	Maole_003148.T1, Maole_005232.T1, Maole_013116.T1, Ma
extracellular space	cellular_c	Maole_000311.T1, Maole_000313.T1, Maole_000803.T1, Ma
pollen germination	biological	Maole_000738.T1, Maole_002136.T1, Maole_002950.T1, Ma
gametophyte devel	biological	Maole_000026.T1, Maole_003557.T1, Maole_004136.T1, Ma
nitrate transport	biological	Maole_001538.T1, Maole_001971.T1, Maole_002365.T1, Ma
ammonium transm	molecular	Maole_000400.T1, Maole_002056.T1, Maole_002707.T1, Ma
glyoxylate cycle	biological	Maole_005071.T1, Maole_008489.T1, Maole_008800.T1, Ma
plant-type hyperse	biological	Maole_000724.T1, Maole_002381.T1, Maole_002477.T1, Ma
phosphate ion tran	biological	Maole_001403.T1, Maole_001971.T1, Maole_002264.T1, Ma
germacradienol sy	molecular	Maole_010832.T1, Maole_013319.T1, Maole_013321.T1, Ma
germacrene-D syn	molecular	Maole_010832.T1, Maole_013319.T1, Maole_013321.T1, Ma
endocytosis	biological	Maole_000738.T1, Maole_000886.T1, Maole_001017.T1, Ma
plant epidermis de	biological	Maole_010336.T1, Maole_012100.T1, Maole_014337.T1, Ma
maintenance of se	biological	Maole_001120.T1, Maole_007749.T1, Maole_018033.T1, Ma
butyrate-CoA liga	molecular	Maole_005058.T1, Maole_005059.T1, Maole_009587.T1, Ma
cell death	biological	Maole_001561.T1, Maole_001631.T1, Maole_002408.T1, Ma
1-aminocycloprop	biological	Maole_008122.T1, Maole_017911.T1, Maole_023406.T1, Ma
oxylipin biosynthe	biological	Maole_000121.T1, Maole_001422.T1, Maole_002003.T1, Ma
histidine phosphot	molecular	Maole_001025.T1, Maole_001826.T1, Maole_005101.T1, Ma
stomatal complex	biological	Maole_000379.T1, Maole_000381.T1, Maole_000440.T1, Ma
calcium channel a	molecular	Maole_001424.T1, Maole_004073.T1, Maole_005877.T1, Ma
non-membrane spa	molecular	Maole_002461.T1, Maole_004238.T1, Maole_004602.T1, Ma
rejection of self p	biological	Maole_000518.T1, Maole_000519.T1, Maole_014480.T1, Ma
secondary active s	molecular	Maole_004916.T1, Maole_004918.T1, Maole_013416.T1, Ma
cuticle developme	biological	Maole_000864.T1, Maole_002411.T1, Maole_003085.T1, Ma
detection of brass	biological	Maole_000473.T1, Maole_006040.T1, Maole_011930.T1, Ma
magnesium ion bi	molecular	Maole_000870.T1, Maole_001038.T1, Maole_001136.T1, Ma
cellular response t	biological	Maole_002693.T1, Maole_007391.T1, Maole_007455.T1, Ma
motile cilium	cellular_c	Maole_004972.T1, Maole_014265.T1, Maole_014267.T1, Ma
response to cold	biological	Maole_000643.T1, Maole_000644.T1, Maole_001316.T1, Ma
proanthocyanidin	biological	Maole_001120.T1, Maole_001467.T1, Maole_001469.T1, Ma
apical plasma mem	cellular_c	Maole_000738.T1, Maole_002596.T1, Maole_004798.T1, Ma
protein phosphata	cellular_c	Maole_001091.T1, Maole_002389.T1, Maole_002874.T1, Ma
phloem nitrate loa	biological	Maole_017085.T1, Maole_017086.T1, Maole_017087.T1, Ma
innate immune res	biological	Maole_001424.T1, Maole_001749.T1, Maole_001757.T1, Ma
transferase activity	molecular	Maole_001286.T1, Maole_001288.T1, Maole_001919.T1, Ma
jasmonic acid and	biological	Maole_004457.T1, Maole_013450.T1, Maole_017660.T1, Ma
oxidoreductase act	molecular	Maole_000498.T1, Maole_001534.T1, Maole_001535.T1, Ma
13-hydroxylupinin	molecular	Maole_004810.T1, Maole_015819.T1, Maole_019477.T1, Ma
cellular copper ion	biological	Maole_001108.T1, Maole_004072.T1, Maole_007427.T1, Ma
response to tempe	biological	Maole_006933.T1, Maole_007143.T1, Maole_007145.T1, Ma
arsenate ion trans	molecular	Maole_015757.T1, Maole_015759.T1, Maole_015760.T1, Ma
hydrogen-exportin	molecular	Maole_001467.T1, Maole_001469.T1, Maole_004003.T1, Ma

lateral root formati	biological	Maole_000211.T1, Maole_001533.T1, Maole_002056.T1, Ma
response to copper	biological	Maole_002636.T1, Maole_002648.T1, Maole_004626.T1, Ma
galactosyltransfera	molecular	Maole_001227.T1, Maole_003589.T1, Maole_013231.T1, Ma
glucose mediated s	biological	Maole_001037.T1, Maole_008181.T1, Maole_011198.T1, Ma
microtubule-based	biological	Maole_002627.T1, Maole_003562.T1, Maole_004598.T1, Ma
endomembrane sys	biological	Maole_000384.T1, Maole_001488.T1, Maole_007183.T1, Ma
regulation of defer	biological	Maole_001561.T1, Maole_002693.T1, Maole_009038.T1, Ma
methionine metabo	biological	Maole_002874.T1, Maole_004885.T1, Maole_007956.T1, Ma
microtubule bindir	molecular	Maole_000636.T1, Maole_001957.T1, Maole_002202.T1, Ma
high-affinity hydro	molecular	Maole_011678.T1, Maole_016696.T1, Maole_021905.T1, Ma
hexose:proton sym	molecular	Maole_011678.T1, Maole_016696.T1, Maole_021905.T1, Ma
regulation of mem	biological	Maole_001610.T1, Maole_004073.T1, Maole_004862.T1, Ma
phospholipase C a	molecular	Maole_001516.T1, Maole_004385.T1, Maole_016666.T1, Ma
phosphatidylcholir	molecular	Maole_001516.T1, Maole_004385.T1, Maole_016666.T1, Ma
fatty acid transport	molecular	Maole_001277.T1, Maole_003007.T1, Maole_018563.T1, Ma
regulation of stom	biological	Maole_001467.T1, Maole_002389.T1, Maole_005195.T1, Ma
regulation of seedl	biological	Maole_000473.T1, Maole_001631.T1, Maole_002777.T1, Ma
polarity specificati	biological	Maole_002191.T1, Maole_002288.T1, Maole_002854.T1, Ma
inositol hexakisph	molecular	Maole_009289.T1, Maole_010021.T1, Maole_022187.T1, Ma
diterpenoid biosyn	biological	Maole_012497.T1, Maole_012499.T1, Maole_012501.T1, Ma
defense response s	biological	Maole_003356.T1, Maole_006211.T1, Maole_006779.T1, Ma
quercetin O-glucos	biological	Maole_004819.T1, Maole_018863.T1, Maole_018864.T1, Ma
kaempferol O-gluc	biological	Maole_004819.T1, Maole_018863.T1, Maole_018864.T1, Ma
lead ion transport	biological	Maole_012520.T1, Maole_012595.T1, Maole_012596.T1, Ma
terpenoid transport	biological	Maole_012520.T1, Maole_012595.T1, Maole_012596.T1, Ma
nucleotide transpo	biological	Maole_002707.T1, Maole_003886.T1, Maole_006692.T1, Ma
dipeptide transport	molecular	Maole_000747.T1, Maole_001986.T1, Maole_001987.T1, Ma
tripeptide transpor	molecular	Maole_000747.T1, Maole_001986.T1, Maole_001987.T1, Ma
dipeptide transport	biological	Maole_000747.T1, Maole_001986.T1, Maole_001987.T1, Ma
tripeptide transpor	biological	Maole_000747.T1, Maole_001986.T1, Maole_001987.T1, Ma
isoflavone 2'-hydro	molecular	Maole_018465.T1, Maole_019629.T1, Maole_019630.T1, Ma
response to water	biological	Maole_000411.T1, Maole_001432.T1, Maole_001467.T1, Ma
phosphatidylinosit	molecular	Maole_006773.T1, Maole_006774.T1, Maole_009992.T1, Ma
root meristem spec	biological	Maole_010505.T1, Maole_015631.T1, Maole_019167.T1, Ma
cation transmembr	biological	Maole_001469.T1, Maole_002761.T1, Maole_009880.T1, Ma
endoplasmic reticu	cellular_c	Maole_000059.T1, Maole_000498.T1, Maole_000672.T1, Ma
radial microtubula	biological	Maole_000026.T1, Maole_004136.T1, Maole_004750.T1, Ma
glutathione S-conj	molecular	Maole_007944.T1, Maole_013780.T1, Maole_020501.T1, Ma
peptidyl-tyrosine p	biological	Maole_001017.T1, Maole_004246.T1, Maole_006766.T1, Ma
ion transport	biological	Maole_001108.T1, Maole_002750.T1, Maole_003498.T1, Ma
1-aminocycloprop	molecular	Maole_004480.T1, Maole_008122.T1, Maole_017911.T1, Ma
regulation of floral	biological	Maole_002779.T1, Maole_002780.T1, Maole_011163.T1, Ma
carbohydrate hom	biological	Maole_005101.T1, Maole_006559.T1, Maole_008775.T1, Ma
xylan 1,4-beta-xyl	molecular	Maole_002406.T1, Maole_006066.T1, Maole_011085.T1, Ma
cadmium ion trans	biological	Maole_001020.T1, Maole_001021.T1, Maole_001022.T1, Ma
inward rectifier po	molecular	Maole_001610.T1, Maole_004862.T1, Maole_012375.T1, Ma
voltage-gated pota	molecular	Maole_001610.T1, Maole_004073.T1, Maole_004862.T1, Ma
malate dehydroger	molecular	Maole_000963.T1, Maole_001023.T1, Maole_020426.T1, Ma

phosphoprotein ph	molecular	Maole_000473.T1, Maole_002458.T1, Maole_002721.T1, Ma
indole glucosinola	biological	Maole_001020.T1, Maole_001021.T1, Maole_001022.T1, Ma
cellular response t	biological	Maole_001316.T1, Maole_006031.T1, Maole_014828.T1, Ma
organic hydroxy c	biological	Maole_001019.T1, Maole_001020.T1, Maole_001021.T1, Ma
phospholipid trans	biological	Maole_005514.T1, Maole_012276.T1, Maole_012537.T1, Ma
p-coumaryl alcoho	biological	Maole_001019.T1, Maole_001020.T1, Maole_001021.T1, Ma
plant-type primary	biological	Maole_000474.T1, Maole_000497.T1, Maole_005453.T1, Ma
racemase and epim	molecular	Maole_007035.T1, Maole_009796.T1, Maole_016082.T1, Ma
response to cyclop	biological	Maole_002461.T1, Maole_003496.T1, Maole_011642.T1, Ma
ion channel activit	molecular	Maole_001610.T1, Maole_004073.T1, Maole_010308.T1, Ma
ATP-dependent he	molecular	Maole_001380.T1, Maole_002505.T1, Maole_006176.T1, Ma
negative regulatio	biological	Maole_000806.T1, Maole_002707.T1, Maole_003886.T1, Ma
guanylate cyclase	molecular	Maole_002304.T1, Maole_006651.T1, Maole_006652.T1, Ma
cGMP biosyntheti	biological	Maole_002304.T1, Maole_006651.T1, Maole_006652.T1, Ma
stromule	cellular_c	Maole_000643.T1, Maole_000644.T1, Maole_001422.T1, Ma
phosphorylation	biological	Maole_001025.T1, Maole_001826.T1, Maole_004457.T1, Ma
L-phenylalanine c	biological	Maole_008291.T1, Maole_014706.T1, Maole_014707.T1, Ma
hydrolase activity,	molecular	Maole_000465.T1, Maole_001123.T1, Maole_001124.T1, Ma
negative regulatio	biological	Maole_001038.T1, Maole_005505.T1, Maole_008868.T1, Ma
UDP-glucose 6-de	molecular	Maole_003773.T1, Maole_012353.T1, Maole_012766.T1
UDP-glucuronate	biological	Maole_003773.T1, Maole_012353.T1, Maole_012766.T1
L-ascorbate oxida	molecular	Maole_000311.T1, Maole_003232.T1, Maole_010356.T1, Ma
phragmoplast	cellular_c	Maole_000026.T1, Maole_000432.T1, Maole_000636.T1, Ma
actin filament bind	molecular	Maole_003610.T1, Maole_005152.T1, Maole_005343.T1, Ma
procambium histo	biological	Maole_000379.T1, Maole_000381.T1, Maole_002288.T1, Ma
transpiration	biological	Maole_001037.T1, Maole_002191.T1, Maole_005818.T1, Ma
cation transport	biological	Maole_002761.T1, Maole_005816.T1, Maole_009880.T1, Ma
anther wall tapetu	biological	Maole_000473.T1, Maole_006040.T1, Maole_011930.T1, Ma
positive gravitropi	biological	Maole_000902.T1, Maole_002556.T1, Maole_005024.T1, Ma
polyamine catabol	biological	Maole_001538.T1, Maole_004167.T1, Maole_008424.T1, Ma
basal plasma mem	cellular_c	Maole_006437.T1, Maole_011128.T1, Maole_012819.T1, Ma
pattern recognitio	biological	Maole_001879.T1, Maole_003326.T1, Maole_009303.T1, Ma
maintenance of flo	biological	Maole_005064.T1, Maole_012785.T1, Maole_015343.T1, Ma
iron ion homeosta	biological	Maole_002750.T1, Maole_003498.T1, Maole_004444.T1, Ma
xylogalacturonan	biological	Maole_003148.T1, Maole_013116.T1, Maole_013119.T1, Ma
fatty acid biosynth	biological	Maole_001023.T1, Maole_001551.T1, Maole_001553.T1, Ma
protein phosphata	molecular	Maole_001091.T1, Maole_002389.T1, Maole_002874.T1, Ma
cellular response t	biological	Maole_011211.T1, Maole_013416.T1, Maole_013449.T1, Ma
metabolic process	biological	Maole_000026.T1, Maole_000285.T1, Maole_000416.T1, Ma
glucomannan 4-be	molecular	Maole_002689.T1, Maole_008616.T1, Maole_017313.T1, Ma
cellular response t	biological	Maole_009289.T1, Maole_010021.T1, Maole_010599.T1, Ma
low-affinity nitrat	molecular	Maole_013147.T1, Maole_013149.T1, Maole_017086.T1, Ma
low-affinity nitrat	biological	Maole_013147.T1, Maole_013149.T1, Maole_017086.T1, Ma
pollen tube	cellular_c	Maole_000211.T1, Maole_000738.T1, Maole_001533.T1, Ma
regulation of ligni	biological	Maole_001019.T1, Maole_001020.T1, Maole_001021.T1, Ma
glutamate recepto	molecular	Maole_001424.T1, Maole_005877.T1, Maole_005880.T1, Ma
cellular response t	biological	Maole_001424.T1, Maole_005877.T1, Maole_005880.T1, Ma
galactose transme	molecular	Maole_000211.T1, Maole_001533.T1, Maole_004942.T1, Ma

integral componen	cellular_c	Maole_000384.T1, Maole_003066.T1, Maole_006085.T1, Mao
tricarboxylic acid c	biological	Maole_003030.T1, Maole_004117.T1, Maole_005071.T1, Mao
transcription regul	molecular	Maole_002728.T1, Maole_003249.T1, Maole_005064.T1, Mao
butyrate metabolic	biological	Maole_005058.T1, Maole_005059.T1, Maole_009587.T1, Mao
tau-protein kinase	molecular	Maole_001137.T1, Maole_002747.T1, Maole_004878.T1, Mao
ATP biosynthetic p	biological	Maole_001467.T1, Maole_001469.T1, Maole_004003.T1, Mao
regulation of grow	biological	Maole_007894.T1, Maole_019219.T1, Maole_022820.T1
positive regulation	biological	Maole_007894.T1, Maole_019219.T1, Maole_022820.T1
sulfate transmemb	molecular	Maole_004916.T1, Maole_004918.T1, Maole_013416.T1, Mao
cyclin-dependent p	molecular	Maole_001703.T1, Maole_001859.T1, Maole_003901.T1, Mao
3-oxoacyl-[acyl-ca	molecular	Maole_003221.T1, Maole_006203.T1, Maole_007633.T1, Mao
defense response	biological	Maole_000465.T1, Maole_000473.T1, Maole_000636.T1, Mao
lipid transport	biological	Maole_001229.T1, Maole_001277.T1, Maole_003204.T1, Mao
glucosinolate:prot	molecular	Maole_013149.T1, Maole_017085.T1, Maole_017086.T1, Mao
phloem glucosinol	biological	Maole_013149.T1, Maole_017085.T1, Maole_017086.T1, Mao
glucosinolate trans	biological	Maole_013149.T1, Maole_017085.T1, Maole_017086.T1, Mao
indole-3-butyrate b	molecular	Maole_003601.T1, Maole_003602.T1, Maole_003604.T1, Mao
xyloglucan biosyn	biological	Maole_000384.T1, Maole_005244.T1, Maole_007183.T1, Mao
secondary metabol	biological	Maole_005983.T1, Maole_007894.T1, Maole_012497.T1, Mao
anthocyanidin 3-O	molecular	Maole_001919.T1, Maole_001920.T1, Maole_006469.T1, Mao
response to nitrate	biological	Maole_001538.T1, Maole_001971.T1, Maole_002365.T1, Mao
potassium ion imp	biological	Maole_001610.T1, Maole_001854.T1, Maole_004862.T1, Mao
malate metabolic p	biological	Maole_000963.T1, Maole_001023.T1, Maole_004117.T1, Mao
potassium ion tran	biological	Maole_001432.T1, Maole_001610.T1, Maole_004073.T1, Mao
high-affinity secon	molecular	Maole_000400.T1, Maole_002056.T1, Maole_002707.T1
phosphatidylinosit	molecular	Maole_000738.T1, Maole_001488.T1, Maole_001649.T1, Mao
secondary growth	biological	Maole_005101.T1, Maole_006559.T1, Maole_018459.T1, Mao
developmental pro	biological	Maole_005343.T1, Maole_009522.T1, Maole_012022.T1, Mao
induced systemic r	biological	Maole_001424.T1, Maole_004554.T1, Maole_007320.T1, Mao
mannosylation	biological	Maole_002136.T1, Maole_002689.T1, Maole_006085.T1, Mao
regulation of meris	biological	Maole_002779.T1, Maole_002780.T1, Maole_003283.T1, Mao
phospholipid bindi	molecular	Maole_004627.T1, Maole_005307.T1, Maole_005630.T1, Mao

**Term**	**Class**	**Gene ID**
Plant-pathogen int	Organism	Maole_000905.T1, Maole_001631.T1, Maole_001879.T1, Mao
Amino sugar and n	Metabolis	Maole_000672.T1, Maole_000921.T1, Maole_000922.T1, Mao
Ether lipid metabo	Metabolis	Maole_001011.T1, Maole_001100.T1, Maole_001516.T1, Mao
Plant hormone sig	Environm	Maole_000473.T1, Maole_000724.T1, Maole_001025.T1, Mao
Galactose metabol	Metabolis	Maole_001037.T1, Maole_001123.T1, Maole_001124.T1, Mao
Linoleic acid meta	Metabolis	Maole_007386.T1, Maole_007927.T1, Maole_010760.T1, Mao
Inositol phosphate	Metabolis	Maole_000738.T1, Maole_001060.T1, Maole_001488.T1, Mao
Starch and sucrose	Metabolis	Maole_000612.T1, Maole_000619.T1, Maole_000922.T1, Mao
Fatty acid elongati	Metabolis	Maole_002411.T1, Maole_003085.T1, Maole_004215.T1, Mao
ABC transporters	Environm	Maole_001545.T1, Maole_004980.T1, Maole_004982.T1, Mao
Phagosome	Cellular P	Maole_001488.T1, Maole_002627.T1, Maole_002924.T1, Mao

Term	Class	Gene ID
response to xenobi	biological	Maole_005120.T1, Maole_005178.T1, Maole_005179.T1, Mao
response to chitin	biological	Maole_000378.T1, Maole_002027.T1, Maole_002707.T1, Mao
drug transmembra	biological	Maole_001019.T1, Maole_001020.T1, Maole_001021.T1, Mao
integral componen	cellular_c	Maole_000212.T1, Maole_000390.T1, Maole_001019.T1, Mao
transcription facto	molecular	Maole_000378.T1, Maole_000437.T1, Maole_000677.T1, Mao
lignin catabolic pr	biological	Maole_002588.T1, Maole_002636.T1, Maole_002645.T1, Mao
auxin-activated sig	biological	Maole_000378.T1, Maole_000437.T1, Maole_000677.T1, Mao
ATPase activity, c	molecular	Maole_000390.T1, Maole_001096.T1, Maole_001545.T1, Mao
hydroquinone:oxy	molecular	Maole_002588.T1, Maole_002636.T1, Maole_002645.T1, Mao
detection of bacter	biological	Maole_004972.T1, Maole_006779.T1, Maole_009317.T1, Mao
embryonic pattern	biological	Maole_000378.T1, Maole_000437.T1, Maole_000677.T1, Mao
regulation of trans	biological	Maole_000378.T1, Maole_000437.T1, Maole_000677.T1, Mao
xenobiotic-transpo	molecular	Maole_000390.T1, Maole_001096.T1, Maole_001545.T1, Mao
regulation of anion	biological	Maole_004972.T1, Maole_006779.T1, Maole_009317.T1, Mao
plant-type vacuole	cellular_c	Maole_000390.T1, Maole_001096.T1, Maole_001931.T1, Mao
response to auxin	biological	Maole_000378.T1, Maole_000437.T1, Maole_000677.T1, Mao
transcription, DNA	biological	Maole_000378.T1, Maole_000437.T1, Maole_000677.T1, Mao
plasmodesma	cellular_c	Maole_001375.T1, Maole_001461.T1, Maole_001545.T1, Mao
xylem and phloem	biological	Maole_000378.T1, Maole_000437.T1, Maole_000677.T1, Mao
hormone-mediated	biological	Maole_001375.T1, Maole_001461.T1, Maole_004639.T1, Mao
ATP binding	molecular	Maole_000390.T1, Maole_001019.T1, Maole_001020.T1, Mao
peptide receptor ac	molecular	Maole_001375.T1, Maole_001461.T1, Maole_004639.T1, Mao
plasma membrane	cellular_c	Maole_001019.T1, Maole_001020.T1, Maole_001021.T1, Mao
root development	biological	Maole_000378.T1, Maole_000437.T1, Maole_000677.T1, Mao
transmembrane rec	molecular	Maole_001375.T1, Maole_001461.T1, Maole_004639.T1, Mao
transmembrane rec	biological	Maole_001375.T1, Maole_001461.T1, Maole_004639.T1, Mao
cell wall macromo	biological	Maole_020579.T1, Maole_023238.T1, Maole_023239.T1, Mao
apoplast	cellular_c	Maole_002588.T1, Maole_002636.T1, Maole_002645.T1, Mao
protein autophosp	biological	Maole_001375.T1, Maole_001461.T1, Maole_004639.T1, Mao
hydrogen-transloc	molecular	Maole_003069.T1, Maole_003305.T1, Maole_003945.T1, Mao
plant-type hyperse	biological	Maole_002245.T1, Maole_002247.T1, Maole_002253.T1, Mao
establishment or m	biological	Maole_003069.T1, Maole_003305.T1, Maole_003945.T1, Mao
protein serine/thre	molecular	Maole_001375.T1, Maole_001461.T1, Maole_004639.T1, Mao
auxin polar transp	biological	Maole_003069.T1, Maole_003305.T1, Maole_003945.T1, Mao
endosome membra	cellular_c	Maole_003069.T1, Maole_003305.T1, Maole_003945.T1, Mao
ubiquitin protein li	molecular	Maole_001375.T1, Maole_001461.T1, Maole_004639.T1, Mao
receptor-mediated	biological	Maole_004972.T1, Maole_009317.T1, Maole_009320.T1, Mao
positive regulation	biological	Maole_005120.T1, Maole_005178.T1, Maole_005179.T1, Mao
inorganic diphosp	molecular	Maole_003069.T1, Maole_003305.T1, Maole_003945.T1, Mao
defense response b	biological	Maole_001020.T1, Maole_001021.T1, Maole_001022.T1, Mao
acropetal auxin tra	biological	Maole_004980.T1, Maole_004982.T1, Maole_011110.T1, Mao
pH reduction	biological	Maole_001461.T1, Maole_004639.T1, Maole_015166.T1, Mao
immune response-	biological	Maole_006779.T1, Maole_015116.T1, Maole_021416.T1, Mao
chitinase activity	molecular	Maole_020579.T1, Maole_023238.T1, Maole_023239.T1, Mao
chitin catabolic pr	biological	Maole_020579.T1, Maole_023238.T1, Maole_023239.T1, Mao
chitin binding	molecular	Maole_020579.T1, Maole_023238.T1, Maole_023239.T1, Mao
glutamate receptor	molecular	Maole_001424.T1, Maole_005877.T1, Maole_005880.T1, Mao

motile cilium	cellular_c	Maole_004972.T1, Maole_014265.T1, Maole_014267.T1, Ma
xyloglucan-specifi	molecular	Maole_002978.T1, Maole_003782.T1, Maole_003783.T1, Ma
cellular response t	biological	Maole_001424.T1, Maole_005877.T1, Maole_005880.T1, Ma
negative regulation	biological	Maole_000378.T1, Maole_000437.T1, Maole_000677.T1, Ma
transmembrane sig	molecular	Maole_001461.T1, Maole_004639.T1, Maole_015166.T1, Ma
vacuole	cellular_c	Maole_000390.T1, Maole_001096.T1, Maole_001931.T1, Ma
protein kinase acti	molecular	Maole_001461.T1, Maole_004639.T1, Maole_004972.T1, Ma
auxin efflux transn	molecular	Maole_004980.T1, Maole_004982.T1, Maole_011110.T1, Ma
polysaccharide cat	biological	Maole_020579.T1, Maole_023238.T1, Maole_023239.T1, Ma
copper ion binding	molecular	Maole_002588.T1, Maole_002636.T1, Maole_002645.T1, Ma
chlorophyll catabo	molecular	Maole_007944.T1, Maole_013780.T1, Maole_020501.T1, Ma
cellular response t	biological	Maole_001020.T1, Maole_001021.T1, Maole_001022.T1, Ma
ionotropic glutama	molecular	Maole_001424.T1, Maole_005877.T1, Maole_005880.T1, Ma
photosystem I	cellular_c	Maole_000212.T1, Maole_015203.T1, Maole_021341.T1, Ma
auxin efflux	biological	Maole_004980.T1, Maole_004982.T1, Maole_011110.T1, Ma
calcium channel ac	molecular	Maole_001424.T1, Maole_005877.T1, Maole_005880.T1, Ma
defense response s	biological	Maole_006779.T1, Maole_015116.T1, Maole_021416.T1, Ma
glutathione S-conj	molecular	Maole_007944.T1, Maole_013780.T1, Maole_020501.T1, Ma
lead ion transport	biological	Maole_012520.T1, Maole_012595.T1, Maole_012596.T1, Ma
terpenoid transpor	biological	Maole_012520.T1, Maole_012595.T1, Maole_012596.T1, Ma
cellular glucan me	biological	Maole_002978.T1, Maole_003782.T1, Maole_003783.T1, Ma
xyloglucan:xylogl	molecular	Maole_002978.T1, Maole_003782.T1, Maole_003783.T1, Ma
polar nucleus	cellular_c	Maole_011635.T1, Maole_013907.T1, Maole_013908.T1, Ma
defense response t	biological	Maole_001020.T1, Maole_001021.T1, Maole_001022.T1, Ma
regulation of salic	biological	Maole_002999.T1, Maole_003050.T1, Maole_012089.T1, Ma
basipetal auxin tra	biological	Maole_004980.T1, Maole_004982.T1, Maole_011110.T1, Ma
guanylate cyclase	molecular	Maole_001461.T1, Maole_004639.T1, Maole_006651.T1, Ma
cGMP biosyntheti	biological	Maole_001461.T1, Maole_004639.T1, Maole_006651.T1, Ma
calcium ion transn	biological	Maole_001424.T1, Maole_005877.T1, Maole_005880.T1, Ma
transmembrane rec	molecular	Maole_006779.T1, Maole_009317.T1, Maole_015116.T1, Ma
receptor serine/thr	molecular	Maole_009320.T1, Maole_010336.T1, Maole_015116.T1, Ma
organic hydroxy c	biological	Maole_001019.T1, Maole_001020.T1, Maole_001021.T1, Ma
p-coumaryl alcoho	biological	Maole_001019.T1, Maole_001020.T1, Maole_001021.T1, Ma
chlorophyll bindin	molecular	Maole_000212.T1, Maole_015203.T1, Maole_021341.T1, Ma
lignin biosynthetic	biological	Maole_002588.T1, Maole_003245.T1, Maole_010114.T1, Ma
response to mecha	biological	Maole_002978.T1, Maole_003786.T1, Maole_003790.T1, Ma
cellular response t	biological	Maole_001424.T1, Maole_002245.T1, Maole_002247.T1, Ma
proton transport	biological	Maole_003069.T1, Maole_003305.T1, Maole_003945.T1, Ma
dipeptide transport	molecular	Maole_001986.T1, Maole_001987.T1, Maole_002591.T1, Ma
tripeptide transpor	molecular	Maole_001986.T1, Maole_001987.T1, Maole_002591.T1, Ma
dipeptide transport	biological	Maole_001986.T1, Maole_001987.T1, Maole_002591.T1, Ma
tripeptide transpor	biological	Maole_001986.T1, Maole_001987.T1, Maole_002591.T1, Ma
abscisic acid trans	biological	Maole_012520.T1, Maole_012595.T1, Maole_012596.T1, Ma
plant-type vacuole	cellular_c	Maole_002591.T1, Maole_003069.T1, Maole_003305.T1, Ma
potassium ion bind	molecular	Maole_003069.T1, Maole_003305.T1, Maole_003945.T1, Ma
response to copper	biological	Maole_002636.T1, Maole_002648.T1, Maole_004626.T1, Ma
vacuolar membran	cellular_c	Maole_000390.T1, Maole_001020.T1, Maole_001021.T1, Ma
pollen maturation	biological	Maole_009320.T1, Maole_010336.T1, Maole_015116.T1, Ma

defense response t	biological	Maole_001424.T1, Maole_002245.T1, Maole_002247.T1, Mao
calcium-mediated	biological	Maole_001424.T1, Maole_005877.T1, Maole_005880.T1, Mao
protein-chromopho	biological	Maole_000212.T1, Maole_015203.T1, Maole_021341.T1, Mao
phenylpropanoid m	biological	Maole_001538.T1, Maole_009465.T1, Maole_010356.T1, Mao
magnesium ion bir	molecular	Maole_000212.T1, Maole_003069.T1, Maole_003305.T1, Mao
regulation of lignii	biological	Maole_001019.T1, Maole_001020.T1, Maole_001021.T1, Mao
transport	biological	Maole_001019.T1, Maole_001020.T1, Maole_001021.T1, Mao
microsporogenesis	biological	Maole_009317.T1, Maole_009320.T1, Maole_010336.T1, Mao
4 iron, 4 sulfur clu	molecular	Maole_000212.T1, Maole_015203.T1, Maole_021341.T1, Mao
induced systemic r	biological	Maole_001424.T1, Maole_007827.T1, Maole_012162.T1, Mao
oxidoreductase act	molecular	Maole_000212.T1, Maole_002588.T1, Maole_002645.T1, Mao
cell wall	cellular_c	Maole_002245.T1, Maole_002247.T1, Maole_002248.T1, Mao
sulfonylurea recep	molecular	Maole_000390.T1, Maole_007944.T1, Maole_013780.T1
oxidoreductase act	molecular	Maole_006214.T1, Maole_017644.T1, Maole_019939.T1
indole glucosinola	biological	Maole_001020.T1, Maole_001021.T1, Maole_001022.T1
membrane	cellular_c	Maole_000182.T1, Maole_001019.T1, Maole_001020.T1, Mao
transmembrane tra	biological	Maole_000390.T1, Maole_004980.T1, Maole_004982.T1, Mao
auxin influx transn	molecular	Maole_004982.T1, Maole_013561.T1, Maole_014744.T1, Mao
auxin influx	biological	Maole_004982.T1, Maole_013561.T1, Maole_014744.T1, Mao
calcium ion transp	biological	Maole_001424.T1, Maole_005877.T1, Maole_005880.T1, Mao
peptide transporter	molecular	Maole_002591.T1, Maole_006581.T1, Maole_006583.T1
high-affinity oligo	molecular	Maole_002591.T1, Maole_006581.T1, Maole_006583.T1
ATPase activity	molecular	Maole_001019.T1, Maole_001020.T1, Maole_001021.T1, Mao
regulation of hydro	biological	Maole_002707.T1, Maole_002999.T1, Maole_003050.T1, Mao
meristem developr	biological	Maole_023259.T1, Maole_023260.T1, Maole_023776.T1, Mao
endomembrane sys	cellular_c	Maole_006779.T1, Maole_015116.T1, Maole_021416.T1, Mao
intramolecular trar	molecular	Maole_002953.T1, Maole_016874.T1, Maole_016875.T1, Mao
sodium ion transpo	biological	Maole_003069.T1, Maole_003305.T1, Maole_003945.T1, Mao
plant-type cell wal	biological	Maole_002978.T1, Maole_003782.T1, Maole_003783.T1, Mao
NAD(P)H oxidase	molecular	Maole_006214.T1, Maole_017644.T1, Maole_019939.T1
11-oxo-beta-amyri	biological	Maole_010943.T1, Maole_010944.T1, Maole_010945.T1
glycyrrhetinate bic	biological	Maole_010943.T1, Maole_010944.T1, Maole_010945.T1
transmembrane rec	molecular	Maole_009320.T1, Maole_010336.T1, Maole_022500.T1, Mao
cold acclimation	biological	Maole_002245.T1, Maole_002247.T1, Maole_002253.T1, Mao
systemic acquired	biological	Maole_001020.T1, Maole_001021.T1, Maole_001022.T1, Mao
floral organ abscis	biological	Maole_009320.T1, Maole_010336.T1, Maole_022500.T1, Mao
response to water	biological	Maole_001931.T1, Maole_002588.T1, Maole_003069.T1, Mao
pentacyclic triterpe	biological	Maole_002953.T1, Maole_016874.T1, Maole_016875.T1, Mao
DNA metabolic pr	biological	Maole_006224.T1, Maole_006226.T1, Maole_006227.T1, Mao
plant-type cell wal	biological	Maole_002978.T1, Maole_003786.T1, Maole_003790.T1, Mao
induced systemic r	biological	Maole_002245.T1, Maole_002247.T1, Maole_002253.T1, Mao
organic phosphona	molecular	Maole_001020.T1, Maole_012596.T1, Maole_012598.T1
photosynthesis	biological	Maole_000212.T1, Maole_015203.T1, Maole_021341.T1, Mao
leaf development	biological	Maole_003069.T1, Maole_003305.T1, Maole_003945.T1, Mao
gravitropism	biological	Maole_001424.T1, Maole_004982.T1, Maole_007827.T1, Mao
response to salt str	biological	Maole_000390.T1, Maole_002245.T1, Maole_002247.T1, Mao
ligand-gated ion cl	molecular	Maole_001424.T1, Maole_014199.T1
lupeol synthase ac	molecular	Maole_016874.T1, Maole_016875.T1

cellular response t	biological	Maole_001424.T1, Maole_014199.T1
adenylate cyclase a	molecular	Maole_021367.T1, Maole_021368.T1
cAMP biosyntheti	biological	Maole_021367.T1, Maole_021368.T1
(+)-abscisic acid D	molecular	Maole_011357.T1, Maole_011358.T1
(+)-abscisic acid D	biological	Maole_011357.T1, Maole_011358.T1
defense response	biological	Maole_004881.T1, Maole_005996.T1, Maole_006651.T1, Ma
cadmium ion trans	molecular	Maole_001020.T1, Maole_001021.T1, Maole_001022.T1
cadmium ion trans	biological	Maole_001020.T1, Maole_001021.T1, Maole_001022.T1
G-protein coupled	molecular	Maole_001424.T1, Maole_016541.T1, Maole_016542.T1
transporter activity	molecular	Maole_000182.T1, Maole_001538.T1, Maole_001986.T1, Ma
protein catabolic p	biological	Maole_002999.T1, Maole_003050.T1, Maole_012089.T1, Ma
electron carrier act	molecular	Maole_000212.T1, Maole_015203.T1, Maole_021341.T1, Ma
response to cold	biological	Maole_002245.T1, Maole_002247.T1, Maole_002248.T1, Ma
triterpenoid biosyn	biological	Maole_002953.T1, Maole_016874.T1
response to leucine	biological	Maole_001986.T1, Maole_001987.T1
response to histidi	biological	Maole_001986.T1, Maole_001987.T1
response to phenyl	biological	Maole_001986.T1, Maole_001987.T1
xenobiotic transpo	biological	Maole_004982.T1, Maole_013561.T1
tetrahydrofolylpoly	biological	Maole_016478.T1, Maole_018141.T1
phytochelatin trans	molecular	Maole_011357.T1, Maole_011358.T1
phytochelatin trans	biological	Maole_011357.T1, Maole_011358.T1
beta-amyrin syntha	molecular	Maole_002953.T1, Maole_016874.T1
innate immune res	biological	Maole_001424.T1, Maole_001461.T1, Maole_004639.T1, Ma

**Term**	**Class**	**Gene ID**
Plant hormone sign	Environm	Maole_000378.T1, Maole_000437.T1, Maole_000677.T1, Ma
ABC transporters	Environm	Maole_001545.T1, Maole_004980.T1, Maole_004982.T1, Ma
Plant-pathogen int	Organism	Maole_004972.T1, Maole_005996.T1, Maole_006214.T1, Ma
Oxidative phospho	Metabolis	Maole_003069.T1, Maole_003305.T1, Maole_003945.T1, Ma

**Size (kb)**	**Core don**	**Gene clus**	**Gene cluster genes**
389.28	AMP-binc	alkaloid	Maole_023229;Maole_023230;Maole_023231;Maol
643.66	Cu_amine	alkaloid	Maole_016080;Maole_016081;Maole_016082;Maol
395.15	Chal_sti_s	lignan-pol	Maole_017148;Maole_017149;Maole_017150;Maol
1282.35	Chal_sti_s	polyketide	Maole_008020;Maole_008021;Maole_008022;Maol
655.51	HMGL-lil	putative	Maole_006918;Maole_006919;Maole_006920;Maol
258.04	Transfera	putative	Maole_008905;Maole_008906;Maole_008907;Maol
924.13	2OG-FeII_	putative	Maole_012577;Maole_012578;Maole_012579;Maol
646.22	Methyltra	putative	Maole_017973;Maole_017974;Maole_017975;Maol
309.6	Methyltra	putative	Maole_017520;Maole_017521;Maole_017522;Maol
506.45	Peptidase_	saccharide	Maole_015577;Maole_015578;Maole_015579;Maol
1125.16	Amino_ox	saccharide	Maole_019301;Maole_019302;Maole_019303;Maol
1217.9	Acetyltran	saccharide	Maole_017274;Maole_017275;Maole_017276;Maol
930.1	UDPGT_2	saccharide	Maole_001301;Maole_001302;Maole_001303;Maol
484.08	Glycos_tr	saccharide	Maole_007253;Maole_007254;Maole_007255;Maol
553.1	Glycos_tr	saccharide	Maole_020684;Maole_020685;Maole_020686;Maol

215.08	Epimerase	saccharide	Maole_021736;Maole_021737;Maole_021738;Maol
756	Acetyltrar	saccharide	Maole_000812;Maole_000813;Maole_000814;Maol
620.5	Epimerase	saccharide	Maole_000957;Maole_000958;Maole_000959;Maol
649.6	Chal_sti_s	saccharide	Maole_002119;Maole_002120;Maole_002121;Maol
439.73	Terpene_s	terpene	Maole_002169;Maole_002170;Maole_002171;Maol
624.05	Epimerase	terpene	Maole_014441;Maole_014442;Maole_014443;Maol
325.95	Acetyltrar	terpene	Maole_017612;Maole_017613;Maole_017614;Maol
442.84	SQHop_cy	terpene	Maole_016872;Maole_016873;Maole_016874;Maol

Collector	Dates	Latitude-	Raw Rea	Raw Bas	Raw Q20	Raw Q30	Clean Re	Clean Ba
Yongpeng	2017.09.1	N23.90, E	5.778	51.15	NA	NA	NA	NA
Yongpeng	2017.09.1	N23.90, E	899.778	134.967	120.195(8	107.959(8	NA	NA
Sihai War	2016.07.0	N23.9, E1	40.972	6.146	6.059(98.(	5.914(96.2	40.181(98	5.930(96.5
Sihai War	2016.07.2	N23.9, E1	48.203	7.23	7.112(98.4	6.903(95.5	47.097(97	6.867(95.(
Sihai War	2016.08.1	N23.9, E1	43.559	6.534	6.451(98.7	6.321(96.7	42.690(98	6.245(95.6
Sihai War	2016.08.2	N23.9, E1	42.383	6.357	6.254(98.4	6.071(95.5	41.475(97	6.063(95.4
Sihai War	2016.08.2	N23.9, E1	47.835	7.175	7.054(98.3	6.842(95.3	46.688(97	6.850(95.5
Dong-Xu	2013.10.0	N24.78, E	46.365	4.59	4.553(99.2	4.356(94.9	44.637(96	4.386(95.6
Dong-Xu	2014.09.0	N24.78, E	44.041	4.404	4.331(98.3	4.115(93.4	41.339(93	4.097(93.(

**Filtered (**	**LTR-RT (*S* /*I* >=3)**
1.79	11.39%
6.60	39.34%
2.91	18.30%
12.82	46.58%
5.06	33.61%
7.34	69.15%
10.29	72.32%
5.57	40.36%
2.61	16.77%

2013; doi:10.1038/nature12817.
que history. BioRxiv. 2018; doi:10.1101/264101.
gb-2013-14-5-r41.
Nature. 2007;449:463. doi:10.1038/nature06148.
)6-604. doi:10.1126/science.1128691.
'0.
2017;49:765. doi:10.1038/ng.3839.
enes regulating pod color. Genome Biology. 2013;14 6:r53. doi:10.1186/gb-2013-14-6-r53.

ırnal. 2017;89 4:789-804. doi:10.1111/tpj.13415.

‹ phytologist. 2015;206 4:1283-96. doi:10.1111/nph.13150.

ence. 2014;345 6201:1181-4. doi:10.1126/science.1255274.
7;546:148. doi:10.1038/nature22380.
07;35 Database issue:D883-7. doi:10.1093/nar/gkl976.

ole_000953.T1, Maole_000954.T1, Maole_002491.T1, Maole_003004.T1, Maole_003005.T1,
ole_005180.T1, Maole_005694.T1, Maole_005695.T1, Maole_005698.T1, Maole_005700.T1,
ole_003481.T1, Maole_003483.T1, Maole_003485.T1, Maole_003488.T1, Maole_003786.T1,
ole_000953.T1, Maole_000954.T1, Maole_002491.T1, Maole_003004.T1, Maole_003005.T1,
ole_002100.T1, Maole_003493.T1, Maole_005145.T1, Maole_005341.T1, Maole_005836.T1,
ole_005210.T1, Maole_006239.T1, Maole_006824.T1, Maole_007334.T1, Maole_007560.T1,
ole_000953.T1, Maole_000954.T1, Maole_002491.T1, Maole_003004.T1, Maole_003005.T1,
ole_001434.T1, Maole_002027.T1, Maole_003161.T1, Maole_003875.T1, Maole_004490.T1,
ole_005631.T1, Maole_005991.T1, Maole_007389.T1, Maole_008518.T1, Maole_010002.T1,
ole_006239.T1, Maole_006379.T1, Maole_006380.T1, Maole_006824.T1, Maole_009283.T1,
ole_000953.T1, Maole_000954.T1, Maole_002491.T1, Maole_003004.T1, Maole_003005.T1,
ole_000838.T1, Maole_000947.T1, Maole_000949.T1, Maole_000952.T1, Maole_000953.T1,
ole_011302.T1, Maole_012742.T1, Maole_014799.T1, Maole_016769.T1, Maole_017626.T1,
ole_014799.T1, Maole_017628.T1, Maole_018434.T1, Maole_020950.T1, Maole_020951.T1,
ole_001461.T1, Maole_002229.T1, Maole_002311.T1, Maole_003124.T1, Maole_003404.T1,
ole_005120.T1, Maole_005178.T1, Maole_005179.T1, Maole_005180.T1, Maole_005694.T1,
ole_000953.T1, Maole_000954.T1, Maole_002491.T1, Maole_003004.T1, Maole_003005.T1,
ole_005145.T1, Maole_005836.T1, Maole_005991.T1, Maole_006239.T1, Maole_006824.T1,
ole_012575.T1, Maole_014799.T1, Maole_016769.T1, Maole_017626.T1, Maole_017628.T1,
ole_006513.T1, Maole_007389.T1, Maole_008116.T1, Maole_008187.T1, Maole_008909.T1,

ole_014799.T1, Maole_017628.T1, Maole_018434.T1, Maole_020950.T1, Maole_020951.T1,
ole_007592.T1, Maole_007594.T1, Maole_007595.T1, Maole_007597.T1, Maole_008212.T1,
ole_002248.T1, Maole_002249.T1, Maole_002250.T1, Maole_002253.T1, Maole_002254.T1,
ole_002181.T1, Maole_002183.T1, Maole_005259.T1, Maole_007733.T1, Maole_015424.T1,
ole_012034.T1, Maole_012036.T1, Maole_012037.T1, Maole_017285.T1, Maole_021133.T1,
ole_000779.T1, Maole_000833.T1, Maole_001049.T1, Maole_001050.T1, Maole_003749.T1,
ole_023241.T1, Maole_023344.T1, Maole_023347.T1, Maole_023983.T1, Maole_023998.T1,
ole_007597.T1, Maole_009013.T1, Maole_013458.T1, Maole_013459.T1, Maole_014253.T1,
ole_023063.T1, Maole_023064.T1, Maole_023690.T1, Maole_024007.T1
ole_023063.T1, Maole_023064.T1, Maole_023690.T1, Maole_024007.T1
ole_005886.T1, Maole_005890.T1, Maole_005893.T1, Maole_005894.T1, Maole_005895.T1,
ole_002335.T1, Maole_002340.T1, Maole_002341.T1, Maole_002343.T1, Maole_008096.T1,
ole_006532.T1, Maole_008212.T1, Maole_010105.T1, Maole_019117.T1, Maole_021175.T1,
ole_000779.T1, Maole_000833.T1, Maole_001049.T1, Maole_001050.T1, Maole_003751.T1,
ole_002038.T1, Maole_002039.T1, Maole_002040.T1, Maole_003774.T1, Maole_004053.T1,
ole_000677.T1, Maole_001428.T1, Maole_001434.T1, Maole_001560.T1, Maole_001767.T1,
ole_004679.T1, Maole_005107.T1, Maole_005210.T1, Maole_005631.T1, Maole_006239.T1,
ole_015129.T1, Maole_017478.T1, Maole_017931.T1, Maole_018177.T1, Maole_019080.T1,
ole_000677.T1, Maole_001428.T1, Maole_001429.T1, Maole_001434.T1, Maole_001560.T1,
ole_000953.T1, Maole_000954.T1, Maole_002180.T1, Maole_002183.T1, Maole_003004.T1,
ole_002181.T1, Maole_002183.T1, Maole_002318.T1, Maole_002319.T1, Maole_002320.T1,
ole_001434.T1, Maole_002027.T1, Maole_003161.T1, Maole_003875.T1, Maole_004490.T1,
ole_005894.T1, Maole_005895.T1, Maole_007887.T1
ole_019959.T1, Maole_019963.T1, Maole_020579.T1, Maole_023090.T1, Maole_023091.T1,
ole_001434.T1, Maole_002027.T1, Maole_003161.T1, Maole_003875.T1, Maole_004490.T1,
ole_006532.T1, Maole_008212.T1, Maole_010105.T1, Maole_019117.T1, Maole_021175.T1,
ole_000953.T1, Maole_000954.T1, Maole_003004.T1, Maole_003005.T1, Maole_003774.T1,
ole_001434.T1, Maole_001560.T1, Maole_002027.T1, Maole_002249.T1, Maole_002250.T1,
ole_022500.T1, Maole_022504.T1, Maole_022507.T1, Maole_022510.T1, Maole_022511.T1,
ole_002181.T1, Maole_002183.T1, Maole_002318.T1, Maole_002319.T1, Maole_002320.T1,
ole_014799.T1, Maole_016769.T1, Maole_017626.T1, Maole_018434.T1, Maole_020950.T1,
ole_015129.T1, Maole_017478.T1, Maole_017931.T1, Maole_018177.T1, Maole_019080.T1,
ole_002341.T1, Maole_002342.T1, Maole_002343.T1, Maole_004561.T1, Maole_004613.T1,
ole_000529.T1, Maole_000531.T1, Maole_000838.T1, Maole_000911.T1, Maole_001047.T1,
ole_014253.T1, Maole_016516.T1, Maole_019829.T1, Maole_019870.T1, Maole_020460.T1,
ole_003786.T1, Maole_003790.T1, Maole_003791.T1, Maole_003793.T1, Maole_019618.T1
ole_006532.T1, Maole_008212.T1, Maole_010105.T1, Maole_019117.T1, Maole_021175.T1,
ole_001434.T1, Maole_001560.T1, Maole_002027.T1, Maole_003001.T1, Maole_003161.T1,
ole_006226.T1, Maole_006227.T1, Maole_006262.T1, Maole_019907.T1, Maole_019908.T1
ole_005259.T1, Maole_007733.T1, Maole_015424.T1, Maole_015426.T1, Maole_015427.T1,
ole_017628.T1, Maole_018434.T1, Maole_020950.T1, Maole_020951.T1, Maole_020952.T1,
ole_005259.T1, Maole_007733.T1, Maole_015424.T1, Maole_015426.T1, Maole_015427.T1,
ole_016084.T1, Maole_016085.T1, Maole_021078.T1, Maole_021081.T1
ole_016084.T1, Maole_016085.T1, Maole_021078.T1, Maole_021081.T1
ole_013807.T1, Maole_013808.T1
ole_022500.T1, Maole_022504.T1, Maole_022507.T1, Maole_022510.T1, Maole_022511.T1,
ole_023241.T1, Maole_023344.T1, Maole_023347.T1, Maole_023983.T1, Maole_023998.T1,
ole_006828.T1, Maole_015129.T1, Maole_017478.T1, Maole_017931.T1, Maole_018177.T1,

ole_023241.T1, Maole_023344.T1, Maole_023347.T1, Maole_023983.T1, Maole_023998.T1,
ole_023241.T1, Maole_023344.T1, Maole_023347.T1, Maole_023983.T1, Maole_023998.T1,
ole_000911.T1, Maole_000947.T1, Maole_000949.T1, Maole_000952.T1, Maole_000953.T1,
ole_016084.T1, Maole_016085.T1, Maole_021078.T1, Maole_021081.T1
ole_016084.T1, Maole_016085.T1, Maole_021078.T1, Maole_021081.T1
ole_016084.T1, Maole_016085.T1, Maole_021078.T1, Maole_021081.T1
ole_016084.T1, Maole_016085.T1, Maole_021078.T1, Maole_021081.T1
ole_016084.T1, Maole_016085.T1, Maole_021078.T1, Maole_021081.T1
ole_007597.T1, Maole_009013.T1, Maole_013458.T1, Maole_013459.T1, Maole_014253.T1,
ole_005991.T1, Maole_010025.T1, Maole_022920.T1
ole_005890.T1, Maole_005893.T1, Maole_005895.T1, Maole_007887.T1, Maole_007888.T1
ole_005890.T1, Maole_005893.T1, Maole_005895.T1, Maole_007887.T1, Maole_007888.T1
ole_000677.T1, Maole_001428.T1, Maole_001434.T1, Maole_001560.T1, Maole_002027.T1,
ole_003488.T1, Maole_005499.T1, Maole_007741.T1, Maole_009743.T1, Maole_009762.T1,
ole_019305.T1
ole_016084.T1, Maole_016085.T1, Maole_021078.T1, Maole_021081.T1
ole_007733.T1, Maole_015426.T1, Maole_015427.T1, Maole_017521.T1, Maole_019116.T1
ole_002183.T1, Maole_002319.T1, Maole_002322.T1, Maole_002329.T1, Maole_002335.T1,
ole_008673.T1, Maole_008917.T1, Maole_017045.T1, Maole_017791.T1, Maole_018969.T1,
ole_020266.T1, Maole_020268.T1, Maole_020576.T1, Maole_022877.T1, Maole_023342.T1
ole_023063.T1, Maole_023064.T1, Maole_023690.T1, Maole_024007.T1
ole_020460.T1, Maole_021816.T1, Maole_022916.T1, Maole_022917.T1
ole_012036.T1, Maole_012037.T1, Maole_017285.T1, Maole_024088.T1
ole_020576.T1, Maole_022877.T1, Maole_023342.T1
ole_003834.T1, Maole_003836.T1, Maole_003945.T1, Maole_006532.T1, Maole_008212.T1,
ole_008100.T1, Maole_008101.T1
ole_004615.T1, Maole_005111.T1, Maole_005712.T1, Maole_005715.T1, Maole_012033.T1,
ole_004615.T1, Maole_005111.T1, Maole_005712.T1, Maole_005715.T1, Maole_012033.T1,
ole_010276.T1, Maole_021480.T1
ole_013992.T1, Maole_013993.T1, Maole_013994.T1, Maole_014003.T1
ole_004615.T1, Maole_005111.T1, Maole_005712.T1, Maole_005715.T1, Maole_012033.T1,
ole_021342.T1, Maole_022379.T1, Maole_023736.T1, Maole_023737.T1, Maole_023741.T1,
ole_009743.T1, Maole_014799.T1, Maole_017628.T1, Maole_018434.T1, Maole_020950.T1,
ole_020576.T1, Maole_022877.T1, Maole_023342.T1
ole_020460.T1, Maole_021816.T1
ole_023574.T1
ole_023574.T1
ole_012036.T1, Maole_012037.T1, Maole_017285.T1, Maole_024088.T1
ole_000953.T1, Maole_000954.T1, Maole_003004.T1, Maole_003005.T1, Maole_003774.T1,
ole_010787.T1, Maole_010789.T1
ole_023241.T1, Maole_023344.T1, Maole_023347.T1, Maole_023983.T1, Maole_023998.T1,
ole_013909.T1, Maole_013911.T1, Maole_013912.T1, Maole_013913.T1, Maole_013914.T1,
ole_000677.T1, Maole_001434.T1, Maole_002027.T1, Maole_003161.T1, Maole_003875.T1,

ole_003786.T1, Maole_003790.T1, Maole_003791.T1, Maole_003793.T1, Maole_003796.T1,
ole_003786.T1, Maole_003790.T1, Maole_003791.T1, Maole_003793.T1, Maole_003796.T1,
ole_010533.T1

ole_013807.T1, Maole_013808.T1
ole_000953.T1, Maole_000954.T1, Maole_002491.T1, Maole_002978.T1, Maole_002999.T1,
ole_008100.T1, Maole_008101.T1

ole_024112.T1
ole_012036.T1, Maole_012037.T1, Maole_017285.T1, Maole_024088.T1
ole_016084.T1, Maole_016085.T1, Maole_021078.T1, Maole_021081.T1
ole_002181.T1, Maole_002183.T1, Maole_002318.T1, Maole_002319.T1, Maole_002320.T1,
ole_002181.T1, Maole_002183.T1, Maole_002318.T1, Maole_002319.T1, Maole_002320.T1,
ole_011061.T1, Maole_011062.T1, Maole_013627.T1, Maole_020845.T1, Maole_020847.T1,
ole_007909.T1, Maole_008161.T1, Maole_017777.T1, Maole_020576.T1, Maole_022877.T1,
ole_005259.T1, Maole_007733.T1, Maole_015424.T1, Maole_015426.T1, Maole_015427.T1,
ole_006990.T1, Maole_021600.T1
ole_003808.T1, Maole_005120.T1, Maole_005178.T1, Maole_005179.T1, Maole_005180.T1,
ole_008100.T1, Maole_008101.T1, Maole_015699.T1, Maole_015702.T1, Maole_015705.T1
ole_003197.T1, Maole_005149.T1, Maole_015936.T1, Maole_015986.T1, Maole_016870.T1,
ole_015649.T1, Maole_020266.T1, Maole_020268.T1, Maole_023486.T1, Maole_023487.T1,
ole_003791.T1, Maole_003793.T1, Maole_003796.T1, Maole_019618.T1

ole_015768.T1
ole_012036.T1, Maole_012037.T1, Maole_017285.T1, Maole_024088.T1
ole_023580.T1

ole_008212.T1, Maole_021175.T1, Maole_021594.T1

ole_013807.T1, Maole_013808.T1

ole_001428.T1, Maole_001434.T1, Maole_001560.T1, Maole_002027.T1, Maole_002978.T1,
ole_006598.T1, Maole_006603.T1, Maole_006604.T1, Maole_008476.T1, Maole_016084.T1,
ole_016084.T1, Maole_016085.T1, Maole_021078.T1, Maole_021081.T1, Maole_021238.T1,
ole_005259.T1, Maole_007733.T1, Maole_010272.T1, Maole_010273.T1, Maole_010274.T1,

ole_012177.T1, Maole_012178.T1, Maole_015768.T1, Maole_020036.T1, Maole_022028.T1,
ole_004489.T1, Maole_004491.T1, Maole_006869.T1, Maole_010845.T1, Maole_010846.T1
ole_003102.T1, Maole_004233.T1, Maole_006598.T1, Maole_006603.T1, Maole_006604.T1,

ole_000162.T1, Maole_000211.T1, Maole_000256.T1, Maole_000311.T1, Maole_000313.T1,
ole_000100.T1, Maole_000132.T1, Maole_000162.T1, Maole_000198.T1, Maole_000211.T1,
ole_000080.T1, Maole_000100.T1, Maole_000162.T1, Maole_000311.T1, Maole_000313.T1,
ole_000379.T1, Maole_000381.T1, Maole_000473.T1, Maole_000517.T1, Maole_000518.T1,
ole_000110.T1, Maole_000116.T1, Maole_000328.T1, Maole_000379.T1, Maole_000381.T1,
ole_000473.T1, Maole_000519.T1, Maole_000690.T1, Maole_000698.T1, Maole_000893.T1,
ole_000473.T1, Maole_000519.T1, Maole_000690.T1, Maole_000698.T1, Maole_000893.T1,
ole_000519.T1, Maole_000690.T1, Maole_000698.T1, Maole_000839.T1, Maole_000893.T1,
ole_000473.T1, Maole_000519.T1, Maole_000690.T1, Maole_000698.T1, Maole_000893.T1,
ole_000473.T1, Maole_000519.T1, Maole_000690.T1, Maole_000698.T1, Maole_000893.T1,
ole_000473.T1, Maole_000518.T1, Maole_000519.T1, Maole_000690.T1, Maole_000698.T1,
ole_000328.T1, Maole_000493.T1, Maole_000497.T1, Maole_000619.T1, Maole_000643.T1,
ole_000455.T1, Maole_000473.T1, Maole_000517.T1, Maole_000518.T1, Maole_000519.T1,
ole_000517.T1, Maole_000518.T1, Maole_000519.T1, Maole_000839.T1, Maole_001008.T1,
ole_000518.T1, Maole_000519.T1, Maole_000806.T1, Maole_002899.T1, Maole_004432.T1,
ole_001538.T1, Maole_002313.T1, Maole_002510.T1, Maole_002556.T1, Maole_002862.T1,
ole_001631.T1, Maole_002396.T1, Maole_002742.T1, Maole_002747.T1, Maole_002777.T1,
ole_000749.T1, Maole_000806.T1, Maole_000934.T1, Maole_000945.T1, Maole_001020.T1,
ole_000803.T1, Maole_000806.T1, Maole_000934.T1, Maole_001037.T1, Maole_001096.T1,
ole_000518.T1, Maole_000519.T1, Maole_004432.T1, Maole_006992.T1, Maole_007381.T1,
ole_000735.T1, Maole_000886.T1, Maole_000922.T1, Maole_001108.T1, Maole_001488.T1,
ole_000747.T1, Maole_000749.T1, Maole_000806.T1, Maole_001432.T1, Maole_001481.T1,
ole_000922.T1, Maole_001108.T1, Maole_001137.T1, Maole_001227.T1, Maole_001356.T1,
ole_006773.T1, Maole_006774.T1, Maole_007462.T1, Maole_007590.T1, Maole_007834.T1,
ole_001757.T1, Maole_002721.T1, Maole_002724.T1, Maole_002777.T1, Maole_002782.T1,
ole_000474.T1, Maole_000497.T1, Maole_000558.T1, Maole_000672.T1, Maole_000806.T1,
ole_000749.T1, Maole_001432.T1, Maole_001538.T1, Maole_001722.T1, Maole_001986.T1,
ole_002240.T1, Maole_002389.T1, Maole_002693.T1, Maole_002707.T1, Maole_003227.T1,
ole_000612.T1, Maole_000619.T1, Maole_000643.T1, Maole_000644.T1, Maole_000864.T1,
ole_000922.T1, Maole_001108.T1, Maole_001561.T1, Maole_001566.T1, Maole_001609.T1,
ole_000497.T1, Maole_000613.T1, Maole_000922.T1, Maole_001360.T1, Maole_001992.T1,
ole_000806.T1, Maole_001020.T1, Maole_001021.T1, Maole_001022.T1, Maole_001316.T1,
ole_001022.T1, Maole_001931.T1, Maole_004881.T1, Maole_004982.T1, Maole_011288.T1,
ole_002648.T1, Maole_003245.T1, Maole_004210.T1, Maole_004626.T1, Maole_006703.T1,
ole_004215.T1, Maole_004810.T1, Maole_005654.T1, Maole_005846.T1, Maole_006203.T1,
ole_000473.T1, Maole_000844.T1, Maole_001426.T1, Maole_002239.T1, Maole_002556.T1,
ole_004982.T1, Maole_005925.T1, Maole_006437.T1, Maole_008596.T1, Maole_011110.T1,
ole_003000.T1, Maole_004301.T1, Maole_006692.T1, Maole_006693.T1, Maole_006694.T1,
ole_006386.T1, Maole_006896.T1, Maole_009465.T1, Maole_010599.T1, Maole_011873.T1,
ole_001020.T1, Maole_001021.T1, Maole_001022.T1, Maole_001277.T1, Maole_001467.T1,
ole_009317.T1, Maole_009320.T1, Maole_012158.T1, Maole_014265.T1, Maole_014267.T1,
ole_002648.T1, Maole_003245.T1, Maole_004210.T1, Maole_004626.T1, Maole_006703.T1,

ole_001106.T1, Maole_001610.T1, Maole_002239.T1, Maole_002556.T1, Maole_003232.T1,
ole_002782.T1, Maole_003283.T1, Maole_003326.T1, Maole_003557.T1, Maole_003560.T1,
ole_001631.T1, Maole_001879.T1, Maole_002070.T1, Maole_002652.T1, Maole_002762.T1,
ole_000643.T1, Maole_000644.T1, Maole_001106.T1, Maole_001123.T1, Maole_001124.T1,
ole_003604.T1, Maole_003605.T1, Maole_003607.T1, Maole_005101.T1, Maole_006559.T1,
ole_005388.T1, Maole_007462.T1, Maole_007590.T1, Maole_009364.T1, Maole_009509.T1,
ole_001931.T1, Maole_004980.T1, Maole_004982.T1, Maole_005385.T1, Maole_007944.T1,
ole_009320.T1, Maole_014265.T1, Maole_014267.T1, Maole_014269.T1, Maole_014270.T1,
ole_000490.T1, Maole_000564.T1, Maole_000636.T1, Maole_000643.T1, Maole_000644.T1,
ole_001210.T1, Maole_001277.T1, Maole_001432.T1, Maole_002056.T1, Maole_002146.T1,
ole_001404.T1, Maole_001854.T1, Maole_001931.T1, Maole_002240.T1, Maole_002591.T1,
ole_006885.T1, Maole_009289.T1, Maole_009320.T1, Maole_009432.T1, Maole_010021.T1,
ole_001126.T1, Maole_001380.T1, Maole_001610.T1, Maole_001631.T1, Maole_002381.T1,
ole_002782.T1, Maole_005818.T1, Maole_006779.T1, Maole_007101.T1, Maole_007703.T1,
ole_001931.T1, Maole_004982.T1, Maole_007944.T1, Maole_011159.T1, Maole_011357.T1,
ole_005654.T1, Maole_005846.T1, Maole_008020.T1, Maole_009817.T1, Maole_016461.T1,
ole_001432.T1, Maole_001722.T1, Maole_001947.T1, Maole_002264.T1, Maole_003027.T1,
ole_002777.T1, Maole_002782.T1, Maole_002792.T1, Maole_003326.T1, Maole_003848.T1,
ole_006933.T1, Maole_007270.T1, Maole_007272.T1, Maole_009100.T1, Maole_009213.T1,
ole_002221.T1, Maole_002411.T1, Maole_002620.T1, Maole_003656.T1, Maole_003976.T1,
ole_001210.T1, Maole_004167.T1, Maole_006194.T1, Maole_006295.T1, Maole_008763.T1,
ole_002313.T1, Maole_002477.T1, Maole_002510.T1, Maole_003000.T1, Maole_003084.T1,
ole_008959.T1, Maole_011670.T1, Maole_012116.T1, Maole_013130.T1, Maole_014477.T1,
ole_001301.T1, Maole_001488.T1, Maole_001566.T1, Maole_001631.T1, Maole_003987.T1,
ole_006692.T1, Maole_006693.T1, Maole_006694.T1, Maole_006695.T1, Maole_006799.T1,
ole_001021.T1, Maole_001022.T1, Maole_001277.T1, Maole_001310.T1, Maole_001426.T1,
ole_002591.T1, Maole_002950.T1, Maole_003000.T1, Maole_003848.T1, Maole_004073.T1,
ole_003085.T1, Maole_004215.T1, Maole_005654.T1, Maole_005846.T1, Maole_006444.T1,
ole_002783.T1, Maole_005453.T1, Maole_006085.T1, Maole_006235.T1, Maole_006236.T1,
ole_009364.T1, Maole_009509.T1, Maole_009644.T1, Maole_011710.T1, Maole_012158.T1,
ole_003203.T1, Maole_005013.T1, Maole_006581.T1, Maole_006583.T1, Maole_007599.T1,
ole_009509.T1, Maole_009644.T1, Maole_011710.T1, Maole_012158.T1, Maole_012507.T1,
ole_000737.T1, Maole_000932.T1, Maole_001277.T1, Maole_001310.T1, Maole_001610.T1,
ole_006695.T1, Maole_009102.T1, Maole_015626.T1, Maole_020423.T1, Maole_022732.T1,
ole_006695.T1, Maole_009102.T1, Maole_015626.T1, Maole_020423.T1, Maole_022732.T1,
ole_001091.T1, Maole_001137.T1, Maole_001757.T1, Maole_002102.T1, Maole_002742.T1,
ole_003204.T1, Maole_004697.T1, Maole_005064.T1, Maole_005344.T1, Maole_005531.T1,
ole_002556.T1, Maole_003232.T1, Maole_003905.T1, Maole_004230.T1, Maole_004334.T1,
ole_002239.T1, Maole_003232.T1, Maole_003369.T1, Maole_003426.T1, Maole_004896.T1,
ole_000465.T1, Maole_000672.T1, Maole_001106.T1, Maole_001108.T1, Maole_001210.T1,
ole_006830.T1, Maole_009242.T1, Maole_013596.T1, Maole_015073.T1, Maole_015516.T1,
ole_001561.T1, Maole_002003.T1, Maole_002433.T1, Maole_002693.T1, Maole_003848.T1,
ole_002556.T1, Maole_003232.T1, Maole_003905.T1, Maole_004230.T1, Maole_005232.T1,
ole_001123.T1, Maole_001124.T1, Maole_001125.T1, Maole_001209.T1, Maole_001210.T1,
ole_000922.T1, Maole_000995.T1, Maole_001201.T1, Maole_002689.T1, Maole_002818.T1,
ole_002389.T1, Maole_002556.T1, Maole_003203.T1, Maole_003496.T1, Maole_003754.T1,
ole_002556.T1, Maole_003232.T1, Maole_003905.T1, Maole_004230.T1, Maole_006790.T1,
ole_002742.T1, Maole_004230.T1, Maole_004704.T1, Maole_005307.T1, Maole_006574.T1,

ole_003283.T1, Maole_003557.T1, Maole_003560.T1, Maole_003994.T1, Maole_005013.T1,
ole_003560.T1, Maole_003905.T1, Maole_006885.T1, Maole_007703.T1, Maole_008182.T1,
ole_012595.T1, Maole_012596.T1, Maole_012598.T1, Maole_015802.T1, Maole_016455.T1,
ole_005846.T1, Maole_008020.T1, Maole_009817.T1, Maole_016461.T1, Maole_016463.T1,
ole_006695.T1, Maole_015626.T1, Maole_020423.T1, Maole_022732.T1, Maole_022733.T1,
ole_006695.T1, Maole_009102.T1, Maole_015626.T1, Maole_020423.T1, Maole_022732.T1,
ole_007599.T1, Maole_009063.T1, Maole_009534.T1, Maole_011321.T1, Maole_011322.T1,
ole_002689.T1, Maole_003437.T1, Maole_005911.T1, Maole_006168.T1, Maole_008616.T1,
ole_000418.T1, Maole_000886.T1, Maole_000995.T1, Maole_001100.T1, Maole_001375.T1,
ole_005925.T1, Maole_007391.T1, Maole_008670.T1, Maole_011601.T1, Maole_011761.T1,
ole_002365.T1, Maole_002389.T1, Maole_002408.T1, Maole_002693.T1, Maole_003444.T1,
ole_003605.T1, Maole_003607.T1, Maole_003920.T1, Maole_004237.T1, Maole_004874.T1,
ole_009725.T1, Maole_015802.T1, Maole_019177.T1, Maole_019303.T1, Maole_021844.T1,
ole_008447.T1, Maole_008616.T1, Maole_014867.T1, Maole_017163.T1, Maole_017313.T1,
ole_003989.T1, Maole_004942.T1, Maole_010427.T1, Maole_011595.T1, Maole_011678.T1,
ole_002390.T1, Maole_002591.T1, Maole_002816.T1, Maole_003084.T1, Maole_005483.T1,
ole_003085.T1, Maole_004215.T1, Maole_004554.T1, Maole_005654.T1, Maole_005846.T1,
ole_001935.T1, Maole_001957.T1, Maole_002202.T1, Maole_002627.T1, Maole_003562.T1,
ole_016686.T1, Maole_019159.T1, Maole_019300.T1, Maole_020423.T1, Maole_022732.T1,
ole_012828.T1, Maole_014831.T1, Maole_014832.T1, Maole_015464.T1, Maole_015610.T1,
ole_006692.T1, Maole_006693.T1, Maole_006694.T1, Maole_006695.T1, Maole_006799.T1,
ole_006693.T1, Maole_006694.T1, Maole_006695.T1, Maole_014397.T1, Maole_015626.T1,
ole_000644.T1, Maole_000870.T1, Maole_000932.T1, Maole_001310.T1, Maole_001422.T1,
ole_006695.T1, Maole_015626.T1, Maole_020423.T1, Maole_022732.T1, Maole_022733.T1,
ole_011110.T1, Maole_011111.T1, Maole_011159.T1, Maole_012819.T1, Maole_013561.T1,
ole_007145.T1, Maole_008181.T1, Maole_011198.T1, Maole_011377.T1, Maole_011378.T1,
ole_006386.T1, Maole_006896.T1, Maole_009465.T1, Maole_009883.T1, Maole_010599.T1,
ole_004430.T1, Maole_004565.T1, Maole_005543.T1, Maole_006169.T1, Maole_006681.T1,
ole_003557.T1, Maole_003905.T1, Maole_004167.T1, Maole_004878.T1, Maole_005102.T1,
ole_007270.T1, Maole_007272.T1, Maole_009100.T1, Maole_010479.T1, Maole_011181.T1,
ole_002221.T1, Maole_003656.T1, Maole_003754.T1, Maole_003805.T1, Maole_004874.T1,
ole_002202.T1, Maole_003315.T1, Maole_004334.T1, Maole_004457.T1, Maole_004704.T1,
ole_011321.T1, Maole_011322.T1, Maole_016343.T1, Maole_016345.T1, Maole_020028.T1,
ole_001022.T1, Maole_001277.T1, Maole_001578.T1, Maole_002495.T1, Maole_003007.T1,
ole_001757.T1, Maole_002102.T1, Maole_002707.T1, Maole_003283.T1, Maole_007074.T1,
ole_008616.T1, Maole_010934.T1, Maole_012144.T1, Maole_012145.T1, Maole_016155.T1,
ole_001631.T1, Maole_001639.T1, Maole_002469.T1, Maole_002634.T1, Maole_003557.T1,
ole_002240.T1, Maole_002689.T1, Maole_002693.T1, Maole_003227.T1, Maole_003498.T1,
ole_001124.T1, Maole_001125.T1, Maole_001277.T1, Maole_001310.T1, Maole_001424.T1,
ole_007749.T1, Maole_011444.T1, Maole_017089.T1, Maole_017128.T1, Maole_018033.T1,
ole_002207.T1, Maole_004430.T1, Maole_004565.T1, Maole_005543.T1, Maole_006169.T1,
ole_001757.T1, Maole_002236.T1, Maole_003560.T1, Maole_003905.T1, Maole_004255.T1,
ole_003560.T1, Maole_005013.T1, Maole_005101.T1, Maole_006559.T1, Maole_006688.T1,
ole_017868.T1, Maole_017870.T1, Maole_017871.T1, Maole_017874.T1
ole_002313.T1, Maole_002510.T1, Maole_002618.T1, Maole_002924.T1, Maole_003000.T1,
ole_019303.T1, Maole_022244.T1, Maole_022842.T1, Maole_022844.T1
ole_012596.T1, Maole_012598.T1, Maole_021844.T1, Maole_024010.T1
ole_005935.T1, Maole_013454.T1, Maole_013456.T1, Maole_014963.T1, Maole_014964.T1,

ole_012448.T1, Maole_012507.T1, Maole_012857.T1, Maole_013030.T1, Maole_013163.T1,
ole_019762.T1, Maole_019764.T1, Maole_019766.T1, Maole_019767.T1
ole_002510.T1, Maole_003000.T1, Maole_003084.T1, Maole_003989.T1, Maole_004942.T1,
ole_002510.T1, Maole_003000.T1, Maole_003084.T1, Maole_003989.T1, Maole_004942.T1,
ole_002510.T1, Maole_003000.T1, Maole_003084.T1, Maole_003989.T1, Maole_004942.T1,
ole_012276.T1, Maole_012537.T1, Maole_013721.T1, Maole_015061.T1
ole_002239.T1, Maole_002588.T1, Maole_002636.T1, Maole_002645.T1, Maole_002648.T1,
ole_001919.T1, Maole_001920.T1, Maole_002124.T1, Maole_003601.T1, Maole_003602.T1,
ole_009724.T1, Maole_011052.T1, Maole_014080.T1, Maole_014832.T1, Maole_015610.T1,
ole_001608.T1, Maole_002704.T1, Maole_002715.T1, Maole_002728.T1, Maole_002884.T1,
ole_012448.T1, Maole_013030.T1, Maole_019253.T1, Maole_019256.T1, Maole_019257.T1
ole_012448.T1, Maole_013030.T1, Maole_019253.T1, Maole_019256.T1, Maole_019257.T1
ole_012448.T1, Maole_013030.T1, Maole_019253.T1, Maole_019256.T1, Maole_019257.T1
ole_002029.T1, Maole_003601.T1, Maole_003602.T1, Maole_003604.T1, Maole_003605.T1,
ole_009636.T1, Maole_016612.T1, Maole_016977.T1, Maole_016978.T1, Maole_023496.T1,
ole_001854.T1, Maole_004862.T1, Maole_008809.T1, Maole_009636.T1, Maole_010016.T1,
ole_004142.T1, Maole_004721.T1, Maole_006480.T1, Maole_007285.T1, Maole_007473.T1,
ole_001826.T1, Maole_004927.T1, Maole_005453.T1, Maole_005574.T1, Maole_006203.T1,
ole_009320.T1, Maole_014265.T1, Maole_014267.T1, Maole_014269.T1, Maole_014270.T1,
ole_002761.T1, Maole_003602.T1, Maole_003848.T1, Maole_004972.T1, Maole_009317.T1,
ole_001535.T1, Maole_002003.T1, Maole_002070.T1, Maole_003218.T1, Maole_004412.T1,
ole_003886.T1, Maole_004301.T1, Maole_006692.T1, Maole_006693.T1, Maole_006694.T1,
ole_001920.T1, Maole_003601.T1, Maole_003602.T1, Maole_003604.T1, Maole_003605.T1,
ole_002510.T1, Maole_003084.T1, Maole_003989.T1, Maole_004942.T1, Maole_009968.T1,
ole_007391.T1, Maole_008670.T1, Maole_008854.T1, Maole_011601.T1, Maole_011761.T1,
ole_003886.T1, Maole_006692.T1, Maole_006693.T1, Maole_006694.T1, Maole_006695.T1,
ole_006670.T1, Maole_006766.T1, Maole_007126.T1, Maole_007170.T1, Maole_008105.T1,
ole_006040.T1, Maole_011930.T1, Maole_018021.T1, Maole_020499.T1, Maole_020500.T1,
ole_009636.T1, Maole_016612.T1, Maole_016977.T1, Maole_016978.T1, Maole_023496.T1
ole_004142.T1, Maole_004721.T1, Maole_006480.T1, Maole_007285.T1, Maole_007473.T1,
ole_000870.T1, Maole_001037.T1, Maole_001240.T1, Maole_002028.T1, Maole_004139.T1,
ole_004073.T1, Maole_005925.T1, Maole_010308.T1, Maole_016032.T1, Maole_016358.T1,
ole_000932.T1, Maole_002239.T1, Maole_003100.T1, Maole_003232.T1, Maole_003369.T1,
ole_011216.T1, Maole_018279.T1, Maole_020321.T1
ole_006559.T1, Maole_013416.T1, Maole_016271.T1, Maole_016575.T1, Maole_016576.T1,
ole_020423.T1, Maole_022732.T1, Maole_022733.T1, Maole_022737.T1
ole_020423.T1, Maole_022732.T1, Maole_022733.T1, Maole_022737.T1
ole_003848.T1, Maole_004268.T1, Maole_005514.T1, Maole_011987.T1, Maole_012448.T1,
ole_023259.T1, Maole_023260.T1, Maole_023776.T1, Maole_024056.T1, Maole_024121.T1
ole_011813.T1, Maole_013561.T1, Maole_014744.T1, Maole_017309.T1, Maole_017713.T1
ole_011813.T1, Maole_013561.T1, Maole_014744.T1, Maole_017309.T1, Maole_017713.T1
ole_001920.T1, Maole_003601.T1, Maole_003602.T1, Maole_003604.T1, Maole_003605.T1,
ole_001920.T1, Maole_003601.T1, Maole_003602.T1, Maole_003604.T1, Maole_003605.T1,
ole_009038.T1, Maole_012805.T1, Maole_013299.T1, Maole_016166.T1, Maole_021727.T1
ole_011111.T1, Maole_011159.T1, Maole_012819.T1, Maole_013561.T1, Maole_014744.T1,
ole_011111.T1, Maole_011159.T1, Maole_013561.T1, Maole_014744.T1, Maole_017309.T1,
ole_001516.T1, Maole_002241.T1, Maole_002477.T1, Maole_002558.T1, Maole_002634.T1,
ole_002510.T1, Maole_003000.T1, Maole_003084.T1, Maole_003989.T1, Maole_004942.T1,

ole_010762.T1, Maole_010763.T1, Maole_013630.T1, Maole_017544.T1, Maole_019799.T1,
ole_002102.T1, Maole_002433.T1, Maole_003283.T1, Maole_007074.T1, Maole_007504.T1,
ole_020423.T1, Maole_022732.T1, Maole_022733.T1, Maole_022737.T1
ole_020423.T1, Maole_022732.T1, Maole_022733.T1, Maole_022737.T1
ole_011514.T1, Maole_011533.T1, Maole_011536.T1, Maole_018810.T1, Maole_019308.T1,
ole_004750.T1, Maole_011642.T1, Maole_017660.T1, Maole_017900.T1, Maole_018513.T1,
ole_022732.T1, Maole_022733.T1, Maole_022737.T1
ole_022732.T1, Maole_022733.T1, Maole_022737.T1
ole_022732.T1, Maole_022733.T1, Maole_022737.T1
ole_022732.T1, Maole_022733.T1, Maole_022737.T1
ole_022732.T1, Maole_022733.T1, Maole_022737.T1
ole_016914.T1, Maole_018122.T1, Maole_018123.T1, Maole_018139.T1
ole_016914.T1, Maole_018122.T1, Maole_018123.T1, Maole_018139.T1
ole_005925.T1, Maole_006040.T1, Maole_007602.T1, Maole_008868.T1, Maole_010241.T1,
ole_002783.T1, Maole_003315.T1, Maole_005453.T1, Maole_005607.T1, Maole_006085.T1,
ole_006095.T1, Maole_007723.T1, Maole_007826.T1, Maole_007979.T1, Maole_007982.T1,
ole_015760.T1, Maole_015762.T1, Maole_016010.T1
ole_001458.T1, Maole_001907.T1, Maole_001986.T1, Maole_001987.T1, Maole_002288.T1,
ole_014708.T1, Maole_020182.T1
ole_012008.T1, Maole_014209.T1, Maole_014584.T1, Maole_018577.T1, Maole_020639.T1,
ole_011163.T1, Maole_019167.T1, Maole_019219.T1, Maole_020819.T1, Maole_021520.T1,
ole_001919.T1, Maole_001920.T1, Maole_001968.T1, Maole_002003.T1, Maole_002950.T1,
ole_009725.T1, Maole_011813.T1, Maole_013408.T1, Maole_015802.T1, Maole_017713.T1,
ole_011378.T1, Maole_020605.T1, Maole_020610.T1, Maole_020826.T1, Maole_023519.T1,
ole_003920.T1, Maole_004554.T1, Maole_004874.T1, Maole_004880.T1, Maole_004982.T1,
ole_001516.T1, Maole_001971.T1, Maole_004164.T1, Maole_004385.T1, Maole_005101.T1,
ole_011678.T1, Maole_016696.T1, Maole_021905.T1, Maole_023851.T1
ole_009038.T1, Maole_009043.T1, Maole_012805.T1, Maole_013299.T1, Maole_013870.T1,
ole_017109.T1, Maole_018437.T1
ole_010021.T1, Maole_022187.T1, Maole_022607.T1
ole_002389.T1, Maole_002408.T1, Maole_002433.T1, Maole_002693.T1, Maole_005221.T1,
ole_003283.T1, Maole_003994.T1, Maole_005818.T1, Maole_007320.T1, Maole_009099.T1,
ole_002264.T1, Maole_002313.T1, Maole_002510.T1, Maole_003084.T1, Maole_003978.T1,
ole_018122.T1, Maole_018123.T1, Maole_018139.T1, Maole_023259.T1, Maole_023260.T1,
ole_007956.T1, Maole_011880.T1, Maole_012004.T1, Maole_020919.T1
ole_020896.T1
ole_005625.T1, Maole_005626.T1, Maole_005709.T1, Maole_008264.T1, Maole_018644.T1,
ole_010762.T1, Maole_013630.T1, Maole_017544.T1
ole_014702.T1, Maole_018279.T1, Maole_020321.T1
ole_007690.T1, Maole_013454.T1, Maole_016657.T1, Maole_016925.T1, Maole_021285.T1,
ole_000564.T1, Maole_000569.T1, Maole_000643.T1, Maole_000644.T1, Maole_000737.T1,
ole_004878.T1, Maole_004982.T1, Maole_005520.T1, Maole_005796.T1, Maole_006799.T1,
ole_023171.T1, Maole_024003.T1
ole_009636.T1, Maole_016612.T1, Maole_016977.T1, Maole_016978.T1, Maole_023496.T1,
ole_009636.T1, Maole_016612.T1, Maole_016977.T1, Maole_016978.T1, Maole_023496.T1,
ole_017544.T1, Maole_022398.T1
ole_002782.T1, Maole_003326.T1, Maole_006040.T1, Maole_006885.T1, Maole_008854.T1,
ole_008946.T1, Maole_010457.T1, Maole_011380.T1, Maole_013709.T1, Maole_016687.T1,

ole_018524.T1, Maole_022120.T1
ole_022842.T1, Maole_022844.T1
ole_007703.T1, Maole_009317.T1, Maole_014516.T1, Maole_015735.T1, Maole_015737.T1,
ole_020502.T1, Maole_022100.T1
ole_011288.T1, Maole_017673.T1
ole_013119.T1, Maole_013926.T1, Maole_016237.T1, Maole_017124.T1
ole_001404.T1, Maole_002276.T1, Maole_003232.T1, Maole_004161.T1, Maole_004164.T1,
ole_003322.T1, Maole_003592.T1, Maole_003848.T1, Maole_005152.T1, Maole_005741.T1,
ole_004750.T1, Maole_011163.T1, Maole_011642.T1, Maole_011716.T1, Maole_019320.T1,
ole_002816.T1, Maole_003000.T1, Maole_003203.T1, Maole_003978.T1, Maole_006386.T1,
ole_016665.T1
ole_009587.T1, Maole_014829.T1, Maole_016525.T1, Maole_018783.T1, Maole_023290.T1
ole_003356.T1, Maole_003886.T1, Maole_005012.T1, Maole_005902.T1, Maole_006211.T1,
ole_003978.T1, Maole_007342.T1, Maole_008874.T1, Maole_008875.T1, Maole_012283.T1,
ole_017586.T1, Maole_023762.T1, Maole_023849.T1
ole_017586.T1, Maole_023762.T1, Maole_023849.T1
ole_001137.T1, Maole_001639.T1, Maole_002747.T1, Maole_004627.T1, Maole_004673.T1,
ole_015667.T1, Maole_020436.T1, Maole_023262.T1
ole_018034.T1, Maole_018035.T1, Maole_019820.T1, Maole_022419.T1, Maole_023245.T1
ole_014829.T1
ole_002693.T1, Maole_002777.T1, Maole_002782.T1, Maole_003886.T1, Maole_005005.T1,
ole_023943.T1
ole_006203.T1, Maole_007386.T1, Maole_007757.T1, Maole_007758.T1, Maole_007927.T1,
ole_006559.T1, Maole_013870.T1, Maole_017331.T1, Maole_017679.T1, Maole_018459.T1,
ole_000473.T1, Maole_000690.T1, Maole_000698.T1, Maole_000803.T1, Maole_001757.T1,
ole_005880.T1, Maole_005935.T1, Maole_007827.T1, Maole_012162.T1, Maole_012375.T1,
ole_006670.T1, Maole_007126.T1, Maole_008105.T1, Maole_009096.T1, Maole_009918.T1,
ole_019406.T1, Maole_019409.T1, Maole_019412.T1, Maole_019422.T1
ole_016575.T1, Maole_016576.T1, Maole_018524.T1, Maole_022120.T1
ole_004881.T1, Maole_005846.T1, Maole_006203.T1, Maole_007360.T1, Maole_009025.T1,
ole_017922.T1, Maole_020499.T1, Maole_023564.T1, Maole_023753.T1
ole_001467.T1, Maole_001730.T1, Maole_001836.T1, Maole_002742.T1, Maole_004334.T1,
ole_008703.T1, Maole_009038.T1, Maole_012554.T1, Maole_012805.T1, Maole_013299.T1,
ole_014269.T1, Maole_014270.T1, Maole_014272.T1, Maole_014274.T1, Maole_014275.T1
ole_001422.T1, Maole_001616.T1, Maole_001968.T1, Maole_002003.T1, Maole_002136.T1,
ole_007749.T1, Maole_010114.T1, Maole_010115.T1, Maole_018033.T1, Maole_018034.T1,
ole_005343.T1, Maole_007590.T1, Maole_011401.T1, Maole_012022.T1, Maole_012857.T1,
ole_004885.T1, Maole_007956.T1, Maole_009027.T1, Maole_011880.T1, Maole_012004.T1,
ole_017092.T1
ole_001879.T1, Maole_002304.T1, Maole_003326.T1, Maole_003754.T1, Maole_004874.T1,
ole_001920.T1, Maole_003601.T1, Maole_003602.T1, Maole_003604.T1, Maole_003605.T1,
ole_017900.T1, Maole_023885.T1
ole_004412.T1, Maole_004771.T1, Maole_005807.T1, Maole_006444.T1, Maole_006445.T1,
ole_019478.T1
ole_007690.T1, Maole_016925.T1, Maole_021718.T1
ole_008706.T1, Maole_008854.T1, Maole_011377.T1, Maole_011378.T1, Maole_012617.T1,
ole_015762.T1, Maole_016010.T1
ole_011444.T1, Maole_016457.T1, Maole_017089.T1, Maole_021942.T1

ole_002556.T1, Maole_004942.T1, Maole_005343.T1, Maole_006199.T1, Maole_006799.T1,
ole_005110.T1, Maole_006703.T1, Maole_007427.T1, Maole_010114.T1, Maole_010115.T1,
ole_013988.T1, Maole_014629.T1, Maole_015078.T1, Maole_016517.T1, Maole_017104.T1,
ole_014370.T1, Maole_018100.T1, Maole_021130.T1
ole_006933.T1, Maole_007270.T1, Maole_007272.T1, Maole_009100.T1, Maole_010479.T1,
ole_017220.T1, Maole_017719.T1, Maole_021993.T1, Maole_023850.T1
ole_012805.T1, Maole_013299.T1, Maole_021727.T1
ole_011880.T1, Maole_012004.T1, Maole_020919.T1
ole_004598.T1, Maole_004704.T1, Maole_006521.T1, Maole_006574.T1, Maole_007898.T1,
ole_023851.T1
ole_023851.T1
ole_010308.T1, Maole_012375.T1, Maole_014228.T1, Maole_016473.T1, Maole_017476.T1,
ole_020979.T1
ole_020979.T1
ole_019303.T1, Maole_022842.T1
ole_006936.T1, Maole_007427.T1, Maole_007964.T1, Maole_009883.T1, Maole_012158.T1,
ole_002782.T1, Maole_006040.T1, Maole_006885.T1, Maole_010110.T1, Maole_011930.T1,
ole_004874.T1, Maole_004880.T1, Maole_005818.T1, Maole_006269.T1, Maole_006421.T1,
ole_022607.T1
ole_013616.T1, Maole_016086.T1, Maole_016087.T1, Maole_016088.T1, Maole_018928.T1,
ole_015116.T1, Maole_017452.T1, Maole_021416.T1, Maole_023259.T1, Maole_023260.T1,
ole_019766.T1, Maole_019767.T1
ole_019766.T1, Maole_019767.T1
ole_012598.T1, Maole_016455.T1
ole_012598.T1, Maole_016455.T1
ole_006693.T1, Maole_006694.T1, Maole_006695.T1, Maole_014397.T1
ole_002401.T1, Maole_002591.T1, Maole_006581.T1, Maole_006583.T1
ole_002401.T1, Maole_002591.T1, Maole_006581.T1, Maole_006583.T1
ole_002401.T1, Maole_002591.T1, Maole_006581.T1, Maole_006583.T1
ole_002401.T1, Maole_002591.T1, Maole_006581.T1, Maole_006583.T1
ole_021407.T1, Maole_021422.T1, Maole_023931.T1
ole_001561.T1, Maole_001610.T1, Maole_001931.T1, Maole_002003.T1, Maole_002070.T1,
ole_022983.T1, Maole_022984.T1
ole_019514.T1, Maole_021520.T1
ole_011357.T1, Maole_011358.T1, Maole_011512.T1, Maole_012931.T1, Maole_013184.T1,
ole_000856.T1, Maole_001011.T1, Maole_001394.T1, Maole_001512.T1, Maole_001514.T1,
ole_011642.T1, Maole_019320.T1
ole_020502.T1, Maole_022100.T1
ole_009038.T1, Maole_010336.T1, Maole_019580.T1, Maole_021727.T1, Maole_023299.T1
ole_004072.T1, Maole_004444.T1, Maole_004445.T1, Maole_007455.T1, Maole_007690.T1,
ole_023406.T1, Maole_023943.T1
ole_014267.T1, Maole_014592.T1, Maole_020819.T1
ole_017644.T1, Maole_019939.T1, Maole_023357.T1
ole_012828.T1, Maole_015464.T1, Maole_015610.T1
ole_001120.T1, Maole_002207.T1, Maole_006682.T1, Maole_008396.T1
ole_014228.T1, Maole_016473.T1, Maole_020517.T1
ole_010308.T1, Maole_012375.T1, Maole_014228.T1, Maole_016473.T1, Maole_017476.T1,
ole_021181.T1

ole_003813.T1, Maole_004051.T1, Maole_004164.T1, Maole_004785.T1, Maole_005101.T1,
ole_002761.T1
ole_023361.T1
ole_001022.T1
ole_015061.T1
ole_001022.T1
ole_008706.T1, Maole_008707.T1, Maole_020226.T1
ole_020896.T1
ole_011691.T1, Maole_011692.T1, Maole_011813.T1, Maole_013579.T1, Maole_014669.T1,
ole_017476.T1, Maole_020187.T1, Maole_020517.T1, Maole_020738.T1, Maole_023468.T1
ole_007910.T1, Maole_007923.T1, Maole_009180.T1, Maole_011216.T1, Maole_011626.T1,
ole_005344.T1, Maole_006356.T1, Maole_006692.T1, Maole_006693.T1, Maole_006694.T1,
ole_007101.T1, Maole_007703.T1, Maole_009505.T1, Maole_021063.T1
ole_007101.T1, Maole_007703.T1, Maole_009505.T1, Maole_021063.T1
ole_002634.T1, Maole_005005.T1, Maole_005902.T1, Maole_007236.T1, Maole_008053.T1,
ole_005940.T1, Maole_011211.T1, Maole_013449.T1, Maole_017660.T1, Maole_017679.T1,
ole_014708.T1, Maole_020182.T1, Maole_020384.T1, Maole_023283.T1
ole_001125.T1, Maole_001609.T1, Maole_001700.T1, Maole_002146.T1, Maole_002406.T1,
ole_010293.T1, Maole_013647.T1, Maole_013650.T1, Maole_014926.T1, Maole_017432.T1,

ole_013216.T1, Maole_016506.T1, Maole_021202.T1
ole_000687.T1, Maole_001907.T1, Maole_002202.T1, Maole_003754.T1, Maole_004136.T1,
ole_005612.T1, Maole_014517.T1, Maole_015016.T1, Maole_015679.T1, Maole_015838.T1,
ole_003283.T1, Maole_003994.T1, Maole_009099.T1, Maole_013408.T1, Maole_018391.T1,
ole_008181.T1, Maole_011198.T1, Maole_011589.T1, Maole_014603.T1, Maole_015140.T1,
ole_011357.T1, Maole_011358.T1, Maole_011512.T1, Maole_012931.T1, Maole_013184.T1,
ole_017214.T1, Maole_020499.T1, Maole_023564.T1, Maole_023753.T1
ole_005343.T1, Maole_005421.T1, Maole_005925.T1, Maole_006799.T1, Maole_012022.T1,
ole_008734.T1, Maole_009465.T1, Maole_011827.T1, Maole_012136.T1
ole_014835.T1, Maole_015354.T1, Maole_019453.T1, Maole_020655.T1, Maole_021573.T1,
ole_011953.T1, Maole_017166.T1, Maole_017261.T1, Maole_017653.T1, Maole_022283.T1
ole_017992.T1, Maole_018063.T1, Maole_021240.T1
ole_004445.T1, Maole_006712.T1, Maole_008396.T1, Maole_009091.T1, Maole_009522.T1,
ole_013926.T1, Maole_017124.T1
ole_001992.T1, Maole_002411.T1, Maole_003085.T1, Maole_003221.T1, Maole_004215.T1,
ole_004885.T1, Maole_007956.T1, Maole_011880.T1, Maole_012004.T1, Maole_020919.T1
ole_016271.T1, Maole_016575.T1, Maole_016576.T1, Maole_018524.T1, Maole_020092.T1
ole_000636.T1, Maole_002028.T1, Maole_002067.T1, Maole_003496.T1, Maole_004136.T1,
ole_020684.T1, Maole_022720.T1
ole_022187.T1
ole_019768.T1, Maole_019770.T1, Maole_021249.T1, Maole_021255.T1
ole_019768.T1, Maole_019770.T1, Maole_021249.T1, Maole_021255.T1
ole_002950.T1, Maole_003000.T1, Maole_004942.T1, Maole_005343.T1, Maole_011954.T1,
ole_001022.T1, Maole_002818.T1
ole_007827.T1, Maole_012162.T1, Maole_014199.T1, Maole_016541.T1, Maole_016542.T1
ole_007827.T1, Maole_012162.T1, Maole_014199.T1, Maole_016541.T1, Maole_016542.T1
ole_013121.T1, Maole_022760.T1

ole_007183.T1, Maole_010423.T1, Maole_010934.T1, Maole_012144.T1, Maole_012145.T1,
ole_008489.T1, Maole_008800.T1, Maole_010540.T1, Maole_013292.T1, Maole_014705.T1,
ole_012785.T1, Maole_013186.T1
ole_014829.T1
ole_007074.T1, Maole_023299.T1
ole_011444.T1, Maole_017089.T1, Maole_021942.T1

ole_018524.T1, Maole_022120.T1
ole_008890.T1, Maole_010735.T1, Maole_012400.T1, Maole_012797.T1, Maole_014519.T1,
ole_014142.T1, Maole_014250.T1
ole_001419.T1, Maole_001879.T1, Maole_002003.T1, Maole_002433.T1, Maole_003326.T1,
ole_003543.T1, Maole_005344.T1, Maole_005531.T1, Maole_005534.T1, Maole_005535.T1,
ole_017087.T1, Maole_017092.T1
ole_017087.T1, Maole_017092.T1
ole_017087.T1, Maole_017092.T1
ole_003605.T1, Maole_003607.T1, Maole_016806.T1
ole_007656.T1, Maole_014553.T1, Maole_017220.T1
ole_012499.T1, Maole_012501.T1, Maole_013616.T1, Maole_018460.T1, Maole_018461.T1,
ole_007394.T1, Maole_010914.T1, Maole_015615.T1, Maole_015908.T1, Maole_015909.T1,
ole_002816.T1, Maole_003203.T1, Maole_007108.T1, Maole_009067.T1, Maole_009465.T1,
ole_010016.T1, Maole_012342.T1, Maole_015677.T1, Maole_016473.T1
ole_008489.T1, Maole_016016.T1, Maole_020426.T1, Maole_021181.T1
ole_004862.T1, Maole_010308.T1, Maole_012375.T1, Maole_013596.T1, Maole_014228.T1,

ole_005343.T1, Maole_017719.T1, Maole_021993.T1
ole_023357.T1
ole_012405.T1, Maole_020854.T1
ole_007827.T1, Maole_012162.T1, Maole_014199.T1, Maole_016541.T1, Maole_016542.T1,
ole_008616.T1, Maole_010934.T1, Maole_012144.T1, Maole_012145.T1, Maole_016155.T1,
ole_003557.T1, Maole_003560.T1, Maole_003994.T1, Maole_005102.T1, Maole_009505.T1,
ole_006702.T1, Maole_008292.T1, Maole_009473.T1, Maole_009758.T1, Maole_011161.T1,

ole_002777.T1, Maole_002782.T1, Maole_003326.T1, Maole_003625.T1, Maole_004073.T1,
ole_001037.T1, Maole_002029.T1, Maole_002826.T1, Maole_003688.T1, Maole_003773.T1,
ole_001616.T1, Maole_004385.T1, Maole_008809.T1, Maole_009636.T1, Maole_016612.T1,
ole_001163.T1, Maole_001631.T1, Maole_001826.T1, Maole_002381.T1, Maole_002556.T1,
ole_001125.T1, Maole_001240.T1, Maole_002240.T1, Maole_002406.T1, Maole_003227.T1,
ole_010762.T1, Maole_010763.T1, Maole_013630.T1, Maole_017544.T1, Maole_021768.T1,
ole_001516.T1, Maole_001649.T1, Maole_004385.T1, Maole_005343.T1, Maole_005612.T1,
ole_001037.T1, Maole_001123.T1, Maole_001124.T1, Maole_001125.T1, Maole_001481.T1,
ole_005654.T1, Maole_005846.T1, Maole_008020.T1, Maole_009817.T1, Maole_016461.T1,
ole_011110.T1, Maole_011111.T1, Maole_011159.T1, Maole_011401.T1, Maole_013561.T1,
ole_003356.T1, Maole_003562.T1, Maole_005483.T1, Maole_006351.T1, Maole_006933.T1,

ole_005180.T1, Maole_005694.T1, Maole_005695.T1, Maole_005698.T1, Maole_005700.T1,
ole_003161.T1, Maole_005120.T1, Maole_005178.T1, Maole_005179.T1, Maole_005180.T1,
ole_001022.T1, Maole_001931.T1, Maole_004881.T1, Maole_004982.T1, Maole_011288.T1,
ole_001020.T1, Maole_001021.T1, Maole_001022.T1, Maole_001096.T1, Maole_001375.T1,
ole_001434.T1, Maole_001560.T1, Maole_002027.T1, Maole_003161.T1, Maole_003875.T1,
ole_002648.T1, Maole_003245.T1, Maole_004210.T1, Maole_004626.T1, Maole_006703.T1,
ole_001434.T1, Maole_001538.T1, Maole_001560.T1, Maole_002027.T1, Maole_003001.T1,
ole_001931.T1, Maole_004980.T1, Maole_004982.T1, Maole_007944.T1, Maole_011110.T1,
ole_002648.T1, Maole_003245.T1, Maole_004210.T1, Maole_004626.T1, Maole_006703.T1,
ole_009320.T1, Maole_014265.T1, Maole_014267.T1, Maole_014269.T1, Maole_014270.T1,
ole_001434.T1, Maole_002027.T1, Maole_003161.T1, Maole_003875.T1, Maole_004490.T1,
ole_001434.T1, Maole_001560.T1, Maole_002027.T1, Maole_003001.T1, Maole_003161.T1,
ole_001931.T1, Maole_004982.T1, Maole_007944.T1, Maole_011159.T1, Maole_011357.T1,
ole_009320.T1, Maole_014265.T1, Maole_014267.T1, Maole_014269.T1, Maole_014270.T1,
ole_002591.T1, Maole_003069.T1, Maole_003305.T1, Maole_003945.T1, Maole_006532.T1,
ole_001434.T1, Maole_001560.T1, Maole_002027.T1, Maole_002249.T1, Maole_002250.T1,
ole_001434.T1, Maole_001560.T1, Maole_002027.T1, Maole_003001.T1, Maole_003161.T1,
ole_001931.T1, Maole_002245.T1, Maole_002247.T1, Maole_002253.T1, Maole_002591.T1,
ole_001434.T1, Maole_002027.T1, Maole_003161.T1, Maole_003875.T1, Maole_004490.T1,
ole_004972.T1, Maole_005013.T1, Maole_006647.T1, Maole_006651.T1, Maole_006652.T1,
ole_001021.T1, Maole_001022.T1, Maole_001096.T1, Maole_001375.T1, Maole_001461.T1,
ole_004972.T1, Maole_005013.T1, Maole_006647.T1, Maole_006651.T1, Maole_006652.T1,
ole_001022.T1, Maole_001375.T1, Maole_001424.T1, Maole_001461.T1, Maole_001538.T1,
ole_001434.T1, Maole_002027.T1, Maole_003161.T1, Maole_003875.T1, Maole_004490.T1,
ole_004972.T1, Maole_005013.T1, Maole_006647.T1, Maole_006651.T1, Maole_006652.T1,
ole_004972.T1, Maole_005013.T1, Maole_006647.T1, Maole_006651.T1, Maole_006652.T1,
ole_023241.T1, Maole_023344.T1, Maole_023347.T1, Maole_023983.T1, Maole_023998.T1,
ole_002648.T1, Maole_002978.T1, Maole_002999.T1, Maole_003050.T1, Maole_003245.T1,
ole_004972.T1, Maole_005013.T1, Maole_006647.T1, Maole_006651.T1, Maole_006652.T1,
ole_006532.T1, Maole_008212.T1, Maole_010105.T1, Maole_019117.T1, Maole_020721.T1,
ole_003197.T1, Maole_006779.T1, Maole_015116.T1, Maole_015936.T1, Maole_017068.T1,
ole_006532.T1, Maole_008212.T1, Maole_010105.T1, Maole_019117.T1, Maole_021175.T1,
ole_004972.T1, Maole_005013.T1, Maole_006647.T1, Maole_006651.T1, Maole_006652.T1,
ole_004982.T1, Maole_006532.T1, Maole_008212.T1, Maole_010105.T1, Maole_011288.T1,
ole_004972.T1, Maole_006532.T1, Maole_008212.T1, Maole_009317.T1, Maole_009320.T1,
ole_004972.T1, Maole_005013.T1, Maole_006647.T1, Maole_006651.T1, Maole_006652.T1,
ole_014265.T1, Maole_014267.T1, Maole_014269.T1, Maole_014270.T1, Maole_014272.T1,
ole_005180.T1, Maole_005694.T1, Maole_005695.T1, Maole_005698.T1, Maole_005700.T1,
ole_006532.T1, Maole_008212.T1, Maole_010105.T1, Maole_019117.T1, Maole_020721.T1,
ole_004972.T1, Maole_009317.T1, Maole_009320.T1, Maole_014265.T1, Maole_014267.T1,
ole_011111.T1, Maole_011159.T1, Maole_013561.T1, Maole_014744.T1, Maole_017309.T1,
ole_022500.T1, Maole_022504.T1, Maole_022507.T1, Maole_022510.T1, Maole_022511.T1,
ole_023259.T1, Maole_023260.T1, Maole_023776.T1, Maole_024056.T1, Maole_024121.T1
ole_023241.T1, Maole_023344.T1, Maole_023347.T1, Maole_023983.T1, Maole_023998.T1,
ole_023241.T1, Maole_023344.T1, Maole_023347.T1, Maole_023983.T1, Maole_023998.T1,
ole_023241.T1, Maole_023344.T1, Maole_023347.T1, Maole_023983.T1, Maole_023998.T1,
ole_007827.T1, Maole_012162.T1, Maole_014199.T1, Maole_016541.T1, Maole_016542.T1

ole_014269.T1, Maole_014270.T1, Maole_014272.T1, Maole_014274.T1, Maole_014275.T1
ole_003786.T1, Maole_003790.T1, Maole_003791.T1, Maole_003793.T1, Maole_019618.T1
ole_007827.T1, Maole_012162.T1, Maole_014199.T1, Maole_016541.T1, Maole_016542.T1
ole_001434.T1, Maole_002027.T1, Maole_003161.T1, Maole_003875.T1, Maole_004490.T1,
ole_022500.T1, Maole_022504.T1, Maole_022507.T1, Maole_022510.T1, Maole_022511.T1,
ole_002591.T1, Maole_003069.T1, Maole_003305.T1, Maole_003945.T1, Maole_005877.T1,
ole_005013.T1, Maole_006647.T1, Maole_006651.T1, Maole_006652.T1, Maole_006779.T1,
ole_011111.T1, Maole_011159.T1, Maole_013561.T1, Maole_014744.T1, Maole_017309.T1,
ole_023241.T1, Maole_023344.T1, Maole_023347.T1, Maole_023983.T1, Maole_023998.T1,
ole_002648.T1, Maole_003245.T1, Maole_004210.T1, Maole_004626.T1, Maole_006167.T1,
ole_020502.T1, Maole_022100.T1
ole_011288.T1, Maole_017673.T1
ole_007827.T1, Maole_012162.T1, Maole_014199.T1, Maole_016541.T1, Maole_016542.T1
ole_021342.T1, Maole_022379.T1, Maole_023736.T1, Maole_023737.T1, Maole_023741.T1,
ole_011111.T1, Maole_011159.T1, Maole_013561.T1, Maole_014744.T1, Maole_017309.T1,
ole_007827.T1, Maole_012162.T1, Maole_014199.T1, Maole_016541.T1, Maole_016542.T1
ole_023259.T1, Maole_023260.T1, Maole_023776.T1, Maole_024056.T1, Maole_024121.T1
ole_020502.T1, Maole_022100.T1
ole_012598.T1, Maole_016455.T1
ole_012598.T1, Maole_016455.T1
ole_003786.T1, Maole_003790.T1, Maole_003791.T1, Maole_003793.T1, Maole_003796.T1,
ole_003786.T1, Maole_003790.T1, Maole_003791.T1, Maole_003793.T1, Maole_003796.T1,
ole_013909.T1, Maole_013911.T1, Maole_013912.T1, Maole_013913.T1, Maole_013914.T1,
ole_001986.T1, Maole_001987.T1, Maole_002707.T1, Maole_002999.T1, Maole_003050.T1,
ole_013992.T1, Maole_013993.T1, Maole_013994.T1, Maole_014003.T1
ole_011111.T1, Maole_011159.T1, Maole_013561.T1, Maole_014744.T1, Maole_017309.T1,
ole_006652.T1, Maole_021063.T1, Maole_022507.T1
ole_006652.T1, Maole_021063.T1, Maole_022507.T1
ole_007827.T1, Maole_012162.T1, Maole_014199.T1, Maole_016541.T1, Maole_016542.T1
ole_021416.T1, Maole_023259.T1, Maole_023260.T1, Maole_023776.T1, Maole_024056.T1,
ole_022503.T1, Maole_023259.T1, Maole_023260.T1, Maole_023776.T1, Maole_024056.T1,
ole_001022.T1
ole_001022.T1
ole_021342.T1, Maole_022379.T1, Maole_023736.T1, Maole_023737.T1, Maole_023741.T1,
ole_010115.T1, Maole_010254.T1, Maole_010356.T1, Maole_010359.T1, Maole_010362.T1,
ole_003791.T1, Maole_003793.T1, Maole_003796.T1, Maole_019618.T1
ole_002253.T1, Maole_003197.T1, Maole_014199.T1, Maole_015936.T1, Maole_017068.T1
ole_006532.T1, Maole_008212.T1, Maole_010105.T1, Maole_019117.T1, Maole_020721.T1,
ole_006581.T1, Maole_006583.T1
ole_006581.T1, Maole_006583.T1
ole_006581.T1, Maole_006583.T1
ole_006581.T1, Maole_006583.T1
ole_012598.T1, Maole_016455.T1
ole_003945.T1, Maole_006532.T1, Maole_006581.T1, Maole_006583.T1, Maole_008212.T1,
ole_008212.T1, Maole_021175.T1, Maole_021594.T1
ole_006703.T1, Maole_010114.T1, Maole_010115.T1
ole_001022.T1, Maole_001096.T1, Maole_001545.T1, Maole_001931.T1, Maole_002591.T1,
ole_023259.T1, Maole_023260.T1, Maole_023776.T1, Maole_024121.T1

ole_002248.T1, Maole_002249.T1, Maole_002250.T1, Maole_002253.T1, Maole_002254.T1,
ole_007827.T1, Maole_012162.T1, Maole_014199.T1, Maole_016541.T1, Maole_016542.T1
ole_021342.T1, Maole_022379.T1, Maole_023736.T1, Maole_023737.T1, Maole_023741.T1,
ole_010359.T1, Maole_010362.T1, Maole_012789.T1, Maole_017647.T1
ole_003945.T1, Maole_008212.T1, Maole_015203.T1, Maole_021175.T1, Maole_021341.T1,
ole_001022.T1
ole_001022.T1, Maole_004881.T1, Maole_011288.T1, Maole_012520.T1, Maole_012595.T1,
ole_015116.T1, Maole_023259.T1, Maole_023260.T1, Maole_023776.T1, Maole_024056.T1,
ole_021342.T1, Maole_022379.T1, Maole_023736.T1, Maole_023737.T1, Maole_023741.T1,
ole_014199.T1, Maole_016541.T1, Maole_016542.T1
ole_002648.T1, Maole_003245.T1, Maole_003596.T1, Maole_010254.T1, Maole_010356.T1,
ole_002249.T1, Maole_002250.T1, Maole_002253.T1, Maole_002254.T1, Maole_002978.T1,

ole_001021.T1, Maole_001022.T1, Maole_001538.T1, Maole_001986.T1, Maole_001987.T1,
ole_011110.T1, Maole_011111.T1, Maole_011159.T1, Maole_011288.T1, Maole_013561.T1,
ole_017309.T1
ole_017309.T1
ole_007827.T1, Maole_012162.T1, Maole_014199.T1, Maole_016541.T1, Maole_016542.T1

ole_001022.T1, Maole_004881.T1, Maole_011288.T1, Maole_012520.T1, Maole_012595.T1,
ole_012089.T1, Maole_013992.T1, Maole_013993.T1, Maole_013994.T1, Maole_014003.T1
ole_024056.T1, Maole_024121.T1
ole_023259.T1, Maole_023260.T1, Maole_023776.T1, Maole_024056.T1, Maole_024121.T1
ole_016881.T1
ole_008212.T1, Maole_021175.T1, Maole_021594.T1
ole_003786.T1, Maole_003790.T1, Maole_003791.T1, Maole_003793.T1, Maole_003796.T1,

ole_022503.T1, Maole_023259.T1, Maole_023260.T1, Maole_023776.T1, Maole_024121.T1
ole_003197.T1, Maole_015936.T1, Maole_017068.T1
ole_002245.T1, Maole_002247.T1, Maole_002253.T1, Maole_003197.T1, Maole_015936.T1,
ole_023259.T1, Maole_023260.T1, Maole_023776.T1, Maole_024121.T1
ole_003305.T1, Maole_003945.T1, Maole_006532.T1, Maole_008212.T1, Maole_010105.T1,
ole_016881.T1
ole_006262.T1
ole_003791.T1, Maole_003793.T1, Maole_003796.T1, Maole_019618.T1, Maole_022510.T1,
ole_003197.T1, Maole_015936.T1, Maole_017068.T1

ole_021342.T1, Maole_022379.T1, Maole_023736.T1, Maole_023737.T1, Maole_023741.T1,
ole_006532.T1, Maole_008212.T1, Maole_010105.T1, Maole_015873.T1, Maole_019117.T1,
ole_012162.T1, Maole_013561.T1, Maole_014199.T1, Maole_016541.T1, Maole_016542.T1
ole_002248.T1, Maole_002249.T1, Maole_002250.T1, Maole_002253.T1, Maole_002254.T1,

ole_006652.T1, Maole_010336.T1, Maole_012520.T1, Maole_012595.T1, Maole_012596.T1,

ole_001987.T1, Maole_002591.T1, Maole_006581.T1, Maole_006583.T1, Maole_009465.T1,
ole_013992.T1, Maole_013993.T1, Maole_013994.T1, Maole_014003.T1
ole_021342.T1, Maole_022379.T1, Maole_023736.T1, Maole_023737.T1, Maole_023741.T1,
ole_002249.T1, Maole_002250.T1, Maole_002253.T1, Maole_002254.T1, Maole_002707.T1,

ole_007827.T1, Maole_012162.T1, Maole_014199.T1, Maole_016541.T1, Maole_016542.T1,

ole_001434.T1, Maole_001560.T1, Maole_002027.T1, Maole_002978.T1, Maole_003001.T1,
ole_011110.T1, Maole_011111.T1, Maole_011159.T1, Maole_011401.T1, Maole_013561.T1,
ole_006779.T1, Maole_009317.T1, Maole_009320.T1, Maole_014265.T1, Maole_014267.T1,
ole_006167.T1, Maole_006532.T1, Maole_008212.T1, Maole_010105.T1, Maole_012798.T1,

le_023232;Maole_023233;Maole_023234;Maole_023235;Maole_023236
le_016083;Maole_016084;Maole_016085;Maole_016086;Maole_016087;Maole_016088;Maol
le_017151;Maole_017152;Maole_017153;Maole_017154
le_008023;Maole_008024
le_006921;Maole_006922;Maole_006923;Maole_006924;Maole_006925;Maole_006926;Maol
le_008908;Maole_008909;Maole_008910;Maole_008911;Maole_008912;Maole_008913;Maol
le_012580;Maole_012581;Maole_012582;Maole_012583;Maole_012584;Maole_012585;Maol
le_017976;Maole_017977;Maole_017978;Maole_017979;Maole_017980;Maole_017981;Maol
le_017523;Maole_017524;Maole_017525;Maole_017526;Maole_017527;Maole_017528;Maol
le_015580;Maole_015581;Maole_015582;Maole_015583;Maole_015584;Maole_015585
le_019304;Maole_019305;Maole_019306;Maole_019307;Maole_019308;Maole_019309;Maol
le_017277;Maole_017278;Maole_017279;Maole_017280;Maole_017281;Maole_017282;Maol
le_001304;Maole_001305;Maole_001306;Maole_001307
le_007256;Maole_007257;Maole_007258;Maole_007259;Maole_007260;Maole_007261;Maol
le_020687;Maole_020688;Maole_020689

le_021739;Maole_021740;Maole_021741;Maole_021742
le_000815;Maole_000816;Maole_000817;Maole_000818;Maole_000819;Maole_000820;Maol
le_000960;Maole_000961;Maole_000962;Maole_000963;Maole_000964;Maole_000965;Maol
le_002122;Maole_002123;Maole_002124;Maole_002125;Maole_002126;Maole_002127;Maol
le_002172;Maole_002173;Maole_002174;Maole_002175;Maole_002176;Maole_002177;Maol
le_014444;Maole_014445;Maole_014446;Maole_014447;Maole_014448;Maole_014449;Maol
le_017615;Maole_017616;Maole_017617;Maole_017618
le_016875;Maole_016876;Maole_016877;Maole_016878

Clean Q2	Clean Q3	Average l	Multiple	Uniquely Mapped (M)
NA	NA	8,852	NA	NA
NA	NA	150	NA	NA
5.883(99.2	5.774(97.4	147.6	7.486(18.0	31.861(79.29 %)
6.812(99.3	6.667(97.1	145.8	0.726(1.54	45.286(96.15 %)
6.207(99.4	6.117(98.0	146.3	4.921(11.5	36.389(85.24 %)
6.014(99.2	5.886(97.1	146.2	1.027(2.48	39.583(95.44 %)
6.793(99.2	6.645(97.0	146.7	1.012(2.17	43.978(94.20 %)
4.365(99.5	4.229(96.4	98.3	3.871(8.67	4.603(10.31 %)
4.047(98.8	3.911(95.5	99.1	2.305(5.58	35.394(85.62 %)

Maole_005495.T1, Maole_005948.T1, Maole_006013.T1, Maole_006014.T1, Maole_006015.
Maole_005702.T1, Maole_007182.T1, Maole_007191.T1, Maole_007193.T1, Maole_007194.
Maole_003790.T1, Maole_003791.T1, Maole_003793.T1, Maole_003796.T1, Maole_004175.
Maole_003774.T1, Maole_005495.T1, Maole_005948.T1, Maole_006013.T1, Maole_006014.
Maole_005991.T1, Maole_006239.T1, Maole_006824.T1, Maole_008187.T1, Maole_009283.
Maole_007706.T1, Maole_009182.T1, Maole_009283.T1, Maole_009647.T1, Maole_010002.
Maole_005495.T1, Maole_006013.T1, Maole_006014.T1, Maole_006015.T1, Maole_006016.
Maole_004776.T1, Maole_005713.T1, Maole_007054.T1, Maole_009104.T1, Maole_009186.
Maole_010025.T1, Maole_012936.T1, Maole_013071.T1, Maole_015433.T1, Maole_016034.
Maole_012810.T1, Maole_012936.T1, Maole_016782.T1, Maole_020743.T1, Maole_021244.
Maole_003774.T1, Maole_004372.T1, Maole_004375.T1, Maole_004377.T1, Maole_005495.
Maole_000954.T1, Maole_001031.T1, Maole_001054.T1, Maole_001363.T1, Maole_001611.
Maole_018434.T1, Maole_020950.T1, Maole_020951.T1, Maole_021052.T1, Maole_022416.
Maole_020952.T1, Maole_023348.T1, Maole_023352.T1, Maole_023550.T1
Maole_003405.T1, Maole_003481.T1, Maole_003483.T1, Maole_003485.T1, Maole_003488.
Maole_005695.T1, Maole_005698.T1, Maole_005700.T1, Maole_005702.T1, Maole_007182.
Maole_005495.T1, Maole_006013.T1, Maole_006014.T1, Maole_006015.T1, Maole_006016.
Maole_009283.T1, Maole_012810.T1, Maole_012929.T1, Maole_012936.T1, Maole_016782.
Maole_018434.T1, Maole_019745.T1, Maole_020950.T1, Maole_020951.T1, Maole_020952.
Maole_012607.T1, Maole_015102.T1, Maole_016670.T1, Maole_016728.T1, Maole_016782.

Maole_020952.T1, Maole_023348.T1, Maole_023352.T1, Maole_023550.T1
Maole_009013.T1, Maole_013458.T1, Maole_013459.T1, Maole_014253.T1, Maole_016516.
Maole_002311.T1, Maole_002312.T1, Maole_002491.T1, Maole_002809.T1, Maole_002999.
Maole_015426.T1, Maole_015427.T1, Maole_017521.T1, Maole_019116.T1
Maole_021135.T1, Maole_024088.T1
Maole_003750.T1, Maole_003751.T1, Maole_003752.T1, Maole_005746.T1, Maole_005748.
Maole_024019.T1, Maole_024051.T1, Maole_024069.T1, Maole_024098.T1
Maole_016516.T1, Maole_019870.T1, Maole_022795.T1

Maole_007887.T1, Maole_007888.T1
Maole_008097.T1, Maole_008098.T1, Maole_008100.T1, Maole_008101.T1, Maole_016629.
Maole_021178.T1, Maole_021594.T1
Maole_005194.T1, Maole_005746.T1, Maole_005748.T1, Maole_006987.T1, Maole_007919.
Maole_004056.T1, Maole_004057.T1, Maole_004058.T1, Maole_004207.T1, Maole_004208.
Maole_002027.T1, Maole_003001.T1, Maole_003161.T1, Maole_003875.T1, Maole_004490.
Maole_006379.T1, Maole_006380.T1, Maole_006824.T1, Maole_007032.T1, Maole_007334.
Maole_019808.T1
Maole_001767.T1, Maole_002027.T1, Maole_002301.T1, Maole_003001.T1, Maole_003161.
Maole_003005.T1, Maole_003069.T1, Maole_003305.T1, Maole_003774.T1, Maole_003945.
Maole_002321.T1, Maole_002322.T1, Maole_002329.T1, Maole_002335.T1, Maole_002340.
Maole_004776.T1, Maole_005499.T1, Maole_005713.T1, Maole_007054.T1, Maole_009104.

Maole_023241.T1, Maole_023344.T1, Maole_023347.T1, Maole_023400.T1, Maole_023402.
Maole_004776.T1, Maole_005713.T1, Maole_007054.T1, Maole_009104.T1, Maole_009186.
Maole_021178.T1, Maole_021594.T1
Maole_005495.T1, Maole_006013.T1, Maole_006014.T1, Maole_006015.T1, Maole_006016.
Maole_002978.T1, Maole_003161.T1, Maole_003786.T1, Maole_003790.T1, Maole_003791.
Maole_022513.T1
Maole_002321.T1, Maole_002322.T1, Maole_002329.T1, Maole_002335.T1, Maole_002340.
Maole_020951.T1, Maole_021052.T1, Maole_023348.T1, Maole_023352.T1, Maole_023550.
Maole_019808.T1
Maole_004614.T1, Maole_004615.T1, Maole_005111.T1, Maole_005712.T1, Maole_005715.
Maole_001054.T1, Maole_001212.T1, Maole_001213.T1, Maole_001279.T1, Maole_001461.
Maole_021816.T1, Maole_022795.T1

Maole_021178.T1, Maole_021594.T1
Maole_003875.T1, Maole_004490.T1, Maole_004776.T1, Maole_005062.T1, Maole_005713.

Maole_015774.T1, Maole_017521.T1, Maole_017527.T1
Maole_023348.T1, Maole_023352.T1, Maole_023550.T1
Maole_019750.T1

Maole_022513.T1
Maole_024019.T1, Maole_024051.T1, Maole_024069.T1, Maole_024098.T1
Maole_019080.T1, Maole_019808.T1

Maole_024019.T1, Maole_024051.T1, Maole_024069.T1, Maole_024098.T1
Maole_024019.T1, Maole_024051.T1, Maole_024069.T1, Maole_024098.T1
Maole_000954.T1, Maole_001031.T1, Maole_001047.T1, Maole_001766.T1, Maole_001983.

Maole_016516.T1, Maole_019829.T1, Maole_019870.T1, Maole_020460.T1, Maole_021816.

Maole_003161.T1, Maole_003875.T1, Maole_004490.T1, Maole_004776.T1, Maole_005120.
Maole_010839.T1, Maole_011302.T1, Maole_011737.T1, Maole_011738.T1, Maole_014148.

Maole_002340.T1, Maole_002341.T1, Maole_002342.T1, Maole_002343.T1, Maole_004829.
Maole_019059.T1

Maole_010105.T1, Maole_017068.T1, Maole_019117.T1, Maole_021175.T1, Maole_021178.

Maole_012034.T1, Maole_012036.T1, Maole_012037.T1, Maole_013805.T1, Maole_013806.
Maole_012034.T1, Maole_012036.T1, Maole_012037.T1, Maole_013805.T1, Maole_013806.

Maole_012034.T1, Maole_012036.T1, Maole_012037.T1, Maole_013805.T1, Maole_013806.
Maole_023742.T1
Maole_020951.T1, Maole_020952.T1, Maole_023348.T1, Maole_023352.T1, Maole_023550.

Maole_004207.T1, Maole_004208.T1, Maole_005495.T1, Maole_006013.T1, Maole_006014.

Maole_024019.T1, Maole_024051.T1, Maole_024069.T1, Maole_024098.T1
Maole_020459.T1
Maole_004490.T1, Maole_004776.T1, Maole_005269.T1, Maole_005713.T1, Maole_007054.

Maole_019618.T1
Maole_019618.T1

Maole_003004.T1, Maole_003005.T1, Maole_003050.T1, Maole_003774.T1, Maole_003782.

Maole_002321.T1, Maole_002322.T1, Maole_002329.T1, Maole_002335.T1, Maole_002340.
Maole_002321.T1, Maole_002322.T1, Maole_002329.T1, Maole_002335.T1, Maole_002340.
Maole_020848.T1, Maole_020850.T1, Maole_022017.T1, Maole_022019.T1, Maole_022020.
Maole_023342.T1
Maole_017521.T1

Maole_005694.T1, Maole_005695.T1, Maole_005698.T1, Maole_005700.T1, Maole_005702.

Maole_017068.T1, Maole_017439.T1, Maole_017441.T1, Maole_020579.T1, Maole_023241.
Maole_023489.T1

Maole_003001.T1, Maole_003161.T1, Maole_003786.T1, Maole_003790.T1, Maole_003791.
Maole_016085.T1, Maole_021078.T1, Maole_021081.T1, Maole_021238.T1, Maole_021896.
Maole_023059.T1, Maole_023061.T1, Maole_023063.T1, Maole_023064.T1, Maole_023690.
Maole_010276.T1, Maole_015424.T1, Maole_015426.T1, Maole_015427.T1, Maole_017521.

Maole_022128.T1, Maole_022130.T1, Maole_022264.T1, Maole_022954.T1, Maole_023795.

Maole_008161.T1, Maole_008476.T1, Maole_011566.T1, Maole_014681.T1, Maole_014682.

Maole_000328.T1, Maole_000379.T1, Maole_000400.T1, Maole_000418.T1, Maole_000432.
Maole_000270.T1, Maole_000379.T1, Maole_000381.T1, Maole_000390.T1, Maole_000400.
Maole_000379.T1, Maole_000400.T1, Maole_000418.T1, Maole_000432.T1, Maole_000465.
Maole_000519.T1, Maole_000690.T1, Maole_000698.T1, Maole_001017.T1, Maole_001097.
Maole_000390.T1, Maole_000416.T1, Maole_000455.T1, Maole_000473.T1, Maole_000490.
Maole_001017.T1, Maole_001097.T1, Maole_001301.T1, Maole_001375.T1, Maole_001561.
Maole_001097.T1, Maole_001301.T1, Maole_001375.T1, Maole_001631.T1, Maole_001757.
Maole_001097.T1, Maole_001375.T1, Maole_001631.T1, Maole_001757.T1, Maole_001879.
Maole_001097.T1, Maole_001301.T1, Maole_001375.T1, Maole_001631.T1, Maole_001757.
Maole_001097.T1, Maole_001301.T1, Maole_001375.T1, Maole_001631.T1, Maole_001757.
Maole_000893.T1, Maole_001097.T1, Maole_001301.T1, Maole_001375.T1, Maole_001631.
Maole_000644.T1, Maole_000690.T1, Maole_000698.T1, Maole_000856.T1, Maole_000864.
Maole_000690.T1, Maole_000698.T1, Maole_000893.T1, Maole_001017.T1, Maole_001097.
Maole_001106.T1, Maole_001209.T1, Maole_001210.T1, Maole_001703.T1, Maole_002406.
Maole_005388.T1, Maole_005935.T1, Maole_006992.T1, Maole_007322.T1, Maole_007323.
Maole_003203.T1, Maole_003989.T1, Maole_004301.T1, Maole_004916.T1, Maole_004918.
Maole_002782.T1, Maole_004457.T1, Maole_005101.T1, Maole_005388.T1, Maole_005505.
Maole_001021.T1, Maole_001022.T1, Maole_001037.T1, Maole_001096.T1, Maole_001108.
Maole_001237.T1, Maole_001467.T1, Maole_001469.T1, Maole_001516.T1, Maole_001616.
Maole_007382.T1, Maole_007383.T1, Maole_007979.T1, Maole_007982.T1, Maole_008636.
Maole_001566.T1, Maole_001609.T1, Maole_002136.T1, Maole_002556.T1, Maole_002736.
Maole_001931.T1, Maole_001986.T1, Maole_001987.T1, Maole_002202.T1, Maole_002556.
Maole_001512.T1, Maole_001514.T1, Maole_001566.T1, Maole_001578.T1, Maole_001609.
Maole_008596.T1, Maole_009067.T1, Maole_009364.T1, Maole_009509.T1, Maole_009644.
Maole_002950.T1, Maole_002955.T1, Maole_003560.T1, Maole_003905.T1, Maole_004246.
Maole_000921.T1, Maole_000922.T1, Maole_001096.T1, Maole_001108.T1, Maole_001137.
Maole_001987.T1, Maole_002264.T1, Maole_002401.T1, Maole_002591.T1, Maole_002750.
Maole_003813.T1, Maole_004304.T1, Maole_005344.T1, Maole_005388.T1, Maole_005418.
Maole_000870.T1, Maole_000934.T1, Maole_001106.T1, Maole_001123.T1, Maole_001124.
Maole_001971.T1, Maole_002136.T1, Maole_002693.T1, Maole_002736.T1, Maole_002854.
Maole_002136.T1, Maole_002235.T1, Maole_002239.T1, Maole_002406.T1, Maole_002556.
Maole_001422.T1, Maole_001879.T1, Maole_001986.T1, Maole_001987.T1, Maole_002191.
Maole_011401.T1, Maole_012520.T1, Maole_012595.T1, Maole_012596.T1, Maole_012598.
Maole_010114.T1, Maole_010115.T1, Maole_010254.T1, Maole_010356.T1, Maole_010359.
Maole_007633.T1, Maole_008020.T1, Maole_009248.T1, Maole_009294.T1, Maole_009817.
Maole_003232.T1, Maole_003905.T1, Maole_003953.T1, Maole_004189.T1, Maole_004230.
Maole_011111.T1, Maole_011128.T1, Maole_011159.T1, Maole_011407.T1, Maole_013561.
Maole_006695.T1, Maole_006799.T1, Maole_007830.T1, Maole_007939.T1, Maole_008122.
Maole_014059.T1, Maole_017085.T1, Maole_017086.T1, Maole_017087.T1, Maole_017092.
Maole_001469.T1, Maole_001878.T1, Maole_001935.T1, Maole_003007.T1, Maole_004003.
Maole_014269.T1, Maole_014270.T1, Maole_014272.T1, Maole_014274.T1, Maole_014275.
Maole_010114.T1, Maole_010115.T1, Maole_010254.T1, Maole_010356.T1, Maole_010359.

Maole_003905.T1, Maole_004189.T1, Maole_004230.T1, Maole_004398.T1, Maole_004564.
Maole_006885.T1, Maole_009320.T1, Maole_009505.T1, Maole_010110.T1, Maole_010336.
Maole_002777.T1, Maole_002782.T1, Maole_004939.T1, Maole_005023.T1, Maole_006040.
Maole_001125.T1, Maole_001209.T1, Maole_001210.T1, Maole_001422.T1, Maole_001481.
Maole_007973.T1, Maole_009509.T1, Maole_011670.T1, Maole_011691.T1, Maole_011692.
Maole_009644.T1, Maole_011710.T1, Maole_012158.T1, Maole_012507.T1, Maole_013130.
Maole_011110.T1, Maole_011111.T1, Maole_011159.T1, Maole_011357.T1, Maole_011358.
Maole_014272.T1, Maole_014274.T1, Maole_014275.T1, Maole_015116.T1, Maole_021416.
Maole_000687.T1, Maole_000724.T1, Maole_000839.T1, Maole_000844.T1, Maole_000870.
Maole_002862.T1, Maole_003007.T1, Maole_003601.T1, Maole_003602.T1, Maole_003604.
Maole_003227.T1, Maole_003283.T1, Maole_003557.T1, Maole_003560.T1, Maole_004251.
Maole_010110.T1, Maole_010336.T1, Maole_011519.T1, Maole_015116.T1, Maole_018059.
Maole_002505.T1, Maole_003065.T1, Maole_004548.T1, Maole_004862.T1, Maole_005101.
Maole_009317.T1, Maole_011589.T1, Maole_011885.T1, Maole_014516.T1, Maole_014730.
Maole_011358.T1, Maole_011401.T1, Maole_013561.T1, Maole_013780.T1, Maole_014744.
Maole_016463.T1, Maole_016467.T1, Maole_016855.T1, Maole_017397.T1, Maole_017398.
Maole_003543.T1, Maole_003978.T1, Maole_004980.T1, Maole_004982.T1, Maole_005385.
Maole_006885.T1, Maole_007101.T1, Maole_007703.T1, Maole_008432.T1, Maole_009317.
Maole_010479.T1, Maole_011181.T1, Maole_012665.T1, Maole_014746.T1, Maole_016665.
Maole_004139.T1, Maole_004215.T1, Maole_004337.T1, Maole_005005.T1, Maole_005372.
Maole_010083.T1, Maole_011832.T1, Maole_012061.T1, Maole_016177.T1, Maole_016571.
Maole_003989.T1, Maole_004073.T1, Maole_004862.T1, Maole_004942.T1, Maole_005018.
Maole_014488.T1, Maole_017155.T1, Maole_018592.T1, Maole_018597.T1, Maole_018598.
Maole_004072.T1, Maole_004407.T1, Maole_004696.T1, Maole_004714.T1, Maole_004972.
Maole_007939.T1, Maole_009029.T1, Maole_009102.T1, Maole_009610.T1, Maole_009888.
Maole_001467.T1, Maole_001610.T1, Maole_001986.T1, Maole_001987.T1, Maole_002003.
Maole_004430.T1, Maole_004695.T1, Maole_004819.T1, Maole_005152.T1, Maole_006581.
Maole_006445.T1, Maole_008020.T1, Maole_009817.T1, Maole_015027.T1, Maole_016461.
Maole_007631.T1, Maole_008706.T1, Maole_008707.T1, Maole_010934.T1, Maole_011523.
Maole_012507.T1, Maole_013112.T1, Maole_014602.T1, Maole_014641.T1, Maole_016011.
Maole_009063.T1, Maole_009534.T1, Maole_009883.T1, Maole_009884.T1, Maole_009885.
Maole_014602.T1, Maole_014641.T1, Maole_015873.T1, Maole_016011.T1, Maole_017094.
Maole_001854.T1, Maole_002003.T1, Maole_002028.T1, Maole_002241.T1, Maole_002433.
Maole_022733.T1, Maole_022737.T1
Maole_022733.T1, Maole_022737.T1
Maole_002747.T1, Maole_002874.T1, Maole_003283.T1, Maole_004047.T1, Maole_004885.
Maole_005534.T1, Maole_005535.T1, Maole_006100.T1, Maole_006269.T1, Maole_008292.
Maole_006799.T1, Maole_006933.T1, Maole_007108.T1, Maole_008706.T1, Maole_008873.
Maole_006485.T1, Maole_007024.T1, Maole_008505.T1, Maole_008512.T1, Maole_008790.
Maole_001404.T1, Maole_002239.T1, Maole_002406.T1, Maole_002736.T1, Maole_002782.
Maole_016278.T1, Maole_017277.T1, Maole_017648.T1, Maole_017866.T1, Maole_017867.
Maole_004457.T1, Maole_004880.T1, Maole_005622.T1, Maole_005625.T1, Maole_005626.
Maole_006799.T1, Maole_006933.T1, Maole_011813.T1, Maole_014744.T1, Maole_016169.
Maole_001609.T1, Maole_001700.T1, Maole_002029.T1, Maole_002146.T1, Maole_002240.
Maole_003148.T1, Maole_003152.T1, Maole_003310.T1, Maole_005237.T1, Maole_005998.
Maole_003813.T1, Maole_003920.T1, Maole_004100.T1, Maole_004101.T1, Maole_004104.
Maole_006799.T1, Maole_006933.T1, Maole_008706.T1, Maole_011813.T1, Maole_012819.
Maole_007898.T1, Maole_009038.T1, Maole_009432.T1, Maole_013413.T1, Maole_015214.

Maole_006688.T1, Maole_007322.T1, Maole_007323.T1, Maole_007590.T1, Maole_009505.
Maole_008868.T1, Maole_009099.T1, Maole_009320.T1, Maole_010110.T1, Maole_010293.
Maole_019968.T1, Maole_024010.T1
Maole_016467.T1, Maole_016855.T1, Maole_017397.T1, Maole_017398.T1
Maole_022737.T1
Maole_022733.T1, Maole_022737.T1
Maole_016343.T1, Maole_016345.T1, Maole_020028.T1, Maole_020657.T1
Maole_010257.T1, Maole_013332.T1, Maole_013450.T1, Maole_013694.T1, Maole_014796.
Maole_001616.T1, Maole_002003.T1, Maole_002913.T1, Maole_003259.T1, Maole_003261.
Maole_011764.T1, Maole_014580.T1, Maole_017220.T1, Maole_018579.T1, Maole_019075.
Maole_003754.T1, Maole_006095.T1, Maole_007108.T1, Maole_007303.T1, Maole_007320.
Maole_006311.T1, Maole_006471.T1, Maole_012682.T1, Maole_014919.T1, Maole_016382.
Maole_022842.T1, Maole_024010.T1
Maole_022720.T1, Maole_023419.T1, Maole_023466.T1
Maole_016696.T1, Maole_021905.T1, Maole_022760.T1, Maole_023851.T1
Maole_006351.T1, Maole_006386.T1, Maole_006581.T1, Maole_006583.T1, Maole_006896.
Maole_005902.T1, Maole_006203.T1, Maole_007236.T1, Maole_008020.T1, Maole_009064.
Maole_004136.T1, Maole_004156.T1, Maole_004334.T1, Maole_004457.T1, Maole_004598.
Maole_022733.T1, Maole_022737.T1
Maole_016115.T1
Maole_007939.T1, Maole_008831.T1, Maole_009102.T1, Maole_009610.T1, Maole_009888.
Maole_020423.T1, Maole_022732.T1, Maole_022733.T1, Maole_022737.T1
Maole_001485.T1, Maole_001538.T1, Maole_001608.T1, Maole_001616.T1, Maole_002028.
Maole_022737.T1
Maole_014744.T1, Maole_014867.T1, Maole_016169.T1, Maole_017309.T1, Maole_019453.
Maole_017719.T1, Maole_020605.T1, Maole_020610.T1, Maole_020826.T1, Maole_021993.
Maole_011873.T1, Maole_012405.T1, Maole_015423.T1, Maole_017086.T1, Maole_017734.
Maole_006682.T1, Maole_007455.T1, Maole_007749.T1, Maole_008178.T1, Maole_008703.
Maole_005432.T1, Maole_005520.T1, Maole_005818.T1, Maole_006885.T1, Maole_008387.
Maole_012665.T1, Maole_014746.T1, Maole_016953.T1, Maole_019070.T1, Maole_019929.
Maole_004972.T1, Maole_005408.T1, Maole_005414.T1, Maole_006095.T1, Maole_006885.
Maole_005453.T1, Maole_006574.T1, Maole_007285.T1, Maole_007898.T1, Maole_014858.
Maole_020657.T1
Maole_004156.T1, Maole_004881.T1, Maole_005236.T1, Maole_005385.T1, Maole_005935.
Maole_007101.T1, Maole_007504.T1, Maole_009096.T1, Maole_009099.T1, Maole_009289.
Maole_017313.T1, Maole_022720.T1
Maole_003596.T1, Maole_003994.T1, Maole_004548.T1, Maole_005414.T1, Maole_005818.
Maole_004444.T1, Maole_004445.T1, Maole_004880.T1, Maole_005625.T1, Maole_005626.
Maole_001931.T1, Maole_001971.T1, Maole_001986.T1, Maole_001987.T1, Maole_002288.
Maole_018034.T1, Maole_018035.T1, Maole_019820.T1, Maole_023245.T1
Maole_006681.T1, Maole_006682.T1, Maole_007455.T1, Maole_007749.T1, Maole_008178.
Maole_005335.T1, Maole_005625.T1, Maole_005626.T1, Maole_005709.T1, Maole_007703.
Maole_007590.T1, Maole_009505.T1, Maole_011163.T1, Maole_013413.T1, Maole_013870.

Maole_003084.T1, Maole_003989.T1, Maole_004003.T1, Maole_004942.T1, Maole_007843.

Maole_015044.T1, Maole_019510.T1, Maole_019512.T1, Maole_021285.T1

Maole_014161.T1, Maole_016444.T1, Maole_016890.T1, Maole_018026.T1, Maole_018075.

Maole_009968.T1, Maole_010427.T1, Maole_010920.T1, Maole_011595.T1, Maole_011678.
Maole_009968.T1, Maole_010427.T1, Maole_010920.T1, Maole_011595.T1, Maole_011678.
Maole_009968.T1, Maole_010427.T1, Maole_010920.T1, Maole_011595.T1, Maole_011678.

Maole_002680.T1, Maole_002761.T1, Maole_003030.T1, Maole_003232.T1, Maole_003245.
Maole_003604.T1, Maole_003605.T1, Maole_003607.T1, Maole_006469.T1, Maole_007425.
Maole_017223.T1, Maole_018863.T1, Maole_018864.T1, Maole_019391.T1, Maole_019762.
Maole_002899.T1, Maole_003249.T1, Maole_003848.T1, Maole_004136.T1, Maole_004251.

Maole_003607.T1, Maole_010554.T1, Maole_011514.T1, Maole_011533.T1, Maole_011536.
Maole_023771.T1
Maole_012342.T1, Maole_015677.T1, Maole_016473.T1
Maole_011207.T1, Maole_011872.T1, Maole_013830.T1, Maole_015154.T1, Maole_020869.
Maole_006315.T1, Maole_010425.T1, Maole_013359.T1, Maole_014142.T1, Maole_014640.
Maole_014272.T1, Maole_014274.T1, Maole_014275.T1
Maole_009320.T1, Maole_012093.T1, Maole_012448.T1, Maole_014265.T1, Maole_014267.
Maole_004771.T1, Maole_004939.T1, Maole_005807.T1, Maole_005983.T1, Maole_006211.
Maole_006695.T1, Maole_007939.T1, Maole_009102.T1, Maole_013780.T1, Maole_014397.
Maole_003607.T1, Maole_006469.T1, Maole_010554.T1, Maole_011514.T1, Maole_011533.
Maole_010427.T1, Maole_010920.T1, Maole_011595.T1, Maole_011678.T1, Maole_012299.
Maole_011764.T1, Maole_014580.T1, Maole_017220.T1, Maole_018579.T1, Maole_023840.
Maole_014397.T1, Maole_016665.T1
Maole_009038.T1, Maole_010336.T1, Maole_010383.T1, Maole_013689.T1, Maole_014174.
Maole_023561.T1, Maole_023562.T1, Maole_023563.T1, Maole_023564.T1, Maole_023753.

Maole_011207.T1, Maole_011872.T1, Maole_013830.T1, Maole_015154.T1, Maole_020869.
Maole_004787.T1, Maole_005372.T1, Maole_005908.T1, Maole_006257.T1, Maole_006337.
Maole_019075.T1, Maole_020187.T1, Maole_020738.T1, Maole_021717.T1
Maole_004896.T1, Maole_006485.T1, Maole_008505.T1, Maole_008790.T1, Maole_009025.

Maole_018524.T1, Maole_022120.T1, Maole_023357.T1

Maole_013030.T1, Maole_013130.T1, Maole_019253.T1, Maole_019256.T1, Maole_019257.

Maole_003607.T1, Maole_006469.T1, Maole_010554.T1, Maole_011514.T1, Maole_011533.
Maole_003607.T1, Maole_006469.T1, Maole_010554.T1, Maole_011514.T1, Maole_011533.

Maole_017309.T1, Maole_019453.T1, Maole_021185.T1, Maole_021573.T1, Maole_023076.
Maole_021185.T1, Maole_023076.T1
Maole_003226.T1, Maole_003813.T1, Maole_004164.T1, Maole_004363.T1, Maole_004409.
Maole_009968.T1, Maole_010427.T1, Maole_010920.T1, Maole_011595.T1, Maole_011678.

Maole_022398.T1
Maole_009096.T1, Maole_009099.T1, Maole_009289.T1, Maole_010021.T1, Maole_010661.

Maole_019310.T1, Maole_022414.T1
Maole_019320.T1

Maole_010293.T1, Maole_010920.T1, Maole_011026.T1, Maole_011930.T1, Maole_013856.
Maole_006235.T1, Maole_006236.T1, Maole_007108.T1, Maole_007285.T1, Maole_007631.
Maole_009303.T1, Maole_009636.T1, Maole_011670.T1, Maole_011953.T1, Maole_012673.

Maole_002408.T1, Maole_002433.T1, Maole_002779.T1, Maole_003203.T1, Maole_003560.

Maole_021894.T1
Maole_022090.T1, Maole_022471.T1, Maole_022820.T1
Maole_003601.T1, Maole_003602.T1, Maole_003604.T1, Maole_003605.T1, Maole_003607.
Maole_018533.T1, Maole_019177.T1, Maole_019303.T1, Maole_021844.T1, Maole_022842.
Maole_023523.T1
Maole_005024.T1, Maole_005664.T1, Maole_006199.T1, Maole_006311.T1, Maole_008382.
Maole_005276.T1, Maole_006315.T1, Maole_006559.T1, Maole_009043.T1, Maole_009242.

Maole_013950.T1, Maole_016971.T1, Maole_017331.T1, Maole_018459.T1, Maole_021727.

Maole_005344.T1, Maole_005421.T1, Maole_006068.T1, Maole_006095.T1, Maole_006199.
Maole_011589.T1, Maole_012467.T1, Maole_014640.T1, Maole_015766.T1, Maole_017911.
Maole_003989.T1, Maole_004942.T1, Maole_006614.T1, Maole_007342.T1, Maole_009968.
Maole_023776.T1, Maole_024056.T1, Maole_024121.T1

Maole_024085.T1

Maole_021718.T1
Maole_000738.T1, Maole_000870.T1, Maole_000893.T1, Maole_000945.T1, Maole_001017.
Maole_008706.T1, Maole_009248.T1, Maole_011288.T1, Maole_011813.T1, Maole_012819.

Maole_023771.T1
Maole_023771.T1

Maole_010110.T1, Maole_011930.T1, Maole_020499.T1, Maole_023564.T1, Maole_023753.
Maole_016855.T1, Maole_017228.T1, Maole_017245.T1, Maole_017398.T1, Maole_022347.

Maole_015738.T1, Maole_024056.T1

Maole_004251.T1, Maole_004697.T1, Maole_005276.T1, Maole_009619.T1, Maole_009962.
Maole_010601.T1, Maole_012144.T1, Maole_012145.T1, Maole_012448.T1, Maole_013030.
Maole_020819.T1, Maole_022090.T1, Maole_022471.T1
Maole_006896.T1, Maole_007342.T1, Maole_009465.T1, Maole_010599.T1, Maole_011873.

Maole_006356.T1, Maole_006539.T1, Maole_006670.T1, Maole_006779.T1, Maole_007057.
Maole_015759.T1, Maole_016981.T1

Maole_004714.T1, Maole_005198.T1, Maole_006702.T1, Maole_008292.T1, Maole_008734.

Maole_005012.T1, Maole_006539.T1, Maole_006885.T1, Maole_009038.T1, Maole_010110.

Maole_009921.T1, Maole_010760.T1, Maole_010762.T1, Maole_010763.T1, Maole_013630.
Maole_023357.T1
Maole_002102.T1, Maole_002191.T1, Maole_002779.T1, Maole_003283.T1, Maole_003994.
Maole_014199.T1, Maole_016541.T1, Maole_016542.T1, Maole_021285.T1
Maole_010383.T1, Maole_011885.T1, Maole_013571.T1, Maole_013689.T1, Maole_014174.

Maole_009817.T1, Maole_014142.T1, Maole_015343.T1, Maole_016401.T1, Maole_017460.

Maole_005071.T1, Maole_005514.T1, Maole_006126.T1, Maole_006766.T1, Maole_006915.
Maole_015202.T1, Maole_017911.T1, Maole_018460.T1, Maole_018461.T1, Maole_018463.

Maole_002411.T1, Maole_002634.T1, Maole_002707.T1, Maole_003085.T1, Maole_004129.
Maole_018035.T1, Maole_019820.T1, Maole_023245.T1
Maole_015061.T1, Maole_016444.T1, Maole_017476.T1, Maole_018075.T1, Maole_019427.
Maole_020919.T1

Maole_006992.T1, Maole_007101.T1, Maole_007703.T1, Maole_007827.T1, Maole_007834.
Maole_003607.T1, Maole_005232.T1, Maole_006469.T1, Maole_007394.T1, Maole_010554.

Maole_007894.T1, Maole_008023.T1, Maole_008615.T1, Maole_009890.T1, Maole_009921.

Maole_016525.T1, Maole_016953.T1, Maole_016978.T1, Maole_018651.T1, Maole_020238.

Maole_009289.T1, Maole_010021.T1, Maole_010760.T1, Maole_010762.T1, Maole_011813.
Maole_015932.T1, Maole_017222.T1
Maole_017746.T1, Maole_023332.T1, Maole_023688.T1

Maole_011181.T1, Maole_012665.T1, Maole_014746.T1, Maole_019070.T1, Maole_020238.

Maole_008242.T1, Maole_010472.T1, Maole_013157.T1, Maole_013335.T1, Maole_014435.

Maole_020187.T1, Maole_020738.T1, Maole_023468.T1

Maole_015873.T1, Maole_015932.T1, Maole_016612.T1, Maole_016977.T1, Maole_016978.
Maole_020499.T1, Maole_023564.T1, Maole_023753.T1
Maole_008305.T1, Maole_010389.T1, Maole_011318.T1, Maole_011589.T1, Maole_012819.

Maole_018929.T1, Maole_018931.T1, Maole_018932.T1, Maole_023409.T1
Maole_023776.T1, Maole_024056.T1, Maole_024121.T1

Maole_002113.T1, Maole_002588.T1, Maole_002693.T1, Maole_003084.T1, Maole_003203.

Maole_013596.T1, Maole_015254.T1
Maole_001534.T1, Maole_001535.T1, Maole_001551.T1, Maole_001553.T1, Maole_001578.

Maole_008703.T1, Maole_009121.T1, Maole_009522.T1, Maole_012406.T1, Maole_012931.

Maole_020187.T1, Maole_020738.T1, Maole_023468.T1

Maole_006559.T1, Maole_008243.T1, Maole_009027.T1, Maole_009473.T1, Maole_011020.

Maole_016155.T1, Maole_017237.T1, Maole_017713.T1, Maole_023761.T1

Maole_012767.T1, Maole_014546.T1, Maole_017076.T1, Maole_018279.T1, Maole_018847.
Maole_006695.T1, Maole_007148.T1, Maole_009096.T1, Maole_014397.T1, Maole_014516.

Maole_008489.T1, Maole_009064.T1, Maole_010337.T1, Maole_012649.T1, Maole_013127.
Maole_017900.T1, Maole_018614.T1

Maole_003056.T1, Maole_003100.T1, Maole_003227.T1, Maole_004231.T1, Maole_004819.
Maole_018847.T1, Maole_018849.T1, Maole_019149.T1, Maole_020066.T1, Maole_020616.

Maole_004673.T1, Maole_004704.T1, Maole_004750.T1, Maole_005930.T1, Maole_006521.
Maole_018100.T1, Maole_018307.T1, Maole_018627.T1
Maole_018533.T1, Maole_020438.T1
Maole_020436.T1
Maole_013596.T1, Maole_015254.T1

Maole_012819.T1, Maole_014744.T1, Maole_016358.T1, Maole_017309.T1, Maole_019075.

Maole_022866.T1

Maole_015202.T1, Maole_016525.T1, Maole_020854.T1, Maole_021425.T1, Maole_023076.

Maole_005654.T1, Maole_005846.T1, Maole_006203.T1, Maole_006257.T1, Maole_007244.

Maole_004156.T1, Maole_004334.T1, Maole_004598.T1, Maole_004750.T1, Maole_004936.

Maole_012360.T1, Maole_013163.T1, Maole_013339.T1, Maole_016890.T1, Maole_019427.

Maole_017220.T1
Maole_016016.T1, Maole_016525.T1, Maole_016775.T1, Maole_018783.T1, Maole_021847.

Maole_017055.T1, Maole_017814.T1, Maole_020993.T1, Maole_022078.T1, Maole_023612.

Maole_003688.T1, Maole_003848.T1, Maole_004246.T1, Maole_004412.T1, Maole_004881.
Maole_009025.T1, Maole_014400.T1, Maole_015027.T1, Maole_016398.T1, Maole_018540.

Maole_018463.T1, Maole_018465.T1, Maole_018928.T1, Maole_018929.T1, Maole_018931.
Maole_020367.T1, Maole_022414.T1
Maole_010599.T1, Maole_011873.T1, Maole_014059.T1, Maole_016473.T1, Maole_016970.

Maole_016473.T1, Maole_017277.T1, Maole_017476.T1, Maole_020187.T1, Maole_020517.

Maole_021530.T1, Maole_021763.T1
Maole_017313.T1, Maole_022720.T1
Maole_011163.T1, Maole_014267.T1, Maole_014978.T1, Maole_020819.T1, Maole_022471.
Maole_012820.T1, Maole_014711.T1, Maole_015214.T1, Maole_017129.T1, Maole_018140.

Maole_004331.T1, Maole_004972.T1, Maole_005102.T1, Maole_005221.T1, Maole_005388.
Maole_005046.T1, Maole_005607.T1, Maole_005675.T1, Maole_006000.T1, Maole_006066.
Maole_016666.T1, Maole_016977.T1, Maole_016978.T1, Maole_019503.T1, Maole_020979.
Maole_002652.T1, Maole_002693.T1, Maole_003496.T1, Maole_003920.T1, Maole_004100.
Maole_005046.T1, Maole_006391.T1, Maole_007656.T1, Maole_008181.T1, Maole_009181.
Maole_022398.T1
Maole_006126.T1, Maole_006773.T1, Maole_006774.T1, Maole_009740.T1, Maole_010670.
Maole_002146.T1, Maole_002235.T1, Maole_002236.T1, Maole_002240.T1, Maole_002241.
Maole_016463.T1, Maole_016467.T1, Maole_016855.T1, Maole_017397.T1, Maole_017398.
Maole_014744.T1, Maole_016768.T1, Maole_017309.T1, Maole_021185.T1, Maole_022100.
Maole_007270.T1, Maole_007272.T1, Maole_008313.T1, Maole_008839.T1, Maole_009100.

Maole_005702.T1, Maole_007182.T1, Maole_007191.T1, Maole_007193.T1, Maole_007194.
Maole_005694.T1, Maole_005695.T1, Maole_005698.T1, Maole_005700.T1, Maole_005702.
Maole_011401.T1, Maole_012520.T1, Maole_012595.T1, Maole_012596.T1, Maole_012598.
Maole_001424.T1, Maole_001461.T1, Maole_001538.T1, Maole_001545.T1, Maole_001931.
Maole_004490.T1, Maole_004776.T1, Maole_005120.T1, Maole_005178.T1, Maole_005179.
Maole_010114.T1, Maole_010115.T1, Maole_010254.T1, Maole_010356.T1, Maole_010359.
Maole_003161.T1, Maole_003875.T1, Maole_004100.T1, Maole_004101.T1, Maole_004104.
Maole_011111.T1, Maole_011159.T1, Maole_011357.T1, Maole_011358.T1, Maole_011401.
Maole_010114.T1, Maole_010115.T1, Maole_010254.T1, Maole_010356.T1, Maole_010359.
Maole_014272.T1, Maole_014274.T1, Maole_014275.T1, Maole_015116.T1, Maole_021416.
Maole_004776.T1, Maole_005713.T1, Maole_007054.T1, Maole_009104.T1, Maole_009186.
Maole_003875.T1, Maole_004490.T1, Maole_004776.T1, Maole_005062.T1, Maole_005120.
Maole_011358.T1, Maole_011401.T1, Maole_013561.T1, Maole_013780.T1, Maole_014744.
Maole_014272.T1, Maole_014274.T1, Maole_014275.T1, Maole_015116.T1, Maole_015873.
Maole_006581.T1, Maole_006583.T1, Maole_007944.T1, Maole_008212.T1, Maole_010105.
Maole_002978.T1, Maole_003161.T1, Maole_003786.T1, Maole_003790.T1, Maole_003791.
Maole_003875.T1, Maole_004490.T1, Maole_004776.T1, Maole_005062.T1, Maole_005120.
Maole_002707.T1, Maole_003197.T1, Maole_004639.T1, Maole_004972.T1, Maole_004980.
Maole_004776.T1, Maole_005713.T1, Maole_007054.T1, Maole_009104.T1, Maole_009186.
Maole_006779.T1, Maole_009317.T1, Maole_009320.T1, Maole_010200.T1, Maole_010336.
Maole_001545.T1, Maole_001931.T1, Maole_004639.T1, Maole_004881.T1, Maole_004972.
Maole_006779.T1, Maole_009317.T1, Maole_009320.T1, Maole_010200.T1, Maole_010336.
Maole_001931.T1, Maole_002591.T1, Maole_002707.T1, Maole_003069.T1, Maole_003305.
Maole_004776.T1, Maole_005713.T1, Maole_007054.T1, Maole_009104.T1, Maole_009186.
Maole_006779.T1, Maole_009317.T1, Maole_009320.T1, Maole_010200.T1, Maole_010336.
Maole_006779.T1, Maole_009317.T1, Maole_009320.T1, Maole_010200.T1, Maole_010336.
Maole_024019.T1, Maole_024051.T1, Maole_024069.T1, Maole_024098.T1
Maole_003782.T1, Maole_003783.T1, Maole_003786.T1, Maole_003790.T1, Maole_003791.
Maole_006779.T1, Maole_009317.T1, Maole_009320.T1, Maole_010200.T1, Maole_010336.
Maole_021175.T1, Maole_021178.T1, Maole_021594.T1
Maole_020579.T1, Maole_021416.T1, Maole_023241.T1, Maole_023259.T1, Maole_023260.
Maole_021178.T1, Maole_021594.T1
Maole_006779.T1, Maole_007366.T1, Maole_009317.T1, Maole_009320.T1, Maole_010200.
Maole_013561.T1, Maole_014744.T1, Maole_017068.T1, Maole_017309.T1, Maole_017673.
Maole_010105.T1, Maole_014265.T1, Maole_014267.T1, Maole_014269.T1, Maole_014270.
Maole_006779.T1, Maole_009317.T1, Maole_009320.T1, Maole_010200.T1, Maole_010336.
Maole_014274.T1, Maole_014275.T1
Maole_005702.T1, Maole_007182.T1, Maole_007191.T1, Maole_007193.T1, Maole_007194.
Maole_021175.T1, Maole_021178.T1, Maole_021594.T1
Maole_014269.T1, Maole_014270.T1, Maole_014272.T1, Maole_014274.T1, Maole_014275.
Maole_021185.T1
Maole_022513.T1

Maole_024019.T1, Maole_024051.T1, Maole_024069.T1, Maole_024098.T1
Maole_024019.T1, Maole_024051.T1, Maole_024069.T1, Maole_024098.T1
Maole_024019.T1, Maole_024051.T1, Maole_024069.T1, Maole_024098.T1

Maole_004776.T1, Maole_005713.T1, Maole_007054.T1, Maole_009104.T1, Maole_009186.
Maole_022513.T1
Maole_005880.T1, Maole_006532.T1, Maole_006581.T1, Maole_006583.T1, Maole_006647.
Maole_007366.T1, Maole_009317.T1, Maole_009320.T1, Maole_010200.T1, Maole_010203.
Maole_021185.T1
Maole_024019.T1, Maole_024051.T1, Maole_024069.T1, Maole_024098.T1
Maole_006703.T1, Maole_010114.T1, Maole_010115.T1, Maole_010254.T1, Maole_010356.

Maole_023742.T1
Maole_021185.T1

Maole_019618.T1
Maole_019618.T1
Maole_020459.T1
Maole_004972.T1, Maole_005250.T1, Maole_007366.T1, Maole_009317.T1, Maole_009320.

Maole_021185.T1

Maole_024121.T1
Maole_024121.T1

Maole_023742.T1
Maole_012789.T1, Maole_012830.T1, Maole_017647.T1, Maole_019130.T1

Maole_021175.T1, Maole_021178.T1, Maole_021594.T1

Maole_010105.T1, Maole_019117.T1, Maole_021175.T1, Maole_021178.T1, Maole_021594.

Maole_003069.T1, Maole_003305.T1, Maole_003945.T1, Maole_005807.T1, Maole_006532.

Maole_002707.T1, Maole_003197.T1, Maole_006647.T1, Maole_007827.T1, Maole_012162.

Maole_023742.T1

Maole_021342.T1, Maole_021594.T1, Maole_022379.T1, Maole_023736.T1, Maole_023737.

Maole_012596.T1, Maole_012598.T1, Maole_014847.T1, Maole_016455.T1, Maole_016662.
Maole_024121.T1
Maole_023742.T1

Maole_010359.T1, Maole_010362.T1, Maole_012789.T1, Maole_012830.T1, Maole_015203.
Maole_003197.T1, Maole_003782.T1, Maole_003783.T1, Maole_003786.T1, Maole_003790.

Maole_003069.T1, Maole_003305.T1, Maole_003945.T1, Maole_004881.T1, Maole_004972.
Maole_013780.T1, Maole_014744.T1, Maole_017309.T1, Maole_017673.T1, Maole_021185.

Maole_012596.T1, Maole_012598.T1, Maole_014847.T1, Maole_016455.T1, Maole_016662.

Maole_019618.T1

Maole_017068.T1

Maole_010356.T1, Maole_010359.T1, Maole_010362.T1, Maole_015873.T1, Maole_016253.

Maole_022511.T1

Maole_023742.T1
Maole_021175.T1, Maole_021178.T1, Maole_021594.T1

Maole_003069.T1, Maole_003197.T1, Maole_003305.T1, Maole_003945.T1, Maole_006532.

Maole_012598.T1, Maole_016455.T1, Maole_020579.T1, Maole_021063.T1, Maole_022503.

Maole_011288.T1, Maole_017673.T1, Maole_022202.T1, Maole_022821.T1

Maole_023742.T1
Maole_002978.T1, Maole_003197.T1, Maole_003786.T1, Maole_003790.T1, Maole_003791.

Maole_022507.T1

Maole_003161.T1, Maole_003786.T1, Maole_003790.T1, Maole_003791.T1, Maole_003793.
Maole_014744.T1, Maole_016768.T1, Maole_017309.T1, Maole_021185.T1, Maole_022100.
Maole_014269.T1, Maole_014270.T1, Maole_014272.T1, Maole_014274.T1, Maole_014275.
Maole_021175.T1, Maole_021178.T1, Maole_021594.T1

le_016089;Maole_016090;Maole_016091

le_006927;Maole_006928;Maole_006929;Maole_006930
le_008914;Maole_008915;Maole_008916
le_012586;Maole_012587;Maole_012588;Maole_012589;Maole_012590;Maole_012591;Maol
le_017982;Maole_017983;Maole_017984;Maole_017985;Maole_017986;Maole_017987
le_017529;Maole_017530;Maole_017531;Maole_017532;Maole_017533

le_019310;Maole_019311;Maole_019312;Maole_019313;Maole_019314
le_017283;Maole_017284;Maole_017285;Maole_017286

le_007262;Maole_007263;Maole_007264;Maole_007265;Maole_007266

le_000821;Maole_000822;Maole_000823;Maole_000824;Maole_000825;Maole_000826;Maol
le_000966;Maole_000967;Maole_000968
le_002128;Maole_002129
le_002178;Maole_002179;Maole_002180;Maole_002181;Maole_002182;Maole_002183;Maol
le_014450;Maole_014451;Maole_014452;Maole_014453;Maole_014454;Maole_014455;Maol

.T1, Maole_006016.T1, Maole_006017.T1, Maole_009664.T1, Maole_009665.T1, Maole_009
.T1, Maole_007195.T1, Maole_007196.T1, Maole_007198.T1, Maole_007199.T1, Maole_007
.T1, Maole_004856.T1, Maole_005499.T1, Maole_007741.T1, Maole_008287.T1, Maole_008
.T1, Maole_006015.T1, Maole_006016.T1, Maole_006017.T1, Maole_008011.T1, Maole_009
.T1, Maole_009647.T1, Maole_010318.T1, Maole_012710.T1, Maole_012810.T1, Maole_012
.T1, Maole_010025.T1, Maole_010318.T1, Maole_011520.T1, Maole_012607.T1, Maole_012
.T1, Maole_006017.T1, Maole_009665.T1, Maole_009666.T1, Maole_009762.T1, Maole_010
.T1, Maole_010649.T1, Maole_012880.T1, Maole_013187.T1, Maole_014526.T1, Maole_015
.T1, Maole_016670.T1, Maole_018871.T1, Maole_018906.T1, Maole_020217.T1, Maole_021
.T1, Maole_021627.T1, Maole_021799.T1, Maole_021801.T1, Maole_022097.T1, Maole_022
.T1, Maole_005948.T1, Maole_006013.T1, Maole_006014.T1, Maole_006015.T1, Maole_006
.T1, Maole_001774.T1, Maole_001995.T1, Maole_002026.T1, Maole_002030.T1, Maole_002
.T1, Maole_023348.T1, Maole_023352.T1, Maole_023550.T1, Maole_024105.T1

.T1, Maole_004175.T1, Maole_004639.T1, Maole_006968.T1, Maole_007741.T1, Maole_008
.T1, Maole_007191.T1, Maole_007193.T1, Maole_007194.T1, Maole_007195.T1, Maole_007
.T1, Maole_006017.T1, Maole_009665.T1, Maole_009666.T1, Maole_010178.T1, Maole_010
.T1, Maole_018090.T1, Maole_020743.T1, Maole_021244.T1, Maole_021627.T1, Maole_021
.T1, Maole_021052.T1, Maole_023348.T1, Maole_023352.T1, Maole_023550.T1, Maole_024
.T1, Maole_017248.T1, Maole_017511.T1, Maole_020144.T1, Maole_020217.T1, Maole_020

.T1, Maole_019870.T1, Maole_021175.T1, Maole_021594.T1, Maole_022795.T1
.T1, Maole_003005.T1, Maole_003050.T1, Maole_003197.T1, Maole_004342.T1, Maole_004

.T1, Maole_007919.T1, Maole_008738.T1, Maole_012519.T1, Maole_012618.T1, Maole_012

.T1, Maole_016630.T1, Maole_016636.T1, Maole_023204.T1, Maole_023205.T1, Maole_023

.T1, Maole_008096.T1, Maole_008097.T1, Maole_008098.T1, Maole_008100.T1, Maole_008
.T1, Maole_005649.T1, Maole_005882.T1, Maole_005883.T1, Maole_005885.T1, Maole_005
.T1, Maole_004776.T1, Maole_005062.T1, Maole_005120.T1, Maole_005178.T1, Maole_005
.T1, Maole_007389.T1, Maole_007560.T1, Maole_007706.T1, Maole_008518.T1, Maole_009

.T1, Maole_003875.T1, Maole_004083.T1, Maole_004084.T1, Maole_004490.T1, Maole_004
.T1, Maole_005171.T1, Maole_005495.T1, Maole_006013.T1, Maole_006014.T1, Maole_006
.T1, Maole_002341.T1, Maole_002342.T1, Maole_002343.T1, Maole_004856.T1, Maole_005
.T1, Maole_009186.T1, Maole_010559.T1, Maole_010560.T1, Maole_010649.T1, Maole_012

.T1, Maole_023574.T1, Maole_023778.T1, Maole_023780.T1, Maole_023782.T1, Maole_023
.T1, Maole_010649.T1, Maole_012880.T1, Maole_013187.T1, Maole_014526.T1, Maole_018

.T1, Maole_006017.T1, Maole_007730.T1, Maole_009664.T1, Maole_009665.T1, Maole_009
.T1, Maole_003793.T1, Maole_003796.T1, Maole_003875.T1, Maole_004490.T1, Maole_004

.T1, Maole_002341.T1, Maole_002342.T1, Maole_002343.T1, Maole_004856.T1, Maole_005
.T1, Maole_024105.T1

.T1, Maole_008767.T1, Maole_008769.T1, Maole_011157.T1, Maole_011402.T1, Maole_012
.T1, Maole_001995.T1, Maole_002030.T1, Maole_002032.T1, Maole_002100.T1, Maole_002

.T1, Maole_007054.T1, Maole_007081.T1, Maole_009104.T1, Maole_009186.T1, Maole_010

.T1, Maole_001984.T1, Maole_002038.T1, Maole_002039.T1, Maole_002229.T1, Maole_002

.T1, Maole_022795.T1

.T1, Maole_005178.T1, Maole_005179.T1, Maole_005180.T1, Maole_005269.T1, Maole_005
.T1, Maole_014799.T1, Maole_016769.T1, Maole_017243.T1, Maole_017626.T1, Maole_017

.T1, Maole_005259.T1, Maole_005840.T1, Maole_005841.T1, Maole_007733.T1, Maole_007

.T1, Maole_021594.T1, Maole_024061.T1

.T1, Maole_013807.T1, Maole_013808.T1, Maole_017285.T1, Maole_024088.T1, Maole_024
.T1, Maole_013807.T1, Maole_013808.T1, Maole_017285.T1, Maole_024088.T1, Maole_024

.T1, Maole_013807.T1, Maole_013808.T1, Maole_017285.T1, Maole_024088.T1, Maole_024

.T1

.T1, Maole_006015.T1, Maole_006016.T1, Maole_006017.T1, Maole_006939.T1, Maole_007

.T1, Maole_008609.T1, Maole_009104.T1, Maole_009186.T1, Maole_010649.T1, Maole_012

.T1, Maole_003783.T1, Maole_003786.T1, Maole_003790.T1, Maole_003791.T1, Maole_003

.T1, Maole_002341.T1, Maole_002342.T1, Maole_002343.T1, Maole_004856.T1, Maole_005
.T1, Maole_002341.T1, Maole_002342.T1, Maole_002343.T1, Maole_004856.T1, Maole_005
.T1

.T1, Maole_006938.T1, Maole_007182.T1, Maole_007191.T1, Maole_007193.T1, Maole_007

.T1, Maole_023344.T1, Maole_023347.T1, Maole_023983.T1, Maole_024051.T1, Maole_024

.T1, Maole_003793.T1, Maole_003796.T1, Maole_003875.T1, Maole_004490.T1, Maole_004
.T1, Maole_021898.T1, Maole_023059.T1, Maole_023061.T1, Maole_023063.T1, Maole_023
.T1, Maole_024007.T1
.T1, Maole_021480.T1

.T1

.T1, Maole_014826.T1, Maole_020576.T1, Maole_021896.T1, Maole_021898.T1, Maole_022

.T1, Maole_000465.T1, Maole_000473.T1, Maole_000497.T1, Maole_000517.T1, Maole_000
.T1, Maole_000418.T1, Maole_000473.T1, Maole_000474.T1, Maole_000497.T1, Maole_000
.T1, Maole_000473.T1, Maole_000518.T1, Maole_000519.T1, Maole_000687.T1, Maole_000
.T1, Maole_001137.T1, Maole_001301.T1, Maole_001375.T1, Maole_001631.T1, Maole_001
.T1, Maole_000517.T1, Maole_000518.T1, Maole_000519.T1, Maole_000636.T1, Maole_000
.T1, Maole_001631.T1, Maole_001703.T1, Maole_001757.T1, Maole_001879.T1, Maole_002
.T1, Maole_001879.T1, Maole_002191.T1, Maole_002288.T1, Maole_002304.T1, Maole_002
.T1, Maole_002191.T1, Maole_002288.T1, Maole_002304.T1, Maole_002326.T1, Maole_002
.T1, Maole_001879.T1, Maole_002191.T1, Maole_002288.T1, Maole_002304.T1, Maole_002
.T1, Maole_001879.T1, Maole_002191.T1, Maole_002288.T1, Maole_002304.T1, Maole_002
.T1, Maole_001757.T1, Maole_001879.T1, Maole_002191.T1, Maole_002288.T1, Maole_002
.T1, Maole_000870.T1, Maole_000886.T1, Maole_000934.T1, Maole_000945.T1, Maole_001
.T1, Maole_001137.T1, Maole_001561.T1, Maole_001703.T1, Maole_001757.T1, Maole_001
.T1, Maole_002704.T1, Maole_002715.T1, Maole_003356.T1, Maole_004167.T1, Maole_004
.T1, Maole_007381.T1, Maole_007383.T1, Maole_007462.T1, Maole_007979.T1, Maole_007
.T1, Maole_004942.T1, Maole_006692.T1, Maole_006693.T1, Maole_006694.T1, Maole_006
.T1, Maole_005612.T1, Maole_006437.T1, Maole_006559.T1, Maole_006766.T1, Maole_006
.T1, Maole_001237.T1, Maole_001501.T1, Maole_001545.T1, Maole_001609.T1, Maole_001
.T1, Maole_001854.T1, Maole_001931.T1, Maole_002003.T1, Maole_002240.T1, Maole_002
.T1, Maole_009003.T1, Maole_009440.T1, Maole_009441.T1, Maole_010148.T1, Maole_011
.T1, Maole_002854.T1, Maole_002913.T1, Maole_003092.T1, Maole_003101.T1, Maole_003
.T1, Maole_002899.T1, Maole_003498.T1, Maole_004704.T1, Maole_004880.T1, Maole_005
.T1, Maole_001854.T1, Maole_002136.T1, Maole_002141.T1, Maole_002689.T1, Maole_002
.T1, Maole_009992.T1, Maole_011128.T1, Maole_011710.T1, Maole_011885.T1, Maole_012
.T1, Maole_004798.T1, Maole_006040.T1, Maole_006885.T1, Maole_007703.T1, Maole_008
.T1, Maole_001227.T1, Maole_001356.T1, Maole_001467.T1, Maole_001566.T1, Maole_001
.T1, Maole_003203.T1, Maole_003498.T1, Maole_003901.T1, Maole_003978.T1, Maole_004
.T1, Maole_005557.T1, Maole_005622.T1, Maole_006080.T1, Maole_006356.T1, Maole_006
.T1, Maole_001125.T1, Maole_001209.T1, Maole_001210.T1, Maole_001404.T1, Maole_001
.T1, Maole_002913.T1, Maole_003092.T1, Maole_003101.T1, Maole_003148.T1, Maole_003
.T1, Maole_002689.T1, Maole_002783.T1, Maole_003100.T1, Maole_003148.T1, Maole_003
.T1, Maole_002288.T1, Maole_002634.T1, Maole_002707.T1, Maole_002761.T1, Maole_002
.T1, Maole_013561.T1, Maole_014847.T1, Maole_015061.T1, Maole_016253.T1, Maole_016
.T1, Maole_010362.T1, Maole_012789.T1, Maole_012830.T1, Maole_017647.T1, Maole_019
.T1, Maole_010035.T1, Maole_011380.T1, Maole_013961.T1, Maole_014142.T1, Maole_014
.T1, Maole_006799.T1, Maole_006933.T1, Maole_008706.T1, Maole_011813.T1, Maole_012
.T1, Maole_014744.T1, Maole_014835.T1, Maole_015354.T1, Maole_017309.T1, Maole_017
.T1, Maole_008318.T1, Maole_008618.T1, Maole_008831.T1, Maole_009610.T1, Maole_009
.T1, Maole_017128.T1, Maole_021494.T1, Maole_022202.T1
.T1, Maole_004136.T1, Maole_004156.T1, Maole_004334.T1, Maole_004598.T1, Maole_004
.T1, Maole_015116.T1, Maole_015873.T1, Maole_019951.T1, Maole_020968.T1, Maole_021
.T1, Maole_010362.T1, Maole_012789.T1, Maole_012830.T1, Maole_017647.T1, Maole_019

.T1, Maole_004862.T1, Maole_004982.T1, Maole_005232.T1, Maole_006085.T1, Maole_006
.T1, Maole_011163.T1, Maole_011519.T1, Maole_015116.T1, Maole_020819.T1, Maole_022
.T1, Maole_006624.T1, Maole_006885.T1, Maole_009303.T1, Maole_009320.T1, Maole_009
.T1, Maole_002239.T1, Maole_002406.T1, Maole_002588.T1, Maole_002634.T1, Maole_002
.T1, Maole_012116.T1, Maole_014466.T1, Maole_014475.T1, Maole_014477.T1, Maole_014
.T1, Maole_013299.T1, Maole_014602.T1, Maole_014641.T1, Maole_016011.T1, Maole_017
.T1, Maole_011401.T1, Maole_013561.T1, Maole_013780.T1, Maole_014744.T1, Maole_015
.T1, Maole_023259.T1, Maole_023260.T1, Maole_023776.T1, Maole_024056.T1, Maole_024
.T1, Maole_000886.T1, Maole_000899.T1, Maole_000905.T1, Maole_001017.T1, Maole_001
.T1, Maole_003605.T1, Maole_003607.T1, Maole_004129.T1, Maole_004301.T1, Maole_004
.T1, Maole_005483.T1, Maole_006036.T1, Maole_006351.T1, Maole_006581.T1, Maole_006
.T1, Maole_022187.T1, Maole_022607.T1, Maole_023032.T1, Maole_023259.T1, Maole_023
.T1, Maole_005102.T1, Maole_005110.T1, Maole_005195.T1, Maole_005823.T1, Maole_006
.T1, Maole_015116.T1, Maole_015735.T1, Maole_015737.T1, Maole_015738.T1, Maole_020
.T1, Maole_016253.T1, Maole_020501.T1, Maole_020502.T1, Maole_022100.T1, Maole_023
.T1
.T1, Maole_005514.T1, Maole_005555.T1, Maole_005595.T1, Maole_006386.T1, Maole_006
.T1, Maole_009320.T1, Maole_010110.T1, Maole_010336.T1, Maole_011519.T1, Maole_012
.T1, Maole_019070.T1, Maole_020238.T1
.T1, Maole_005461.T1, Maole_005483.T1, Maole_005654.T1, Maole_005814.T1, Maole_005
.T1, Maole_016933.T1, Maole_017223.T1, Maole_019391.T1, Maole_019762.T1, Maole_021
.T1, Maole_005555.T1, Maole_006614.T1, Maole_006692.T1, Maole_006693.T1, Maole_006
.T1, Maole_018866.T1, Maole_019404.T1, Maole_019405.T1, Maole_019406.T1, Maole_019
.T1, Maole_005074.T1, Maole_006040.T1, Maole_006885.T1, Maole_007690.T1, Maole_008
.T1, Maole_011345.T1, Maole_011352.T1, Maole_011813.T1, Maole_011975.T1, Maole_012
.T1, Maole_002056.T1, Maole_002433.T1, Maole_002652.T1, Maole_003007.T1, Maole_003
.T1, Maole_006583.T1, Maole_007425.T1, Maole_008596.T1, Maole_009043.T1, Maole_010
.T1, Maole_016463.T1, Maole_016467.T1, Maole_016855.T1, Maole_017397.T1, Maole_017
.T1, Maole_012144.T1, Maole_012145.T1, Maole_013763.T1, Maole_016155.T1, Maole_020
.T1, Maole_017094.T1, Maole_018026.T1, Maole_019951.T1, Maole_020000.T1, Maole_020
.T1, Maole_011321.T1, Maole_011322.T1, Maole_011614.T1, Maole_011616.T1, Maole_011
.T1, Maole_018026.T1, Maole_019951.T1, Maole_020000.T1, Maole_020968.T1, Maole_021
.T1, Maole_002627.T1, Maole_002680.T1, Maole_002689.T1, Maole_003007.T1, Maole_003

.T1, Maole_005119.T1, Maole_005557.T1, Maole_006561.T1, Maole_007046.T1, Maole_007
.T1, Maole_008299.T1, Maole_008305.T1, Maole_009025.T1, Maole_010110.T1, Maole_011
.T1, Maole_011813.T1, Maole_014744.T1, Maole_016169.T1, Maole_017713.T1, Maole_017
.T1, Maole_012531.T1, Maole_012626.T1, Maole_012627.T1, Maole_012678.T1, Maole_013
.T1, Maole_003227.T1, Maole_003232.T1, Maole_004072.T1, Maole_004164.T1, Maole_004
.T1, Maole_017868.T1, Maole_017870.T1, Maole_017871.T1, Maole_017874.T1, Maole_019
.T1, Maole_006651.T1, Maole_006652.T1, Maole_006992.T1, Maole_007170.T1, Maole_007
.T1, Maole_016237.T1, Maole_017713.T1, Maole_018075.T1, Maole_020238.T1, Maole_020
.T1, Maole_002406.T1, Maole_002634.T1, Maole_002826.T1, Maole_003056.T1, Maole_003
.T1, Maole_006467.T1, Maole_007183.T1, Maole_007391.T1, Maole_007855.T1, Maole_008
.T1, Maole_004105.T1, Maole_004982.T1, Maole_005664.T1, Maole_006199.T1, Maole_006
.T1, Maole_014744.T1, Maole_015343.T1, Maole_016169.T1, Maole_017309.T1, Maole_017
.T1, Maole_016032.T1, Maole_016169.T1, Maole_019947.T1, Maole_021441.T1, Maole_021

.T1, Maole_011163.T1, Maole_014267.T1, Maole_019167.T1, Maole_020819.T1, Maole_021
.T1, Maole_010336.T1, Maole_011519.T1, Maole_011589.T1, Maole_013413.T1, Maole_018

.T1, Maole_016271.T1, Maole_017210.T1, Maole_017313.T1, Maole_018443.T1, Maole_019
.T1, Maole_003356.T1, Maole_003625.T1, Maole_004331.T1, Maole_004714.T1, Maole_005
.T1, Maole_021717.T1, Maole_023840.T1
.T1, Maole_007391.T1, Maole_007427.T1, Maole_007964.T1, Maole_009038.T1, Maole_009
.T1, Maole_016806.T1

.T1, Maole_007687.T1, Maole_007749.T1, Maole_007966.T1, Maole_009067.T1, Maole_009
.T1, Maole_009817.T1, Maole_009921.T1, Maole_010337.T1, Maole_014142.T1, Maole_014
.T1, Maole_004656.T1, Maole_004704.T1, Maole_004750.T1, Maole_004936.T1, Maole_006

.T1, Maole_011813.T1, Maole_015626.T1, Maole_017713.T1, Maole_020423.T1, Maole_022

.T1, Maole_002389.T1, Maole_002406.T1, Maole_002680.T1, Maole_002761.T1, Maole_002

.T1, Maole_021185.T1, Maole_021573.T1, Maole_023076.T1, Maole_023466.T1
.T1
.T1, Maole_019231.T1, Maole_019968.T1, Maole_021494.T1, Maole_022202.T1
.T1, Maole_009091.T1, Maole_010452.T1, Maole_018033.T1, Maole_018034.T1, Maole_018
.T1, Maole_009888.T1, Maole_011163.T1, Maole_011813.T1, Maole_012336.T1, Maole_013
.T1, Maole_020238.T1
.T1, Maole_007323.T1, Maole_007703.T1, Maole_007834.T1, Maole_008292.T1, Maole_008
.T1, Maole_017660.T1, Maole_017900.T1, Maole_019947.T1, Maole_021441.T1, Maole_023

.T1, Maole_007843.T1, Maole_008874.T1, Maole_008875.T1, Maole_008946.T1, Maole_009
.T1, Maole_010021.T1, Maole_011406.T1, Maole_015735.T1, Maole_015737.T1, Maole_015

.T1, Maole_007074.T1, Maole_007143.T1, Maole_007145.T1, Maole_007360.T1, Maole_007
.T1, Maole_006068.T1, Maole_006356.T1, Maole_006651.T1, Maole_006652.T1, Maole_007
.T1, Maole_002411.T1, Maole_002779.T1, Maole_003007.T1, Maole_003203.T1, Maole_003

.T1, Maole_008703.T1, Maole_009029.T1, Maole_009091.T1, Maole_010452.T1, Maole_010
.T1, Maole_008182.T1, Maole_008264.T1, Maole_008713.T1, Maole_008961.T1, Maole_009
.T1, Maole_017331.T1, Maole_018459.T1, Maole_019167.T1, Maole_020819.T1, Maole_021

.T1, Maole_008313.T1, Maole_009968.T1, Maole_010427.T1, Maole_010920.T1, Maole_011

.T1, Maole_019951.T1, Maole_020968.T1, Maole_022845.T1

.T1, Maole_012299.T1, Maole_012301.T1, Maole_013121.T1, Maole_013153.T1, Maole_016
.T1, Maole_012299.T1, Maole_012301.T1, Maole_013121.T1, Maole_013153.T1, Maole_016
.T1, Maole_012299.T1, Maole_012301.T1, Maole_013121.T1, Maole_013153.T1, Maole_016

.T1, Maole_004210.T1, Maole_004319.T1, Maole_004626.T1, Maole_005670.T1, Maole_006
.T1, Maole_007749.T1, Maole_009248.T1, Maole_009367.T1, Maole_010554.T1, Maole_011
.T1, Maole_019764.T1, Maole_019766.T1, Maole_019767.T1
.T1, Maole_004457.T1, Maole_004750.T1, Maole_004787.T1, Maole_005046.T1, Maole_005

.T1, Maole_012227.T1, Maole_013045.T1, Maole_016671.T1, Maole_016806.T1, Maole_018

.T1
.T1, Maole_015471.T1, Maole_018948.T1, Maole_019480.T1, Maole_020438.T1, Maole_020

.T1, Maole_014269.T1, Maole_014270.T1, Maole_014272.T1, Maole_014274.T1, Maole_014
.T1, Maole_006444.T1, Maole_006445.T1, Maole_006624.T1, Maole_007894.T1, Maole_008
.T1, Maole_020423.T1, Maole_022732.T1, Maole_022733.T1, Maole_022737.T1, Maole_023
.T1, Maole_011536.T1, Maole_012227.T1, Maole_012853.T1, Maole_013045.T1, Maole_015
.T1, Maole_012301.T1, Maole_013121.T1, Maole_013153.T1, Maole_016696.T1, Maole_021
.T1

.T1, Maole_014466.T1, Maole_014474.T1, Maole_014475.T1, Maole_019406.T1, Maole_019
.T1

.T1
.T1, Maole_006933.T1, Maole_007942.T1, Maole_008143.T1, Maole_008181.T1, Maole_008

.T1, Maole_011051.T1, Maole_012008.T1, Maole_012531.T1, Maole_012626.T1, Maole_012

.T1, Maole_019580.T1, Maole_023299.T1

.T1, Maole_011536.T1, Maole_012227.T1, Maole_012853.T1, Maole_013045.T1, Maole_015
.T1, Maole_011536.T1, Maole_012227.T1, Maole_012853.T1, Maole_013045.T1, Maole_015

.T1

.T1, Maole_005101.T1, Maole_005741.T1, Maole_006417.T1, Maole_006559.T1, Maole_009
.T1, Maole_012299.T1, Maole_012301.T1, Maole_013121.T1, Maole_013153.T1, Maole_016

.T1, Maole_010662.T1, Maole_012819.T1, Maole_014744.T1, Maole_017135.T1, Maole_017

.T1, Maole_016012.T1, Maole_017214.T1, Maole_019075.T1, Maole_019175.T1, Maole_020
.T1, Maole_008706.T1, Maole_008707.T1, Maole_010934.T1, Maole_011523.T1, Maole_012
.T1, Maole_014430.T1, Maole_014477.T1, Maole_014484.T1, Maole_014487.T1, Maole_014

.T1, Maole_004129.T1, Maole_005221.T1, Maole_005607.T1, Maole_005983.T1, Maole_007

.T1, Maole_004714.T1, Maole_006469.T1, Maole_009150.T1, Maole_009337.T1, Maole_010
.T1, Maole_024010.T1

.T1, Maole_009289.T1, Maole_010021.T1, Maole_010293.T1, Maole_010389.T1, Maole_010
.T1, Maole_009289.T1, Maole_010021.T1, Maole_010540.T1, Maole_011406.T1, Maole_012

.T1, Maole_023357.T1

.T1, Maole_007189.T1, Maole_007391.T1, Maole_007427.T1, Maole_007964.T1, Maole_009
.T1, Maole_018391.T1, Maole_019480.T1, Maole_020436.T1, Maole_023406.T1, Maole_023
.T1, Maole_010423.T1, Maole_010427.T1, Maole_010920.T1, Maole_011595.T1, Maole_011

.T1, Maole_001025.T1, Maole_001037.T1, Maole_001091.T1, Maole_001126.T1, Maole_001
.T1, Maole_013413.T1, Maole_013561.T1, Maole_014744.T1, Maole_016169.T1, Maole_016

.T1
.T1, Maole_022350.T1, Maole_022678.T1

.T1, Maole_012626.T1, Maole_012627.T1, Maole_014519.T1, Maole_016167.T1, Maole_016
.T1, Maole_014314.T1, Maole_014685.T1, Maole_015679.T1, Maole_016155.T1, Maole_016

.T1, Maole_014059.T1, Maole_016473.T1, Maole_016686.T1, Maole_017086.T1, Maole_017

.T1, Maole_007108.T1, Maole_007126.T1, Maole_009064.T1, Maole_012375.T1, Maole_013

.T1, Maole_011161.T1, Maole_013130.T1, Maole_013942.T1, Maole_014711.T1, Maole_016

.T1, Maole_012649.T1, Maole_012673.T1, Maole_012805.T1, Maole_013299.T1, Maole_015

.T1, Maole_014142.T1, Maole_017051.T1, Maole_017544.T1, Maole_018651.T1, Maole_022

.T1, Maole_005478.T1, Maole_005818.T1, Maole_006040.T1, Maole_007074.T1, Maole_007

.T1, Maole_014431.T1, Maole_014883.T1, Maole_016337.T1, Maole_017166.T1, Maole_017

.T1, Maole_017992.T1, Maole_020479.T1

.T1, Maole_007590.T1, Maole_007834.T1, Maole_008625.T1, Maole_010832.T1, Maole_011
.T1, Maole_018465.T1, Maole_019627.T1, Maole_021614.T1, Maole_021727.T1, Maole_023

.T1, Maole_004215.T1, Maole_004304.T1, Maole_004457.T1, Maole_004564.T1, Maole_004

.T1, Maole_022482.T1, Maole_023468.T1, Maole_023479.T1

.T1, Maole_009303.T1, Maole_009505.T1, Maole_011885.T1, Maole_011953.T1, Maole_012
.T1, Maole_010914.T1, Maole_011514.T1, Maole_011533.T1, Maole_011536.T1, Maole_012

.T1, Maole_010457.T1, Maole_010943.T1, Maole_010944.T1, Maole_010945.T1, Maole_010

.T1, Maole_020605.T1, Maole_020610.T1, Maole_020826.T1

.T1, Maole_012022.T1, Maole_013630.T1, Maole_016665.T1, Maole_017660.T1, Maole_017

.T1

.T1, Maole_014517.T1, Maole_015032.T1, Maole_015653.T1, Maole_015838.T1, Maole_018

.T1, Maole_017222.T1, Maole_017644.T1, Maole_019939.T1, Maole_019951.T1, Maole_019

.T1, Maole_014580.T1, Maole_017163.T1, Maole_020436.T1, Maole_020438.T1, Maole_021

.T1, Maole_004047.T1, Maole_004862.T1, Maole_004880.T1, Maole_005023.T1, Maole_005

.T1, Maole_001610.T1, Maole_001631.T1, Maole_001730.T1, Maole_001853.T1, Maole_001

.T1, Maole_013184.T1, Maole_015202.T1, Maole_015254.T1, Maole_016686.T1, Maole_016

.T1, Maole_012318.T1, Maole_012865.T1, Maole_013884.T1, Maole_014517.T1, Maole_015

.T1, Maole_019223.T1, Maole_020122.T1
.T1, Maole_015044.T1, Maole_015729.T1, Maole_017644.T1, Maole_019939.T1, Maole_021

.T1, Maole_013357.T1, Maole_016735.T1, Maole_017889.T1, Maole_020092.T1

.T1, Maole_004896.T1, Maole_005944.T1, Maole_006066.T1, Maole_006083.T1, Maole_006
.T1, Maole_022041.T1

.T1, Maole_006574.T1, Maole_007898.T1, Maole_009432.T1, Maole_009928.T1, Maole_010

.T1, Maole_019453.T1, Maole_021573.T1, Maole_021717.T1

.T1

.T1, Maole_007633.T1, Maole_008020.T1, Maole_008873.T1, Maole_009360.T1, Maole_009

.T1, Maole_005237.T1, Maole_005567.T1, Maole_005823.T1, Maole_006068.T1, Maole_006

.T1, Maole_022760.T1

.T1, Maole_023290.T1

.T1

.T1, Maole_004896.T1, Maole_005058.T1, Maole_005059.T1, Maole_005221.T1, Maole_005
.T1, Maole_018541.T1, Maole_018563.T1, Maole_019303.T1, Maole_022244.T1, Maole_022

.T1, Maole_018932.T1, Maole_019031.T1, Maole_019219.T1, Maole_019627.T1, Maole_019

.T1, Maole_017128.T1, Maole_021494.T1, Maole_022202.T1

.T1, Maole_020738.T1, Maole_023468.T1

.T1
.T1

.T1, Maole_005996.T1, Maole_006214.T1, Maole_006670.T1, Maole_006779.T1, Maole_006
.T1, Maole_007035.T1, Maole_007108.T1, Maole_007656.T1, Maole_008181.T1, Maole_008
.T1, Maole_021768.T1, Maole_023496.T1, Maole_023771.T1
.T1, Maole_004101.T1, Maole_004104.T1, Maole_004105.T1, Maole_004111.T1, Maole_004
.T1, Maole_011198.T1, Maole_011709.T1, Maole_015629.T1, Maole_016735.T1, Maole_016

.T1, Maole_012022.T1, Maole_016666.T1, Maole_017719.T1, Maole_019427.T1, Maole_020
.T1, Maole_002406.T1, Maole_003226.T1, Maole_003227.T1, Maole_003315.T1, Maole_003
.T1, Maole_018850.T1, Maole_018852.T1
.T1, Maole_022821.T1, Maole_023076.T1
.T1, Maole_010479.T1, Maole_011181.T1, Maole_011836.T1, Maole_012665.T1, Maole_014

.T1, Maole_007195.T1, Maole_007196.T1, Maole_007198.T1, Maole_007199.T1, Maole_007
.T1, Maole_007182.T1, Maole_007191.T1, Maole_007193.T1, Maole_007194.T1, Maole_007
.T1, Maole_013561.T1, Maole_014847.T1, Maole_016253.T1, Maole_016455.T1, Maole_016
.T1, Maole_001986.T1, Maole_001987.T1, Maole_002245.T1, Maole_002247.T1, Maole_002
.T1, Maole_005180.T1, Maole_005694.T1, Maole_005695.T1, Maole_005698.T1, Maole_005
.T1, Maole_010362.T1, Maole_012789.T1, Maole_012830.T1, Maole_017647.T1, Maole_019
.T1, Maole_004105.T1, Maole_004490.T1, Maole_004776.T1, Maole_004982.T1, Maole_005
.T1, Maole_013561.T1, Maole_013780.T1, Maole_014744.T1, Maole_016253.T1, Maole_016
.T1, Maole_010362.T1, Maole_012789.T1, Maole_012830.T1, Maole_017647.T1, Maole_019
.T1, Maole_023259.T1, Maole_023260.T1, Maole_023776.T1, Maole_024056.T1, Maole_024
.T1, Maole_010649.T1, Maole_012880.T1, Maole_013187.T1, Maole_014526.T1, Maole_018
.T1, Maole_005178.T1, Maole_005179.T1, Maole_005180.T1, Maole_005694.T1, Maole_005
.T1, Maole_016253.T1, Maole_020501.T1, Maole_020502.T1, Maole_022100.T1
.T1, Maole_021416.T1, Maole_023259.T1, Maole_023260.T1, Maole_023776.T1, Maole_024
.T1, Maole_011357.T1, Maole_011358.T1, Maole_013780.T1, Maole_016253.T1, Maole_019
.T1, Maole_003793.T1, Maole_003796.T1, Maole_003875.T1, Maole_004490.T1, Maole_004
.T1, Maole_005178.T1, Maole_005179.T1, Maole_005180.T1, Maole_005694.T1, Maole_005
.T1, Maole_004982.T1, Maole_005013.T1, Maole_005250.T1, Maole_006581.T1, Maole_006
.T1, Maole_010649.T1, Maole_012880.T1, Maole_013187.T1, Maole_014526.T1, Maole_018
.T1, Maole_011524.T1, Maole_011670.T1, Maole_014265.T1, Maole_014267.T1, Maole_014
.T1, Maole_004980.T1, Maole_004982.T1, Maole_005013.T1, Maole_005250.T1, Maole_005
.T1, Maole_011524.T1, Maole_011670.T1, Maole_014265.T1, Maole_014267.T1, Maole_014
.T1, Maole_003596.T1, Maole_003945.T1, Maole_004100.T1, Maole_004101.T1, Maole_004
.T1, Maole_010649.T1, Maole_011288.T1, Maole_012880.T1, Maole_013187.T1, Maole_014
.T1, Maole_011524.T1, Maole_011670.T1, Maole_014265.T1, Maole_014267.T1, Maole_014
.T1, Maole_011524.T1, Maole_011670.T1, Maole_014265.T1, Maole_014267.T1, Maole_014

.T1, Maole_003793.T1, Maole_003796.T1, Maole_004210.T1, Maole_004626.T1, Maole_006
.T1, Maole_011524.T1, Maole_011670.T1, Maole_014265.T1, Maole_014267.T1, Maole_014

.T1, Maole_023344.T1, Maole_023347.T1, Maole_023776.T1, Maole_023983.T1, Maole_024

.T1, Maole_010203.T1, Maole_010336.T1, Maole_011524.T1, Maole_011670.T1, Maole_014
.T1, Maole_019117.T1, Maole_021175.T1, Maole_021178.T1, Maole_021594.T1
.T1, Maole_014272.T1, Maole_014274.T1, Maole_014275.T1, Maole_019117.T1, Maole_021
.T1, Maole_011524.T1, Maole_011670.T1, Maole_014265.T1, Maole_014267.T1, Maole_014

.T1, Maole_007195.T1, Maole_007196.T1, Maole_007198.T1, Maole_007199.T1, Maole_007

.T1

.T1, Maole_010649.T1, Maole_012880.T1, Maole_013187.T1, Maole_014526.T1, Maole_018

.T1, Maole_007944.T1, Maole_008212.T1, Maole_010105.T1, Maole_011357.T1, Maole_011
.T1, Maole_011524.T1, Maole_011670.T1, Maole_014265.T1, Maole_014267.T1, Maole_014

.T1, Maole_010359.T1, Maole_010362.T1, Maole_012789.T1, Maole_012830.T1, Maole_017

.T1, Maole_012089.T1, Maole_013992.T1, Maole_013993.T1, Maole_013994.T1, Maole_014

.T1

.T1, Maole_006581.T1, Maole_006583.T1, Maole_007944.T1, Maole_008212.T1, Maole_010

.T1, Maole_014199.T1, Maole_015936.T1, Maole_016541.T1, Maole_016542.T1, Maole_017

.T1, Maole_023741.T1, Maole_023742.T1

.T1, Maole_017673.T1, Maole_021234.T1

.T1, Maole_017647.T1, Maole_019130.T1, Maole_021341.T1, Maole_021342.T1, Maole_022
.T1, Maole_003791.T1, Maole_003793.T1, Maole_003796.T1, Maole_004210.T1, Maole_009

.T1, Maole_004982.T1, Maole_006169.T1, Maole_006532.T1, Maole_008212.T1, Maole_009
.T1, Maole_022100.T1, Maole_022821.T1

.T1, Maole_017673.T1

.T1, Maole_017647.T1, Maole_019117.T1, Maole_021175.T1, Maole_021178.T1, Maole_021

.T1, Maole_007944.T1, Maole_008212.T1, Maole_009738.T1, Maole_010105.T1, Maole_013

.T1, Maole_022508.T1, Maole_023238.T1, Maole_023239.T1, Maole_023241.T1, Maole_023

.T1, Maole_003793.T1, Maole_003796.T1, Maole_015936.T1, Maole_017068.T1, Maole_019

.T1, Maole_003796.T1, Maole_003875.T1, Maole_004100.T1, Maole_004101.T1, Maole_004
.T1, Maole_022821.T1
.T1, Maole_015116.T1, Maole_017644.T1, Maole_019939.T1, Maole_021416.T1, Maole_023

le_012592;Maole_012593;Maole_012594;Maole_012595

le_000827;Maole_000828;Maole_000829;Maole_000830;Maole_000831;Maole_000832;Maol

le_002184;Maole_002185
le_014456

666.T1, Maole_010178.T1, Maole_010179.T1, Maole_010180.T1, Maole_010181.T1, Maole_
201.T1, Maole_007202.T1, Maole_007203.T1, Maole_007204.T1, Maole_007205.T1, Maole_
288.T1, Maole_009743.T1, Maole_011302.T1, Maole_014799.T1, Maole_016769.T1, Maole_
664.T1, Maole_009665.T1, Maole_009666.T1, Maole_010178.T1, Maole_010179.T1, Maole_
929.T1, Maole_012936.T1, Maole_013143.T1, Maole_013537.T1, Maole_013876.T1, Maole_
936.T1, Maole_013071.T1, Maole_016670.T1, Maole_016782.T1, Maole_018906.T1, Maole_
178.T1, Maole_010179.T1, Maole_010180.T1, Maole_010181.T1, Maole_010531.T1, Maole_
982.T1, Maole_015983.T1, Maole_018119.T1, Maole_018229.T1, Maole_018251.T1, Maole_
244.T1, Maole_021626.T1, Maole_021627.T1, Maole_021897.T1, Maole_022097.T1, Maole_
164.T1, Maole_023127.T1
016.T1, Maole_006017.T1, Maole_008011.T1, Maole_009664.T1, Maole_009665.T1, Maole_
032.T1, Maole_002100.T1, Maole_002435.T1, Maole_002538.T1, Maole_002617.T1, Maole_

287.T1, Maole_008288.T1, Maole_009743.T1, Maole_011302.T1, Maole_014331.T1, Maole_
196.T1, Maole_007198.T1, Maole_007199.T1, Maole_007201.T1, Maole_007202.T1, Maole_
179.T1, Maole_010180.T1, Maole_010181.T1, Maole_010531.T1, Maole_010533.T1, Maole_
799.T1, Maole_021801.T1, Maole_022097.T1, Maole_022164.T1, Maole_023127.T1
105.T1
658.T1, Maole_020874.T1, Maole_021574.T1, Maole_021799.T1, Maole_021801.T1, Maole_

456.T1, Maole_005171.T1, Maole_005495.T1, Maole_005948.T1, Maole_005966.T1, Maole_

902.T1, Maole_014131.T1, Maole_014132.T1, Maole_014133.T1, Maole_014134.T1, Maole_

206.T1, Maole_023208.T1, Maole_023211.T1

101.T1, Maole_008287.T1, Maole_008288.T1, Maole_008738.T1, Maole_011014.T1, Maole_
886.T1, Maole_005890.T1, Maole_005893.T1, Maole_005894.T1, Maole_005895.T1, Maole_
179.T1, Maole_005180.T1, Maole_005266.T1, Maole_005269.T1, Maole_005694.T1, Maole_
182.T1, Maole_009283.T1, Maole_009647.T1, Maole_010002.T1, Maole_010025.T1, Maole_

776.T1, Maole_004968.T1, Maole_004969.T1, Maole_005062.T1, Maole_005120.T1, Maole_
015.T1, Maole_006016.T1, Maole_006017.T1, Maole_006532.T1, Maole_007363.T1, Maole_
259.T1, Maole_007733.T1, Maole_015424.T1, Maole_015426.T1, Maole_015427.T1, Maole_
880.T1, Maole_013187.T1, Maole_013274.T1, Maole_014526.T1, Maole_014799.T1, Maole_

983.T1, Maole_024051.T1, Maole_024069.T1, Maole_024102.T1
119.T1, Maole_018229.T1, Maole_018251.T1, Maole_019211.T1, Maole_019276.T1, Maole_

666.T1, Maole_010178.T1, Maole_010179.T1, Maole_010180.T1, Maole_010181.T1, Maole_
856.T1, Maole_005713.T1, Maole_007054.T1, Maole_007081.T1, Maole_009104.T1, Maole_

259.T1, Maole_007733.T1, Maole_015424.T1, Maole_015426.T1, Maole_015427.T1, Maole_

033.T1, Maole_012034.T1, Maole_012036.T1, Maole_012037.T1, Maole_013805.T1, Maole_
758.T1, Maole_003052.T1, Maole_003069.T1, Maole_003079.T1, Maole_003305.T1, Maole_

649.T1, Maole_012880.T1, Maole_013187.T1, Maole_014526.T1, Maole_015815.T1, Maole_

434.T1, Maole_002435.T1, Maole_002758.T1, Maole_002809.T1, Maole_003004.T1, Maole_

694.T1, Maole_005695.T1, Maole_005698.T1, Maole_005700.T1, Maole_005702.T1, Maole_
627.T1, Maole_017628.T1, Maole_017630.T1, Maole_017632.T1, Maole_018434.T1, Maole_

908.T1, Maole_007909.T1, Maole_009345.T1, Maole_010272.T1, Maole_010273.T1, Maole_

112.T1
112.T1

112.T1

877.T1, Maole_007878.T1, Maole_008537.T1, Maole_008974.T1, Maole_009664.T1, Maole_

880.T1, Maole_013187.T1, Maole_013491.T1, Maole_013493.T1, Maole_014526.T1, Maole_

793.T1, Maole_003796.T1, Maole_005495.T1, Maole_005948.T1, Maole_006013.T1, Maole_

259.T1, Maole_007733.T1, Maole_015424.T1, Maole_015426.T1, Maole_015427.T1, Maole_
259.T1, Maole_007733.T1, Maole_015424.T1, Maole_015426.T1, Maole_015427.T1, Maole_

194.T1, Maole_007195.T1, Maole_007196.T1, Maole_007198.T1, Maole_007199.T1, Maole_

069.T1

776.T1, Maole_005062.T1, Maole_005713.T1, Maole_007054.T1, Maole_007081.T1, Maole_
064.T1, Maole_023690.T1, Maole_024007.T1

877.T1, Maole_023342.T1

518.T1, Maole_000519.T1, Maole_000558.T1, Maole_000672.T1, Maole_000690.T1, Maole_
498.T1, Maole_000517.T1, Maole_000518.T1, Maole_000519.T1, Maole_000672.T1, Maole_
690.T1, Maole_000698.T1, Maole_000747.T1, Maole_000886.T1, Maole_000893.T1, Maole_
703.T1, Maole_001757.T1, Maole_001859.T1, Maole_001879.T1, Maole_002093.T1, Maole_
643.T1, Maole_000644.T1, Maole_000690.T1, Maole_000698.T1, Maole_000738.T1, Maole_
191.T1, Maole_002288.T1, Maole_002304.T1, Maole_002326.T1, Maole_002461.T1, Maole_
326.T1, Maole_002461.T1, Maole_002469.T1, Maole_002652.T1, Maole_002704.T1, Maole_
461.T1, Maole_002469.T1, Maole_002652.T1, Maole_002704.T1, Maole_002715.T1, Maole_
326.T1, Maole_002461.T1, Maole_002469.T1, Maole_002652.T1, Maole_002704.T1, Maole_
326.T1, Maole_002461.T1, Maole_002469.T1, Maole_002652.T1, Maole_002704.T1, Maole_
304.T1, Maole_002326.T1, Maole_002461.T1, Maole_002469.T1, Maole_002652.T1, Maole_
019.T1, Maole_001020.T1, Maole_001021.T1, Maole_001022.T1, Maole_001037.T1, Maole_
859.T1, Maole_001879.T1, Maole_002093.T1, Maole_002102.T1, Maole_002288.T1, Maole_
238.T1, Maole_004432.T1, Maole_005896.T1, Maole_005897.T1, Maole_005898.T1, Maole_
982.T1, Maole_008636.T1, Maole_009003.T1, Maole_009364.T1, Maole_009440.T1, Maole_
695.T1, Maole_006799.T1, Maole_007939.T1, Maole_008831.T1, Maole_008874.T1, Maole_
885.T1, Maole_007422.T1, Maole_007590.T1, Maole_007602.T1, Maole_007834.T1, Maole_
854.T1, Maole_001931.T1, Maole_002012.T1, Maole_002239.T1, Maole_002264.T1, Maole_
510.T1, Maole_002591.T1, Maole_002652.T1, Maole_002855.T1, Maole_003084.T1, Maole_
670.T1, Maole_012114.T1, Maole_012116.T1, Maole_014466.T1, Maole_014469.T1, Maole_
148.T1, Maole_003152.T1, Maole_003315.T1, Maole_003778.T1, Maole_003953.T1, Maole_
633.T1, Maole_006480.T1, Maole_006693.T1, Maole_006799.T1, Maole_007024.T1, Maole_
736.T1, Maole_002747.T1, Maole_002854.T1, Maole_002913.T1, Maole_003092.T1, Maole_
158.T1, Maole_012405.T1, Maole_012507.T1, Maole_013299.T1, Maole_013579.T1, Maole_
182.T1, Maole_009099.T1, Maole_009320.T1, Maole_010110.T1, Maole_010336.T1, Maole_
609.T1, Maole_001639.T1, Maole_001931.T1, Maole_001971.T1, Maole_002136.T1, Maole_
188.T1, Maole_004189.T1, Maole_004275.T1, Maole_005018.T1, Maole_005555.T1, Maole_
706.T1, Maole_007322.T1, Maole_007323.T1, Maole_007366.T1, Maole_007462.T1, Maole_
481.T1, Maole_001957.T1, Maole_002221.T1, Maole_002236.T1, Maole_002239.T1, Maole_
152.T1, Maole_003315.T1, Maole_003778.T1, Maole_003953.T1, Maole_003987.T1, Maole_
232.T1, Maole_003315.T1, Maole_003594.T1, Maole_003848.T1, Maole_004142.T1, Maole_
779.T1, Maole_002783.T1, Maole_003326.T1, Maole_003356.T1, Maole_003498.T1, Maole_
455.T1, Maole_016662.T1, Maole_017673.T1, Maole_022100.T1
130.T1
250.T1, Maole_015819.T1, Maole_015820.T1, Maole_016461.T1, Maole_016463.T1, Maole_
627.T1, Maole_013216.T1, Maole_013610.T1, Maole_013625.T1, Maole_014744.T1, Maole_
395.T1, Maole_018862.T1, Maole_019075.T1, Maole_020351.T1, Maole_020655.T1, Maole_
888.T1, Maole_011813.T1, Maole_012845.T1, Maole_015626.T1, Maole_017713.T1, Maole_

750.T1, Maole_004881.T1, Maole_004936.T1, Maole_005102.T1, Maole_005385.T1, Maole_
416.T1, Maole_023259.T1, Maole_023260.T1, Maole_023776.T1, Maole_024056.T1, Maole_
130.T1

799.T1, Maole_006933.T1, Maole_008706.T1, Maole_010934.T1, Maole_011813.T1, Maole_
090.T1, Maole_022471.T1, Maole_023032.T1, Maole_023259.T1, Maole_023260.T1, Maole_
466.T1, Maole_009598.T1, Maole_010110.T1, Maole_010336.T1, Maole_010480.T1, Maole_
636.T1, Maole_002645.T1, Maole_002648.T1, Maole_002680.T1, Maole_003232.T1, Maole_
487.T1, Maole_014488.T1, Maole_016806.T1, Maole_018590.T1, Maole_018592.T1, Maole_
094.T1, Maole_018026.T1, Maole_018086.T1, Maole_019580.T1, Maole_019951.T1, Maole_
802.T1, Maole_016253.T1, Maole_016768.T1, Maole_017309.T1, Maole_018563.T1, Maole_
121.T1
023.T1, Maole_001025.T1, Maole_001038.T1, Maole_001060.T1, Maole_001100.T1, Maole_
500.T1, Maole_005846.T1, Maole_006896.T1, Maole_007145.T1, Maole_007939.T1, Maole_
583.T1, Maole_006830.T1, Maole_007944.T1, Maole_008313.T1, Maole_009505.T1, Maole_
260.T1, Maole_023776.T1, Maole_024121.T1
040.T1, Maole_006559.T1, Maole_006885.T1, Maole_007602.T1, Maole_007923.T1, Maole_
436.T1, Maole_021416.T1, Maole_023259.T1, Maole_023260.T1, Maole_023776.T1, Maole_
076.T1

896.T1, Maole_007342.T1, Maole_007429.T1, Maole_007599.T1, Maole_007843.T1, Maole_
448.T1, Maole_013030.T1, Maole_014516.T1, Maole_014517.T1, Maole_015116.T1, Maole_

846.T1, Maole_005902.T1, Maole_006713.T1, Maole_006972.T1, Maole_007143.T1, Maole_
085.T1
694.T1, Maole_006695.T1, Maole_007024.T1, Maole_007973.T1, Maole_008775.T1, Maole_
407.T1, Maole_019416.T1, Maole_020051.T1
292.T1, Maole_008299.T1, Maole_009317.T1, Maole_009320.T1, Maole_009720.T1, Maole_
082.T1, Maole_015626.T1, Maole_017713.T1, Maole_019159.T1, Maole_020423.T1, Maole_
084.T1, Maole_004129.T1, Maole_004139.T1, Maole_004304.T1, Maole_004457.T1, Maole_
308.T1, Maole_010601.T1, Maole_011128.T1, Maole_011836.T1, Maole_011954.T1, Maole_
398.T1, Maole_017460.T1, Maole_019303.T1, Maole_022244.T1, Maole_022347.T1, Maole_
212.T1, Maole_020226.T1, Maole_020651.T1, Maole_020652.T1
968.T1, Maole_021679.T1, Maole_022600.T1, Maole_023194.T1
827.T1, Maole_011873.T1, Maole_015423.T1, Maole_016343.T1, Maole_016345.T1, Maole_
679.T1, Maole_022600.T1, Maole_023194.T1
065.T1, Maole_003084.T1, Maole_003356.T1, Maole_003593.T1, Maole_003813.T1, Maole_

074.T1, Maole_007504.T1, Maole_007956.T1, Maole_008546.T1, Maole_009067.T1, Maole_
161.T1, Maole_011318.T1, Maole_012785.T1, Maole_014400.T1, Maole_014711.T1, Maole_
922.T1, Maole_018075.T1, Maole_019947.T1, Maole_020238.T1, Maole_021075.T1, Maole_
067.T1, Maole_013216.T1, Maole_013222.T1, Maole_013359.T1, Maole_013625.T1, Maole_
167.T1, Maole_004251.T1, Maole_004819.T1, Maole_004896.T1, Maole_005074.T1, Maole_
950.T1
386.T1, Maole_007927.T1, Maole_008053.T1, Maole_008077.T1, Maole_008338.T1, Maole_
517.T1, Maole_023564.T1, Maole_023753.T1
100.T1, Maole_003227.T1, Maole_003594.T1, Maole_003688.T1, Maole_003773.T1, Maole_
616.T1, Maole_008670.T1, Maole_010434.T1, Maole_011601.T1, Maole_011709.T1, Maole_
311.T1, Maole_006437.T1, Maole_006799.T1, Maole_008596.T1, Maole_008868.T1, Maole_
713.T1, Maole_017992.T1, Maole_018075.T1, Maole_020238.T1, Maole_023564.T1, Maole_
727.T1, Maole_023802.T1

008.T1, Maole_021164.T1, Maole_021520.T1, Maole_022471.T1
021.T1, Maole_020296.T1, Maole_020616.T1, Maole_021763.T1, Maole_023032.T1, Maole_

171.T1, Maole_022716.T1, Maole_022718.T1, Maole_022720.T1, Maole_022911.T1, Maole_
237.T1, Maole_005388.T1, Maole_005518.T1, Maole_006214.T1, Maole_006647.T1, Maole_

289.T1, Maole_010021.T1, Maole_010035.T1, Maole_011406.T1, Maole_012467.T1, Maole_

092.T1, Maole_009248.T1, Maole_009883.T1, Maole_009968.T1, Maole_010920.T1, Maole_
400.T1, Maole_016011.T1, Maole_016012.T1, Maole_016230.T1, Maole_016461.T1, Maole_
521.T1, Maole_006574.T1, Maole_006933.T1, Maole_007270.T1, Maole_007272.T1, Maole_

732.T1, Maole_022733.T1, Maole_022737.T1

826.T1, Maole_003030.T1, Maole_003067.T1, Maole_003596.T1, Maole_003754.T1, Maole_

035.T1, Maole_019820.T1, Maole_022265.T1, Maole_022266.T1, Maole_023245.T1, Maole_
515.T1, Maole_013785.T1, Maole_014580.T1, Maole_016012.T1, Maole_016169.T1, Maole_

523.T1, Maole_009272.T1, Maole_009354.T1, Maole_009506.T1, Maole_009552.T1, Maole_
802.T1

725.T1, Maole_010549.T1, Maole_010606.T1, Maole_010780.T1, Maole_011288.T1, Maole_
738.T1, Maole_021900.T1, Maole_022187.T1, Maole_022607.T1, Maole_023564.T1, Maole_

703.T1, Maole_007707.T1, Maole_007944.T1, Maole_009945.T1, Maole_010427.T1, Maole_
143.T1, Maole_007145.T1, Maole_007386.T1, Maole_007927.T1, Maole_008382.T1, Maole_
560.T1, Maole_003813.T1, Maole_004129.T1, Maole_004880.T1, Maole_005250.T1, Maole_

780.T1, Maole_011345.T1, Maole_011352.T1, Maole_011975.T1, Maole_012082.T1, Maole_
099.T1, Maole_011589.T1, Maole_012008.T1, Maole_012531.T1, Maole_014209.T1, Maole_
008.T1, Maole_021164.T1, Maole_021520.T1, Maole_022471.T1, Maole_023357.T1

444.T1, Maole_011595.T1, Maole_011678.T1, Maole_012299.T1, Maole_012301.T1, Maole_

696.T1, Maole_021905.T1, Maole_022760.T1, Maole_023851.T1
696.T1, Maole_021905.T1, Maole_022760.T1, Maole_023851.T1
696.T1, Maole_021905.T1, Maole_022760.T1, Maole_023851.T1

703.T1, Maole_007427.T1, Maole_007429.T1, Maole_008800.T1, Maole_009619.T1, Maole_
514.T1, Maole_011533.T1, Maole_011536.T1, Maole_012227.T1, Maole_012328.T1, Maole_

221.T1, Maole_005935.T1, Maole_006356.T1, Maole_006561.T1, Maole_006722.T1, Maole_

253.T1, Maole_018810.T1, Maole_019308.T1, Maole_019310.T1, Maole_020367.T1, Maole_

690.T1, Maole_021075.T1, Maole_021076.T1, Maole_021503.T1, Maole_023053.T1, Maole_

275.T1, Maole_016806.T1
018.T1, Maole_008023.T1, Maole_008615.T1, Maole_009890.T1, Maole_009921.T1, Maole_
851.T1
908.T1, Maole_015909.T1, Maole_016671.T1, Maole_016806.T1, Maole_018253.T1, Maole_
905.T1, Maole_022760.T1, Maole_023851.T1

580.T1, Maole_021727.T1, Maole_023299.T1, Maole_023388.T1

625.T1, Maole_008706.T1, Maole_011198.T1, Maole_011946.T1, Maole_012640.T1, Maole_

627.T1, Maole_012678.T1, Maole_013216.T1, Maole_013222.T1, Maole_013359.T1, Maole_

908.T1, Maole_015909.T1, Maole_016671.T1, Maole_016806.T1, Maole_018253.T1, Maole_
908.T1, Maole_015909.T1, Maole_016671.T1, Maole_016806.T1, Maole_018253.T1, Maole_

138.T1, Maole_009142.T1, Maole_009433.T1, Maole_009473.T1, Maole_009740.T1, Maole_
696.T1, Maole_021905.T1, Maole_022760.T1, Maole_023851.T1

309.T1, Maole_021900.T1, Maole_022187.T1, Maole_022607.T1, Maole_023753.T1

499.T1, Maole_020616.T1, Maole_021717.T1, Maole_022051.T1, Maole_023054.T1, Maole_
144.T1, Maole_012145.T1, Maole_013681.T1, Maole_013763.T1, Maole_016155.T1, Maole_
488.T1, Maole_014490.T1, Maole_015659.T1, Maole_017166.T1, Maole_017261.T1, Maole_

189.T1, Maole_007236.T1, Maole_007386.T1, Maole_007927.T1, Maole_009505.T1, Maole_

554.T1, Maole_011345.T1, Maole_011352.T1, Maole_011514.T1, Maole_011533.T1, Maole_

505.T1, Maole_011813.T1, Maole_012093.T1, Maole_012819.T1, Maole_013422.T1, Maole_
283.T1, Maole_013292.T1, Maole_015759.T1, Maole_015762.T1, Maole_016011.T1, Maole_

038.T1, Maole_009719.T1, Maole_012093.T1, Maole_012520.T1, Maole_012554.T1, Maole_
943.T1
678.T1, Maole_012299.T1, Maole_012301.T1, Maole_013121.T1, Maole_013153.T1, Maole_

137.T1, Maole_001192.T1, Maole_001216.T1, Maole_001240.T1, Maole_001356.T1, Maole_
266.T1, Maole_017309.T1, Maole_017673.T1, Maole_017713.T1, Maole_018075.T1, Maole_

171.T1, Maole_016175.T1, Maole_016181.T1, Maole_016393.T1, Maole_016505.T1, Maole_
890.T1, Maole_019427.T1, Maole_021632.T1

128.T1, Maole_021494.T1, Maole_022202.T1, Maole_023761.T1

413.T1, Maole_013571.T1, Maole_013689.T1, Maole_015116.T1, Maole_015577.T1, Maole_

953.T1, Maole_017122.T1, Maole_017515.T1, Maole_018625.T1, Maole_018627.T1, Maole_

932.T1, Maole_017889.T1, Maole_020092.T1, Maole_021727.T1, Maole_023357.T1

121.T1, Maole_022398.T1, Maole_023129.T1, Maole_023130.T1, Maole_023131.T1

101.T1, Maole_007360.T1, Maole_007504.T1, Maole_008182.T1, Maole_009096.T1, Maole_

261.T1, Maole_017547.T1

950.T1, Maole_012276.T1, Maole_012537.T1, Maole_012640.T1, Maole_013319.T1, Maole_
406.T1, Maole_023943.T1

589.T1, Maole_005005.T1, Maole_005101.T1, Maole_005102.T1, Maole_005344.T1, Maole_

114.T1, Maole_012162.T1, Maole_014199.T1, Maole_014480.T1, Maole_016382.T1, Maole_
227.T1, Maole_012853.T1, Maole_013045.T1, Maole_015615.T1, Maole_015908.T1, Maole_

946.T1, Maole_012497.T1, Maole_012499.T1, Maole_012501.T1, Maole_012757.T1, Maole_

713.T1, Maole_022121.T1, Maole_022187.T1, Maole_022760.T1, Maole_023130.T1, Maole_

627.T1, Maole_019439.T1, Maole_021441.T1, Maole_023802.T1

968.T1, Maole_020517.T1, Maole_020565.T1, Maole_020968.T1, Maole_022452.T1

763.T1, Maole_022716.T1, Maole_022718.T1, Maole_023419.T1

101.T1, Maole_005102.T1, Maole_005983.T1, Maole_006095.T1, Maole_006315.T1, Maole_

854.T1, Maole_002141.T1, Maole_002390.T1, Maole_002465.T1, Maole_002512.T1, Maole_

925.T1, Maole_020854.T1, Maole_021425.T1, Maole_021718.T1, Maole_022460.T1, Maole_

838.T1, Maole_016007.T1, Maole_016171.T1, Maole_017131.T1, Maole_019936.T1, Maole_

877.T1, Maole_022970.T1

485.T1, Maole_007473.T1, Maole_008505.T1, Maole_009114.T1, Maole_009181.T1, Maole_

277.T1, Maole_010601.T1, Maole_011515.T1, Maole_011642.T1, Maole_011836.T1, Maole_

817.T1, Maole_012405.T1, Maole_012496.T1, Maole_013054.T1, Maole_014142.T1, Maole_

315.T1, Maole_007855.T1, Maole_008242.T1, Maole_008800.T1, Maole_009213.T1, Maole_

534.T1, Maole_005535.T1, Maole_005838.T1, Maole_005996.T1, Maole_006315.T1, Maole_
842.T1, Maole_022844.T1

629.T1, Maole_020860.T1, Maole_021407.T1, Maole_021422.T1, Maole_022820.T1, Maole_

885.T1, Maole_007126.T1, Maole_007462.T1, Maole_007723.T1, Maole_008105.T1, Maole_
447.T1, Maole_008465.T1, Maole_009754.T1, Maole_009796.T1, Maole_011198.T1, Maole_

554.T1, Maole_005101.T1, Maole_005664.T1, Maole_006040.T1, Maole_006199.T1, Maole_
933.T1, Maole_017109.T1, Maole_017197.T1, Maole_018437.T1, Maole_018561.T1, Maole_

589.T1, Maole_020979.T1, Maole_021195.T1, Maole_021993.T1, Maole_022983.T1, Maole_
594.T1, Maole_003773.T1, Maole_004142.T1, Maole_004819.T1, Maole_005046.T1, Maole_

746.T1, Maole_016166.T1, Maole_016457.T1, Maole_017719.T1, Maole_018100.T1, Maole_

201.T1, Maole_007202.T1, Maole_007203.T1, Maole_007204.T1, Maole_007205.T1, Maole_
195.T1, Maole_007196.T1, Maole_007198.T1, Maole_007199.T1, Maole_007201.T1, Maole_
662.T1, Maole_017673.T1, Maole_022100.T1
248.T1, Maole_002249.T1, Maole_002250.T1, Maole_002253.T1, Maole_002254.T1, Maole_
700.T1, Maole_005702.T1, Maole_005713.T1, Maole_007054.T1, Maole_007081.T1, Maole_
130.T1
062.T1, Maole_005713.T1, Maole_007054.T1, Maole_007081.T1, Maole_009104.T1, Maole_
768.T1, Maole_017309.T1, Maole_020501.T1, Maole_020502.T1, Maole_021185.T1, Maole_
130.T1
121.T1
119.T1, Maole_018229.T1, Maole_018251.T1, Maole_019211.T1, Maole_019276.T1, Maole_
695.T1, Maole_005698.T1, Maole_005700.T1, Maole_005702.T1, Maole_005713.T1, Maole_

056.T1, Maole_024121.T1
117.T1, Maole_020501.T1, Maole_020502.T1, Maole_021175.T1, Maole_021178.T1, Maole_
982.T1, Maole_005713.T1, Maole_007054.T1, Maole_007081.T1, Maole_009104.T1, Maole_
695.T1, Maole_005698.T1, Maole_005700.T1, Maole_005702.T1, Maole_005713.T1, Maole_
583.T1, Maole_006647.T1, Maole_006651.T1, Maole_006652.T1, Maole_006779.T1, Maole_
119.T1, Maole_018229.T1, Maole_018251.T1, Maole_019211.T1, Maole_019276.T1, Maole_
269.T1, Maole_014270.T1, Maole_014272.T1, Maole_014274.T1, Maole_014275.T1, Maole_
996.T1, Maole_006167.T1, Maole_006224.T1, Maole_006226.T1, Maole_006227.T1, Maole_
269.T1, Maole_014270.T1, Maole_014272.T1, Maole_014274.T1, Maole_014275.T1, Maole_
104.T1, Maole_004105.T1, Maole_004639.T1, Maole_004881.T1, Maole_004972.T1, Maole_
526.T1, Maole_014744.T1, Maole_016478.T1, Maole_017309.T1, Maole_017673.T1, Maole_
269.T1, Maole_014270.T1, Maole_014272.T1, Maole_014274.T1, Maole_014275.T1, Maole_
269.T1, Maole_014270.T1, Maole_014272.T1, Maole_014274.T1, Maole_014275.T1, Maole_

703.T1, Maole_007944.T1, Maole_009606.T1, Maole_009607.T1, Maole_009608.T1, Maole_
269.T1, Maole_014270.T1, Maole_014272.T1, Maole_014274.T1, Maole_014275.T1, Maole_

051.T1, Maole_024056.T1, Maole_024069.T1, Maole_024121.T1

265.T1, Maole_014267.T1, Maole_014270.T1, Maole_014272.T1, Maole_014274.T1, Maole_

175.T1, Maole_021178.T1, Maole_021594.T1, Maole_022503.T1
269.T1, Maole_014270.T1, Maole_014272.T1, Maole_014274.T1, Maole_014275.T1, Maole_

201.T1, Maole_007202.T1, Maole_007203.T1, Maole_007204.T1, Maole_007205.T1, Maole_

119.T1, Maole_018229.T1, Maole_018251.T1, Maole_019211.T1, Maole_019276.T1, Maole_

358.T1, Maole_013780.T1, Maole_016253.T1, Maole_016881.T1, Maole_019117.T1, Maole_
269.T1, Maole_014270.T1, Maole_014272.T1, Maole_014274.T1, Maole_014275.T1, Maole_

647.T1, Maole_019130.T1

003.T1, Maole_014265.T1, Maole_014267.T1, Maole_014269.T1, Maole_014270.T1, Maole_

105.T1, Maole_011357.T1, Maole_011358.T1, Maole_013780.T1, Maole_016253.T1, Maole_

068.T1, Maole_023238.T1, Maole_023998.T1

379.T1, Maole_023736.T1, Maole_023737.T1, Maole_023741.T1, Maole_023742.T1
606.T1, Maole_009607.T1, Maole_009608.T1, Maole_009738.T1, Maole_015936.T1, Maole_

317.T1, Maole_009320.T1, Maole_009465.T1, Maole_010105.T1, Maole_011288.T1, Maole_

594.T1, Maole_022100.T1

780.T1, Maole_015873.T1, Maole_015936.T1, Maole_017068.T1, Maole_019117.T1, Maole_

344.T1, Maole_023347.T1, Maole_023983.T1, Maole_023998.T1, Maole_024019.T1, Maole_

618.T1

104.T1, Maole_004105.T1, Maole_004490.T1, Maole_004776.T1, Maole_005062.T1, Maole_

259.T1, Maole_023260.T1, Maole_023776.T1, Maole_024056.T1, Maole_024121.T1

le_000833;Maole_000834

010184.T1, Maole_010531.T1, Maole_010533.T1, Maole_012412.T1, Maole_014489.T1, Ma
007208.T1, Maole_007209.T1, Maole_007211.T1, Maole_007214.T1, Maole_009325.T1, Ma
017243.T1, Maole_017439.T1, Maole_017441.T1, Maole_017626.T1, Maole_017627.T1, Ma
010180.T1, Maole_010181.T1, Maole_010184.T1, Maole_010531.T1, Maole_010533.T1, Ma
013981.T1, Maole_014503.T1, Maole_016782.T1, Maole_018093.T1, Maole_020743.T1, Ma
020418.T1, Maole_020448.T1, Maole_020658.T1, Maole_021291.T1, Maole_022328.T1, Ma
010533.T1, Maole_010839.T1, Maole_012412.T1, Maole_014489.T1, Maole_014652.T1
019211.T1, Maole_019276.T1, Maole_021645.T1, Maole_023134.T1, Maole_023892.T1
022164.T1, Maole_022328.T1, Maole_022920.T1

009666.T1, Maole_010178.T1, Maole_010179.T1, Maole_010180.T1, Maole_010181.T1, Ma
003004.T1, Maole_003005.T1, Maole_003069.T1, Maole_003305.T1, Maole_003405.T1, Ma

014799.T1, Maole_015166.T1, Maole_015292.T1, Maole_016769.T1, Maole_017243.T1, Ma
007203.T1, Maole_007205.T1, Maole_007206.T1, Maole_007208.T1, Maole_007209.T1, Ma
012412.T1, Maole_014489.T1, Maole_014652.T1

021897.T1

005968.T1, Maole_005969.T1, Maole_006013.T1, Maole_006014.T1, Maole_006015.T1, Ma

014135.T1, Maole_014148.T1, Maole_014150.T1, Maole_016802.T1, Maole_016803.T1, Ma

011817.T1, Maole_012083.T1, Maole_012519.T1, Maole_012618.T1, Maole_012895.T1, Ma
006598.T1, Maole_006603.T1, Maole_006604.T1, Maole_007053.T1, Maole_007055.T1, Ma
005695.T1, Maole_005698.T1, Maole_005700.T1, Maole_005702.T1, Maole_005713.T1, Ma
010318.T1, Maole_011520.T1, Maole_012607.T1, Maole_012929.T1, Maole_012936.T1, Ma

005178.T1, Maole_005179.T1, Maole_005180.T1, Maole_005194.T1, Maole_005266.T1, Ma
008212.T1, Maole_009664.T1, Maole_009665.T1, Maole_009666.T1, Maole_009867.T1, Ma
017521.T1, Maole_019116.T1, Maole_019531.T1, Maole_019748.T1, Maole_019749.T1, Ma
017628.T1, Maole_018119.T1, Maole_018229.T1, Maole_018251.T1, Maole_018434.T1, Ma

023134.T1

010184.T1, Maole_011817.T1, Maole_012412.T1, Maole_014489.T1, Maole_015503.T1, Ma
009186.T1, Maole_010649.T1, Maole_012880.T1, Maole_013187.T1, Maole_014526.T1, Ma

017521.T1, Maole_019116.T1, Maole_019531.T1, Maole_019748.T1, Maole_019749.T1, Ma

013806.T1, Maole_013807.T1, Maole_013808.T1, Maole_017285.T1, Maole_017521.T1, Ma
003363.T1, Maole_003481.T1, Maole_003488.T1, Maole_003493.T1, Maole_003681.T1, Ma

015816.T1, Maole_018022.T1, Maole_018119.T1, Maole_018229.T1, Maole_018251.T1, Ma

003005.T1, Maole_003102.T1, Maole_003124.T1, Maole_003296.T1, Maole_003401.T1, Ma

005713.T1, Maole_006011.T1, Maole_006150.T1, Maole_006961.T1, Maole_007054.T1, Ma
019907.T1, Maole_019908.T1, Maole_020950.T1, Maole_020951.T1, Maole_021052.T1, Ma

010274.T1, Maole_010276.T1, Maole_010517.T1, Maole_010889.T1, Maole_010890.T1, Ma

009665.T1, Maole_009666.T1, Maole_010178.T1, Maole_010179.T1, Maole_010180.T1, Ma

014786.T1, Maole_018119.T1, Maole_018229.T1, Maole_018251.T1, Maole_019211.T1, Ma

006014.T1, Maole_006015.T1, Maole_006016.T1, Maole_006017.T1, Maole_009664.T1, Ma

017521.T1, Maole_019116.T1, Maole_019531.T1, Maole_019748.T1, Maole_019749.T1, Ma
017521.T1, Maole_019116.T1, Maole_019531.T1, Maole_019748.T1, Maole_019749.T1, Ma

007201.T1, Maole_007202.T1, Maole_007203.T1, Maole_007204.T1, Maole_007205.T1, Ma

007563.T1, Maole_009104.T1, Maole_009186.T1, Maole_010102.T1, Maole_010649.T1, Ma

000698.T1, Maole_000737.T1, Maole_000738.T1, Maole_000747.T1, Maole_000806.T1, Ma
000690.T1, Maole_000698.T1, Maole_000747.T1, Maole_000749.T1, Maole_000806.T1, Ma
000934.T1, Maole_000995.T1, Maole_001097.T1, Maole_001106.T1, Maole_001137.T1, Ma
002102.T1, Maole_002191.T1, Maole_002288.T1, Maole_002304.T1, Maole_002326.T1, Ma
000806.T1, Maole_000870.T1, Maole_000886.T1, Maole_000893.T1, Maole_000905.T1, Ma
002469.T1, Maole_002652.T1, Maole_002693.T1, Maole_002704.T1, Maole_002715.T1, Ma
002715.T1, Maole_002721.T1, Maole_002724.T1, Maole_002777.T1, Maole_002779.T1, Ma
002721.T1, Maole_002724.T1, Maole_002777.T1, Maole_002778.T1, Maole_002779.T1, Ma
002715.T1, Maole_002721.T1, Maole_002724.T1, Maole_002777.T1, Maole_002779.T1, Ma
002715.T1, Maole_002721.T1, Maole_002724.T1, Maole_002777.T1, Maole_002779.T1, Ma
002704.T1, Maole_002715.T1, Maole_002721.T1, Maole_002724.T1, Maole_002777.T1, Ma
001100.T1, Maole_001277.T1, Maole_001380.T1, Maole_001422.T1, Maole_001467.T1, Ma
002304.T1, Maole_002326.T1, Maole_002396.T1, Maole_002469.T1, Maole_002652.T1, Ma
006194.T1, Maole_006295.T1, Maole_006722.T1, Maole_006992.T1, Maole_007000.T1, Ma
009441.T1, Maole_009509.T1, Maole_009644.T1, Maole_010148.T1, Maole_011670.T1, Ma
008875.T1, Maole_009029.T1, Maole_009092.T1, Maole_009102.T1, Maole_009465.T1, Ma
008596.T1, Maole_009067.T1, Maole_009272.T1, Maole_009320.T1, Maole_010110.T1, Ma
002390.T1, Maole_002591.T1, Maole_002618.T1, Maole_002680.T1, Maole_002736.T1, Ma
003218.T1, Maole_003227.T1, Maole_003356.T1, Maole_003498.T1, Maole_003987.T1, Ma
014470.T1, Maole_014471.T1, Maole_014474.T1, Maole_014475.T1, Maole_014477.T1, Ma
003987.T1, Maole_004072.T1, Maole_004407.T1, Maole_004627.T1, Maole_004673.T1, Ma
007898.T1, Maole_008382.T1, Maole_008946.T1, Maole_009102.T1, Maole_009522.T1, Ma
003148.T1, Maole_003778.T1, Maole_003953.T1, Maole_003987.T1, Maole_004072.T1, Ma
014602.T1, Maole_014641.T1, Maole_014835.T1, Maole_015354.T1, Maole_015835.T1, Ma
011519.T1, Maole_011589.T1, Maole_011930.T1, Maole_013785.T1, Maole_014470.T1, Ma
002556.T1, Maole_002591.T1, Maole_002618.T1, Maole_002627.T1, Maole_002689.T1, Ma
006386.T1, Maole_006581.T1, Maole_006583.T1, Maole_007024.T1, Maole_007342.T1, Ma
009038.T1, Maole_009068.T1, Maole_009364.T1, Maole_009509.T1, Maole_009644.T1, Ma
002389.T1, Maole_002406.T1, Maole_003030.T1, Maole_003100.T1, Maole_003232.T1, Ma
004072.T1, Maole_005074.T1, Maole_005232.T1, Maole_005237.T1, Maole_005293.T1, Ma
004230.T1, Maole_004231.T1, Maole_004268.T1, Maole_004721.T1, Maole_004896.T1, Ma
003501.T1, Maole_003560.T1, Maole_003602.T1, Maole_003848.T1, Maole_003886.T1, Ma

016467.T1, Maole_016855.T1, Maole_017397.T1, Maole_017398.T1, Maole_018850.T1, Ma
015471.T1, Maole_016169.T1, Maole_016201.T1, Maole_016505.T1, Maole_016506.T1, Ma
021185.T1, Maole_021717.T1, Maole_022866.T1, Maole_023076.T1
020423.T1, Maole_022722.T1, Maole_022732.T1, Maole_022733.T1, Maole_022737.T1

006369.T1, Maole_007723.T1, Maole_008242.T1, Maole_008946.T1, Maole_009336.T1, Ma
024121.T1

012022.T1, Maole_012144.T1, Maole_012145.T1, Maole_013561.T1, Maole_013863.T1, Ma
023776.T1, Maole_024056.T1, Maole_024121.T1
011020.T1, Maole_011519.T1, Maole_011930.T1, Maole_011953.T1, Maole_012210.T1, Ma
003245.T1, Maole_003356.T1, Maole_004139.T1, Maole_004167.T1, Maole_004210.T1, Ma
018597.T1, Maole_018598.T1, Maole_018866.T1, Maole_019404.T1, Maole_019405.T1, Ma
020000.T1, Maole_020968.T1, Maole_021164.T1, Maole_021679.T1, Maole_022600.T1, Ma
020501.T1, Maole_020502.T1, Maole_021185.T1, Maole_022100.T1, Maole_022821.T1, Ma

001108.T1, Maole_001126.T1, Maole_001137.T1, Maole_001163.T1, Maole_001168.T1, Ma
009063.T1, Maole_009152.T1, Maole_011321.T1, Maole_011322.T1, Maole_011709.T1, Ma
010016.T1, Maole_011163.T1, Maole_011357.T1, Maole_011358.T1, Maole_012342.T1, Ma

007964.T1, Maole_008122.T1, Maole_009213.T1, Maole_009320.T1, Maole_009724.T1, Ma
024056.T1, Maole_024121.T1

009029.T1, Maole_009063.T1, Maole_009245.T1, Maole_009522.T1, Maole_009534.T1, Ma
015735.T1, Maole_015737.T1, Maole_015738.T1, Maole_015838.T1, Maole_023032.T1, Ma

007145.T1, Maole_008020.T1, Maole_008271.T1, Maole_008809.T1, Maole_008825.T1, Ma

008874.T1, Maole_008875.T1, Maole_009029.T1, Maole_009092.T1, Maole_009968.T1, Ma

010110.T1, Maole_010401.T1, Maole_011161.T1, Maole_011519.T1, Maole_011930.T1, Ma
022732.T1, Maole_022733.T1, Maole_022737.T1
004529.T1, Maole_005023.T1, Maole_005101.T1, Maole_005385.T1, Maole_005557.T1, Ma
012360.T1, Maole_012448.T1, Maole_012817.T1, Maole_012857.T1, Maole_013030.T1, Ma
022678.T1, Maole_022842.T1, Maole_022844.T1

020028.T1, Maole_020657.T1, Maole_021494.T1

003886.T1, Maole_004047.T1, Maole_004129.T1, Maole_004161.T1, Maole_004304.T1, Ma

009096.T1, Maole_009099.T1, Maole_009644.T1, Maole_009918.T1, Maole_010994.T1, Ma
015116.T1, Maole_015137.T1, Maole_015343.T1, Maole_015577.T1, Maole_016398.T1, Ma
021076.T1, Maole_021441.T1, Maole_023564.T1, Maole_023753.T1, Maole_023802.T1
015083.T1, Maole_015471.T1, Maole_016505.T1, Maole_016506.T1, Maole_016544.T1, Ma
005293.T1, Maole_005432.T1, Maole_005492.T1, Maole_005709.T1, Maole_006066.T1, Ma

009038.T1, Maole_009367.T1, Maole_009921.T1, Maole_010383.T1, Maole_012448.T1, Ma

004117.T1, Maole_004167.T1, Maole_004231.T1, Maole_004721.T1, Maole_004819.T1, Ma
011761.T1, Maole_011764.T1, Maole_012180.T1, Maole_013116.T1, Maole_013118.T1, Ma
009068.T1, Maole_009289.T1, Maole_009465.T1, Maole_010021.T1, Maole_010035.T1, Ma
023753.T1

023259.T1, Maole_023260.T1, Maole_023776.T1, Maole_024121.T1

023885.T1
006651.T1, Maole_006652.T1, Maole_007391.T1, Maole_007462.T1, Maole_007973.T1, Ma

012554.T1, Maole_012666.T1, Maole_012805.T1, Maole_013299.T1, Maole_013870.T1, Ma

012299.T1, Maole_012301.T1, Maole_014047.T1, Maole_014059.T1, Maole_015044.T1, Ma
016463.T1, Maole_016467.T1, Maole_016686.T1, Maole_016855.T1, Maole_017397.T1, Ma
007898.T1, Maole_008242.T1, Maole_009100.T1, Maole_009432.T1, Maole_009720.T1, Ma

004070.T1, Maole_004073.T1, Maole_004139.T1, Maole_004206.T1, Maole_004319.T1, Ma

023837.T1
016266.T1, Maole_016278.T1, Maole_017713.T1, Maole_018075.T1, Maole_019031.T1, Ma

010035.T1, Maole_011161.T1, Maole_011519.T1, Maole_011589.T1, Maole_011929.T1, Ma

012520.T1, Maole_012595.T1, Maole_012596.T1, Maole_012598.T1, Maole_012707.T1, Ma
023753.T1

010540.T1, Maole_011589.T1, Maole_012308.T1, Maole_012428.T1, Maole_013121.T1, Ma
008616.T1, Maole_008734.T1, Maole_009038.T1, Maole_010120.T1, Maole_011377.T1, Ma
005276.T1, Maole_005622.T1, Maole_005654.T1, Maole_005983.T1, Maole_006356.T1, Ma

013596.T1, Maole_017623.T1, Maole_018033.T1, Maole_018034.T1, Maole_018035.T1, Ma
014584.T1, Maole_018021.T1, Maole_018577.T1, Maole_018644.T1, Maole_020639.T1, Ma

013121.T1, Maole_013153.T1, Maole_016457.T1, Maole_016696.T1, Maole_018100.T1, Ma

010098.T1, Maole_010114.T1, Maole_010115.T1, Maole_010254.T1, Maole_010356.T1, Ma
012853.T1, Maole_013045.T1, Maole_013616.T1, Maole_015908.T1, Maole_015909.T1, Ma

006960.T1, Maole_007037.T1, Maole_007046.T1, Maole_007386.T1, Maole_007927.T1, Ma

020368.T1, Maole_020370.T1, Maole_022414.T1

023449.T1

010457.T1, Maole_010943.T1, Maole_010944.T1, Maole_010945.T1, Maole_010946.T1, Ma

018810.T1, Maole_019308.T1, Maole_019310.T1, Maole_020367.T1, Maole_020368.T1, Ma

014904.T1, Maole_016349.T1, Maole_016416.T1, Maole_016448.T1, Maole_016521.T1, Ma

013625.T1, Maole_014209.T1, Maole_015083.T1, Maole_015471.T1, Maole_016505.T1, Ma

018810.T1, Maole_019308.T1, Maole_019310.T1, Maole_020367.T1, Maole_020368.T1, Ma
018810.T1, Maole_019308.T1, Maole_019310.T1, Maole_020367.T1, Maole_020368.T1, Ma

014893.T1, Maole_016171.T1, Maole_017131.T1, Maole_019235.T1, Maole_019936.T1, Ma

023418.T1, Maole_023564.T1, Maole_023753.T1
018140.T1, Maole_020212.T1, Maole_020226.T1, Maole_020651.T1, Maole_020652.T1, Ma
017653.T1, Maole_019412.T1, Maole_019416.T1, Maole_020721.T1, Maole_022283.T1, Ma

009506.T1, Maole_009921.T1, Maole_010337.T1, Maole_010389.T1, Maole_011827.T1, Ma

011536.T1, Maole_011975.T1, Maole_012082.T1, Maole_012227.T1, Maole_012853.T1, Ma

013561.T1, Maole_013616.T1, Maole_014088.T1, Maole_014555.T1, Maole_014721.T1, Ma
016171.T1, Maole_016666.T1, Maole_016981.T1, Maole_017131.T1, Maole_018571.T1, Ma

012595.T1, Maole_012596.T1, Maole_012598.T1, Maole_012666.T1, Maole_012805.T1, Ma

016696.T1, Maole_016944.T1, Maole_017623.T1, Maole_018059.T1, Maole_021905.T1, Ma

001516.T1, Maole_001523.T1, Maole_001561.T1, Maole_001608.T1, Maole_001749.T1, Ma
019453.T1, Maole_019799.T1, Maole_021573.T1

016506.T1, Maole_016914.T1, Maole_018073.T1, Maole_018122.T1, Maole_018123.T1, Ma

018886.T1, Maole_019703.T1, Maole_021416.T1, Maole_022121.T1, Maole_022326.T1, Ma

018783.T1, Maole_019427.T1, Maole_019580.T1, Maole_020830.T1, Maole_022482.T1, Ma

009099.T1, Maole_011589.T1, Maole_012707.T1, Maole_015735.T1, Maole_015737.T1, Ma

013321.T1, Maole_013322.T1, Maole_013721.T1, Maole_014451.T1, Maole_014453.T1, Ma

005483.T1, Maole_005505.T1, Maole_005654.T1, Maole_005846.T1, Maole_005902.T1, Ma

016410.T1, Maole_016541.T1, Maole_016542.T1, Maole_017166.T1, Maole_017261.T1, Ma
015909.T1, Maole_016237.T1, Maole_016671.T1, Maole_016806.T1, Maole_018253.T1, Ma

013082.T1, Maole_013616.T1, Maole_015393.T1, Maole_016086.T1, Maole_016087.T1, Ma

023131.T1

006391.T1, Maole_006559.T1, Maole_007108.T1, Maole_007294.T1, Maole_007322.T1, Ma

002693.T1, Maole_003028.T1, Maole_003092.T1, Maole_003356.T1, Maole_003778.T1, Ma

023761.T1

020051.T1, Maole_020851.T1, Maole_022052.T1, Maole_023357.T1, Maole_023563.T1

009724.T1, Maole_011051.T1, Maole_011052.T1, Maole_012524.T1, Maole_012678.T1, Ma

013335.T1, Maole_014314.T1, Maole_014435.T1, Maole_014517.T1, Maole_014685.T1, Ma

014250.T1, Maole_016461.T1, Maole_016463.T1, Maole_016467.T1, Maole_016855.T1, Ma

010277.T1, Maole_010472.T1, Maole_010606.T1, Maole_011642.T1, Maole_011804.T1, Ma

006356.T1, Maole_006382.T1, Maole_006485.T1, Maole_006651.T1, Maole_006652.T1, Ma

023409.T1

009096.T1, Maole_009303.T1, Maole_009317.T1, Maole_009320.T1, Maole_009364.T1, Ma
012180.T1, Maole_012353.T1, Maole_012360.T1, Maole_012766.T1, Maole_012828.T1, Ma

006311.T1, Maole_006356.T1, Maole_006559.T1, Maole_006706.T1, Maole_006799.T1, Ma
019175.T1, Maole_021014.T1, Maole_021015.T1, Maole_021289.T1, Maole_022497.T1

022984.T1
005625.T1, Maole_005626.T1, Maole_005675.T1, Maole_005709.T1, Maole_006066.T1, Ma

019070.T1, Maole_019324.T1, Maole_020238.T1, Maole_021993.T1

007208.T1, Maole_007209.T1, Maole_007211.T1, Maole_007214.T1, Maole_009325.T1, Ma
007202.T1, Maole_007203.T1, Maole_007205.T1, Maole_007206.T1, Maole_007208.T1, Ma

002591.T1, Maole_002707.T1, Maole_003069.T1, Maole_003197.T1, Maole_003305.T1, Ma
007182.T1, Maole_007191.T1, Maole_007193.T1, Maole_007194.T1, Maole_007195.T1, Ma

009186.T1, Maole_009465.T1, Maole_010649.T1, Maole_012880.T1, Maole_013187.T1, Ma
022100.T1, Maole_022821.T1

023134.T1
007054.T1, Maole_007081.T1, Maole_007182.T1, Maole_007191.T1, Maole_007193.T1, Ma

021594.T1, Maole_022100.T1, Maole_022821.T1
009186.T1, Maole_010649.T1, Maole_012880.T1, Maole_013187.T1, Maole_013561.T1, Ma
007054.T1, Maole_007081.T1, Maole_007182.T1, Maole_007191.T1, Maole_007193.T1, Ma
007944.T1, Maole_009317.T1, Maole_009320.T1, Maole_010200.T1, Maole_010203.T1, Ma
023134.T1
014430.T1, Maole_014431.T1, Maole_015116.T1, Maole_015166.T1, Maole_015167.T1, Ma
006262.T1, Maole_006647.T1, Maole_006651.T1, Maole_006652.T1, Maole_006779.T1, Ma
014430.T1, Maole_014431.T1, Maole_015116.T1, Maole_015166.T1, Maole_015167.T1, Ma
004980.T1, Maole_004982.T1, Maole_005013.T1, Maole_005250.T1, Maole_005877.T1, Ma
018119.T1, Maole_018141.T1, Maole_018229.T1, Maole_018251.T1, Maole_019211.T1, Ma
014430.T1, Maole_014431.T1, Maole_015116.T1, Maole_015167.T1, Maole_015667.T1, Ma
014430.T1, Maole_014431.T1, Maole_015116.T1, Maole_015167.T1, Maole_015667.T1, Ma

009738.T1, Maole_010114.T1, Maole_010115.T1, Maole_010254.T1, Maole_010356.T1, Ma
014430.T1, Maole_014431.T1, Maole_015116.T1, Maole_015166.T1, Maole_015167.T1, Ma

014275.T1, Maole_014430.T1, Maole_014431.T1, Maole_014883.T1, Maole_014887.T1, Ma

014430.T1, Maole_014431.T1, Maole_015116.T1, Maole_015167.T1, Maole_015667.T1, Ma

007208.T1, Maole_007209.T1, Maole_007211.T1, Maole_007214.T1, Maole_009325.T1, Ma

023134.T1

020501.T1, Maole_020502.T1, Maole_020579.T1, Maole_021175.T1, Maole_021178.T1, Ma
014430.T1, Maole_014431.T1, Maole_014883.T1, Maole_014887.T1, Maole_015166.T1, Ma

014272.T1, Maole_014274.T1, Maole_014275.T1, Maole_015873.T1

019117.T1, Maole_020501.T1, Maole_020502.T1, Maole_021175.T1, Maole_021178.T1, Ma

017068.T1, Maole_019130.T1, Maole_019618.T1, Maole_022503.T1, Maole_022508.T1, Ma[illegible]

011401.T1, Maole_011670.T1, Maole_012520.T1, Maole_012595.T1, Maole_012596.T1, Ma[illegible]

021175.T1, Maole_021178.T1, Maole_021594.T1

024051.T1, Maole_024069.T1, Maole_024098.T1

005713.T1, Maole_007054.T1, Maole_007081.T1, Maole_007366.T1, Maole_009104.T1, Ma

ole_014652.T1
ole_009326.T1, Maole_010377.T1, Maole_013041.T1, Maole_015999.T1, Maole_020337.T1,
ole_017628.T1, Maole_017630.T1, Maole_017632.T1, Maole_018434.T1, Maole_019618.T1,
ole_012412.T1, Maole_014489.T1, Maole_014652.T1, Maole_015503.T1
ole_021152.T1, Maole_021244.T1, Maole_021574.T1, Maole_021627.T1, Maole_021799.T1,
ole_023960.T1

ole_010184.T1, Maole_010531.T1, Maole_010533.T1, Maole_012412.T1, Maole_014489.T1,
ole_003493.T1, Maole_003684.T1, Maole_003774.T1, Maole_003945.T1, Maole_004079.T1,

ole_017626.T1, Maole_017627.T1, Maole_017628.T1, Maole_017630.T1, Maole_017632.T1,
ole_007211.T1, Maole_007214.T1, Maole_009325.T1, Maole_009326.T1, Maole_010377.T1,

ole_006016.T1, Maole_006017.T1, Maole_006903.T1, Maole_007127.T1, Maole_007726.T1,

ole_016807.T1, Maole_016809.T1, Maole_017692.T1, Maole_019473.T1, Maole_022440.T1

ole_012897.T1, Maole_012902.T1, Maole_014131.T1, Maole_014132.T1, Maole_014133.T1,
ole_007441.T1, Maole_007887.T1, Maole_007888.T1, Maole_008287.T1, Maole_008476.T1,
ole_006486.T1, Maole_006487.T1, Maole_006961.T1, Maole_006987.T1, Maole_007054.T1,
ole_013071.T1, Maole_013581.T1, Maole_013713.T1, Maole_013714.T1, Maole_014126.T1,

ole_005269.T1, Maole_005694.T1, Maole_005695.T1, Maole_005698.T1, Maole_005700.T1,
ole_010105.T1, Maole_010178.T1, Maole_010179.T1, Maole_010180.T1, Maole_010181.T1,
ole_019750.T1, Maole_019753.T1, Maole_019754.T1, Maole_019755.T1, Maole_019756.T1,
ole_019211.T1, Maole_019276.T1, Maole_020950.T1, Maole_020951.T1, Maole_020952.T1,

ole_020026.T1
ole_017439.T1, Maole_017441.T1, Maole_018119.T1, Maole_018229.T1, Maole_018251.T1,

ole_019750.T1, Maole_019753.T1, Maole_019754.T1, Maole_019755.T1, Maole_019756.T1,

ole_018137.T1, Maole_019907.T1, Maole_019908.T1, Maole_023205.T1, Maole_023211.T1,
ole_003774.T1, Maole_003921.T1, Maole_003945.T1, Maole_004053.T1, Maole_004056.T1,

ole_018328.T1, Maole_019065.T1, Maole_019106.T1, Maole_019211.T1, Maole_019276.T1,

ole_003538.T1, Maole_003540.T1, Maole_003774.T1, Maole_003834.T1, Maole_003836.T1,

ole_007081.T1, Maole_007182.T1, Maole_007191.T1, Maole_007193.T1, Maole_007194.T1,
ole_023550.T1, Maole_024105.T1

ole_012742.T1, Maole_014253.T1, Maole_015424.T1, Maole_015426.T1, Maole_015427.T1,

ole_010181.T1, Maole_010184.T1, Maole_012412.T1, Maole_013028.T1, Maole_014489.T1,

ole_019276.T1, Maole_022946.T1, Maole_023134.T1

ole_009665.T1, Maole_009666.T1, Maole_010178.T1, Maole_010179.T1, Maole_010180.T1,

ole_019750.T1, Maole_019753.T1, Maole_019754.T1, Maole_019755.T1, Maole_019756.T1,
ole_019750.T1, Maole_019753.T1, Maole_019754.T1, Maole_019755.T1, Maole_019756.T1,

ole_007208.T1, Maole_007209.T1, Maole_007211.T1, Maole_007214.T1, Maole_008287.T1,

ole_012880.T1, Maole_012895.T1, Maole_012897.T1, Maole_013187.T1, Maole_014526.T1,

ole_000886.T1, Maole_000893.T1, Maole_000945.T1, Maole_000995.T1, Maole_001011.T1,
ole_000856.T1, Maole_000893.T1, Maole_000900.T1, Maole_000921.T1, Maole_000922.T1,
ole_001301.T1, Maole_001375.T1, Maole_001467.T1, Maole_001469.T1, Maole_001501.T1,
ole_002396.T1, Maole_002461.T1, Maole_002469.T1, Maole_002516.T1, Maole_002534.T1,
ole_000963.T1, Maole_001017.T1, Maole_001019.T1, Maole_001020.T1, Maole_001021.T1,
ole_002721.T1, Maole_002724.T1, Maole_002777.T1, Maole_002778.T1, Maole_002779.T1,
ole_002782.T1, Maole_002792.T1, Maole_002950.T1, Maole_002955.T1, Maole_003283.T1,
ole_002780.T1, Maole_002782.T1, Maole_002792.T1, Maole_002950.T1, Maole_002955.T1,
ole_002782.T1, Maole_002950.T1, Maole_002955.T1, Maole_003283.T1, Maole_003326.T1,
ole_002782.T1, Maole_002950.T1, Maole_002955.T1, Maole_003283.T1, Maole_003326.T1,
ole_002779.T1, Maole_002782.T1, Maole_002950.T1, Maole_002955.T1, Maole_003283.T1,
ole_001469.T1, Maole_001516.T1, Maole_001538.T1, Maole_001566.T1, Maole_001608.T1,
ole_002704.T1, Maole_002715.T1, Maole_002747.T1, Maole_002777.T1, Maole_002778.T1,
ole_007322.T1, Maole_007323.T1, Maole_007381.T1, Maole_007383.T1, Maole_007473.T1,
ole_011710.T1, Maole_012114.T1, Maole_012116.T1, Maole_012158.T1, Maole_012375.T1,
ole_009883.T1, Maole_009888.T1, Maole_010427.T1, Maole_011595.T1, Maole_011678.T1,
ole_010241.T1, Maole_010336.T1, Maole_011128.T1, Maole_011519.T1, Maole_011670.T1,
ole_002855.T1, Maole_002902.T1, Maole_003084.T1, Maole_003232.T1, Maole_003498.T1,
ole_004070.T1, Maole_004161.T1, Maole_004251.T1, Maole_004337.T1, Maole_004500.T1,
ole_014480.T1, Maole_014484.T1, Maole_014487.T1, Maole_014488.T1, Maole_014490.T1,
ole_004714.T1, Maole_004972.T1, Maole_005074.T1, Maole_005232.T1, Maole_005237.T1,
ole_009883.T1, Maole_009884.T1, Maole_009885.T1, Maole_010920.T1, Maole_011614.T1,
ole_004188.T1, Maole_004189.T1, Maole_005074.T1, Maole_005232.T1, Maole_005237.T1,
ole_015873.T1, Maole_016011.T1, Maole_016032.T1, Maole_016059.T1, Maole_016410.T1,
ole_018021.T1, Maole_019476.T1, Maole_020499.T1, Maole_020500.T1, Maole_021520.T1,
ole_002736.T1, Maole_002854.T1, Maole_002913.T1, Maole_003066.T1, Maole_003084.T1,
ole_007429.T1, Maole_007455.T1, Maole_008703.T1, Maole_009336.T1, Maole_009465.T1,
ole_009740.T1, Maole_010425.T1, Maole_011670.T1, Maole_011710.T1, Maole_011885.T1,
ole_003594.T1, Maole_003610.T1, Maole_003773.T1, Maole_003976.T1, Maole_004113.T1,
ole_005518.T1, Maole_005675.T1, Maole_006085.T1, Maole_006351.T1, Maole_006468.T1,
ole_005244.T1, Maole_005261.T1, Maole_005453.T1, Maole_005675.T1, Maole_005998.T1,
ole_004161.T1, Maole_004972.T1, Maole_005101.T1, Maole_005250.T1, Maole_005421.T1,

ole_018852.T1, Maole_019045.T1, Maole_019046.T1, Maole_019047.T1, Maole_019049.T1,
ole_017713.T1, Maole_019950.T1, Maole_020238.T1, Maole_020690.T1, Maole_023564.T1,

ole_009599.T1, Maole_009725.T1, Maole_010472.T1, Maole_010549.T1, Maole_010606.T1,

ole_014744.T1, Maole_016169.T1, Maole_016201.T1, Maole_016237.T1, Maole_016473.T1,

ole_012318.T1, Maole_012906.T1, Maole_015116.T1, Maole_017166.T1, Maole_017261.T1,
ole_004626.T1, Maole_004787.T1, Maole_004819.T1, Maole_005005.T1, Maole_005102.T1,
ole_019406.T1, Maole_019407.T1, Maole_019416.T1, Maole_021679.T1, Maole_023357.T1
ole_023194.T1, Maole_023299.T1
ole_023076.T1, Maole_024010.T1

ole_001216.T1, Maole_001240.T1, Maole_001419.T1, Maole_001481.T1, Maole_001523.T1,
ole_013149.T1, Maole_015027.T1, Maole_015085.T1, Maole_015629.T1, Maole_016343.T1,
ole_013780.T1, Maole_014534.T1, Maole_015044.T1, Maole_015677.T1, Maole_016166.T1,

ole_009852.T1, Maole_010110.T1, Maole_010241.T1, Maole_010293.T1, Maole_010336.T1,

ole_009880.T1, Maole_010418.T1, Maole_010423.T1, Maole_010569.T1, Maole_010599.T1,
ole_023259.T1, Maole_023260.T1, Maole_023776.T1, Maole_024056.T1, Maole_024121.T1

ole_009064.T1, Maole_009178.T1, Maole_009209.T1, Maole_009494.T1, Maole_009791.T1,

ole_010308.T1, Maole_010427.T1, Maole_010461.T1, Maole_010849.T1, Maole_010920.T1,

ole_013596.T1, Maole_013889.T1, Maole_014265.T1, Maole_014267.T1, Maole_014269.T1,

ole_006068.T1, Maole_006080.T1, Maole_006391.T1, Maole_006559.T1, Maole_007024.T1,
ole_013163.T1, Maole_013339.T1, Maole_013689.T1, Maole_014314.T1, Maole_014602.T1,

ole_004457.T1, Maole_004564.T1, Maole_004589.T1, Maole_004862.T1, Maole_005046.T1,

ole_011407.T1, Maole_011507.T1, Maole_011508.T1, Maole_011804.T1, Maole_011880.T1,
ole_017992.T1, Maole_018063.T1, Maole_018540.T1, Maole_018541.T1, Maole_019901.T1,

ole_016854.T1, Maole_018032.T1, Maole_018073.T1, Maole_019590.T1, Maole_020525.T1,
ole_006194.T1, Maole_006468.T1, Maole_006561.T1, Maole_006647.T1, Maole_007046.T1,

ole_012805.T1, Maole_013174.T1, Maole_013299.T1, Maole_017163.T1, Maole_017544.T1,

ole_004896.T1, Maole_005896.T1, Maole_005897.T1, Maole_005944.T1, Maole_006066.T1,
ole_013119.T1, Maole_013610.T1, Maole_013744.T1, Maole_013763.T1, Maole_013926.T1,
ole_010293.T1, Maole_011128.T1, Maole_011813.T1, Maole_011873.T1, Maole_012335.T1,

ole_008670.T1, Maole_008809.T1, Maole_008839.T1, Maole_009245.T1, Maole_009364.T1,

ole_013950.T1, Maole_015577.T1, Maole_015766.T1, Maole_015932.T1, Maole_016525.T1,

ole_015423.T1, Maole_016166.T1, Maole_016169.T1, Maole_016446.T1, Maole_016657.T1,
ole_017398.T1, Maole_018140.T1, Maole_018651.T1, Maole_018850.T1, Maole_018852.T1,
ole_010277.T1, Maole_010472.T1, Maole_010479.T1, Maole_011181.T1, Maole_011515.T1,

ole_004601.T1, Maole_004787.T1, Maole_005046.T1, Maole_005102.T1, Maole_005408.T1,

ole_019219.T1, Maole_020438.T1, Maole_020819.T1, Maole_022051.T1, Maole_022090.T1,

ole_012257.T1, Maole_012405.T1, Maole_014270.T1, Maole_014275.T1, Maole_014312.T1,

ole_012819.T1, Maole_014400.T1, Maole_014603.T1, Maole_014847.T1, Maole_015027.T1,

ole_014269.T1, Maole_014270.T1, Maole_016175.T1, Maole_016266.T1, Maole_016349.T1,
ole_011378.T1, Maole_012093.T1, Maole_012805.T1, Maole_013299.T1, Maole_013332.T1,
ole_007386.T1, Maole_007757.T1, Maole_007758.T1, Maole_007827.T1, Maole_007927.T1,

ole_018059.T1, Maole_019159.T1, Maole_019820.T1, Maole_022265.T1, Maole_022266.T1,
ole_021894.T1, Maole_024085.T1

ole_020721.T1, Maole_021905.T1, Maole_022760.T1, Maole_023851.T1

ole_010359.T1, Maole_010362.T1, Maole_010419.T1, Maole_011059.T1, Maole_011264.T1,
ole_016086.T1, Maole_016671.T1, Maole_016806.T1, Maole_018033.T1, Maole_018034.T1,

ole_008546.T1, Maole_009011.T1, Maole_009918.T1, Maole_010338.T1, Maole_010339.T1,

ole_012497.T1, Maole_012499.T1, Maole_012501.T1, Maole_012757.T1, Maole_013082.T1,

ole_020370.T1, Maole_021892.T1, Maole_022414.T1

ole_016525.T1, Maole_016775.T1, Maole_016978.T1, Maole_017403.T1, Maole_019513.T1,

ole_016506.T1, Maole_016544.T1, Maole_016854.T1, Maole_018032.T1, Maole_018073.T1,

ole_020370.T1, Maole_022414.T1
ole_020370.T1, Maole_022414.T1

ole_020979.T1, Maole_021632.T1, Maole_023357.T1, Maole_023491.T1

ole_020940.T1, Maole_021069.T1, Maole_021208.T1, Maole_022768.T1
ole_023496.T1, Maole_023917.T1, Maole_023968.T1

ole_011873.T1, Maole_012093.T1, Maole_012222.T1, Maole_012520.T1, Maole_012595.T1,

ole_013045.T1, Maole_013709.T1, Maole_014904.T1, Maole_015908.T1, Maole_015909.T1,

ole_014744.T1, Maole_014766.T1, Maole_014948.T1, Maole_015921.T1, Maole_016230.T1,
ole_019950.T1, Maole_020092.T1, Maole_020107.T1, Maole_020979.T1, Maole_022187.T1,

ole_012819.T1, Maole_013299.T1, Maole_013579.T1, Maole_013950.T1, Maole_014400.T1,

ole_022760.T1, Maole_023851.T1

ole_001826.T1, Maole_001845.T1, Maole_001853.T1, Maole_001907.T1, Maole_001920.T1,

ole_018139.T1, Maole_019057.T1, Maole_019306.T1, Maole_021091.T1

ole_023129.T1, Maole_023130.T1, Maole_023131.T1, Maole_023259.T1, Maole_023260.T1,

ole_023299.T1

ole_015738.T1, Maole_016181.T1, Maole_018063.T1, Maole_018123.T1, Maole_020436.T1,

ole_014705.T1, Maole_015061.T1, Maole_016032.T1, Maole_016245.T1, Maole_016410.T1,

ole_006031.T1, Maole_006085.T1, Maole_006356.T1, Maole_006559.T1, Maole_006756.T1,

ole_017653.T1, Maole_018595.T1, Maole_018604.T1, Maole_018605.T1, Maole_020548.T1,
ole_018810.T1, Maole_019308.T1, Maole_019310.T1, Maole_020367.T1, Maole_020368.T1,

ole_016088.T1, Maole_016687.T1, Maole_017214.T1, Maole_018460.T1, Maole_018461.T1,

ole_007323.T1, Maole_007500.T1, Maole_008401.T1, Maole_009038.T1, Maole_010356.T1,

ole_004150.T1, Maole_004500.T1, Maole_004771.T1, Maole_005101.T1, Maole_005236.T1,

ole_012828.T1, Maole_013222.T1, Maole_013359.T1, Maole_014579.T1, Maole_014831.T1,

ole_015032.T1, Maole_015137.T1, Maole_015838.T1, Maole_016067.T1, Maole_017861.T1,

ole_017397.T1, Maole_017398.T1, Maole_017460.T1, Maole_018850.T1, Maole_018852.T1

ole_011964.T1, Maole_012220.T1, Maole_013065.T1, Maole_013253.T1, Maole_013335.T1,

ole_006885.T1, Maole_007126.T1, Maole_007148.T1, Maole_007320.T1, Maole_007323.T1,

ole_009509.T1, Maole_009644.T1, Maole_009945.T1, Maole_010110.T1, Maole_010308.T1,
ole_013339.T1, Maole_013763.T1, Maole_014831.T1, Maole_014832.T1, Maole_014867.T1,

ole_007230.T1, Maole_007320.T1, Maole_007366.T1, Maole_007391.T1, Maole_007964.T1,

ole_007035.T1, Maole_007285.T1, Maole_008181.T1, Maole_008264.T1, Maole_008465.T1,

ole_009326.T1, Maole_010377.T1, Maole_013041.T1, Maole_015999.T1, Maole_020337.T1,
ole_007209.T1, Maole_007211.T1, Maole_007214.T1, Maole_009325.T1, Maole_009326.T1,

ole_003945.T1, Maole_004639.T1, Maole_004881.T1, Maole_004972.T1, Maole_004980.T1,
ole_007196.T1, Maole_007198.T1, Maole_007199.T1, Maole_007201.T1, Maole_007202.T1,

ole_013561.T1, Maole_014526.T1, Maole_014744.T1, Maole_017309.T1, Maole_018069.T1,

ole_007194.T1, Maole_007195.T1, Maole_007196.T1, Maole_007198.T1, Maole_007199.T1,

ole_014526.T1, Maole_014744.T1, Maole_017309.T1, Maole_018119.T1, Maole_018229.T1,
ole_007194.T1, Maole_007195.T1, Maole_007196.T1, Maole_007198.T1, Maole_007199.T1,
ole_010336.T1, Maole_011111.T1, Maole_011288.T1, Maole_011357.T1, Maole_011358.T1,

ole_015667.T1, Maole_020715.T1, Maole_020721.T1, Maole_021063.T1, Maole_021416.T1,
ole_007366.T1, Maole_007944.T1, Maole_009317.T1, Maole_009320.T1, Maole_009984.T1,
ole_015667.T1, Maole_020715.T1, Maole_020721.T1, Maole_021063.T1, Maole_021416.T1,
ole_005880.T1, Maole_006214.T1, Maole_006532.T1, Maole_006581.T1, Maole_006583.T1,
ole_019276.T1, Maole_023134.T1
ole_020715.T1, Maole_020721.T1, Maole_021063.T1, Maole_021416.T1, Maole_022500.T1,
ole_020715.T1, Maole_020721.T1, Maole_021063.T1, Maole_021416.T1, Maole_022500.T1,

ole_010359.T1, Maole_010362.T1, Maole_012089.T1, Maole_012789.T1, Maole_012830.T1,
ole_015667.T1, Maole_015873.T1, Maole_020715.T1, Maole_020721.T1, Maole_021063.T1,

ole_015116.T1, Maole_015166.T1, Maole_015167.T1, Maole_015667.T1, Maole_015873.T1,

ole_020715.T1, Maole_020721.T1, Maole_021063.T1, Maole_021416.T1, Maole_022500.T1,

ole_009326.T1, Maole_010377.T1, Maole_013041.T1, Maole_015999.T1, Maole_020337.T1,

ole_021594.T1, Maole_022100.T1, Maole_022821.T1, Maole_023238.T1, Maole_023239.T1,
ole_015873.T1, Maole_018787.T1, Maole_020715.T1, Maole_020721.T1, Maole_021063.T1,

ole_021594.T1, Maole_022100.T1, Maole_022821.T1

ole_023239.T1, Maole_023259.T1, Maole_023260.T1, Maole_024019.T1

ole_012598.T1, Maole_013561.T1, Maole_014265.T1, Maole_014267.T1, Maole_014269.T1,

ole_009186.T1, Maole_010649.T1, Maole_012880.T1, Maole_013187.T1, Maole_014526.T1,

Maole_020346.T1, Maole_021033.T1, Maole_022371.T1, Maole_022372.T1, Maole_022373
Maole_020950.T1, Maole_020951.T1, Maole_020952.T1, Maole_021052.T1, Maole_021133

Maole_021801.T1, Maole_022097.T1, Maole_022164.T1, Maole_023127.T1, Maole_023666

Maole_014652.T1, Maole_014826.T1, Maole_015503.T1, Maole_015784.T1, Maole_017955
Maole_004489.T1, Maole_004491.T1, Maole_004679.T1, Maole_004870.T1, Maole_004968

Maole_018434.T1, Maole_020900.T1, Maole_021052.T1, Maole_022500.T1, Maole_022504
Maole_011014.T1, Maole_012027.T1, Maole_012028.T1, Maole_013042.T1, Maole_014526

Maole_007728.T1, Maole_007730.T1, Maole_008673.T1, Maole_008917.T1, Maole_009664

Maole_014134.T1, Maole_014135.T1, Maole_014148.T1, Maole_014150.T1, Maole_015427
Maole_010889.T1, Maole_010890.T1, Maole_012383.T1, Maole_012384.T1, Maole_015203
Maole_007081.T1, Maole_007182.T1, Maole_007191.T1, Maole_007193.T1, Maole_007194
Maole_015433.T1, Maole_015455.T1, Maole_016034.T1, Maole_016670.T1, Maole_016782

Maole_005702.T1, Maole_005713.T1, Maole_006011.T1, Maole_006150.T1, Maole_006486
Maole_010184.T1, Maole_011036.T1, Maole_011062.T1, Maole_011817.T1, Maole_012383
Maole_019757.T1, Maole_021133.T1, Maole_021135.T1, Maole_023204.T1, Maole_023205
Maole_023127.T1, Maole_023134.T1, Maole_023348.T1, Maole_023352.T1, Maole_023550

Maole_019211.T1, Maole_019276.T1, Maole_019418.T1, Maole_019419.T1, Maole_019420

Maole_019757.T1, Maole_021133.T1, Maole_021135.T1, Maole_023204.T1, Maole_023205

Maole_024088.T1, Maole_024112.T1
Maole_004057.T1, Maole_004058.T1, Maole_004175.T1, Maole_004372.T1, Maole_004375

Maole_020748.T1, Maole_020900.T1, Maole_021963.T1, Maole_023024.T1, Maole_023134

Maole_004233.T1, Maole_004288.T1, Maole_004342.T1, Maole_004354.T1, Maole_004362

Maole_007195.T1, Maole_007196.T1, Maole_007198.T1, Maole_007199.T1, Maole_007201

Maole_015768.T1, Maole_015774.T1, Maole_015815.T1, Maole_015816.T1, Maole_016516

Maole_015503.T1, Maole_016334.T1, Maole_016766.T1, Maole_017439.T1, Maole_017441

Maole_010181.T1, Maole_010184.T1, Maole_010531.T1, Maole_010533.T1, Maole_011817

Maole_019757.T1, Maole_021133.T1, Maole_021135.T1, Maole_023204.T1, Maole_023205
Maole_019757.T1, Maole_021133.T1, Maole_021135.T1, Maole_023204.T1, Maole_023205

Maole_008288.T1, Maole_008931.T1, Maole_009325.T1, Maole_009326.T1, Maole_010377

Maole_018119.T1, Maole_018229.T1, Maole_018251.T1, Maole_018328.T1, Maole_019106

Maole_001017.T1, Maole_001019.T1, Maole_001020.T1, Maole_001021.T1, Maole_001022
Maole_000934.T1, Maole_000944.T1, Maole_000995.T1, Maole_001011.T1, Maole_001013
Maole_001516.T1, Maole_001545.T1, Maole_001608.T1, Maole_001610.T1, Maole_001612
Maole_002693.T1, Maole_002704.T1, Maole_002715.T1, Maole_002721.T1, Maole_002724
Maole_001022.T1, Maole_001037.T1, Maole_001038.T1, Maole_001096.T1, Maole_001097
Maole_002780.T1, Maole_002782.T1, Maole_002792.T1, Maole_002950.T1, Maole_002955
Maole_003326.T1, Maole_003557.T1, Maole_003560.T1, Maole_003905.T1, Maole_003994
Maole_003283.T1, Maole_003326.T1, Maole_003557.T1, Maole_003560.T1, Maole_003905
Maole_003557.T1, Maole_003560.T1, Maole_003905.T1, Maole_003994.T1, Maole_004229
Maole_003557.T1, Maole_003560.T1, Maole_003905.T1, Maole_003994.T1, Maole_004229
Maole_003326.T1, Maole_003557.T1, Maole_003560.T1, Maole_003905.T1, Maole_003994
Maole_001609.T1, Maole_001616.T1, Maole_001722.T1, Maole_001948.T1, Maole_001968
Maole_002779.T1, Maole_002780.T1, Maole_002782.T1, Maole_002792.T1, Maole_002950
Maole_007979.T1, Maole_007982.T1, Maole_008636.T1, Maole_008747.T1, Maole_008763
Maole_012507.T1, Maole_012809.T1, Maole_013456.T1, Maole_014466.T1, Maole_014469
Maole_011813.T1, Maole_011873.T1, Maole_013121.T1, Maole_013416.T1, Maole_015626
Maole_011885.T1, Maole_012116.T1, Maole_012405.T1, Maole_012507.T1, Maole_013130
Maole_003778.T1, Maole_003825.T1, Maole_003987.T1, Maole_004003.T1, Maole_004072
Maole_004696.T1, Maole_004697.T1, Maole_004896.T1, Maole_005102.T1, Maole_005152
Maole_016363.T1, Maole_018590.T1, Maole_018592.T1, Maole_018595.T1, Maole_018597
Maole_005293.T1, Maole_005518.T1, Maole_005675.T1, Maole_006040.T1, Maole_006085
Maole_011616.T1, Maole_011813.T1, Maole_012819.T1, Maole_013454.T1, Maole_014744
Maole_005293.T1, Maole_005518.T1, Maole_005675.T1, Maole_005998.T1, Maole_006085
Maole_016970.T1, Maole_017094.T1, Maole_017395.T1, Maole_018026.T1, Maole_018086
Maole_022973.T1, Maole_023032.T1, Maole_023259.T1, Maole_023260.T1, Maole_023561
Maole_003092.T1, Maole_003101.T1, Maole_003148.T1, Maole_003315.T1, Maole_003498
Maole_009880.T1, Maole_009883.T1, Maole_009884.T1, Maole_009885.T1, Maole_010461
Maole_012116.T1, Maole_012158.T1, Maole_012405.T1, Maole_012507.T1, Maole_012805
Maole_004164.T1, Maole_004167.T1, Maole_004210.T1, Maole_004231.T1, Maole_004363
Maole_006649.T1, Maole_006718.T1, Maole_007035.T1, Maole_007183.T1, Maole_007285
Maole_006083.T1, Maole_006085.T1, Maole_006189.T1, Maole_006235.T1, Maole_006236
Maole_005483.T1, Maole_005607.T1, Maole_005818.T1, Maole_005902.T1, Maole_006031

Maole_019477.T1, Maole_019478.T1, Maole_022891.T1
Maole_023753.T1

Maole_011275.T1, Maole_011288.T1, Maole_011444.T1, Maole_011642.T1, Maole_012520

Maole_016478.T1, Maole_016978.T1, Maole_017713.T1, Maole_018075.T1, Maole_018141

Maole_017653.T1, Maole_017922.T1, Maole_019057.T1, Maole_020499.T1, Maole_022090
Maole_005902.T1, Maole_006066.T1, Maole_006083.T1, Maole_006194.T1, Maole_006295

Maole_001608.T1, Maole_001616.T1, Maole_001725.T1, Maole_001749.T1, Maole_001826
Maole_016345.T1, Maole_016665.T1, Maole_016736.T1, Maole_016806.T1, Maole_016855
Maole_016253.T1, Maole_016393.T1, Maole_020501.T1, Maole_020502.T1, Maole_020819

Maole_010601.T1, Maole_010642.T1, Maole_011216.T1, Maole_011406.T1, Maole_011519

Maole_010780.T1, Maole_011110.T1, Maole_011111.T1, Maole_011159.T1, Maole_011288

Maole_009817.T1, Maole_009961.T1, Maole_010589.T1, Maole_010601.T1, Maole_011377

Maole_011345.T1, Maole_011352.T1, Maole_011401.T1, Maole_011595.T1, Maole_011678

Maole_014270.T1, Maole_014272.T1, Maole_014274.T1, Maole_014275.T1, Maole_014711

Maole_007189.T1, Maole_007322.T1, Maole_007323.T1, Maole_007462.T1, Maole_007859
Maole_014685.T1, Maole_015679.T1, Maole_016444.T1, Maole_016890.T1, Maole_017476

Maole_005101.T1, Maole_005102.T1, Maole_005408.T1, Maole_005483.T1, Maole_005505

Maole_012004.T1, Maole_013130.T1, Maole_013486.T1, Maole_013607.T1, Maole_013889
Maole_020438.T1, Maole_021240.T1, Maole_023519.T1, Maole_023523.T1

Maole_021075.T1, Maole_021076.T1, Maole_021089.T1, Maole_021207.T1, Maole_022973
Maole_007360.T1, Maole_008264.T1, Maole_008512.T1, Maole_008546.T1, Maole_008839

Maole_017660.T1, Maole_017900.T1, Maole_018604.T1, Maole_018605.T1, Maole_018644

Maole_006189.T1, Maole_006194.T1, Maole_006295.T1, Maole_006391.T1, Maole_006485
Maole_014580.T1, Maole_015629.T1, Maole_017109.T1, Maole_017124.T1, Maole_017220
Maole_012819.T1, Maole_013422.T1, Maole_013561.T1, Maole_014088.T1, Maole_014721

Maole_009509.T1, Maole_009636.T1, Maole_009644.T1, Maole_009945.T1, Maole_010569

Maole_016612.T1, Maole_016971.T1, Maole_016977.T1, Maole_016978.T1, Maole_017222

Maole_016770.T1, Maole_016970.T1, Maole_017128.T1, Maole_018033.T1, Maole_018034
Maole_019425.T1, Maole_019929.T1, Maole_020092.T1, Maole_020441.T1, Maole_021130
Maole_011642.T1, Maole_012665.T1, Maole_013157.T1, Maole_013230.T1, Maole_013335

Maole_005902.T1, Maole_005908.T1, Maole_006933.T1, Maole_007037.T1, Maole_007143

Maole_022471.T1, Maole_023753.T1, Maole_023893.T1

Maole_014516.T1, Maole_014704.T1, Maole_014711.T1, Maole_015305.T1, Maole_015657

Maole_015140.T1, Maole_015785.T1, Maole_015802.T1, Maole_015962.T1, Maole_016169

Maole_016696.T1, Maole_021415.T1, Maole_022760.T1, Maole_023332.T1, Maole_023728
Maole_015844.T1, Maole_017163.T1, Maole_017313.T1, Maole_017544.T1, Maole_017900
Maole_008077.T1, Maole_008318.T1, Maole_008734.T1, Maole_009505.T1, Maole_009506

Maole_023245.T1, Maole_023837.T1

Maole_012626.T1, Maole_012627.T1, Maole_012789.T1, Maole_012830.T1, Maole_013216
Maole_018035.T1, Maole_018253.T1, Maole_018810.T1, Maole_019308.T1, Maole_019310

Maole_010982.T1, Maole_010994.T1, Maole_011289.T1, Maole_011642.T1, Maole_012360

Maole_013616.T1, Maole_015393.T1, Maole_016086.T1, Maole_016087.T1, Maole_016088

Maole_020238.T1, Maole_020402.T1, Maole_020589.T1, Maole_021475.T1, Maole_022497

Maole_019590.T1, Maole_020525.T1, Maole_020690.T1, Maole_021075.T1, Maole_021076

Maole_012596.T1, Maole_012598.T1, Maole_012809.T1, Maole_013630.T1, Maole_014400

Maole_016226.T1, Maole_016671.T1, Maole_016806.T1, Maole_018253.T1, Maole_018563

Maole_016525.T1, Maole_016657.T1, Maole_016951.T1, Maole_017163.T1, Maole_017237
Maole_023357.T1

Maole_014555.T1, Maole_015577.T1, Maole_015835.T1, Maole_015844.T1, Maole_015932

Maole_001947.T1, Maole_001948.T1, Maole_002162.T1, Maole_002221.T1, Maole_002276

Maole_023776.T1, Maole_024056.T1, Maole_024121.T1

Maole_020479.T1, Maole_021900.T1, Maole_022187.T1, Maole_022452.T1, Maole_023564

Maole_016416.T1, Maole_016470.T1, Maole_016521.T1, Maole_016735.T1, Maole_016877

Maole_007143.T1, Maole_007145.T1, Maole_007236.T1, Maole_007322.T1, Maole_007323

Maole_021877.T1, Maole_022283.T1, Maole_022700.T1, Maole_022906.T1, Maole_023053
Maole_020370.T1, Maole_021892.T1, Maole_022414.T1

Maole_018463.T1, Maole_018465.T1, Maole_018928.T1, Maole_018929.T1, Maole_018931

Maole_010359.T1, Maole_010362.T1, Maole_010425.T1, Maole_010461.T1, Maole_011407[illegible]

Maole_005293.T1, Maole_005514.T1, Maole_005935.T1, Maole_006444.T1, Maole_006445[illegible]

Maole_014832.T1, Maole_015154.T1, Maole_015610.T1, Maole_016115.T1, Maole_016544

Maole_018625.T1, Maole_018627.T1, Maole_019320.T1, Maole_019439.T1, Maole_021300

Maole_013744.T1, Maole_013863.T1, Maole_014435.T1, Maole_014926.T1, Maole_015032

Maole_007823.T1, Maole_008299.T1, Maole_008315.T1, Maole_008338.T1, Maole_008505

Maole_011710.T1, Maole_011885.T1, Maole_011953.T1, Maole_012158.T1, Maole_012236
Maole_015464.T1, Maole_015610.T1, Maole_016082.T1, Maole_016155.T1, Maole_016733

Maole_009038.T1, Maole_009289.T1, Maole_009466.T1, Maole_009598.T1, Maole_010021

Maole_008713.T1, Maole_009181.T1, Maole_009724.T1, Maole_009754.T1, Maole_009796

Maole_020346.T1, Maole_021033.T1, Maole_022371.T1, Maole_022372.T1, Maole_022373
Maole_010377.T1, Maole_013042.T1, Maole_014526.T1, Maole_015999.T1, Maole_018119

Maole_004982.T1, Maole_005013.T1, Maole_005807.T1, Maole_005877.T1, Maole_005880
Maole_007203.T1, Maole_007204.T1, Maole_007205.T1, Maole_007206.T1, Maole_007208

Maole_018119.T1, Maole_018229.T1, Maole_018251.T1, Maole_018328.T1, Maole_019106

Maole_007201.T1, Maole_007202.T1, Maole_007203.T1, Maole_007204.T1, Maole_007205

Maole_018251.T1, Maole_019211.T1, Maole_019276.T1, Maole_019618.T1, Maole_020748
Maole_007201.T1, Maole_007202.T1, Maole_007203.T1, Maole_007204.T1, Maole_007205
Maole_011524.T1, Maole_011670.T1, Maole_013561.T1, Maole_013780.T1, Maole_014265

Maole_022500.T1, Maole_022503.T1, Maole_022504.T1, Maole_022507.T1, Maole_022508
Maole_010200.T1, Maole_010203.T1, Maole_010336.T1, Maole_011110.T1, Maole_011111
Maole_022500.T1, Maole_022504.T1, Maole_022507.T1, Maole_022510.T1, Maole_022511
Maole_006647.T1, Maole_006651.T1, Maole_006652.T1, Maole_006779.T1, Maole_007827

Maole_022507.T1, Maole_022510.T1, Maole_022511.T1, Maole_022513.T1, Maole_023259
Maole_022507.T1, Maole_022510.T1, Maole_022511.T1, Maole_022513.T1, Maole_023259

Maole_013780.T1, Maole_013992.T1, Maole_013993.T1, Maole_013994.T1, Maole_014003
Maole_021416.T1, Maole_022500.T1, Maole_022503.T1, Maole_022504.T1, Maole_022507

Maole_018787.T1, Maole_020715.T1, Maole_020721.T1, Maole_021063.T1, Maole_021415

Maole_022507.T1, Maole_022510.T1, Maole_022511.T1, Maole_022513.T1, Maole_023259

Maole_020346.T1, Maole_021033.T1, Maole_022371.T1, Maole_022372.T1, Maole_022373

Maole_023241.T1, Maole_023344.T1, Maole_023347.T1, Maole_023983.T1, Maole_024019
Maole_021415.T1, Maole_021416.T1, Maole_022500.T1, Maole_022503.T1, Maole_022504

Maole_014270.T1, Maole_014272.T1, Maole_014274.T1, Maole_014275.T1, Maole_014430

Maole_015873.T1, Maole_018119.T1, Maole_018229.T1, Maole_018251.T1, Maole_018328

.T1, Maole_023452.T1, Maole_023453.T1
.T1, Maole_021135.T1, Maole_023348.T1, Maole_023352.T1, Maole_023550.T1, Maole_024

.T1

.T1, Maole_017956.T1, Maole_023511.T1
.T1, Maole_004969.T1, Maole_004987.T1, Maole_005107.T1, Maole_005145.T1, Maole_005

.T1, Maole_022507.T1, Maole_022510.T1, Maole_022511.T1, Maole_022513.T1, Maole_022
.T1, Maole_015999.T1, Maole_018119.T1, Maole_020114.T1, Maole_020115.T1, Maole_020

.T1, Maole_009665.T1, Maole_009666.T1, Maole_009867.T1, Maole_010178.T1, Maole_010

.T1, Maole_016802.T1, Maole_016803.T1, Maole_016807.T1, Maole_016809.T1, Maole_017
.T1, Maole_015774.T1, Maole_015784.T1, Maole_015853.T1, Maole_017527.T1, Maole_017
.T1, Maole_007195.T1, Maole_007196.T1, Maole_007198.T1, Maole_007199.T1, Maole_007
.T1, Maole_018871.T1, Maole_018906.T1, Maole_020020.T1, Maole_020217.T1, Maole_020

.T1, Maole_006487.T1, Maole_006961.T1, Maole_006987.T1, Maole_006990.T1, Maole_007
.T1, Maole_012384.T1, Maole_012412.T1, Maole_012510.T1, Maole_012895.T1, Maole_013
.T1, Maole_023206.T1, Maole_023208.T1, Maole_023211.T1
.T1

.T1, Maole_019618.T1, Maole_020748.T1, Maole_021133.T1, Maole_021135.T1, Maole_021

.T1, Maole_023206.T1, Maole_023208.T1, Maole_023211.T1

.T1, Maole_004377.T1, Maole_004639.T1, Maole_004679.T1, Maole_004870.T1, Maole_004

.T1

.T1, Maole_004613.T1, Maole_004614.T1, Maole_004615.T1, Maole_004752.T1, Maole_004

.T1, Maole_007202.T1, Maole_007203.T1, Maole_007204.T1, Maole_007205.T1, Maole_007

.T1, Maole_017521.T1, Maole_017527.T1, Maole_018137.T1, Maole_018667.T1, Maole_019

.T1

.T1, Maole_012089.T1, Maole_012412.T1, Maole_013992.T1, Maole_013993.T1, Maole_013

.T1, Maole_023206.T1, Maole_023208.T1, Maole_023211.T1
.T1, Maole_023206.T1, Maole_023208.T1, Maole_023211.T1

.T1, Maole_010787.T1, Maole_010789.T1, Maole_013041.T1, Maole_013640.T1, Maole_015

.T1, Maole_019211.T1, Maole_019276.T1, Maole_019618.T1, Maole_020748.T1, Maole_021

.T1, Maole_001071.T1, Maole_001097.T1, Maole_001100.T1, Maole_001120.T1, Maole_001
.T1, Maole_001019.T1, Maole_001020.T1, Maole_001021.T1, Maole_001022.T1, Maole_001
.T1, Maole_001616.T1, Maole_001631.T1, Maole_001749.T1, Maole_001757.T1, Maole_001
.T1, Maole_002742.T1, Maole_002747.T1, Maole_002777.T1, Maole_002778.T1, Maole_002
.T1, Maole_001136.T1, Maole_001137.T1, Maole_001138.T1, Maole_001240.T1, Maole_001
.T1, Maole_003283.T1, Maole_003326.T1, Maole_003557.T1, Maole_003560.T1, Maole_003
.T1, Maole_004229.T1, Maole_004238.T1, Maole_004246.T1, Maole_004406.T1, Maole_004
.T1, Maole_003994.T1, Maole_004229.T1, Maole_004238.T1, Maole_004246.T1, Maole_004
.T1, Maole_004238.T1, Maole_004246.T1, Maole_004406.T1, Maole_004432.T1, Maole_004
.T1, Maole_004238.T1, Maole_004246.T1, Maole_004406.T1, Maole_004432.T1, Maole_004
.T1, Maole_004229.T1, Maole_004238.T1, Maole_004246.T1, Maole_004406.T1, Maole_004
.T1, Maole_001986.T1, Maole_001987.T1, Maole_002162.T1, Maole_002189.T1, Maole_002
.T1, Maole_002955.T1, Maole_003283.T1, Maole_003309.T1, Maole_003326.T1, Maole_003
.T1, Maole_008839.T1, Maole_009003.T1, Maole_009053.T1, Maole_009054.T1, Maole_009
.T1, Maole_014470.T1, Maole_014471.T1, Maole_014474.T1, Maole_014475.T1, Maole_014
.T1, Maole_015757.T1, Maole_015759.T1, Maole_015760.T1, Maole_015762.T1, Maole_016
.T1, Maole_013299.T1, Maole_014466.T1, Maole_014475.T1, Maole_014477.T1, Maole_014
.T1, Maole_004230.T1, Maole_004319.T1, Maole_004337.T1, Maole_004407.T1, Maole_004
.T1, Maole_005421.T1, Maole_005432.T1, Maole_005461.T1, Maole_005483.T1, Maole_005
.T1, Maole_018598.T1, Maole_018599.T1, Maole_018603.T1, Maole_018604.T1, Maole_018
.T1, Maole_006351.T1, Maole_006468.T1, Maole_006649.T1, Maole_006702.T1, Maole_006
.T1, Maole_014963.T1, Maole_014964.T1, Maole_015044.T1, Maole_015423.T1, Maole_016
.T1, Maole_006468.T1, Maole_006649.T1, Maole_006718.T1, Maole_006756.T1, Maole_007
.T1, Maole_018847.T1, Maole_019951.T1, Maole_020000.T1, Maole_020066.T1, Maole_020
.T1, Maole_023562.T1, Maole_023563.T1, Maole_023564.T1, Maole_023753.T1, Maole_023
.T1, Maole_003522.T1, Maole_003778.T1, Maole_003848.T1, Maole_003953.T1, Maole_003
.T1, Maole_010606.T1, Maole_011288.T1, Maole_011512.T1, Maole_011614.T1, Maole_011
.T1, Maole_012809.T1, Maole_012820.T1, Maole_013174.T1, Maole_013299.T1, Maole_013
.T1, Maole_004819.T1, Maole_004896.T1, Maole_005102.T1, Maole_005152.T1, Maole_005
.T1, Maole_007427.T1, Maole_007690.T1, Maole_007855.T1, Maole_008706.T1, Maole_008
.T1, Maole_006480.T1, Maole_006521.T1, Maole_006799.T1, Maole_006850.T1, Maole_006
.T1, Maole_006211.T1, Maole_006356.T1, Maole_006559.T1, Maole_007143.T1, Maole_007

.T1, Maole_012595.T1, Maole_012596.T1, Maole_012598.T1, Maole_012707.T1, Maole_013

.T1, Maole_019299.T1, Maole_019929.T1, Maole_020238.T1, Maole_021503.T1, Maole_021

.T1, Maole_022283.T1, Maole_023032.T1, Maole_023259.T1, Maole_023260.T1, Maole_023
.T1, Maole_006703.T1, Maole_006850.T1, Maole_007108.T1, Maole_007143.T1, Maole_007

.T1, Maole_001907.T1, Maole_001918.T1, Maole_001936.T1, Maole_001947.T1, Maole_001
.T1, Maole_017085.T1, Maole_017086.T1, Maole_017087.T1, Maole_017092.T1, Maole_017
.T1, Maole_022100.T1, Maole_022471.T1, Maole_022821.T1

.T1, Maole_011930.T1, Maole_013045.T1, Maole_013163.T1, Maole_013792.T1, Maole_013

.T1, Maole_011321.T1, Maole_011322.T1, Maole_011512.T1, Maole_011867.T1, Maole_012

.T1, Maole_011378.T1, Maole_011706.T1, Maole_011762.T1, Maole_012434.T1, Maole_012

.T1, Maole_011975.T1, Maole_012082.T1, Maole_012299.T1, Maole_012301.T1, Maole_012

.T1, Maole_015577.T1, Maole_016217.T1, Maole_016457.T1, Maole_016925.T1, Maole_017

.T1, Maole_007912.T1, Maole_008382.T1, Maole_008512.T1, Maole_008800.T1, Maole_008
.T1, Maole_017839.T1, Maole_018075.T1, Maole_018863.T1, Maole_018864.T1, Maole_019

.T1, Maole_005607.T1, Maole_005908.T1, Maole_006095.T1, Maole_006365.T1, Maole_006

.T1, Maole_014641.T1, Maole_015305.T1, Maole_016032.T1, Maole_016242.T1, Maole_016

.T1
.T1, Maole_009267.T1, Maole_009268.T1, Maole_009446.T1, Maole_009918.T1, Maole_010

.T1, Maole_019175.T1, Maole_019562.T1, Maole_021063.T1, Maole_021212.T1, Maole_021

.T1, Maole_006897.T1, Maole_007035.T1, Maole_007108.T1, Maole_007473.T1, Maole_008
.T1, Maole_017313.T1, Maole_017718.T1, Maole_018140.T1, Maole_018579.T1, Maole_020
.T1, Maole_014744.T1, Maole_014835.T1, Maole_014948.T1, Maole_015324.T1, Maole_015

.T1, Maole_011601.T1, Maole_011710.T1, Maole_011761.T1, Maole_011764.T1, Maole_012

.T1, Maole_017331.T1, Maole_017644.T1, Maole_017861.T1, Maole_018571.T1, Maole_019

.T1, Maole_018035.T1, Maole_018327.T1, Maole_019820.T1, Maole_020517.T1, Maole_021
.T1, Maole_021530.T1, Maole_021573.T1, Maole_022051.T1
.T1, Maole_013695.T1, Maole_014435.T1, Maole_014746.T1, Maole_015032.T1, Maole_015

.T1, Maole_007145.T1, Maole_007176.T1, Maole_007189.T1, Maole_008141.T1, Maole_008

.T1, Maole_015737.T1, Maole_015794.T1, Maole_016410.T1, Maole_017065.T1, Maole_017

.T1, Maole_016455.T1, Maole_016662.T1, Maole_017245.T1, Maole_017515.T1, Maole_017

.T1, Maole_023851.T1, Maole_024091.T1
.T1, Maole_018644.T1, Maole_020605.T1, Maole_020610.T1, Maole_020826.T1, Maole_021
.T1, Maole_009817.T1, Maole_009921.T1, Maole_011406.T1, Maole_011827.T1, Maole_011

.T1, Maole_013625.T1, Maole_014669.T1, Maole_014704.T1, Maole_015932.T1, Maole_016
.T1, Maole_019820.T1, Maole_020367.T1, Maole_020368.T1, Maole_020370.T1, Maole_021

.T1, Maole_012448.T1, Maole_012817.T1, Maole_013339.T1, Maole_013456.T1, Maole_013

.T1, Maole_016687.T1, Maole_017214.T1, Maole_018460.T1, Maole_018461.T1, Maole_018

.T1

.T1, Maole_021089.T1, Maole_021207.T1, Maole_022089.T1

.T1, Maole_014496.T1, Maole_014919.T1, Maole_015753.T1, Maole_016181.T1, Maole_016

.T1, Maole_018810.T1, Maole_019308.T1, Maole_019310.T1, Maole_020367.T1, Maole_020

.T1, Maole_017309.T1, Maole_017383.T1, Maole_017415.T1, Maole_017515.T1, Maole_017

.T1, Maole_016455.T1, Maole_016971.T1, Maole_017135.T1, Maole_017222.T1, Maole_017

.T1, Maole_002389.T1, Maole_002426.T1, Maole_002458.T1, Maole_002505.T1, Maole_002

.T1, Maole_023753.T1

.T1, Maole_017403.T1, Maole_017586.T1, Maole_019149.T1, Maole_019215.T1, Maole_019

.T1, Maole_008020.T1, Maole_008053.T1, Maole_008401.T1, Maole_008489.T1, Maole_008

.T1

.T1, Maole_018932.T1, Maole_019031.T1, Maole_019219.T1, Maole_019435.T1, Maole_019

.T1, Maole_011523.T1, Maole_011678.T1, Maole_011873.T1, Maole_012236.T1, Maole_012

.T1, Maole_006559.T1, Maole_006649.T1, Maole_006718.T1, Maole_006885.T1, Maole_007

.T1, Maole_016700.T1, Maole_016854.T1, Maole_017223.T1, Maole_018863.T1, Maole_018

.T1

.T1, Maole_015137.T1, Maole_015653.T1, Maole_015844.T1, Maole_016230.T1, Maole_016

.T1, Maole_008706.T1, Maole_008707.T1, Maole_008737.T1, Maole_008842.T1, Maole_008

.T1, Maole_012375.T1, Maole_012507.T1, Maole_013413.T1, Maole_013571.T1, Maole_013
.T1, Maole_016735.T1, Maole_016763.T1, Maole_017197.T1, Maole_018679.T1, Maole_020

.T1, Maole_010293.T1, Maole_010480.T1, Maole_011813.T1, Maole_011930.T1, Maole_012

.T1, Maole_011051.T1, Maole_011052.T1, Maole_011198.T1, Maole_012180.T1, Maole_012

.T1, Maole_023452.T1, Maole_023453.T1
.T1, Maole_020346.T1, Maole_021033.T1, Maole_022368.T1, Maole_022371.T1, Maole_022

.T1, Maole_006169.T1, Maole_006214.T1, Maole_006532.T1, Maole_006581.T1, Maole_006
.T1, Maole_007209.T1, Maole_007211.T1, Maole_007214.T1, Maole_009104.T1, Maole_009

.T1, Maole_019211.T1, Maole_019276.T1, Maole_020748.T1, Maole_021963.T1, Maole_023

.T1, Maole_007206.T1, Maole_007208.T1, Maole_007209.T1, Maole_007211.T1, Maole_007

.T1, Maole_021963.T1, Maole_023134.T1
.T1, Maole_007206.T1, Maole_007208.T1, Maole_007209.T1, Maole_007211.T1, Maole_007
.T1, Maole_014267.T1, Maole_014269.T1, Maole_014270.T1, Maole_014272.T1, Maole_014

.T1, Maole_022510.T1, Maole_022511.T1, Maole_022512.T1, Maole_022513.T1, Maole_023
.T1, Maole_011159.T1, Maole_011288.T1, Maole_011357.T1, Maole_011358.T1, Maole_011
.T1, Maole_022513.T1, Maole_023259.T1, Maole_023260.T1, Maole_023776.T1, Maole_023
.T1, Maole_008212.T1, Maole_009317.T1, Maole_009320.T1, Maole_009465.T1, Maole_010

.T1, Maole_023260.T1, Maole_023776.T1, Maole_023917.T1, Maole_024056.T1, Maole_024
.T1, Maole_023260.T1, Maole_023776.T1, Maole_023917.T1, Maole_024056.T1, Maole_024

.T1, Maole_017647.T1, Maole_019130.T1, Maole_019618.T1, Maole_020501.T1, Maole_020
.T1, Maole_022508.T1, Maole_022510.T1, Maole_022511.T1, Maole_022512.T1, Maole_022

.T1, Maole_021416.T1, Maole_022500.T1, Maole_022503.T1, Maole_022504.T1, Maole_022

.T1, Maole_023260.T1, Maole_023776.T1, Maole_023917.T1, Maole_024056.T1, Maole_024

.T1, Maole_023452.T1, Maole_023453.T1

.T1, Maole_024051.T1, Maole_024069.T1, Maole_024098.T1
.T1, Maole_022507.T1, Maole_022508.T1, Maole_022510.T1, Maole_022511.T1, Maole_022

.T1, Maole_014744.T1, Maole_014847.T1, Maole_016455.T1, Maole_016662.T1, Maole_017

.T1, Maole_019106.T1, Maole_019211.T1, Maole_019276.T1, Maole_019618.T1, Maole_020

105.T1

210.T1, Maole_005341.T1, Maole_005416.T1, Maole_005495.T1, Maole_005619.T1, Maole_

974.T1, Maole_024105.T1
117.T1, Maole_020119.T1, Maole_020346.T1, Maole_021033.T1, Maole_022368.T1, Maole_

179.T1, Maole_010180.T1, Maole_010181.T1, Maole_010184.T1, Maole_010531.T1, Maole_

692.T1, Maole_019473.T1, Maole_019956.T1, Maole_019959.T1, Maole_019963.T1, Maole_
955.T1, Maole_017956.T1, Maole_018022.T1, Maole_019065.T1, Maole_019067.T1, Maole_
201.T1, Maole_007202.T1, Maole_007203.T1, Maole_007204.T1, Maole_007205.T1, Maole_
418.T1, Maole_020448.T1, Maole_020658.T1, Maole_020839.T1, Maole_021244.T1, Maole_

054.T1, Maole_007081.T1, Maole_007182.T1, Maole_007191.T1, Maole_007193.T1, Maole_
425.T1, Maole_013426.T1, Maole_013428.T1, Maole_013627.T1, Maole_014489.T1, Maole_

963.T1, Maole_023134.T1

967.T1, Maole_004968.T1, Maole_004969.T1, Maole_005107.T1, Maole_005145.T1, Maole_

753.T1, Maole_005120.T1, Maole_005178.T1, Maole_005179.T1, Maole_005180.T1, Maole_

206.T1, Maole_007208.T1, Maole_007209.T1, Maole_007211.T1, Maole_007214.T1, Maole_

531.T1, Maole_019748.T1, Maole_019749.T1, Maole_019750.T1, Maole_019753.T1, Maole_

994.T1, Maole_014003.T1, Maole_014489.T1, Maole_014681.T1, Maole_015503.T1, Maole_

999.T1, Maole_017643.T1, Maole_020337.T1, Maole_020346.T1, Maole_021033.T1, Maole_

963.T1, Maole_023024.T1, Maole_023134.T1, Maole_023400.T1, Maole_023402.T1, Maole_

123.T1, Maole_001124.T1, Maole_001125.T1, Maole_001136.T1, Maole_001137.T1, Maole_
037.T1, Maole_001096.T1, Maole_001097.T1, Maole_001108.T1, Maole_001120.T1, Maole_
879.T1, Maole_001904.T1, Maole_001931.T1, Maole_001948.T1, Maole_001957.T1, Maole_
779.T1, Maole_002780.T1, Maole_002782.T1, Maole_002792.T1, Maole_002950.T1, Maole_
277.T1, Maole_001301.T1, Maole_001375.T1, Maole_001380.T1, Maole_001467.T1, Maole_
905.T1, Maole_003994.T1, Maole_004229.T1, Maole_004238.T1, Maole_004246.T1, Maole_
432.T1, Maole_004459.T1, Maole_004600.T1, Maole_004798.T1, Maole_004878.T1, Maole_
406.T1, Maole_004432.T1, Maole_004459.T1, Maole_004600.T1, Maole_004798.T1, Maole_
459.T1, Maole_004600.T1, Maole_004798.T1, Maole_004878.T1, Maole_004972.T1, Maole_
459.T1, Maole_004600.T1, Maole_004798.T1, Maole_004878.T1, Maole_004972.T1, Maole_
432.T1, Maole_004459.T1, Maole_004600.T1, Maole_004798.T1, Maole_004878.T1, Maole_
191.T1, Maole_002221.T1, Maole_002239.T1, Maole_002276.T1, Maole_002401.T1, Maole_
475.T1, Maole_003525.T1, Maole_003557.T1, Maole_003560.T1, Maole_003905.T1, Maole_
056.T1, Maole_009267.T1, Maole_009268.T1, Maole_009440.T1, Maole_009441.T1, Maole_
477.T1, Maole_014480.T1, Maole_014484.T1, Maole_014487.T1, Maole_014488.T1, Maole_
010.T1, Maole_016575.T1, Maole_016576.T1, Maole_016686.T1, Maole_016696.T1, Maole_
487.T1, Maole_014488.T1, Maole_014602.T1, Maole_014730.T1, Maole_014835.T1, Maole_
582.T1, Maole_004697.T1, Maole_005074.T1, Maole_005102.T1, Maole_005293.T1, Maole_
518.T1, Maole_005877.T1, Maole_005880.T1, Maole_006036.T1, Maole_006194.T1, Maole_
605.T1, Maole_018866.T1, Maole_019404.T1, Maole_019405.T1, Maole_019406.T1, Maole_
718.T1, Maole_006799.T1, Maole_007035.T1, Maole_007183.T1, Maole_007285.T1, Maole_
253.T1, Maole_016657.T1, Maole_016686.T1, Maole_017163.T1, Maole_017245.T1, Maole_
631.T1, Maole_007690.T1, Maole_007855.T1, Maole_008292.T1, Maole_008616.T1, Maole_
351.T1, Maole_020655.T1, Maole_020968.T1, Maole_021008.T1, Maole_021164.T1, Maole_
776.T1, Maole_024121.T1
987.T1, Maole_004072.T1, Maole_004500.T1, Maole_004598.T1, Maole_004627.T1, Maole_
616.T1, Maole_011867.T1, Maole_011873.T1, Maole_012819.T1, Maole_013147.T1, Maole_
408.T1, Maole_013579.T1, Maole_013870.T1, Maole_014466.T1, Maole_014475.T1, Maole_
276.T1, Maole_005432.T1, Maole_005461.T1, Maole_005492.T1, Maole_005625.T1, Maole_
707.T1, Maole_009038.T1, Maole_009446.T1, Maole_009754.T1, Maole_009796.T1, Maole_
933.T1, Maole_007285.T1, Maole_007473.T1, Maole_007631.T1, Maole_007656.T1, Maole_
145.T1, Maole_007148.T1, Maole_007236.T1, Maole_007366.T1, Maole_007391.T1, Maole_

335.T1, Maole_014339.T1, Maole_014435.T1, Maole_014603.T1, Maole_014847.T1, Maole_

573.T1, Maole_023564.T1, Maole_023753.T1

418.T1, Maole_023564.T1, Maole_023753.T1, Maole_023776.T1, Maole_024121.T1
145.T1, Maole_007236.T1, Maole_007360.T1, Maole_007723.T1, Maole_007853.T1, Maole_

971.T1, Maole_002067.T1, Maole_002202.T1, Maole_002221.T1, Maole_002381.T1, Maole_
109.T1, Maole_018059.T1, Maole_019303.T1, Maole_019946.T1, Maole_020206.T1, Maole_

863.T1, Maole_014088.T1, Maole_014228.T1, Maole_014314.T1, Maole_014685.T1, Maole_

819.T1, Maole_013561.T1, Maole_013596.T1, Maole_013780.T1, Maole_014744.T1, Maole_

649.T1, Maole_012661.T1, Maole_012840.T1, Maole_013061.T1, Maole_013497.T1, Maole_

375.T1, Maole_013121.T1, Maole_013153.T1, Maole_013889.T1, Maole_014228.T1, Maole_

719.T1, Maole_020499.T1, Maole_021718.T1, Maole_021969.T1, Maole_021993.T1, Maole_

868.T1, Maole_009337.T1, Maole_009364.T1, Maole_009466.T1, Maole_009598.T1, Maole_
427.T1, Maole_019764.T1, Maole_019766.T1, Maole_019767.T1, Maole_020187.T1, Maole_

559.T1, Maole_007108.T1, Maole_007272.T1, Maole_007322.T1, Maole_007323.T1, Maole_

970.T1, Maole_017133.T1, Maole_017395.T1, Maole_017679.T1, Maole_018026.T1, Maole_

994.T1, Maole_011085.T1, Maole_012324.T1, Maole_012531.T1, Maole_012627.T1, Maole_

727.T1, Maole_023235.T1, Maole_023419.T1, Maole_024085.T1

489.T1, Maole_008505.T1, Maole_008713.T1, Maole_008736.T1, Maole_008763.T1, Maole_
107.T1, Maole_020684.T1, Maole_020797.T1, Maole_020940.T1, Maole_021069.T1, Maole_
354.T1, Maole_016104.T1, Maole_016169.T1, Maole_016226.T1, Maole_017280.T1, Maole_

088.T1, Maole_012158.T1, Maole_012507.T1, Maole_012672.T1, Maole_012878.T1, Maole_

118.T1, Maole_019137.T1, Maole_019939.T1, Maole_020683.T1, Maole_020807.T1, Maole_

909.T1, Maole_023245.T1, Maole_023761.T1

137.T1, Maole_015543.T1, Maole_015653.T1, Maole_017595.T1, Maole_017660.T1, Maole_

143.T1, Maole_008465.T1, Maole_008523.T1, Maole_008706.T1, Maole_008800.T1, Maole_

432.T1, Maole_017922.T1, Maole_018021.T1, Maole_018513.T1, Maole_019476.T1, Maole_

595.T1, Maole_017673.T1, Maole_019177.T1, Maole_019223.T1, Maole_019227.T1, Maole_

063.T1, Maole_021425.T1, Maole_021727.T1, Maole_022720.T1, Maole_023419.T1, Maole_
873.T1, Maole_012162.T1, Maole_012809.T1, Maole_013174.T1, Maole_013332.T1, Maole_

226.T1, Maole_016505.T1, Maole_016506.T1, Maole_016521.T1, Maole_016525.T1, Maole_
407.T1, Maole_021892.T1, Maole_022414.T1, Maole_022628.T1, Maole_023245.T1

607.T1, Maole_013763.T1, Maole_014370.T1, Maole_014963.T1, Maole_014964.T1, Maole_

463.T1, Maole_018465.T1, Maole_018928.T1, Maole_018929.T1, Maole_018931.T1, Maole_

455.T1, Maole_017135.T1, Maole_017383.T1, Maole_017544.T1, Maole_017861.T1, Maole_

368.T1, Maole_020370.T1, Maole_021407.T1, Maole_021892.T1, Maole_021969.T1, Maole_

713.T1, Maole_017861.T1, Maole_017911.T1, Maole_017922.T1, Maole_018676.T1, Maole_

644.T1, Maole_017911.T1, Maole_019118.T1, Maole_019453.T1, Maole_019939.T1, Maole_

620.T1, Maole_002680.T1, Maole_002693.T1, Maole_002747.T1, Maole_002761.T1, Maole_

513.T1, Maole_021008.T1, Maole_021164.T1, Maole_021289.T1, Maole_021942.T1, Maole_

737.T1, Maole_008809.T1, Maole_009064.T1, Maole_009360.T1, Maole_009636.T1, Maole_

627.T1, Maole_019629.T1, Maole_019630.T1, Maole_020612.T1, Maole_020860.T1, Maole_

467.T1, Maole_012666.T1, Maole_012805.T1, Maole_013299.T1, Maole_014042.T1, Maole_

244.T1, Maole_007826.T1, Maole_008299.T1, Maole_008615.T1, Maole_008839.T1, Maole_

864.T1, Maole_019146.T1, Maole_019391.T1, Maole_019590.T1, Maole_019762.T1, Maole_

242.T1, Maole_016245.T1, Maole_017595.T1, Maole_017718.T1, Maole_017770.T1, Maole_

854.T1, Maole_009289.T1, Maole_009303.T1, Maole_009367.T1, Maole_009921.T1, Maole_

689.T1, Maole_014265.T1, Maole_014267.T1, Maole_014269.T1, Maole_014270.T1, Maole_
896.T1, Maole_020940.T1, Maole_021069.T1, Maole_021289.T1, Maole_022034.T1, Maole_

210.T1, Maole_012554.T1, Maole_012805.T1, Maole_013299.T1, Maole_013870.T1, Maole_

353.T1, Maole_012360.T1, Maole_012531.T1, Maole_012766.T1, Maole_012828.T1, Maole_

372.T1, Maole_022373.T1, Maole_023450.T1, Maole_023452.T1, Maole_023453.T1, Maole_

583.T1, Maole_006647.T1, Maole_006651.T1, Maole_006652.T1, Maole_006779.T1, Maole_
186.T1, Maole_009325.T1, Maole_009326.T1, Maole_010377.T1, Maole_010649.T1, Maole_

024.T1, Maole_023134.T1

214.T1, Maole_009104.T1, Maole_009186.T1, Maole_009325.T1, Maole_009326.T1, Maole_

214.T1, Maole_009104.T1, Maole_009186.T1, Maole_009325.T1, Maole_009326.T1, Maole_
274.T1, Maole_014275.T1, Maole_014430.T1, Maole_014431.T1, Maole_014744.T1, Maole_

259.T1, Maole_023260.T1, Maole_023776.T1, Maole_023917.T1, Maole_024056.T1, Maole_
401.T1, Maole_011524.T1, Maole_011670.T1, Maole_012520.T1, Maole_012595.T1, Maole_
917.T1, Maole_024056.T1, Maole_024121.T1
105.T1, Maole_010200.T1, Maole_010203.T1, Maole_010336.T1, Maole_011110.T1, Maole_

121.T1
121.T1

502.T1, Maole_022100.T1
513.T1, Maole_023259.T1, Maole_023260.T1, Maole_023776.T1, Maole_023917.T1, Maole_

507.T1, Maole_022508.T1, Maole_022510.T1, Maole_022511.T1, Maole_022512.T1, Maole_

121.T1

512.T1, Maole_023385.T1, Maole_023917.T1, Maole_024121.T1

309.T1, Maole_017644.T1, Maole_017673.T1, Maole_019117.T1, Maole_020721.T1, Maole_

748.T1, Maole_021963.T1, Maole_023024.T1, Maole_023134.T1

_005691.T1, Maole_005696.T1, Maole_005759.T1, Maole_005836.T1, Maole_005991.T1, Ma

_022371.T1, Maole_022372.T1, Maole_022373.T1, Maole_023450.T1, Maole_023452.T1, Ma

_011036.T1, Maole_011061.T1, Maole_011062.T1, Maole_011817.T1, Maole_012089.T1, Ma

_020026.T1, Maole_020114.T1, Maole_020115.T1, Maole_020117.T1, Maole_020119.T1, Ma
_021248.T1, Maole_021253.T1, Maole_021341.T1, Maole_021342.T1, Maole_021438.T1, Ma
_007206.T1, Maole_007208.T1, Maole_007209.T1, Maole_007211.T1, Maole_007214.T1, Ma
_021291.T1, Maole_021626.T1, Maole_021627.T1, Maole_021897.T1, Maole_022097.T1, Ma

_007194.T1, Maole_007195.T1, Maole_007196.T1, Maole_007198.T1, Maole_007199.T1, Ma
_014581.T1, Maole_014582.T1, Maole_014612.T1, Maole_015115.T1, Maole_015424.T1, Ma

_005210.T1, Maole_005238.T1, Maole_005341.T1, Maole_005631.T1, Maole_005649.T1, Ma

_005495.T1, Maole_005694.T1, Maole_005695.T1, Maole_005698.T1, Maole_005700.T1, Ma

_007563.T1, Maole_007837.T1, Maole_008287.T1, Maole_008288.T1, Maole_009104.T1, Ma

_019754.T1, Maole_019755.T1, Maole_019756.T1, Maole_019757.T1, Maole_021234.T1, Ma

_016630.T1, Maole_019618.T1, Maole_019956.T1, Maole_019959.T1, Maole_019963.T1, Ma

_022371.T1, Maole_022372.T1, Maole_022373.T1, Maole_023452.T1, Maole_023453.T1, Ma

_023778.T1, Maole_023780.T1, Maole_023782.T1, Maole_024102.T1

_001216.T1, Maole_001240.T1, Maole_001277.T1, Maole_001301.T1, Maole_001360.T1, Ma
_001136.T1, Maole_001227.T1, Maole_001277.T1, Maole_001301.T1, Maole_001317.T1, Ma
_002056.T1, Maole_002191.T1, Maole_002221.T1, Maole_002239.T1, Maole_002288.T1, Ma
_003283.T1, Maole_003309.T1, Maole_003326.T1, Maole_003475.T1, Maole_003525.T1, Ma
_001469.T1, Maole_001485.T1, Maole_001488.T1, Maole_001545.T1, Maole_001561.T1, Ma
_004406.T1, Maole_004432.T1, Maole_004459.T1, Maole_004600.T1, Maole_004798.T1, Ma
_004972.T1, Maole_005013.T1, Maole_005326.T1, Maole_005478.T1, Maole_005492.T1, Ma
_004878.T1, Maole_004972.T1, Maole_005013.T1, Maole_005326.T1, Maole_005478.T1, Ma
_005013.T1, Maole_005326.T1, Maole_005478.T1, Maole_005492.T1, Maole_005520.T1, Ma
_005013.T1, Maole_005326.T1, Maole_005478.T1, Maole_005492.T1, Maole_005520.T1, Ma
_004972.T1, Maole_005013.T1, Maole_005326.T1, Maole_005329.T1, Maole_005478.T1, Ma
_002411.T1, Maole_002414.T1, Maole_002456.T1, Maole_002469.T1, Maole_002477.T1, Ma
_003994.T1, Maole_004229.T1, Maole_004246.T1, Maole_004406.T1, Maole_004432.T1, Ma
_010083.T1, Maole_010120.T1, Maole_010148.T1, Maole_010338.T1, Maole_010339.T1, Ma
_014490.T1, Maole_014602.T1, Maole_014641.T1, Maole_014963.T1, Maole_014964.T1, Ma
_017086.T1, Maole_017713.T1, Maole_018524.T1, Maole_019968.T1, Maole_020423.T1, Ma
_015354.T1, Maole_015543.T1, Maole_015873.T1, Maole_016011.T1, Maole_016032.T1, Ma
_005461.T1, Maole_005483.T1, Maole_005675.T1, Maole_005807.T1, Maole_005814.T1, Ma
_006247.T1, Maole_006351.T1, Maole_006581.T1, Maole_006583.T1, Maole_006647.T1, Ma
_019407.T1, Maole_019408.T1, Maole_019409.T1, Maole_019410.T1, Maole_019412.T1, Ma
_007427.T1, Maole_007690.T1, Maole_007855.T1, Maole_008706.T1, Maole_008707.T1, Ma
_017309.T1, Maole_018059.T1, Maole_019300.T1, Maole_019968.T1, Maole_020423.T1, Ma
_009446.T1, Maole_009754.T1, Maole_010016.T1, Maole_010934.T1, Maole_011155.T1, Ma
_021679.T1, Maole_022452.T1, Maole_022600.T1, Maole_022866.T1, Maole_022906.T1, Ma

_004696.T1, Maole_004819.T1, Maole_005074.T1, Maole_005232.T1, Maole_005237.T1, Ma
_013149.T1, Maole_014931.T1, Maole_015423.T1, Maole_015591.T1, Maole_015794.T1, Ma
_014477.T1, Maole_014487.T1, Maole_014488.T1, Maole_014602.T1, Maole_014641.T1, Ma
_005626.T1, Maole_005709.T1, Maole_005908.T1, Maole_005930.T1, Maole_006066.T1, Ma
_010934.T1, Maole_012088.T1, Maole_012144.T1, Maole_012145.T1, Maole_012180.T1, Ma
_008447.T1, Maole_008616.T1, Maole_008706.T1, Maole_008707.T1, Maole_008713.T1, Ma
_008382.T1, Maole_009064.T1, Maole_009303.T1, Maole_009317.T1, Maole_009320.T1, Ma

_015027.T1, Maole_015032.T1, Maole_015140.T1, Maole_015653.T1, Maole_015659.T1, Ma

_007944.T1, Maole_008489.T1, Maole_008713.T1, Maole_008763.T1, Maole_008800.T1, Ma

_002389.T1, Maole_002408.T1, Maole_002505.T1, Maole_002516.T1, Maole_002627.T1, Ma
_020826.T1, Maole_022244.T1, Maole_022628.T1, Maole_022842.T1, Maole_022844.T1

_015236.T1, Maole_016012.T1, Maole_016217.T1, Maole_016473.T1, Maole_017043.T1, Ma

_014931.T1, Maole_015061.T1, Maole_015591.T1, Maole_015794.T1, Maole_015802.T1, Ma

_013520.T1, Maole_013763.T1, Maole_014128.T1, Maole_014236.T1, Maole_014314.T1, Ma

_015061.T1, Maole_016169.T1, Maole_016473.T1, Maole_016696.T1, Maole_016770.T1, Ma

_023030.T1, Maole_023753.T1

_010293.T1, Maole_010480.T1, Maole_011381.T1, Maole_011507.T1, Maole_011678.T1, Ma
_022866.T1, Maole_023468.T1, Maole_023479.T1, Maole_023519.T1, Maole_023523.T1

_007462.T1, Maole_007944.T1, Maole_008053.T1, Maole_008143.T1, Maole_008401.T1, Ma

_018862.T1, Maole_019509.T1, Maole_019580.T1, Maole_019951.T1, Maole_020103.T1, Ma

_012819.T1, Maole_012828.T1, Maole_013216.T1, Maole_013515.T1, Maole_013607.T1, Ma

_009053.T1, Maole_009054.T1, Maole_009056.T1, Maole_009114.T1, Maole_009181.T1, Ma
_022720.T1, Maole_023469.T1, Maole_023840.T1
_017309.T1, Maole_017383.T1, Maole_017415.T1, Maole_017713.T1, Maole_017809.T1, Ma

_013112.T1, Maole_014580.T1, Maole_014602.T1, Maole_014641.T1, Maole_015407.T1, Ma

_021614.T1, Maole_021727.T1, Maole_022187.T1, Maole_022367.T1, Maole_022898.T1, Ma

_017900.T1, Maole_018160.T1, Maole_019070.T1, Maole_019165.T1, Maole_019320.T1, Ma

_009064.T1, Maole_009432.T1, Maole_009465.T1, Maole_009636.T1, Maole_010277.T1, Ma

_020878.T1, Maole_021900.T1, Maole_022906.T1, Maole_023447.T1, Maole_023776.T1

_019303.T1, Maole_019453.T1, Maole_019901.T1, Maole_020823.T1, Maole_020830.T1, Ma

_024085.T1
_013709.T1, Maole_014199.T1, Maole_014397.T1, Maole_014555.T1, Maole_016102.T1, Ma

_017222.T1, Maole_017647.T1, Maole_018073.T1, Maole_019130.T1, Maole_020589.T1, Ma

_016393.T1, Maole_016696.T1, Maole_016881.T1, Maole_017197.T1, Maole_017544.T1, Ma

_018932.T1, Maole_019031.T1, Maole_019219.T1, Maole_019435.T1, Maole_019627.T1, Ma

_017911.T1, Maole_019118.T1, Maole_020558.T1, Maole_021494.T1, Maole_022367.T1, Ma

_022347.T1, Maole_022350.T1, Maole_022414.T1, Maole_022678.T1, Maole_023030.T1

_018847.T1, Maole_018869.T1, Maole_019137.T1, Maole_019399.T1, Maole_019453.T1, Ma

_020558.T1, Maole_021573.T1, Maole_021614.T1, Maole_021727.T1, Maole_022367.T1, Ma

_002818.T1, Maole_002826.T1, Maole_002955.T1, Maole_003259.T1, Maole_003261.T1, Ma

_022906.T1, Maole_023290.T1, Maole_023612.T1, Maole_023762.T1, Maole_023849.T1

_009817.T1, Maole_010337.T1, Maole_010425.T1, Maole_010934.T1, Maole_011377.T1, Ma

_021407.T1, Maole_021422.T1, Maole_022350.T1, Maole_022352.T1, Maole_022678.T1, Ma

_014603.T1, Maole_015140.T1, Maole_015294.T1, Maole_015629.T1, Maole_015873.T1, Ma

_009038.T1, Maole_009320.T1, Maole_009446.T1, Maole_009992.T1, Maole_010016.T1, Ma

_019764.T1, Maole_019766.T1, Maole_019767.T1, Maole_021014.T1, Maole_021015.T1, Ma

_018140.T1, Maole_018625.T1, Maole_018627.T1, Maole_018869.T1, Maole_019045.T1, Ma

_010021.T1, Maole_010098.T1, Maole_010336.T1, Maole_010457.T1, Maole_010661.T1, Ma

_014272.T1, Maole_014274.T1, Maole_014275.T1, Maole_014602.T1, Maole_014641.T1, Ma
_022497.T1, Maole_023466.T1

_013950.T1, Maole_014088.T1, Maole_014948.T1, Maole_015324.T1, Maole_015873.T1, Ma

_013339.T1, Maole_014579.T1, Maole_014831.T1, Maole_014832.T1, Maole_015610.T1, Ma

_023556.T1, Maole_023558.T1, Maole_023986.T1, Maole_023988.T1, Maole_024067.T1

_007827.T1, Maole_007944.T1, Maole_008212.T1, Maole_009317.T1, Maole_009320.T1, Ma
_011635.T1, Maole_012880.T1, Maole_013041.T1, Maole_013042.T1, Maole_013187.T1, Ma

_010377.T1, Maole_010649.T1, Maole_011635.T1, Maole_012880.T1, Maole_013041.T1, Ma

_010377.T1, Maole_010649.T1, Maole_011635.T1, Maole_012880.T1, Maole_013041.T1, Ma
_014883.T1, Maole_014887.T1, Maole_015116.T1, Maole_015166.T1, Maole_015167.T1, Ma

_024121.T1
_012596.T1, Maole_012598.T1, Maole_012798.T1, Maole_013561.T1, Maole_013780.T1, Ma

_011111.T1, Maole_011288.T1, Maole_011524.T1, Maole_011670.T1, Maole_012162.T1, Ma

_024056.T1, Maole_024121.T1

_022513.T1, Maole_023259.T1, Maole_023260.T1, Maole_023385.T1, Maole_023776.T1, Ma

021175.T1, Maole_021178.T1, Maole_021594.T1, Maole_022202.T1, Maole_022503.T1, Ma

ole_006013.T1, Maole_006014.T1, Maole_006015.T1, Maole_006016.T1, Maole_006017.T1,

ole_023453.T1, Maole_023556.T1, Maole_023558.T1, Maole_023986.T1, Maole_023988.T1,

ole_012412.T1, Maole_012510.T1, Maole_012895.T1, Maole_012897.T1, Maole_013491.T1,

ole_020579.T1, Maole_020900.T1, Maole_021600.T1, Maole_022440.T1, Maole_022503.T1,
ole_021896.T1, Maole_021898.T1, Maole_022379.T1, Maole_023511.T1, Maole_023736.T1,
ole_007441.T1, Maole_007523.T1, Maole_007563.T1, Maole_007837.T1, Maole_008287.T1,
ole_022164.T1, Maole_022328.T1, Maole_023960.T1

ole_007201.T1, Maole_007202.T1, Maole_007203.T1, Maole_007204.T1, Maole_007205.T1,
ole_015426.T1, Maole_015427.T1, Maole_015503.T1, Maole_018581.T1, Maole_018972.T1,

ole_005752.T1, Maole_005991.T1, Maole_006239.T1, Maole_006342.T1, Maole_006343.T1,

ole_005702.T1, Maole_005712.T1, Maole_005715.T1, Maole_006013.T1, Maole_006014.T1,

ole_009186.T1, Maole_009325.T1, Maole_009326.T1, Maole_010102.T1, Maole_010377.T1,

ole_021480.T1, Maole_021613.T1, Maole_022416.T1, Maole_022935.T1, Maole_023204.T1,

ole_023090.T1, Maole_023092.T1, Maole_023402.T1, Maole_023574.T1, Maole_023780.T1,

ole_023752.T1, Maole_024037.T1

ole_001375.T1, Maole_001403.T1, Maole_001404.T1, Maole_001419.T1, Maole_001420.T1,
ole_001360.T1, Maole_001375.T1, Maole_001390.T1, Maole_001394.T1, Maole_001403.T1,
ole_002304.T1, Maole_002326.T1, Maole_002401.T1, Maole_002406.T1, Maole_002461.T1,
ole_003560.T1, Maole_003905.T1, Maole_003994.T1, Maole_004047.T1, Maole_004229.T1,
ole_001631.T1, Maole_001649.T1, Maole_001703.T1, Maole_001730.T1, Maole_001757.T1,
ole_004878.T1, Maole_004972.T1, Maole_005013.T1, Maole_005326.T1, Maole_005388.T1,
ole_005520.T1, Maole_005622.T1, Maole_005818.T1, Maole_005898.T1, Maole_006040.T1,
ole_005492.T1, Maole_005520.T1, Maole_005622.T1, Maole_005818.T1, Maole_005898.T1,
ole_005622.T1, Maole_005818.T1, Maole_005898.T1, Maole_006040.T1, Maole_006561.T1,
ole_005622.T1, Maole_005818.T1, Maole_005898.T1, Maole_006040.T1, Maole_006561.T1,
ole_005492.T1, Maole_005520.T1, Maole_005622.T1, Maole_005818.T1, Maole_005898.T1,
ole_002620.T1, Maole_002627.T1, Maole_002652.T1, Maole_002680.T1, Maole_002750.T1,
ole_004457.T1, Maole_004548.T1, Maole_004600.T1, Maole_004602.T1, Maole_004878.T1,
ole_011670.T1, Maole_011832.T1, Maole_011872.T1, Maole_012061.T1, Maole_012114.T1,
ole_015044.T1, Maole_016011.T1, Maole_016363.T1, Maole_017094.T1, Maole_018026.T1,
ole_021494.T1, Maole_021905.T1, Maole_022120.T1, Maole_022202.T1, Maole_022732.T1,
ole_016410.T1, Maole_016970.T1, Maole_017395.T1, Maole_017660.T1, Maole_017679.T1,
ole_005908.T1, Maole_006036.T1, Maole_006194.T1, Maole_006351.T1, Maole_006468.T1,
ole_006830.T1, Maole_007024.T1, Maole_007108.T1, Maole_007145.T1, Maole_007294.T1,
ole_019413.T1, Maole_019415.T1, Maole_019416.T1, Maole_019417.T1, Maole_019422.T1,
ole_009317.T1, Maole_009320.T1, Maole_009446.T1, Maole_009754.T1, Maole_009796.T1,
ole_020854.T1, Maole_021285.T1, Maole_021425.T1, Maole_022100.T1, Maole_022732.T1,
ole_011161.T1, Maole_012088.T1, Maole_012144.T1, Maole_012145.T1, Maole_012180.T1,
ole_022983.T1, Maole_022984.T1, Maole_023194.T1, Maole_023612.T1

ole_005244.T1, Maole_005293.T1, Maole_005453.T1, Maole_005483.T1, Maole_005518.T1,
ole_016201.T1, Maole_016457.T1, Maole_016770.T1, Maole_017085.T1, Maole_017086.T1,
ole_014721.T1, Maole_015294.T1, Maole_015835.T1, Maole_015873.T1, Maole_016011.T1,
ole_006083.T1, Maole_006194.T1, Maole_006295.T1, Maole_006819.T1, Maole_006850.T1,
ole_012283.T1, Maole_012426.T1, Maole_012602.T1, Maole_012716.T1, Maole_012805.T1,
ole_009267.T1, Maole_009268.T1, Maole_009754.T1, Maole_010934.T1, Maole_011207.T1,
ole_009506.T1, Maole_009921.T1, Maole_010098.T1, Maole_010110.T1, Maole_010337.T1,

ole_015794.T1, Maole_015802.T1, Maole_015921.T1, Maole_016217.T1, Maole_016245.T1,

ole_009064.T1, Maole_009181.T1, Maole_009268.T1, Maole_009606.T1, Maole_009607.T1,

ole_002680.T1, Maole_002706.T1, Maole_002707.T1, Maole_002742.T1, Maole_002747.T1,

ole_017383.T1, Maole_017911.T1, Maole_018086.T1, Maole_019071.T1, Maole_019580.T1,

ole_016343.T1, Maole_016345.T1, Maole_016457.T1, Maole_016686.T1, Maole_017309.T1,

ole_014685.T1, Maole_014838.T1, Maole_014993.T1, Maole_015671.T1, Maole_015896.T1,

ole_017476.T1, Maole_019159.T1, Maole_020187.T1, Maole_020298.T1, Maole_020517.T1,

ole_011710.T1, Maole_012093.T1, Maole_012158.T1, Maole_012210.T1, Maole_012255.T1,

ole_008523.T1, Maole_008706.T1, Maole_008800.T1, Maole_008874.T1, Maole_008875.T1,

ole_020873.T1, Maole_020919.T1, Maole_021091.T1, Maole_021900.T1, Maole_022187.T1,

ole_013625.T1, Maole_013889.T1, Maole_014506.T1, Maole_014728.T1, Maole_014831.T1,

ole_009724.T1, Maole_009796.T1, Maole_010083.T1, Maole_010768.T1, Maole_011051.T1,

ole_017861.T1, Maole_018069.T1, Maole_018869.T1, Maole_019399.T1, Maole_019453.T1,

ole_015612.T1, Maole_016612.T1, Maole_016977.T1, Maole_016978.T1, Maole_017094.T1,

ole_023357.T1

ole_019439.T1, Maole_019947.T1, Maole_020238.T1, Maole_021441.T1, Maole_023802.T1,

ole_010308.T1, Maole_010389.T1, Maole_010601.T1, Maole_011059.T1, Maole_011211.T1,

ole_021285.T1, Maole_021452.T1, Maole_021844.T1, Maole_022244.T1, Maole_022842.T1,

ole_016253.T1, Maole_016541.T1, Maole_016542.T1, Maole_016855.T1, Maole_017163.T1,

ole_021202.T1, Maole_021821.T1, Maole_022779.T1, Maole_023248.T1

ole_017660.T1, Maole_017719.T1, Maole_017900.T1, Maole_017929.T1, Maole_019320.T1,

ole_019629.T1, Maole_019630.T1, Maole_020612.T1, Maole_020860.T1, Maole_021407.T1,

ole_022776.T1, Maole_023235.T1, Maole_023728.T1

ole_019520.T1, Maole_019799.T1, Maole_019929.T1, Maole_020198.T1, Maole_020436.T1,

ole_023235.T1

ole_003286.T1, Maole_003501.T1, Maole_003525.T1, Maole_003596.T1, Maole_003597.T1,

ole_011378.T1, Maole_011411.T1, Maole_012144.T1, Maole_012145.T1, Maole_012649.T1,

ole_022820.T1, Maole_023409.T1, Maole_023931.T1, Maole_024025.T1

ole_016012.T1, Maole_016155.T1, Maole_016226.T1, Maole_016253.T1, Maole_016473.T1,

ole_010110.T1, Maole_010336.T1, Maole_010401.T1, Maole_010614.T1, Maole_010733.T1,

ole_021089.T1

ole_019046.T1, Maole_019047.T1, Maole_019320.T1, Maole_019399.T1, Maole_019764.T1,

ole_010662.T1, Maole_010832.T1, Maole_011411.T1, Maole_011519.T1, Maole_011885.T1,

ole_015116.T1, Maole_015407.T1, Maole_015659.T1, Maole_017094.T1, Maole_017166.T1,

ole_016104.T1, Maole_016230.T1, Maole_016971.T1, Maole_017331.T1, Maole_017383.T1,

ole_016082.T1, Maole_016155.T1, Maole_016700.T1, Maole_016733.T1, Maole_016735.T1,

ole_009465.T1, Maole_010105.T1, Maole_010200.T1, Maole_010203.T1, Maole_010336.T1,
ole_013907.T1, Maole_013908.T1, Maole_013909.T1, Maole_013911.T1, Maole_013912.T1,

ole_013042.T1, Maole_013187.T1, Maole_013907.T1, Maole_013908.T1, Maole_013909.T1,

ole_013042.T1, Maole_013187.T1, Maole_013907.T1, Maole_013908.T1, Maole_013909.T1,
ole_015667.T1, Maole_015936.T1, Maole_016253.T1, Maole_016541.T1, Maole_016542.T1,

ole_014265.T1, Maole_014267.T1, Maole_014269.T1, Maole_014270.T1, Maole_014272.T1,

ole_012520.T1, Maole_012595.T1, Maole_012596.T1, Maole_012598.T1, Maole_013561.T1,

ole_023917.T1, Maole_024056.T1, Maole_024121.T1

ole_022508.T1, Maole_023259.T1, Maole_023260.T1, Maole_023776.T1, Maole_023917.T1,

Maole_006167.T1, Maole_006239.T1, Maole_006379.T1, Maole_006380.T1, Maol

Maole_024067.T1

Maole_013627.T1, Maole_013992.T1, Maole_013993.T1, Maole_013994.T1, Maol

Maole_022508.T1, Maole_023090.T1, Maole_023091.T1, Maole_023092.T1, Maol
Maole_023737.T1, Maole_023741.T1, Maole_023742.T1
Maole_008288.T1, Maole_008519.T1, Maole_008609.T1, Maole_008931.T1, Maol

Maole_007206.T1, Maole_007208.T1, Maole_007209.T1, Maole_007211.T1, Maol
Maole_018973.T1, Maole_018974.T1, Maole_019117.T1, Maole_020579.T1, Maol

Maole_006464.T1, Maole_006513.T1, Maole_006532.T1, Maole_006824.T1, Maol

Maole_006015.T1, Maole_006016.T1, Maole_006017.T1, Maole_006121.T1, Maol

Maole_010649.T1, Maole_011635.T1, Maole_012880.T1, Maole_013041.T1, Maol

Maole_023205.T1, Maole_023206.T1, Maole_023208.T1, Maole_023211.T1, Maol

Maole_023782.T1

Maole_001424.T1, Maole_001432.T1, Maole_001467.T1, Maole_001469.T1, Maol
Maole_001419.T1, Maole_001420.T1, Maole_001424.T1, Maole_001432.T1, Maol
Maole_002469.T1, Maole_002505.T1, Maole_002510.T1, Maole_002591.T1, Maol
Maole_004238.T1, Maole_004246.T1, Maole_004406.T1, Maole_004432.T1, Maol
Maole_001859.T1, Maole_001878.T1, Maole_001879.T1, Maole_001931.T1, Maol
Maole_005478.T1, Maole_005492.T1, Maole_005520.T1, Maole_005622.T1, Maol
Maole_006561.T1, Maole_006647.T1, Maole_006651.T1, Maole_006652.T1, Maol
Maole_006040.T1, Maole_006561.T1, Maole_006647.T1, Maole_006651.T1, Maol
Maole_006647.T1, Maole_006651.T1, Maole_006652.T1, Maole_006670.T1, Maol
Maole_006647.T1, Maole_006651.T1, Maole_006652.T1, Maole_006670.T1, Maol
Maole_006040.T1, Maole_006561.T1, Maole_006647.T1, Maole_006651.T1, Maol
Maole_002778.T1, Maole_002782.T1, Maole_002855.T1, Maole_002884.T1, Maol
Maole_004972.T1, Maole_005013.T1, Maole_005326.T1, Maole_005388.T1, Maol
Maole_012116.T1, Maole_012845.T1, Maole_013673.T1, Maole_013830.T1, Maol
Maole_018590.T1, Maole_018592.T1, Maole_018595.T1, Maole_018597.T1, Maol
Maole_022733.T1, Maole_022737.T1, Maole_022760.T1, Maole_023851.T1
Maole_017900.T1, Maole_017922.T1, Maole_018086.T1, Maole_018590.T1, Maol
Maole_006581.T1, Maole_006583.T1, Maole_006713.T1, Maole_006829.T1, Maol
Maole_007429.T1, Maole_007687.T1, Maole_007690.T1, Maole_007944.T1, Maol
Maole_019476.T1, Maole_020715.T1, Maole_022215.T1, Maole_022216.T1, Maol
Maole_010849.T1, Maole_010934.T1, Maole_011930.T1, Maole_012088.T1, Maol
Maole_022733.T1, Maole_022737.T1, Maole_023419.T1
Maole_012285.T1, Maole_012342.T1, Maole_012360.T1, Maole_012716.T1, Maol

Maole_005675.T1, Maole_006085.T1, Maole_006351.T1, Maole_006468.T1, Maol
Maole_017087.T1, Maole_017092.T1, Maole_017673.T1, Maole_018059.T1, Maol
Maole_016612.T1, Maole_016977.T1, Maole_016978.T1, Maole_017094.T1, Maol
Maole_007143.T1, Maole_007145.T1, Maole_007236.T1, Maole_007360.T1, Maol
Maole_013116.T1, Maole_013119.T1, Maole_013153.T1, Maole_013299.T1, Maol
Maole_011523.T1, Maole_011813.T1, Maole_011872.T1, Maole_011987.T1, Maol
Maole_010389.T1, Maole_011377.T1, Maole_011378.T1, Maole_011406.T1, Maol

Maole_016455.T1, Maole_016662.T1, Maole_017089.T1, Maole_017245.T1, Maol

Maole_009608.T1, Maole_009738.T1, Maole_010083.T1, Maole_010114.T1, Maol

Maole_002762.T1, Maole_002818.T1, Maole_003067.T1, Maole_003087.T1, Maol

Maole_020161.T1, Maole_020499.T1, Maole_020616.T1, Maole_021199.T1, Maol

Maole_017673.T1, Maole_018563.T1, Maole_018630.T1, Maole_018701.T1, Maol

Maole_016043.T1, Maole_016461.T1, Maole_016463.T1, Maole_016467.T1, Maol

Maole_020738.T1, Maole_021905.T1, Maole_022760.T1, Maole_023468.T1, Maol

Maole_013301.T1, Maole_013408.T1, Maole_013450.T1, Maole_013486.T1, Maol

Maole_008959.T1, Maole_009067.T1, Maole_009364.T1, Maole_009738.T1, Maol

Maole_023299.T1, Maole_023479.T1, Maole_023513.T1, Maole_023612.T1

Maole_014832.T1, Maole_015154.T1, Maole_015464.T1, Maole_015579.T1, Maol

Maole_011052.T1, Maole_011211.T1, Maole_011709.T1, Maole_011832.T1, Maol

Maole_020198.T1, Maole_020351.T1, Maole_020616.T1, Maole_020655.T1, Maol

Maole_017220.T1, Maole_017644.T1, Maole_018026.T1, Maole_018327.T1, Maol

Maole_024121.T1

Maole_011264.T1, Maole_011377.T1, Maole_011378.T1, Maole_013253.T1, Maol

Maole_022844.T1, Maole_023519.T1, Maole_023523.T1, Maole_024010.T1

Maole_017383.T1, Maole_017398.T1, Maole_017544.T1, Maole_017911.T1, Maol

Maole_019423.T1, Maole_020103.T1, Maole_021285.T1, Maole_021993.T1, Maol

Maole_021422.T1, Maole_022350.T1, Maole_022352.T1, Maole_022678.T1, Maol

Maole_020438.T1, Maole_020565.T1, Maole_020616.T1, Maole_020830.T1, Maol

Maole_003652.T1, Maole_003656.T1, Maole_003754.T1, Maole_003773.T1, Maol

Maole_013127.T1, Maole_013971.T1, Maole_014828.T1, Maole_014838.T1, Maol

Maole_016696.T1, Maole_017163.T1, Maole_017249.T1, Maole_017378.T1, Maol

Maole_011519.T1, Maole_012285.T1, Maole_012342.T1, Maole_012496.T1, Maol

Maole_019947.T1, Maole_020161.T1, Maole_020797.T1, Maole_020807.T1, Maol

Maole_011953.T1, Maole_012257.T1, Maole_012375.T1, Maole_012448.T1, Maol

Maole_017261.T1, Maole_017476.T1, Maole_017644.T1, Maole_017653.T1, Maol

Maole_017713.T1, Maole_017922.T1, Maole_018869.T1, Maole_018886.T1, Maol

Maole_017223.T1, Maole_018644.T1, Maole_018863.T1, Maole_018864.T1, Maol

Maole_010943.T1, Maole_010944.T1, Maole_010945.T1, Maole_010946.T1, Maol
Maole_013913.T1, Maole_013914.T1, Maole_014526.T1, Maole_015999.T1, Maol

Maole_013911.T1, Maole_013912.T1, Maole_013913.T1, Maole_013914.T1, Maol

Maole_013911.T1, Maole_013912.T1, Maole_013913.T1, Maole_013914.T1, Maol
Maole_016662.T1, Maole_016768.T1, Maole_017068.T1, Maole_017309.T1, Maol

Maole_014274.T1, Maole_014275.T1, Maole_014430.T1, Maole_014431.T1, Maol

Maole_014199.T1, Maole_014265.T1, Maole_014267.T1, Maole_014269.T1, Maol

Maole_024056.T1, Maole_024121.T1

Figure

Click here to access/download;Figure;Fig_S1.pdf

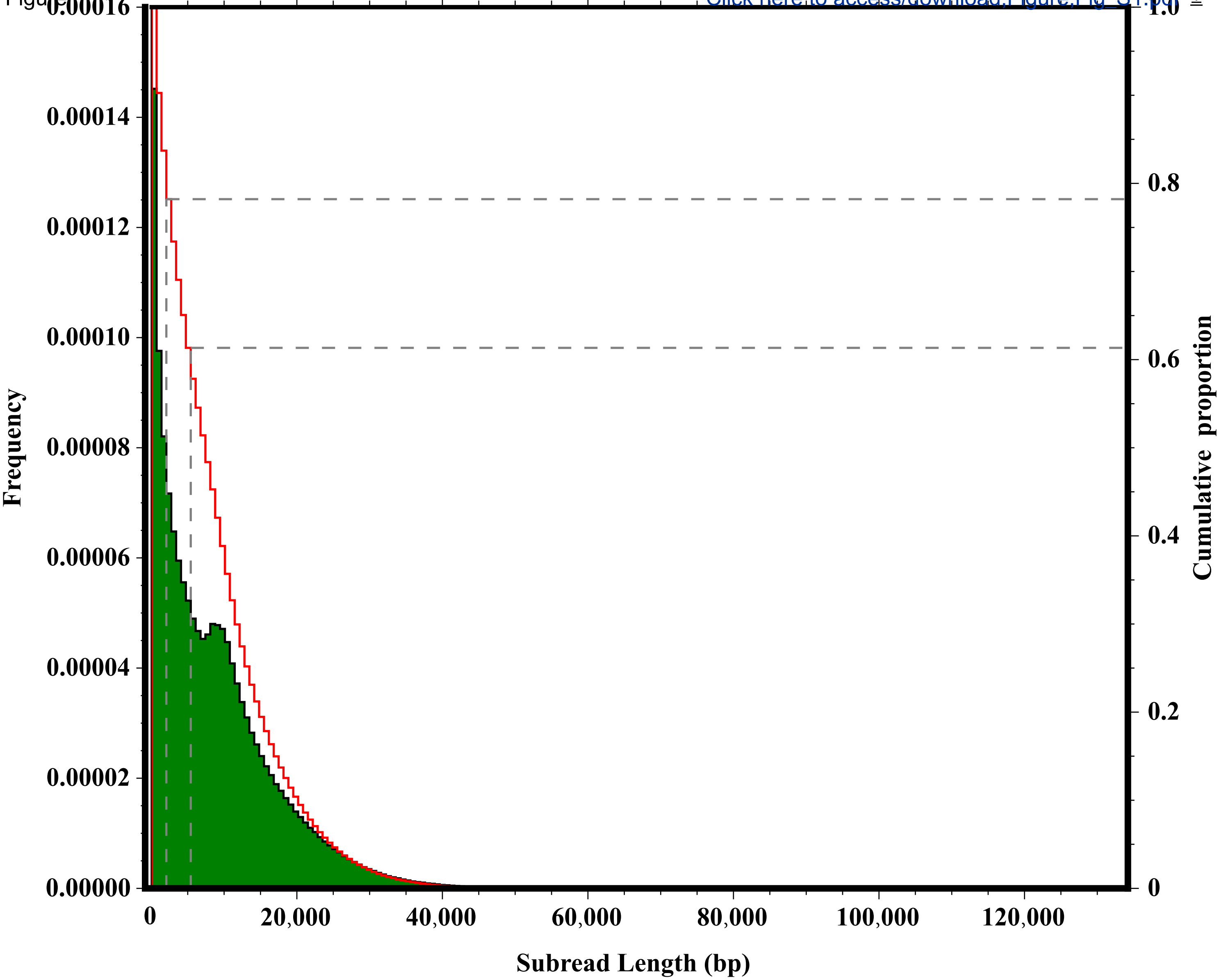

Figure
Click here to access/download;Figure;Fig_S2.pdf

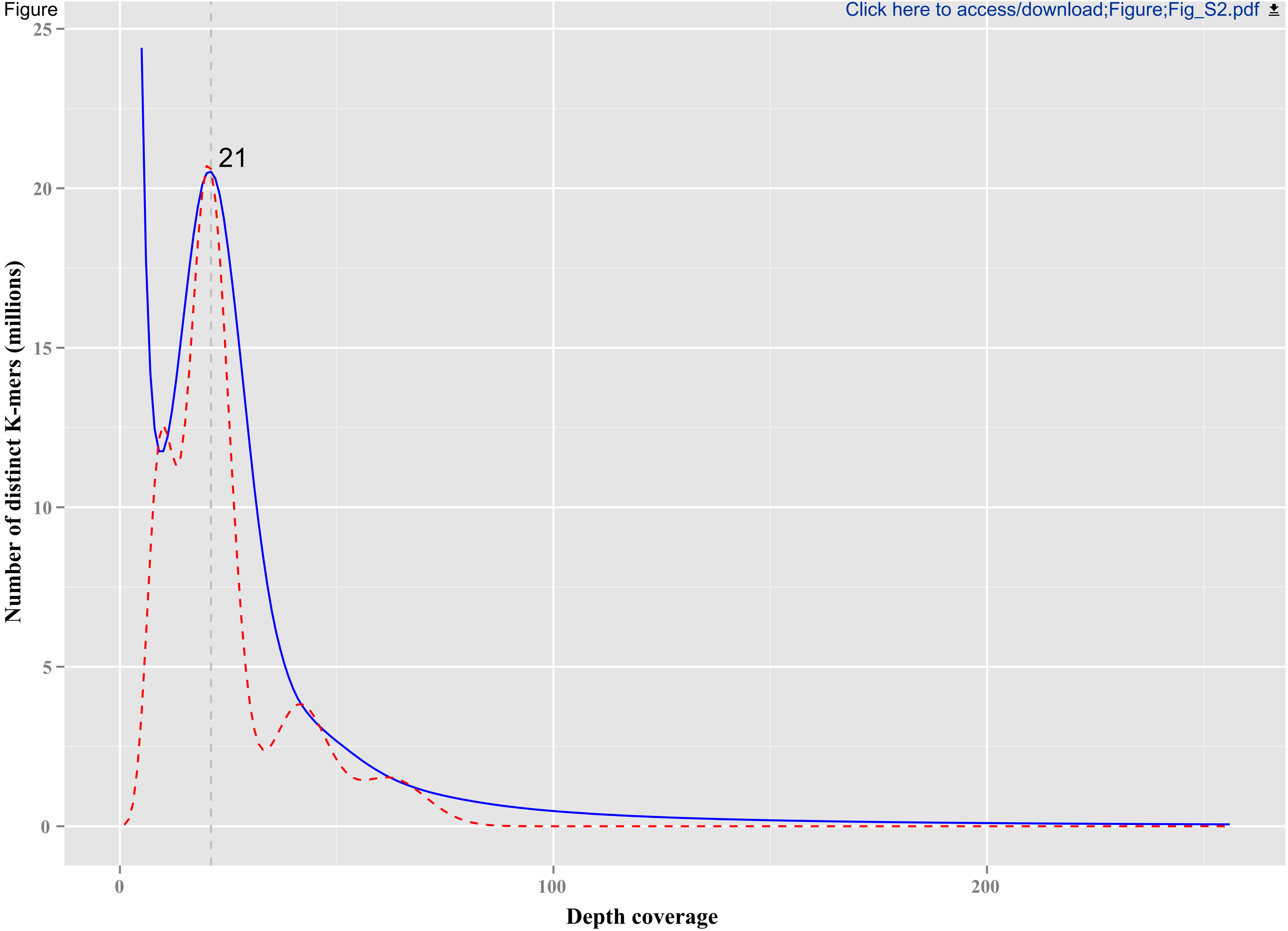

Figure Click here to access/download;Figure;Fig_S3.pdf

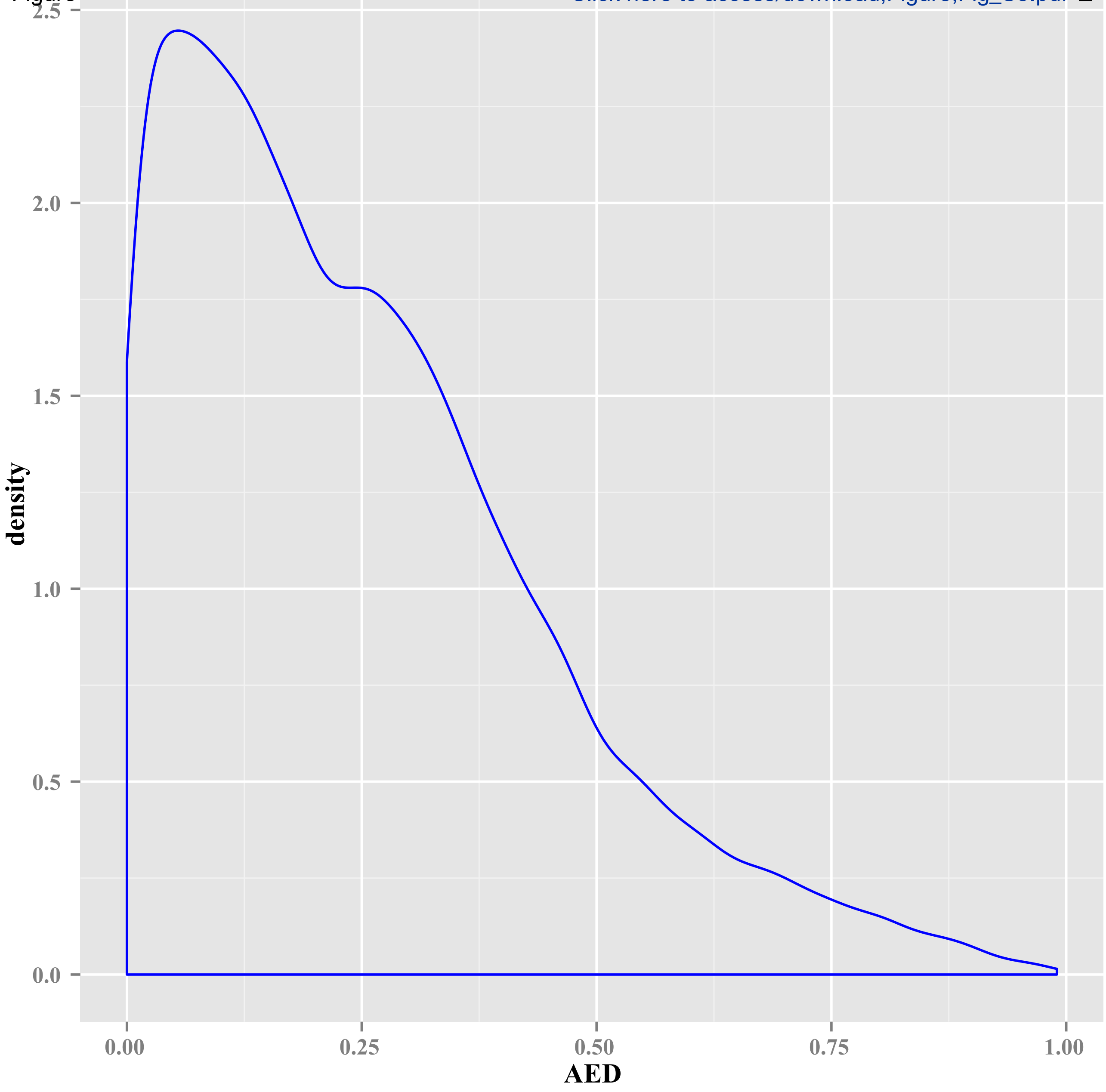

Figure
Click here to access/download;Figure;Fig_S4.pdf

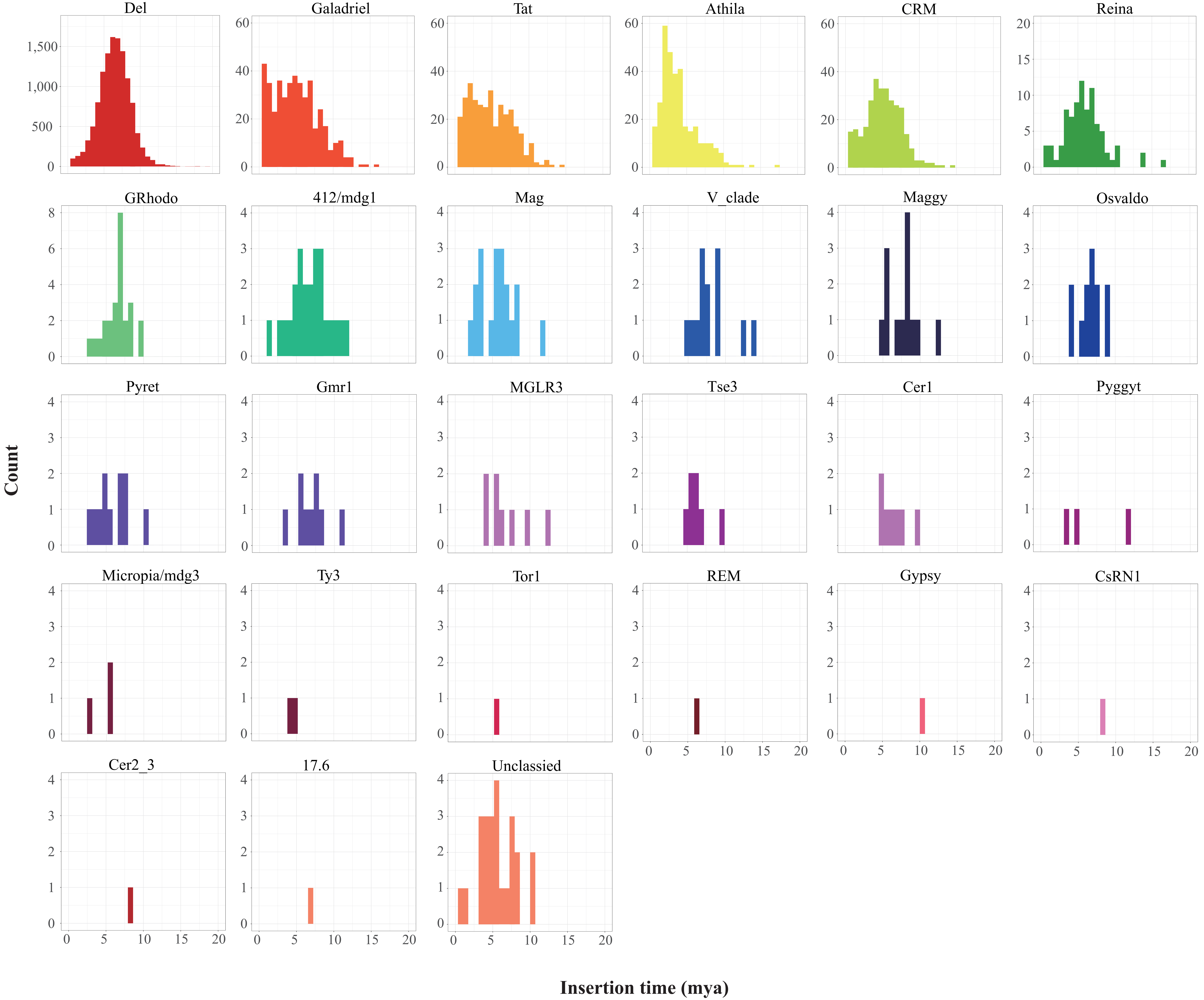

Figure

Click here to access/download;Figure;Fig_S5.pdf

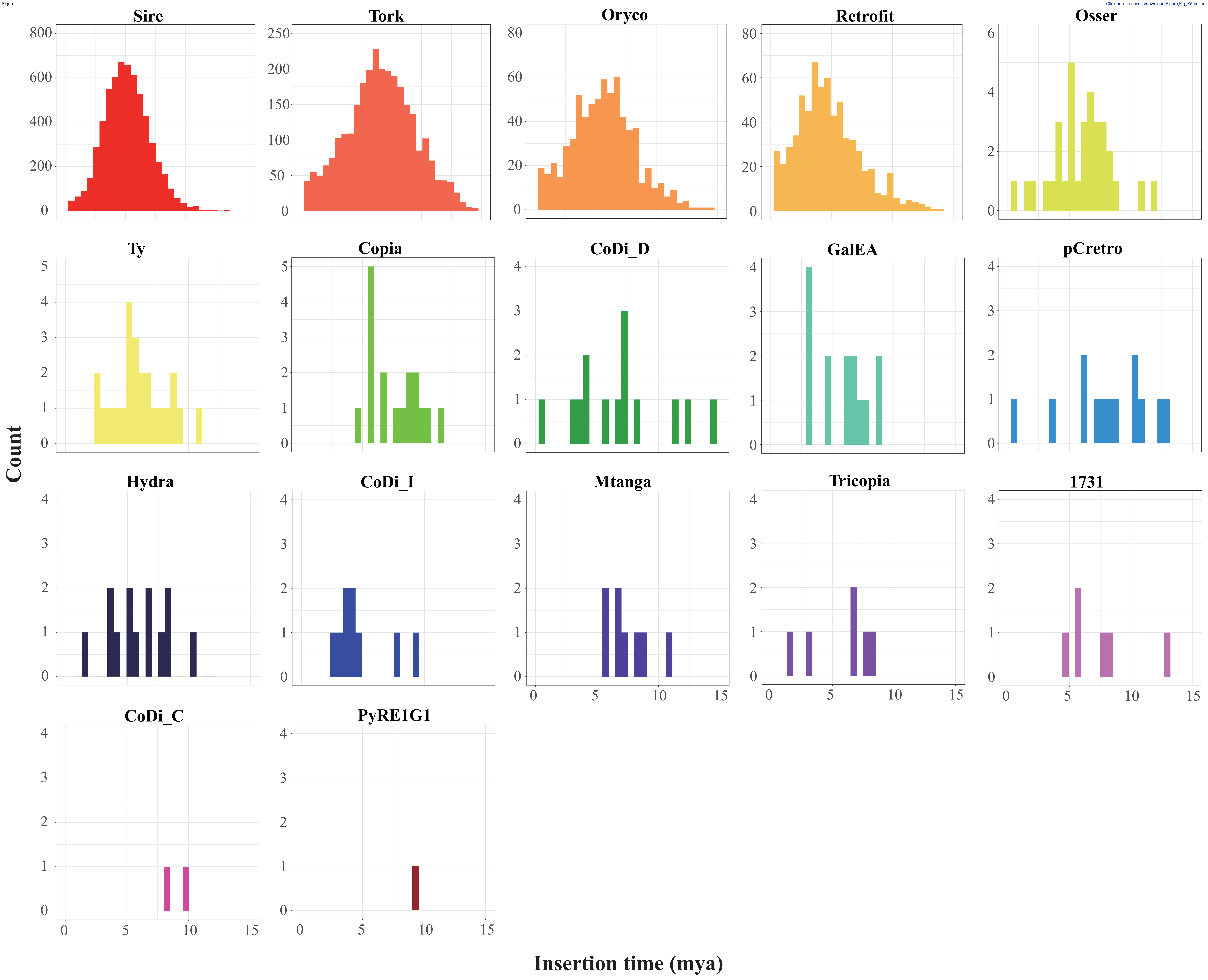

Figure

Click here to access/download;Figure;Fig_S6.pdf

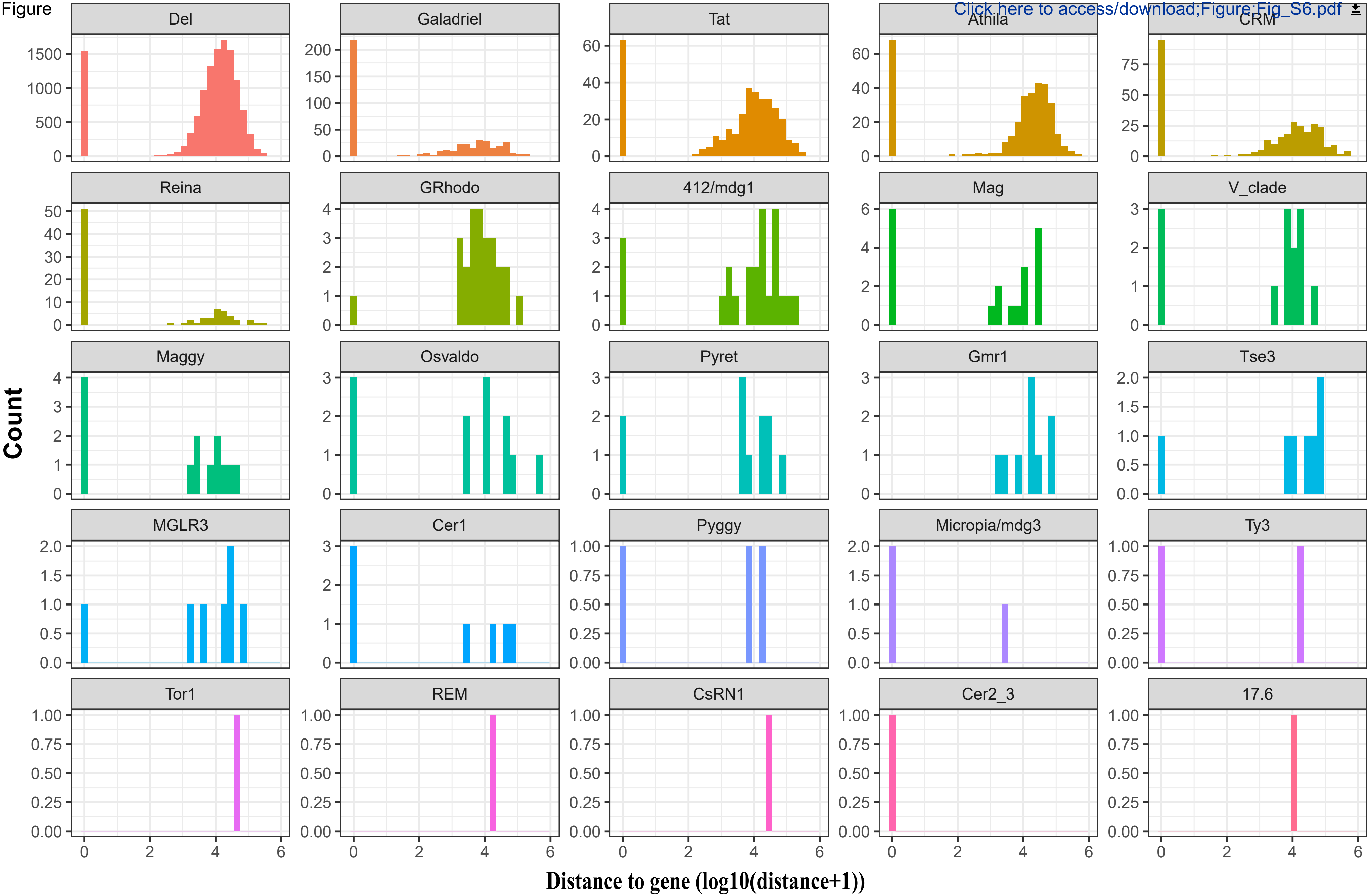

Figure

Click here to access/download;Figure;Fig_S7.pdf

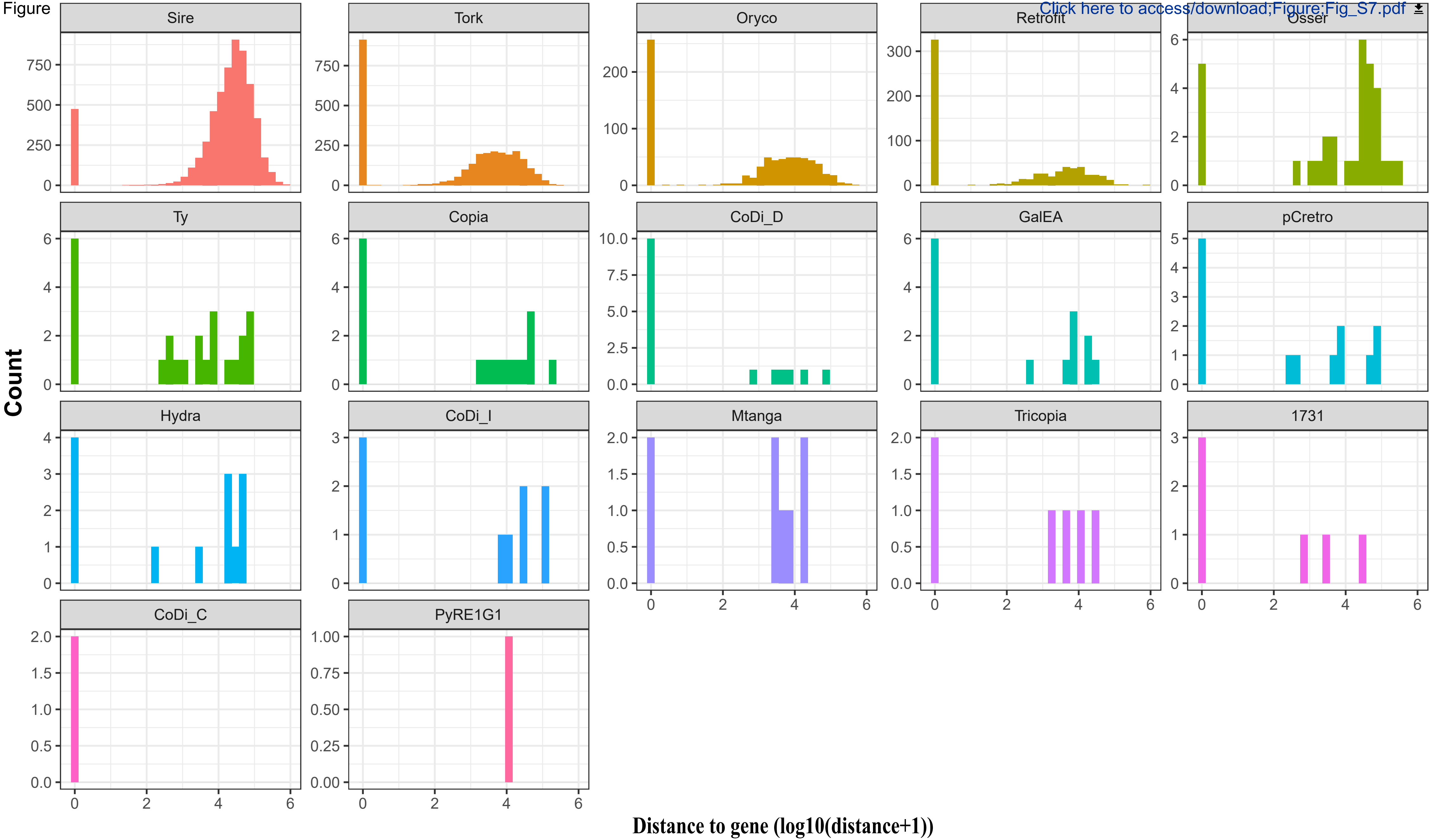

Click here to access/download;Figure;Fig_S8.png

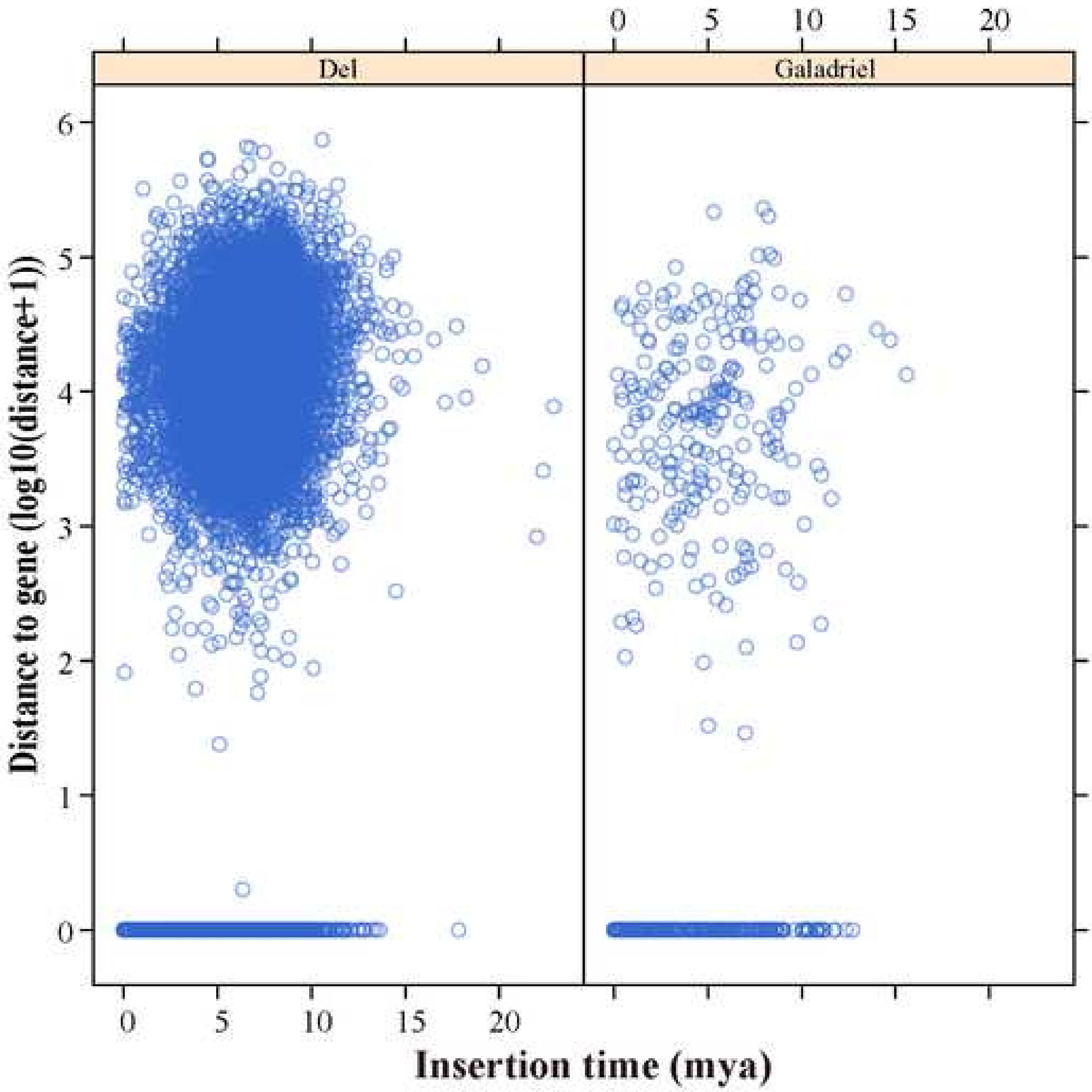

Figure

Click here to access/download;Figure;Fig_S9.png

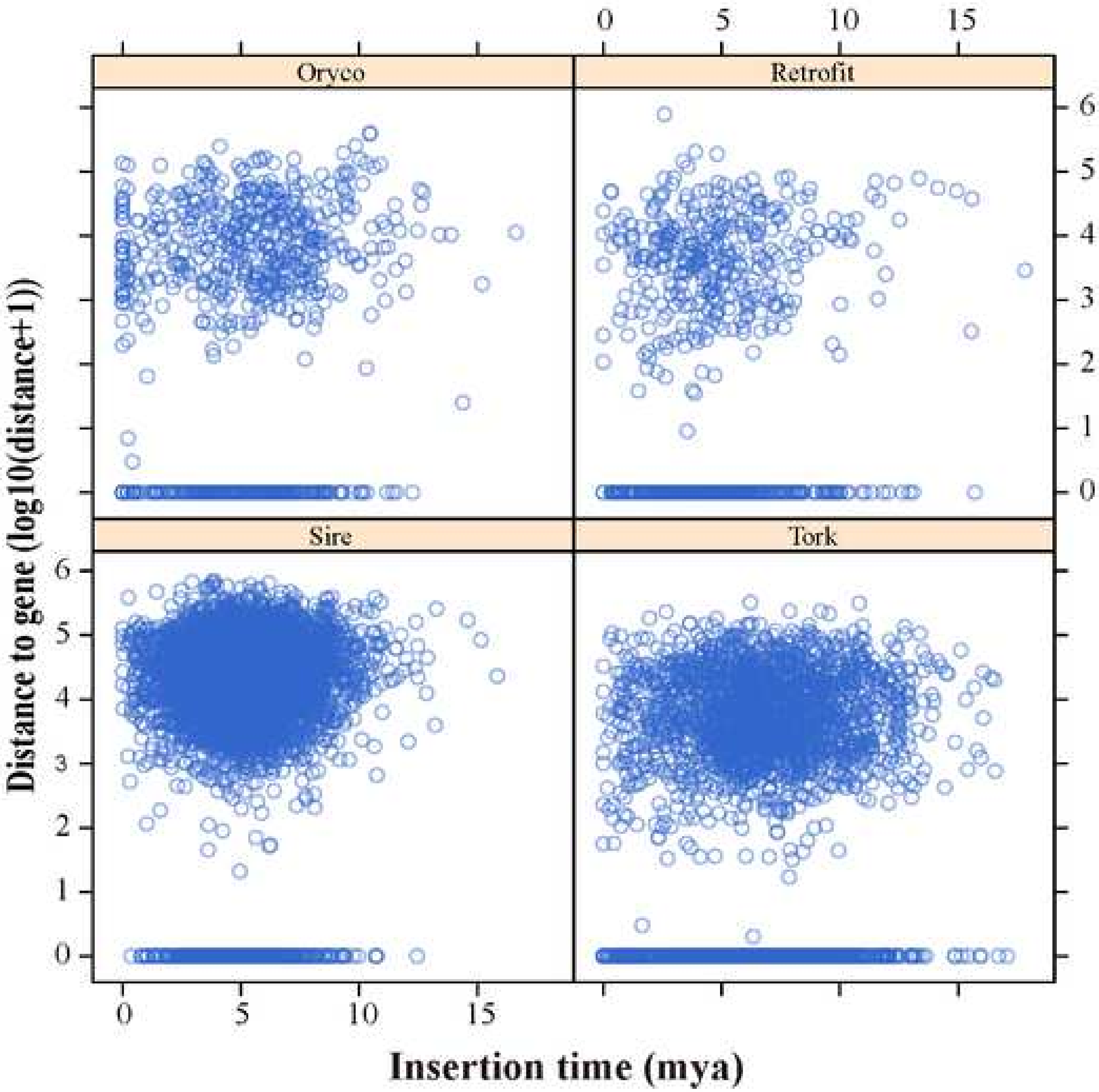

Figure

Click here to access/download;Figure;Fig_S10.pdf

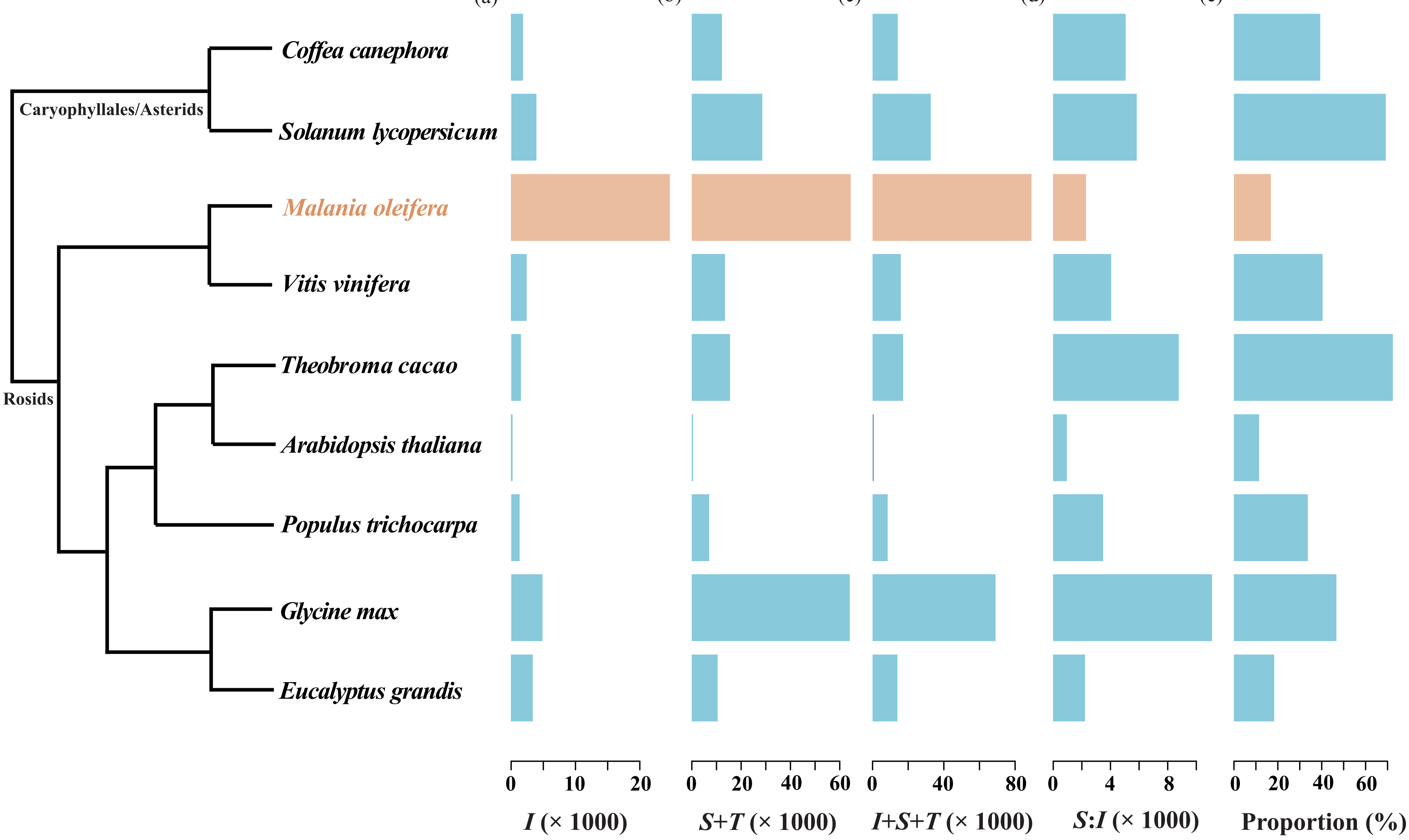

Figure

Click here to access/download;Figure;Fig_S11.pdf

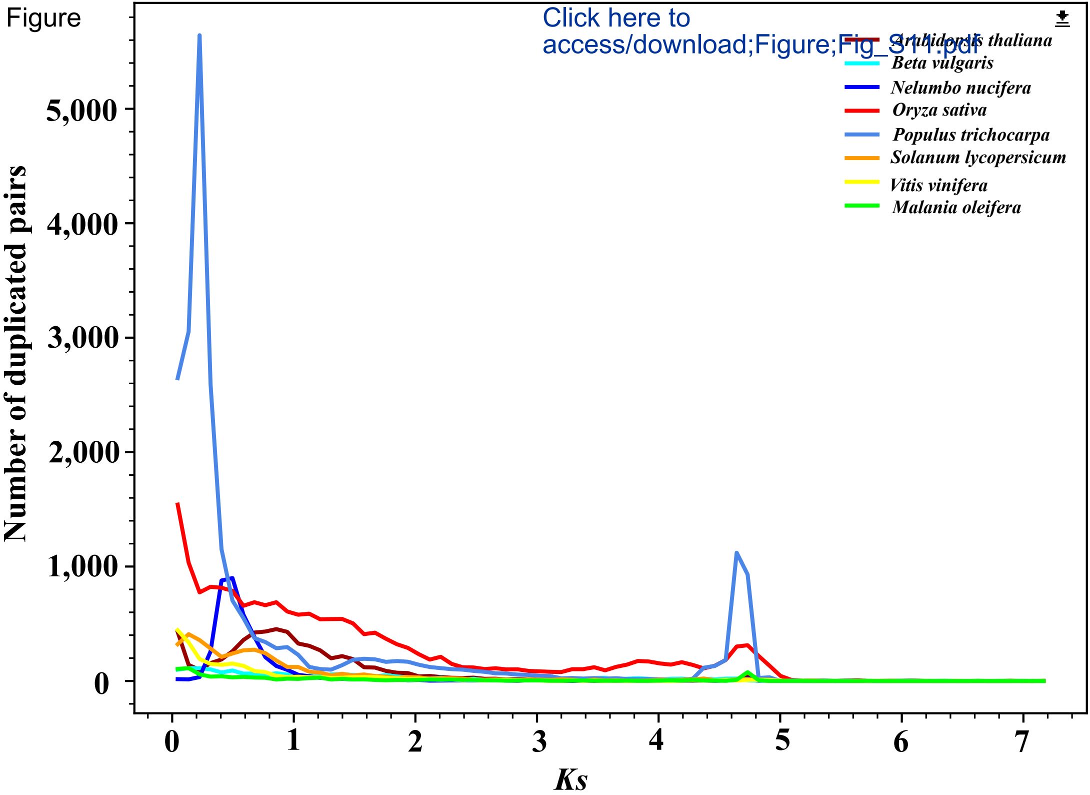

Figure

Click here to access/download;Figure;Fig_S12.pdf

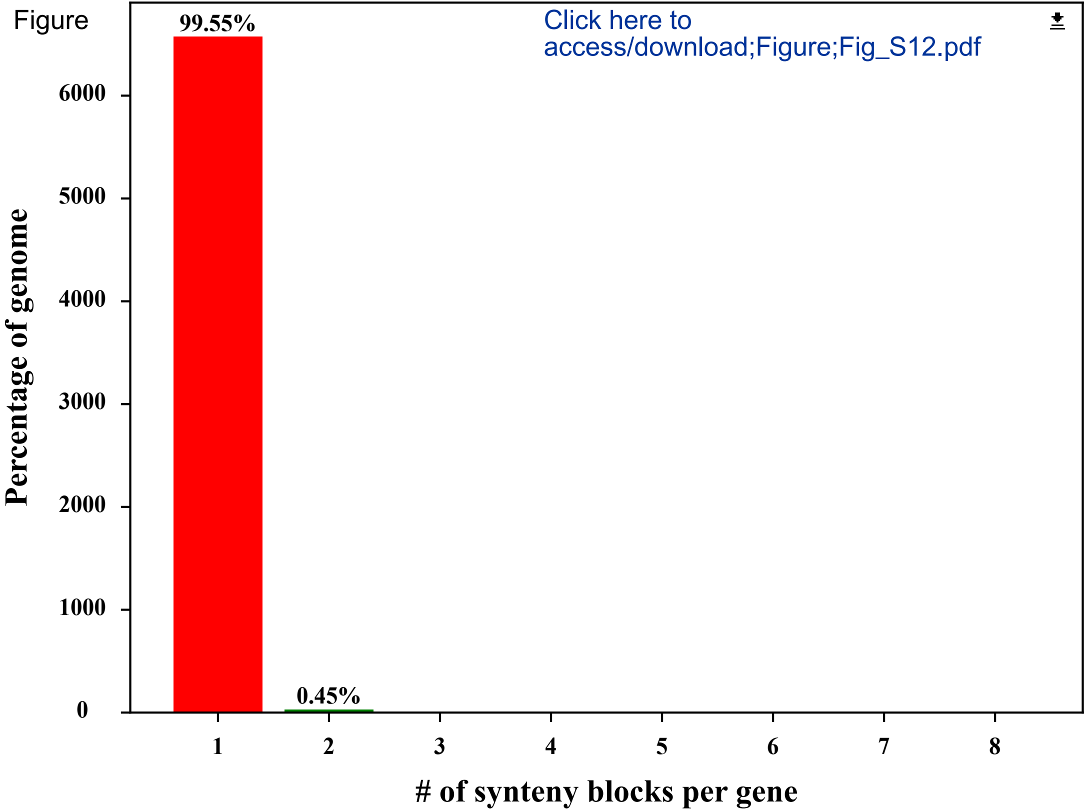

Figure

Click here to access/download

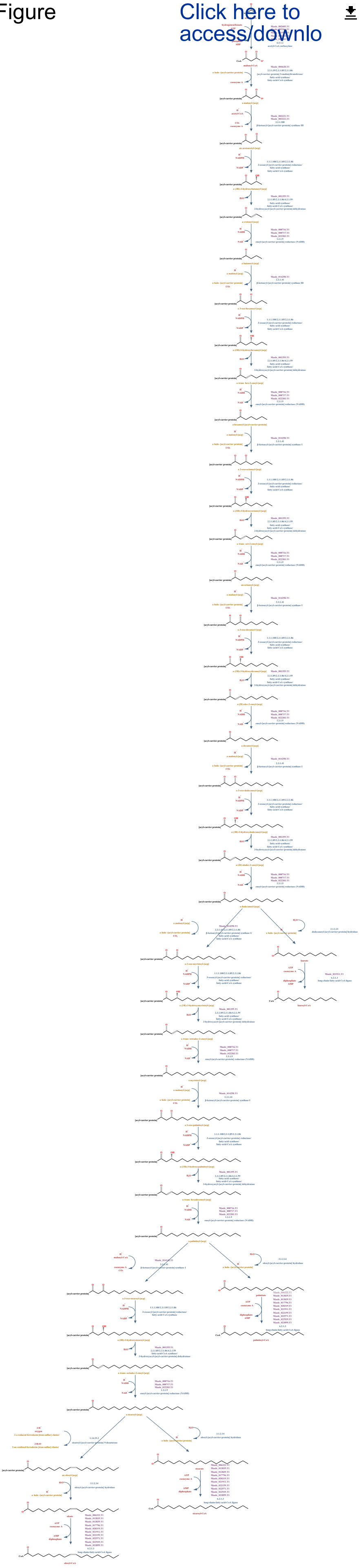

Click here to access/download
Supplementary Material
Supplementary File 1.docx

Click here to access/download
Supplementary Material
Supplementary File 2.txt

Supplementary Material

Click here to access/download
Supplementary Material
Supplementary File 3.phy

Click here to access/download
Supplementary Material
Supplementary File 4.docx
