# Supplementary material for: Genome sequence of Malania oleifera, a tree with great value for nervonic acid production
Source: Gigascience. 2019 Jan 24;8(2):giy164. doi: 10.1093/gigascience/giy164 (PMC6377399; doi:10.1093/gigascience/giy164)
Supplement: Supplemental Files [file giy164_supplemental_files.zip › Supplementary File 1.docx]

1. **Genome assembly**
   1. **Assembly with long noisy PacBio reads**
      1. **assemble using SMARTDENOVO**

smartdenovo.pl -c 1 -t 12 -k 17 -p smrtdenovo all.subreads.fasta > Makefile

make all

- - 1. **polish ×2 using arrow with PacBio reads**

ln smrtdenovo.dmo.cns ref.fa -s

blasr pacbio.fofn ref.fa -out pacbio.fofn.bam --bam --bestn 10 --minMatch 12 --maxMatch 30 --nproc 20 --minSubreadLength 50 --minAlnLength 50 --minPctSimilarity 70 --minPctAccuracy 70 --hitPolicy randombest --randomSeed 1

samtools sort -m 4G --threads 40 -o pacbio.fofn.bam all_mapped.bam

samtools faidx ref.fa

samtools index all_mapped.bam

pbindex all_mapped.bam

arrow -j 40 all_mapped.bam -r ref.fa -o consensus.fa -o consensus.fq -o variants.gff

- 1. **Scaffolding with 10X Genomics linked reads**
     1. **mapping 10X reads to the polished contigs using longranger**

ln consensus.fa ref.fa -s

longranger mkref ref.fa

longranger align \

--reference=refdata-ref \

--id=mapping \

--jobmode=sge \

--maxjobs=30 \

--mempercore=2 \

--fastqs=10Xgenomics/fastqs/raw \

--sample=NDHX00184-AK145,NDHX00184-AK146,NDHX00184-AK147,NDHX00184-AK148

**1.2.2 scaffolding using ARCS**

ls mapping/ALIGNER_CS/ALIGNER/_ALIGNER/ATTACH_BCS/fork*/chnk*/files/output.bam.sort.bam > bam.list

arcs -f ref.fa -a bam.list -s 98 -c 5 -l 0 -z 500 -m 50-1000 -d 0 -e 30000 -r 0.05 -v --bx -D

graph= ref.fa.scaff_s98_c5_l0_d0_e30000_r0.05_original.gv

makeTSVfile.py $graph ref.fa.c5_e30000_r0.05.tigpair_checkpoint.tsv ref.fa

touch empty.fof

LINKS -f ref.fa -s empty.fof -k 20 -b $f.c5_e30000_r0.05 -l 5 -t 2 -a 0.3

cut -f1 -d “,” ref.fa.c5_e30000_r0.05.scaffolds.fa > scaffolds.fa

- - 1. **polish using arrow with PacBio reads**

ln scaffolds.fa ref.fa -s

The following is same as 1.1.2.

- - 1. **polish ×3 using pilon with 10X Genomics reads**

ln consensus.fa ref.fa -s

# mapping illumina reads to the genome

bowtie2 --local -p 40 -x ref.fa -1 reads.R1.fastq.gz -2 reads.R2.fastq.gz --rg-id X --rg "SM:X" --rg "PL:illumina" --rg "LB:lib1" --rg "PU:unit1" | samtools sort --threads 40 > mapped.sort.bam

# remove duplicates

gatk MarkDuplicates --MAX_FILE_HANDLES_FOR_READ_ENDS_MAP 1024 -I mapped.sort.bam -O mapped.final.bam -M mapped.sort.bam.dup_metrics --REMOVE_DUPLICATES true

samtools index mapped.final.bam

# run pilon

java -jar pilon.jar --genome ref.fa --bam mapped.final.bam \

--output pilon --outdir ./ --changes \

--diploid --dumpreads --threads 40

**2. Quality assessment of the assembly**

ln pilon.fasta ref.fa -s

# run BUSCO

BUSCO.py -i ref.fa -o ref.fa -l BUSCO_db/embryophyta_odb9 -c 8 -z -m geno –long

# call SNP

bowtie2 --local -p 40 -x ref.fa -1 reads.R1.fastq.gz -2 reads.R2.fastq.gz --rg-id X --rg "SM:X" --rg "PL:illumina" --rg "LB:lib1" --rg "PU:unit1" | samtools sort --threads 40 > mapped.sort.bam

gatk MarkDuplicates --MAX_FILE_HANDLES_FOR_READ_ENDS_MAP 1024 -I mapped.sort.bam -O mapped.final.bam -M mapped.sort.bam.dup_metrics --REMOVE_DUPLICATES true

samtools mpileup --output-tags DP,AD -gf ref.fa mapped.final.bam | bcftools call -cv > samtools.vcf

1. **Assembly of RNA-Seq**

# mapping reads to the genome

ln pilon.fasta ref.fa -s

hisat2-build ref.fa ref.fa

hisat2 -p 20 --dta -x ref.fa -1 reads_1.fq -2 reads_2.fq --dta-cufflinks --summary-file SAMPLE.summary --new-summary --rg-id SAMPLE | samtools sort > SAMPLE.bam

# reference-based assembly using StingTie

stringtie -p 20 -o SAMPLE.gtf -l SAMPLE SAMPLE.bam

# genome-guilded assembly using Trinity

Trinity --min_kmer_cov 10 --output trinityGG_out_dir --genome_guided_bam SAMPLE.bam --max_memory 200G --genome_guided_max_intron 10000 --CPU 20 --genome_guided_min_coverage 3

# de novo assembly using Trinity

Trinity --KMER_SIZE 25 --output trinity_out_dir --no_version_check --seqType fq --max_memory 200G --left reads_1.fq --right reads_2.fq --CPU 20 --min_kmer_cov 10

1. **repeat annotation**

# de novo build repeat library using RepeatModeler

BuildDatabase -name RMdbase -engine ncbi ref.fa

RepeatModeler -engine ncbi -pa 20 -database RMdbase

# classify repeat elements

Mlib=`ls */consensi.fa.classified`

RepeatMasker -lib $Mlib ref.fa -parallel 20 -dir . -gff

1. **gene annotation**

# maker config file (maker_opts.ctl)

#-----Genome (these are always required)

genome=ref.fa.masked #genome sequence (fasta file or fasta embeded in GFF3 file)

organism_type=eukaryotic #eukaryotic or prokaryotic. Default is eukaryotic

#-----Re-annotation Using MAKER Derived GFF3

maker_gff= #MAKER derived GFF3 file

est_pass=0 #use ESTs in maker_gff: 1 = yes, 0 = no

altest_pass=0 #use alternate organism ESTs in maker_gff: 1 = yes, 0 = no

protein_pass=0 #use protein alignments in maker_gff: 1 = yes, 0 = no

rm_pass=0 #use repeats in maker_gff: 1 = yes, 0 = no

model_pass=0 #use gene models in maker_gff: 1 = yes, 0 = no

pred_pass=0 #use ab-initio predictions in maker_gff: 1 = yes, 0 = no

other_pass=0 #passthrough anyything else in maker_gff: 1 = yes, 0 = no

#-----EST Evidence (for best results provide a file for at least one)

est=est.clust.fa #set of ESTs or assembled mRNA-seq in fasta format

altest= #EST/cDNA sequence file in fasta format from an alternate organism

est_gff= #aligned ESTs or mRNA-seq from an external GFF3 file

altest_gff= #aligned ESTs from a closly relate species in GFF3 format

#-----Protein Homology Evidence (for best results provide a file for at least one)

protein=pep.clust.fa #protein sequence file in fasta format (i.e. from mutiple oransisms)

protein_gff= #aligned protein homology evidence from an external GFF3 file

#-----Repeat Masking (leave values blank to skip repeat masking)

model_org= #select a model organism for RepBase masking in RepeatMasker

rmlib= #provide an organism specific repeat library in fasta format for RepeatMasker

repeat_protein= #provide a fasta file of transposable element proteins for RepeatRunner

rm_gff= #pre-identified repeat elements from an external GFF3 file

prok_rm=0 #forces MAKER to repeatmask prokaryotes (no reason to change this), 1 = yes, 0 = no

softmask=1 #use soft-masking rather than hard-masking in BLAST (i.e. seg and dust filtering)

#-----Gene Prediction

snaphmm= #SNAP HMM file

gmhmm= #GeneMark HMM file

augustus_species=BUSCO_4097971693 #Augustus gene prediction species model

fgenesh_par_file= #FGENESH parameter file

pred_gff= #ab-initio predictions from an external GFF3 file

model_gff= #annotated gene models from an external GFF3 file (annotation pass-through)

est2genome=0 #infer gene predictions directly from ESTs, 1 = yes, 0 = no

protein2genome=0 #infer predictions from protein homology, 1 = yes, 0 = no

trna=0 #find tRNAs with tRNAscan, 1 = yes, 0 = no

snoscan_rrna= #rRNA file to have Snoscan find snoRNAs

unmask=0 #also run ab-initio prediction programs on unmasked sequence, 1 = yes, 0 = no

#-----Other Annotation Feature Types (features MAKER doesn't recognize)

other_gff= #extra features to pass-through to final MAKER generated GFF3 file

#-----External Application Behavior Options

alt_peptide=C #amino acid used to replace non-standard amino acids in BLAST databases

cpus=1 #max number of cpus to use in BLAST and RepeatMasker (not for MPI, leave 1 when using MPI)

#-----MAKER Behavior Options

max_dna_len=100000 #length for dividing up contigs into chunks (increases/decreases memory usage)

min_contig=1 #skip genome contigs below this length (under 10kb are often useless)

pred_flank=200 #flank for extending evidence clusters sent to gene predictors

pred_stats=0 #report AED and QI statistics for all predictions as well as models

AED_threshold=1 #Maximum Annotation Edit Distance allowed (bound by 0 and 1)

min_protein=0 #require at least this many amino acids in predicted proteins

alt_splice=0 #Take extra steps to try and find alternative splicing, 1 = yes, 0 = no

always_complete=0 #extra steps to force start and stop codons, 1 = yes, 0 = no

map_forward=0 #map names and attributes forward from old GFF3 genes, 1 = yes, 0 = no

keep_preds=0 #Concordance threshold to add unsupported gene prediction (bound by 0 and 1)

split_hit=10000 #length for the splitting of hits (expected max intron size for evidence alignments)

single_exon=0 #consider single exon EST evidence when generating annotations, 1 = yes, 0 = no

single_length=250 #min length required for single exon ESTs if 'single_exon is enabled'

correct_est_fusion=0 #limits use of ESTs in annotation to avoid fusion genes

tries=5 #number of times to try a contig if there is a failure for some reason

clean_try=0 #remove all data from previous run before retrying, 1 = yes, 0 = no

clean_up=0 #removes theVoid directory with individual analysis files, 1 = yes, 0 = no

TMP= #specify a directory other than the system default temporary directory for temporary files

# run maker pipeline

maker

1. **orthologs identification**

orthomcl_config=~/bin/orthomclSoftware-v2.0.9/config/orthomcl.config.template

orthomclInstallSchema $orthomcl_config

mkdir compliantFasta -p

for SP in `cut -f 1 sp.list`

do

orthomclAdjustFasta $SP ref/$SP/pep.faa 1

mv $SP.fasta compliantFasta

done

# filter

orthomclFilterFasta compliantFasta 10 20

# all vs all blast

formatdb -t goodProteins.fasta -i goodProteins.fasta -p T

blastall -F 'm S' -v 100000 -b 100000 -e 1e-5 -a 8 -i goodProteins.fasta -d goodProteins.fasta -o all_VS_all.out.tab -m 8 -p blastp

orthomclBlastParser all_VS_all.out.tab compliantFasta/ | sort -k 1,1 -k 2,2 -u > similarSequences.txt

# load to mySQL

orthomclLoadBlast $orthomcl_config similarSequences.txt

orthomclPairs $orthomcl_config orthomc_pairs.log cleanup=no

orthomclDumpPairsFiles $orthomcl_config

# cluster

mcl mclInput --abc -I 1.5 -o mcloutput

orthomclMclToGroups OG_ 1 < mcloutput > groups.txt

1. **phylogenetic reconstruction and dating**

path=/share/home/…/orthologs/bin

###############################

# single copy gene groups

###############################

python $path/singlecopy_group.py \

groups.txt species.design > singlecopy_groups.txt

#################################

# cancatenate single copy cds
#################################

python $path/singlecopy_cds_concatenated.py \

groups.txt species.design ../cds.fasta > singlecopy_cds.fa

#################################

# convert protein alignment to cds alignment

#################################

perl bin/pepMfa_to_cdsMfa.pl data/singlecopy_aligned.faa data/singlecopy_cds.fa > data/singlecopy_cds_aligned.fa

#################################

# Remove spurious sequences and columns using TrimAl

#################################

trimal -gt 0.8 -st 0.001 -cons 60 -in data/singlecopy_cds_aligned.fa -out data/singlecopy_cds_aligned.trimal.fa

#################################

# convert fasta to phylip format

#################################

python bin/convert_fasta2phylip.py data/singlecopy_cds_aligned.trimal.fa data/singlecopy_cds_aligned.phy

#################################

# phylogenetic reconstruction

#################################

# evolutionary model selection

iqtree -s singlecopy_aligned.trimal.phy -m MF -nt 20

# compute the ML tree

./iqtree -s singlecopy_aligned.trimal.phy -m JTT+F+R5 -bb 1000 -alrt 1000 -nt 20

##################################

# reroot tree

##################################

cd /media/12TB/liuhui/Malania_oleifera/phylo_dating

Rscript bin/reroot_tree.R data/singlecopy_aligned.trimal.phy.treefile Oryza_sativa data/Mol_IQtree.ml.tre

# Rscript bin/reroot_tree_with_support_value.R data/singlecopy_aligned.trimal.phy.treefile Oryza_sativa data/Mol_IQtree.sp.ml.tre

# sed -i 's/Root;$/;/' data/Mol_IQtree.sp.ml.tre
sed "s/;/'@1.3';/" data/Mol_IQtree.ml.tre > data/Mol_IQtree_baseml.ml.tre

sed -i '1i15 1' data/Mol_IQtree_Cal.ml.tre

###########################

# Dating

###########################

#(1) Rough estimation of the substitution rate

cd /Pinus1/Liuhui/Malania_oleifera/phylo_dating/baseml
baseml &> baseml.log

rate=$(grep -A 1 'Substitution' mlb | tail -n 1 | awk '{print $1}')

Rscript ../bin/rgnene_gamma.R $rate

# (2) Branch Lengths

### mcmctree.ctl

# seed = -1

# seqfile = ../data/singlecopy_cds_aligned.phy

# treefile = ../data/Xso_IQtree_Cal.ml.tre

# outfile = out_usedata2

#

# ndata = 1

# usedata = 2 * 0: no data; 1:seq like; 2:normal approximation

# clock = 2 * 1: global clock; 2: independent rates; 3: correlated rates

# RootAge = '<10' * constraint on root age, used if no fossil for root.

#

# model = 7 * 0:JC69, 1:K80, 2:F81, 3:F84, 4:HKY85

# alpha = 0.5 * alpha for gamma rates at sites

# ncatG = 5 * No. categories in discrete gamma

#

# cleandata = 0 * remove sites with ambiguity data (1:yes, 0:no)?

#

# BDparas = 1 1 0 * birth, death, sampling

# kappa_gamma = 6 2 * gamma prior for kappa

# alpha_gamma = 1 1 * gamma prior for alpha

#

# rgene_gamma = 1 1.4 * gamma prior for rate for genes

# sigma2_gamma = 1 4.5 * gamma prior for sigma^2 (for clock=2 or 3)

#

# finetune = 1: 0.2 0.11 0.12 0.1 0.15 * times, rates, mixing, paras, RateParas

#

# print = 1

# burnin = 100000

# sampfreq = 200

# nsample = 10000

cd /Pinus1/Liuhui/Malania_oleifera/phylo_dating/branch_lengths

mcmctree &> branch_lengths.log

# (3) Estimation of Time and Rate by MCMC Analysis

cd /Pinus1/Liuhui/Malania_oleifera/phylo_dating/dating_rep1

cp ../branch_lengths/out.BV in.BV

# /media/12TB/liuhui/bin/paml4.9h/bin/mcmctree &> mcmctree.log
mcmctree &> mcmctree.log

mv FigTree.tre Mol_IQtree_mcmctree.ml.tre
