# Supplementary material for: Genome sequence of Malania oleifera, a tree with great value for nervonic acid production
Source: Gigascience. 2019 Jan 24;8(2):giy164. doi: 10.1093/gigascience/giy164 (PMC6377399; doi:10.1093/gigascience/giy164)

1. scaffold13 - Gene Cluster 1. Type = lignan-polyketide. Location: 981159 - 1376309 nt.


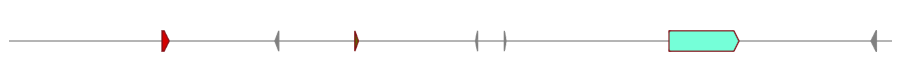


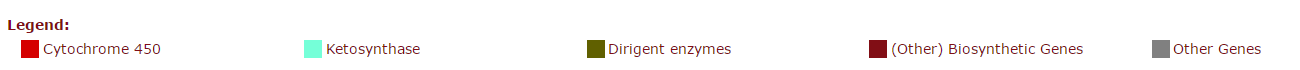


1. scaffold132 - Gene Cluster 2. Type = putative. Location: 34174 - 689685 nt.


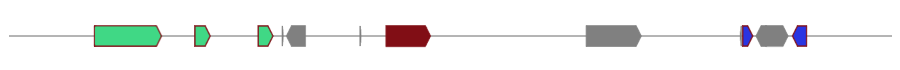


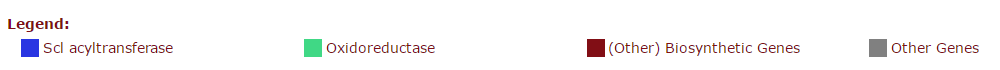


1. scaffold148 - Gene Cluster 3. Type = terpene. Location: 234014 - 673747 nt.


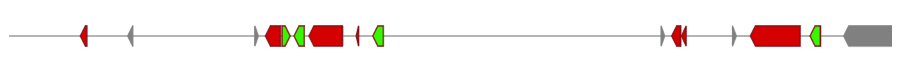


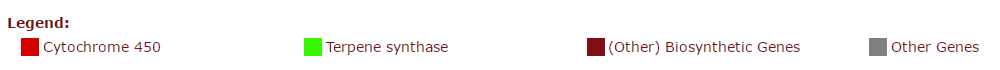


1. scaffold154 - Gene Cluster 4. Type = putative. Location: 2232746 - 2490786 nt.


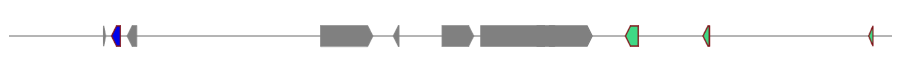


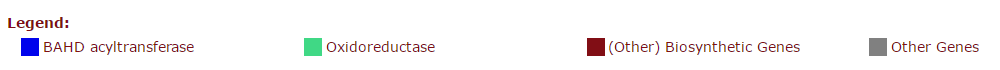


1. scaffold160 - Gene Cluster 5. Type = saccharide. Location: 580781 - 1087231 nt.


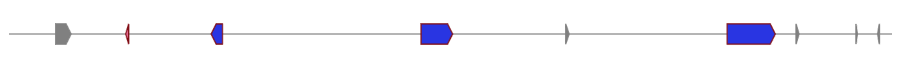


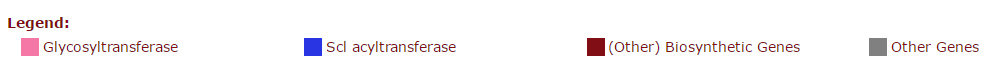


1. scaffold170 - Gene Cluster 6. Type = saccharide. Location: 1847925 - 2973087 nt.


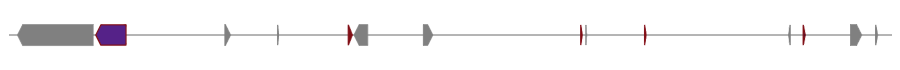


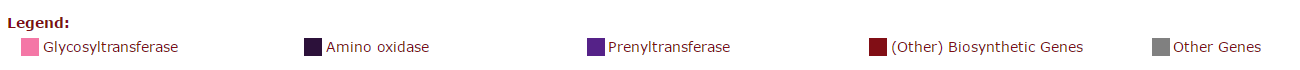


1. scaffold175 - Gene Cluster 7. Type = terpene. Location: 582938 - 1206986 nt.


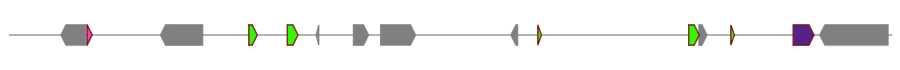


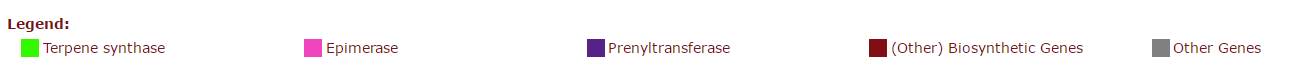


1. scaffold21 - Gene Cluster 8. Type = saccharide-polyketide. Location: 9844715 - 10494316 nt.


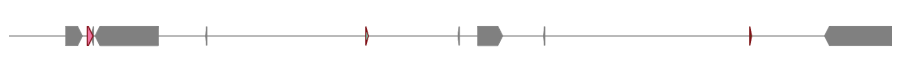


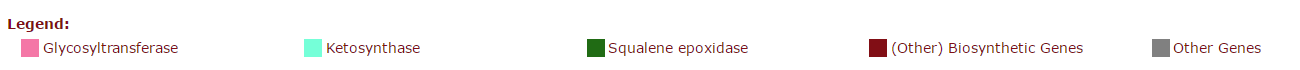


1. scaffold268 - Gene Cluster 9. Type = putative. Location: 1079703 - 2003831 nt.


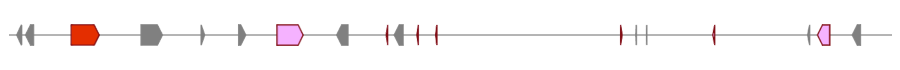


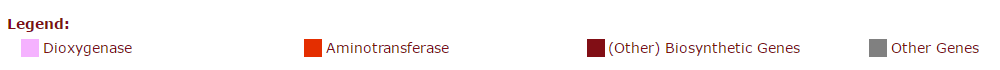


1. scaffold28 - Gene Cluster 10. Type = saccharide. Location: 511674 - 1729579 nt.


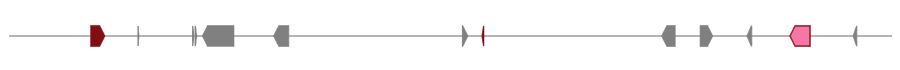


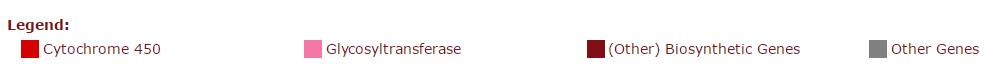


1. scaffold304 - Gene Cluster 11. Type = terpene. Location: 1065427 - 1391381 nt.


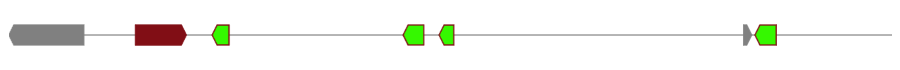


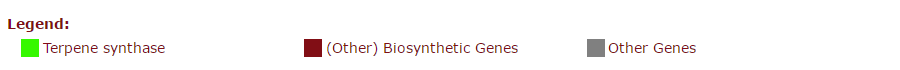


1. scaffold317 - Gene Cluster 12. Type = putative. Location: 295028 - 941249 nt.


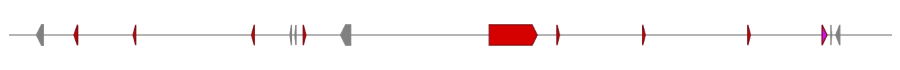


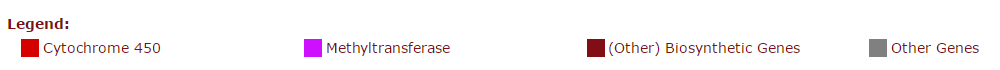


1. scaffold33 - Gene Cluster 13. Type = saccharide. Location: 7536717 - 8466817 nt.


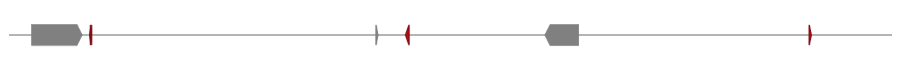


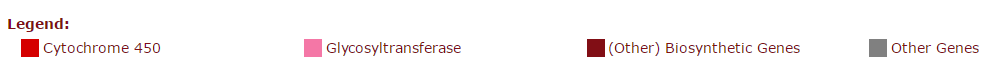


1. scaffold338 - Gene Cluster 14. Type = putative. Location: 760835 - 1070430 nt.


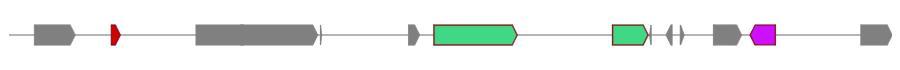


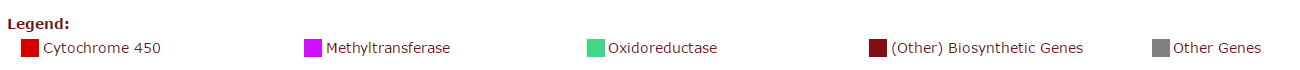


1. scaffold34 - Gene Cluster 15. Type = saccharide. Location: 5724863 - 6208948 nt.


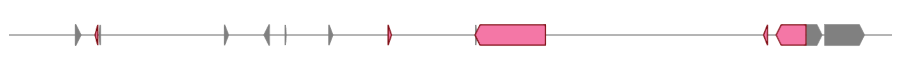


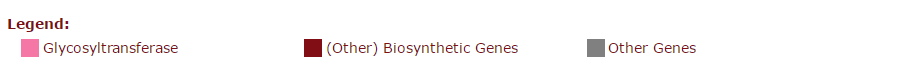


1. scaffold36 - Gene Cluster 16. Type = saccharide. Location: 2373331 - 2926426 nt.


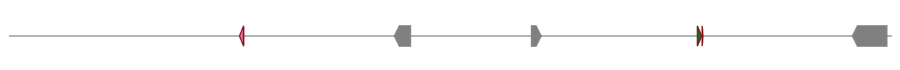


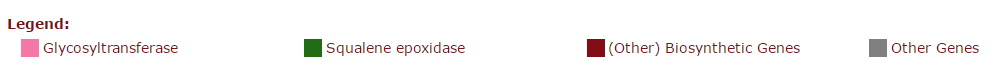


1. scaffold383 - Gene Cluster 17. Type = alkaloid. Location: 1 - 389285 nt.


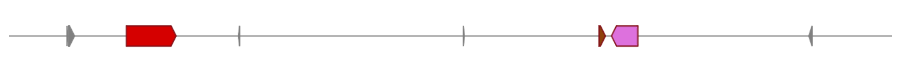


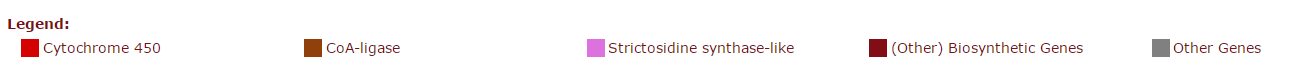


1. scaffold394 - Gene Cluster 18. Type = saccharide. Location: 578831 - 793915 nt.


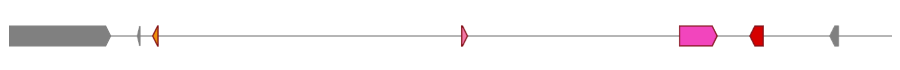


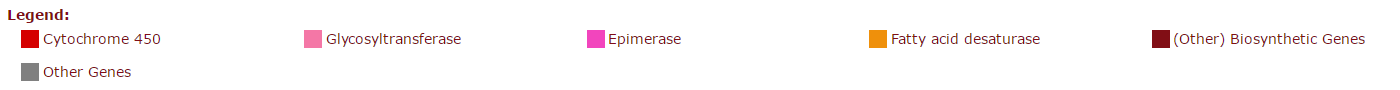


1. scaffold42 - Gene Cluster 19. Type = terpene. Location: 1159540 - 1602377 nt.


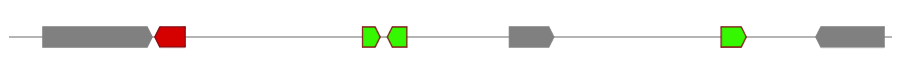


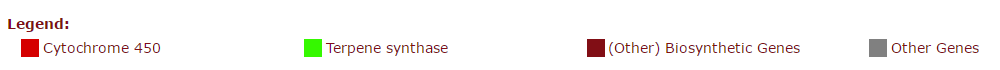


1. scaffold54 - Gene Cluster 20. Type = saccharide. Location: 7265034 - 8021036 nt.


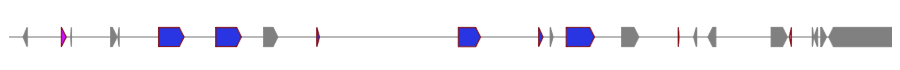


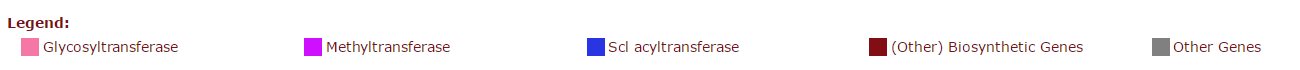


1. scaffold67 - Gene Cluster 21. Type = alkaloid. Location: 2978547 - 3622210 nt.


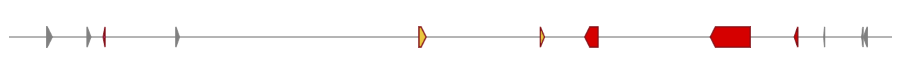


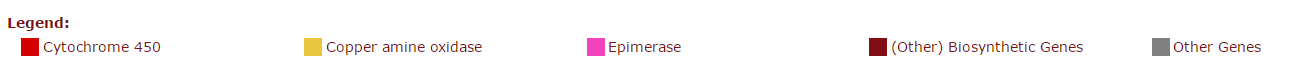


1. scaffold73 - Gene Cluster 22. Type = saccharide. Location: 1429564 - 2050061 nt.


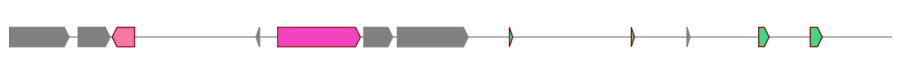


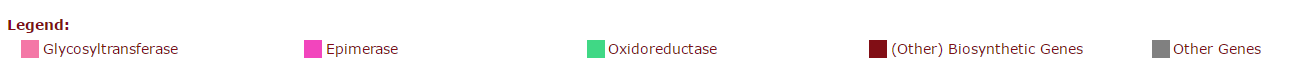


1. scaffold8 - Gene Cluster 23. Type = polyketide. Location: 4529957 - 5812302 nt.


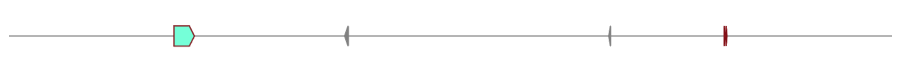


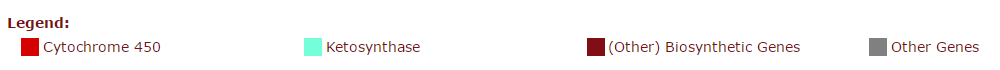

Supplement: Supplemental Files [file giy164_supplemental_files.zip › Supplementary File 4.docx]
