# Supplementary material for: Genome sequence of Malania oleifera, a tree with great value for nervonic acid production
Source: Gigascience. 2019 Jan 24;8(2):giy164. doi: 10.1093/gigascience/giy164 (PMC6377399; doi:10.1093/gigascience/giy164)
Supplement: Response_to_Reviewer_Comments_Original_Submission.pdf [file giy164_response_to_reviewer_comments_original_submission.pdf]

Author's Response To Reviewer Comments

Close

Scott Edmunds, Editor
GigaScience

Dear Dr. Edmunds,

RE: ID: GIGA-D-18-00301 - “Genome sequence of Malania oleifera, an endangered tree with great value for nervonic acid production”

Enclosed, please find the revised version of the above-mentioned MS. We would like to start with showing our great appreciation for the helpful and constructive comments and suggestions from you and the reviewers. These comments were helpful in improving and clarifying many parts of the manuscript.

##
Comments from Scott Edmunds, Editor
1. More methodological detail (software version numbers, and we strongly recommend including protocols in protocols.io: https://www.protocols.io/groups/gigascience-journal, or reusing or adapting relevant protocols there).
Response: We now provide detailed information on all commands and parameter settings for genome assembly, quality assessment of genome assembly, transcriptome assembly from RNA-seq, repeat and gene annotation, ortholog identification, phylogenetic reconstruction and dating (see Supplementary File 1). This file has also been uploaded to protocols.io, with a DOI code: dx.doi.org/10.17504/protocols.io.u7nezme.

2. RRIDs are also useful for the reproducibility of the software:
ftp://penguin.genomics.cn/pub/10.5524/RRID/RRIDlist.pdf
Response: Yes, we now provide RRID alongside each software where applicable.

##
Comments from Catherine Jane Nock (Reviewer #1)
Reviewer #1: This manuscript provides the first genome assembly and genomic resources for the threatened tree Malania oleifera. The manuscript provides extensive novel information for this species and the order Santalales. The extremely high repeat content and low heterozygosity reported are of particular interest, as is the identification of candidate genes in the VLCFA biosynthesis pathway.

1. Despite the species listing on IUCN Red List as vulnerable, the authors incorrectly refer to the species as 'endangered' in the title, keywords and throughout the manuscript. Needs to be corrected to either 'threatened' or 'vulnerable'.
Response: The reviewer is correct, and in our revised version, we have now replaced ‘endangered’ with ‘vulnerable’. The revised title now reads “Genome sequence of Malania oleifera, a tree with great value for nervonic acid production”.

2. Substantial supplementary data is provided with this manuscript, but there is very limited detail beyond which programs were used for the de novo assembly pipelines. More information on the parameters applied is needed, at least for the v0.7 assembly used for scaffolding. In general, limited detail is provided on the analysis methods used in the study.
Response: We now provide detailed information on all commands and parameter settings used for genome assembly, quality assessment of genome assembly, transcriptome assembly, repeat and gene annotation, ortholog identification and phylogenetic reconstruction and dating. All information is provided both as a supplementary file (Supplementary File 1), and is uploaded to protocols.io under DOI code: dx.doi.org/10.17504/protocols.io.u7nezme.

3. A major concern is the phylogenetic analysis and molecular dating based on single copy genes. Details of the alignment and analysis methods and results are not provided. A strict molecular clock is applied to the concatenated alignment. While there is no report of testing for clocklike evolution, previous evidence suggests that this highly unlikely. Many relationships within the tree are not supported by previous studies but this is not discussed e.g. Vitales (Vitis) + Santalales (Malania) clade, Proteales (Nelumbo) + Ranunculales (Acquilegia) clade rather than successive sister groups diverging after appearance of core eudicots rather than before. What outgroup was used? The >250 mya divergence between Orzya and eudicots is substantially older than previous estimates. What were the clade support values? The divergence dates and relationships inferred are, at best, unreliable.
Responses: We have revisited this part of the analyses, using IQ-Tree for phylogenetic reconstruction and MCMCTREE from PAML for dating. Substitution model selection and molecular clock testing were added. In short, tree topologies remained the same and all branches gain good support from bootstrap analysis, while dating was, in our opinion, improved and is now more accurate. We (did) provide names/IDs of the single copy genes identified for each of the plant genomes analyzed (please see Supplementary File 2), the alignment (please see Supplementary File 3), as well as detailed description of the updated phylogenetic reconstruction, dating and molecular evolution testing, and results (lines 416-456. As stated higher, all commands and parameter settings used are provided both as a supplementary file (Supplementary File 1), and is uploaded to protocols.io under DOI code: dx.doi.org/10.17504/protocols.io.u7nezme.

4. The reported repeat content of the genome (82%) is extremely high and a thorough analysis of the most abundant repeats (LTR-RT, 58.23% of genome) is presented. If most were the result of ancient amplification how can the high number of intact LTR-RT (Fig S10) and low removal rates and be explained?
Responses: Well, the fact that there are still many intact LTR-RTs from ancient amplification is exactly the result of low removal rates. We do not think there is a contradiction. The mechanism(s) underlying the both the low recent accumulation of LTR-RT amplifications and the low rate of DNA removal are unknown in this study (and are unknown in all other published studies). We can only make the observation at this time, and hope this encourages future studies to investigate this question.

Minor Comments
5. Sentence at Line 90-94 is too long and difficult to interpret. Distinction between effects in

healthy individuals and those with a genetic defect is unclear. Are the authors suggesting a link between demyelination and obesity?
Responses: In the revised version of our manuscript we have changed this sentence to: "Nervonic acid is an important component in myelin biosynthesis in the central and peripheral nervous system. Myelin is generally localized to the sphingomyelin of animal cell membranes [12], where it has been proposed to enhance human brain function. Treatment of myelin disorders may attenuate or prevent various psychotic disorders [13, 14]".

6. Replace 'Chun et S. Lee' with 'Chun & SK Lee'. Author citation for the species probably unnecessary in abstract.
Responses: Done.

7. Line 91: 'nerve system' correct to nervous system
Responses: Done.

8. Line 104-105: clarify what is meant by 'species' endangering mechanism'
Responses: We replaced "species' endangering mechanism" with "provide valuable tools for the genomic dissection of the species' genetic diversity and its population demography for future conservation purposes". Please see lines 103-105.

9. Line179: report k-mer value of 17 in addition to depth
Responses: Done. Please see line 177.

10. Line 201-202: '71.80% gene completeness as determined by BUSCO assessment (Table S4)' comes before the description of gene annotation methods.
Responses: Yes. BUSCO assessment is a mean we used to assess the quality of different versions of genome assembly, it is not part of gene annotation.

11. Numbering is inconsistent, e.g. Line 141 '51,149,552,938 bases', Line 244 '313.36 million reads'. Suggest using million/billion or Mb/Gb throughout
Responses: "51,149,552,938 bases" was changed to "51.15 Gb", see line 140.

##
Comments from Stephen Tsui (Reviewer #2)
Reviewer #2: This article described the genome sequencing of Malania oleifera Chun et S. Lee, a member of the Olacaceae family. A lot of work has been done but more efforts should be paid to highlight the importance of this genome sequence and related resources. The following comments should be helpful for the revision of this piece of work.

1. The quality of the genome is in general high. However, the gene number (24,094) is a bit less than expected because many plants have 40,000 to 60,000 genes. It is not known whether this is related to the parameters used for the gene prediction. Explanation / support about the gene number should be provided.
Responses: Gene numbers in plant genomes differ widely. Most plant genomes for which 40,000 to 60,000 genes have been reported are either hugely inflated (because they still contain several thousands of TEs), or are from polyploids, or are from paleopolyploids, i.e. genomes

that have undergone ancient whole genome duplications. Plant genomes for which no or very few polyploidy events have been reported, usually have genomes encoding about 23,000 to 25,000 genes, so the gene content of Malania, for which there is no evidence for recent or ancient genome duplications, is well within expectations.

2. Quite a lot of paragraphs discuss the LTR-RT in the plant. However, relatively less efforts were put on the metabolic uniqueness of this plant. What make this plant to be so important for genome sequencing? The authors should try to explore those unique features of this plant through the genome they generated. Otherwise, the genome data has not been fully utilized.
Responses: Much effort went into discussing LTR-RT, because these make up the majority of the genome sequence. Dynamics of LTR-RT accumulation and affinity of LTR-RT to genes is potentially important for further functional characterization, given the significant roles of functional innovation revealed for LTR TE. However, the significant accumulation of nervonic acid in the seeds, is what makes Malania an important species to sequence because of its metabolic uniqueness and potential. We have tried to stress that as well.

3. The title has mentioned that Malania oleifera has great value for nervonic acid production. However, it is surprising that the results about the production of nervonic acid has not been presented in the main text after the introduction.
Responses: Describing the genes involved in the production of nervonic acid was for sure one of our main objectives for this paper. However, without additional molecular genetic experiments (e.g. knockouts of important biosynthetic steps) and proper biochemical product analyses, we cannot identify the rate limiting steps of nervonic acid production in Malania. Therefore, such analysis is beyond the scope of this current work.

4. In this study, many genes related to the VLCFA biosynthesis were identified. Since the transcriptomes of different parts of the plant had been determined, I am curious to know whether these genes are differentially expressed in different body parts and how the expression patterns correlates the VLCFA distribution in the plan.
Responses: We agree with the reviewer that gene expression will for sure provide valuable information on genes or the gene regulatory network related to the VLCFA biosynthesis. However, the focus of the study presented here was on genome assembly and genome annotation, and the RNA-seq data that were generated were primarily used to support gene prediction and annotation. Indeed, the current RNA-seq data generated in the present study are suited for gene annotation, but not very suitable for reliable gene expression analyses, which requires a much more elaborate experimental design. Consequently, these generated RNA-seq data cannot be used in proper gene expression analyses. We believe that the exceptional quality and detail of the genome assembly and TE analysis justify publication. Hopefully, when future funding can be secured (being able to publish the assembly and annotation should increase our chances ...), we hope to be able to perform adequate transcriptome analyses to further study and unravel VLCFA biosynthesis.

Finally, we hope that you will find the revised version meeting the journal standards,

Sincerely Yours,
Jian-Feng Mao

Close
