# Supplementary material for: Genome sequence of Malania oleifera, a tree with great value for nervonic acid production
Source: Gigascience. 2019 Jan 24;8(2):giy164. doi: 10.1093/gigascience/giy164 (PMC6377399; doi:10.1093/gigascience/giy164)
Supplement: Reviewer_1_Report_Original_Submission -- Catherine Jane Nock, Ph.D — 9/3/2018 Reviewed [file giy164_reviewer_1_report_original_submission.pdf]

Reviewer Report

Title: Genome sequence of Malania oleifera, a tree with great value for nervonic acid production

Version: Original Submission **Date:** 9/3/2018

Reviewer name: Catherine Jane Nock, Ph.D.

Reviewer Comments to Author:

This manuscript provides the first genome assembly and genomic resources for the threatened tree Malania oleifera. The manuscript provides extensive novel information for this species and the order Santalales. The extremely high repeat content and low heterozygosity reported are of particular interest, as is the identification of candidate genes in the VLCFA biosynthesis pathway.
1. Despite the species listing on IUCN Red List as vulnerable, the authors incorrectly refer to the species as 'endangered' in the title, keywords and throughout the manuscript. Needs to be corrected to either 'threatened' or 'vulnerable'.
2. Substantial supplementary data is provided with this manuscript, but there is very limited detail beyond which programs were used for the de novo assembly pipelines. More information on the parameters applied is needed, at least for the v0.7 assembly used for scaffolding. In general, limited detail is provided on the analysis methods used in the study.
3. A major concern is the phylogenetic analysis and molecular dating based on single copy genes. Details of the alignment and analysis methods and results are not provided. A strict molecular clock is applied to the concatenated alignment. While there is no report of testing for clocklike evolution, previous evidence suggests that this highly unlikely. Many relationships within the tree are not supported by previous studies but this is not discussed e.g. Vitales (Vitis) + Santalales (Malania) clade, Proteales (Nelumbo) + Ranunculales (Acquilegia) clade rather than successive sister groups diverging after appearance of core eudicots rather than before. What outgroup was used? The >250 mya divergence between Orzya and eudicots is substantially older than previous estimates. What were the clade support values? The divergence dates and relationships inferred are, at best, unreliable.
4. The reported repeat content of the genome (82%) is extremely high and a thorough analysis of the the most abundant repeats (LTR-RT, 58.23% of genome) is presented. If most were the result of ancient amplification how can the high number of intact LTR-RT (Fig S10) and low removal rates and be explained?
Minor Comments
5. Sentence at Line 90-94 is too long and difficult to interpret. Distinction between effects in healthy individuals and those with a genetic defect is unclear. Are the authors suggesting a link between demyelination and obesity?
6. Replace 'Chun et S. Lee' with 'Chun & SK Lee'. Author citation for the species probably unnecessary in abstract.
7. Line 91: 'nerve system' correct to nervous system
8. Line 104-105: clarify what is meant by 'species' endangering mechanism'
9. Line179: report k-mer value of 17 in addition to depth

10. Line 201-202: '71.80% gene completeness as determined by BUSCO assessment (Table S4)' comes before the description of gene annotation methods.
11. Numbering is inconsistent, e.g. Line 141 '51,149,552,938 bases', Line 244 '313.36 million reads'. Suggest using million/billion or Mb/Gb throughout

Level of Interest

Please indicate how interesting you found the manuscript: Choose an item.

Quality of Written English

Please indicate the quality of language in the manuscript: Choose an item.

Declaration of Competing Interests

Please complete a declaration of competing interests, considering the following questions:

- Have you in the past five years received reimbursements, fees, funding, or salary from an organisation that may in any way gain or lose financially from the publication of this manuscript, either now or in the future?
- Do you hold any stocks or shares in an organisation that may in any way gain or lose financially from the publication of this manuscript, either now or in the future?
- Do you hold or are you currently applying for any patents relating to the content of the manuscript?
- Have you received reimbursements, fees, funding, or salary from an organization that holds or has applied for patents relating to the content of the manuscript?
- Do you have any other financial competing interests?
- Do you have any non-financial competing interests in relation to this paper?

If you can answer no to all of the above, write 'I declare that I have no competing interests' below. If your reply is yes to any, please give details below.

I declare that I have no competing interests

I agree to the open peer review policy of the journal. I understand that my name will be included on my report to the authors and, if the manuscript is accepted for publication, my named report including any attachments I upload will be posted on the website along with the authors' responses. I agree for my report to be made available under an Open Access Creative Commons CC-BY license (http://creativecommons.org/licenses/by/4.0/). I understand that any comments which I do not wish to be included in my named report can be included as confidential comments to the editors, which will not be published.

I agree to the open peer review policy of the journal

To further support our reviewers, we have joined with Publons, where you can gain additional credit to further highlight your hard work (see: https://publons.com/journal/530/gigascience). On publication of this paper, your review will be automatically added to Publons, you can then choose whether or not to claim your Publons credit. I understand this statement.

Yes Choose an item.
