# Supplementary material for: Genome sequence of Malania oleifera, a tree with great value for nervonic acid production
Source: Gigascience. 2019 Jan 24;8(2):giy164. doi: 10.1093/gigascience/giy164 (PMC6377399; doi:10.1093/gigascience/giy164)
Supplement: Reviewer_2_Report_Original_Submission -- Stephen Tsui — 9/12/2018 Reviewed [file giy164_reviewer_2_report_original_submission.pdf]

Reviewer Report

Title: Genome sequence of Malania oleifera, a tree with great value for nervonic acid production

Version: Original Submission **Date:** 9/12/2018

Reviewer name: Stephen Tsui

Reviewer Comments to Author:

This article described the genome sequencing of Malania oleifera Chun et S. Lee, a member of the Olacaceae family. A lot of work has been done but more efforts should be paid to highlight the importance of this genome sequence and related resources. The following comments should be helpful for the revision of this piece of work.

1. The quality of the genome is in general high. However, the gene number (24,094) is a bit less than expected because many plants have 40,000 to 60,000 genes. It is not known whether this is related to the parameters used for the gene prediction. Explanation / support about the gene number should be provided.
2. Quite a lot of paragraphs discuss the LTR-RT in the plant. However, relatively less efforts were put on the metabolic uniqueness of this plant. What make this plant to be so important for genome sequencing? The authors should try to explore those unique features of this plant through the genome they generated. Otherwise, the genome data has not been fully utilized.
3. The title has mentioned that Malania oleifera has great value for nervonic acid production. However, it is surprising that the results about the production of nervonic acid has not been presented in the main text after the introduction.
4. In this study, many genes related to the VLCFA biosynthesis were identified. Since the transcriptomes of different parts of the plant had been determined, I am curious to know whether these genes are differentially expressed in different body parts and how the expression patterns correlates the VLCFA distribution in the plan.

Level of Interest

Please indicate how interesting you found the manuscript: Choose an item.

Quality of Written English

Please indicate the quality of language in the manuscript: Choose an item.

Declaration of Competing Interests

Please complete a declaration of competing interests, considering the following questions:

- Have you in the past five years received reimbursements, fees, funding, or salary from an organisation that may in any way gain or lose financially from the publication of this manuscript, either now or in the future?
- Do you hold any stocks or shares in an organisation that may in any way gain or lose financially from the publication of this manuscript, either now or in the future?
- Do you hold or are you currently applying for any patents relating to the content of the manuscript?
- Have you received reimbursements, fees, funding, or salary from an organization that holds or has applied for patents relating to the content of the manuscript?
- Do you have any other financial competing interests?
- Do you have any non-financial competing interests in relation to this paper?

If you can answer no to all of the above, write 'I declare that I have no competing interests' below. If your reply is yes to any, please give details below.

I declare that I have no competing interests.

I agree to the open peer review policy of the journal. I understand that my name will be included on my report to the authors and, if the manuscript is accepted for publication, my named report including any attachments I upload will be posted on the website along with the authors' responses. I agree for my report to be made available under an Open Access Creative Commons CC-BY license (http://creativecommons.org/licenses/by/4.0/). I understand that any comments which I do not wish to be included in my named report can be included as confidential comments to the editors, which will not be published.

I agree to the open peer review policy of the journal

To further support our reviewers, we have joined with Publons, where you can gain additional credit to further highlight your hard work (see: https://publons.com/journal/530/gigascience). On publication of this paper, your review will be automatically added to Publons, you can then choose whether or not to claim your Publons credit. I understand this statement.

Yes Choose an item.
